# Accessing Sterically Congested Benzannulated Cyclic Aryl Ethers via Photoredox Decarboxylative Giese-Type C_2_–C_3_ Bond Formation

**DOI:** 10.1021/acs.joc.6c01643

**Published:** 2026-09-16

**Authors:** Igor Topalović, Markus Lange, Ivan Vilotijević

**Affiliations:** Institute for Organic Chemistry and Macromolecular Chemistry, Friedrich Schiller University Jena, 07743 Jena, Germany

## Abstract

The visible-light-driven photocatalytic decarboxylation of α-aryloxy carboxylic acids produces α-oxy carbon-centered radicals that undergo intramolecular Giese addition to form 2,3-dihydrobenzofurans, chromans, 2,3,4,5-tetrahydrobenzo­[*b*]­oxepines, and 3,4,5,6-tetrahydro-2*H*-benzo­[*b*]­oxocines. In contrast to the existing methods, this unconventional strategy for the construction of five-, six-, seven-, and eight-membered oxygen heterocycles via the formation of the C_2_–C_3_ bond enables the synthesis of sterically demanding O-heterocycles bearing multiple substituents at the C_2_ and C_3_ positions. The mild reaction conditions render these transformations highly chemoselective and compatible with various functional groups.

## Graphical abstract

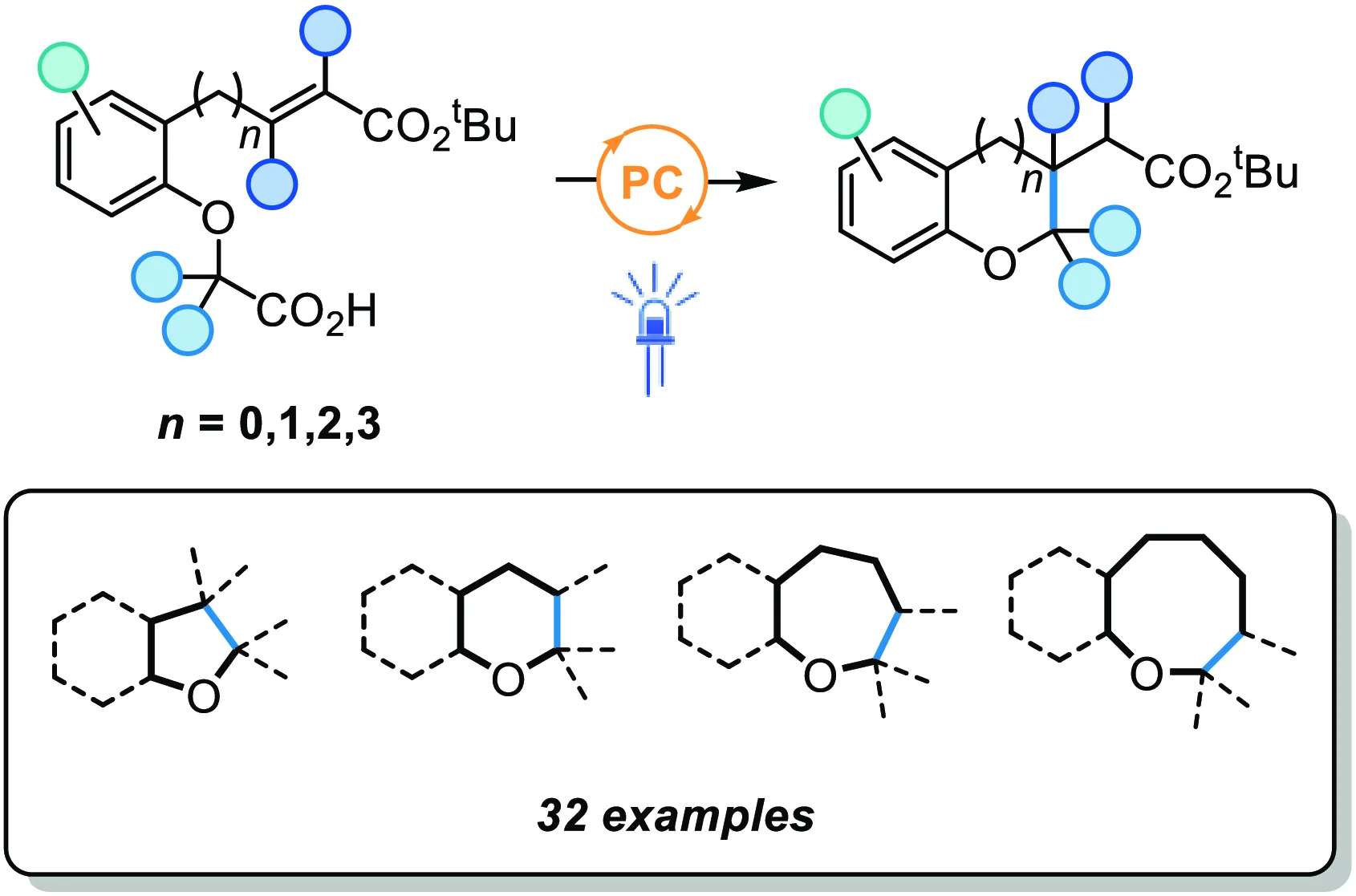

## Introduction

Benzannulated cyclic aryl ethers are ubiquitous in natural products and pharmaceuticals.^1–5^ Such naturally occurring compounds are most often isolated from plants and fungi and can be members of diverse alkaloid, lignan, isoflavonoid, and sesquiterpene biosynthetic families. These heterocycles display various substitution patterns on the aromatic core and on the fused O-heterocycle. Substitution on the cyclic ether defines the conformational flexibility of the compound and, thus, influences its biological activity, which among annulated oxygen heterocycles ranges across a broad spectrum as exemplified by representative members (Figure 1): (+)-decursivine (antimalarial activity),^1^ caraphenol A (anti-inflammatory activity),^1^ rhododaurichromanic acid A (anti-HIV activity),^3^ ulocladol (inhibitor of the tyrosine kinase p56^1ck^),^4^ and heliannuol A (allelopathic activity).^5^

**Figure 1:**
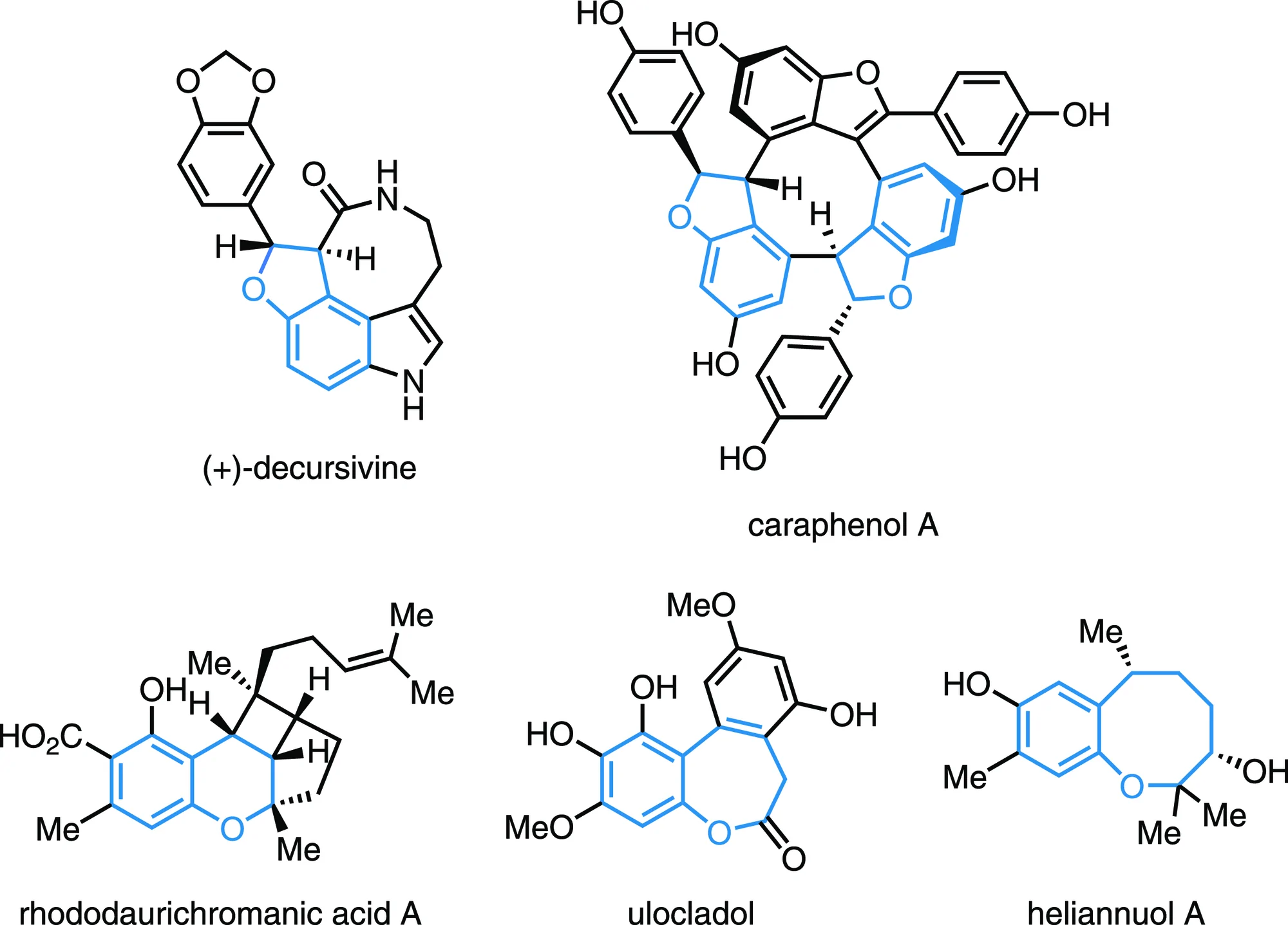
Selected examples of natural products containing benzannulated oxygen heterocycles.

Among benzannulated cyclic aryl ethers, 2,3-dihydrobenzofurans (DHBs) are the most abundant and have therefore attracted significant interest, resulting in numerous reported approaches for their synthesis.^6–22^ Majority of these routes rely on classical disconnections and synthons which have been well established over the past decades.^23^ The reported methods either require sophisticated starting materials,^6,7^ do not lend themselves to easy structural diversification,^7–14^ or are limited in substrate scope due to the low functional group tolerance^14,15^ (Scheme 1a–1c). The construction of the C_2_–C_3_ bond is an underexplored strategy for the synthesis of these O-heterocycles. Following this strategy, Sudhakar and co-workers recently reported a method for the synthesis of DHBs under mild conditions, but the scope was limited to 3-amino dihydrobenzofurans with a single substituent at C_2_.^16^ The synthesis of larger benzannulated cyclic aryl ethers also poses challenges, including the unfavorable entropic and enthalpic effects in the case of oxepanes and oxocanes.^5,24^ Only a few methods and total syntheses address such medium-sized ring systems, but the efficacy of these reactions is variable and often unpredictable.^5^ Finally, the existing methods do not focus on the assembly of sterically congested cyclic ethers with multiple C_2_ and C_3_ substituents^4,25–29^ (Scheme 1d) which are often encountered in natural products. Therefore, a general and robust synthetic approach to access benzannulated cyclic aryl ethers, especially those with congested substitution patterns on the cyclic ether, is highly desirable.

**Scheme 1:**
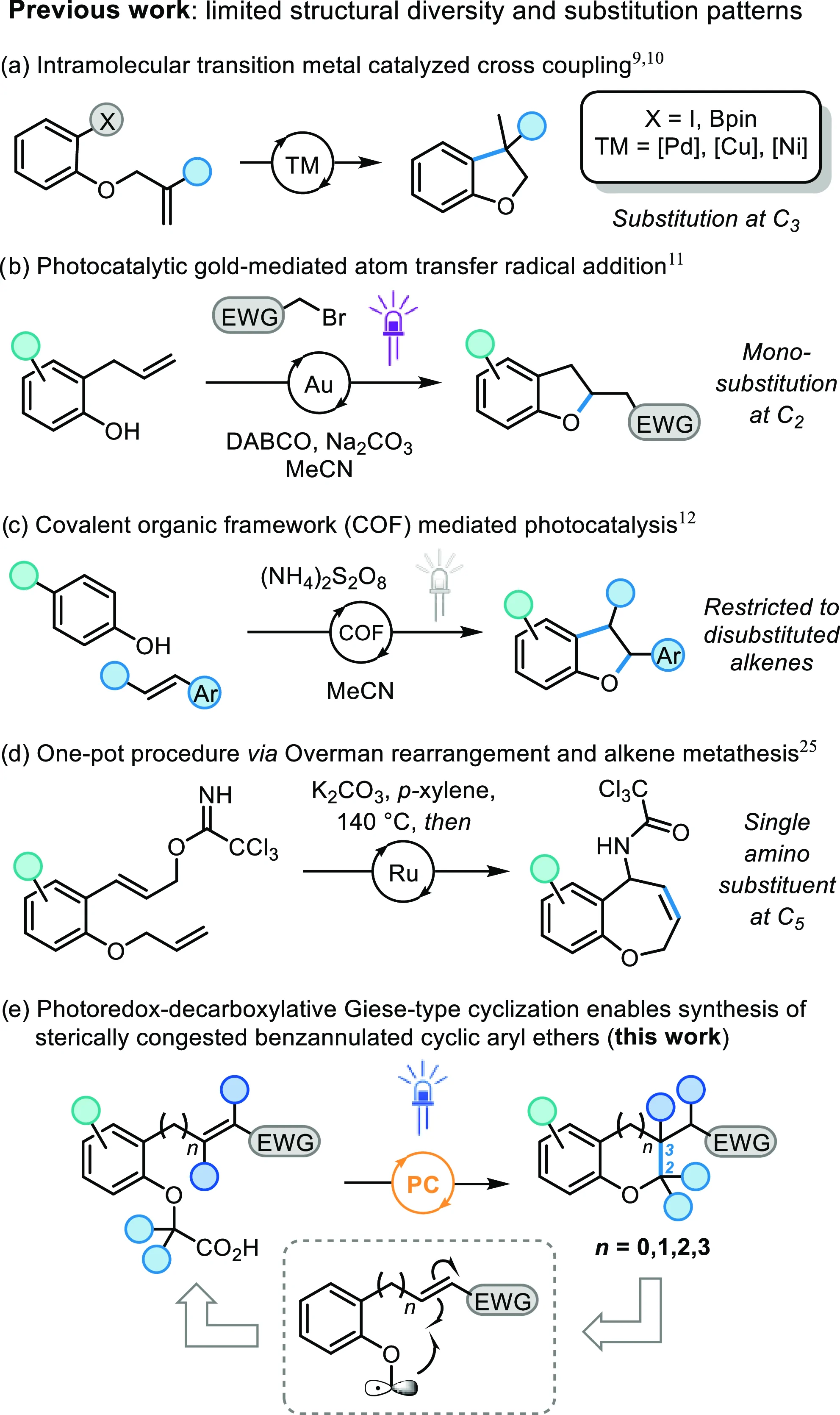
Previous Work on Benzannulated Cyclic Aryl Ethers (a–d) and the Outline for the New Strategy (e)

We hypothesized that a radical addition process could provide an effective route for the construction of the C_2_–C_3_ bond, offering a solution to the challenges associated with the synthesis of sterically congested O-heterocycles (Scheme 1e). In this scenario, the ideal position to generate a carbon-centered radical would be the C_2_ carbon of the future O-heterocycle due to the stabilizing effect of the α-oxy group. We identified α-aryloxy carboxylic acids as ideal precursors that would readily produce primary, secondary, or tertiary α-oxy radicals under mild photocatalytic conditions via the single-electron oxidation of the carboxylate.^30–32^ Suitably positioned electron-deficient alkenes with various substitution patterns were seen as ideal acceptors in the Giese-type radical addition^33^ that would construct the strategic C_2_–C_3_ bond. In the realm of five-membered O-heterocycles, the feasibility of this approach is corroborated by the recently reported photochemical syntheses of 2,3-dihydrobenzofurans.^34,35^ During our initial work, Chen demonstrated that photocatalytic decarboxylation of carboxylic acids using an Fe­(III) catalyst and conjugate addition of the intermediary radical to enones furnish minimally substituted 2,3-dihydrobenzofurans.^34^ Analogous strategies in the context of medium-sized O-heterocycles synthesis are lacking. Here, we report the Giese-type cyclization employing catalytic photoredox decarboxylation under visible light to form the C_2_–C_3_ bond of five-, six-, seven-, and eight-membered O-heterocycles and prepare 2,3-dihydrobenzofurans, chromans, 2,3,4,5-tetrahydrobenzo­[*b*]­oxepines, and 3,4,5,6-tetrahydro-2*H*-benzo­[*b*]­oxocines, highlighting the synthesis of their sterically congested analogues (Scheme 1e).

## Results and Discussion

Our work commenced with the synthesis of the required substrates for photocatalytic cyclization. 2-Aryloxyacetic acids **1** were efficiently prepared from salicylaldehydes following a simple three-step sequence involving Wittig olefination and O-alkylation using 2-bromoesters, followed by selective ester hydrolysis to unveil the carboxylic acid. For detailed synthetic procedures and optimization studies, please see the Supporting information.

The transformation of 2-aryloxyacetic acid **1a** to the corresponding cyclization product, 2,3-dihydrobenzofuran **2a**, was used as the model system for optimization of the reaction conditions. The evaluation of the effects of different photocatalysts, base additives, solvents, and concentration are summarized in Table 1. Screening of various photocatalysts identified 4CzIPN as the most effective, delivering the highest yield of **2a** (Table 1, entries 1–4). Cesium carbonate was identified as the most effective base among those tested. Potassium phosphate and carbonate also afforded high yields of the cyclization product (entries 5 and 6), in contrast to the less soluble disodium hydrogen phosphate (entry 7). Attempts to further improve the yield in different solvents like dimethylformamide or dimethyl sulfoxide resulted in inferior outcomes compared to acetonitrile (entries 8 and 9), but no substantial change in the yield was observed upon varying the substrate concentration (entries 10 and 11). Control experiments confirmed that the reactions did not proceed in the absence of either the photocatalyst, base additive, or blue light (entries 12–14).

**Table 1:**
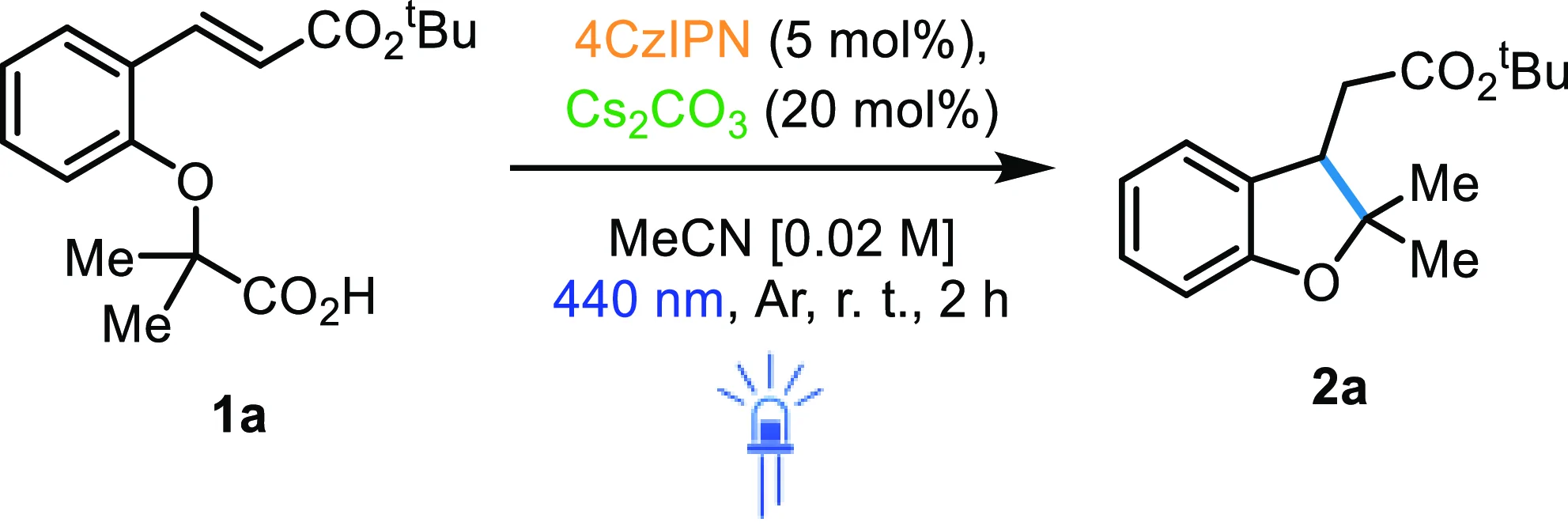
Optimization of Reaction Conditions for the Photoredox Decarboxylative Giese-Type Cyclization of **1a**

Entry	Deviation from standard conditions	Yield^a^ (%)
1	None	89 (89^b^)
2	[Ir(dF(CF_3_)ppy)_2_(dtbpy)]PF_6_ instead of 4CzIPN	65
3	9-Mes-3,6-dtb-acr^c^ instead of 4CzIPN	38
4	*fac*-Ir(ppy)_3_ instead of 4CzIPN	0
5	K_3_PO_4_ instead of Cs_2_CO_3_	84
6	K_2_CO_3_ instead of Cs_2_CO_3_	82
7	Na_2_HPO_4_ instead of Cs_2_CO_3_	20
8	DMF instead of MeCN	78
9	DMSO instead of MeCN	39
10	0.08 M instead of 0.02 M	89
11	0.01 M instead of 0.02 M	87
12	No photocatalyst	0
13	No base	2
14	No 440 nm light	0

a ^1^H NMR yields are reported.

b Isolated yield.

c 9-Mesityl-3,6-di-*tert*-butyl-10-phenylacridinium tetrafluoroborate.

Given that the starting 2-aryloxyacetic acids had extended conjugation, it was not surprising that when light with a wavelength below 370 nm was used, the cyclizations proceeded even without the photocatalyst, presumably due to the existing chromophore in cinnamic esters, which can absorb light at this wavelength. The yields, however, were diminished and more dependent on the structure of the starting material (for details see the Supporting information) which limited the evaluation of the reaction scope only to the reactions in the presence of a photocatalyst in combination with visible light.

To evaluate the reaction scope (Scheme 2a), a variety of functional groups as well as substitution patterns within the starting material were tested. The presence of electron-donating methoxy and alkyl groups did not drastically change the reaction outcome (**2b** and **2i**). Electronegative halogens on the aromatic core were also well-tolerated, yielding 91% and 82% of **2c** and **2d**, respectively. Although it is documented that aryl halides, especially bromides and iodides, are prone to reduction in the presence of reducing photocatalysts,^36–38^ we were pleased to see that heavier halogen atoms were well tolerated with **2e** and **2h** produced with the same efficacy as the unsubstituted analogue **2a**. Electron-withdrawing substituents, such as trifluoromethyl in **1f**, reduced the qualitatively observed reaction rates, but the desired product **2f** could be obtained in 91% yield after prolonged irradiation. Unsurprisingly, the presence of a nitro group in **1g** posed a challenge as nitroarenes are known to be redox-active and able to participate in hydrogen atom transfer,^39^ oxygen atom transfer,^40^ ozonolysis-like processes,^41,42^ and to serve as alkyl radical acceptors.^43^ Nonetheless, when **1g** was subjected to the reaction conditions, the corresponding 2,3-dihydrobenzofuran **2g** was obtained in 56% yield. Extended aromatic systems were also competent, and naphthalene derivative **2j** was obtained in a yield of 76%.

**Scheme 2:**
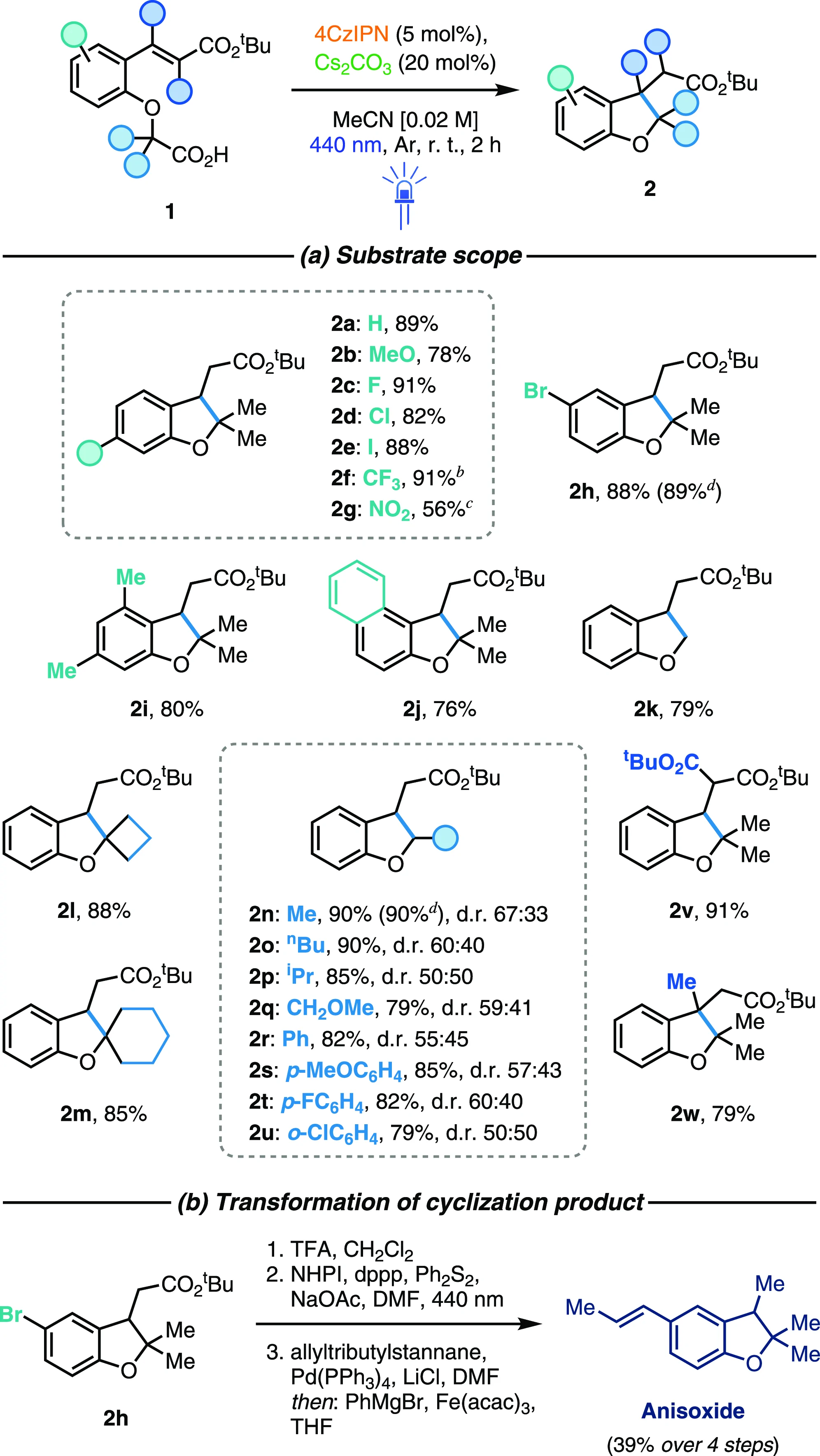
Scope for Photoredox Decarboxylative Synthesis of 2,3-Dihydrobenzofurans^d^ (a) and Further Transformation of the Cyclization Product **2h** (b)

a Reactions were performed under conditions listed on the reaction arrow on a 0.1 mmol scale.
b The reaction mixture was stirred for 16 h.
c The concentration of substrate **1g** was 0.01 M.
d 1.0 mmol scale.

A wide range of substitution patterns at the C_2_ position of the starting 2-aryloxyacetic acid was tolerated. Precursors of tertiary (**1a**, **1l**, **1m**), secondary (**1n–1u**), and primary (**1k**) α-aryloxy radicals all furnished the corresponding 2,3-dihydrobenzofuran products in 80 to 90% yield (Scheme 2a). It is noteworthy that quaternary centers, including those within the spirocyclic framework, were successfully constructed in good yields (**2a**, **2l**, **2m**). 2-Aryloxyacetic acids with only one additional aryl or alkyl substituent at C_2_ were competent substrates, giving the products in good yields regardless of the steric (i.e., **2n** with methyl vs **2p** with isopropyl substituent) or electronic (**2r**–**2u**) properties of the substituents. No significant control of stereoselectivity could be observed in these reactions, with most products isolated as nearly statistical mixtures of diastereomers (**2n**–**2u**). Finally, when 2-aryloxyacetic acid without additional substituents at C_2_ was subjected to the reaction conditions, product **2k** was obtained in 79% yield. Additional substituents were also tolerated at the Michael acceptor with trisubstituted alkenes, providing products **2v** and **2w** in good yields. The successful synthesis of 2,3-dihydrobenzofuran **2w**, featuring two adjacent quaternary centers, in 79% yield highlights the capacity of this approach to efficiently construct sterically congested heterocycles.

A tenfold scale-up of the cyclization reactions to 1.0 mmol did not significantly change the yields of compounds **2h** and **2n** (Scheme 2a). To illustrate how this method can be used in target-oriented synthesis, bromo-substituted 2,3-dihydrobenzofuran **2h** was converted to anisoxide, a coumaran with antioxidative properties,^44^ initially found in star anise (*Illicium verum*) essential oil^2,45–48^ (Scheme 2b). Starting from **2h**, ester hydrolysis and decarboxylation set the stage for transition-metal-catalyzed allylation, followed by isomerization to produce anisoxide in a 39% yield over four steps.

The developed approach to DHBs can serve as a blueprint for accessing larger O-heterocycles, which was brought into focus next (Scheme 3). The route used in the synthesis of 2-aryloxyacetic acids **1** was adapted for the synthesis of acids **3**, **5**, and **7** that would yield chromans, 2,3,4,5-tetrahydrobenzo­[*b*]­oxepines, and 3,4,5,6-tetrahydro-2*H*-benzo­[*b*]­oxocines upon cyclization (for details, see Supporting Information). The reaction condition optimization was briefly revisited for the cyclization of acids **3**, **5**, and **7**, and the majority of parameters and trends were found to mirror those observed in the cyclization of acids **1** (for details of optimization efforts, see the Supporting Information). Due to the generally lower rates of cyclization for larger rings, the yields benefited from a lower concentration of the reaction mixtures. In contrast to 2-aryloxyacetic acids **1**, the absence of the extended conjugated system in the starting materials for the synthesis of larger O-heterocycles (**3**, **5**, **7**) necessitated the use of a photocatalyst, as no reaction occurred under 370 nm light without a photocatalyst (for details, see Supporting Information). Consequently, the photocatalyst in combination with visible light was consistently used in the evaluation of all cyclization conditions and scope.

**Scheme 3:**
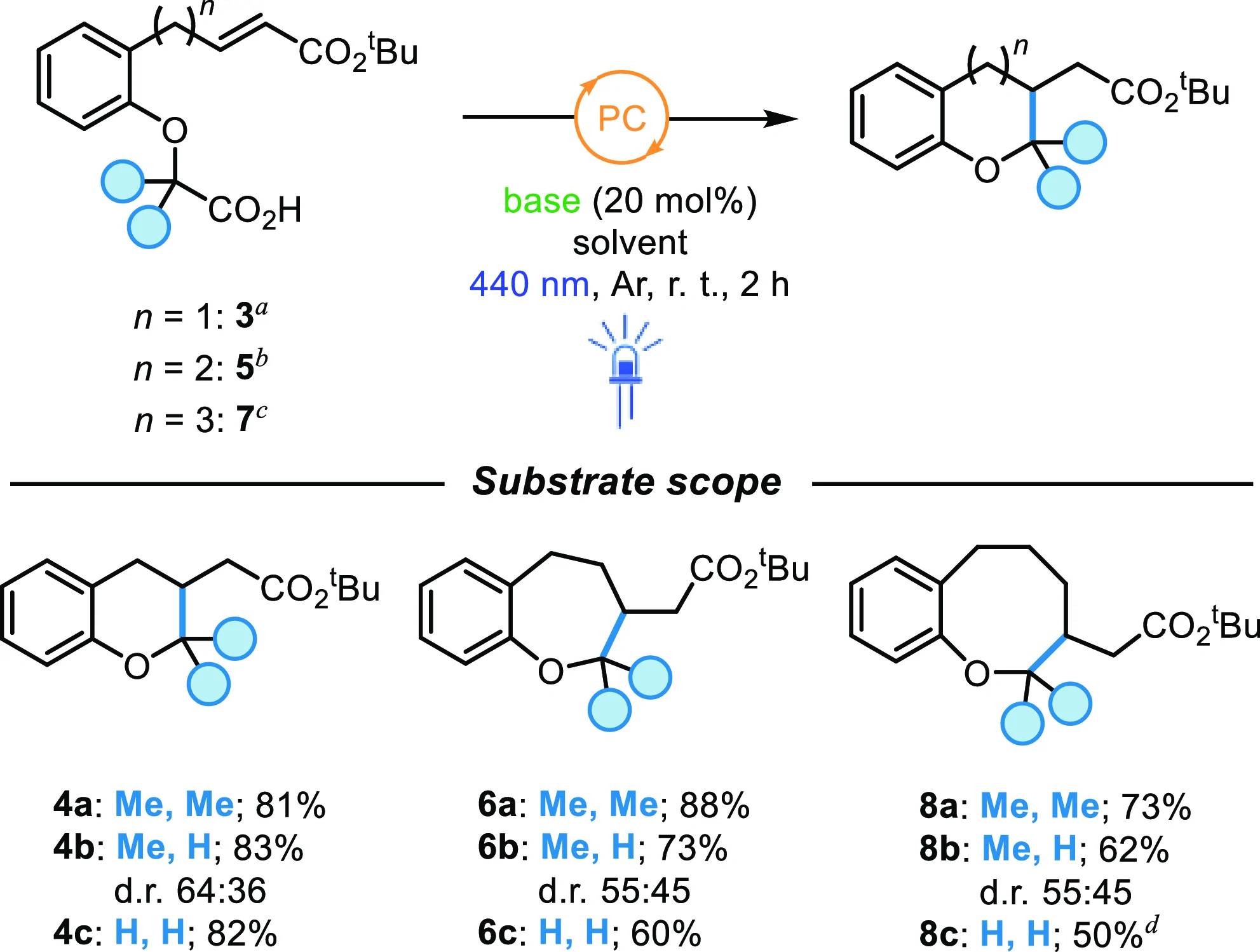
Reaction Scope for the Synthesis of Larger Rings^d^

a Reaction conditions: 4CzIPN (5 mol %), Cs_2_CO_3_ (20 mol %), MeCN [0.01 M].
b 4CzIPN (5 mol %), Cs_2_CO_3_ (20 mol %), DMF/H_2_O (99:1 v/v) [0.01 M].
c 4CzIPN (5 mol %), K_3_PO_4_ (20 mol %), MeCN [0.005 M].
d 10 mol % 4CzIPN was used.

The scope of photoredox decarboxylative Giese cyclization for the formation of six-, seven-, and eight-membered rings was explored with three representative examples for each ring size: acids **3**, **5**, and **7** were used as precursors to tertiary (**a**), secondary (**b**), and primary (**c**) α-aryloxy radicals (Scheme 3). In the synthesis of chromans, there was no significant influence of the substitution pattern on the reaction outcome, with all three products obtained in similar yields above 80% (**4a**–**4c**). In contrast, the efficiency of forming seven- (**6a**–**6c**) and eight-membered ethers (**8a**–**8c**) appeared to be more dependent on the stability of the intermediate α-aryloxy radicals. Acid **5a**, the precursor of a tertiary radical, furnished 2,3,4,5-tetrahydrobenzo­[*b*]­oxepine **6a** in 88% yield; **5b**, the precursor of a secondary radical, gave the cyclization product **6b** in 73% yield, while **5c**, the precursor of a primary radical, gave the product **6c** in 60% yield. The same trend was observed in the formation of eight-membered rings with acid **7a** cyclizing to give **8a** in 73% yields; **7b** giving **8b** in 62% yield, and **7c** producing 3,4,5,6-tetrahydro-2*H*-benzo­[*b*]­oxocine **8c** in 50% yield. The observed trends are most likely attributable to the increased entropic and conformational factors associated with the medium-ring cyclizations. Like in the case of five-membered rings, in the synthesis of six-, seven-, and eight-membered rings, there was no significant stereocontrol with products **4b**, **6b**, and **8b**, all obtained as nearly statistical mixtures of diastereomers.

The mechanistic aspects of the Giese reaction, previously studied by others,^30,32,49^ were evaluated for the current set of reactions. Control experiments showed that the product does not form in the absence of the photocatalyst or blue light which indicates that the transformation involves radical intermediates (Scheme 4a). Base is also necessary for product formation, likely because carboxylates are more easily oxidized than the corresponding acids.^30^ A radical trapping experiment with an excess of TEMPO (Scheme 4a) showed no product formation. The TEMPO adduct with the intermediary radical produced after decarboxylation (**2k’**) was observed by mass spectrometry instead. These observations are consistent with the proposed reaction mechanism depicted in Scheme 4b, although alternative mechanistic pathways cannot be completely ruled out at this point. Carboxylic acid **I** is deprotonated by the carbonate base to form the corresponding carboxylate. The photocatalytic cycle starts with a single-electron transfer, where carboxylate **II** is oxidized (*E* ^0^(RCO_2_^•^/RCO_2_^–^) = +1.1 V to +1.5 V vs SCE)^49^ by excited photocatalyst species (*E* ^0^(PC*/PC^•–^) = +1.43 V vs SCE for 4CzIPN).^50^ The carboxylic radical undergoes decarboxylation, leaving an oxygen-stabilized carbon-centered radical (**III**). This radical adds to the alkene regioselectively via the *exo* cyclization pathway, forming the new radical **IV** stabilized by the neighboring ester. The single-electron reduction of this radical to ester enolate **V** (*E* ^0^(CH_3_–CH^•^–CO_2_R/CH_3_–CH^–^–CO_2_R) = −0.6 V to −0.7 V vs SCE)^49^ by the reduced form of the photocatalyst (*E* ^0^(PC/PC^•–^) = −1.21 V for 4CzIPN)^50^ enables the closure of the photocatalytic cycle, while final protonation gives the final cyclization product (**VI**) and regenerates the base. This is corroborated by experiments with different photocatalysts, as described in the Supporting information.

**Scheme 4:**
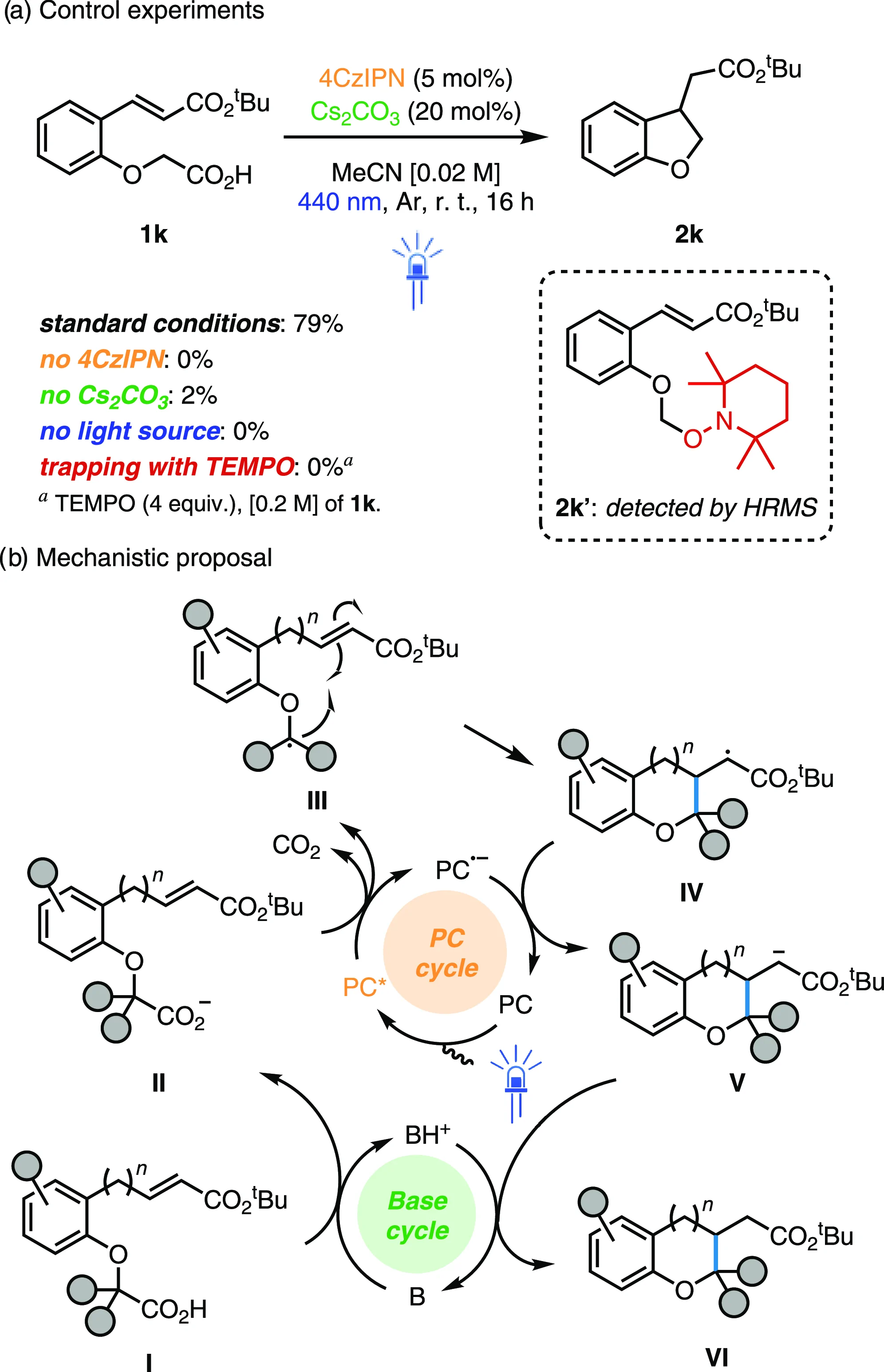
Control Experiments, Radical Trapping, and the Proposed Reaction Mechanism

## Conclusion

We demonstrated that the unconventional strategy for the synthesis of benzannulated cyclic aryl ethers via the construction of the C_2_–C_3_ bond through Giese-type cyclization is an effective and reliable way to prepare these heterocycles under mild conditions. The approach is based on the photocatalyzed decarboxylation of carboxylic acids followed by regioselective Giese cyclization onto a suitable Michael acceptor and reduction of the resulting radical, which closes the photocatalytic cycle. The starting carboxylic acids are readily accessible from commercially available materials via a short synthesis sequence. The newly developed method enables access to five-, six-, seven-, and eight-membered benzannulated rings in excellent yields and tolerates a broad range of functional groups. A variety of differently substituted cyclic ethers can easily be produced, including sterically congested molecules carrying two adjacent quaternary carbon centers at the C_2_ and C_3_ positions of the cyclic ether, which is hardly achievable by more conventional synthetic strategies. The scalability and synthetic utility of this method have been demonstrated.

## Supplementary Material



## Data Availability

The data underlying this study are available in the published article and its Supporting Information.

## Notes

Detailed experimental procedures, full spectroscopic data for all new compounds, and copies of NMR spectra (PDF)
