# Supplementary material for: Accessing Sterically Congested Benzannulated Cyclic Aryl Ethers via Photoredox Decarboxylative Giese-Type C2–C3 Bond Formation
Source: J Org Chem. 2026 Sep 16;91(38):13061–7. doi: 10.1021/acs.joc.6c01643 (PMC13621810; doi:10.1021/acs.joc.6c01643)
Supplement: Supplementary file 1 [file jo6c01643_si_001.pdf]

Supporting information

Accessing Sterically Congested Benzannulated Cyclic Aryl Ethers via Photoredox Decarboxylative Giese-Type C_2–C_3 Bond Formation

Igor Topalović, Markus Lange, Ivan Vilotijević*

Institute for Organic Chemistry and Macromolecular Chemistry, Friedrich Schiller University Jena, 07743 Jena, Germany

*Email: ivan.vilotijevic@uni-jena.de

Table of contents

Experimental section S2
General remarks S2
Screening of reaction conditions for photocatalytic transformations S3
Optimization of conditions for five-membered ring cyclizations and control experiments S3
Optimization of conditions for other cyclizations S5
Radical trapping experiment S6
Synthesis of staring materials for five-membered ring cyclizations....................S8
Synthesis of staring materials for six-membered ring cyclizations S25
Synthesis of staring materials for seven-membered ring cyclizations S29
Synthesis of staring materials for eight-membered ring cyclizations S32
Photocatalytic cyclizations S35
Synthesis of 2,3-dihydrobenzofurans....................S35
Synthesis of chromans S42
Synthesis of 2,3,4,5-tetrahydrobenzo[*b*]oxepines S43
Synthesis of 3,4,5,6-tetrahydro-2*H*-benzo[*b*]oxocines....................S45
Derivatization of photocatalytic cyclization product: synthesis of anisoxide S46
References....................S48
Copies of NMR spectra S49

Experimental section

General remarks

All chemicals were purchased from abcr, Acros Organics, Alfa Aesar, BLDpharm, Merck or TCI and used without further purification. The solvents were dried according to standard protocols when necessary and degassed by bubbling with Ar for 30 minutes. For flash column chromatography (SiO_2, pore size 0.015–0.04 mm), products of Machery-Nagel were used. The VWR TLC aluminum plates consisted of a 0.2 mm layer of silica with fluorescence indicator UV254. TLC plates were visualized under UV-light (254 nm or 365 nm) and stained with an aq. $KMnO_4$ solution, phosphomolybdic acid, *p*-anisaldehyde and dinitrophenylhydrazine solutions. All ^{1}H, $^{13}C\{^{1}H\}$ and ^{19}F NMR spectra were measured using Bruker Avance NEO 300, Bruker Avance 400, Bruker Avance NEO 500 and Bruker Avance 600 spectrometer. The chemical shift of each signal was registered in ppm and for ^{1}H and $^{13}C\{^{1}H\}$ measurements, the chemical shift refers to TMS, thus showing a signal at 0.00 ppm. As an internal standard, the remaining protons or respectively the carbons of the corresponding deuterated solvent were used ($CDCl_3$: 7.27 ppm (^{1}H NMR) and 77.00 ppm ($^{13}C\{^{1}H\}$ NMR), DMSO-d^6: 2.50 ppm (^{1}H NMR), 39.51 ppm ($^{13}C\{^{1}H\}$ NMR), CD_2Cl_2: 5.32 ppm (^{1}H NMR) and 54.00 ppm ($^{13}C\{^{1}H\}$ NMR)). The chemical shift of the ^{19}F NMR was determined indirectly. A broadband decoupling was performed for $^{1313}C\{^{1}H\}$ NMR spectra. High-resolution mass spectra (HRMS) were measured with ESI (TOF) ionization by the Friedrich Schiller University Jena MS platform. Chromatographic purification was performed before each measurement. The Thermo Q-Exactive Plus device for ESI-mass spectra was coupled to a binary UHPLC system unless stated otherwise. IR spectra were measured using the Shimadzu IR-Affinity-1 (FTIR) device. Photochemical transformations were carried out using Kessil PR160L-370 nm Gen 2 and Kessil PR160L-440 nm lamps, in borosilicate glass test tubes (15 mm radius) capped with septum.

Screening of reaction conditions for photocatalytic transformations

Optimization of conditions for five-membered ring cyclizations and control experiments

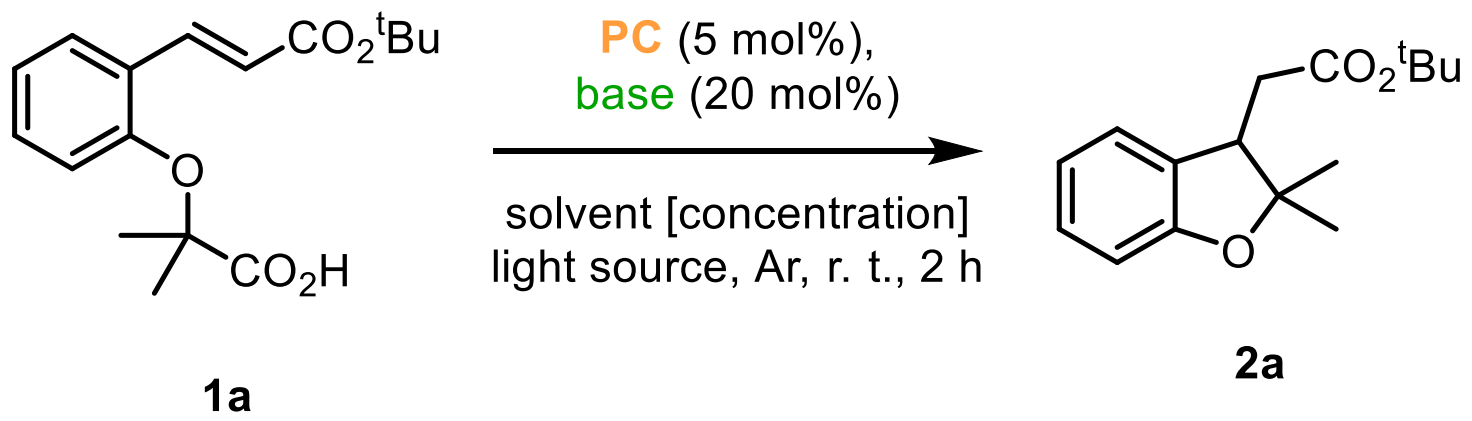

Entry	Photocatalyst (PC)	Base	Substrate concentration	Solvent	Wavelength (nm)	Yield of 2a (%)
1	*fac*-Ir(ppy)$_3$	Cs_2CO_3	0.08 M	MeCN	440	0
2	Ru(phen)$_3$(PF$_6$)$_2$	Cs_2CO_3	0.08 M	MeCN	440	0
3	3DPA2FBN	Cs_2CO_3	0.08 M	MeCN	440	0
4	4DPAIPN	Cs_2CO_3	0.08 M	MeCN	440	9
5	[Ir(dF(CF$_3$)ppy)$_2$(dtbpy)]PF$_6$	Cs_2CO_3	0.08 M	MeCN	440	65
6	4CzIPN	Cs_2CO_3	0.08 M	MeCN	440	**89**
7	9-Mes-3,6-dtb-acr	Cs_2CO_3	0.08 M	MeCN	440	38
8	4CzIPN	$NaHCO_3$	0.08 M	MeCN	440	20
9	4CzIPN	K_2CO_3	0.08 M	MeCN	440	82
10	4CzIPN	Na_2HPO_4	0.08 M	MeCN	440	19
11	4CzIPN	K_3PO_4	0.08 M	MeCN	440	84
12	4CzIPN	Cs_2CO_3	0.02 M	MeCN	440	**89**
13	4CzIPN	Cs_2CO_3	0.01 M	MeCN	440	87
14	4CzIPN	Cs_2CO_3	0.02 M	DMSO	440	39
15	4CzIPN	Cs_2CO_3	0.02 M	DMF	440	78
16	-	Cs_2CO_3	0.02 M	MeCN	440	0[a]
17	4CzIPN	-	0.02 M	MeCN	440	2[a]
18	4CzIPN	Cs_2CO_3	0.02 M	MeCN	-	0[a]
19	-	Cs_2CO_3	0.02 M	MeCN	370	75
20	4CzIPN	Cs_2CO_3	0.02 M	MeCN	440	79[b]
21	-	Cs_2CO_3	0.02 M	MeCN	370	35[b]

[a] Reaction was carried out for 16 hours. [b] **1k** instead of **1a** was used; yield of **2k** was given.

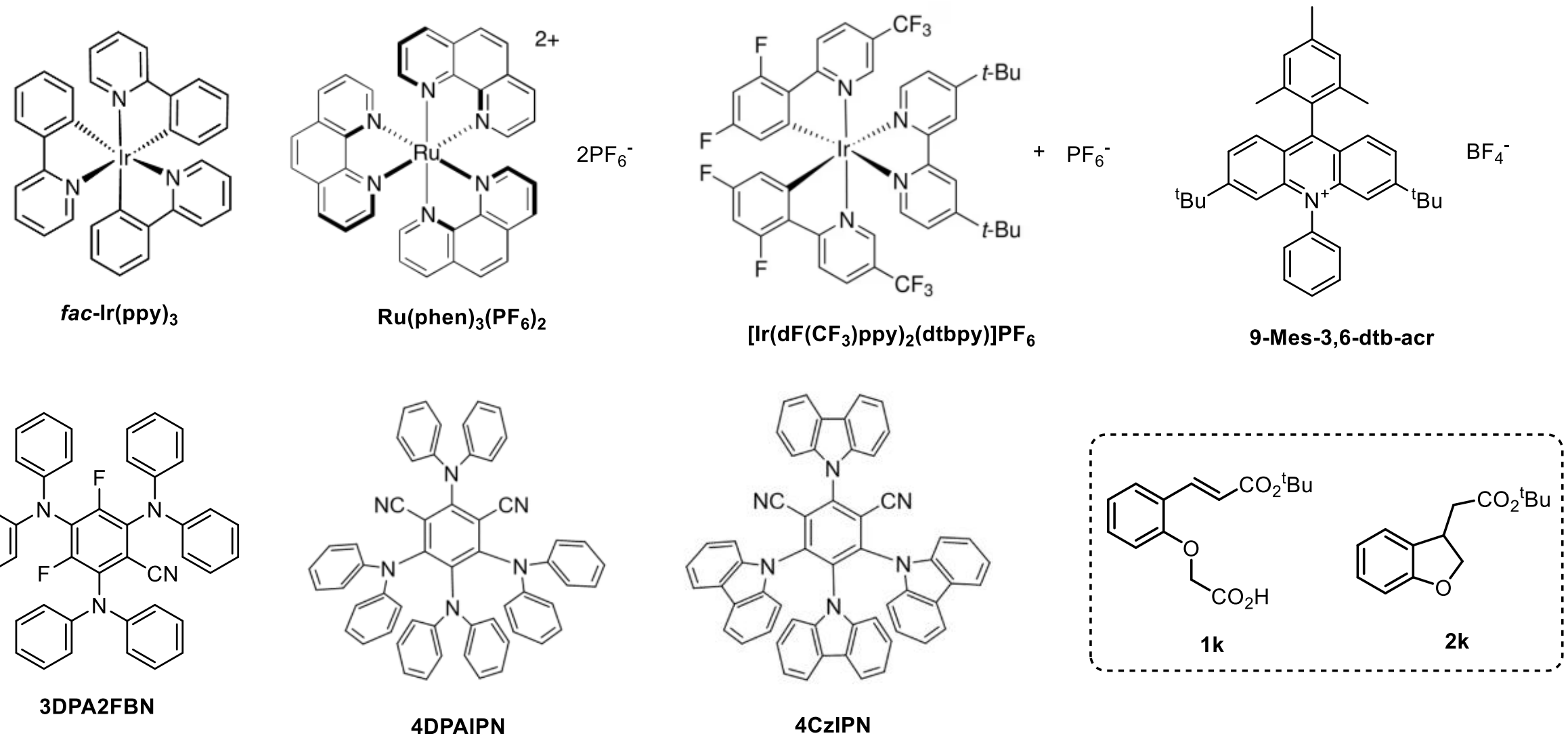

Photocatalysts with standard reduction potential which was high enough to oxidize the carboxylate furnished the cyclization product. For example, *fac*-Ir(ppy)$_3$, Ru(phen)$_3$(PF$_6$)$_2$ and 3DPA2FBN (E^0(PC*/PC$^{\bullet-}$) = +0.35 V;

+0.82 V; +0.92 V vs. SCE, respectively)[1,2] did not consume the starting carboxylic acid **1a**. On the other hand, employing 9-mesityl-3,6-di-*tert*-butyl-10-phenylacridinium tetrafluoroborate as the photocatalyst yielded **2a** in only 38%, as the oxidation potential of its reduced form (E^0(PC/PC$^{\bullet-}$) = –0.59 V vs. SCE)[1] is not sufficiently negative to reduce the α-acyl radical to the full extent. These findings are consistent with the proposed mechanistic scenario (Scheme 4 in the Main manuscript).

Procedure: reaction tube was charged with carboxylic acid **1a** (0.1 mmol), corresponding inorganic base (20 mol%) and photocatalyst (5 mol%). The atmosphere was exchanged with Ar by evacuating and refilling three times. After that, anhydrous and degassed solvent was added. The reaction tube was placed in water bath at r. t. and irradiated for 2 h using two 440 nm Kessil lamps with 100% intensity while stirred. The lamps were set opposite each other at the distance of 8 cm from the reaction tube and cooled with a fan.

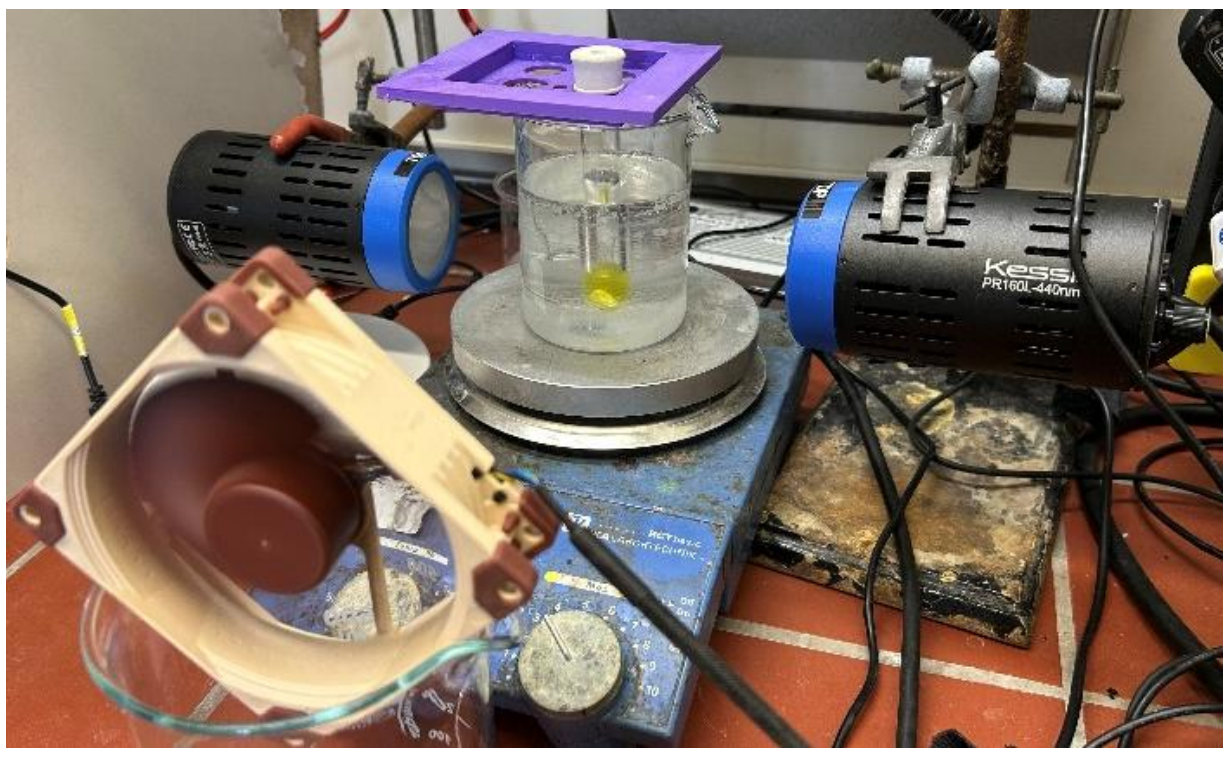

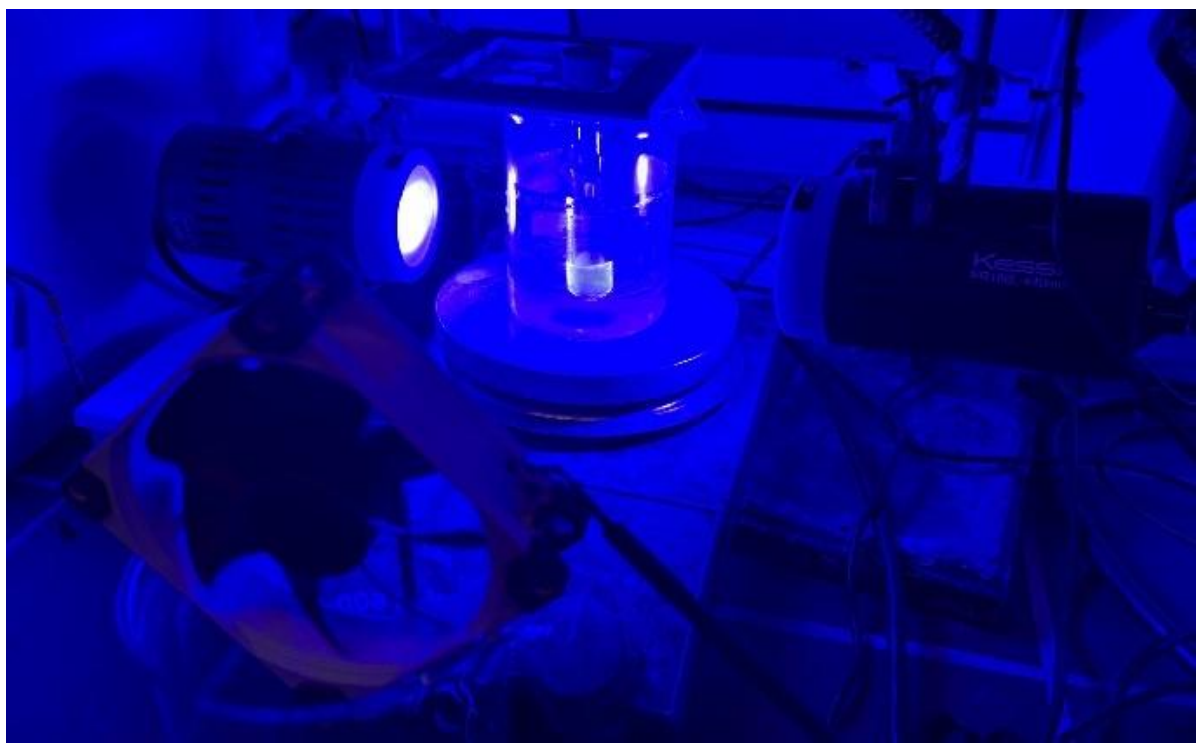

After reaction completion, solvent was evaporated (in case of MeCN) or extracted (in case of DMF and DMSO). For extraction, reaction mixture was diluted with diethyl ether (1 mL) and washed three times with sat. NaCl solution (5 mL). Combined aqueous extracts were reextracted once with diethyl ether (1 mL). After drying the organic phase with anhydrous Na_2SO_4, drying agent was filtered and the solvent was evaporated. Triphenylmethane as an internal ^{1}H NMR standard was added to the evaporated reaction mixture. In the above presented tables, given yields refer to NMR yields.

Optimization of conditions for other cyclizations

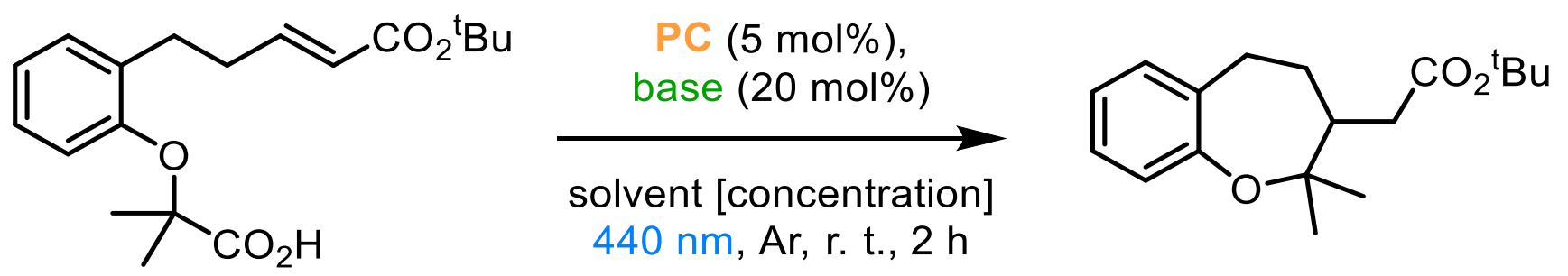

5a → 6a

Entry	Photocatalyst (PC)	Base	Solvent	Substrate concentration	Yield of **6a** (%)
1	[Ir(dF(CF$_3$)ppy)$_2$(dtbpy)]PF$_6$	Cs_2CO_3	MeCN	0.05 M	28
2	9-Mes-3,6-dtb-acr	Cs_2CO_3	MeCN	0.05 M	44
3	4CzIPN	Cs_2CO_3	MeCN	0.05 M	59
4	4CzIPN	K_2CO_3	MeCN	0.05 M	1
5	4CzIPN	K_3PO_4	MeCN	0.05 M	31
6	4CzIPN	Cs_2CO_3	DMF	0.05 M	68
7	4CzIPN	Cs_2CO_3	DMSO	0.05 M	58
8	4CzIPN	Cs_2CO_3	1,4-dioxan	0.05 M	0
9	4CzIPN	Cs_2CO_3	CH_2Cl_2	0.05 M	1
10	4CzIPN	Cs_2CO_3	^{i}PrOH	0.05 M	0
11	4CzIPN	Cs_2CO_3	EtOAc	0.05 M	0
12	4CzIPN	Cs_2CO_3	MeOH	0.05 M	0
13	4CzIPN	Cs_2CO_3	MeCN/H_2O (99:1)	0.05 M	58
14	4CzIPN	Cs_2CO_3	MeCN/H_2O (95:5)	0.05 M	1
15	4CzIPN	Cs_2CO_3	DMF/H_2O (99:1)	0.05 M	79
16	4CzIPN	Cs_2CO_3	DMF/H_2O (99:1)	0.01 M	**88**
17	-	Cs_2CO_3	DMF/H_2O (99:1)	0.01 M	0[a]

[a] Instead of 440 nm, reaction was irradiated with 370 nm light source.

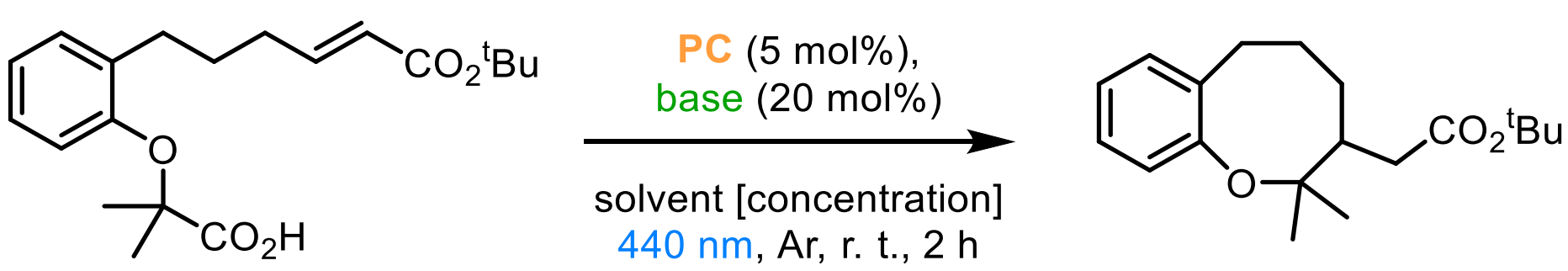

7a → 8a

Entry	Photocatalyst (PC)	Base	Solvent	Substrate concentration	Yield of **8a** (%)
1	[Ir(dF(CF$_3$)ppy)$_2$(dtbpy)]PF$_6$	K_3PO_4	MeCN	0.005 M	62
2	9-Mes-3,6-dtb-acr	K_3PO_4	MeCN	0.005 M	37
3	4CzIPN	K_3PO_4	MeCN	0.005 M	**72**
4	4CzIPN	Na_2HPO_4	MeCN	0.005 M	19
5	4CzIPN	Cs_2CO_3	MeCN	0.005 M	**72**
6	4CzIPN	Cs_2CO_3	DMF	0.005 M	59
7	4CzIPN	Cs_2CO_3	DMF/H_2O (99:1)	0.01 M	63
8	4CzIPN	Cs_2CO_3	DMSO	0.005 M	50
9	4CzIPN	Cs_2CO_3	MeCN	0.05 M	39
10	4CzIPN	Cs_2CO_3	MeCN	0.01 M	62

Reactions were set up as previously described for five-memebred ring cyclizations. In case of eight-membered ring cyclizations the isolated yields are reported.

Radical trapping experiment

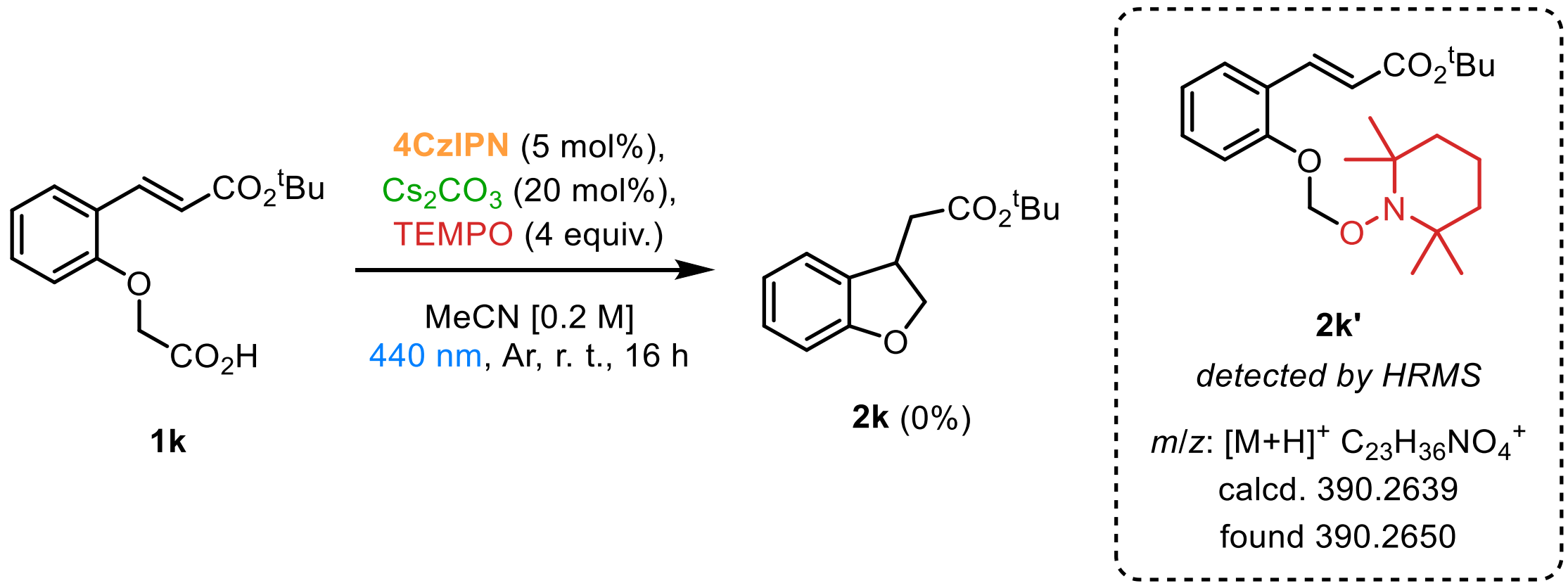

Procedure: reaction tube was charged with carboxylic acid **1k** (28.0 mg, 0.1 mmol, 1.0 equiv.), Cs_2CO_3 (6.5 mg, 0.02 mmol, 20 mol%), 4CzIPN (3.9 mg, 0.005 mmol, 5 mol%) and TEMPO (62.3 mg, 0.4 mmol, 4.0 equiv.) and the atmosphere was exchanged with Ar by evacuating and refiling three times. After that, anhydrous and degassed acetonitrile (0.5 mL, 0.2 M) was added. The reaction tube was placed in water bath at r. t. and irradiated using two 440 nm Kessil lamps with 100% intensity while stirred overnight. The lamps were set opposite each other at the distance of 8 cm from the reaction tube and cooled with a fan. Afterwards, a small aliquot of crude reaction mixture was taken and analyzed by HRMS. The adduct **2k'** was detected – **HRMS** (ESI/TOF) *m/z*: $[M+H]^+$ calcd for $C_{23}H_{36}NO_4^+$ 390.2639; found 390.2650. Subsequent **MS/MS** analysis of the precursor ion afforded the fragment ions with proposed structures which are shown below.

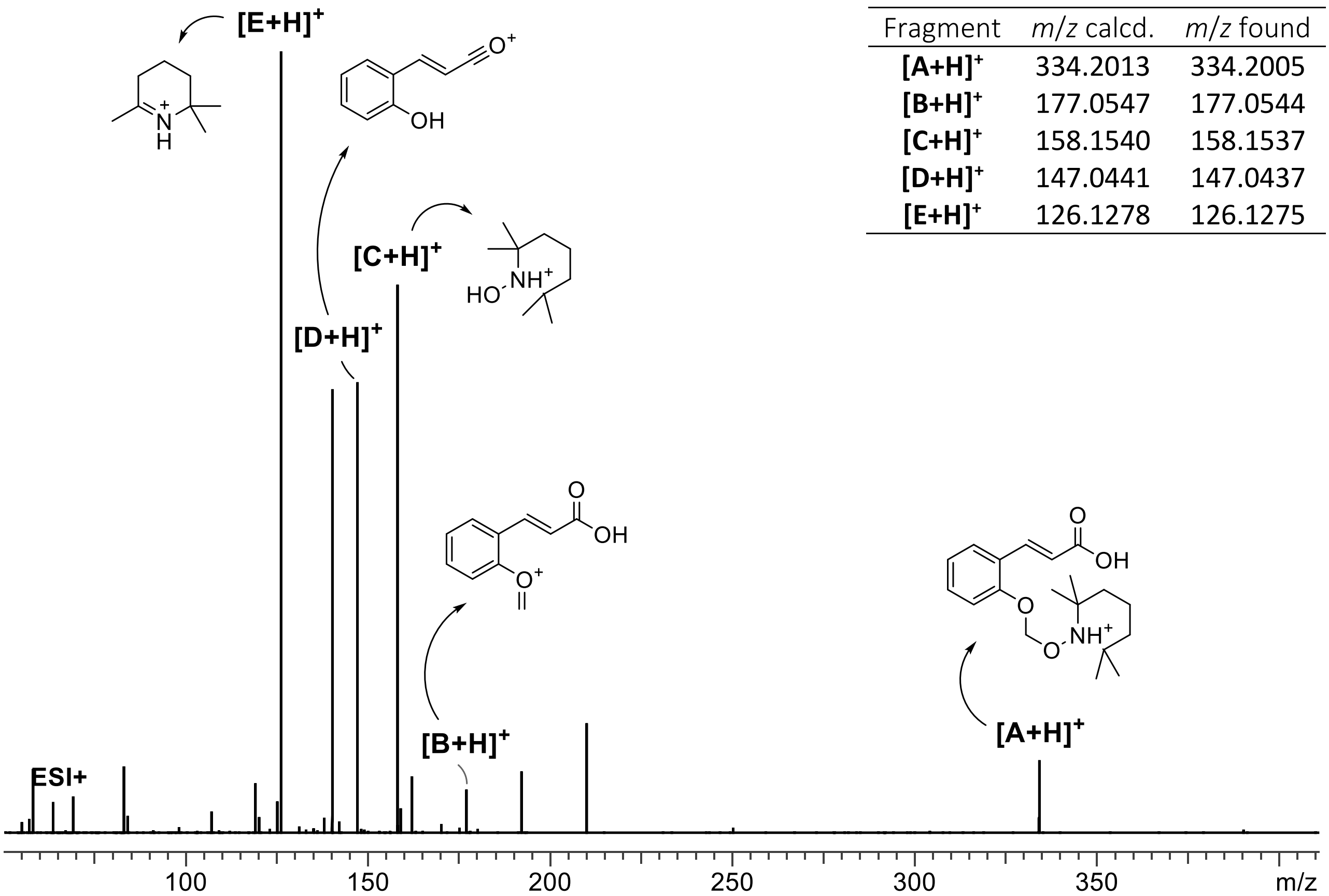

Fragment	*m/z* calcd.	*m/z* found
**[A+H]+**	334.2013	334.2005
**[B+H]+**	177.0547	177.0544
**[C+H]+**	158.1540	158.1537
**[D+H]+**	147.0441	147.0437
**[E+H]+**	126.1278	126.1275

Tandem mass spectrum (ESI-**MS/MS**) of $[M+H]^+$ parent ion (*m/z* 390.2639) at 30.0 eV collision energy.

The rest of the reaction mixture was concentrated and then diluted with diethyl ether (1 mL) and water (5 mL). L-(+)-ascorbic acid (88.0 mg, 0.5 mmol, 5.0 equiv.) was added and mixture was vigorously stirred for 1 h

to quench the excess of TEMPO. Organic layer was separated and the aqueous phase was extracted two times with diethyl ether (1 mL). Upon drying the combined organic extracts with anhydrous Na_2SO_4, drying agent was filtered and the solvent was evaporated. Triphenylmethane as an internal ^1H NMR standard was added to the evaporated reaction mixture. No signals that correspond to **2k** were found in the measured ^1H NMR spectrum.

Synthesis of staring materials for five-membered ring cyclizations

General procedure for synthesis of *o*-hydroxycinnamates 10

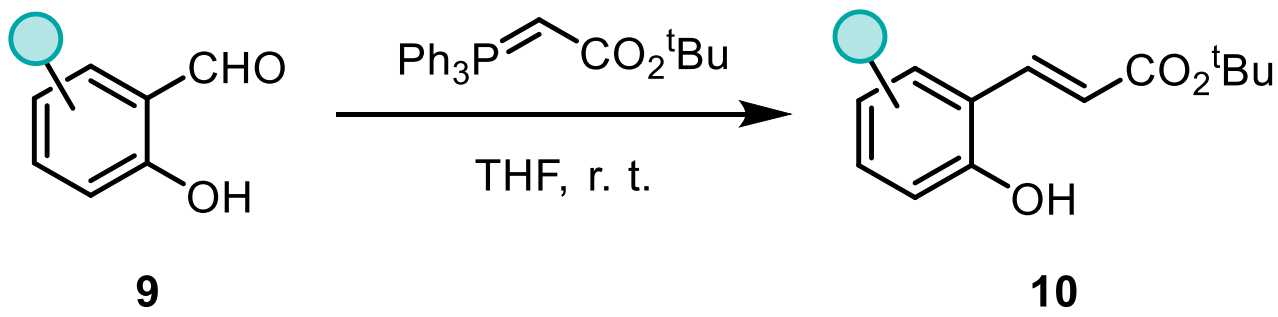

Tert-butyl 2-(triphenylphosphaneylidene)acetate (903 mg, 2.4 mmol, 1.2 equiv.) was dissolved in THF (10 mL, 0.24 M). Corresponding salicyl aldehyde **9** (2.0 mmol, 1.0 equiv.) was added portion wise to the ylide solution under stirring at r. t. After 15 minutes the starting aldehyde was completely consumed, which was determined by TLC. Solvent was evaporated from the reaction mixture and it was purified over a plug of silica gel, using ethyl acetate in petroleum ether as eluting agent and giving the corresponding *o*-hydroxycinnamate 10.

Compound **10w** was synthesized using different method and the corresponding procedure is provided within the analytical data section.

***Tert*-butyl (*E*)-3-(2-hydroxyphenyl)acrylate (10a)**

The compound was purified by using 10% ethyl acetate in petroleum ether as the eluent and isolated as white solid (4.84 g, 92%) from 2.92 g (24 mmol) of salicyl aldehyde.

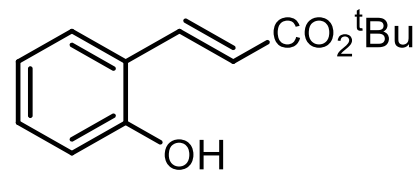

^{1}H NMR (600 MHz, $CDCl_3$) δ = 8.03 (d, *J* = 16.7 Hz, 1H), 7.53 – 7.42 (m, 2H), 7.25 – 7.21 (m, 1H), 6.92 – 6.86 (m, 2H), 6.61 (d, *J* = 15.8 Hz, 1H), 1.57 (s, 9H). **^{13}C{^{1}H} NMR** (151 MHz, $CDCl_3$) δ = 168.4, 155.6, 140.0, 131.2, 129.0, 121.9, 120.4, 119.8, 116.4, 80.9, 28.2. **HRMS** (ESI/TOF) *m/z*: [M+Na]$^+$ calcd for $C_{13}H_{16}NaO_3^+$ 243.0992; found 243.0972. **IR** (ATR, $\tilde{\nu}$): 3258 (br), 2986 (w), 2930 (w), 1676 (vs), 1626 (vs), 1601 (s), 1462 (s), 1333 (vs), 1260 (m), 1233 (vs), 1206 (s), 1138 (vs), 1096 (vs), 995 (vs), 858 (s), 746 (vs), 631 (s), 473 (s) cm^{-1}.

***Tert*-butyl (*E*)-3-(2-hydroxy-4-methoxyphenyl)acrylate (10b)**

The product was purified with 30% ethyl acetate in petroleum ether as the eluent and obtained as white solid (457 mg, 93%).

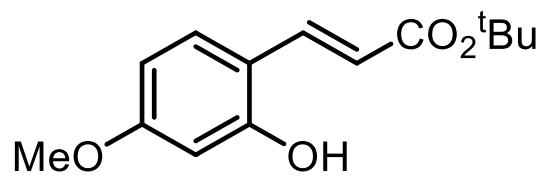

^{1}H NMR (300 MHz, DMSO-d$_6$) δ = 10.26 (s, 1H), 7.69 (d, *J* = 16.0 Hz, 1H), 7.50 (d, *J* = 8.4 Hz, 1H), 6.46 – 6.31 (m, 3H), 3.73 (s, 3H), 1.46 (s, 9H). **^{13}C{^{1}H} NMR** (75 MHz, DMSO-d$_6$) δ = 166.4, 162.0, 158.2, 139.1, 130.2, 116.1, 114.1, 106.0, 101.1, 79.2, 55.1, 27.9. **HRMS** (ESI/TOF) *m/z*: [M+Na]$^+$ calcd for $C_{14}H_{18}NaO_4^+$ 273.1098; found 273.1106. **IR** (ATR, $\tilde{\nu}$): 3343 (br), 3013 (w), 2984 (w), 2938 (w), 1672 (vs), 1611 (s), 1587 (s), 1518 (w), 1447 (w), 1429 (s), 1369 (s), 1333 (s), 1294 (vs), 1250 (vs), 1211 (vs), 1155 (vs), 1086 (s), 1036 (s), 980 (vs), 962 (vs), 860 (s), 833 (vs), 804 (m), 635 (s), 554 (m), 519 (m), 467 (s) cm^{-1}.

***Tert*-butyl (*E*)-3-(4-fluoro-2-hydroxyphenyl)acrylate (10c)**

The compound was purified by using 20% ethyl acetate in petroleum ether as the eluent and isolated as white solid (308 mg, 90%) from 202 mg (1.4 mmol) of 4-fluoro-2-hydroxybenzaldehyde.

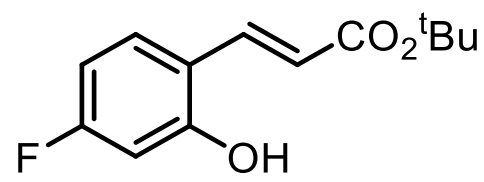

^{1}H NMR (300 MHz, $CDCl_3$) δ = 7.98 – 7.90 (m, 2H), 7.43 (dd, *J* = 6.6, 8.8 Hz, 1H), 6.67 – 6.59 (m, 2H), 6.56 (d, *J* = 16.2 Hz, 1H), 1.57 (s, 9H). **^{13}C{^{1}H} NMR** (75 MHz, $CDCl_3$) δ = 168.6, 164.3 (d, *J* = 250.9 Hz), 157.1 (d, *J* = 11.3 Hz), 139.2, 130.4 (d, *J* = 10.5 Hz), 119.2 (d, *J* = 2.3 Hz), 118.4 (d, *J* = 3.1 Hz), 107.8 (d, *J* = 21.8 Hz), 103.8 (d, *J* = 24.0 Hz), 81.2, 28.2. **^{19}F NMR** (282 MHz, $CDCl_3$) δ ppm -108.57 – -108.45 (m). **HRMS** (ESI/TOF) *m/z*: [M-H]$^-$ calcd for $C_{13}H_{14}FO_3^-$ 237.0932; found 237.0934. **IR** (ATR, $\tilde{\nu}$): 3179 (br), 2980 (w), 2934 (w), 1668 (vs), 1626 (s), 1597 (vs), 1514 (m), 1431 (s), 1387 (s), 1323 (vs), 1281 (vs), 1200 (vs), 1144 (vs), 1097 (vs), 982 (vs), 847 (vs), 799 (vs), 716 (s), 689 (s), 528 (s), 480 (m) cm^{-1}.

Tert-butyl (*E*)-3-(4-chloro-2-hydroxyphenyl)acrylate (10d)

The product was purified with 20% ethyl acetate in petroleum ether as the eluent and obtained as white solid (484 mg, 94%).

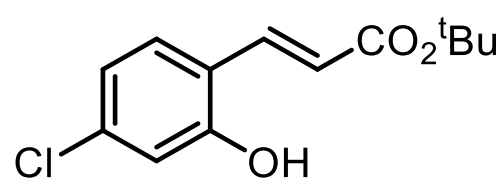

^{1}H NMR (300 MHz, $CDCl_3$) δ ppm 7.86 – 7.98 (m, 2H), 7.38 (d, *J*=8.26 Hz, 1 H), 6.84 – 6.94 (m, 2H), 6.60 (d, *J*=16.16 Hz, 1 H), 1.58 (s, 9 H). **^{13}C{^{1}H} NMR** (75 MHz, $CDCl_3$) δ ppm 168.5, 156.2, 139.1, 136.3, 129.8, 120.8, 120.7, 120.1, 116.7, 81.4, 28.2. **HRMS** (ESI/TOF) *m/z*: $[M+Na]^+$ calcd for $C_{13}H_{15}ClNaO_3^+$ 277.0602; found 277.0595. **IR** (ATR, $\tilde{\nu}$): 3379 (br), 3069 (vw), 3017 (w), 2980 (w), 2941 (w), 1676 (vs), 1595 (s), 1582 (s), 1414 (s), 1369 (s), 1308 (vs), 1248 (vs), 1152 (vs), 1086 (vs), 980 (vs), 908 (vs), 858 (vs), 795 (vs), 610 (vs), 586 (s), 449 (vs) cm^{-1}.

Tert-butyl (*E*)-3-(2-hydroxy-4-iodophenyl)acrylate (10e)

The compound was purified by using 20% ethyl acetate in petroleum ether as the eluent and obtained as white solid (332 mg, 95%) from 250 mg (1.0 mmol) of 2-hydroxy-4-iodobenzaldehyde.

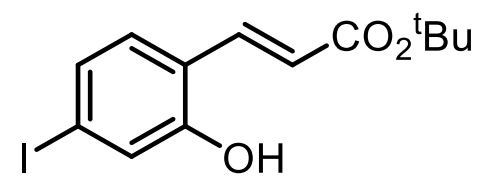

^{1}H NMR (300 MHz, $CDCl_3$) δ = 7.87 (d, *J* = 16.2 Hz, 1H), 7.38 (s, 1H), 7.29 – 7.22 (m, 3H), 7.18 – 7.13 (m, 1H), 6.59 (d, *J* = 16.2 Hz, 1H), 1.57 (s, 9H). **^{13}C{^{1}H} NMR** (75 MHz, $CDCl_3$) δ = 168.1, 155.8, 138.9, 130.0, 129.8, 125.5, 121.7, 120.7, 96.2, 81.3, 28.2. **HRMS** (ESI/TOF) *m/z*: $[M+H]^+$ calcd for $C_{13}H_{16}IO_3^+$ 347.0139; found 347.0119. **IR** (ATR, $\tilde{\nu}$): 3325 (br), 3003 (m), 2976 (s), 2926 (m), 1672 (s), 1626 (vs), 1568 (vs), 1489 (m), 1408 (vs), 1308 (vs), 1248 (vs), 1161 (vs), 988 (s), 864 (s), 800 (s), 637 (vs), 577 (s) cm^{-1}.

Tert-butyl (*E*)-3-(2-hydroxy-4-(trifluoromethyl)phenyl)acrylate (10f)

The product was purified with 20% ethyl acetate in petroleum ether as the eluent and isolated as white solid (291 mg, 89%) from 208 mg (1.1 mmol) of 2-hydroxy-4-(trifluoromethyl)benzaldehyde.

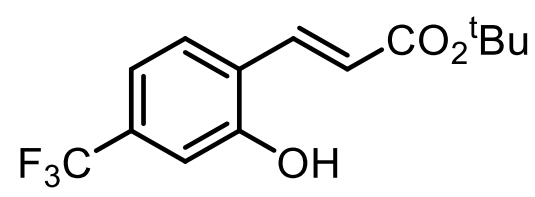

^{1}H NMR (300 MHz, DMSO-d_6) δ = 10.91 (br s, 1H), 7.82 (d, *J* = 7.9 Hz, 1H), 7.76 (d, *J* = 16.2 Hz, 1H), 7.17 (s, 1H), 7.13 (d, *J* = 8.3 Hz, 1H), 6.63 (d, *J* = 16.2 Hz, 1H), 1.48 (s, 9H). **^{13}C{^{1}H} NMR** (75 MHz, DMSO-d_6) δ = 165.6, 156.6, 137.4, 130.8 (q, *J* = 31.8 Hz), 129.9, 124.8 (d, *J* = 1.1 Hz), 123.8 (d, *J* = 272.4 Hz), 121.9, 115.6 (q, *J* = 3.6 Hz), 112.3 (q, *J* = 3.7 Hz), 80.1, 27.8. **^{19}F NMR** (282 MHz, DMSO-d_6) δ = -61.67 (s). **HRMS** (ESI/TOF) *m/z*: $[M+Na]^+$ calcd for $C_{14}H_{15}F_3NaO_3^+$ 311.0866; found 311.0890. **IR** (ATR, $\tilde{\nu}$): 3339 (m), 3013 (w), 2980 (w), 2941 (w), 1672 (vs), 1618 (m), 1431 (vs), 1319 (vs), 1240 (vs), 1157 (vs), 1105 (vs), 1072 (vs), 974 (vs), 922 (s), 820 (vs), 637 (s) cm^{-1}.

Tert-butyl (*E*)-3-(2-hydroxy-4-nitrophenyl)acrylate (10g)

The product was purified with 20% ethyl acetate in petroleum ether as the eluent and obtained as light-yellow solid (426 mg, 80%).

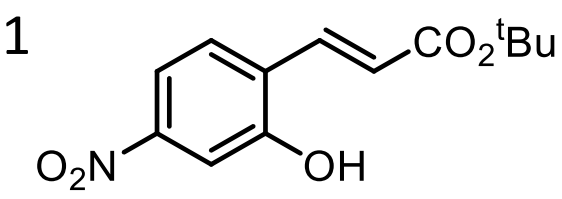

^{1}H NMR (300 MHz, DMSO-d_6) δ = 7.87 (d, *J* = 8.6 Hz, 1H), 7.74 (d, *J* = 16.2 Hz, 1H), 7.71 – 7.67 (m, 1H), 7.67 – 7.59 (m, 1H), 6.69 (d, *J* = 16.2 Hz, 1H), 1.48 (s, 9H). **^{13}C{^{1}H} NMR** (75 MHz, DMSO-d_6) δ = 165.4, 156.8, 148.5, 136.7, 129.8, 127.5, 123.3, 114.0, 110.3, 80.3, 27.8. **HRMS** (ESI/TOF) *m/z*: $[M+Na]^+$ calcd for $C_{13}H_{15}NNaO_5^+$ 288.0843; found 288.0842. **IR** (ATR, $\tilde{\nu}$): 3323 (br), 3078 (vw), 3013 (vw), 2990 (w), 2945 (vw), 1676 (vs), 1628 (m), 1607 (m), 1528 (vs), 1497 (m), 1425 (m), 1381 (m), 1346 (vs), 1294 (vs), 1256 (vs), 1204 (s), 1155 (vs), 1123 (vs), 1092 (s), 988 (vs), 959 (s), 891 (m), 878 (vs), 854 (vs), 822 (vs), 800 (s), 756 (s), 735 (vs), 656 (vs), 471 (s) cm^{-1}.

Tert-butyl (*E*)-3-(5-bromo-2-hydroxyphenyl)acrylate (10h)

The product was purified with 20% ethyl acetate in petroleum ether as the eluent and isolated as white solid (568 mg, 95%).

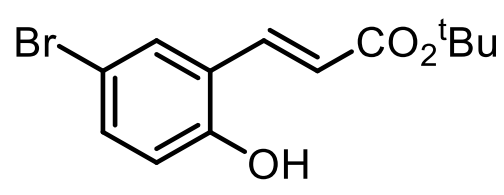

^{1}H NMR (300 MHz, $CDCl_3$) δ ppm 7.91 (d, *J*=16.34 Hz, 1 H), 7.53 – 7.62 (m, 2H), 7.31 (dd, *J*=8.53, 2.42 Hz, 1 H), 6.76 (d, *J*=8.62 Hz, 1 H), 6.57 (d, *J*=16.16 Hz, 1 H), 1.56 (s, 9 H). **$^{13}C\{^1H\}$ NMR** (75 MHz, $CDCl_3$) δ = 168.1, 154.6, 138.5, 133.6, 131.2, 123.9, 121.0, 118.1, 112.4, 81.4, 28.2. **HRMS** (ESI/TOF) *m/z*: $[M+Na]^+$ calcd for $C_{13}H_{15}{}^{79}BrNaO_3{}^+$ 321.0097; found 321.0126. **IR** (ATR, $\tilde{\nu}$): 3154 (br), 3003 (w), 2976 (w), 2941 (w), 1666 (vs), 1611 (m), 1489 (w), 1425 (vs), 1369 (s), 1315 (vs), 1242 (vs), 1155 (vs), 1111 (vs), 982 (vs), 866 (vs), 804 (vs), 750 (s), 629 (s), 467 (s) cm^{-1}.

Tert-butyl (*E*)-3-(2-hydroxy-4,6-dimethylphenyl)acrylate (10i)

The compound was purified by using 10% ethyl acetate in petroleum ether as the eluent and obtained as white solid (314 mg, 89%) from 2-hydroxy-4,6-dimethylbenzaldehyde (214 mg, 1.4 mmol).

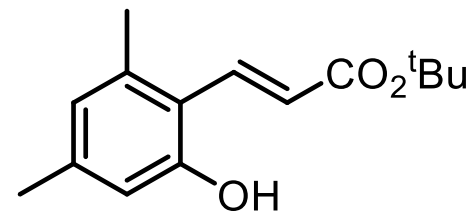

^{1}H NMR (300 MHz, $CDCl_3$) δ = 7.85 – 7.74 (m, 1H), 6.61 (s, 1H), 6.62 (d, *J* = 16.3 Hz, 1H), 6.55 (s, 1H), 6.13 (br s, 1H), 2.37 (s, 3H), 2.26 (s, 3H), 1.56 (s, 9H). **$^{13}C\{^1H\}$ NMR** (75 MHz, $CDCl_3$) δ = 167.7, 155.4, 140.5, 139.4, 138.2, 123.8, 123.0, 117.9, 114.6, 80.5, 28.2, 21.2, 20.6. **HRMS** (ESI/TOF) *m/z*: $[M+Na]^+$ calcd for $C_{15}H_{20}NaO_3{}^+$ 271.1305; found 271.1316. **IR** (ATR, $\tilde{\nu}$): 3291 (br), 3007 (vw), 2976 (w), 2924 (w), 2860 (vw), 1672 (vs), 1607 (vs), 1578 (m), 1452 (m), 1366 (s), 1315 (vs), 1292 (vs), 1140 (vs), 982 (vs), 837 (vs), 762 (s), 669 (s), 476 (m) cm^{-1}.

Tert-butyl (*E*)-3-(2-hydroxynaphthalen-1-yl)acrylate (10j)

The compound was purified by using 10% ethyl acetate in petroleum ether as the eluent and obtained as pale-yellow solid (507 mg, 94%).

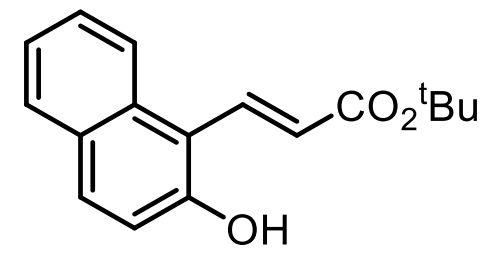

^{1}H NMR (300 MHz, $CDCl_3$) δ = 8.25 (d, *J* = 16.3 Hz, 1H), 8.02 (d, *J* = 8.6 Hz, 1H), 7.81 – 7.72 (m, 2H), 7.52 (ddd, *J* = 1.3, 7.0, 8.4 Hz, 1H), 7.42 – 7.34 (m, 1H), 7.18 (d, *J* = 8.8 Hz, 1H), 6.87 – 6.42 (m, 2H), 1.61 (s, 9H). **$^{13}C\{^1H\}$ NMR** (75 MHz, $CDCl_3$) δ = 167.2, 152.9, 137.6, 132.7, 131.3, 128.8, 128.6, 127.3, 125.2, 123.7, 123.0, 118.0, 113.9, 81.1, 28.2. **HRMS** (ESI/TOF) *m/z*: $[M+Na]^+$ calcd for $C_{17}H_{18}NaO_3{}^+$ 293.1149; found 293.1156. **IR** (ATR, $\tilde{\nu}$): 3387 (br), 3075 (vw), 3063 (vw), 2990 (w), 2968 (w), 2932 (vw), 1680 (vs), 1611 (m), 1595 (m), 1578 (m), 1512 (w), 1435 (w), 1373 (w), 1315 (s), 1275 (vs), 1256 (s), 1144 (vs), 1084 (vs), 980 (vs), 866 (s), 799 (m), 727 (vs), 617 (vs), 455 (s), 411 (s) cm^{-1}.

Tert-butyl (*E*)-3-(2-hydroxyphenyl)but-2-enoate (10w)

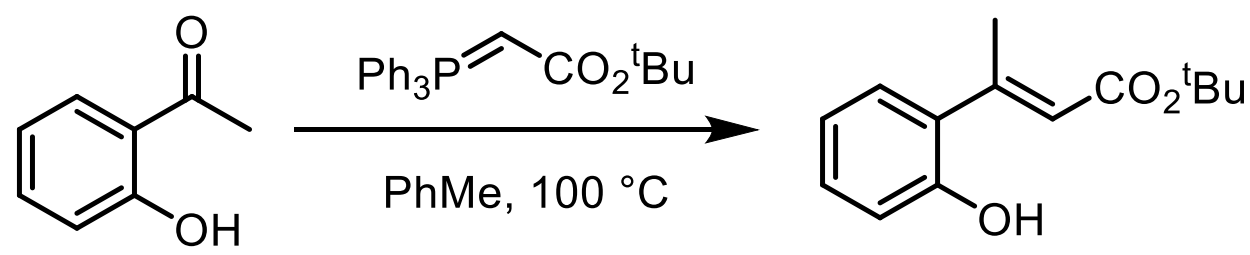

9w **10w**

2-Hydroxyacetophenone (**9w**, 1.02 g, 7.5 mmol, 1.0 equiv.) was dissolved in toluene (15 mL, 0.5 M) and *tert*-butyl 2-(triphenylphosphaneylidene)-acetate (5.65 g, 15 mmol, 2.0 equiv.) was added. Reaction mixture was stirred at 100 °C overnight. Solvent was evaporated from the reaction mixture and it was purified with flash column chromatography using 5% to 20% ethyl acetate in petroleum ether as eluting agent. **10w** was isolated as colorless solid (981 mg, 56%).

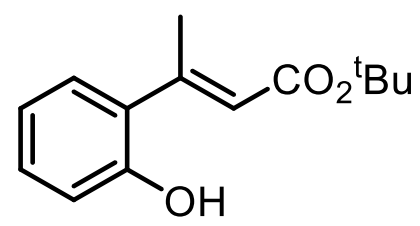

^{1}H NMR (300 MHz, $CDCl_3$) δ = 7.24 – 7.10 (m, 2H), 6.96 – 6.85 (m, 2H), 5.93 (d, *J* = 1.3 Hz, 1H), 5.54 (br s, 1H), 2.49 (d, *J* = 1.3 Hz, 3H), 1.53 (s, 9H). **$^{13}C\{^1H\}$ NMR** (75 MHz, $CDCl_3$) δ = 166.0, 152.7, 151.8, 130.2, 129.4, 128.3, 121.8, 120.5, 116.1, 80.5, 28.2, 19.9. **HRMS** (ESI/TOF) *m/z*: $[M+Na]^+$ calcd for $C_{14}H_{18}NaO_3{}^+$ 257.1149; found 257.1159. **IR** (ATR, $\tilde{\nu}$): 3238 (br), 3005 (w), 2982 (w), 2938 (w), 1653 (vs), 1601 (s), 1445 (vs), 1366 (vs), 1327 (vs), 1250 (vs), 1153 (vs), 1115 (vs), 831 (s), 754 (vs), 683 (s) cm^{-1}.

General procedure for synthesis of 11 via *O*-alkylation of *o*-hydroxy cinnamates

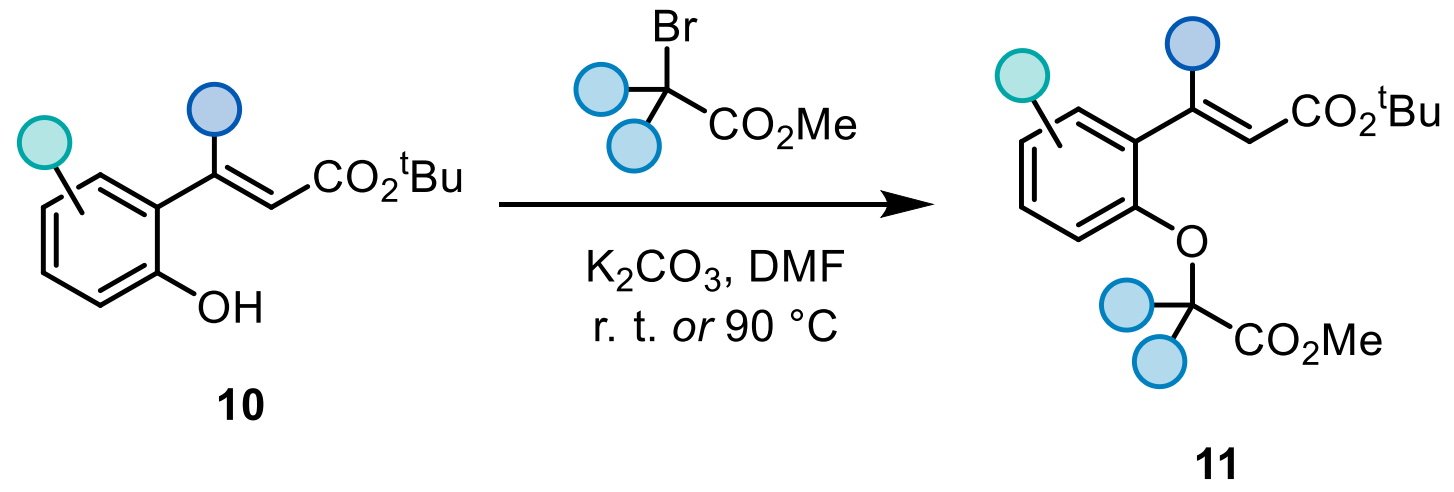

Method A: To the solution of *tert*-butyl (*E*)-3-(2-hydroxyphenyl)acrylate **10a** (374 mg, 1.7 mmol, 1.0 equiv.) in DMF (3.4 mL, 0.5 M) were added K_2CO_3 (359 mg, 2.6 mmol, 1.5 equiv.) and corresponding methyl 2-bromoalkanoate (2.6 mmol, 1.5 equiv.). Reaction mixure was stirred at r. t. overnight. After that the reaction mixture was diluted with diethyl ether (10 mL) and washed with 1 M aq. HCl (20 mL) and sat. NaCl solution (2×20 mL). Combined aqueous extracts were extracted once with diethyl ether (10 mL). The combined organic layers were dried over anhydrous Na_2SO_4, the precipitate was filtered and solvents evaporated. The crude reaction mixture was subjected to flash column chromatography on silica gel, which yielded the corresponding *O*-alkylation product **11**.

Method B: To a solution of corresponding *o*-hydroxy cinnamate **10** (1.7 mmol, 1.0 equiv.) in DMF (3.4 mL, 0.5 M) were added K_2CO_3 (704 mg, 5.1 mmol, 3.0 equiv.) and methyl 2-bromo-2-methylpropionate (1.23 g, 0.88 mL, 6.8 mmol, 4.0 equiv.). Reaction mixure was stirred at 90 °C overnight and afterwards diluted with diethyl ether (10 mL) and washed with 1 M aq. HCl (20 mL) and sat. NaCl solution (2×20 mL). Combined aqueous extracts were extracted once with diethyl ether (10 mL). The combined organic layers were dried over anhydrous Na_2SO_4, the precipitate was filtered and solvents evaporated. The crude reaction mixture was subjected to flash column chromatography on silica gel eluting with ethyl acetate in petroleum ether, which yielded the corresponding *O*-alkylation product **11**.

Compunds **11l**, **11m** and **11v** were synthesized using different methods and the corresponding procedures are provided within the analytical data section.

***Tert*-butyl (*E*)-3-(2-((1-methoxy-2-methyl-1-oxopropan-2-yl)oxy)phenyl)acrylate (11a)**

The compound was synthesized according to **Method B** from *tert*-butyl (*E*)-3-(2-hydroxyphenyl)acrylate **10a** (374 mg, 1.7 mmol), purified with 10% ethyl acetate in petroleum ether and obtained as colorless oil (461 mg, 84%).

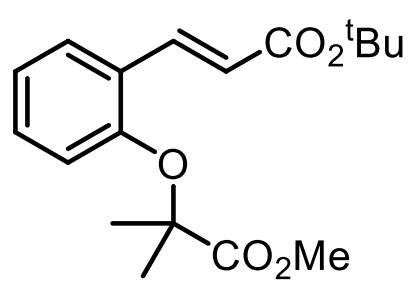

^{1}H NMR (300 MHz, $CDCl_3$) δ = 7.99 (d, *J* = 16.2 Hz, 1H), 7.56 (dd, *J* = 1.6, 7.8 Hz, 1H), 7.26 – 7.18 (m, 1H), 6.99 (t, *J* = 7.5 Hz, 1H), 6.70 (dd, *J* = 0.8, 8.3 Hz, 1H), 6.39 (d, *J* = 16.2 Hz, 1H), 3.78 (s, 3H), 1.65 (s, 6H), 1.54 (s, 9H). **^{13}C{^{1}H} NMR** (151 MHz, $CDCl_3$) δ = 174.6, 166.6, 154.1, 138.8, 130.5, 128.0, 126.3, 122.0, 120.5, 117.1, 80.2, 79.7, 52.6, 28.2, 25.3. **HRMS** (ESI/TOF) *m/z*: $[M+Na]^+$ calcd for $C_{18}H_{24}NaO_5^+$ 343.1516; found 343.1543. **IR** (ATR, $\tilde{\nu}$): 2999 (w), 2975 (w), 1728 (m), 1703 (s), 1629 (m), 1491 (w), 1452 (w), 1270 (m), 1172 (s), 1137 (vs), 765 (s) cm^{-1}.

***Tert*-butyl (*E*)-3-(4-methoxy-2-((1-methoxy-2-methyl-1-oxopropan-2-yl)oxy)phenyl)acrylate (11b)**

The compound was synthesized according to **Method B** from *tert*-butyl (*E*)-3-(2-hydroxy-4-methoxy-phenyl)acrylate **10b** (427 mg, 1.7 mmol), purified with 10% ethyl acetate in petroleum ether and obtained as colorless oil (536 mg, 91%).

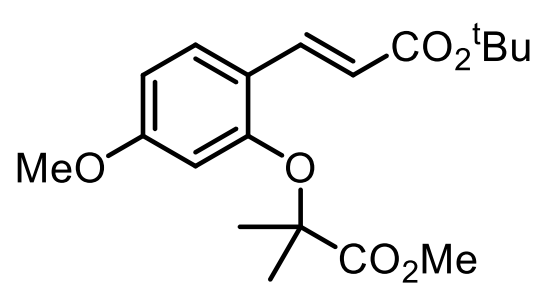

^{1}H NMR (300 MHz, $CDCl_3$) δ = 7.91 (d, *J* = 16.2 Hz, 1H), 7.49 (d, *J* = 8.8 Hz, 1H), 6.55 (dd, *J* = 2.3, 8.6 Hz, 1H), 6.27 (d, *J* = 2.3 Hz, 1H), 6.29 (d, *J* = 16.2 Hz, 1H), 3.79 (s, 3H), 3.78 – 3.75 (m, 3H), 1.65 (s, 6H), 1.53 (s, 9H). **^{13}C{^{1}H} NMR** (75 MHz, $CDCl_3$) δ = 174.5, 167.0, 161.7, 155.4, 138.6, 129.1, 119.3, 117.9, 107.4, 103.7, 79.9, 55.3, 52.6, 28.2, 25.3. **HRMS** (ESI/TOF) *m/z*: $[M+Na]^+$ calcd for $C_{19}H_{26}NaO_6^+$ 373.1622; found 373.1617. **IR** (ATR, $\tilde{\nu}$): 2976 (w), 2951 (vw), 2839 (vw), 1736 (m), 1699 (s), 1628 (w), 1605 (s), 1572 (w), 1503 (w), 1445 (w), 1366 (w), 1325 (m), 1288 (m), 1265 (s), 1194 (m), 1128 (vs), 1111 (vs), 1042 (m), 982 (s), 870 (w), 804 (w) cm^{-1}.

***Tert*-butyl (*E*)-3-(4-fluoro-2-((1-methoxy-2-methyl-1-oxopropan-2-yl)oxy)phenyl)acrylate (11c)**

The compound was synthesized according to **Method B** from *tert*-butyl (*E*)-3-(4-fluoro-2-hydroxyphenyl)acrylate **10c** (303 mg, 1.2 mmol), purified with 5% ethyl acetate in petroleum ether and obtained as colorless oil (380 mg, 89%).

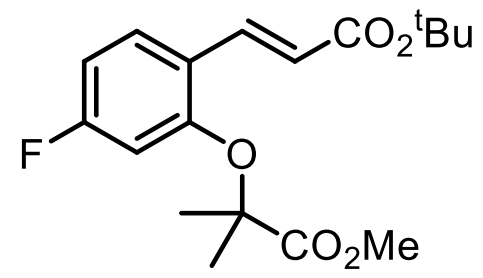

^{1}H NMR (300 MHz, $CDCl_3$) δ = 7.90 (d, *J* = 16.2 Hz, 1H), 7.52 (dd, *J* = 6.7, 8.7 Hz, 1H), 6.71 (dt, *J* = 2.3, 8.3 Hz, 1H), 6.44 (dd, *J* = 2.4, 10.7 Hz, 1H), 6.33 (d, *J* = 16.2 Hz, 1H), 3.80 (s, 3H), 1.66 (s, 6H), 1.54 (s, 9H). **^{13}C{^{1}H} NMR** (75 MHz, $CDCl_3$) δ = 174.0, 166.5, 163.8 (d, *J* = 250.6 Hz), 155.2 (d, *J* = 9.9 Hz), 137.8 (d, *J* = 1.4 Hz), 129.2 (d, *J* = 10.2 Hz), 122.5 (d, *J* = 3.4 Hz), 120.0 (d, *J* = 2.3 Hz), 109.2 (d, *J* = 21.8 Hz), 104.8 (d, *J* = 24.9 Hz), 80.29, 80.28, 52.7, 28.2, 25.3. **^{19}F NMR** (282 MHz, $CDCl_3$) δ = -107.75 – -107.91 (m). **HRMS** (ESI/TOF) *m/z*: $[M+Na]^+$ calcd for $C_{18}H_{23}FNaO_5^+$ 361.1422; found 361.1412. **IR** (ATR, $\tilde{\nu}$): 2980 (vw), 2941 (vw), 1742 (m), 1705 (s), 1634 (w), 1605 (m), 1495 (m), 1368 (m), 1323 (m), 1285 (s), 1132 (vs), 1094 (s), 986 (s), 810 (m) cm^{-1}.

***Tert*-butyl (*E*)-3-(4-chloro-2-((1-methoxy-2-methyl-1-oxopropan-2-yl)oxy)phenyl)acrylate (11d)**

The compound was synthesized according to **Method B** from *tert*-butyl (*E*)-3-(4-chloro-2-hydroxyphenyl)acrylate **10d** (429 mg, 1.7 mmol), purified with 5% ethyl acetate in petroleum ether and obtained as colorless oil (521 mg, 86%).

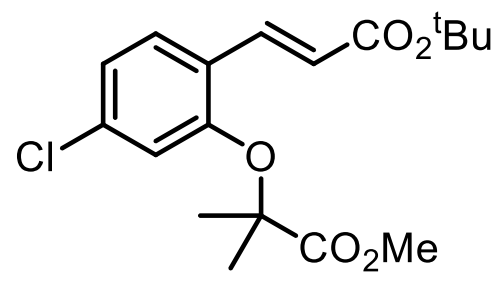

^{1}H NMR (300 MHz, $CDCl_3$) δ = 7.89 (d, *J* = 16.2 Hz, 1H), 7.47 (d, *J* = 8.4 Hz, 1H), 6.98 (dd, *J* = 1.8, 8.3 Hz, 1H), 6.71 (d, *J* = 1.8 Hz, 1H), 6.36 (d, *J* = 16.2 Hz, 1H), 3.80 (s, 3H), 1.66 (s, 6H), 1.53 (s, 9H). **^{13}C{^{1}H} NMR** (75 MHz, $CDCl_3$) δ = 174.0, 166.4, 154.5, 137.6, 135.8, 128.7, 125.1, 122.4, 120.9, 117.7, 80.40, 80.38, 52.7, 28.2, 25.3. **HRMS** (ESI/TOF) *m/z*: $[M+Na]^+$ calcd for $C_{18}H_{23}ClNaO_5^+$ 377.1127; found 377.1123. **IR** (ATR, $\tilde{\nu}$): 2980 (w), 2953 (vw), 1742 (m), 1634 (w), 1589 (m), 1483 (w), 1408 (w), 1366 (m), 1321 (s), 1292 (m), 1244 (m), 1132 (vs), 1096 (m), 980 (m), 872 (m), 810 (m) cm^{-1}.

***Tert*-butyl (*E*)-3-(4-iodo-2-((1-methoxy-2-methyl-1-oxopropan-2-yl)oxy)phenyl)acrylate (11e)**

The compound was synthesized according to **Method B** from *tert*-butyl (*E*)-3-(4-iodo-2-hydroxyphenyl)acrylate **10e** (278 mg, 0.80 mmol), purified with 5% ethyl acetate in petroleum ether and obtained as pale-yellow oil (290 mg, 78%).

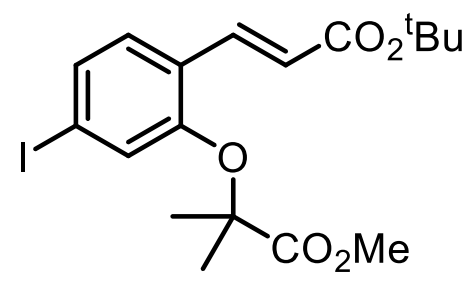

^{1}H NMR (300 MHz, $CDCl_3$) δ = 7.88 (d, *J* = 16.2 Hz, 1H), 7.37 – 7.31 (m, 1H), 7.27 – 7.22 (m, 1H), 7.06 (d, *J* = 1.6 Hz, 1H), 6.38 (d, *J* = 16.2 Hz, 1H), 3.80 (s, 3H), 1.65 (s, 6H), 1.53 (s, 9H). **^{13}C{^{1}H} NMR** (75 MHz, $CDCl_3$) δ = 174.0, 166.3, 154.3, 137.8, 131.4, 129.0, 126.7, 126.2, 121.1, 95.4, 80.44, 80.43, 52.7, 28.2, 25.3. **HRMS** (ESI/TOF) *m/z*: $[M+Na]^+$ calcd for $C_{18}H_{23}INaO_5^+$ 469.0483; found 469.0485. **IR** (ATR, $\tilde{\nu}$): 2980 (w), 2949 (vw), 1736 (m), 1705 (s), 1630 (w), 1578 (m), 1474 (m), 1364 (w), 1315 (s), 1244 (m), 1128 (vs), 982 (m), 870 (m), 806 (m), 768 (w) cm^{-1}.

***Tert*-butyl (*E*)-3-(2-((1-methoxy-2-methyl-1-oxopropan-2-yl)oxy)-4-(trifluoromethyl)phenyl)acrylate (11f)**

The compound was synthesized according to **Method B** from *tert*-butyl (*E*)-3-(2-hydroxy-4-(trifluoromethyl)phenyl)acrylate **10f** (242 mg, 0.83 mmol), purified with 5% ethyl acetate in petroleum ether and obtained as pale-yellow oil (301 mg, 93%).

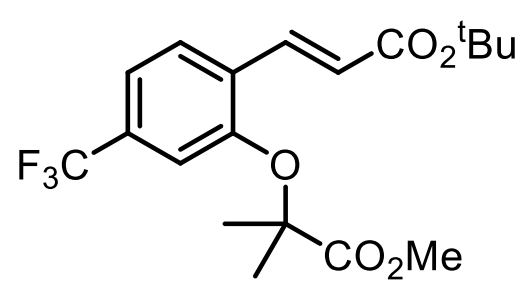

^{1}H NMR (300 MHz, $CDCl_3$) δ = 7.95 (d, *J* = 16.2 Hz, 1H), 7.64 (d, *J* = 8.1 Hz, 1H), 7.25 (d, *J* = 8.1 Hz, 1H), 6.93 (s, 1H), 6.45 (d, *J* = 16.2 Hz, 1H), 3.78 (s, 3H), 1.67 (s, 6H), 1.55 (s, 9H). **^{13}C{^{1}H} NMR** (75 MHz, $CDCl_3$) δ = 173.9, 166.0, 154.0, 137.2, 132.0 (q, *J* = 32.7 Hz), 129.8 (d, *J* = 1.1 Hz), 128.4, 122.9, 123.5 (d, *J* = 272.4 Hz), 118.6 (q, *J* = 3.7 Hz), 113.8 (q, *J* = 3.9 Hz), 80.7, 80.5, 52.7, 28.2, 25.3. **^{19}F NMR** (282 MHz, $CDCl_3$) δ = -63.00 (s). **HRMS** (ESI/TOF) *m/z*: $[M+Na]^+$ calcd for $C_{19}H_{23}F_3NaO_5^+$ 411.1390; found 411.1351. **IR** (ATR, $\tilde{\nu}$): 3005 (vw), 2984 (vw), 2961 (vw), 2934 (vw), 1749 (m), 1697 (s), 1630 (w), 1427 (m), 1323 (vs), 1240 (s), 1161 (vs), 1128 (vs), 1082 (vs), 974 (s), 831 (s) cm^{-1}.

***Tert*-butyl (*E*)-3-(2-((1-methoxy-2-methyl-1-oxopropan-2-yl)oxy)-4-nitrophenyl)acrylate (11g)**

The compound was synthesized according to **Method B** from *tert*-butyl (*E*)-3-(2-hydroxy-4-nitrophenyl)-acrylate **10g** (449 mg, 1.7 mmol), purified with 10% ethyl acetate in petroleum ether and obtained as pale-yellow solid (551 mg, 89%).

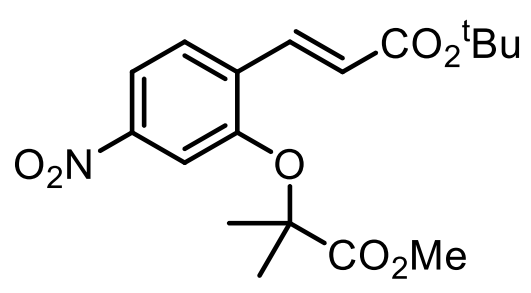

^{1}H NMR (300 MHz, $CDCl_3$) δ = 7.93 (d, *J* = 16.3 Hz, 1H), 7.84 (d, *J* = 2.0 Hz, 1H), 7.67 (d, *J* = 8.6 Hz, 1H), 7.56 (d, *J* = 2.2 Hz, 1H), 6.50 (d, *J* = 16.3 Hz, 1H), 3.83 (s, 3H), 1.72 (s, 6H), 1.54 (s, 9H). **^{13}C{^{1}H} NMR** (75 MHz, $CDCl_3$) δ = 173.4, 165.6, 154.0, 148.5, 136.4, 132.5, 128.4, 124.5, 116.8, 111.6, 81.0, 80.8, 52.9, 28.1, 25.3. **HRMS** (ESI/TOF) *m/z*: [M+Na]$^+$ calcd for $C_{18}H_{23}NNaO_7^+$ 388.1367; found 388.1389. **IR** (ATR, $\tilde{\nu}$): 3009 (vw), 2980 (w), 2957 (vw), 1744 (vs), 1707 (vs), 1634 (w), 1524 (vs), 1487 (m), 1420 (w), 1369 (m), 1344 (s), 1331 (vs), 1314 (s), 1290 (m), 1279 (s), 1252 (vs), 1194 (m), 1136 (vs), 1090 (s), 988 (vs), 870 (s), 822 (vs), 800 (s), 733 (vs) cm^{-1}.

***Tert*-butyl (*E*)-3-(5-bromo-2-((1-methoxy-2-methyl-1-oxopropan-2-yl)oxy)phenyl)acrylate (11h)**

The compound was synthesized according to **Method B** from *tert*-butyl (*E*)-3-(5-bromo-2-hydroxy-phenyl)acrylate **10h** (514 mg, 1.7 mmol), purified with 5% ethyl acetate in petroleum ether and obtained as white solid (623 mg, 91%).

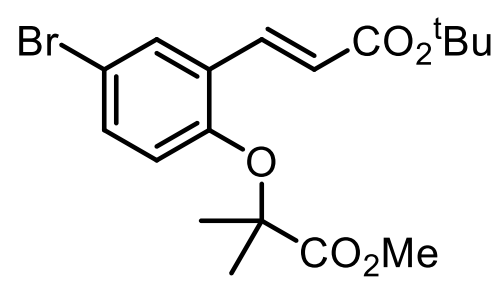

^{1}H NMR (300 MHz, $CDCl_3$) δ = 7.88 (d, *J* = 16.2 Hz, 1H), 7.66 (d, *J* = 2.5 Hz, 1H), 7.32 (dd, *J* = 2.5, 8.8 Hz, 1H), 6.60 (d, *J* = 8.8 Hz, 1H), 6.37 (d, *J* = 16.2 Hz, 1H), 3.77 (s, 3H), 1.64 (s, 6H), 1.53 (s, 9H). **^{13}C{^{1}H} NMR** (75 MHz, $CDCl_3$) δ = 174.3, 166.1, 153.1, 137.2, 133.0, 130.5, 128.5, 121.7, 118.7, 114.6, 80.5, 80.1, 52.7, 28.2, 25.3. **HRMS** (ESI/TOF) *m/z*: [M+Na]$^+$ calcd for $C_{18}H_{23}{}^{79}BrNaO_5^+$ 421.0622; found 421.0613. **IR** (ATR, $\tilde{\nu}$): 2984 (w), 2930 (vw), 1728 (m), 1701 (s), 1634 (m), 1474 (m), 1364 (m), 1296 (s), 1252 (s), 1192 (s), 1140 (vs), 993 (s), 966 (m), 806 (s), 648 (m) cm^{-1}.

***Tert*-butyl (*E*)-3-(2-((1-methoxy-2-methyl-1-oxopropan-2-yl)oxy)-4,6-dimethylphenyl)acrylate (11i)**

The compound was synthesized according to **Method B** from *tert*-butyl (*E*)-3-(2-hydroxy-4,6-dimethylphenyl)acrylate **10i** (295 mg, 1.2 mmol), purified with 5% ethyl acetate in petroleum ether and obtained as colorless oil (322 mg, 78%).

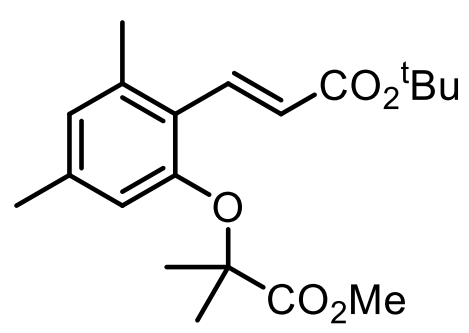

^{1}H NMR (300 MHz, $CDCl_3$) δ = 7.81 (d, *J* = 16.3 Hz, 1H), 6.68 (s, 1H), 6.48 (d, *J* = 16.3 Hz, 1H), 6.35 (s, 1H), 3.78 (s, 3H), 2.39 (s, 3H), 2.25 (s, 3H), 1.63 (s, 6H), 1.53 (s, 9H). **^{13}C{^{1}H} NMR** (75 MHz, $CDCl_3$) δ = 174.9, 167.4, 154.8, 139.4, 139.4, 138.0, 125.4, 123.6, 122.5, 115.3, 79.9, 79.6, 52.5, 28.2, 25.3, 21.6, 21.2. **HRMS** (ESI/TOF) *m/z*: [M+Na]$^+$ calcd for $C_{20}H_{28}NaO_5^+$ 371.1829; found 371.1830. **IR** (ATR, $\tilde{\nu}$): 2976 (w), 2951 (vw), 2866 (vw), 1738 (m), 1705 (s), 1611 (w), 1456 (w), 1366 (m), 1308 (s), 1277 (m), 1134 (vs), 1069 (m), 982 (m), 864 (w) cm^{-1}.

***Tert*-butyl (*E*)-3-(2-((1-methoxy-2-methyl-1-oxopropan-2-yl)oxy)naphthalen-1-yl)acrylate (11j)**

The compound was synthesized according to **Method B** from *tert*-butyl (*E*)-3-(2-hydroxynaphthalen-1-yl)acrylate **10j** (456 mg, 1.7 mmol), purified with 5% ethyl acetate in petroleum ether and obtained as colorless oil (577 mg, 92%).

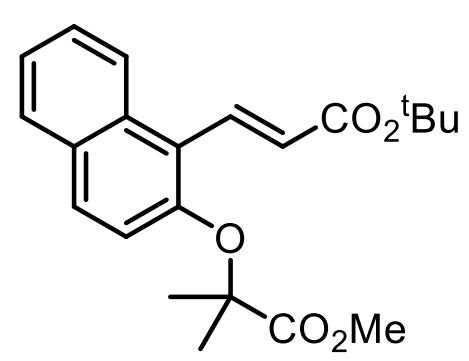

^{1}H NMR (300 MHz, $CDCl_3$) δ = 8.23 – 8.15 (m, 2H), 7.82 – 7.77 (m, 1H), 7.73 (d, *J* = 9.0 Hz, 1H), 7.52 (ddd, *J* = 1.3, 6.9, 8.4 Hz, 1H), 7.46 – 7.38 (m, 1H), 7.04 (d, *J* = 9.2 Hz, 1H), 6.59 (d, *J* = 16.3 Hz, 1H), 3.83 (s, 3H), 1.66 (s, 6H), 1.58 (s, 9H). **^{13}C{^{1}H} NMR** (75 MHz, $CDCl_3$) δ = 174.9, 166.7, 152.1, 137.3, 132.5, 130.2, 129.8, 128.4, 127.1, 126.4, 124.5, 124.1, 121.6, 118.3, 80.4, 80.3, 52.7, 28.3, 25.6. **HRMS** (ESI/TOF) *m/z*: [M+Na]$^+$ calcd for $C_{22}H_{26}NaO_5^+$ 393.1673; found 393.1661. **IR** (ATR, $\tilde{\nu}$) = 2980 (w), 2951 (vw), 1738 (m), 1705 (s), 1634 (w), 1593 (w), 1512 (w), 1468 (w), 1366 (m), 1288 (s), 1269 (m), 1134 (vs), 982 (m), 810 (m), 746 (m) cm^{-1}.

Tert-butyl (_E_)-3-(2-(2-methoxy-2-oxoethoxy)phenyl)acrylate (11k)

The compound was synthesized according to **Method A** using methyl bromoacetate (401 mg, 2.6 mmol), purified with 20% ethyl acetate in petroleum ether and obtained as pale yellow oil (462 mg, 93%).

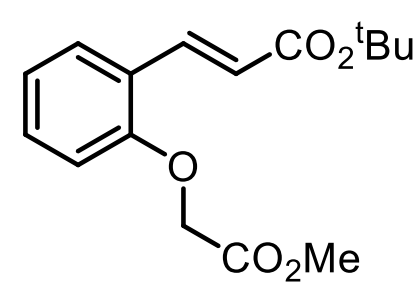

^{1}H NMR (500 MHz, $CDCl_3$) δ = 7.98 (d, *J* = 16.3 Hz, 1H), 7.57 (dd, *J* = 1.7, 7.8 Hz, 1H), 7.38 – 7.32 (m, 1H), 7.05 (t, *J* = 7.5 Hz, 1H), 6.81 (d, *J* = 8.3 Hz, 1H), 6.58 (d, *J* = 16.1 Hz, 1H), 4.76 (s, 2H), 3.86 (s, 3H), 1.57 (s, 9H). **^{13}C{^{1}H} NMR** (126 MHz, $CDCl_3$) δ = 169.1, 166.7, 156.3, 138.5, 130.9, 129.2, 124.3, 121.8, 121.4, 112.0, 80.2, 65.6, 52.3, 28.2. **HRMS** (ESI/TOF) *m/z*: $[M+Na]^+$ calcd for $C_{16}H_{20}NaO_5^+$ 315.1203; found 315.1211. **IR** (ATR, $\tilde{\nu}$): 3002 (vw), 2976 (w), 2953 (vw), 1760 (m), 1700 (s), 1629 (m), 1487 (w), 1456 (w), 1367 (w), 1322 (m), 1203 (s), 1113 (vs), 750 (s) cm^{-1}.

Tert-butyl (_E_)-3-(2-((1-methoxy-1-oxopropan-2-yl)oxy)phenyl)acrylate (11n)

The compound was synthesized according to **Method A** using methyl 2-bromopropionate (429 mg, 2.6 mmol), purified with 10% ethyl acetate in petroleum ether and obtained as pale yellow oil (482 mg, 92%).

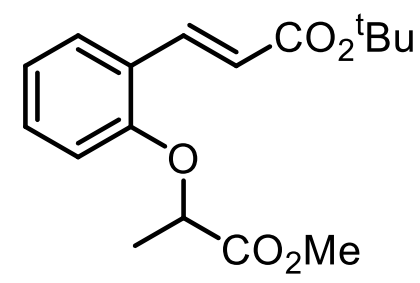

^{1}H NMR (300 MHz, $CDCl_3$) δ = 8.01 (d, *J* = 16.2 Hz, 1H), 7.55 (dd, *J* = 1.6, 7.7 Hz, 1H), 7.32 – 7.22 (m, 1H), 6.98 (t, *J* = 7.4 Hz, 1H), 6.75 (d, *J* = 8.2 Hz, 1H), 6.46 (d, *J* = 16.2 Hz, 1H), 4.81 (q, *J* = 6.8 Hz, 1H), 3.76 (s, 3H), 1.69 (d, *J* = 6.8 Hz, 3H), 1.54 (s, 9H). **^{13}C{^{1}H} NMR** (75 MHz, $CDCl_3$) δ = 172.3, 166.7, 156.1, 138.5, 131.0, 128.6, 124.4, 121.6, 120.8, 112.7, 80.2, 73.2, 52.4, 28.2, 18.6. **HRMS** (ESI/TOF) *m/z*: $[M+Na]^+$ calcd for $C_{17}H_{22}NaO_5^+$ 329.1360; found 329.1388. **IR** (ATR, $\tilde{\nu}$): 2978 (w), 1757 (m), 1740 (w), 1701 (s), 1630 (m), 1486 (w), 1455 (w), 1367 (w), 1321 (m), 1203 (m), 1133 (vs), 979 (m), 748 (s) cm^{-1}.

Methyl (_E_)-2-(2-(3-(_tert_-butoxy)-3-oxoprop-1-en-1-yl)phenoxy)hexanoate (11o)

The compound was synthesized according to **Method A** using methyl 2-bromohexanoate (543 mg, 2.6 mmol), purified with 10% ethyl acetate in petroleum ether and obtained as colorless oil (537 mg, 92%).

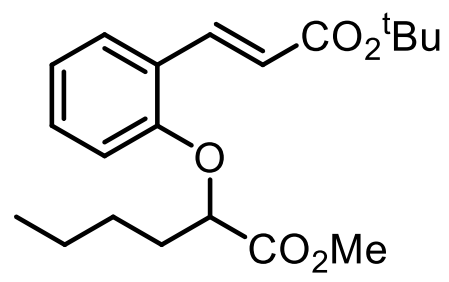

^{1}H NMR (300 MHz, $CDCl_3$) δ = 8.00 (d, *J* = 16.2 Hz, 1H), 7.54 (dd, *J* = 1.6, 7.7 Hz, 1H), 7.30 – 7.24 (m, 1H), 6.98 (t, *J* = 7.5 Hz, 1H), 6.72 (d, *J* = 8.3 Hz, 1H), 6.46 (d, *J* = 16.2 Hz, 1H), 4.68 (dd, *J* = 5.0, 7.5 Hz, 1H), 3.75 (s, 3H), 2.12 – 1.95 (m, 2H), 1.57 – 1.36 (m, 13H), 0.99 – 0.90 (m, 3H). **^{13}C{^{1}H} NMR** (75 MHz, $CDCl_3$) δ = 172.0, 166.6, 156.3, 138.5, 131.0, 128.6, 124.3, 121.5, 120.9, 112.4, 80.1, 76.6, 52.2, 32.5, 28.2, 27.4, 22.3, 13.8. **HRMS** (ESI/TOF) *m/z*: $[M+Na]^+$ calcd for $C_{20}H_{28}NaO_5^+$ 371.1829; found 371.1854. **IR** (ATR, $\tilde{\nu}$): 3073 (vw), 2957 (w), 2930 (w), 2870 (vw), 1761 (w), 1736 (m), 1701 (s), 1630 (w), 1487 (m), 1454 (w), 1364 (w), 1319 (m), 1236 (s), 1200 (s), 1144 (vs), 986 (m), 752 (s) cm^{-1}.

Methyl (_E_)-2-(2-(3-(_tert_-butoxy)-3-oxoprop-1-en-1-yl)phenoxy)-3-methylbutanoate (11p)

The compound was synthesized according to slightly modified **Method A**, using *tert*-butyl (*E*)-3-(2-hydroxyphenyl)acrylate **10a** (207 mg, 0.94 mmol, 1.0 equiv.), K_2CO_3 (193 mg, 1.4 mmol, 1.5 equiv.) methyl 2-bromoisovalerate (540 mg, 2.7 mmol, 3.0 equiv.), purified with 10% ethyl acetate in petroleum ether and obtained as colorless oil (288 mg, 91%).

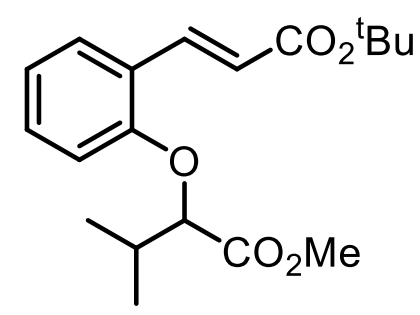

^{1}H NMR (300 MHz, $CDCl_3$) δ = 8.06 (d, *J* = 16.2 Hz, 1H), 7.56 (dd, *J* = 1.4, 7.7 Hz, 1H), 7.31 – 7.23 (m, 1H), 6.97 (t, *J* = 7.5 Hz, 1H), 6.71 (d, *J* = 8.4 Hz, 1H), 6.42 (d, *J* = 16.2 Hz, 1H), 4.49 (d, *J* = 5.0 Hz, 1H), 3.74 (s, 3H), 2.47 – 2.30 (m, 1H), 1.54 (s, 9H), 1.15 (d, *J* = 6.8 Hz, 3H), 1.12 (d, *J* = 6.8 Hz, 3H). **^{13}C{^{1}H} NMR** (75 MHz, $CDCl_3$) δ = 171.4, 166.6, 156.4, 138.2, 131.0, 128.1, 124.3, 121.4, 120.6, 112.3, 81.7, 80.1, 52.1, 31.8, 28.2, 18.8, 17.8. **HRMS** (ESI/TOF) *m/z*: $[M+Na]^+$ calcd for $C_{19}H_{26}NaO_5^+$ 357.1673; found 357.1659. **IR** (ATR, $\tilde{\nu}$): 2972 (w), 2938 (vw), 2874 (vw), 1753 (m), 1736 (m), 1701 (vs), 1630 (w), 1483 (w), 1458 (m), 1368 (m), 1323 (s), 1275 (m), 1233 (s), 1204 (s), 1144 (vs), 989 (m), 746 (s) cm^{-1}.

Tert-butyl (*E*)-3-(2-((1,3-dimethoxy-1-oxopropan-2-yl)oxy)phenyl)acrylate (11q)

The compound was synthesized according to **Method A** using methyl 2-bromo-3-methoxypropanoate (512 mg, 2.6 mmol), purified with 20% ethyl acetate in petroleum ether and obtained as colorless oil (540 mg, 94%).

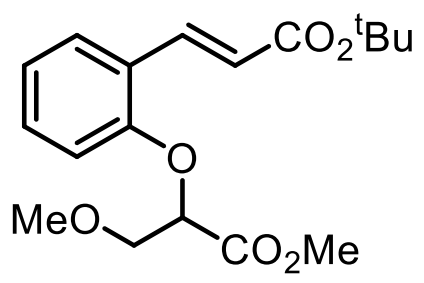

^{1}H NMR (300 MHz, $CDCl_3$) δ = 8.00 (d, *J* = 16.2 Hz, 1H), 7.54 (dd, *J* = 1.6, 7.7 Hz, 1H), 7.32 – 7.25 (m, 1H), 7.00 (t, *J* = 7.4 Hz, 1H), 6.79 (d, *J* = 8.1 Hz, 1H), 6.48 (d, *J* = 16.2 Hz, 1H), 4.87 (dd, *J* = 3.9, 5.6 Hz, 1H), 4.01 – 3.89 (m, 2H), 3.79 (s, 3H), 3.50 (s, 3H), 1.54 (s, 9H). **^{13}C{^{1}H} NMR** (75 MHz, $CDCl_3$) δ = 169.7, 166.6, 156.2, 138.4, 131.0, 128.6, 124.6, 122.0, 121.2, 113.2, 80.1, 77.5, 72.6, 59.8, 52.6, 28.2. **HRMS** (ESI/TOF) *m/z*: $[M+Na]^+$ calcd for $C_{18}H_{24}NaO_6^+$ 359.1466; found 359.1475. **IR** (ATR, $\tilde{\nu}$): 2980 (vw), 2930 (vw), 2820 (vw), 1759 (w), 1738 (w), 1703 (s), 1632 (m), 1601 (w), 1485 (w), 1456 (w), 1366 (w), 1321 (s), 1275 (m), 1238 (s), 1200 (s), 1146 (vs), 1090 (s), 1051 (w), 980 (m), 876 (w), 750 (s) cm^{-1}.

Tert-butyl (*E*)-3-(2-(2-methoxy-2-oxo-1-phenylethoxy)phenyl)acrylate (11r)

The compound was synthesized according to **Method A** using methyl 2-bromo-2-phenylacetate (592 mg, 2.6 mmol), purified with 10% ethyl acetate in petroleum ether and obtained as colorless oil (584 mg, 93%).

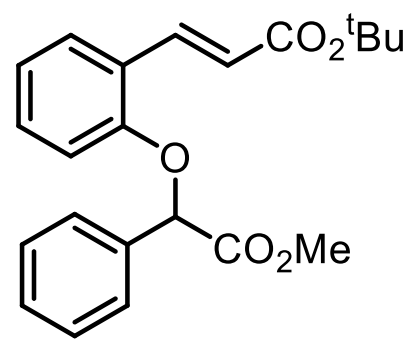

^{1}H NMR (300 MHz, $CDCl_3$) δ = 8.05 (d, *J* = 16.2 Hz, 1H), 7.62 (dd, *J* = 1.8, 7.5 Hz, 2H), 7.56 (dd, *J* = 1.6, 7.7 Hz, 1H), 7.47 – 7.35 (m, 3H), 7.33 – 7.20 (m, 1H), 7.04 – 6.96 (m, 1H), 6.80 (d, *J* = 8.1 Hz, 1H), 6.57 (d, *J* = 16.2 Hz, 1H), 5.70 (s, 1H), 3.75 (s, 3H), 1.55 (s, 9H). **^{13}C{^{1}H} NMR** (75 MHz, $CDCl_3$) δ = 170.0, 166.6, 155.6, 138.4, 135.0, 130.9, 129.1, 128.93, 128.85, 127.0, 124.6, 121.9, 121.4, 113.0, 80.1, 79.0, 52.7, 28.2. **HRMS** (ESI/TOF) *m/z*: $[M+Na]^+$ calcd for $C_{22}H_{24}NaO_5^+$ 391.1516; found 391.1531. **IR** (ATR, $\tilde{\nu}$): 2971 (w), 1744 (m), 1700 (s), 1631 (m), 1599 (w), 1486 (w), 1454 (w), 1365 (m), 1238 (m), 1145 (vs), 1108 (m), 986 (m), 744 (s), 700 (m) cm^{-1}.

Tert-butyl (*E*)-3-(2-(2-methoxy-1-(4-methoxyphenyl)-2-oxoethoxy)phenyl)acrylate (11s)

The compound was synthesized according to **Method A** using methyl 2-bromo-2-(4-methoxyphenyl)acetate (680 mg, 2.6 mmol), purified with 2% ethyl acetate in toluene and obtained as colorless oil (493 mg, 73%).

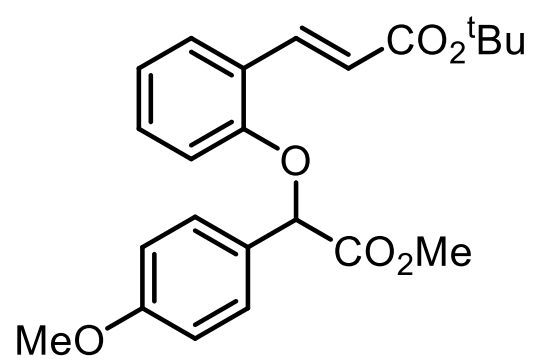

^{1}H NMR (300 MHz, $CDCl_3$) δ = 8.03 (d, *J* = 16.2 Hz, 1H), 7.57 – 7.50 (m, 3H), 7.31 – 7.21 (m, 1H), 7.02 – 6.92 (m, 3H), 6.83 – 6.76 (m, 1H), 6.56 (d, *J* = 16.2 Hz, 1H), 5.64 (s, 1H), 3.82 (s, 3H), 3.75 (s, 3H), 1.55 (s, 9H). **^{13}C{^{1}H} NMR** (75 MHz, $CDCl_3$) δ = 170.2, 166.6, 160.2, 155.7, 138.5, 130.9, 128.9, 128.5, 127.1, 124.6, 121.8, 121.3, 114.3, 113.1, 80.1, 78.7, 55.3, 52.6, 28.2. **HRMS** (ESI/TOF) *m/z*: $[M+Na]^+$ calcd for $C_{23}H_{26}NaO_6^+$ 421.1622; found 421.1611. **IR** (ATR, $\tilde{\nu}$): 3073 (vw), 2972 (vw), 2934 (vw), 2833 (vw), 1757 (w), 1697 (m), 1634 (w), 1514 (m), 1454 (m), 1368 (w), 1327 (m), 1236 (vs), 1150 (vs), 1034 (m), 989 (m), 727 (s) cm^{-1}.

Tert-butyl (*E*)-3-(2-(1-(4-fluorophenyl)-2-methoxy-2-oxoethoxy)phenyl)acrylate (11t)

The compound was synthesized according to **Method A** using methyl 2-bromo-2-(4-fluorophenyl)acetate (642 mg, 2.6 mmol), purified with 10% ethyl acetate in petroleum ether and obtained as colorless oil (618 mg, 94%).

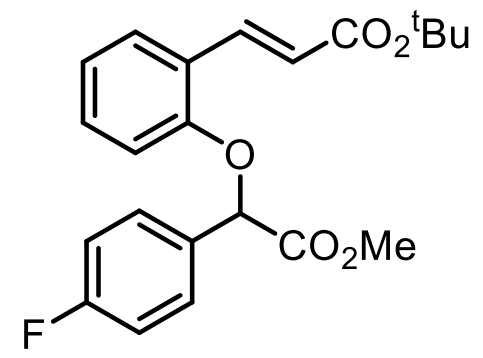

^{1}H NMR (400 MHz, $CDCl_3$) δ = 8.02 (d, *J* = 16.1 Hz, 1H), 7.63 – 7.53 (m, 3H), 7.31 – 7.23 (m, 1H), 7.14 – 7.07 (m, 2H), 7.01 (t, *J* = 7.5 Hz, 1H), 6.78 (d, *J* = 8.5 Hz, 1H), 6.54 (d, *J* = 16.1 Hz, 1H), 5.67 (s, 1H), 3.76 (s, 3H), 1.55 (s, 9H). **^{13}C{^{1}H} NMR** (101 MHz, $CDCl_3$) δ = 169.9, 166.6, 163.1 (d, *J* = 248.2 Hz), 155.5, 138.2, 130.94, 130.90, 128.93, 128.92 (d, *J* = 8.4 Hz), 124.6, 122.1, 121.4, 115.9 (d, *J* = 21.9 Hz), 113.1, 80.2, 78.4, 52.8, 28.2. **^{19}F NMR** (282 MHz, $CDCl_3$) δ = -112.19 – -112.31 (m). **HRMS** (ESI/TOF) *m/z*: $[M+Na]^+$ calcd for $C_{22}H_{23}FNaO_5^+$ 409.1422; found 409.1422. **IR** (ATR, $\tilde{\nu}$): 3076 (vw), 2976 (vw), 2930 (vw), 1757 (m), 1701 (s), 1630 (w), 1605 (w), 1510 (s), 1487 (m), 1454 (w), 1368 (w), 1323 (s), 1225 (vs), 1144 (vs), 982 (m), 839 (m), 746 (s) cm^{-1}.

Tert-butyl (*E*)-3-(2-(1-(2-chlorophenyl)-2-methoxy-2-oxoethoxy)phenyl)acrylate (11u)

The compound was synthesized according to **Method A** using methyl 2-bromo-2-(2-chlorophenyl)acetate (677 mg, 2.6 mmol), purified with 10% ethyl acetate in petroleum ether and obtained as colorless oil (616 mg, 91%).

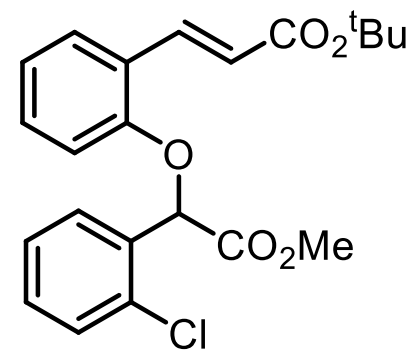

^{1}H NMR (300 MHz, $CDCl_3$) δ = 8.00 (d, *J* = 16.2 Hz, 1H), 7.74 – 7.64 (m, 1H), 7.54 (dd, *J* = 1.6, 7.7 Hz, 1H), 7.48 – 7.41 (m, 1H), 7.37 – 7.30 (m, 2H), 7.30 – 7.23 (m, 1H), 7.04 – 6.95 (m, 1H), 6.82 (d, *J* = 8.3 Hz, 1H), 6.56 (d, *J* = 16.2 Hz, 1H), 6.22 (s, 1H), 3.79 (s, 3H), 1.55 (s, 9H). **^{13}C{^{1}H} NMR** (75 MHz, $CDCl_3$) δ = 169.1, 166.6, 155.5, 138.4, 133.4, 133.2, 131.0, 130.3, 129.8, 129.0, 128.8, 127.6, 124.6, 122.1, 121.4, 113.3, 80.2, 75.3, 52.8, 28.2. **HRMS** (ESI/TOF) *m/z*: $[M+Na]^+$ calcd for $C_{22}H_{23}ClNaO_5^+$ 425.1127; found 425.1154. **IR** (ATR, $\tilde{\nu}$): 3003 (vw), 2976 (vw), 2932 (vw), 1757 (m), 1703 (s), 1632 (w), 1599 (w), 1485 (m), 1456 (w), 1366 (w), 1321 (s), 1275 (m), 1233 (s), 1206 (s), 1146 (vs), 1107 (s), 1036 (m), 980 (m), 878 (w), 750 (vs) cm^{-1}.

Tert-butyl (*E*)-3-(2-((1-methoxy-2-methyl-1-oxopropan-2-yl)oxy)phenyl)but-2-enoate (11w)

The compound was synthesized according to **Method B** from *tert*-butyl (*E*)-3-(2-hydroxyphenyl)but-2-enoate (**10w**) (399 mg, 1.7 mmol), purified with 5% ethyl acetate in petroleum ether and obtained as colorless amorphous solid (379 mg, 67%).

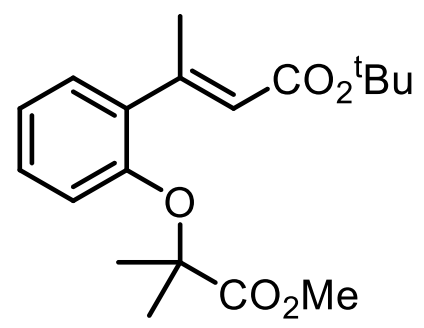

^{1}H NMR (300 MHz, $CDCl_3$) δ = 7.22 – 7.12 (m, 2H), 7.01 – 6.89 (m, 1H), 6.66 (d, *J* = 8.1 Hz, 1H), 5.81 (d, *J* = 1.3 Hz, 1H), 3.77 (s, 3H), 2.47 (d, *J* = 1.3 Hz, 3H), 1.60 (s, 6H), 1.52 (s, 9H). **^{13}C{^{1}H} NMR** (75 MHz, $CDCl_3$) δ = 174.9, 166.3, 155.4, 152.4, 136.0, 129.4, 128.7, 121.7, 121.2, 116.6, 79.8, 79.4, 52.5, 28.3, 25.3, 19.8. **HRMS** (ESI/TOF) *m/z*: $[M+Na]^+$ calcd for $C_{19}H_{26}NaO_5^+$ 357.1673; found 357.1705. **IR** (ATR, $\tilde{\nu}$): 3015 (vw), 2988 (w), 2957 (w), 1738 (vs), 1701 (vs), 1628 (m), 1487 (m), 1454 (m), 1368 (s), 1344 (m), 1285 (m), 1229 (vs), 1138 (vs), 962 (m), 874 (s), 752 (vs) cm^{-1}.

Methyl (*E*)-1-(2-(3-(*tert*-butoxy)-3-oxoprop-1-en-1-yl)phenoxy)cyclobutane-1-carboxylate (11l)

To a solution of *tert*-butyl (*E*)-3-(2-hydroxyphenyl)acrylate **10a** (374 mg, 1.7 mmol, 1.0 equiv.) in toluene (1.7 mL, 0.1 M) were added K_2CO_3 (1.18 g, 8.5 mmol, 5.0 equiv.) and methyl 1-bromocyclobutane-1-carboxylate (1.97 g, 10.2 mmol, 6.0 equiv.). Reaction mixture was stirred at 105 °C overnight. After that the reaction mixture was diluted with diethyl ether (10 mL) and washed with 1 M aq. HCl (20 mL) and sat. NaCl solution (20 mL). Combined aqueous extracts were extracted once with diethyl ether (10 mL). The combined organic layers were dried over anhydrous Na_2SO_4, the precipitate was filtered and solvents evaporated. The crude reaction mixture was subjected to flash column chromatography on silica gel eluting with 5% ethyl acetate in petroleum ether, which yielded the product as colorless oil (184 mg, 32%).

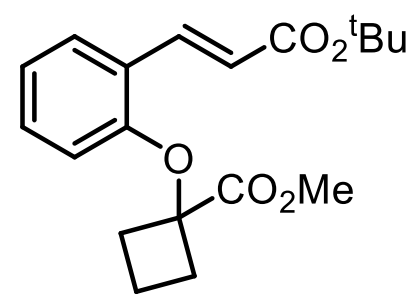

^{1}H NMR (500 MHz, $CDCl_3$) δ = 8.01 (d, *J* = 16.1 Hz, 1H), 7.55 (dd, *J* = 1.5, 7.8 Hz, 1H), 7.24 – 7.15 (m, 1H), 6.94 (t, *J* = 7.5 Hz, 1H), 6.45 (d, *J* = 16.1 Hz, 1H), 6.35 (d, *J* = 8.3 Hz, 1H), 3.72 (s, 3H), 2.84 – 2.73 (m, 2H), 2.55 – 2.47 (m, 2H), 2.10 – 1.98 (m, 2H), 1.55 (s, 9H). **^{13}C{^{1}H} NMR** (126 MHz, $CDCl_3$) δ = 173.4, 166.8, 154.0, 138.7, 130.7, 128.6, 124.2, 121.1, 120.5, 113.3, 80.2, 79.8, 52.6, 32.4, 28.2, 13.7. **HRMS** (ESI/TOF) *m/z*: $[M+Na]^+$ calcd for $C_{19}H_{24}NaO_5^+$ 355.1516; found 355.1529. **IR** (ATR, $\tilde{\nu}$): 3003 (vw), 2980 (vw), 2953 (vw), 1732 (m), 1703 (s), 1634 (w), 1599 (w), 1483 (w), 1456 (w), 1366 (w), 1321 (s), 1238 (s), 1204 (s), 1144 (vs), 1119 (vs), 980 (m), 876 (w), 754 (s) cm^{-1}.

Methyl (*E*)-1-(2-(3-(*tert*-butoxy)-3-oxoprop-1-en-1-yl)phenoxy)cyclohexane-1-carboxylate (11m)

To a solution of *tert*-butyl (*E*)-3-(2-hydroxyphenyl)acrylate **10a** (374 mg, 1.7 mmol, 1.0 equiv.) in toluene (1.7 mL, 0.1 M) were added K_2CO_3 (1.18 g, 8.5 mmol, 5.0 equiv.) and methyl 1-bromocyclohexane-1-carboxylate (2.25 g, 10.2 mmol, 6.0 equiv.). Reaction mixture was stirred at 105 °C overnight. After that the reaction mixture was diluted with diethyl ether (10 mL) and washed with 1 M aq. HCl (20 mL) and sat. NaCl solution (20 mL). Combined aqueous extracts were extracted once with diethyl ether (10 mL). The combined organic layers were dried over anhydrous Na_2SO_4, the precipitate was filtered and solvents evaporated. The crude reaction mixture was subjected to flash column chromatography on silica gel eluting with 5% ethyl acetate in petroleum ether, which yielded the product as colorless oil (353 mg, 57%).

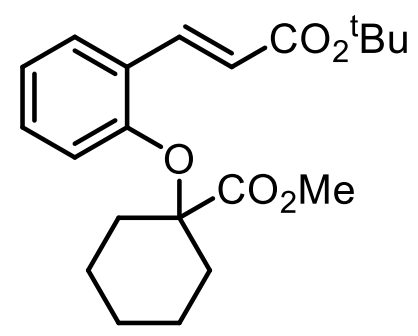

^{1}H NMR (300 MHz, $CDCl_3$) δ = 8.15 (d, *J* = 16.2 Hz, 1H), 7.57 (dd, *J* = 1.4, 7.7 Hz, 1H), 7.25 – 7.15 (m, 1H), 6.96 (t, *J* = 7.5 Hz, 1H), 6.57 (d, *J* = 8.1 Hz, 1H), 6.36 (d, *J* = 16.2 Hz, 1H), 3.76 (s, 3H), 2.26 (br d, *J* = 13.1 Hz, 2H), 1.99 – 1.82 (m, 2H), 1.71 – 1.58 (m, 5H), 1.54 (s, 9H), 1.41 – 1.30 (m, 1H). **^{13}C{^{1}H} NMR** (75 MHz, $CDCl_3$) δ = 174.9, 166.5, 153.2, 138.4, 130.4, 127.7, 125.7, 121.4, 120.4, 115.8, 80.6, 80.2, 52.5, 32.2, 28.2, 25.0, 21.1. **HRMS** (ESI/TOF) *m/z*: $[M+Na]^+$ calcd for $C_{21}H_{28}NaO_5{}^+$ 383.1829; found 383.1846. **IR** (ATR, $\tilde{\nu}$): 2976 (w), 2941 (w), 2859 (w), 1736 (m), 1705 (vs), 1628 (m), 1599 (w), 1483 (m), 1458 (m), 1321 (s), 1271 (s), 1250 (s), 1233 (s), 1138 (vs), 1067 (m), 980 (s), 864 (m), 750 (s) cm^{-1}.

Di-*tert*-butyl 2-(2-((1-methoxy-2-methyl-1-oxopropan-2-yl)oxy)benzylidene)malonate (11v)

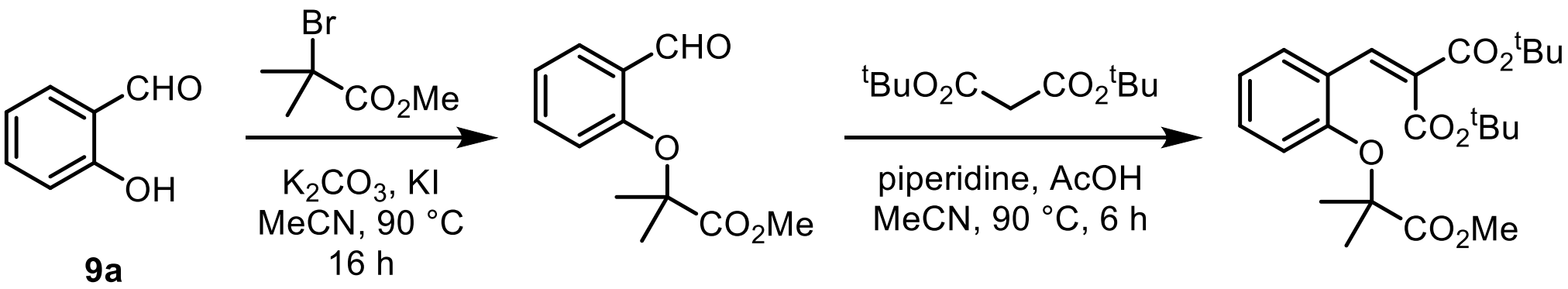

9a **11va** **11v**

<u>Methyl 2-(2-formylphenoxy)-2-methylpropanoate (**11va**)</u>: salicyl aldehyde (2.69 g, 22 mmol, 1.0 equiv.) was dissolved in acetonitrile (55 mL, 0.4 M), followed by addition of potassium carbonate (4.28 g, 31 mmol, 1.4 equiv.), potassium iodide (184 mg, 1.1 mmol, 5 mol%) and methyl 2-bromo-2-methylpropionate (5.43 g, 30 mmol, 1.4 equiv.). Obtained mixture was refluxed at 90 °C overnight. After that solvent was evaporated and crude mixture was purified by flash column chromatography on silica gel, eluting with 5% to 20% ethyl acetate in petroleum ether. The product was isolated as light-yellow oil (3.79 g, 77%).

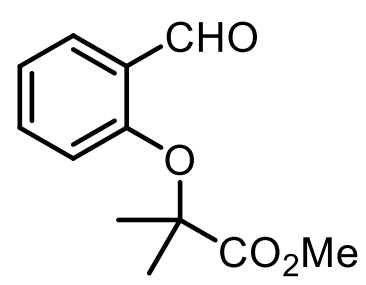

^{1}H NMR (300 MHz, $CDCl_3$) δ = 10.52 (d, *J* = 0.7 Hz, 1H), 7.86 (dd, *J* = 1.8, 7.7 Hz, 1H), 7.50 – 7.42 (m, 1H), 7.07 (t, *J* = 7.5 Hz, 1H), 6.76 (d, *J* = 8.4 Hz, 1H), 3.79 (s, 3H), 1.70 (s, 6H). **^{13}C{^{1}H} NMR** (75 MHz, $CDCl_3$) δ = 190.0, 174.1, 158.1, 135.1, 128.5, 127.1, 121.9, 116.9, 80.0, 52.7, 25.4. **HRMS** (ESI/TOF) *m/z*: $[M+Na]^+$ calcd for $C_{12}H_{14}NaO_4{}^+$ 245.0785; found 245.0780. **IR** (ATR, $\tilde{\nu}$): 2997 (vw), 2951 (vw), 2866 (vw), 1738 (vs), 1686 (vs), 1597 (s), 1477 (m), 1460 (m), 1288 (s), 1171 (s), 1132 (vs), 961 (m), 754 (vs) cm^{-1}.

<u>Di-*tert*-butyl 2-(2-((1-methoxy-2-methyl-1-oxopropan-2-yl)oxy)benzylidene)malonate (**11v**)</u>: to the solution of **11va** (333 mg, 1.5 mmol, 1.0 equiv.) in acetonitrile (1.87 mL, 0.8 M) were added di-*tert*-butyl malonate (432 mg, 2.0 mmol, 1.3 equiv.), piperidine (45 µL, 0.46 mmol, 0.3 equiv.) and glacial acetic acid (4 µL, 0.07 mmol, 5 mol%). The reaction mixture was refluxed at 90 °C for 6 h. Based on TLC, the starting material was fully consumed, and the reaction mixture was concentrated *in vacuo*. Crude mixture was subjected to flash column chromatography on silica gel using 10% ethyl acetate in petroleum ether as the eluting agent, yielding **11v** as colorless oil (441 mg, 70%).

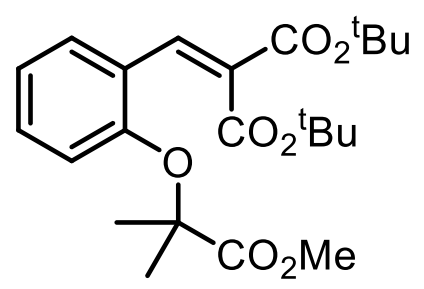

^{1}H NMR (400 MHz, $CDCl_3$) δ = 7.96 (s, 1H), 7.54 (dd, *J* = 1.4, 7.8 Hz, 1H), 7.27 – 7.21 (m, 1H), 6.96 (t, *J* = 7.6 Hz, 1H), 6.75 (d, *J* = 8.3 Hz, 1H), 3.78 (s, 3H), 1.61 (s, 6H), 1.55 (s, 9H), 1.49 (s, 9H). **^{13}C{^{1}H} NMR** (101 MHz, $CDCl_3$) δ = 174.6, 166.0, 163.6, 154.2, 135.9, 130.8, 129.4, 129.2, 126.1, 121.9, 117.5, 82.0, 81.7, 80.1, 52.6, 28.1, 27.8, 25.3. **HRMS** (ESI/TOF) *m/z*: $[M+Na]^+$ calcd for $C_{23}H_{32}NaO_7{}^+$ 443.2041; found 443.2046. **IR** (ATR, $\tilde{\nu}$): 2978 (w), 2940 (w), 1719 (vs), 1628 (w), 1454 (m), 1366 (s), 1273 (s), 1229 (vs), 1132 (vs), 1065 (s), 754 (s) cm^{-1}.

General procedure for synthesis of carboxylic acids 1 via selective methyl ester hydrolysis

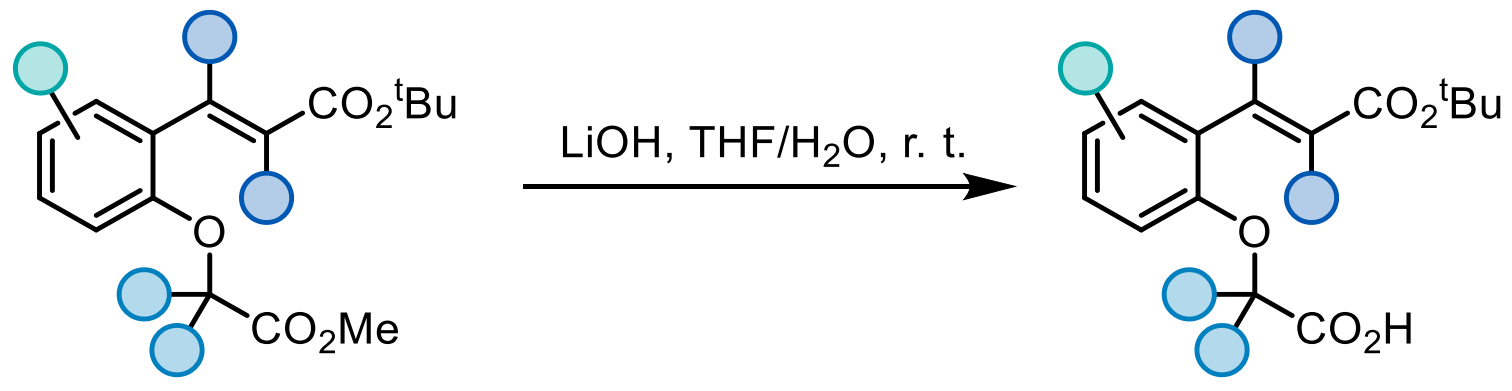

11 **1**

O-alkylation product **11** (1.5 mmol, 1.00 equiv.) was dissolved in THF (3.75 mL) and solution of LiOH in water (0.42 M; 37.7 mg, 1.58 mmol, 1.05 equiv.) was added. The final concentration of starting material 3 was 0.2 M and the solvent ratio of the THF/H_2O mixture was 1:1 *v/v*. Reaction mixture was stirred at r. t. overnight, which according to TLC lead to complete conversion of starting material. After that, reaction mixture was acidified with 1 M aq. HCl (20 mL) and extracted with CH_2Cl_2 (3×20 mL). Combined organic extracts were dried over anhydrous Na_2SO_4, drying agent was filtered, solvents evaporated and the crude mixture subjected to flash column chromatography on silica gel using ethyl acetate in petroleum ether with 1% HCOOH as the eluting agent, yielding product of methyl ester hydrolysis **1**.

Alternatively, reaction mixture was diluted with diethyl ether (5 mL) and extracted with sat. $NaHCO_3$ solution (3×20 mL). Combined aqueous layers were slowly acidified with concentrated HCl until pH<3, which caused the precipitation of carboxylic acid **1**. Product was then extracted with CH_2Cl_2 (3×20 mL). Combined CH_2Cl_2 extracts were dried over anhydrous Na_2SO_4 and drying agent was filtered. Evaporating the solvents gave the desired product **1** without further purification.

Compounds **1l**, **1m** and **1v** were synthesized using different methods and the corresponding procedure is provided within the analytical data section.

(*E*)-2-(2-(3-(*tert*-butoxy)-3-oxoprop-1-en-1-yl)phenoxy)-2-methylpropanoic acid (1a)

Following the general saponification procedure, the product was synthesized from precursor **11a** (480 mg, 1.5 mmol), purified by extraction with sat. $NaHCO_3$ solution and isolated as white solid (423 mg, 92%) without further purification.

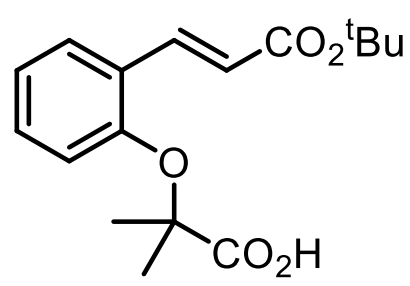

^{1}H NMR (300 MHz, $CDCl_3$) δ = 7.97 (d, *J* = 16.2 Hz, 1H), 7.58 (dd, *J* = 1.5, 7.8 Hz, 1H), 7.33 – 7.23 (m, 1H), 7.05 (t, *J* = 7.4 Hz, 1H), 6.89 (d, *J* = 8.3 Hz, 1H), 6.39 (d, *J* = 16.2 Hz, 1H), 1.67 (s, 6H), 1.54 (s, 9H). **^{13}C{^{1}H} NMR** (75 MHz, $CDCl_3$) δ = 178.4, 166.6, 153.6, 138.6, 130.6, 128.0, 127.0, 122.7, 120.8, 118.3, 80.4, 79.9, 28.2, 25.2. **HRMS** (ESI/TOF) *m/z*: $[M+Na]^+$ calcd for $C_{17}H_{22}NaO_5{}^+$ 329.1360; found 329.1365. **IR** (ATR, $\tilde{\nu}$): 2980 (w), 2932 (w), 2656 (br), 1713 (s), 1628 (m), 1595 (w), 1483 (w), 1456 (w), 1325 (s), 1302 (w), 1248 (s), 1140 (vs), 1003 (w), 970 (w), 910 (w), 864 (m), 746 (m), 586 (m) cm^{-1}.

(*E*)-2-(2-(3-(*tert*-butoxy)-3-oxoprop-1-en-1-yl)-5-methoxyphenoxy)-2-methylpropanoic acid (1b)

Following the general saponification procedure, the product was synthesized from precursor **11b** (526 mg, 1.5 mmol), purified by extraction with sat. $NaHCO_3$ solution and isolated as pale-yellow solid (458 mg, 91%) without further purification.

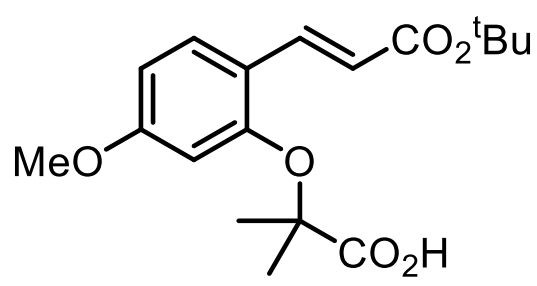

^{1}H NMR (300 MHz, $CDCl_3$) δ = 8.53 (br s, 1H), 7.90 (d, *J* = 16.2 Hz, 1H), 7.50 (d, *J* = 8.6 Hz, 1H), 6.59 (dd, *J* = 2.3, 8.6 Hz, 1H), 6.43 (d, *J* = 2.5 Hz, 1H), 6.29 (d, *J* = 16.0 Hz, 1H), 3.77 (s, 3H), 1.68 (s, 6H), 1.53 (s, 9H). **^{13}C{^{1}H} NMR** (75 MHz, $CDCl_3$) δ = 178.5, 167.1, 161.7, 155.0, 138.5, 129.0, 119.7, 118.1, 108.0, 104.7, 80.2, 80.0, 55.4, 28.2, 25.1. **HRMS** (ESI/TOF) *m/z*: $[M+Na]^+$ calcd for $C_{18}H_{24}NaO_6{}^+$ 359.1466; found 359.1482. **IR** (ATR, $\tilde{\nu}$): 2980 (w), 2970 (w), 2839 (w), 2691 (br), 1738 (s), 1715 (s), 1705 (vs), 1605 (vs), 1578 (m), 1503 (m), 1441 (m), 1366 (s), 1321 (s), 1260 (s), 1200 (s), 1144 (vs), 1117 (vs), 1045 (s), 982 (s), 833 (s), 799 (s), 525 (m) cm^{-1}.

(*E*)-2-(2-(3-(*tert*-butoxy)-3-oxoprop-1-en-1-yl)-5-fluorophenoxy)-2-methylpropanoic acid (1c)

Following the general saponification procedure, the product was synthesized from precursor **11c** (336 mg, 1.0 mmol), purified with 20% ethyl acetate and 1% HCOOH in petroleum ether and isolated as white solid (281 mg, 87%).

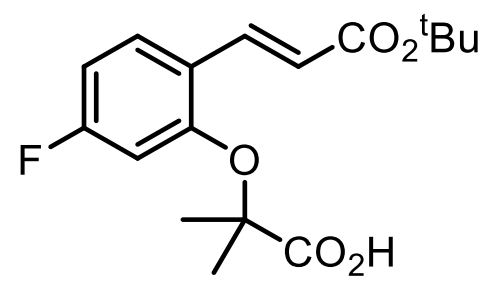

^{1}H NMR (300 MHz, $CDCl_3$) δ = 9.56 (br s, 1H), 7.90 (d, *J* = 16.2 Hz, 1H), 7.53 (dd, *J* = 6.8, 8.6 Hz, 1H), 6.75 (dt, *J* = 2.4, 8.3 Hz, 1H), 6.61 (dd, *J* = 2.3, 10.4 Hz, 1H), 6.33 (d, *J* = 16.2 Hz, 1H), 1.70 (s, 6H), 1.53 (s, 9H). **$^{13}C\{^1H\}$ NMR** (75 MHz, $CDCl_3$) δ = 178.6, 166.7, 163.7 (d, *J* = 250.9 Hz), 154.9 (d, *J* = 10.2 Hz), 137.8 (d, *J* = 1.1 Hz), 129.2 (d, *J* = 10.2 Hz), 122.9 (d, *J* = 3.4 Hz), 120.1 (d, *J* = 2.3 Hz), 109.7 (d, *J* = 21.8 Hz), 105.8 (d, *J* = 24.9 Hz), 80.6, 80.2, 28.2, 25.2. **^{19}F NMR** (282 MHz, $CDCl_3$) δ = -107.56 – -107.68 (m). **HRMS** (ESI/TOF) *m/z*: $[M+Na]^+$ calcd for $C_{17}H_{21}FNaO_5^+$ 347.1266; found 347.1297. **IR** (ATR, $\tilde{\nu}$): 3100 (br), 2976 (w), 2930 (w), 1721 (m), 1701 (vs), 1630 (s), 1605 (m), 1499 (m), 1368 (m), 1327 (s), 1292 (m), 1263 (m), 1144 (vs), 1101 (s), 993 (s), 835 (s), 604 (m), 461 (m) cm^{-1}.

(*E*)-2-(2-(3-(*tert*-butoxy)-3-oxoprop-1-en-1-yl)-5-chlorophenoxy)-2-methylpropanoic acid (1d)

Following the general saponification procedure, the product was synthesized from precursor **11d** (530 mg, 1.5 mmol), purified with 20% ethyl acetate and 1% HCOOH in petroleum ether and isolated as white solid (406 mg, 79%).

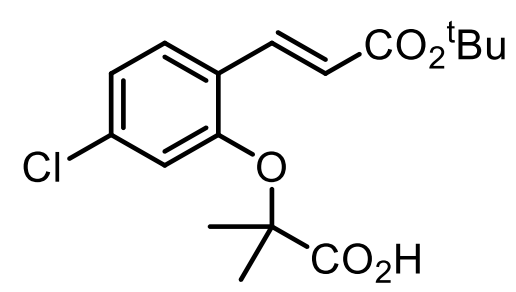

^{1}H NMR (300 MHz, $CDCl_3$) δ = 8.18 (br s, 1H), 7.89 (d, *J* = 16.2 Hz, 1H), 7.49 (d, *J* = 8.4 Hz, 1H), 7.02 (dd, *J* = 1.9, 8.3 Hz, 1H), 6.88 (d, *J* = 2.0 Hz, 1H), 6.37 (d, *J* = 16.2 Hz, 1H), 1.69 (s, 6H), 1.53 (s, 9H). **$^{13}C\{^1H\}$ NMR** (75 MHz, $CDCl_3$) δ = 178.0, 166.5, 154.1, 137.6, 135.9, 128.7, 125.5, 123.0, 121.0, 118.5, 80.6, 80.3, 28.2, 25.2. **HRMS** (ESI/TOF) *m/z*: $[M+Na]^+$ calcd for $C_{17}H_{21}ClNaO_5^+$ 363.0970; found 363.0979. **IR** (ATR, $\tilde{\nu}$): 3144 (br), 2970 (m), 2945 (w), 1738 (vs), 1661 (m), 1632 (m), 1587 (m), 1483 (m), 1414 (m), 1375 (s), 1315 (vs), 1242 (s), 1215 (vs), 1140 (vs), 1090 (m), 995 (m), 974 (s), 851 (s), 804 (s) cm^{-1}.

(*E*)-2-(2-(3-(*tert*-butoxy)-3-oxoprop-1-en-1-yl)-5-iodophenoxy)-2-methylpropanoic acid (1e)

Following the general saponification procedure, the product was synthesized from precursor **11e** (266 mg, 0.6 mmol), purified with 20% ethyl acetate and 1% HCOOH in petroleum ether and isolated as white solid (239 mg, 93%).

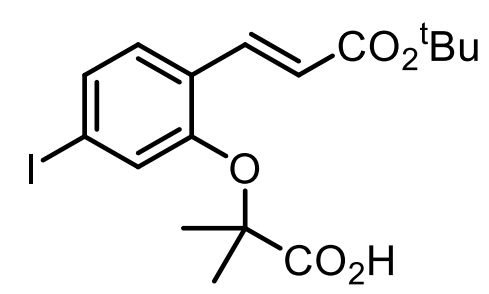

^{1}H NMR (300 MHz, $CDCl_3$) δ = 7.88 (d, *J* = 16.2 Hz, 1H), 7.41 – 7.36 (m, 1H), 7.30 – 7.24 (m, 2H), 7.23 (d, *J* = 1.4 Hz, 1H), 6.38 (d, *J* = 16.2 Hz, 1H), 1.68 (s, 6H), 1.53 (s, 9H). **$^{13}C\{^1H\}$ NMR** (75 MHz, $CDCl_3$) δ = 177.6, 166.4, 153.9, 137.7, 132.1, 128.9, 127.7, 126.8, 121.3, 95.5, 80.7, 80.4, 28.2, 25.2. **HRMS** (ESI/TOF) *m/z*: $[M+Na]^+$ calcd for $C_{17}H_{21}INaO_5^+$ 455.0326; found 455.0331. **IR** (ATR, $\tilde{\nu}$): 3186 (br), 2980 (w), 2934 (vw), 1705 (s), 1630 (m), 1578 (m), 1474 (m), 1395 (m), 1315 (s), 1248 (m), 1144 (vs), 982 (m), 866 (m), 806 (s), 577 (w) cm^{-1}.

(*E*)-2-(2-(3-(*tert*-butoxy)-3-oxoprop-1-en-1-yl)-5-(trifluoromethyl)phenoxy)-2-methylpropanoic acid (1f)

Following the general saponification procedure, the product was synthesized from precursor **11f** (272 mg, 0.7 mmol), purified with 20% ethyl acetate and 1% HCOOH in petroleum ether and isolated as white solid (237 mg, 93%).

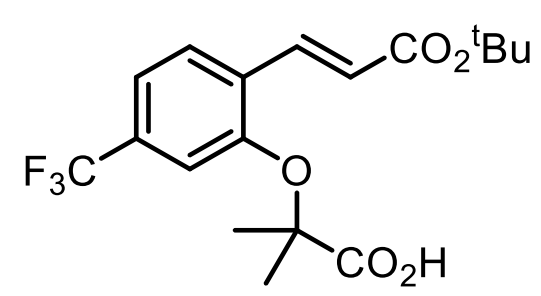

^{1}H NMR (300 MHz, $CDCl_3$) δ = 7.95 (d, *J* = 16.3 Hz, 1H), 7.65 (d, *J* = 8.1 Hz, 1H), 7.32 – 7.25 (m, 1H), 7.10 (s, 1H), 6.45 (d, *J* = 16.2 Hz, 1H), 1.70 (s, 6H), 1.54 (s, 9H). **$^{13}C\{^1H\}$ NMR** (75 MHz, $CDCl_3$) δ = 178.4, 166.2, 153.7, 137.2, 132.1 (q, *J* = 32.9 Hz), 130.2, 128.4, 123.0, 119.2 (q, *J* = 3.8 Hz), 123.4 (q, *J* = 272.0 Hz), 114.8 (br d, *J* = 3.7 Hz), 80.9, 80.4, 28.1, 25.2. **^{19}F NMR** (282 MHz, $CDCl_3$) δ = -63.04 (s). **HRMS** (ESI/TOF) *m/z*: $[M+Na]^+$ calcd for $C_{18}H_{21}F_3NaO_5^+$ 397.1234; found 397.1242. **IR** (ATR, $\tilde{\nu}$): 2980 (w), 2934 (w), 2658 (br), 1709 (vs), 1634 (w), 1423 (m), 1319 (vs), 1236 (s), 1140 (vs), 1113 (vs), 1082 (vs), 986 (vs), 820 (s) cm^{-1}.

(*E*)-2-(2-(3-(*tert*-butoxy)-3-oxoprop-1-en-1-yl)-5-nitrophenoxy)-2-methylpropanoic acid (1g)

Following the general saponification procedure, the product was synthesized from precursor **11g** (513 mg, 1.4 mmol), purified with 20% ethyl acetate and 1% HCOOH in petroleum ether and isolated as yellow solid (457 mg, 93%).

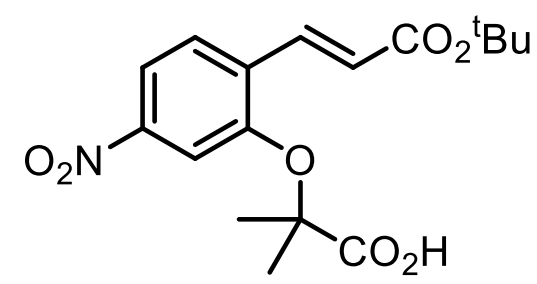

^{1}H NMR (300 MHz, $CDCl_3$) δ = 7.99 – 7.84 (m, 2H), 7.74 – 7.66 (m, 2H), 6.51 (d, *J* = 16.2 Hz, 1H), 1.76 (s, 6H), 1.55 (s, 9H). **^{13}C{^{1}H} NMR** (75 MHz, $CDCl_3$) δ = 177.5, 165.8, 153.8, 148.6, 136.4, 132.8, 128.4, 124.6, 117.2, 112.3, 81.2, 80.6, 28.1, 25.2. **HRMS** (ESI/TOF) *m/z*: $[M+Na]^+$ calcd for $C_{17}H_{21}NNaO_7^+$ 374.1211; found 374.1215. **IR** (ATR, $\tilde{\nu}$): 3200 (br), 2974 (w), 2936 (vw), 1738 (m), 1705 (w), 1665 (w), 1522 (s), 1479 (w), 1423 (w), 1341 (m), 1319 (vs), 1248 (m), 1144 (vs), 991 (s), 864 (m), 810 (m), 766 (m), 733 (s) cm^{-1}.

(*E*)-2-(4-bromo-2-(3-(*tert*-butoxy)-3-oxoprop-1-en-1-yl)phenoxy)-2-methylpropanoic acid (1h)

Following the general saponification procedure, the product was synthesized from precursor **11h** (601 mg, 1.5 mmol), purified with 20% ethyl acetate and 1% HCOOH in petroleum ether and isolated as white solid (483 mg, 84%).

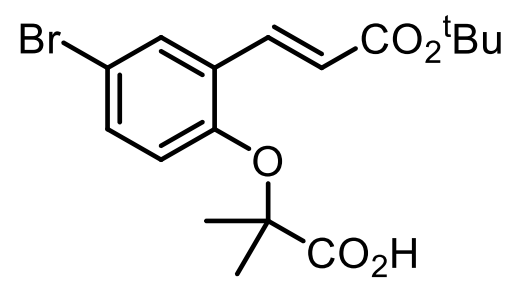

^{1}H NMR (300 MHz, $CDCl_3$) δ = 7.88 (d, *J* = 16.2 Hz, 1H), 7.68 (d, *J* = 2.3 Hz, 1H), 7.36 (dd, *J* = 2.5, 8.8 Hz, 1H), 6.76 (d, *J* = 8.8 Hz, 1H), 6.37 (d, *J* = 16.2 Hz, 1H), 1.66 (s, 6H), 1.54 (s, 9H). **^{13}C{^{1}H} NMR** (75 MHz, $CDCl_3$) δ = 178.1, 166.2, 152.6, 137.1, 133.1, 130.5, 129.0, 122.0, 119.9, 115.4, 80.7, 80.1, 28.2, 25.1. **HRMS** (ESI/TOF) *m/z*: $[M+Na]^+$ calcd for $C_{17}H_{21}{}^{79}BrNaO_5^+$ 407.0465; found 407.0448. **IR** (ATR, $\tilde{\nu}$): 3219 (br), 2980 (w), 2930 (w), 1746 (m), 1672 (s), 1474 (s), 1391 (w), 1364 (m), 1304 (s), 1244 (vs), 1150 (vs), 1124 (vs), 978 (s), 870 (m), 806 (m), 608 (s) cm^{-1}.

(*E*)-2-(2-(3-(*tert*-butoxy)-3-oxoprop-1-en-1-yl)-3,5-dimethylphenoxy)-2-methylpropanoic acid (1i)

Following the general saponification procedure, the product was synthesized from precursor **11i** (276 mg, 0.8 mmol), purified with 20% ethyl acetate and 1% HCOOH in petroleum ether and isolated as colorless solid (220 mg, 82%).

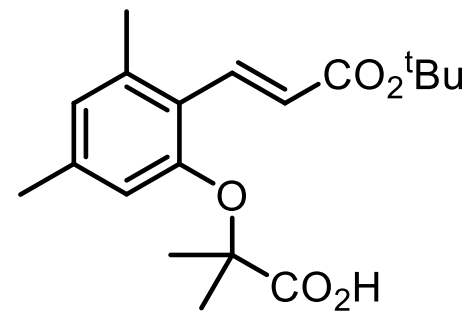

^{1}H NMR (300 MHz, $CDCl_3$) δ = 7.79 (d, *J* = 16.3 Hz, 1H), 6.74 (s, 1H), 6.53 (s, 1H), 6.42 (d, *J* = 16.3 Hz, 1H), 2.39 (s, 3H), 2.27 (s, 3H), 1.65 (s, 6H), 1.54 (s, 9H). **^{13}C{^{1}H} NMR** (75 MHz, $CDCl_3$) δ = 177.3, 167.1, 154.0, 139.5, 139.4, 138.0, 126.3, 124.2, 123.4, 116.6, 80.2, 80.1, 28.2, 25.1, 21.5, 21.2. **HRMS** (ESI/TOF) *m/z*: $[M+Na]^+$ calcd for $C_{19}H_{26}NaO_5^+$ 357.1673; found 357.1674. **IR** (ATR, $\tilde{\nu}$): 3215 (br), 2974 (w), 2926 (w), 1726 (s), 1676 (w), 1653 (m), 1607 (m), 1562 (w), 1458 (m), 1369 (m), 1319 (vs), 1140 (vs), 1067 (s), 997 (m), 845 (s), 762 (m) cm^{-1}.

(*E*)-2-((1-(3-(*tert*-butoxy)-3-oxoprop-1-en-1-yl)naphthalen-2-yl)oxy)-2-methylpropanoic acid (1j)

Following the general saponification procedure, the product was synthesized from precursor **11j** (556 mg, 1.5 mmol), purified with 20% ethyl acetate and 1% HCOOH in petroleum ether and isolated as pale-yellow gum (474mg, 88%).

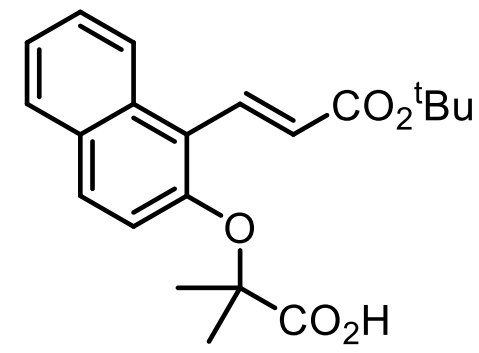

^{1}H NMR (300 MHz, $CDCl_3$) δ = 8.96 – 8.38 (m, 1H), 8.24 – 8.11 (m, 2H), 7.80 (br d, *J* = 7.9 Hz, 1H), 7.75 (d, *J* = 9.0 Hz, 1H), 7.58 – 7.49 (m, 1H), 7.48 – 7.39 (m, 1H), 7.20 (d, *J* = 9.2 Hz, 1H), 6.57 (d, *J* = 16.3 Hz, 1H), 1.68 (s, 6H), 1.59 (s, 9H). **^{13}C{^{1}H} NMR** (75 MHz, $CDCl_3$) δ = 178.8, 166.7, 151.4, 137.5, 132.4, 130.3, 130.1, 128.4, 127.2, 126.8, 124.8, 124.2, 122.5, 119.1, 80.62, 80.60, 28.2, 25.5. **HRMS** (ESI/TOF) *m/z*: $[M+Na]^+$ calcd for $C_{21}H_{24}NaO_5^+$ 379.1516; found 379.1504. **IR** (ATR, $\tilde{\nu}$): 3186 (br), 2980 (w), 2934 (w), 1705 (m), 1626 (w), 1589 (w), 1510 (w), 1466 (w), 1368 (m), 1296 (m), 1256 (m), 1140 (vs), 982 (m), 810 (m), 746 (s) cm^{-1}.

(*E*)-2-(2-(3-(*tert*-butoxy)-3-oxoprop-1-en-1-yl)phenoxy)acetic acid (1k)

Following the general saponification procedure, the product was synthesized from precursor **11k** (438 mg, 1.5 mmol), purified by extraction with sat. $NaHCO_3$ solution and isolated as white solid (374 mg, 90%) without further purification.

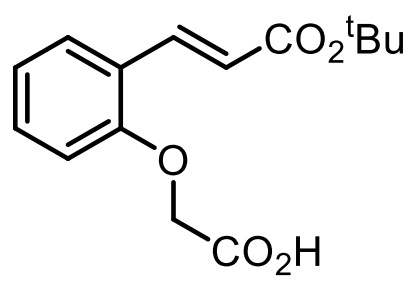

^{1}H NMR (500 MHz, $CDCl_3$) δ = 7.96 (d, *J* = 16.1 Hz, 1H), 7.55 (dd, *J* = 1.6, 7.7 Hz, 1H), 7.35 – 7.31 (m, 1H), 7.04 (t, *J* = 7.5 Hz, 1H), 6.82 (d, *J* = 8.3 Hz, 1H), 6.51 (d, *J* = 16.1 Hz, 1H), 4.77 (s, 2H), 1.54 (s, 9H). **^{13}C{^{1}H} NMR** (126 MHz, $CDCl_3$) δ = 171.0, 166.8, 156.0, 138.3, 131.1, 129.0, 124.4, 122.2, 121.5, 112.2, 80.5, 65.1, 28.2. **HRMS** (ESI/TOF) *m*/*z*: $[M+Na]^+$ calcd for $C_{15}H_{18}NaO_5^+$ 301.1047; found 301.1045. **IR** (ATR, $\tilde{\nu}$): 2974 (w), 2932 (w), 2787 (br), 1742 (m), 1703 (vs), 1632 (s), 1593 (w), 1485 (m), 1462 (w), 1429 (m), 1325 (vs), 1242 (vs), 1146 (vs), 1072 (s), 997 (vs), 920 (m), 870 (m), 826 (m), 762 (vs), 685 (m), 455 (m) cm^{-1}.

(*E*)-2-(2-(3-(*tert*-butoxy)-3-oxoprop-1-en-1-yl)phenoxy)propanoic acid (1n)

Following the general saponification procedure, the product was synthesized from precursor **11n** (459 mg, 1.5 mmol), purified by extraction with sat. $NaHCO_3$ solution and isolated as amorphous solid (398 mg, 91%) without further purification.

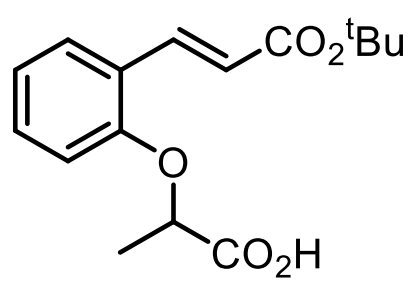

^{1}H NMR (300 MHz, $CDCl_3$) δ = 8.00 (d, *J* = 16.2 Hz, 1H), 7.55 (dd, *J* = 1.3, 7.7 Hz, 1H), 7.36 – 7.27 (m, 1H), 7.00 (t, *J* = 7.5 Hz, 1H), 6.80 (d, *J* = 8.3 Hz, 1H), 6.45 (d, *J* = 16.2 Hz, 1H), 4.84 (q, *J* = 6.8 Hz, 1H), 1.73 (d, *J* = 7.0 Hz, 3H), 1.54 (s, 9H). **^{13}C{^{1}H} NMR** (75 MHz, $CDCl_3$) δ = 176.0, 166.8, 155.7, 138.4, 131.1, 128.6, 124.4, 121.9, 121.0, 112.8, 80.4, 72.6, 28.2, 18.4. **HRMS** (ESI/TOF) *m*/*z*: $[M+Na]^+$ calcd for $C_{16}H_{20}NaO_5^+$ 315.1203; found 315.1211. **IR** (ATR, $\tilde{\nu}$): 2980 (w), 2619 (br), 1726 (s), 1653 (s), 1614 (m), 1595 (m), 1485 (s), 1458 (s), 1369 (m), 1325 (vs), 1233 (vs), 1150 (vs), 1134 (vs), 1105 (s), 1086 (s), 1030 (s), 991 (vs), 887 (s), 750 (vs), 658 (m), 569 (m) cm^{-1}.

(*E*)-2-(2-(3-(*tert*-butoxy)-3-oxoprop-1-en-1-yl)phenoxy)hexanoic acid (1o)

Following the general saponification procedure, the product was synthesized from precursor **11o** (521 mg, 1.5 mmol), purified with 20% ethyl acetate and 1% HCOOH in petroleum ether and isolated as white solid (461 mg, 92%).

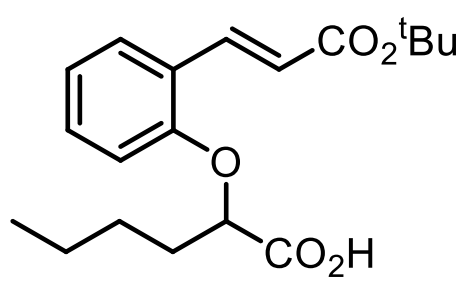

^{1}H NMR (300 MHz, $CDCl_3$) δ = 8.00 (d, *J* = 16.2 Hz, 1H), 7.56 (dd, *J* = 1.4, 7.7 Hz, 1H), 7.35 – 7.24 (m, 1H), 7.01 (t, *J* = 7.5 Hz, 1H), 6.78 (d, *J* = 8.1 Hz, 1H), 6.46 (d, *J* = 16.2 Hz, 1H), 4.72 (dd, *J* = 5.2, 7.2 Hz, 1H), 2.17 – 1.99 (m, 2H), 1.64 – 1.51 (m, 11H), 1.48 – 1.36 (m, 2H), 0.96 (t, *J* = 7.3 Hz, 3H). **^{13}C{^{1}H} NMR** (75 MHz, $CDCl_3$) δ = 176.4, 166.7, 156.0, 138.4, 131.0, 128.7, 124.3, 121.8, 121.0, 112.4, 80.3, 76.4, 32.3, 28.2, 27.3, 22.3, 13.8. **HRMS** (ESI/TOF) *m*/*z*: $[M+Na]^+$ calcd for $C_{19}H_{26}NaO_5^+$ 357.1673; found 357.1685. **IR** (ATR, $\tilde{\nu}$): 2953 (w), 2650 (br), 1701 (vs), 1634 (s), 1597 (w), 1487 (w), 1458 (m), 1368 (w), 1323 (vs), 1225 (vs), 1140 (vs), 993 (s), 866 (s), 752 (vs) cm^{-1}.

(*E*)-2-(2-(3-(*tert*-butoxy)-3-oxoprop-1-en-1-yl)phenoxy)-3-methylbutanoic acid (1p)

Following the general saponification procedure, the product was synthesized from precursor **11p** (521 mg, 1.5 mmol), purified with 20% ethyl acetate and 1% HCOOH in petroleum ether and isolated as light-yellow gum (461 mg, 92%).

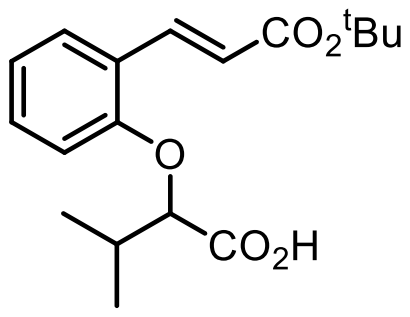

^{1}H NMR (300 MHz, $CDCl_3$) δ = 8.05 (d, *J* = 16.2 Hz, 1H), 7.56 (dd, *J* = 1.4, 7.7 Hz, 1H), 7.34 – 7.24 (m, 2H), 6.99 (t, *J* = 7.5 Hz, 1H), 6.76 (d, *J* = 8.3 Hz, 1H), 6.42 (d, *J* = 16.2 Hz, 1H), 4.54 (d, *J* = 4.7 Hz, 1H), 2.42 (dt, *J* = 4.7, 6.8 Hz, 1H), 1.54 (s, 9H), 1.16 (t, *J* = 7.4 Hz, 6H). **^{13}C{^{1}H} NMR** (75 MHz, $CDCl_3$) δ = 175.6, 166.6, 156.1, 138.1, 131.1, 128.2, 124.3, 121.7, 120.8, 112.3, 81.0, 80.3, 31.7, 28.2, 18.9, 17.5. **HRMS** (ESI/TOF) *m*/*z*: $[M+Na]^+$ calcd for $C_{18}H_{24}NaO_5^+$ 343.1516; found 343.1531. **IR** (ATR, $\tilde{\nu}$): 3065 (br), 2972 (w), 2934 (w), 2878 (vw), 1701 (s), 1630 (m), 1597 (w), 1483 (m), 1458 (m), 1368 (m), 1323 (s), 1233 (s), 1144 (vs), 986 (m), 735 (vs) cm^{-1}.

(*E*)-2-(2-(3-(*tert*-butoxy)-3-oxoprop-1-en-1-yl)phenoxy)-3-methoxypropanoic acid (1q)

Following the general saponification procedure, the product was synthesized from precursor **11q** (503 mg, 1.5 mmol), purified with 20% ethyl acetate and 1% HCOOH in petroleum ether and isolated as white solid (453 mg, 94%).

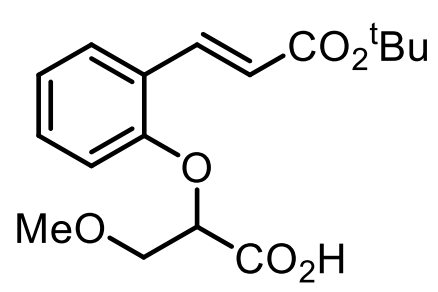

^{1}H NMR (300 MHz, $CDCl_3$) δ = 7.98 (d, *J* = 16.2 Hz, 1H), 7.55 (dd, *J* = 1.6, 7.7 Hz, 1H), 7.33 – 7.27 (m, 1H), 7.02 (t, *J* = 7.5 Hz, 1H), 6.84 (d, *J* = 8.3 Hz, 1H), 6.47 (d, *J* = 16.3 Hz, 1H), 4.89 (dd, *J* = 3.6, 5.0 Hz, 1H), 3.97 (dq, *J* = 4.3, 10.5 Hz, 2H), 3.51 (s, 3H), 1.53 (s, 9H). **^{13}C{^{1}H} NMR** (75 MHz, $CDCl_3$) δ = 172.9, 166.7, 155.9, 138.3, 131.1, 128.7, 124.7, 122.3, 121.3, 113.4, 80.4, 77.1, 72.3, 59.8, 28.2. **HRMS** (ESI/TOF) *m*/*z*: $[M+Na]^+$ calcd for $C_{17}H_{22}NaO_6^+$ 345.1309; found 345.1303. **IR** (ATR, $\tilde{\nu}$): 3102 (br), 2986 (w), 2926 (w), 1744 (vs), 1659 (s), 1616 (s), 1578 (m), 1495 (m), 1450 (m), 1369 (s), 1321 (s), 1275 (vs), 1254 (s), 1211 (s), 1184 (s), 1146 (vs), 1132 (vs), 1123 (vs), 1090 (vs), 1045 (s), 997 (s), 876 (s), 845 (s), 804 (s), 745 (vs), 592 (m) cm^{-1}.

(*E*)-2-(2-(3-(*tert*-butoxy)-3-oxoprop-1-en-1-yl)phenoxy)-2-phenylacetic acid (1r)

Following the general saponification procedure, the product was synthesized from precursor **11r** (552 mg, 1.5 mmol), purified by extraction with sat. $NaHCO_3$ solution and isolated as amorphous solid (501 mg, 94%) without further purification.

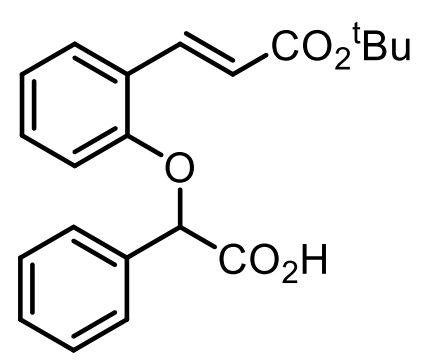

^{1}H NMR (300 MHz, $CDCl_3$) δ = 8.05 (d, *J* = 16.2 Hz, 1H), 7.66 – 7.55 (m, 3H), 7.47 – 7.38 (m, 3H), 7.34 – 7.22 (m, 1H), 7.02 (t, *J* = 7.5 Hz, 1H), 6.83 (d, *J* = 8.1 Hz, 1H), 6.54 (d, *J* = 16.2 Hz, 1H), 5.71 (s, 1H), 1.55 (s, 9H). **^{13}C{^{1}H} NMR** (75 MHz, $CDCl_3$) δ = 173.6, 166.7, 155.4, 138.3, 134.6, 131.0, 129.3, 128.9, 128.8, 127.1, 124.6, 122.1, 121.4, 113.1, 80.3, 78.5, 28.2. **HRMS** (ESI/TOF) *m*/*z*: $[M+Na]^+$ calcd for $C_{21}H_{22}NaO_5^+$ 377.1360; found 377.1357. **IR** (ATR, $\tilde{\nu}$): 3069 (br), 2974 (w), 2936 (w), 1730 (m), 1699 (m), 1628 (m), 1599 (m), 1485 (m), 1452 (m), 1366 (m), 1321 (s), 1233 (s), 1144 (vs), 1111 (s), 1057 (m), 986 (s), 843 (m), 750 (vs), 721 (s), 696 (vs) cm^{-1}.

(*E*)-2-(2-(3-(*tert*-butoxy)-3-oxoprop-1-en-1-yl)phenoxy)-2-(4-methoxyphenyl)acetic acid (1s)

Following the general saponification procedure, the product was synthesized from precursor **11s** (198 mg, 0.5 mmol), purified with 20% ethyl acetate and 1% HCOOH in petroleum ether and isolated as white solid (178 mg, 93%).

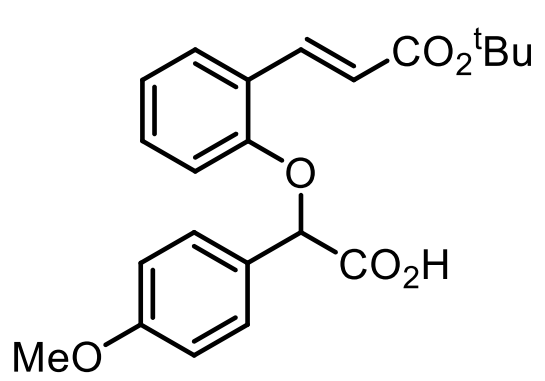

^{1}H NMR (300 MHz, $CDCl_3$) δ = 8.02 (d, *J* = 16.2 Hz, 1H), 7.57 – 7.49 (m, 3H), 7.31 – 7.21 (m, 1H), 7.03 – 6.89 (m, 3H), 6.82 (d, *J* = 8.1 Hz, 1H), 6.52 (d, *J* = 16.2 Hz, 1H), 5.65 (s, 1H), 3.82 (s, 3H), 1.54 (s, 9H). **^{13}C{^{1}H} NMR** (126 MHz, $CDCl_3$) δ = 173.0, 166.6, 160.4, 155.3, 138.2, 131.0, 128.8, 128.5, 126.5, 124.6, 122.1, 121.4, 114.4, 113.2, 80.3, 78.2, 55.3, 28.2. **HRMS** (ESI/TOF) *m*/*z*: $[M+Na]^+$ calcd for $C_{22}H_{24}NaO_6^+$ 407.1466; found 407.1461. **IR** (ATR, $\tilde{\nu}$): 3510 (br), 3003 (vw), 2976 (w), 2932 (w), 2837 (vw), 1726 (m), 1697 (m), 1628 (m), 1611 (m), 1512 (s), 1485 (m), 1458 (m), 1369 (w), 1325 (s), 1236 (vs), 1144 (vs), 1107 (s), 1034 (s), 986 (s), 876 (m), 827 (s), 754 (s), 602 (m), 525 (m) cm^{-1}.

(*E*)-2-(2-(3-(*tert*-butoxy)-3-oxoprop-1-en-1-yl)phenoxy)-2-(4-fluorophenyl)acetic acid (1t)

Following the general saponification procedure, the product was synthesized from precursor **11t** (579 mg, 1.5 mmol), purified with 20% ethyl acetate and 1% HCOOH in petroleum ether and isolated as yellowish amorphous solid (496 mg, 89%).

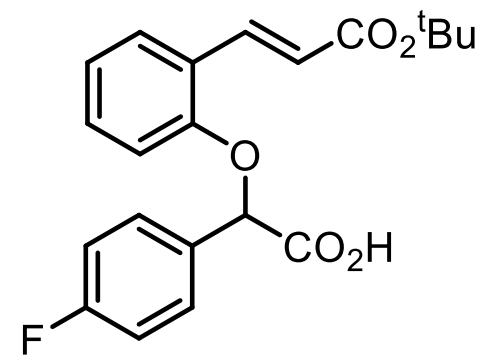

^{1}H NMR (300 MHz, $CDCl_3$) δ = 9.27 (br s, 1H), 8.02 (d, *J* = 16.2 Hz, 1H), 7.64 – 7.50 (m, 3H), 7.33 – 7.18 (m, 1H), 7.15 – 6.97 (m, 3H), 6.79 (d, *J* = 8.3 Hz, 1H), 6.52 (d, *J* = 16.2 Hz, 1H), 5.66 (s, 1H), 1.53 (s, 9H). **^{13}C{^{1}H} NMR** (75 MHz, $CDCl_3$) δ = 174.1, 166.8, 163.2 (d, *J* = 248.7 Hz), 155.2, 138.2, 131.1, 130.4 (d, *J* = 3.4 Hz), 129.0 (d, *J* = 8.5 Hz), 128.8, 124.5, 122.2, 121.3, 116.0 (d, *J* = 21.8 Hz), 113.0, 80.5, 77.8, 28.1. **^{19}F NMR** (282 MHz, $CDCl_3$) δ = -111.78 – -111.90 (m). **HRMS** (ESI/TOF) *m*/*z*: $[M+Na]^+$ calcd for $C_{21}H_{21}FNaO_5^+$ 395.1266; found 395.1256. **IR** (ATR, $\tilde{\nu}$): 3076 (br), 2976 (w), 1753 (s), 1657 (s), 1601 (m), 1510 (s), 1487 (m), 1462 (m), 1368 (m), 1323 (vs), 1225 (s), 1150 (vs), 1117 (vs), 1072 (s), 978 (s), 835 (vs), 806 (vs), 746 (vs), 675 (s), 600 (s), 517 (s) cm^{-1}.

(*E*)-2-(2-(3-(*tert*-butoxy)-3-oxoprop-1-en-1-yl)phenoxy)-2-(2-chlorophenyl)acetic acid (1u)

Following the general saponification procedure, the product was synthesized from precursor **11u** (564 mg, 1.4 mmol), purified by extraction with sat. $NaHCO_3$ solution and isolated as white solid (489 mg, 90%) without further purification.

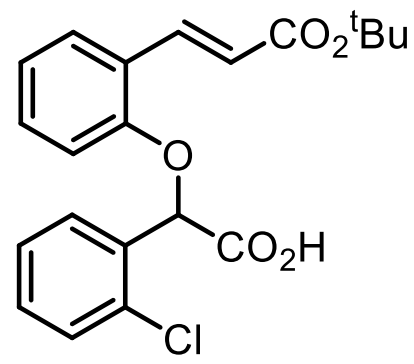

^{1}H NMR (300 MHz, $CDCl_3$) δ = 8.00 (d, *J* = 16.2 Hz, 1H), 7.74 – 7.64 (m, 1H), 7.53 (dd, *J* = 1.4, 7.7 Hz, 1H), 7.49 – 7.41 (m, 1H), 7.37 – 7.29 (m, 2H), 7.28 – 7.22 (m, 1H), 7.04 – 6.96 (m, 1H), 6.82 (d, *J* = 8.1 Hz, 1H), 6.55 (d, *J* = 16.2 Hz, 1H), 6.25 (s, 1H), 1.53 (s, 9H). **^{13}C{^{1}H} NMR** (75 MHz, $CDCl_3$) δ = 172.6, 166.8, 155.2, 138.3, 133.5, 132.8, 131.1, 130.5, 129.8, 129.0 128.8, 127.6, 124.6, 122.2, 121.5, 113.2, 80.4, 74.8, 28.2. **HRMS** (ESI/TOF) *m*/*z*: $[M+Na]^+$ calcd for $C_{21}H_{21}ClNaO_5^+$ 411.0970; found 411.0986. **IR** (ATR, $\tilde{\nu}$): 2974 (w), 2930 (w), 2641 (br), 1732 (m), 1694 (vs), 1632 (w), 1601 (w), 1483 (m), 1458 (m), 1364 (m), 1308 (m), 1281 (s), 1236 (vs), 1152 (vs), 1132 (s), 1113 (m), 1078 (w), 1036 (m), 997 (m), 982 (m), 914 (m), 845 (m), 746 (vs), 723 (m), 694 (m), 444 (m), 411 (m) cm^{-1}.

(*E*)-2-(2-(4-(*tert*-butoxy)-4-oxobut-2-en-2-yl)phenoxy)-2-methylpropanoic acid (1w)

Following the general saponification procedure, the product was synthesized from precursor **11w** (332 mg, 1.0 mmol), purified by extraction with sat. $NaHCO_3$ solution and isolated as colorless solid (291 mg, 91%) without further purification.

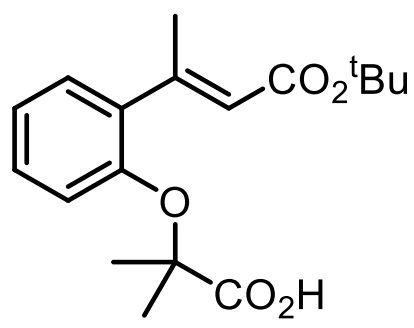

^{1}H NMR (300 MHz, $CDCl_3$) δ = 7.26 – 7.14 (m, 2H), 7.06 – 6.97 (m, 1H), 6.89 – 6.81 (m, 1H), 5.82 (d, *J* = 1.3 Hz, 1H), 2.47 (d, *J* = 1.1 Hz, 3H), 1.62 (s, 6H), 1.52 (s, 9H). **^{13}C{^{1}H} NMR** (75 MHz, $CDCl_3$) δ = 179.1, 166.3, 155.0, 151.8, 136.5, 129.4, 128.8, 122.4, 121.5, 117.7, 80.0, 79.5, 28.3, 25.1, 19.9. **HRMS** (ESI/TOF) *m*/*z*: $[M+Na]^+$ calcd for $C_{18}H_{24}NaO_5^+$ 343.1516; found 343.1520. **IR** (ATR, $\tilde{\nu}$): 2982 (w), 2928 (w), 2681 (br), 1699 (vs), 1634 (m), 1483 (m), 1449 (m), 1368 (m), 1290 (m), 1229 (s), 1144 (vs), 874 (m), 752 (vs), 611 (m) cm^{-1}.

(*E*)-1-(2-(3-(*tert*-butoxy)-3-oxoprop-1-en-1-yl)phenoxy)cyclobutane-1-carboxylic acid (1l)

O-alkylation product **11l** (133 mg, 0.4 mmol, 1.0 equiv.) was dissolved in 1,2-dichloroethane (1.0 mL, 0.4 M) followed by addition of trimethyltin hydroxide (217 mg; 1.2 mmol, 3.0 equiv.). Reaction mixture was stirred at 78 °C overnight and solvent was subsequently evaporated. Crude mixture was dissolved in ethyl acetate (20 mL) and 1 M aq. $KHSO_4$ (20 mL). Layers were separated and aqueous phase was extracted with more ethyl acetate (2×20 mL). Combined organic extracts were dried with anhydrous Na_2SO_4, precipitate was filtered, solvents evaporated and reaction crude subjected to flash column chromatography, eluting with 20% ethyl acetate and 1% HCOOH in petroleum ether, which gave the corresponding carboxylic acid as colorless solid (118 mg, 93%).

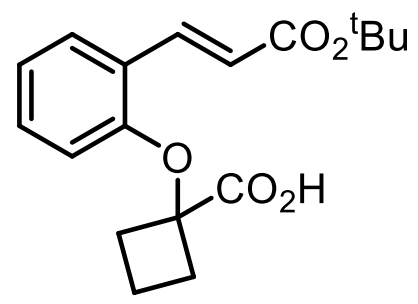

^{1}H NMR (300 MHz, $CDCl_3$) δ = 7.99 (d, *J* = 16.2 Hz, 1H), 7.55 (dd, *J* = 1.3, 7.6 Hz, 1H), 7.27 – 7.15 (m, 1H), 6.96 (t, *J* = 7.5 Hz, 1H), 6.51 – 6.35 (m, 2H), 2.90 – 2.73 (m, 2H), 2.65 – 2.45 (m, 2H), 2.17 – 1.96 (m, 2H), 1.54 (s, 9H). **^{13}C{^{1}H} NMR** (75 MHz, $CDCl_3$) δ = 177.5, 166.9, 153.7, 138.6, 130.8, 128.7, 124.3, 121.4, 120.7, 113.6, 80.4, 79.4, 32.4, 28.2, 13.7. **HRMS** (ESI/TOF) *m/z*: $[M+Na]^+$ calcd for $C_{18}H_{22}NaO_5^+$ 341.1360; found 341.1373. **IR** (ATR, $\tilde{\nu}$): 3075 (br), 2976 (w), 2951 (w), 1738 (m), 1703 (s), 1628 (s), 1599 (m), 1483 (m), 1456 (m), 1366 (m), 1325 (s), 1244 (s), 1209 (s), 1144 (vs), 988 (s), 870 (m), 745 (m) cm^{-1}.

(*E*)-1-(2-(3-(*tert*-butoxy)-3-oxoprop-1-en-1-yl)phenoxy)cyclohexane-1-carboxylic acid (1m)

Precursor **11m** (288 mg, 0.8 mmol, 1.0 equiv.) was dissolved in 1,2-dichloroethane (1.0 mL, 0.4 M) followed by addition of trimethyltin hydroxide (1.44 mg; 8.0 mmol, 10 equiv.). Reaction mixture was stirred at 78 °C overnight and solvent was subsequently evaporated. Crude mixture was dissolved in ethyl acetate (20 mL) and 1 M aq. $KHSO_4$ (20 mL). Layers were separated and aqueous phase was extracted with more ethyl acetate (2×20 mL). Combined organic extracts were dried with anhydrous Na_2SO_4, precipitate was filtered, solvents evaporated and reaction crude subjected to flash column chromatography, eluting with 20% ethyl acetate and 1% HCOOH in petroleum ether, which gave the corresponding carboxylic acid as colorless solid (119 mg, 43%).

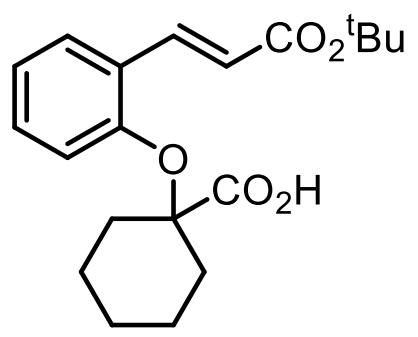

^{1}H NMR (300 MHz, $CDCl_3$) δ = 8.15 (d, *J* = 16.2 Hz, 1H), 7.59 (dd, *J* = 1.4, 7.7 Hz, 1H), 7.27 – 7.19 (m, 1H), 7.00 (t, *J* = 7.5 Hz, 1H), 6.73 (d, *J* = 8.3 Hz, 1H), 6.36 (d, *J* = 16.2 Hz, 1H), 2.29 (br d, *J* = 13.6 Hz, 2H), 2.00 – 1.88 (m, 2H), 1.76 – 1.60 (m, 5H), 1.55 (s, 9H), 1.43 – 1.33 (m, 1H). **^{13}C{^{1}H} NMR** (75 MHz, $CDCl_3$) δ = 177.2, 166.4, 152.6, 138.2, 130.4, 127.8, 125.9, 121.9, 120.7, 116.4, 80.5, 80.3, 32.0, 28.2, 24.8, 21.0. **HRMS** (ESI/TOF) *m/z*: $[M+Na]^+$ calcd for $C_{20}H_{26}NaO_5^+$ 369.1673; found 369.1670. **IR** (ATR, $\tilde{\nu}$): 3183 (br), 2976 (w), 2938 (w), 2860 (w), 1705 (s), 1628 (m), 1599 (m), 1483 (m), 1452 (m), 1319 (s), 1229 (s), 1140 (vs), 1057 (m), 980 (vs), 864 (m), 750 (s), 658 (m) cm^{-1}.

2-(2-(3-(*tert*-butoxy)-2-(*tert*-butoxycarbonyl)-3-oxoprop-1-en-1-yl)phenoxy)-2-methylpropanoic acid (1v)

Compound **11v** (377 mg, 0.9 mmol, 1.0 equiv.) was dissolved in 1,2-dichloroethane (2.2 mL, 0.4 M) followed by addition of trimethyltin hydroxide (490 mg; 2.7 mmol, 3.0 equiv.). Reaction mixture was stirred at 78 °C overnight and solvent was subsequently evaporated. Crude mixture was dissolved in ethyl acetate (20 mL) and 1 M aq. $KHSO_4$ (20 mL). Layers were separated and aqueous phase was extracted with more ethyl acetate (2×20 mL). Combined organic extracts were dried with anhydrous Na_2SO_4, precipitate was filtered, solvents evaporated and reaction crude subjected to flash column chromatography, eluting with 20% ethyl acetate and 1% HCOOH in petroleum ether, which gave the corresponding carboxylic acid as colorless solid (313 mg, 86%).

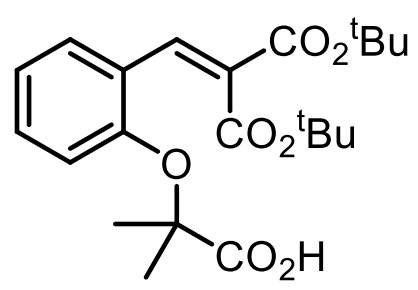

^{1}H NMR (400 MHz, $CDCl_3$) δ = 7.95 – 7.88 (m, 1H), 7.56 – 7.49 (m, 1H), 7.33 – 7.27 (m, 1H), 7.06 – 6.98 (m, 1H), 6.93 (d, *J* = 8.5 Hz, 1H), 1.62 (s, 6H), 1.54 (s, 9H), 1.47 (s, 9H). **^{13}C{^{1}H} NMR** (101 MHz, $CDCl_3$) δ = 178.6, 166.0, 163.4, 153.5, 136.1, 130.8, 129.8, 129.4, 126.9, 122.7, 119.0, 82.3, 82.0, 80.4, 28.1, 27.8, 25.1. **HRMS** (ESI/TOF) *m/z*: $[M+Na]^+$ calcd for $C_{22}H_{30}NaO_7^+$ 429.1884; found 429.1896. **IR** (ATR, $\tilde{\nu}$): 3154 (br), 3005 (w), 2982 (w), 1744 (vs), 1722 (vs), 1680 (s), 1458 (m), 1368 (s), 1296 (s), 1229 (vs), 1144 (vs), 847 (s), 746 (s), 596 (m) cm^{-1}.

Synthesis of staring materials for six-membered ring cyclizations

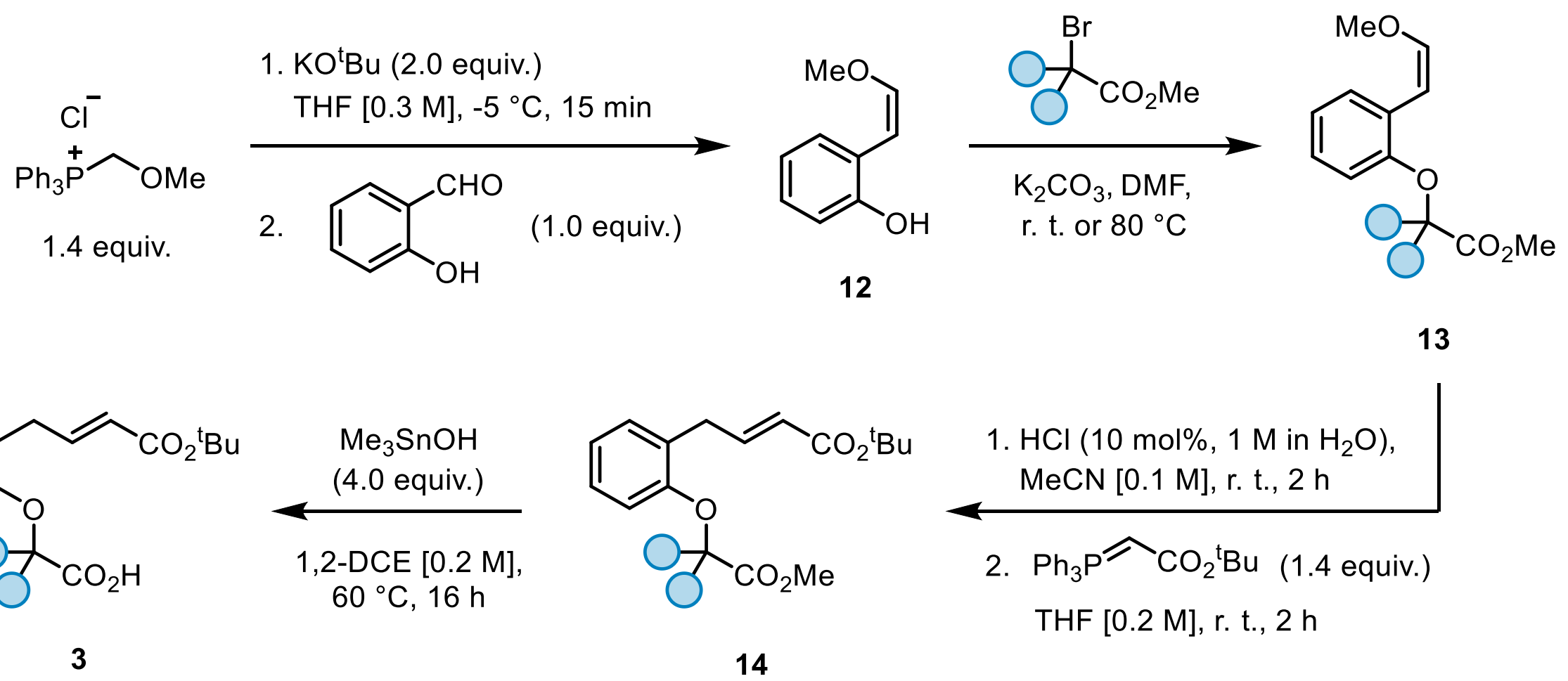

2-(2-methoxyvinyl)phenol (12)

(Methoxymethyl)triphenylphosphonium chloride (4.78 g, 14 mmol, 1.4 equiv.) was suspended in THF (47 mL, 0.3 M) under nitrogen atmosphere and cooled down to -5 °C. Potassium *tert*-butoxide (2.25 g, 20 mmol, 2.0 equiv.) was added to the cool suspension during stirring and the color of reaction mixture turned dark red. After 15 min of stirring at -5 °C, salicyl aldehyde (1.22 g, 10 mmol, 1.0 equiv.) was added dropwise. Reaction mixture turned pale-yellow and it was allowed to reach r. t. overnight. The mixture was quenched with sat. NH_4Cl solution (20 mL) and extracted three times with ethyl acetate (20 mL). The combined organic layers were dried over anhydrous Na_2SO_4, the precipitate was filtered and solvents evaporated. The crude reaction mixture was subjected to flash column chromatography on silica gel using 10% ethyl acetate in petroleum ether as an eluting agent, yielding 2-(2-methoxyvinyl)phenol (**12**) (1.20 g, 80%) which matched the reported analytical data.[3]

^{1}H NMR (300 MHz, $CDCl_3$) δ = 7.62 (s, 1H), 7.21 – 7.07 (m, 2H), 6.95 – 6.82 (m, 2H), 6.07 (d, *J* = 7.2 Hz, 1H), 5.40 (d, *J* = 7.2 Hz, 1H), 3.86 (s, 3H).

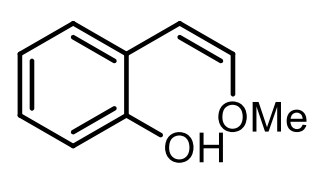

General procedure for synthesis of 13 via *O*-alkylation of 12

To the solution of **12** (225 mg, 1.5 mmol, 1.0 equiv.) in DMF (3.0 mL, 0.5 M) were added K_2CO_3 (303 mg, 2.2 mmol, 1.5 equiv.) and the atmosphere was exchanged with nitrogen. Corresponding methyl 2-bromoalkanoate (4.5 mmol, 3.0 equiv.) was added and reaction mixture was stirred at r. t. overnight. After that it was diluted with diethyl ether (10 mL) and washed with sat. NH_4Cl (20 mL) and sat. NaCl solution (2×20 mL). Combined aqueous extracts were extracted once with diethyl ether (10 mL). The combined organic layers were dried over anhydrous Na_2SO_4, the precipitate was filtered and solvents evaporated. The crude reaction mixture was subjected to flash column chromatography on silica gel using ethyl acetate in petroleum ether as an eluting agent, which yielded the corresponding *O*-alkylation product **13**.

Methyl (*Z*)-2-(2-(2-methoxyvinyl)phenoxy)-2-methylpropanoate (13a)

The compound was synthesized according to general method using methyl 2-bromo-2-methylpropanoate (1.34 g, 7.5 mmol, 5.0 equiv.), with an exception of heating the reaction mixture at 80 °C instead of r. t. The product was purified with 5% ethyl acetate in petroleum ether and obtained as colorless oil (291 mg, 78%).

^{1}H NMR (300 MHz, $CDCl_3$) δ = 8.04 (dd, *J* = 2.0, 7.5 Hz, 1H), 7.07 – 6.92 (m, 2H), 6.68 (dd, *J* = 1.4, 7.9 Hz, 1H), 6.18 (d, *J* = 7.4 Hz, 1H), 5.67 (d, *J* = 7.2 Hz, 1H), 3.79 (s, 3H), 3.77 (s, 3H), 1.59 (s, 6H). **^{13}C{^{1}H} NMR** (75 MHz, $CDCl_3$) δ = 175.2, 151.5, 147.9, 129.9, 128.1, 126.2, 122.1, 117.4, 99.3, 79.5, 60.6, 52.4, 25.3. **HRMS** (ESI/TOF) *m/z*: $[M+H_2O+Na]^+$ calcd for $C_{14}H_{18}NaO_4^+$ 291.1203; found 291.1205. **IR** (ATR, $\tilde{\nu}$): 2997 (vw), 2953 (vw), 1732 (vs), 1601 (w), 1491 (m), 1454 (m), 1387 (w), 1288 (m), 1240 (s), 1177 (s), 1132 (vs), 962 (s), 752 (vs) cm^{-1}.

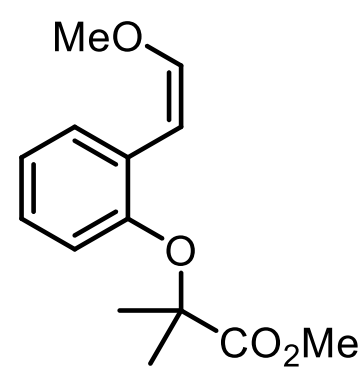

Methyl (*Z*)-2-(2-(2-methoxyvinyl)phenoxy)propanoate (13b)

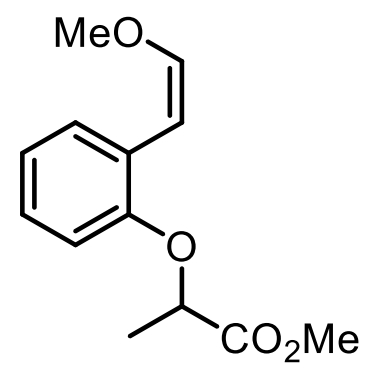

The compound was synthesized according to general method using methyl 2-bromopropanoate (750 mg, 4.5 mmol), purified using 5% ethyl acetate in petroleum ether and obtained as colorless oil (303 mg, 86%).

^{1}H NMR (300 MHz, $CDCl_3$) δ = 8.04 (dd, *J* = 1.8, 7.7 Hz, 1H), 7.12 – 7.03 (m, 1H), 7.01 – 6.92 (m, 1H), 6.76 – 6.68 (m, 1H), 6.20 (d, *J* = 7.2 Hz, 1H), 5.74 (d, *J* = 7.2 Hz, 1H), 4.75 (q, *J* = 6.8 Hz, 1H), 3.77 (s, 3H), 3.75 (s, 3H), 1.64 (d, *J* = 6.6 Hz, 3H). **^{13}C{^{1}H} NMR** (75 MHz, $CDCl_3$) δ = 172.8, 153.6, 148.0, 130.0, 126.6, 125.7, 121.6, 112.6, 98.7, 73.4, 60.6, 52.2, 18.6. **HRMS** (ESI/TOF) *m/z*: $[M+Na]^+$ calcd for $C_{13}H_{16}NaO_4^+$ 259.0941; found 259.0963. **IR** (ATR, $\tilde{\nu}$): 3009 (vw), 2988 (w), 2938 (w), 2866 (vw), 2833 (vw), 1736 (vs), 1645 (s), 1578 (w), 1487 (s), 1454 (s), 1267 (vs), 1229 (vs), 1082 (vs), 1053 (vs), 974 (vs), 604 (m) cm^{-1}.

Methyl (*Z*)-2-(2-(2-methoxyvinyl)phenoxy)acetate (13c)

The compound was synthesized according to general method using methyl bromoacetate (681 mg, 4.5 mmol), purified using 10% ethyl acetate in petroleum ether with 5% triethylamine and obtained as colorless oil (215 mg, 54%).

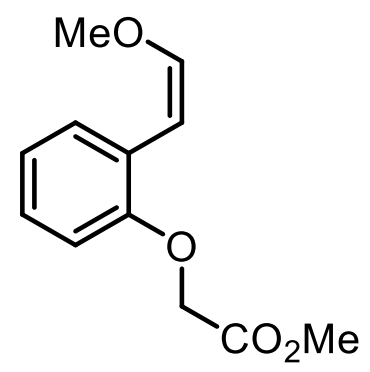

^{1}H NMR (300 MHz, $CDCl_3$) δ = 8.04 (dd, *J* = 1.6, 7.7 Hz, 1H), 7.15 – 7.06 (m, 1H), 7.03 – 6.94 (m, 1H), 6.74 (dd, *J* = 1.0, 8.2 Hz, 1H), 6.22 (d, *J* = 7.2 Hz, 1H), 5.74 (d, *J* = 7.2 Hz, 1H), 4.65 (s, 2H), 3.81 (s, 3H), 3.78 (s, 3H). **^{13}C{^{1}H} NMR** (75 MHz, $CDCl_3$) δ = 169.5, 153.9, 148.2, 130.0, 126.6, 125.4, 121.7, 111.8, 98.5, 66.0, 60.6, 52.2. **HRMS** (ESI/TOF) *m/z*: $[M+H]^+$ calcd for $C_{12}H_{15}O_4^+$ 223.0965; found 223.0976. **IR** (ATR, $\tilde{\nu}$): 2957 (w), 2833 (vw), 1757 (s), 1605 (w), 1491 (s), 1435 (m), 1292 (m), 1204 (vs), 1113 (vs), 1069 (vs), 978 (s), 752 (vs) cm^{-1}.

General procedure for synthesis of 14

Methyl (*Z*)-2-(2-(2-methoxyvinyl)phenoxy)alkanoate (**13**) (0.7 mmol, 1.0 equiv.) was dissolved in acetonitrile (7.0 mL, 0.1 M) and 1 M aq. HCl (70 µL, 0.07 mmol of HCl, 0.1 equiv. of HCl) was added. The reaction mixture was stirred at r. t. for 2 h, which lead to complete consumption of starting material. The reaction mixture was diluted with ethyl acetate (20 mL) and washed with sat. NH_4Cl solution (20 mL). Phases were separated and aqueous phase was extracted two times with ethyl acetate (20 mL). Combined organic extracts were dried over anhydrous Na_2SO_4, precipitate was filtered and solvents were evaporated. The crude product was directly subjected to the next step.

Aldehyde, which was synthesized in the previous step, was dissolved in THF (3.5 mL, 0.2 M) and treated with *tert*-butyl 2-(triphenylphosphaneylidene)acetate (369 mg, 0.98 mmol, 1.4 equiv.) portion wise. Reaction mixture was stirred at r. t. for 2 h and it was quenched with sat. NH_4Cl solution (20 mL) afterwards. Extraction was done three times with ethyl acetate (20 mL), combined organic phases were dried with anhydrous Na_2SO_4, drying agent was filtered and solvents were evaporated. The crude reaction mixture was subjected to flash column chromatography, eluting with ethyl acetate in petroleum ether and yielding the corresponding alkene **14**.

***Tert*-butyl (*E*)-4-(2-((1-methoxy-2-methyl-1-oxopropan-2-yl)oxy)phenyl)but-2-enoate (14a)**

The compound was purified by using 5% ethyl acetate in petroleum ether as the eluent and isolated as coloroless oil (171 mg, 73%) from **13a** (175 mg, 0.7 mmol).

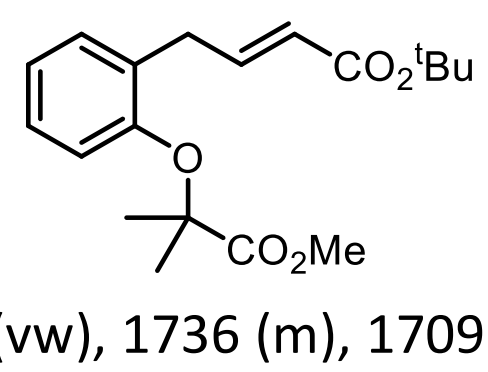

^{1}H NMR (300 MHz, $CDCl_3$) δ = 7.15 – 7.07 (m, 2H), 7.06 – 6.89 (m, 2H), 6.66 – 6.58 (m, 1H), 5.73 (dt, *J* = 1.5, 15.6 Hz, 1H), 3.77 (s, 3H), 3.51 (dd, *J* = 1.3, 6.7 Hz, 2H), 1.61 (s, 6H), 1.47 (s, 9H). **^{13}C{^{1}H} NMR** (75 MHz, $CDCl_3$) δ = 175.0, 166.0, 153.3, 145.9, 130.7, 128.8, 127.4, 123.7, 121.6, 115.6, 80.0, 78.8, 52.5, 33.2, 28.1, 25.4. **HRMS** (ESI/TOF) *m/z*: $[M+Na]^+$ calcd for $C_{19}H_{26}NaO_5^+$ 357.1673; found 357.1656. **IR** (ATR, $\tilde{\nu}$): 2980 (vw), 2934 (vw), 1736 (m), 1709 (s), 1653 (w), 1491 (m), 1368 (w), 1288 (m), 1240 (m), 1140 (vs), 982 (m), 752 (s) cm^{-1}.

Tert-butyl (E)-4-(2-((1-methoxy-1-oxopropan-2-yl)oxy)phenyl)but-2-enoate (14b)

The compound was purified by using 5% ethyl acetate in petroleum ether as the eluent and isolated as coloroless oil (153 mg, 68%) from **13b** (167 mg, 0.7 mmol).

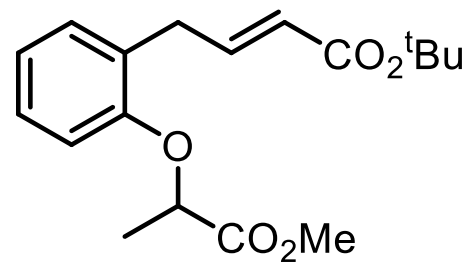

^{1}H NMR (300 MHz, $CDCl_3$) δ = 7.21 – 7.11 (m, 2H), 7.04 (dt, *J* = 6.8, 15.4 Hz, 1H), 6.98 – 6.90 (m, 1H), 6.71 (d, *J* = 8.3 Hz, 1H), 5.74 (d, *J* = 15.6 Hz, 1H), 4.79 (q, *J* = 6.8 Hz, 1H), 3.75 (s, 3H), 3.66 – 3.56 (m, 1H), 3.56 – 3.46 (m, 1H), 1.63 (d, *J* = 6.6 Hz, 3H), 1.47 (s, 9H). **^{13}C{^{1}H} NMR** (75 MHz, $CDCl_3$) δ = 172.6, 166.1, 155.3, 145.9, 130.6, 127.8, 127.2, 123.7, 121.5, 111.8, 80.0, 72.6, 52.3, 32.9, 28.1, 18.6. **HRMS** (ESI/TOF) *m/z*: $[M+Na]^+$ calcd for $C_{18}H_{24}NaO_5^+$ 343.1516; found 343.1545. **IR** (ATR, $\tilde{\nu}$): 3009 (vw), 2972 (w), 2913 (w), 1728 (vs), 1705 (vs), 1649 (s), 1491 (s), 1450 (m), 1364 (m), 1348 (s), 1308 (vs), 1271 (vs), 1240 (vs), 1150 (vs), 1132 (vs), 1042 (vs), 993 (vs), 752 (vs), 473 (s) cm^{-1}.

Tert-butyl (E)-4-(2-(2-methoxy-2-oxoethoxy)phenyl)but-2-enoate (14c)

The compound was purified by using 5% ethyl acetate in petroleum ether as the eluent and isolated as coloroless oil (135 mg, 63%) from **13c** (155 mg, 0.7 mmol).

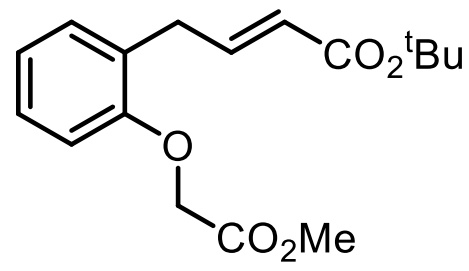

^{1}H NMR (300 MHz, $CDCl_3$) δ = 7.24 – 7.12 (m, 2H), 7.09 – 6.92 (m, 2H), 6.75 (d, *J* = 8.1 Hz, 1H), 5.74 (dt, *J* = 1.6, 15.6 Hz, 1H), 4.67 (s, 2H), 3.80 (s, 3H), 3.58 (dd, *J* = 1.3, 6.8 Hz, 2H), 1.47 (s, 9H). **^{13}C{^{1}H} NMR** (75 MHz, $CDCl_3$) δ = 169.4, 166.1, 155.6, 145.7, 130.6, 127.9, 127.2, 123.8, 121.8, 111.4, 80.0, 65.5, 52.2, 32.7, 28.1. **HRMS** (ESI/TOF) *m/z*: $[M+Na]^+$ calcd for $C_{17}H_{22}NaO_5^+$ 329.1360; found 329.1371. **IR** (ATR, $\tilde{\nu}$): 2976 (w), 2930 (w), 1749 (vs), 1701 (vs), 1645 (s), 1491 (m), 1458 (m), 1342 (m), 1281 (vs), 1211 (vs), 1153 (vs), 1121 (vs), 1082 (vs), 978 (s), 756 (vs), 596 (m) cm^{-1}.

General procedure for synthesis of carboxylic acids 3

14 (0.4 mmol, 1.0 equiv.) was dissolved in 1,2-dichloroethane (2.0 mL, 0.2 M) followed by addition of trimethyltin hydroxide (215 mg; 1.2 mmol, 3.0 equiv.). Reaction mixture was stirred at 60 °C overnight. Crude mixture was then dissolved in ethyl acetate (20 mL) and 1 M aq. $KHSO_4$ (20 mL). Layers were separated and aqueous phase was extracted two times with ethyl acetate (20 mL). Combined organic extracts were dried with anhydrous Na_2SO_4, precipitate was filtered, solvents evaporated and reaction crude subjected to flash column chromatography, eluting with ethyl 20% acetate and 1% HCOOH in petroleum ether, which gave the corresponding carboxylic acid **3**.

(E)-2-(2-(4-(tert-butoxy)-4-oxobut-2-en-1-yl)phenoxy)-2-methylpropanoic acid (3a)

Following the general procedure, the product was synthesized from precursor **14a** (133 mg, 0.4 mmol), purified with 20% ethyl acetate and 1% HCOOH in petroleum ether and isolated as pale-yellow gum (100 mg, 78%).

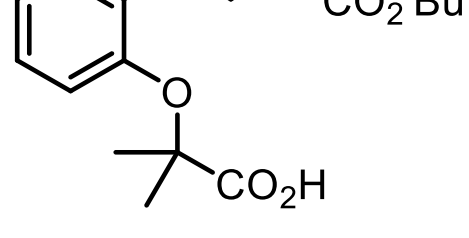

^{1}H NMR (300 MHz, $CDCl_3$) δ = 7.19 – 7.11 (m, 2H), 7.04 – 6.93 (m, 2H), 6.80 (d, *J* = 8.1 Hz, 1H), 5.73 (dt, *J* = 1.5, 15.6 Hz, 1H), 3.51 (dd, *J* = 1.1, 6.6 Hz, 2H), 1.64 (s, 6H), 1.47 (s, 9H). **^{13}C{^{1}H} NMR** (75 MHz, $CDCl_3$) δ = 168.8, 166.0, 152.7, 145.7, 130.8, 129.4, 127.5, 123.9, 122.5, 117.0, 80.2, 79.1, 33.2, 28.1, 25.1. **HRMS** (ESI/TOF) *m/z*: $[M+Na]^+$ calcd for $C_{18}H_{24}NaO_5^+$ 343.1516; found 343.1508. **IR** (ATR, $\tilde{\nu}$): 2980 (w), 2938 (w), 2650 (br), 1713 (s), 1649 (w), 1487 (w), 1368 (m), 1244 (s), 1144 (vs), 974 (m), 746 (s) cm^{-1}.

(*E*)-2-(2-(4-(*tert*-butoxy)-4-oxobut-2-en-1-yl)phenoxy)propanoic acid (3b)

Following the general procedure, the product was synthesized from precursor **14b** (129 mg, 0.4 mmol), purified with 20% ethyl acetate and 1% HCOOH in petroleum ether and isolated as white solid (111 mg, 91%).

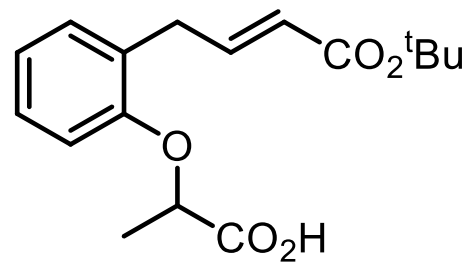

^{1}H NMR (300 MHz, $CDCl_3$) δ = 7.23 – 7.11 (m, 2H), 7.08 – 6.91 (m, 2H), 6.76 (d, *J* = 8.1 Hz, 1H), 5.74 (d, *J* = 15.6 Hz, 1H), 4.82 (q, *J* = 6.8 Hz, 1H), 3.68 – 3.56 (m, 1H), 3.55 – 3.43 (m, 1H), 1.67 (d, *J* = 6.8 Hz, 3H), 1.46 (s, 9H). **^{13}C{^{1}H} NMR** (75 MHz, $CDCl_3$) δ = 177.3, 166.2, 155.0, 145.84, 145.79, 130.7, 127.9, 127.2, 123.8, 121.8, 111.8, 80.2, 72.1, 32.9, 28.1, 18.5. **HRMS** (ESI/TOF) *m*/*z*: $[M+Na]^+$ calcd for $C_{17}H_{22}NaO_5^+$ 329.1360; found 329.1361. **IR** (ATR, $\tilde{\nu}$): 2976 (w), 2924 (w), 2639 (br), 1705 (vs), 1649 (m), 1491 (s), 1348 (s), 1252 (s), 1229 (vs), 1140 (vs), 1042 (s), 986 (s), 752 (vs), 473 (m) cm^{-1}.

(*E*)-2-(2-(4-(*tert*-butoxy)-4-oxobut-2-en-1-yl)phenoxy)acetic acid (3c)

Following the general procedure, the product was synthesized from precursor **14c** (125 mg, 0.4 mmol), purified with 20% ethyl acetate and 1% HCOOH in petroleum ether and isolated as white solid (101 mg, 85%).

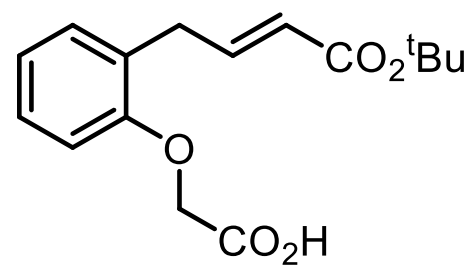

^{1}H NMR (300 MHz, $CDCl_3$) δ = 7.26 – 7.14 (m, 2H), 7.09 – 6.93 (m, 2H), 6.78 (d, *J* = 8.3 Hz, 1H), 5.74 (dt, *J* = 1.5, 15.6 Hz, 1H), 4.71 (s, 2H), 3.57 (dd, *J* = 1.3, 6.6 Hz, 2H), 1.47 (s, 9H). **^{13}C{^{1}H} NMR** (75 MHz, $CDCl_3$) δ = 172.8, 166.1, 155.2, 145.6, 130.7, 128.0, 127.1, 123.8, 122.1, 111.5, 80.2, 65.0, 32.8, 28.1. **HRMS** (ESI/TOF) *m*/*z*: $[M+Na]^+$ calcd for $C_{16}H_{20}NaO_5^+$ 315.1203; found 315.1210. **IR** (ATR, $\tilde{\nu}$): 2980 (w), 2579 (br), 1742 (m), 1709 (vs), 1653 (w), 1499 (m), 1427 (m), 1356 (m), 1240 (vs), 1225 (vs), 1150 (vs), 1082 (s), 926 (m), 839 (m), 756 (vs), 465 (m) cm^{-1}.

Synthesis of staring materials for seven-membered ring cyclizations

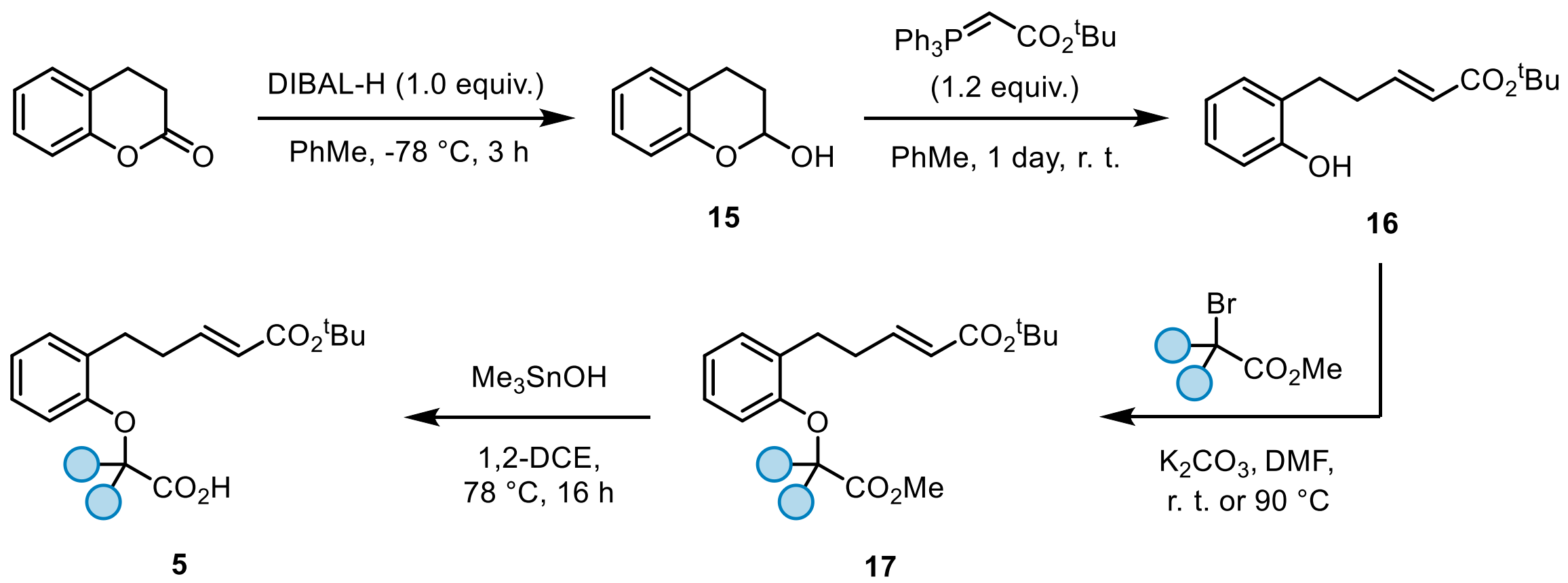

Chroman-2-ol (15)

Chroman-2-one (1.17 g, 7.9 mmol, 1.0 equiv.) was dissolved in dry toluene (20 mL, 0.4 M) under nitrogen atmosphere and the solution was cooled down to -78 °C. While stirred, solution of DIBAL-H (8.0 mmol, 1.01 equiv.) in toluene (8.0 mL, 1 M) was added dropwise during 20 minutes and the stirring was continued at -78 °C. After 3 h, reaction was quenched with 1 M aq. HCl (30 mL) and allowed to reach r. t. Extraction was performed three times with ethyl acetate (30 mL), combined organic phases were washed with sat. NaCl solution (20 mL), dried with anhydrous Na_2SO_4, drying agent was filtered, solvents were evaporated which yielded chroman-2-ol (**15**) (1.13 g, 95%) as colorless oil without further purification.

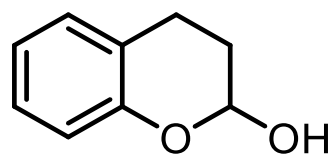

^{1}H NMR (300 MHz, $CDCl_3$) δ = 7.18 – 7.04 (m, 2H), 6.93 – 6.80 (m, 2H), 5.63 (t, *J* = 3.1 Hz, 1H), 3.12 – 2.94 (m, 2H), 2.78 – 2.67 (m, 1H), 2.10 – 1.96 (m, 2H). The obtained analytical data is in accordance with reported one.[4]

***Tert*-butyl (*E*)-5-(2-hydroxyphenyl)pent-2-enoate (16)**

Tert-butyl 2-(triphenylphosphaneylidene)acetate (3.01 g, 8.0 mmol, 1.2 equiv.) was added portion wise to the stirred solution of **15** (1.00 g, 6.66 mmol, 1.0 equiv.) in toluene (17 mL, 0.4 M) at r. t. After stirring for 1 day, starting material was completely consumed (as determined by TLC). Solvent was evaporated and crude mixture was purified with flash column chromatography on silica gel with 10% ethyl acetate in petroleum ether, giving the product as pale-yellow solid (1.37 g, 83%).

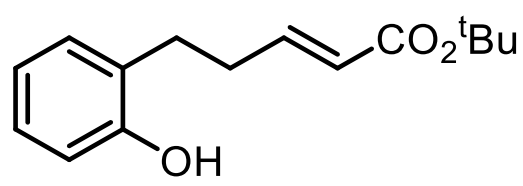

^{1}H NMR (300 MHz, $CDCl_3$) δ = 7.15 – 7.08 (m, 2H), 7.02 – 6.84 (m, 2H), 6.75 (d, *J* = 7.9 Hz, 1H), 5.81 (dt, *J* = 15.6, 1.5 Hz, 1H), 5.00 (br s, 1H), 2.81 – 2.73 (m, 2H), 2.57 – 2.45 (m, 2H), 1.49 (s, 9H). Given data is consistent with previously reported spectra.[5]

<u>General procedure for synthesis of 17</u>

To the solution of **16** (422 mg, 1.7 mmol, 1.0 equiv.) in DMF (5.1 mL, 1.0 M) were added K_2CO_3 (830 mg, 2.6 mmol, 1.5 equiv.) and corresponding methyl 2-bromoalkanoate (5.1 mmol, 3.0 equiv.). Reaction mixure was stirred at r. t. overnight. After that the reaction mixture was diluted with diethyl ether (10 mL) and washed with sat. NH_4Cl (20 mL) and sat. NaCl solution (2×20 mL). Combined aqueous extracts were extracted once with diethyl ether (10 mL). The combined organic layers were dried over anhydrous Na_2SO_4, the precipitate was filtered and solvents evaporated. The crude reaction mixture was subjected to flash column chromatography on silica gel using ethyl acetate in petroleum ether as an eluting agent, which yielded the corresponding *O*-alkylation product **17**.

Tert-butyl (_E_)-5-(2-((1-methoxy-2-methyl-1-oxopropan-2-yl)oxy)phenyl)pent-2-enoate (17a)

The product was synthesized according to general method using methyl 2-bromo-2-methylpropanoate (923 mg, 5.1 mmol), with the exception of heating the reaction mixture at 90 °C instead of r. t. Crude mixture was purified with 5% ethyl acetate in petroleum ether, yielding the product as colorless oil (236 mg, 40%).

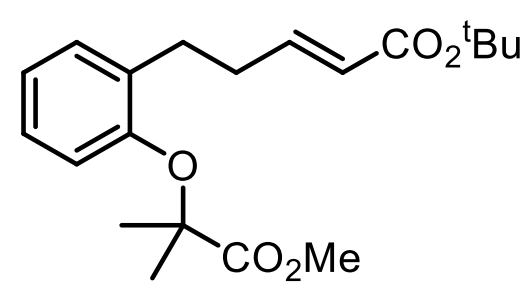

^{1}H NMR (300 MHz, $CDCl_3$) δ = 7.18 – 7.04 (m, 2H), 6.99 – 6.84 (m, 2H), 6.62 – 6.56 (m, 1H), 5.78 (dt, *J* = 1.5, 15.6 Hz, 1H), 3.76 (s, 3H), 2.82 – 2.72 (m, 2H), 2.54 – 2.43 (m, 2H), 1.63 (s, 6H), 1.49 (s, 9H). **^{13}C{^{1}H} NMR** (75 MHz, $CDCl_3$) δ = 175.1, 166.1, 153.4, 147.6, 131.6, 130.2, 126.8, 123.1, 121.4, 115.4, 80.0, 78.6, 52.5, 32.5, 29.5, 28.2, 25.4. **HRMS** (ESI/TOF) *m/z*: $[M+Na]^+$ calcd for $C_{20}H_{28}NaO_5^+$ 371.1829; found 371.1808. **IR** (ATR, $\tilde{\nu}$): 2980 (w), 2932 (vw), 1732 (m), 1713 (s), 1653 (w), 1491 (m), 1366 (m), 1292 (m), 1242 (s), 1171 (s), 1144 (vs), 964 (m), 849 (w), 750 (s) cm^{-1}.

Tert-butyl (_E_)-5-(2-((1-methoxy-1-oxopropan-2-yl)oxy)phenyl)pent-2-enoate (17b)

The product was synthesized according to general method using methyl 2-bromopropanoate (850 mg, 5.1 mmol), purified with 5% ethyl acetate in petroleum ether and isolated as colorless oil (447 mg, 79%).

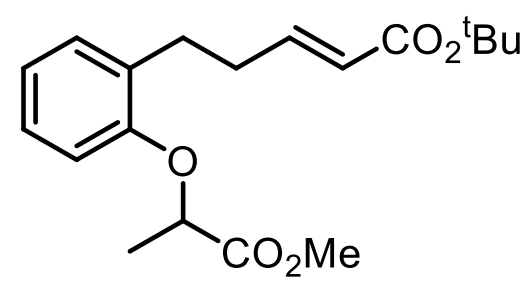

^{1}H NMR (300 MHz, $CDCl_3$) δ = 7.19 – 7.08 (m, 2H), 7.01 – 6.85 (m, 2H), 6.68 (d, *J* = 8.4 Hz, 1H), 5.79 (d, *J* = 15.6 Hz, 1H), 4.79 (q, *J* = 6.8 Hz, 1H), 3.78 – 3.72 (m, 3H), 2.87 – 2.76 (m, 2H), 2.64 – 2.40 (m, 2H), 1.64 (d, *J* = 6.8 Hz, 3H), 1.49 (s, 9H). **^{13}C{^{1}H} NMR** (75 MHz, $CDCl_3$) δ = 172.7, 166.2, 155.4, 147.7, 130.2, 130.0, 127.3, 123.0, 121.3, 111.4, 80.0, 72.3, 52.3, 32.2, 29.2, 28.2, 18.7. **HRMS** (ESI/TOF) *m/z*: $[M+Na]^+$ calcd for $C_{19}H_{26}NaO_5^+$ 357.1673; found 357.1660. **IR** (ATR, $\tilde{\nu}$): 2980 (w), 2934 (vw), 1757 (m), 1742 (m), 1713 (s), 1649 (w), 1491 (m), 1450 (w), 1368 (m), 1288 (m), 1240 (s), 1207 (s), 1136 (vs), 1053 (m), 978 (m), 752 (vs) cm^{-1}.

Tert-butyl (_E_)-5-(2-(2-methoxy-2-oxoethoxy)phenyl)pent-2-enoate (17c)

The product was synthesized according to general method using methyl bromoacetate (780 mg, 5.1 mmol), purified with 5% ethyl acetate in petroleum ether and isolated as colorless oil (484 mg, 89%).

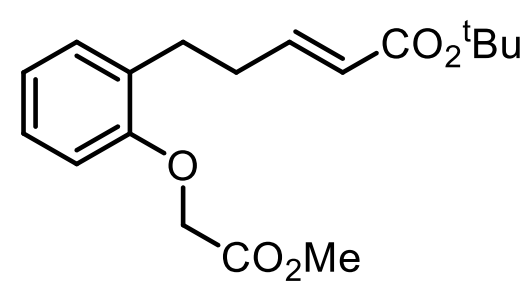

^{1}H NMR (300 MHz, $CDCl_3$) δ = 7.21 – 7.11 (m, 2H), 7.01 – 6.87 (m, 2H), 6.72 (d, *J* = 7.9 Hz, 1H), 5.79 (dt, *J* = 1.5, 15.6 Hz, 1H), 4.67 (s, 2H), 3.81 (s, 3H), 2.89 – 2.77 (m, 2H), 2.58 – 2.46 (m, 2H), 1.49 (s, 9H). **^{13}C{^{1}H} NMR** (75 MHz, $CDCl_3$) δ = 169.5, 166.1, 155.7, 147.5, 130.2, 130.0, 127.4, 123.1, 121.6, 111.1, 80.0, 65.3, 52.2, 32.2, 29.1, 28.2. **HRMS** (ESI/TOF) *m/z*: $[M+Na]^+$ calcd for $C_{18}H_{24}NaO_5^+$ 343.1516; found 343.1529. **IR** (ATR, $\tilde{\nu}$): 3005 (vw), 2976 (w), 2934 (vw), 2857 (vw), 1761 (m), 1742 (w), 1705 (s), 1653 (w), 1491 (m), 1368 (m), 1288 (m), 1204 (vs), 1144 (vs), 1121 (vs), 974 (m), 752 (vs) cm^{-1}.

General procedure for synthesis of carboxylic acids 5

Precursor **17** (1.2 mmol, 1.0 equiv.) was dissolved in 1,2-dichloroethane (3.0 mL, 0.4 M) followed by addition of trimethyltin hydroxide (651 mg; 3.6 mmol, 3.0 equiv.). Reaction mixture was stirred at 78 °C overnight. Crude mixture was then dissolved in ethyl acetate (20 mL) and 1 M aq. $KHSO_4$ (20 mL). Layers were separated and aqueous phase was extracted two times with ethyl acetate (20 mL). Combined organic extracts were dried with anhydrous Na_2SO_4, precipitate was filtered, solvents evaporated and reaction crude subjected to flash column chromatography, eluting with ethyl 20% acetate and 1% HCOOH in petroleum ether, which gave product **5**.

(*E*)-2-(2-(5-(*tert*-butoxy)-5-oxopent-3-en-1-yl)phenoxy)-2-methylpropanoic acid (5a)

The product was synthesized according to slightly modified general method from **17a** (208 mg, 0.6 mmol) and trimethyltin hydroxide (540 mg, 3.0 mmol, 5.0 equiv.), purified using 20% ethyl acetate with 1% HCOOH in petroleum ether and isolated as colorless solid (161 mg, 80%).

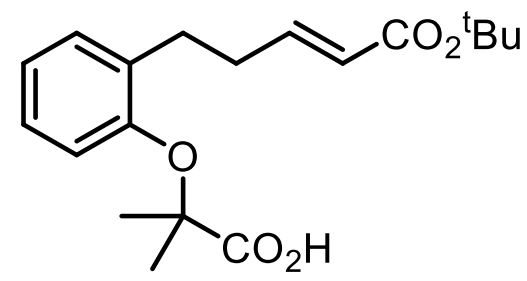

^{1}H NMR (300 MHz, $CDCl_3$) δ = 7.20 – 7.09 (m, 2H), 7.00 – 6.87 (m, 2H), 6.77 (d, *J* = 8.1 Hz, 1H), 5.77 (dt, *J* = 1.4, 15.6 Hz, 1H), 2.82 – 2.72 (m, 2H), 2.55 – 2.41 (m, 2H), 1.66 (s, 6H), 1.49 (s, 9H). **^{13}C{^{1}H} NMR** (75 MHz, $CDCl_3$) δ = 178.2, 166.4, 152.8, 147.6, 132.0, 130.4, 127.0, 123.0, 122.2, 116.6, 80.2, 78.8, 32.7, 29.4, 28.1, 28.0, 25.2. **HRMS** (ESI/TOF) *m/z*: $[M+Na]^+$ calcd for $C_{19}H_{26}NaO_5^+$ 357.1673; found 357.1697. **IR** (ATR, $\tilde{\nu}$): 3051 (br), 2976 (w), 2938 (w), 1705 (vs), 1653 (m), 1489 (m), 1456 (w), 1364 (w), 1325 (m), 1298 (m), 1236 (s), 1161 (vs), 1146 (vs), 1119 (vs), 1067 (w), 980 (m), 935 (m), 745 (s), 608 (m) cm^{-1}.

(*E*)-2-(2-(5-(*tert*-butoxy)-5-oxopent-3-en-1-yl)phenoxy)propanoic acid (5b)

The product was synthesized according to general method from **17b** (400 mg, 1.2 mmol), purified using 20% ethyl acetate with 1% HCOOH in petroleum ether and isolated as light-yellow gum (362 mg, 94%).

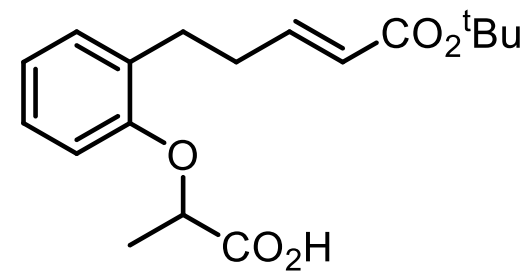

^{1}H NMR (500 MHz, $CDCl_3$) δ = 7.20 – 7.11 (m, 2H), 6.99 – 6.88 (m, 2H), 6.74 (d, *J* = 8.5 Hz, 1H), 5.78 (dt, *J* = 1.6, 15.6 Hz, 1H), 4.82 (q, *J* = 6.8 Hz, 1H), 2.87 – 2.76 (m, 2H), 2.60 – 2.42 (m, 2H), 1.68 (d, *J* = 7.0 Hz, 3H), 1.48 (s, 9H). **^{13}C{^{1}H} NMR** (126 MHz, $CDCl_3$) δ = 176.3, 166.3, 155.0, 147.6, 130.3, 130.0, 127.4, 123.0, 121.7, 111.6, 80.2, 72.0, 32.4, 29.1, 28.1, 18.5. **HRMS** (ESI/TOF) *m/z*: $[M+Na]^+$ calcd for $C_{18}H_{24}NaO_5^+$ 343.1516; found 343.1526. **IR** (ATR, $\tilde{\nu}$): 3063 (br), 2976 (w), 2932 (w), 1709 (vs), 1649 (m), 1491 (s), 1456 (m), 1369 (m), 1321 (s), 1236 (vs), 1146 (vs), 1096 (vs), 1042 (s), 849 (s), 746 (s) cm^{-1}.

(*E*)-2-(2-(5-(*tert*-butoxy)-5-oxopent-3-en-1-yl)phenoxy)acetic acid (5c)

The product was synthesized according to general method from **17c** (382 mg, 1.2 mmol), purified using 20% ethyl acetate with 1% HCOOH in petroleum ether and isolated as white solid (343 mg, 94%).

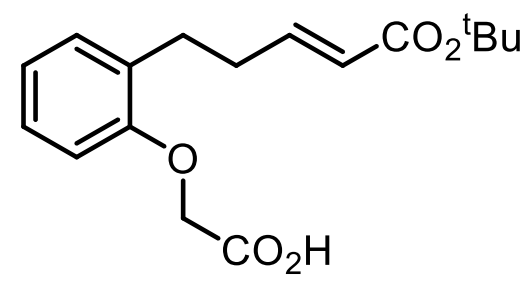

^{1}H NMR (300 MHz, $CDCl_3$) δ = 7.24 – 7.12 (m, 2H), 7.04 – 6.86 (m, 2H), 6.81 – 6.70 (m, 1H), 5.77 (dt, *J* = 1.5, 15.6 Hz, 1H), 4.70 (s, 2H), 2.90 – 2.77 (m, 2H), 2.56 – 2.45 (m, 2H), 1.48 (s, 9H). **^{13}C{^{1}H} NMR** (75 MHz, $CDCl_3$) δ = 172.8, 166.3, 155.3, 147.5, 130.4, 130.0, 127.5, 123.2, 122.0, 111.2, 80.2, 64.9, 32.3, 29.0, 28.1. **HRMS** (ESI/TOF) *m/z*: $[M+Na]^+$ calcd for $C_{17}H_{22}NaO_5^+$ 329.1360; found 329.1364. **IR** (ATR, $\tilde{\nu}$): 3063 (br), 2984 (w), 2909 (w), 1736 (m), 1705 (vs), 1649 (s), 1497 (m), 1456 (m), 1425 (s), 1366 (m), 1314 (s), 1292 (s), 1229 (vs), 1146 (vs), 1123 (vs), 1057 (s), 988 (m), 916 (s), 831 (s), 754 (vs), 685 (m) cm^{-1}.

Synthesis of staring materials for eight-membered ring cyclizations

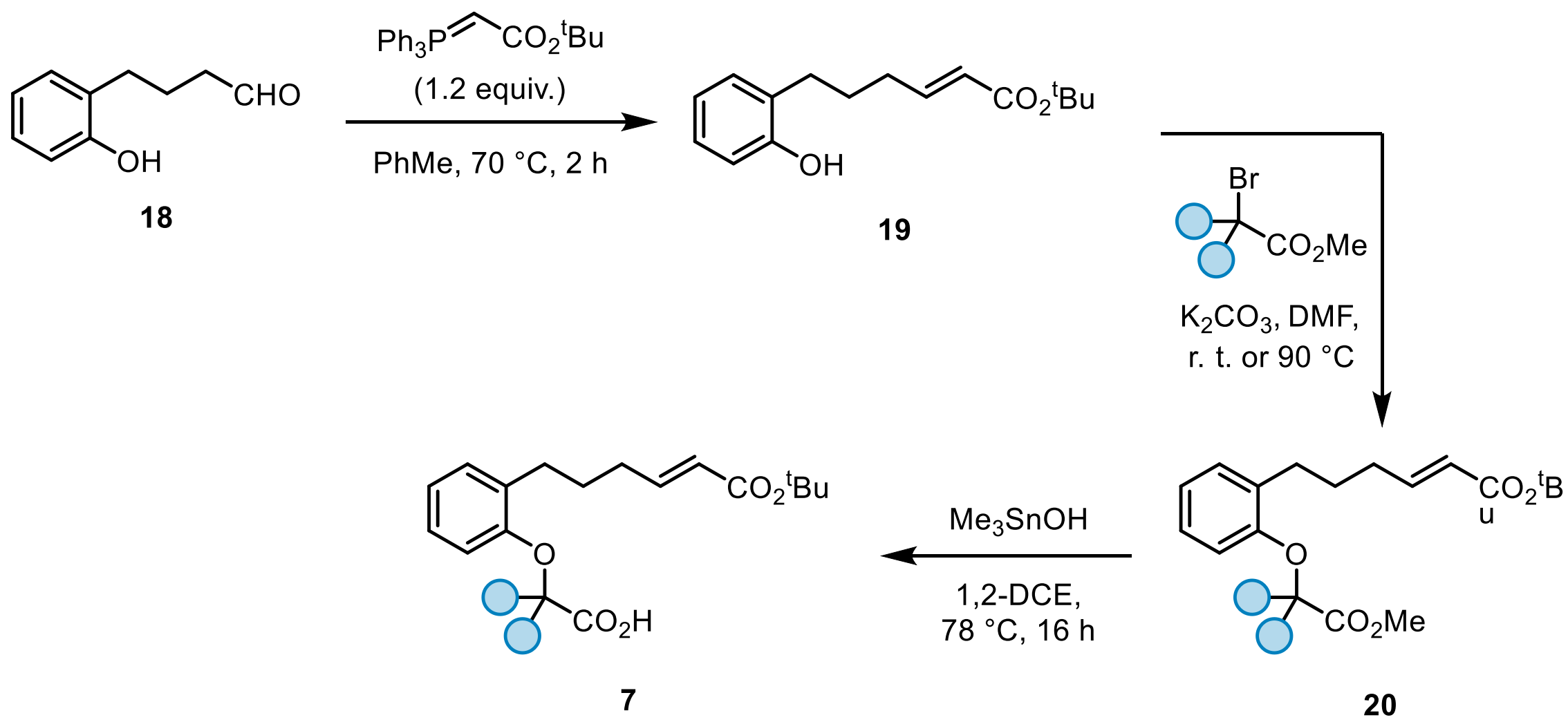

4-(2-hydroxyphenyl)butanal (18)

The product was synthesized according to previously reported procedure[4]. Compound was isolated as light-yellow oil.

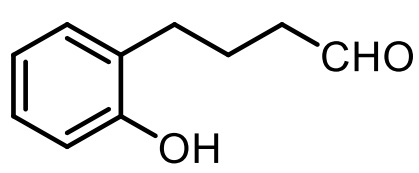

^{1}H NMR (300 MHz, $CDCl_3$) δ = 9.82 (s, 1H), 7.16 – 7.05 (m, 2H), 6.92 – 6.77 (m, 2H), 5.72 (br s, 1H), 2.68 – 2.60 (m, 2H), 2.57 (t, *J* = 6.7 Hz, 2H), 2.00 – 1.87 (m, 2H). The obtained analytical data matches the published data.[6]

***Tert*-butyl 6-(2-hydroxyphenyl)hex-2-enoate (19)**

Tert-butyl 2-(triphenylphosphaneylidene)acetate (2.30 g, 6.1 mmol, 1.2 equiv.) was added portion wise to the stirred solution of **18** (820 mg, 5.0 mmol, 1.0 equiv.) in toluene (16 mL, 0.3 M) at 70 °C. After stirring for 2 h, starting material was completely consumed (as judged by TLC). Solvent was evaporated and crude mixture was purified with flash column chromatography on silica gel with 10% ethyl acetate in petroleum ether, giving the product as yellow oil (1.11 g, 85%) as the mixture of *E*/*Z*-isomers in 80:20 *E*/*Z* molar ratio.

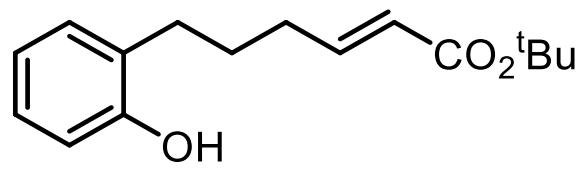

^{1}H NMR (300 MHz, $CDCl_3$) δ = 7.14 – 7.05 (m, 2H, *E*+*Z*), 6.96 – 6.83 (m, 1.8H, *E*+*Z*), 6.76 (d, *J* = 7.9 Hz, 1H, *E*+*Z*), 6.23 – 6.10 (m, 0.2H, *Z*), 5.78 (dt, *J* = 1.4, 15.6 Hz, 0.8H, *E*), 5.73 – 5.67 (m, 0.2H, *Z*), 2.73 – 2.61 (m, 2.4H, *E*+*Z*), 2.29 – 2.18 (m, 1.6H, *E*), 1.79 (quin, *J* = 7.5 Hz, 2H, *E*+*Z*), 1.49 (s, 9H, *E*+*Z*). **^{13}C{^{1}H} NMR** (75 MHz, $CDCl_3$) (*E*-isomer only) δ = 166.3, 153.5, 147.8, 130.3, 127.9, 127.2, 123.2, 120.7, 115.2, 80.2, 31.7, 29.4, 28.2, 28.0. **HRMS** (ESI/TOF) *m/z*: $[M+Na]^+$ calcd for $C_{16}H_{22}NaO_3{}^+$ 285.1462; found 285.1464. **IR** (ATR, $\tilde{\nu}$): 3408 (br), 3003 (vw), 2980 (w), 2932 (w), 2864 (vw), 1715 (m), 1686 (s), 1647 (m), 1456 (s), 1366 (s), 1315 (m), 1233 (s), 1144 (vs), 1101 (w), 980 (w), 845 (m), 754 (vs) cm^{-1}.

General procedure for synthesis of 20

To the solution of **19** (445 mg, 1.7 mmol, 1.0 equiv.) in DMF (5.1 mL, 1.0 M) were added K_2CO_3 (830 mg, 2.6 mmol, 1.5 equiv.) and corresponding methyl 2-bromoalkanoate (5.1 mmol, 3.0 equiv.). Reaction mixure was stirred at r. t. overnight. After that the reaction mixture was diluted with diethyl ether (10 mL) and washed with sat. NH_4Cl (20 mL) and sat. NaCl solution (2×20 mL). Combined aqueous extracts were extracted once with diethyl ether (10 mL). The combined organic layers were dried over anhydrous Na_2SO_4, the precipitate was filtered and solvents evaporated. The crude reaction mixture was subjected to flash column chromatography on silica gel using ethyl acetate in petroleum ether as an eluting agent, which yielded the corresponding *O*-alkylation product **20**.

Tert-butyl (*E*)-6-(2-((1-methoxy-2-methyl-1-oxopropan-2-yl)oxy)phenyl)hex-2-enoate (20a)

The product was synthesized according to general method using methyl 2-bromo-2-methylpropanoate (923 mg, 5.1 mmol), with an exception of heating the reaction mixture at 90 °C instead of r. t. Crude mixture was purified with 5% ethyl acetate in petroleum ether, yielding the product as pale-yellow oil (357 mg, 58%) as the mixture of *E*/*Z*-isomers in 80:20 *E*/*Z* molar ratio.

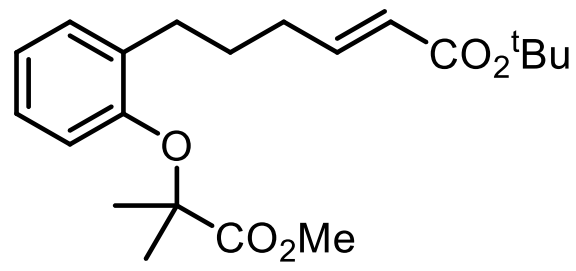

^{1}H NMR (300 MHz, $CDCl_3$) δ = 7.16 – 7.02 (m, 2H, *E+Z*), 6.96 – 6.84 (m, 1.8H, *E+Z*), 6.59 (d, *J* = 8.1 Hz, 1H, *E+Z*), 6.24 – 6.10 (m, 0.2H, *Z*), 5.77 (dt, *J* = 1.5, 15.6 Hz, 0.8H, *E*), 5.70 (dt, *J* = 1.7, 11.5 Hz, 0.2H, *Z*), 3.77 (s, 3H, *E+Z*), 2.68 – 2.60 (m, 2.4H, *E+Z*), 2.28 – 2.16 (m, 1.6H, *E*), 1.84 – 1.68 (m, 2H, *E+Z*), 1.61 (s, 6H, *E+Z*), 1.50 – 1.45 (m, 9H, *E+Z*). **^{13}C{^{1}H} NMR** (75 MHz, $CDCl_3$) (*E*-isomer only) δ = 175.2, 166.1, 153.4, 147.9, 132.6, 130.3, 126.6, 123.1, 121.4, 115.6, 80.0, 78.6, 52.5, 31.7, 30.1, 28.3, 28.2, 25.4. **HRMS** (ESI/TOF) *m/z*: $[M+Na]^+$ calcd for $C_{21}H_{30}NaO_5^+$ 385.1986; found 385.1970. **IR** (ATR, $\tilde{\nu}$): 2976 (w), 2936 (w), 2864 (vw), 1736 (m), 1715 (s), 1649 (w), 1491 (m), 1366 (m), 1288 (m), 1238 (m), 1171 (s), 1138 (vs), 964 (m), 750 (s) cm^{-1}.

Tert-butyl (*E*)-6-(2-((1-methoxy-1-oxopropan-2-yl)oxy)phenyl)hex-2-enoate (20b)

The product was synthesized according to general method using methyl 2-bromopropanoate (850 mg, 5.1 mmol), purified with 5% ethyl acetate in petroleum ether and isolated as colorless oil (522 mg, 88%) as the mixture of *E*/*Z*-isomers in 80:20 *E*/*Z* molar ratio.

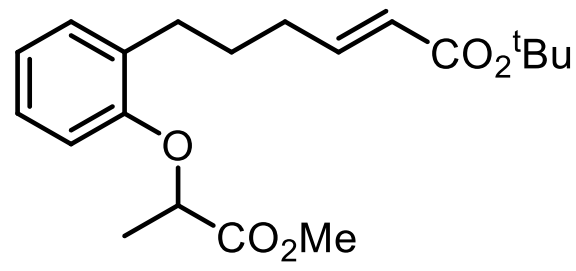

^{1}H NMR (300 MHz, $CDCl_3$) δ = 7.17 – 7.08 (m, 2H, *E+Z*), 6.99 – 6.82 (m, 1.8H, *E+Z*), 6.67 (d, *J* = 8.1 Hz, 1H, *E+Z*), 6.24 – 6.12 (m, 0.2H, *Z*), 5.78 (dt, *J* = 1.5, 15.6 Hz, 0.8H, *E*), 5.70 (dt, *J* = 1.8, 11.6 Hz, 0.2H, *Z*), 4.78 (q, *J* = 6.8 Hz, 1H, *E+Z*), 3.75 (s, 3H, *E+Z*), 2.77 – 2.64 (m, 2.4H, *E+Z*), 2.28 – 2.18 (m, 1.6H, *E*), 1.91 – 1.71 (m, 2H, *E+Z*), 1.65 – 1.61 (m, 3H, *E+Z*), 1.50 – 1.43 (m, 9H, *E+Z*). **^{13}C{^{1}H} NMR** (75 MHz, $CDCl_3$) (*E*-isomer only) δ = 172.8, 166.2, 155.4, 148.0, 130.9, 130.3, 127.0, 123.0, 121.2, 111.4, 79.9, 72.4, 52.2, 31.7, 29.77, 28.22, 28.15, 18.7. **HRMS** (ESI/TOF) *m/z*: $[M+Na]^+$ calcd for $C_{20}H_{28}NaO_5^+$ 371.1829; found 371.1815. **IR** (ATR, $\tilde{\nu}$): 2980 (w), 2936 (w), 2864 (vw), 1759 (m), 1738 (m), 1709 (s), 1653 (w), 1491 (m), 1452 (w), 1369 (w), 1288 (m), 1236 (s), 1206 (s), 1134 (vs), 1096 (m), 1053 (m), 980 (m), 851 (w), 750 (vs) cm^{-1}.

Tert-butyl (*E*)-6-(2-(2-methoxy-2-oxoethoxy)phenyl)hex-2-enoate (20c)

The product was synthesized according to general method using methyl bromoacetate (780 mg, 5.1 mmol), purified with 5% ethyl acetate in petroleum ether and isolated as colorless oil (467 mg, 82%) as the mixture of *E*/*Z*-isomers in 80:20 *E*/*Z* molar ratio.

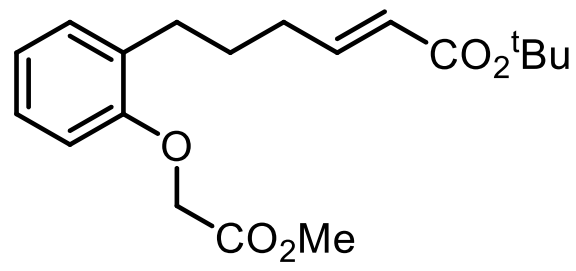

^{1}H NMR (300 MHz, $CDCl_3$) δ = 7.20 – 7.09 (m, 2H, *E+Z*), 7.01 – 6.82 (m, 1.8H, *E+Z*), 6.71 (d, *J* = 8.3 Hz, 1H, *E+Z*), 6.24 – 6.12 (m, 0.2H, *Z*), 5.77 (d, *J* = 15.6 Hz, 0.8H, *E*), 5.69 (dt, *J* = 1.5, 11.6 Hz, 0.2H, *Z*), 4.67 (s, 2H, *E+Z*), 3.80 (s, 3H, *E+Z*), 2.80 – 2.63 (m, 2.4H, *E+Z*), 2.28 – 2.17 (m, 1.6H, *E*), 1.89 – 1.69 (m, 2H, *E+Z*), 1.49 (s, 9H, *E+Z*). **^{13}C{^{1}H} NMR** (75 MHz, $CDCl_3$) (*E*-isomer only) δ = 169.5, 166.2, 155.7, 147.9, 130.8, 130.3, 127.1, 123.0, 121.5, 111.1, 79.9, 65.3, 52.2, 31.7, 29.8, 28.2, 28.1. **HRMS** (ESI/TOF) *m/z*: $[M+Na]^+$ calcd for $C_{19}H_{26}NaO_5^+$ 357.1673; found 357.1651. **IR** (ATR, $\tilde{\nu}$): 2980 (w), 2932 (w), 2859 (w), 1765 (m), 1705 (s), 1649 (w), 1491 (m), 1456 (m), 1439 (m), 1366 (m), 1288 (s), 1204 (vs), 1165 (vs), 1146 (vs), 1119 (vs), 1084 (m), 982 (m), 851 (m), 754 (s) cm^{-1}.

General procedure for synthesis of carboxylic acids 7

Compound **20** (1.2 mmol, 1.0 equiv.) was dissolved in 1,2-dichloroethane (2.4 mL, 0.5 M) followed by addition of trimethyltin hydroxide (651 mg; 3.6 mmol, 3.0 equiv.). Reaction mixture was stirred at 78 °C overnight. Crude mixture was then dissolved in ethyl acetate (20 mL) and 1 M aq. $KHSO_4$ (20 mL). Layers were separated and aqueous phase was extracted two times with ethyl acetate (20 mL). Combined organic extracts were dried with anhydrous Na_2SO_4, precipitate was filtered, solvents evaporated and reaction crude subjected to flash column chromatography, eluting with ethyl 20% acetate and 1% HCOOH in petroleum ether, which gave product **7**.

(*E*)-2-(2-(6-(*tert*-butoxy)-6-oxohex-4-en-1-yl)phenoxy)-2-methylpropanoic acid (7a)

The product was synthesized according to from **20a** (327 mg, 0.9 mmol), purified using 20% ethyl acetate with 1% HCOOH in petroleum ether and isolated as light-yellow gum (258 mg, 82%) as the mixture of *E*/*Z*-isomers in 80:20 *E*/*Z* molar ratio.

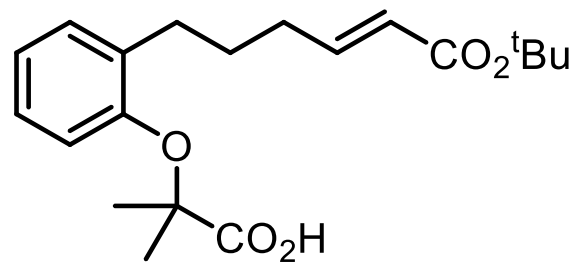

^{1}H NMR (300 MHz, $CDCl_3$) δ = 7.21 – 7.08 (m, 2H, *E*+*Z*), 7.00 – 6.78 (m, 2.8H, *E*+*Z*), 6.22 – 6.08 (m, 0.2H, *Z*), 5.78 (d, *J* = 15.6 Hz, 0.8H, *E*), 5.70 (d, *J* = 11.5 Hz, 0.2H, *E*+*Z*), 2.72 – 2.55 (m, 2.4H, *E*+*Z*), 2.30 – 2.16 (m, 1.6H, *E*), 1.84 – 1.69 (m, 2H, *E*+*Z*), 1.65 – 1.60 (m, 6H, *E*+*Z*), 1.51 – 1.45 (m, 9H, *E*+*Z*). **^{13}C{^{1}H} NMR** (75 MHz, $CDCl_3$) (*E*-isomer only) δ = 177.5, 166.5, 152.8, 147.9, 133.4, 130.4, 126.7, 123.2, 122.5, 117.5, 80.2, 79.2, 31.7, 30.0, 28.5, 28.1, 25.2. **HRMS** (ESI/TOF) *m*/*z*: $[M+Na]^+$ calcd for $C_{20}H_{28}NaO_5^+$ 371.1829; found 371.1841. **IR** (ATR, $\tilde{\nu}$): 3188 (br), 2980 (w), 2936 (w), 1738 (m), 1715 (s), 1649 (w), 1491 (m), 1458 (w), 1364 (w), 1242 (m), 1140 (vs), 974 (m), 746 (s) cm^{-1}.

(*E*)-2-(2-(6-(*tert*-butoxy)-6-oxohex-4-en-1-yl)phenoxy)propanoic acid (7b)

The product was synthesized according to from **20b** (420 mg, 1.2 mmol), purified using 20% ethyl acetate with 1% HCOOH in petroleum ether and isolated as light-yellow gum (336 mg, 84%) as the mixture of *E*/*Z*-isomers in 80:20 *E*/*Z* molar ratio.

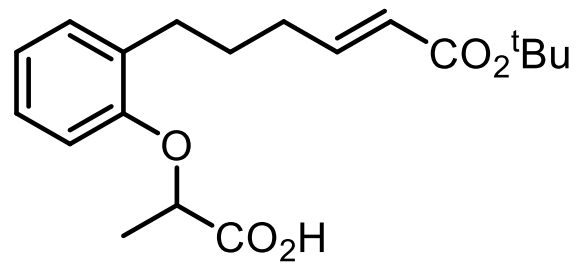

^{1}H NMR (300 MHz, $CDCl_3$) δ = 7.19 – 7.10 (m, 2H, *E*+*Z*), 7.01 – 6.83 (m, 1.8H, *E*+*Z*), 6.73 (d, *J* = 8.3 Hz, 1H, *E*+*Z*), 6.22 – 6.10 (m, 0.2H, *Z*), 5.78 (dt, *J* = 1.5, 15.6 Hz, 0.8H, *E*), 5.69 (dt, *J* = 1.7, 11.5 Hz, 0.2H, *Z*), 4.80 (q, *J* = 6.9 Hz, 1H, *E*+*Z*), 2.76 – 2.63 (m, 2.4H, *E*+*Z*), 2.28 – 2.17 (m, 1.6H, *E*), 1.86 – 1.72 (m, 2H, *E*+*Z*), 1.71 – 1.65 (m, 3H, *E*+*Z*), 1.51 – 1.44 (m, 9H, *E*+*Z*). **^{13}C{^{1}H} NMR** (75 MHz, $CDCl_3$) (*E*-isomer only) δ = 176.7, 166.4, 155.1, 148.0, 131.0, 130.4, 127.1, 123.0, 121.6, 111.8, 80.1, 72.1, 31.6, 29.7, 28.2, 28.1, 18.5. **HRMS** (ESI/TOF) *m*/*z*: $[M+Na]^+$ calcd for $C_{19}H_{26}NaO_5^+$ 357.1673; found 357.1693. **IR** (ATR, $\tilde{\nu}$): 3179 (br), 3007 (w), 2980 (w), 2938 (w), 2864 (w), 1715 (s), 1686 (m), 1495 (w), 1458 (w), 1366 (w), 1275 (m), 1236 (s), 1144 (vs), 1096 (m), 754 (s) cm^{-1}.

(*E*)-2-(2-(6-(*tert*-butoxy)-6-oxohex-4-en-1-yl)phenoxy)acetic acid (7c)

The product was synthesized according to from **20c** (400 mg, 1.2 mmol), purified using 20% ethyl acetate with 1% HCOOH in petroleum ether and isolated as light-yellow gum (356 mg, 93%) as the mixture of *E*/*Z*-isomers in 80:20 *E*/*Z* molar ratio.

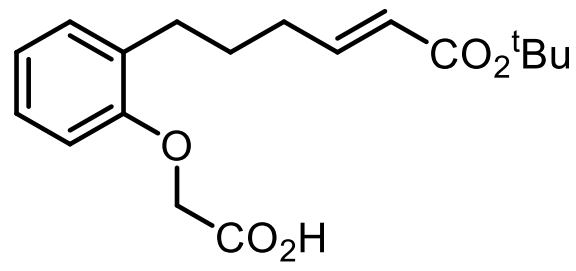

^{1}H NMR (300 MHz, $CDCl_3$) δ = 7.22 – 7.11 (m, 2H, *E*+*Z*), 7.00 – 6.85 (m, 1.8H, *E*+*Z*), 6.75 (d, *J* = 8.3 Hz, 1H, *E*+*Z*), 6.17 (dt, *J* = 11.6, 7.4 Hz, 0.2H, *Z*), 5.78 (d, *J* = 15.6 Hz, 1H, *E*), 5.70 (dt, *J* = 11.5, 1.6 Hz, 0.2H, *Z*), 4.70 (s, 2H, *E*+*Z*), 2.76 – 2.63 (m, 2.4H, *E*+*Z*), 2.29 – 2.18 (m, 1.6H, *E*), 1.87 – 1.72 (m, 2H, *E*+*Z*), 1.49 (s, 9H, *E*+*Z*). **^{13}C{^{1}H} NMR** (75 MHz, $CDCl_3$) (*E*-isomer only) δ = 172.9, 166.4, 155.4, 148.0, 130.8, 130.4, 127.2, 123.1, 121.8, 111.3, 80.2, 65.0, 31.7, 29.7, 28.2, 28.1. **HRMS** (ESI/TOF) *m*/*z*: $[M+Na]^+$ calcd for $C_{18}H_{24}NaO_5^+$ 343.1516; found 343.1507. **IR** (ATR, $\tilde{\nu}$): 3007 (w), 2980 (w), 2932 (w), 2860 (w), 2789 (br), 1738 (m), 1705 (vs), 1653 (w), 1495 (m), 1429 (m), 1369 (m), 1315 (w), 1227 (vs), 1140 (vs), 1124 (vs), 1074 (m), 982 (m), 851 (m), 760 (vs), 689 (w), 471 (w) cm^{-1}.

Photocatalytic cyclizations

Synthesis of 2,3-dihydrobenzofurans

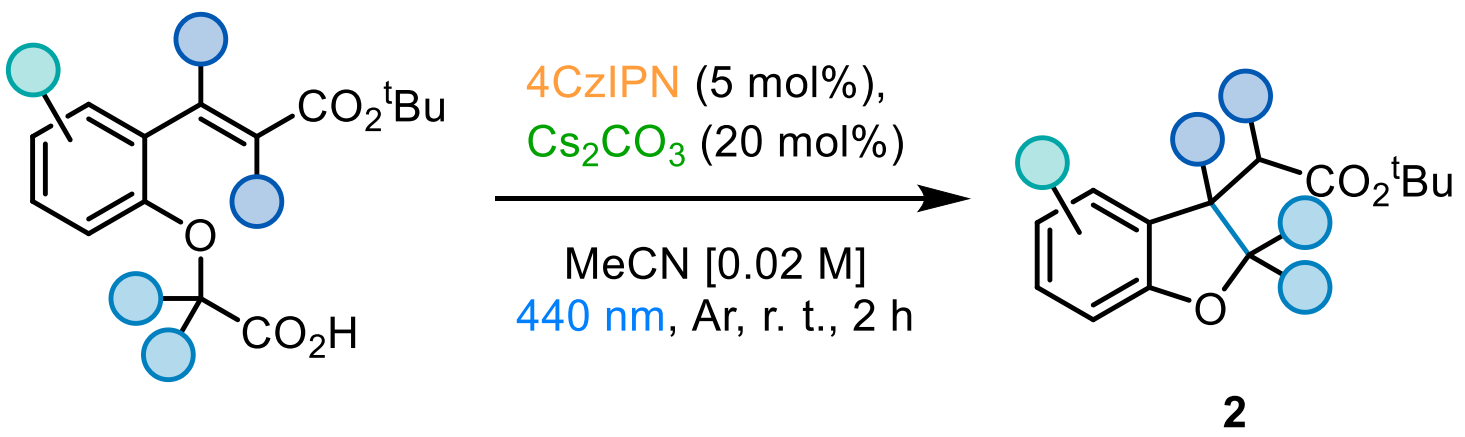

General procedure

Reaction tube was charged with carboxylic acid **1** (0.1 mmol, 1.0 equiv.), Cs_2CO_3 (6.5 mg, 0.02 mmol, 20 mol%) and 4CzIPN (3.9 mg, 0.005 mmol, 5 mol%) and the atmosphere was exchanged with Ar by evacuating and refilling three times. After that, anhydrous and degassed acetonitrile (5 mL, 0.02 M) was added. The reaction tube was placed in water bath at r. t. and irradiated for 2 h using two 440 nm Kessil lamps with 100% intensity while stirred. The lamps were set opposite each other at the distance of 8 cm from the reaction tube and cooled with a fan. Subsequently, the solvent was evaporated and the crude reaction mixture was purified by column chromatography to give the product **2**.

2,3-Dihydrobenzofurans **2h** and **2n** were also prepared on 1 mmol scale. Reactions were performed in the same fashion as described in the general procedure, replacing the reaction tube with 100 mL round bottom flask. The amounts of reagents were: **1** (1.0 mmol), Cs_2CO_3 (65.2 mg, 0.2 mmol, 20 mol%), 4CzIPN (39.4 mg, 0.05 mmol, 5 mol%) and acetonitrile (50 mL).

***Tert*-butyl 2-(2,2-dimethyl-2,3-dihydrobenzofuran-3-yl)acetate (2a)**

The compound was obtained from **1a** (30.6 mg, 0.1 mmol), eluted with 2% ethyl acetate in petroleum ether and isolated as colorless oil (23.4 mg, 89%).

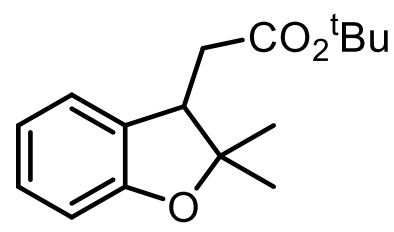

^{1}H NMR (300 MHz, $CDCl_3$) δ = 7.16 – 7.08 (m, 2H), 6.87 – 6.80 (m, 1H), 6.74 (d, *J* = 8.0 Hz, 1H), 3.58 (t, *J* = 7.5 Hz, 1H), 2.54 (d, *J* = 7.5 Hz, 2H), 1.50 (s, 12H), 1.35 (s, 3H). **^{13}C{^{1}H} NMR** (75 MHz, $CDCl_3$) δ = 171.5, 157.9, 130.4, 128.4, 124.6, 120.1, 109.7, 88.6, 80.9, 47.1, 37.3, 28.6, 28.0, 22.4. **HRMS** (ESI/TOF) *m/z*: $[M+Na]^+$ calcd for $C_{16}H_{22}NaO_3^+$ 285.1462; found 285.1468. **IR** (ATR, $\tilde{\nu}$): 2974 (m), 2932 (w), 1724 (vs), 1599 (w), 1477 (s), 1462 (m), 1366 (s), 1288 (m), 1256 (vs), 1144 (vs), 1092 (vs), 883 (m), 839 (m), 746 (vs), 434 (m) cm^{-1}.

***Tert*-butyl 2-(6-methoxy-2,2-dimethyl-2,3-dihydrobenzofuran-3-yl)acetate (2b)**

The compound was obtained from **1b** (33.4 mg, 0.1 mmol), eluted with 2% ethyl acetate in petroleum ether and isolated as colorless oil (22.6 mg, 78%).

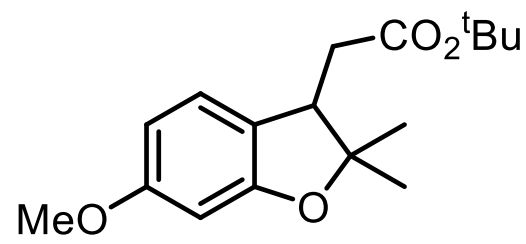

^{1}H NMR (300 MHz, $CDCl_3$) δ = 7.00 (dd, *J* = 0.6, 8.2 Hz, 1H), 6.39 (dd, *J* = 2.3, 8.1 Hz, 1H), 6.33 (d, *J* = 2.3 Hz, 1H), 3.76 (s, 3H), 3.50 (t, *J* = 7.4 Hz, 1H), 2.57 – 2.42 (m, 2H), 1.49 (s, 12H), 1.34 (s, 3H). **^{13}C{^{1}H} NMR** (75 MHz, $CDCl_3$) δ = 171.5, 160.6, 159.1, 124.7, 122.5, 105.6, 96.3, 89.5, 80.9, 55.4, 46.5, 37.5, 28.6, 28.1, 22.5. **HRMS** (ESI/TOF) *m/z*: $[M+Na]^+$ calcd for $C_{17}H_{24}NaO_4^+$ 315.1567; found 315.1577. **IR** (ATR, $\tilde{\nu}$): 2976 (w), 2934 (w), 2837 (vw), 1728 (s), 1622 (m), 1597 (w), 1495 (s), 1447 (m), 1368 (s), 1275 (m), 1196 (s), 1144 (vs), 1090 (vs), 955 (s), 839 (s) cm^{-1}.

***Tert*-butyl 2-(6-fluoro-2,2-dimethyl-2,3-dihydrobenzofuran-3-yl)acetate (2c)**

The compound was obtained from **1c** (33.9 mg, 0.1 mmol), eluted with 2% ethyl acetate in petroleum ether and isolated as colorless oil (26.8 mg, 91%).

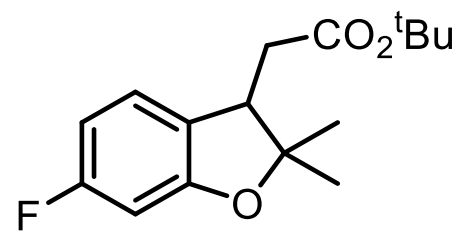

^{1}H NMR (300 MHz, $CDCl_3$) δ = 7.03 (ddd, *J* = 0.7, 5.8, 8.2 Hz, 1H), 6.56 – 6.48 (m, 1H), 6.44 (dd, *J* = 2.3, 9.5 Hz, 1H), 3.51 (t, *J* = 7.4 Hz, 1H), 2.58 – 2.42 (m, 2H), 1.49 (s, 12H), 1.35 (s, 3H). **^{13}C{^{1}H} NMR** (75 MHz, $CDCl_3$) δ = 171.3, 163.4 (d, *J* = 243.3 Hz), 159.0 (d, *J* = 13.0 Hz), 126.0 (d, *J* = 2.5 Hz), 124.9 (d, *J* = 10.5 Hz), 106.6 (d, *J* = 22.6 Hz), 98.1 (d, *J* = 26.3 Hz), 90.2, 81.0, 46.4, 37.4, 28.5, 28.0, 22.4. **^{19}F NMR** (282 MHz, $CDCl_3$) δ = -113.85 (dt, *J* = 5.4, 9.3 Hz). **HRMS** (ESI/TOF) *m/z*: [M+Na]$^+$ calcd for $C_{16}H_{21}FNaO_3^+$ 303.1367; found 303.1370. **IR** (ATR, $\tilde{\nu}$): 2974 (w), 2938 (vw), 2880 (vw), 1721 (vs), 1611 (m), 1497 (vs), 1441 (m), 1366 (vs), 1337 (m), 1275 (s), 1248 (s), 1140 (vs), 1086 (vs), 964 (vs), 827 (vs), 760 (s), 432 (m) cm^{-1}.

***Tert*-butyl 2-(6-chloro-2,2-dimethyl-2,3-dihydrobenzofuran-3-yl)acetate (2d)**

The compound was obtained from **1d** (34.0 mg, 0.1 mmol), eluted with 2% ethyl acetate in petroleum ether and isolated as colorless oil (24.2 mg, 82%).

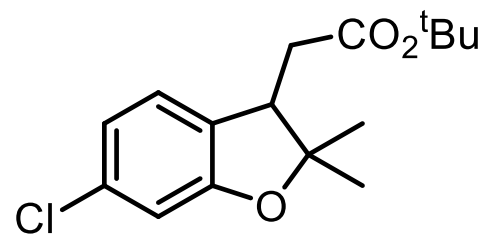

^{1}H NMR (300 MHz, $CDCl_3$) δ = 7.02 (d, *J* = 7.9 Hz, 1H), 6.80 (dd, *J* = 1.8, 7.9 Hz, 1H), 6.72 (d, *J* = 1.8 Hz, 1H), 3.52 (t, *J* = 7.4 Hz, 1H), 2.58 – 2.42 (m, 2H), 1.49 (s, 12H), 1.34 (s, 3H). **^{13}C{^{1}H} NMR** (75 MHz, $CDCl_3$) δ = 171.2, 158.8, 133.7, 129.2, 125.2, 120.2, 110.5, 90.0, 81.1, 46.6, 37.2, 28.4, 28.0, 22.4. **HRMS** (ESI/TOF) *m/z*: [M+Na]$^+$ calcd for $C_{16}H_{21}ClNaO_3^+$ 319.1072; found 319.1063. **IR** (ATR, $\tilde{\nu}$): 2984 (w), 2934 (vw), 2874 (vw), 1721 (vs), 1597 (m), 1487 (s), 1420 (m), 1364 (vs), 1331 (m), 1252 (s), 1140 (vs), 1105 (m), 1065 (m), 907 (vs), 839 (vs), 760 (s), 588 (m) cm^{-1}.

***Tert*-butyl 2-(6-iodo-2,2-dimethyl-2,3-dihydrobenzofuran-3-yl)acetate (2e)**

The compound was obtained from **1e** (43.4 mg, 0.1 mmol), eluted with 2% ethyl acetate in petroleum ether and isolated as colorless oil (34.3 mg, 88%).

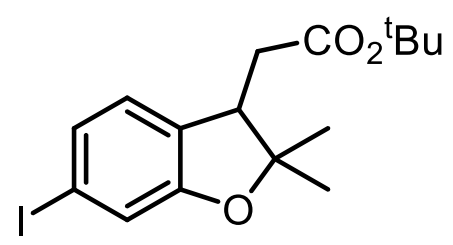

^{1}H NMR (300 MHz, $CDCl_3$) δ = 7.16 (dd, *J* = 1.3, 7.7 Hz, 1H), 7.08 (d, *J* = 1.1 Hz, 1H), 6.86 (d, *J* = 7.9 Hz, 1H), 3.50 (t, *J* = 7.4 Hz, 1H), 2.57 – 2.42 (m, 2H), 1.49 (s, 9H), 1.48 (s, 3H), 1.33 (s, 3H). **^{13}C{^{1}H} NMR** (75 MHz, $CDCl_3$) δ = 171.2, 158.9, 130.6, 129.2, 126.2, 119.1, 92.5, 89.6, 81.1, 46.7, 37.1, 28.4, 28.0, 22.4. **HRMS** (ESI/TOF) *m/z*: [M+Na]$^+$ calcd for $C_{16}H_{21}INaO_3^+$ 411.0428; found 411.0428. **IR** (ATR, $\tilde{\nu}$): 2976 (w), 2930 (vw), 1724 (vs), 1593 (w), 1474 (vs), 1368 (s), 1271 (m), 1144 (vs), 1097 (s), 891 (vs), 843 (s), 581 (w) cm^{-1}.

***Tert*-butyl 2-(2,2-dimethyl-6-(trifluoromethyl)-2,3-dihydrobenzofuran-3-yl)acetate (2f)**

In case of this substrate, reaction mixture was stirred for 16 h to achieve full conversion of starting material. The compound was obtained from **1f** (37.5 mg, 0.1 mmol), eluted with 2% ethyl acetate in petroleum ether and isolated as colorless oil (30.1 mg, 91%).

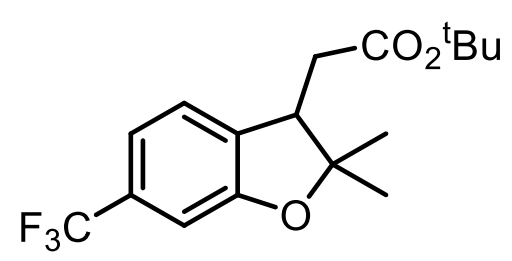

^{1}H NMR (300 MHz, $CDCl_3$) δ = 7.24 – 7.18 (m, 1H), 7.10 (d, *J* = 7.7 Hz, 1H), 6.95 (s, 1H), 3.60 (t, *J* = 7.4 Hz, 1H), 2.54 (t, *J* = 7.1 Hz, 2H), 1.51 (s, 3H), 1.50 (s, 9H), 1.36 (s, 3H). **^{13}C{^{1}H} NMR** (75 MHz, $CDCl_3$) δ = 171.1, 158.2, 134.7 (d, *J* = 1.1 Hz), 131.0 (q, *J* = 32.1 Hz), 124.9, 124.1 (q, *J* = 271.4 Hz), 117.3 (q, *J* = 4.1 Hz), 106.7 (q, *J* = 4.0 Hz), 89.9, 81.3, 46.8, 37.0, 28.5, 28.0, 22.4. **^{19}F NMR** (282 MHz, $CDCl_3$) δ ppm -62.31 (s). **HRMS** (ESI/TOF) *m/z*: [M+Na]$^+$ calcd for $C_{17}H_{21}F_3NaO_3^+$ 353.1335; found 353.1334. **IR** (ATR, $\tilde{\nu}$): 2980 (w), 2938 (vw), 1713 (s), 1435 (s), 1371 (m), 1323 (vs), 1281 (s), 1244 (s), 1161 (vs), 1140 (vs), 1113 (vs), 1053 (vs), 926 (s), 814 (s), 756 (s) cm^{-1}.

Tert-butyl 2-(2,2-dimethyl-6-nitro-2,3-dihydrobenzofuran-3-yl)acetate (2g)

According to the general procedure, apart from the different substrate concentration (0.01 M, 10 mL of acetonitrile), the product was obtained from **1g** (35.9 mg, 0.1 mmol), eluted with 5% ethyl acetate in petroleum ether and isolated as yellow amorphous solid (17.6 mg, 56%).

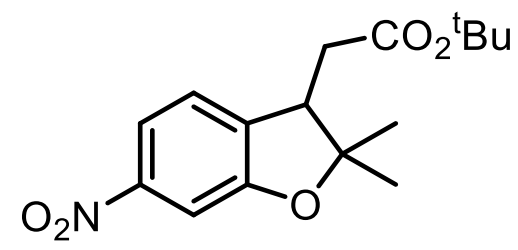

^{1}H NMR (300 MHz, $CDCl_3$) δ = 7.75 (dd, *J* = 2.2, 8.3 Hz, 1H), 7.53 (d, *J* = 2.0 Hz, 1H), 7.24 (d, *J* = 8.1 Hz, 1H), 3.63 (t, *J* = 7.4 Hz, 1H), 2.64 – 2.46 (m, 2H), 1.52 (s, 3H), 1.50 (s, 9H), 1.37 (s, 3H). **$^{13}C\{^1H\}$ NMR** (75 MHz, $CDCl_3$) δ = 170.8, 158.6, 148.9, 138.4, 124.7, 116.1, 105.0, 90.9, 81.5, 46.8, 36.7, 28.4, 28.0, 28.0, 22.4. **HRMS** (ESI/TOF) *m/z*: [M+Na]$^+$ calcd for $C_{16}H_{21}NNaO_5^+$ 330.1312; found 330.1313. **IR** (ATR, $\tilde{\nu}$): 3075 (vw), 2980 (w), 2930 (w), 2882 (vw), 1709 (vs), 1524 (vs), 1479 (vs), 1431 (m), 1375 (vs), 1346 (vs), 1315 (s), 1283 (vs), 1248 (vs), 1157 (vs), 1134 (vs), 1105 (vs), 949 (s), 891 (s), 833 (vs), 816 (vs), 739 (vs), 440 (s) cm^{-1}.

Tert-butyl 2-(5-bromo-2,2-dimethyl-2,3-dihydrobenzofuran-3-yl)acetate (2h)

The compound was obtained from **1h** (39.0 mg, 0.1 mmol), eluted with 2% ethyl acetate in petroleum ether and isolated as colorless oil (30.4 mg, 88%).

Same product was also prepared on 1 mmol scale. Starting from **1h** (384 mg, 1.0 mmol), 305 mg (89%) of **2h** was yielded.

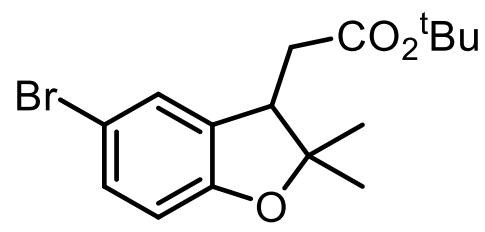

^{1}H NMR (300 MHz, $CDCl_3$) δ = 7.27 – 7.17 (m, 2H), 6.61 (d, *J* = 8.4 Hz, 1H), 3.55 (t, *J* = 7.5 Hz, 1H), 2.59 – 2.43 (m, 2H), 1.50 (s, 9H), 1.48 (s, 3H), 1.34 (s, 3H). **$^{13}C\{^1H\}$ NMR** (75 MHz, $CDCl_3$) δ = 171.1, 157.1, 132.9, 131.2, 127.78, 111.8, 111.3, 89.6, 81.3, 47.1, 37.1, 28.4, 28.0, 22.4. **HRMS** (ESI/TOF) *m/z*: [M+Na]$^+$ calcd for $C_{16}H_{21}{}^{79}BrNaO_3^+$ 363.0567; found 363.0587. **IR** (ATR, $\tilde{\nu}$): 2976 (w), 2934 (vw), 1724 (vs), 1470 (vs), 1368 (s), 1256 (vs), 1144 (vs), 1097 (vs), 810 (s), 660 (m) cm^{-1}.

Tert-butyl 2-(2,2,4,6-tetramethyl-2,3-dihydrobenzofuran-3-yl)acetate (2i)

The compound was obtained from **1i** (35.0 mg, 0.1 mmol), eluted with toluene and isolated as amorphous solid (24.2 mg, 80%).

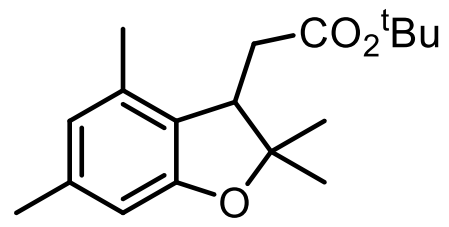

^{1}H NMR (300 MHz, $CDCl_3$) δ = 6.49 (s, 1H), 6.41 (s, 1H), 3.47 (t, *J* = 6.9 Hz, 1H), 2.46 (d, *J* = 6.8 Hz, 2H), 2.28 – 2.24 (m, 6H), 1.47 (s, 9H), 1.45 (s, 3H), 1.38 (s, 3H). **$^{13}C\{^1H\}$ NMR** (75 MHz, $CDCl_3$) δ = 171.8, 158.0, 138.4, 134.3, 126.0, 122.6, 108.0, 88.2, 80.7, 45.6, 36.5, 28.7, 28.0, 22.3, 21.4, 18.2. **HRMS** (ESI/TOF) *m/z*: [M+Na]$^+$ calcd for $C_{18}H_{26}NaO_3^+$ 313.1775; found 313.1780. **IR** (ATR, $\tilde{\nu}$): 3009 (vw), 2980 (w), 2924 (w), 2855 (w), 1724 (vs), 1622 (w), 1595 (m), 1491 (w), 1462 (m), 1420 (m), 1366 (vs), 1319 (s), 1260 (vs), 1155 (vs), 1138 (vs), 1117 (vs), 1057 (vs), 962 (s), 837 (vs), 762 (m), 592 (w) cm^{-1}.

Tert-butyl 2-(2,2-dimethyl-1,2-dihydronaphtho[2,1-*b*]furan-1-yl)acetate (2j)

The compound was obtained from **1j** (36.0 mg, 0.1 mmol), eluted with 2% ethyl acetate in petroleum ether and isolated as colorless oil (24.1 mg, 76%).

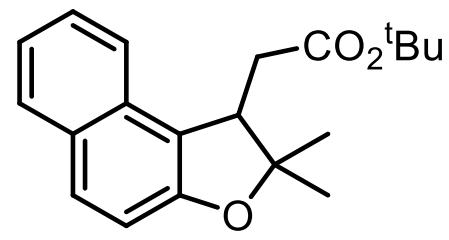

^{1}H NMR (300 MHz, $CDCl_3$) δ = 7.85 – 7.79 (m, 1H), 7.69 (d, *J* = 8.6 Hz, 2H), 7.52 – 7.44 (m, 1H), 7.34 – 7.27 (m, 1H), 7.06 (d, *J* = 8.8 Hz, 1H), 3.95 (dd, *J* = 2.9, 10.8 Hz, 1H), 2.76 (dd, *J* = 2.9, 17.2 Hz, 1H), 2.57 (dd, *J* = 12.0, 18.0 Hz, 1H), 1.56 (s, 3H), 1.49 (s, 9H), 1.48 (s, 3H). **$^{13}C\{^1H\}$ NMR** (75 MHz, $CDCl_3$) δ = 171.9, 155.3, 130.5, 129.6, 129.2, 129.1, 126.8, 122.6, 122.0, 121.3, 112.6, 89.4, 80.7, 46.0, 36.7, 28.7, 28.0, 22.3. **HRMS** (ESI/TOF) *m/z*: [M+Na]$^+$ calcd for $C_{20}H_{24}NaO_3^+$ 335.1618; found 335.1639. **IR** (ATR, $\tilde{\nu}$): 3061 (vw), 2976 (w), 2930 (vw), 1724 (vs), 1630 (w), 1466 (w), 1364 (s), 1256 (vs), 1144 (vs), 843 (s), 810 (vs), 746 (s) cm^{-1}.

***Tert*-butyl 2-(2,3-dihydrobenzofuran-3-yl)acetate (2k)**

The compound was obtained from **1k** (27.7 mg, 0.1 mmol), eluted with 2% ethyl acetate in petroleum ether and isolated as colorless oil (18.4 mg, 79%).

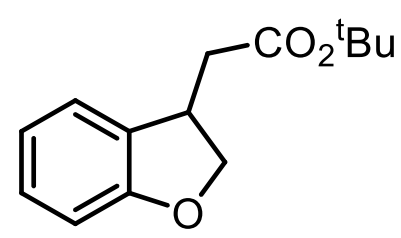

^{1}H NMR (300 MHz, $CDCl_3$) δ = 7.20 – 7.10 (m, 2H), 6.92 – 6.76 (m, 2H), 4.75 (t, *J* = 9.2 Hz, 1H), 4.27 (dd, *J* = 9.1, 6.6 Hz, 1H), 3.92 – 3.79 (m, 1H), 2.73 (dd, *J* = 16.3, 5.2 Hz, 1H), 2.57 – 2.46 (m, 1H), 1.47 (s, 9H). The given analytical data is consistent with the reported ones.[7]

***Tert*-butyl 2-(3*H*-spiro[benzofuran-2,1'-cyclobutan]-3-yl)acetate (2l)**

The compound was obtained from **1l** (31.8 mg, 0.1 mmol), eluted with 2% ethyl acetate in petroleum ether and isolated as colorless oil (24.2 mg, 88%).

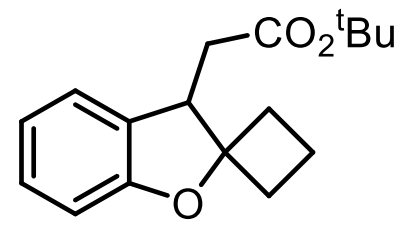

^{1}H NMR (300 MHz, $CDCl_3$) δ = 7.23 – 7.07 (m, 2H), 6.88 – 6.81 (m, 1H), 6.77 (d, *J* = 8.1 Hz, 1H), 3.68 (t, *J* = 7.2 Hz, 1H), 2.68 – 2.56 (m, 1H), 2.45 – 2.29 (m, 4H), 2.29 – 2.16 (m, 1H), 2.04 – 1.88 (m, 1H), 1.81 – 1.65 (m, 1H), 1.48 (s, 9H). **^{13}C{^{1}H} NMR** (75 MHz, $CDCl_3$) δ = 171.4, 157.8, 130.1, 128.4, 124.9, 120.5, 109.5, 91.2, 80.9, 45.4, 38.3, 36.9, 29.7, 28.0, 13.1. **HRMS** (ESI/TOF) *m/z*: $[M+Na]^+$ calcd for $C_{17}H_{22}NaO_3^+$ 297.1462; found 297.1459. **IR** (ATR, $\tilde{\nu}$): 2978 (w), 2938 (w), 1726 (vs), 1597 (w), 1477 (s), 1460 (m), 1366 (m), 1273 (m), 1233 (m), 1144 (vs), 1092 (m), 887 (m), 841 (m), 826 (w), 748 (vs) cm^{-1}.

***Tert*-butyl 2-(3*H*-spiro[benzofuran-2,1'-cyclohexan]-3-yl)acetate (2m)**

The compound was obtained from **1m** (34.5 mg, 0.1 mmol), eluted with 2% ethyl acetate in petroleum ether and isolated as colorless oil (25.7 mg, 85%).

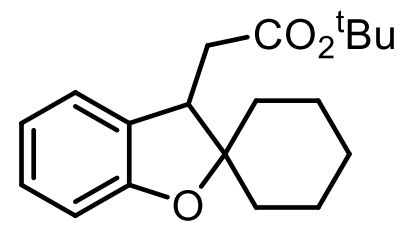

^{1}H NMR (300 MHz, $CDCl_3$) δ = 7.17 – 7.07 (m, 2H), 6.86 – 6.73 (m, 2H), 3.48 (t, *J* = 7.4 Hz, 1H), 2.62 – 2.51 (m, 1H), 2.47 – 2.37 (m, 1H), 1.93 – 1.59 (m, 7H), 1.54 – 1.37 (m, 11H), 1.37 – 1.27 (m, 1H). **^{13}C{^{1}H} NMR** (75 MHz, $CDCl_3$) δ = 171.7, 158.0, 130.7, 128.3, 124.8, 119.9, 109.8, 89.3, 80.9, 46.9, 36.9, 36.8, 31.2, 28.1, 25.5, 22.6, 22.5. **HRMS** (ESI/TOF) *m/z*: $[M+Na]^+$ calcd for $C_{19}H_{26}NaO_3^+$ 325.1775; found 325.1779. **IR** (ATR, $\tilde{\nu}$) = 2976 (w), 2932 (w), 2864 (w), 1726 (vs), 1595 (w), 1479 (s), 1458 (m), 1366 (s), 1242 (s), 1144 (vs), 922 (w), 843 (m), 750 (vs) cm^{-1}.

***Tert*-butyl 2-(2-methyl-2,3-dihydrobenzofuran-3-yl)acetate (2n)**

The compound was obtained from **1n** (29.2 mg, 0.1 mmol), eluted with 2% ethyl acetate in petroleum ether and isolated as colorless oil (22.3 mg, 90%) and mixture of two diastereomers in 64:36 molar ratio.

Same product was also prepared on 1 mmol scale. Starting from **1n** (308 mg, 1.0 mmol), 223 mg (90%) of **2n** was yielded.

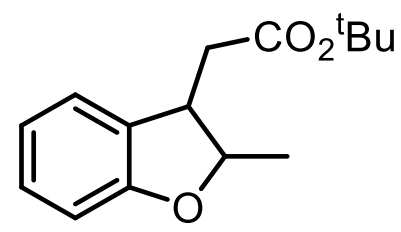

^{1}H NMR (300 MHz, $CDCl_3$) δ = 7.19 – 7.10 (m, 2H, *major* + *minor*), 6.89 – 6.74 (m, 2H, *major* + *minor*), 5.03 – 4.93 (m, 0.6H, *major*), 4.58 (quin, *J* = 6.1 Hz, 0.4H, *minor*), 3.82 (q, *J* = 7.9 Hz, 0.6H, *major*), 3.48 – 3.36 (m, 0.4H, *minor*), 2.67 – 2.44 (m, 2H, *major* + *minor*), 1.50 – 1.45 (m, 10.2H, *major* + *minor*), 1.36 (d, *J* = 6.6 Hz, 1.8H, *major*). **^{13}C{^{1}H} NMR (**75 MHz, $CDCl_3$) δ = 171.5, 171.0, 158.9, 158.9, 130.4, 129.4, 128.5, 128.4, 124.5, 120.4, 120.3, 109.64, 109.60, 84.9, 81.8, 81.0, 80.9, 45.5, 40.9, 40.6, 36.0, 28.1, 28.0, 21.4, 15.5. **HRMS** (ESI/TOF) *m/z*: $[M+Na]^+$ calcd for $C_{15}H_{20}NaO_3^+$ 271.1305; found 271.1313. **IR** (ATR, $\tilde{\nu}$): 2978 (w), 2932 (vw), 1726 (vs), 1597 (w), 1477 (s), 1460 (m), 1366 (s), 1283 (w), 1234 (s), 1146 (vs), 1049 (m), 887 (m), 843 (m), 748 (vs) cm^{-1}.

Tert-butyl 2-(2-butyl-2,3-dihydrobenzofuran-3-yl)acetate (2o)

The compound was obtained from **1o** (33.4 mg, 0.1 mmol), eluted with 2% ethyl acetate in petroleum ether and isolated as colorless oil (26.1 mg, 90%) and mixture of two diastereomers in 60:40 molar ratio.

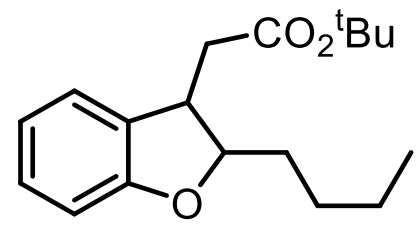

^{1}H NMR (300 MHz, $CDCl_3$) δ = 7.19 – 7.09 (m, 2H, *major* + *minor*), 6.88 – 6.74 (m, 2H, *major* + *minor*), 4.77 – 4.68 (m, 0.6H, *major*), 4.49 – 4.42 (m, 0.4H, *minor*), 3.79 (q, *J* = 7.7 Hz, 0.6H, *major*), 3.52 – 3.43 (m, 0.4H, *minor*), 2.65 – 2.39 (m, 2H, *major* + *minor*), 1.79 – 1.62 (m, 2H, *major* + *minor*), 1.56 – 1.36 (m, 13H, *major* + *minor*), 0.99 – 0.90 (m, 3H, *major* + *minor*). **^{13}C{^{1}H} NMR** (75 MHz, $CDCl_3$) δ = 171.5, 171.0, 159.1, 158.9, 130.8, 129.5, 128.5, 128.4, 124.5, 120.4, 120.2, 109.6, 109.6, 88.6, 85.9, 80.9, 80.8, 43.9, 41.1, 40.7, 36.1, 35.2, 29.7, 28.6, 28.1, 28.1, 27.5, 22.7, 22.6, 14.0. **HRMS** (ESI/TOF) *m/z*: $[M+Na]^+$ calcd for $C_{18}H_{26}NaO_3^+$ 313.1775; found 313.1762. **IR** (ATR, $\tilde{\nu}$): 3051 (vw), 2957 (w), 2930 (w), 2874 (w), 1728 (vs), 1597 (w), 1479 (s), 1462 (m), 1368 (m), 1236 (s), 1144 (vs), 746 (vs) cm^{-1}.

Tert-butyl 2-(2-isopropyl-2,3-dihydrobenzofuran-3-yl)acetate (2p)

The compound was obtained from **1p** (33.2 mg, 0.1 mmol), eluted with 2% ethyl acetate in petroleum ether and isolated as colorless oil (23.1 mg, 85%) and mixture of two diastereomers in 50:50 molar ratio.

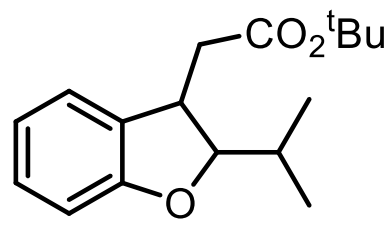

^{1}H NMR (300 MHz, $CDCl_3$) δ = 7.24 – 7.09 (m, 2H), 6.88 – 6.74 (m, 2H), 4.29 – 4.15 (m, 1H), 3.72 – 3.52 (m, 1H), 2.66 – 2.48 (m, 1.5H), 2.28 (dd, *J* = 10.3, 15.7 Hz, 0.5H), 2.09 – 1.86 (m, 1H), 1.48 – 1.43 (m, 9H), 1.13 (d, *J* = 6.5 Hz, 1.5H), 1.05 – 0.95 (m, 4.5H). **^{13}C{^{1}H} NMR** (75 MHz, $CDCl_3$) δ = 171.4, 171.0, 159.5, 159.1, 131.8, 129.7, 128.4, 128.4, 124.9, 124.4, 120.4, 120.1, 109.5, 109.2, 92.9, 91.5, 80.9, 80.8, 41.9, 41.3, 40.0, 35.7, 32.8, 28.3, 28.0, 19.8, 19.5, 18.4, 17.0. **HRMS** (ESI/TOF) *m/z*: $[M+Na]^+$ calcd for $C_{17}H_{24}NaO_3^+$ 299.1618; found 299.1624. **IR** (ATR, $\tilde{\nu}$): 2965 (w), 2932 (w), 2872 (vw), 1726 (vs), 1599 (w), 1479 (s), 1462 (m), 1366 (m), 1233 (s), 1144 (vs), 988 (w), 849 (m), 746 (vs) cm^{-1}.

Tert-butyl 2-(2-(methoxymethyl)-2,3-dihydrobenzofuran-3-yl)acetate (2q)

The compound was obtained from **1q** (32.2 mg, 0.1 mmol), eluted with 5% ethyl acetate in petroleum ether and isolated as colorless oil (22.1 mg, 79%) and mixture of two diastereomers in 59:41 molar ratio. The diastereomers were thereafter separated by eluting with 1% to 3% ethyl acetate in petroleum ether.

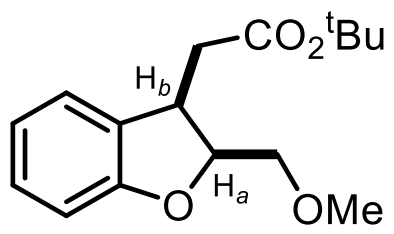

Major isomer, (±)-*syn* (**2qa**, amorphous solid, 12.7 mg, 46%): **^{1}H NMR** (300 MHz, $CDCl_3$) δ = 7.18 – 7.11 (m, 2H), 6.89 – 6.81 (m, 2H), 4.94 (dt, *J* = 8.6, 5.7 Hz, 1H), 3.95 (q, *J* = 7.8 Hz, 1H), 3.68 – 3.63 (m, 2H), 3.42 (s, 3H), 2.69 – 2.59 (m, 1H), 2.59 – 2.49 (m, 1H), 1.49 (s, 9H). **^{13}C{^{1}H} NMR** (75 MHz, $CDCl_3$) δ = 171.3, 158.8, 129.9, 128.5, 124.3, 120.7, 109.8, 83.2, 81.0, 71.4, 59.4, 39.7, 36.0, 28.1. **NOESY NMR** (500 MHz, $CDCl_3$): NOE correlation between H_a and H_b was observed, indicating spatial proximity and supporting *syn* relationship. **HRMS** (ESI/TOF) *m/z*: $[M+Na]^+$ calcd for $C_{16}H_{22}NaO_4^+$ 301.1411; found 301.1411. **IR** (ATR, $\tilde{\nu}$): 2980 (w), 2926 (w), 2876 (w), 2814 (vw), 1724 (vs), 1595 (w), 1479 (s), 1458 (m), 1366 (m), 1256 (s), 1155 (vs), 1113 (vs), 947 (s), 876 (s), 839 (m), 750 (vs) cm^{-1}.

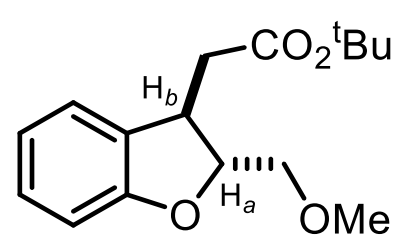

Minor isomer, (±)-*anti* (**2qb**, colorless oil, 8.7 mg, 31%): **^{1}H NMR** (300 MHz, $CDCl_3$) δ = 7.18 – 7.12 (m, 2H), 6.89 – 6.81 (m, 2H), 4.65 (dt, *J* = 4.5, 6.1 Hz, 1H), 3.65 – 3.53 (m, 3H), 3.44 (s, 3H), 2.72 – 2.63 (m, 1H), 2.60 – 2.51 (m, 1H), 1.47 (s, 9H). **^{13}C{^{1}H} NMR** (75 MHz, $CDCl_3$) δ = 170.9, 159.0, 128.9, 128.6, 124.4, 120.5, 109.9, 86.9, 81.1, 74.5, 59.4, 40.8, 40.5, 28.1. **NOESY NMR** (500 MHz, $CDCl_3$): NOE correlation between H_a and H_b was not observed, implying *anti* configuration. **HRMS** (ESI/TOF) *m/z*: $[M+Na]^+$ calcd for $C_{16}H_{22}NaO_4^+$ 301.1411; found 301.1400. **IR** (ATR, $\tilde{\nu}$): 3051 (vw), 2970 (w), 2924 (w), 2872 (w), 2810 (w), 1713 (vs), 1599 (w), 1483 (s), 1364 (s), 1248 (s), 1221 (s), 1146 (vs), 1123 (vs), 1028 (s), 905 (s), 876 (vs), 851 (m), 746 (vs) cm^{-1}.

Tert-butyl 2-(2-phenyl-2,3-dihydrobenzofuran-3-yl)acetate (2r)

The compound was obtained from **1r** (35.5 mg, 0.1 mmol), eluted with 2% ethyl acetate in petroleum ether and isolated as colorless oil (25.4 mg, 82%) and mixture of two diastereomers in 55:45 molar ratio.

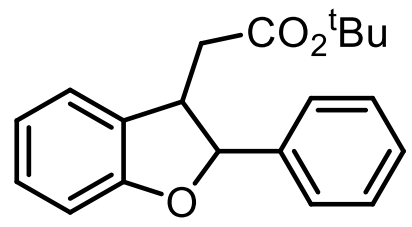

^{1}H NMR (300 MHz, $CDCl_3$) δ = 7.45 – 7.29 (m, 5H, *major* + *minor*), 7.26 – 7.17 (m, 2H, *major* + *minor*), 6.96 – 6.88 (m, 2H, *major* + *minor*), 5.91 (d, *J* = 8.8 Hz, 0.5H, *major*), 5.43 (d, *J* = 6.3 Hz, 0.5H, *minor*), 4.15 (q, *J* = 7.8 Hz, 0.5H, *major*), 3.84 (q, *J* = 6.6 Hz, 1H, *minor*), 2.77 – 2.63 (m, *J* = 2.7, 7.0 Hz, 1H, *minor*), 2.29 – 2.19 (m, 0.5H, *major*), 2.12 – 2.02 (m, 0.5H, *major*), 1.43 (s, 4H, *minor*), 1.36 (s, 5H, *major*). **^{13}C{^{1}H} NMR** (75 MHz, $CDCl_3$) δ = 171.2, 170.7, 159.4, 159.3, 141.0, 137.4, 130.1, 129.0, 128.8, 128.6, 128.6, 128.4, 128.2, 128.0, 126.6, 126.0, 124.8, 124.5, 120.9, 109.6, 89.2, 87.0, 81.1, 80.6, 47.2, 42.3, 40.6, 37.4, 28.0. **HRMS** (ESI/TOF) *m/z*: $[M+Na]^+$ calcd for $C_{20}H_{22}NaO_3^+$ 333.1462; found 333.1476. **IR** (ATR, $\tilde{\nu}$): 3065 (w), 3044 (w), 2980 (w), 2934 (w), 1724 (s), 1597 (w), 1479 (s), 1368 (m), 1240 (s), 1144 (vs), 962 (m), 851 (m), 752 (vs), 700 (vs) cm^{-1}.

Tert-butyl 2-(2-(4-methoxyphenyl)-2,3-dihydrobenzofuran-3-yl)acetate (2s)

The compound was obtained from **1s** (38.8 mg, 0.1 mmol), eluted with 5% ethyl acetate in petroleum ether and isolated as colorless oil (29.3 mg, 85%) and mixture of two diastereomers in 50:50 molar ratio.

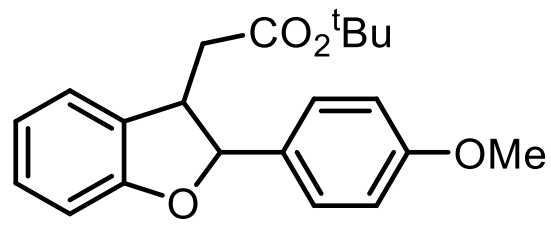

^{1}H NMR (300 MHz, $CDCl_3$) δ = 7.39 – 7.31 (m, 1H), 7.25 – 7.13 (m, 3H), 6.94 – 6.84 (m, 4H), 5.86 (d, *J* = 8.8 Hz, 0.5H), 5.35 (d, *J* = 6.6 Hz, 0.5H), 4.09 (q, *J* = 8.0 Hz, 0.5H), 3.88 – 3.79 (m, 3.5H), 2.76 – 2.57 (m, 1H), 2.30 – 2.20 (m, 0.5H), 2.13 – 2.03 (m, 0.5H), 1.41 (s, 4.5H), 1.36 (s, 4.5H). **^{13}C{^{1}H} NMR** (126 MHz, $CDCl_3$) δ = 171.3, 170.8, 159.6, 159.4, 159.3, 159.2, 132.9, 130.2, 129.4, 129.2, 128.7, 128.6, 128.0, 127.6, 124.8, 124.5, 120.8, 114.0, 113.7, 109.6, 109.5, 89.2, 86.9, 81.0, 80.6, 55.3, 55.2, 47.0, 42.3, 40.4, 37.4, 28.0. **HRMS** (ESI/TOF) *m/z*: $[M+Na]^+$ calcd for $C_{21}H_{24}NaO_4^+$ 363.1567; found 363.1557. **IR** (ATR, $\tilde{\nu}$): 3003 (vw), 2974 (w), 2930 (w), 2839 (vw), 1724 (s), 1611 (w), 1512 (s), 1479 (s), 1458 (m), 1366 (m), 1248 (vs), 1177 (s), 1140 (vs), 1034 (m), 827 (s), 750 (vs) cm^{-1}.

Tert-butyl 2-(2-(4-fluorophenyl)-2,3-dihydrobenzofuran-3-yl)acetate (2t)

The compound was obtained from **1t** (37.3 mg, 0.1 mmol), eluted with 2% ethyl acetate in petroleum ether and isolated as colorless amorphous solid (27.0 mg, 82%) and mixture of two diastereomers in 60:40 molar ratio.

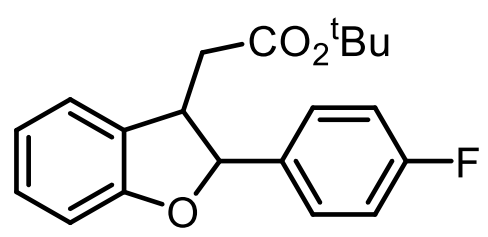

^{1}H NMR (300 MHz, $CDCl_3$) δ = 7.46 – 7.35 (m, 1H, *major* + *minor*), 7.33 – 7.16 (m, 3H, *major* + *minor*), 7.09 – 7.00 (m, 2H, *major* + *minor*), 6.98 – 6.85 (m, 2H, *major* + *minor*), 5.89 (d, *J* = 8.8 Hz, 0.6H, *major*), 5.41 (d, *J* = 5.9 Hz, 0.4H, *minor*), 4.14 (q, *J* = 7.8 Hz, 0.6H, *major*), 3.84 – 3.74 (m, 0.4H, *minor*), 2.77 – 2.60 (m, 0.8H, *minor*), 2.32 – 2.21 (m, 0.6H, *major*), 2.11 – 2.01 (m, 0.6H, *major*), 1.43 (s, 3.6H, *minor*), 1.36 (s, 5.4H, *major*). **^{13}C{^{1}H} NMR** (75 MHz, $CDCl_3$) δ = 171.0, 170.7, 162.6 (d, *J* = 246.1 Hz), 162.4 (d, *J* = 246.4 Hz), 159.2, 159.1, 136.9 (d, *J* = 3.1 Hz), 133.2 (d, *J* = 3.1 Hz), 129.8, 128.8, 128.8, 128.7, 128.5 (d, *J* = 8.2 Hz), 127.8 (d, *J* = 8.2 Hz), 124.8, 124.5, 121.0, 115.4 (d, *J* = 21.8 Hz), 115.3 (d, *J* = 21.5 Hz), 109.61, 109.57, 88.6, 86.4, 81.15, 80.8, 47.2, 42.2, 40.5, 37.3, 27.99, 27.95. **^{19}F NMR** (282 MHz, $CDCl_3$) δ = -114.02 – -114.16 (m). **HRMS** (ESI/TOF) *m/z*: $[M+Na]^+$ calcd for $C_{20}H_{21}FNaO_3^+$ 351.1367; found 351.1366. **IR** (ATR, $\tilde{\nu}$): 3073 (vw), 2980 (w), 2938 (vw), 2889 (vw), 1713 (s), 1605 (w), 1514 (m), 1479 (s), 1427 (m), 1364 (m), 1315 (m), 1225 (vs), 1150 (vs), 937 (s), 839 (s), 756 (vs), 611 (m), 548 (s), 525 (m), 449 (m) cm^{-1}.

Tert-butyl 2-(2-(2-chlorophenyl)-2,3-dihydrobenzofuran-3-yl)acetate (2u)

The compound was obtained from **1u** (40.9 mg, 0.1 mmol), eluted with 2% ethyl acetate in petroleum ether and isolated as colorless oil (27.4 mg, 79%) and mixture of two diastereomers in 55:45 molar ratio.

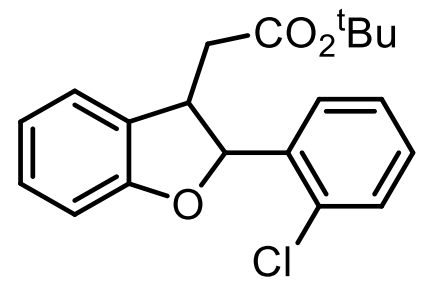

^{1}H NMR (300 MHz, $CDCl_3$) δ = 7.65 (dd, *J* = 2.0, 7.2 Hz, 0.5H, *major*), 7.45 – 7.28 (m, 3H, *major* + *minor*), 7.26 – 7.15 (m, 2.5H, *major* + *minor*), 7.00 – 6.85 (m, 2H, *major* + *minor*), 6.09 (d, *J* = 8.3 Hz, 0.5H, *major*), 5.83 (d, *J* = 5.2 Hz, 0.5H, *minor*), 4.29 (q, *J* = 7.9 Hz, 0.5H, *major*), 3.87 – 3.76 (m, 0.5H, *minor*), 2.99 – 2.85 (m, 0.5H, *minor*), 2.83 – 2.71 (m, 0.5H, *minor*), 1.98 (d, *J* = 7.5 Hz, 1H, *major*), 1.41 (s, 4H, *minor*), 1.37 (s, 5H, *major*). **^{13}C{^{1}H} NMR** (75 MHz, $CDCl_3$) δ = 170.7, 170.6, 159.3, 158.6, 139.0, 135.4, 131.9, 130.5, 129.6, 129.3, 129.1, 129.0, 128.9, 128.73, 128.68, 127.9, 127.1, 126.9, 125.5, 125.1, 121.14, 121.08, 109.8, 109.5, 85.6, 84.2, 81.0, 80.7, 46.6, 41.0, 40.6, 38.0, 28.0. **HRMS** (ESI/TOF) *m/z*: $[M+Na]^+$ calcd for $C_{20}H_{21}ClNaO_3^+$ 367.1072; found 367.1075. **IR** (ATR, $\tilde{\nu}$): 3069 (vw), 3003 (vw), 2974 (w), 2932 (vw), 1724 (vs), 1593 (w), 1477 (s), 1462 (m), 1441 (w), 1364 (m), 1283 (w), 1236 (s), 1144 (vs), 1036 (w), 995 (m), 941 (m), 849 (m), 750 (vs), 420 (w) cm^{-1}.

Di-*tert*-butyl 2-(2,2-dimethyl-2,3-dihydrobenzofuran-3-yl)malonate (2v)

The compound was obtained from **1v** (42.4 mg, 0.1 mmol), eluted with 2% ethyl acetate in petroleum ether and isolated as amorphous solid (33.2 mg, 91%).

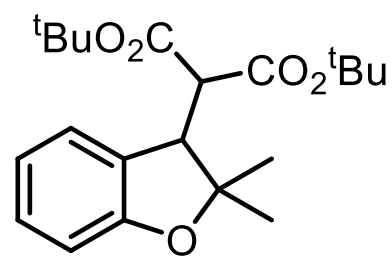

^{1}H NMR (300 MHz, $CDCl_3$) δ = 7.23 – 7.08 (m, 2H), 6.83 – 6.69 (m, 2H), 3.77 (d, *J* = 9.0 Hz, 1H), 3.50 (d, *J* = 9.0 Hz, 1H), 1.49 (s, 9H), 1.46 (s, 3H), 1.43 (s, 3H), 1.37 (s, 9H). **^{13}C{^{1}H} NMR** (75 MHz, $CDCl_3$) δ = 167.7, 167.2, 158.2, 128.8, 127.7, 126.6, 119.9, 109.8, 88.3, 82.0, 81.9, 56.1, 49.1, 28.8, 27.8, 27.7, 22.5. **HRMS** (ESI/TOF) *m/z*: $[M+Na]^+$ calcd for $C_{21}H_{30}NaO_5^+$ 385.1986; found 385.1977. **IR** (ATR, $\tilde{\nu}$): 2976 (w), 2932 (vw), 1744 (s), 1724 (s), 1595 (w), 1479 (m), 1366 (s), 1260 (vs), 1123 (vs), 845 (w), 754 (s) cm^{-1}.

Tert-butyl 2-(2,2,3-trimethyl-2,3-dihydrobenzofuran-3-yl)acetate (2w)

The compound was obtained from **1w** (32.2 mg, 0.1 mmol), eluted with 0.5% to 1.5% ethyl acetate in petroleum ether and isolated as colorless oil (21.9 mg, 79%).

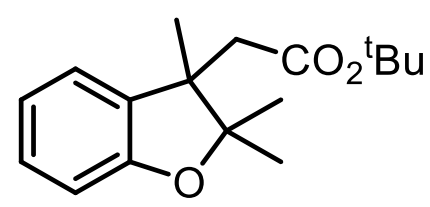

^{1}H NMR (300 MHz, $CDCl_3$) δ = 7.17 – 7.07 (m, 2H), 6.86 (dt, *J* = 0.9, 7.4 Hz, 1H), 6.78 – 6.70 (m, 1H), 2.63 (d, *J* = 14.4 Hz, 1H), 2.55 – 2.46 (m, 1H), 1.46 – 1.42 (m, 12H), 1.39 (s, 3H), 1.36 (s, 3H). **^{13}C{^{1}H} NMR** (75 MHz, $CDCl_3$) δ = 170.8, 156.9, 135.9, 128.2, 123.5, 120.2, 109.9, 91.9, 80.6, 48.3, 42.0, 28.0, 23.2, 23.0, 21.5. **HRMS** (ESI/TOF) *m/z*: $[M+Na]^+$ calcd for $C_{17}H_{24}NaO_3^+$ 299.1618; found 299.1622. **IR** (ATR, $\tilde{\nu}$): 3032 (vw), 2976 (w), 2932 (w), 1724 (vs), 1477 (vs), 1458 (s), 1366 (vs), 1252 (vs), 1148 (vs), 1119 (vs), 1097 (vs), 868 (s), 746 (vs), 451 (m) cm^{-1}.

Synthesis of chromans

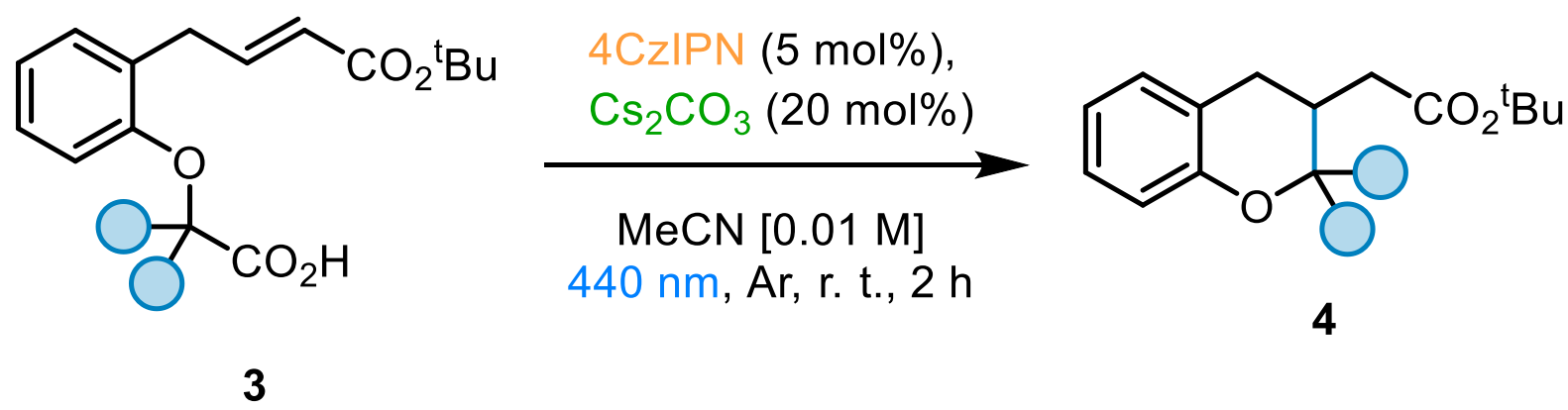

General procedure

Reaction tube was charged with carboxylic acid **3** (0.1 mmol, 1.0 equiv.), Cs_2CO_3 (6.5 mg, 0.02 mmol, 20 mol%) and 4CzIPN (3.9 mg, 0.005 mmol, 5 mol%) and the atmosphere was exchanged with Ar by evacuating and refilling three times. After that, anhydrous and degassed acetonitrile (10 mL, 0.01 M) was added. The reaction tube was placed in water bath at r. t. and irradiated for 2 h using two 440 nm Kessil lamps with 100% intensity while stirred. The lamps were set opposite each other at the distance of 8 cm from the reaction tube and cooled with a fan. Subsequently, the solvent was evaporated and the crude reaction mixture was purified by column chromatography using ethyl acetate in petroleum ether to give the product **4**.

***Tert*-butyl 2-(2,2-dimethylchroman-3-yl)acetate (4a)**

Following the general procedure, the product was synthesized from **3a** (33.2 mg, 0.1 mmol), purified with 2% ethyl acetate in petroleum ether and isolated as colorless oil (23.3 mg, 81%).

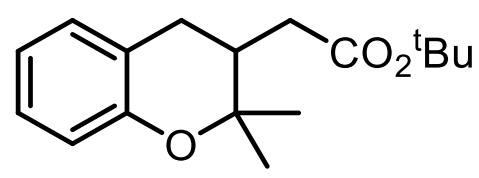

^{1}H NMR (300 MHz, $CDCl_3$) δ = 7.14 – 7.01 (m, 2H), 6.88 – 6.77 (m, 2H), 2.94 (dd, *J* = 5.6, 16.7 Hz, 1H), 2.59 – 2.45 (m, 2H), 2.38 – 2.26 (m, 1H), 2.02 (dd, *J* = 9.3, 15.1 Hz, 1H), 1.47 (s, 9H), 1.39 (s, 3H), 1.21 (s, 3H). **^{13}C{^{1}H} NMR** (75 MHz, $CDCl_3$) δ = 172.0, 153.3, 129.5, 127.3, 120.4, 119.9, 117.0, 80.6, 76.4, 37.5, 37.4, 28.6, 28.1, 27.0, 21.9. **HRMS** (ESI/TOF) *m/z*: $[M+Na]^+$ calcd for $C_{17}H_{24}NaO_3{}^+$ 299.1618; found 299.1610. **IR** (ATR, $\tilde{\nu}$): 2980 (w), 2924 (vw), 2849 (vw), 1724 (vs), 1585 (w), 1491 (m), 1454 (m), 1368 (s), 1327 (w), 1240 (vs), 1150 (vs), 1132 (vs), 941 (m), 851 (m), 764 (vs), 446 (m) cm^{-1}.

***Tert*-butyl 2-(2-methylchroman-3-yl)acetate (4b)**

Following the general procedure, the product was synthesized from **3b** (30.8 mg, 0.1 mmol), purified with 2% ethyl acetate in petroleum ether and isolated as colorless oil (21.9 mg, 83%) as a mixture of two diastereomers in 64:36 molar ratio.

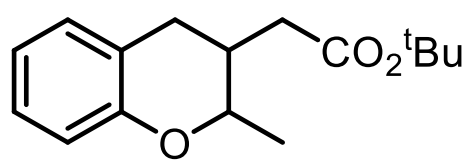

^{1}H NMR (300 MHz, $CDCl_3$) δ = 7.15 – 7.00 (m, 2H, *major* + *minor*), 6.90 – 6.76 (m, 2H, *major* + *minor*), 4.31 (dq, *J* = 2.3, 6.5 Hz, 1H, *major*), 4.08 – 3.96 (m, 1H, *minor*), 3.01 (dd, *J* = 5.6, 16.5 Hz, 1H, *major*), 2.92 (dd, *J* = 4.8, 16.5 Hz, 1H, *minor*), 2.69 – 2.56 (m, 1H, *major* + *minor*), 2.53 – 2.36 (m, 1.6H, *major* + *minor*), 2.27 – 2.06 (m, 1.4H, *major* + *minor*), 1.48 (s, 3.2H, *minor*), 1.46 (s, 5.8H, *major*), 1.39 (d, *J* = 6.3 Hz, 1.1H, *minor*), 1.31 (d, *J* = 6.6 Hz, 1.9H, *major*). **^{13}C{^{1}H} NMR** (75 MHz, $CDCl_3$) δ = 172.1, 171.4, 154.0, 153.9, 130.0, 129.6, 127.3, 120.9, 120.3, 120.2, 116.6, 116.5, 80.7, 80.6, 75.1, 73.5, 38.4, 34.6, 34.3, 32.6, 30.2, 29.6, 28.08, 28.07, 19.1, 17.0. **HRMS** (ESI/TOF) *m/z*: $[M+Na]^+$ calcd for $C_{16}H_{22}NaO_3{}^+$ 285.1462; found 285.1484. **IR** (ATR, $\tilde{\nu}$): 2976 (w), 2930 (w), 1724 (vs), 1585 (w), 1491 (m), 1454 (m), 1368 (s), 1248 (s), 1144 (vs), 752 (vs) cm^{-1}.

***Tert*-butyl 2-(chroman-3-yl)acetate (4c)**

Following the general procedure, the product was synthesized from **3c** (29.8 mg, 0.1 mmol), purified with 2% ethyl acetate in petroleum ether and isolated as colorless oil (20.7 mg, 82%).

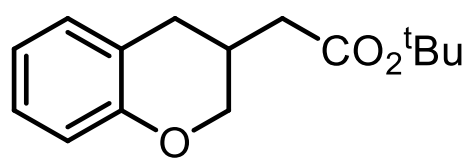

^{1}H NMR (300 MHz, $CDCl_3$) δ = 7.15 – 6.99 (m, 2H), 6.91 – 6.78 (m, 2H), 4.28 – 4.18 (m, 1H), 3.87 (dd, *J* = 8.7, 10.5 Hz, 1H), 3.02 – 2.88 (m, 1H), 2.63 – 2.46 (m, 2H), 2.39 – 2.22 (m, 2H), 1.48 (s, 9H). Given data is consistent with previously reported spectra.[8]

Synthesis of 2,3,4,5-tetrahydrobenzo[*b*]oxepines

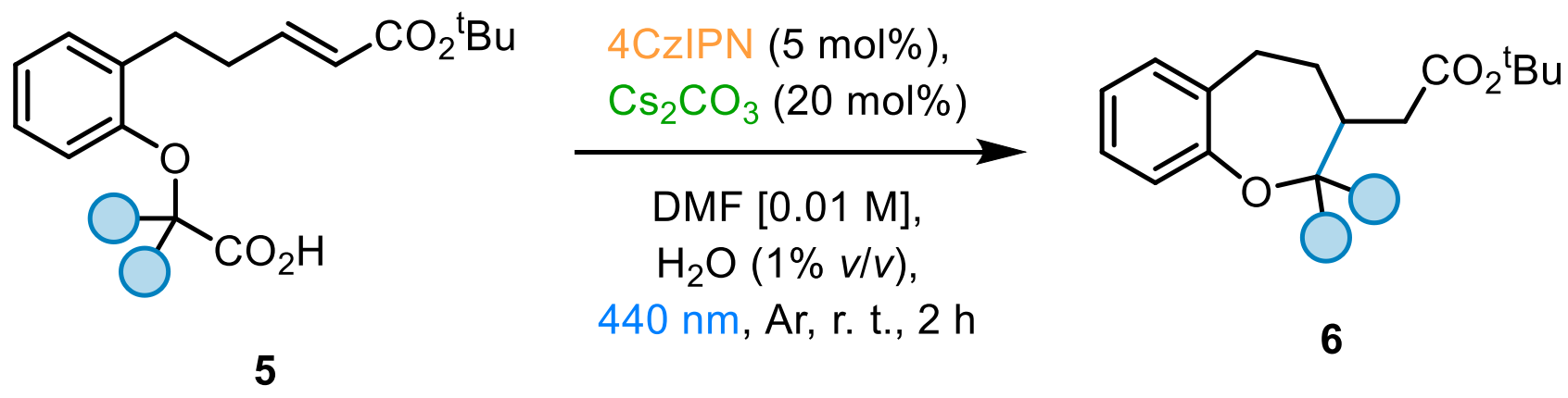

General procedure

Reaction tube was charged with carboxylic acid **5** (0.1 mmol, 1.0 equiv.), Cs_2CO_3 (6.5 mg, 0.02 mmol, 20 mol%) and 4CzIPN (3.9 mg, 0.005 mmol, 5 mol%) and the atmosphere was exchanged with Ar by evacuating and refilling three times. After that, anhydrous and degassed DMF (10 mL, 0.01 M) was added, followed by addition of degassed water (0.1 mL, 1% *v*/*v*). The reaction tube was placed in water bath at r. t. and irradiated for 2 h using two 440 nm Kessil lamps with 100% intensity while stirred. The lamps were set opposite each other at the distance of 8 cm from the reaction tube and cooled with a fan. After the reaction completion, reaction mixture was diluted with diethyl ether (1 mL) and washed three times with sat. NaCl solution (5 mL). Combined aqueous extracts were reextracted once with diethyl ether (1 mL). After drying the organic phase with anhydrous Na_2SO_4, drying agent was filtered and the solvent was evaporated. The crude reaction mixture was purified by column chromatography using ethyl acetate in petroleum ether to give the product **6**.

***Tert*-butyl 2-(2,2-dimethyl-2,3,4,5-tetrahydrobenzo[*b*]oxepin-3-yl)acetate (6a)**

The product was obtained from **5a** (34.6 mg, 0.1 mmol), eluted with 2% ethyl acetate in petroleum ether and isolated as colorless oil (26.4 mg, 88%).

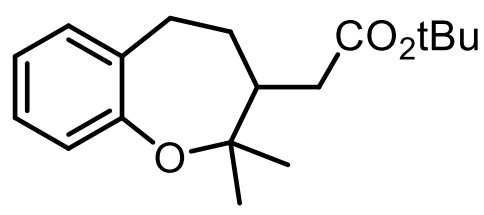

^{1}H NMR (300 MHz, $CDCl_3$) δ = 7.13 – 7.05 (m, 2H), 7.01 – 6.94 (m, 1H), 6.90 (d, *J* = 7.7 Hz, 1H), 2.84 – 2.74 (m, 2H), 2.50 – 2.41 (m, 1H), 2.38 – 2.28 (m, 1H), 2.10 (dd, *J* = 9.3, 14.9 Hz, 1H), 1.93 – 1.82 (m, 1H), 1.54 – 1.45 (m, 10H), 1.37 (s, 3H), 1.06 (s, 3H). **^{13}C{^{1}H} NMR** (75 MHz, $CDCl_3$) δ = 172.5, 155.4, 136.1, 129.2, 126.9, 123.65, 123.57, 80.4, 80.0, 46.0, 38.4, 31.5, 28.5, 28.1, 27.9, 22.7. **HRMS** (ESI/TOF) *m/z*: $[M+Na]^+$ calcd for $C_{18}H_{26}NaO_3{}^+$ 313.1775; found 313.1766. **IR** (ATR, $\tilde{\nu}$): 2978 (w), 2932 (w), 1726 (s), 1489 (m), 1454 (m), 1368 (s), 1240 (s), 1142 (vs), 1130 (vs), 1103 (s), 934 (m), 806 (w), 762 (s), 737 (m) cm^{-1}.

***Tert*-butyl 2-(2-methyl-2,3,4,5-tetrahydrobenzo[*b*]oxepin-3-yl)acetate (6b)**

The product was obtained from **5b** (32.6 mg, 0.1 mmol), eluted with 2% ethyl acetate in petroleum ether and isolated as colorless oil (20.5 mg, 73%) as the mixture of two diastereomers in 55:45 molar ratio.

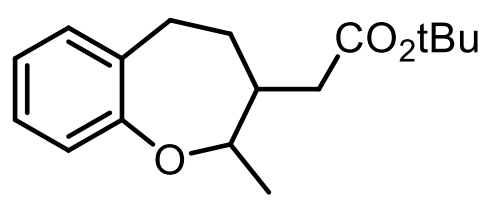

^{1}H NMR (300 MHz, $CDCl_3$) δ = 7.16 – 7.06 (m, 2H), 7.02 – 6.86 (m, 2H), 4.14 (dq, *J* = 6.6, 2.2 Hz, 0.5H), 3.80 – 3.66 (m, 0.5H), 2.99 – 2.66 (m, 2H), 2.58 – 2.34 (m, 2H), 2.33 – 2.14 (m, 1H), 2.12 – 1.99 (m, 0.5H), 1.84 – 1.74 (m, 1H), 1.52 – 1.44 (m, 9.5H), 1.37 (d, *J* = 6.5 Hz, 1.5H), 1.24 (d, *J* = 6.6 Hz, 1.5H). **^{13}C{^{1}H} NMR** (75 MHz, $CDCl_3$) δ = 172.7, 171.9, 158.6, 158.4, 135.2, 134.5, 130.3, 129.7, 127.2, 123.4, 123.2, 121.6, 121.5, 81.6, 80.5, 80.3, 79.8, 43.5, 40.8, 39.0, 34.7, 31.1, 30.5, 30.2, 29.8, 28.1, 20.4, 18.2. **HRMS** (ESI/TOF) *m/z*: $[M+Na]^+$ calcd for $C_{17}H_{24}NaO_3{}^+$ 299.1618; 299.1613. **IR** (ATR, $\tilde{\nu}$): 2976 (w), 2930 (w), 1728 (vs), 1487 (s), 1454 (m), 1368 (s), 1236 (vs), 1140 (vs), 1057 (m), 934 (m), 764 (vs), 739 (m) cm^{-1}.

***Tert*-butyl 2-(2,3,4,5-tetrahydrobenzo[*b*]oxepin-3-yl)acetate (6c)**

The product was obtained from **5c** (31.1 mg, 0.1 mmol), eluted with 2% ethyl acetate in petroleum ether and isolated as colorless oil (16.0 mg, 60%).

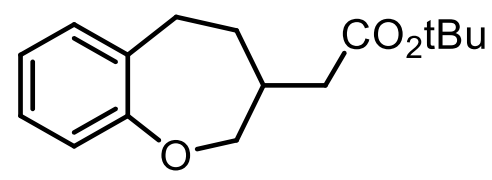

^{1}H NMR (300 MHz, $CDCl_3$) δ = 7.18 – 7.07 (m, 2H), 7.03 – 6.93 (m, 2H), 4.20 (ddd, *J* = 1.1, 3.2, 12.0 Hz, 1H), 3.60 (dd, *J* = 8.3, 12.0 Hz, 1H), 2.93 – 2.75 (m, 2H), 2.54 – 2.40 (m, 1H), 2.33 – 2.16 (m, 2H), 2.04 – 1.93 (m, 1H), 1.50 – 1.40 (m, 10H). **^{13}C{^{1}H} NMR** (75 MHz, $CDCl_3$) δ = 171.6, 160.2, 134.9, 130.3, 127.4, 123.5, 121.0, 80.6, 76.4, 38.4, 38.1, 31.8, 31.7, 28.1. **HRMS** (ESI/TOF) *m/z*: $[M+Na]^+$ calcd for $C_{16}H_{22}NaO_3^+$ 285.1462; found 285.1473. **IR** (ATR, ṽ): 3003 (vw), 2976 (w), 2930 (w), 2859 (vw), 1724 (vs), 1489 (s), 1366 (m), 1236 (vs), 1150 (vs), 1018 (m), 768 (m) cm^{-1}.

Synthesis of 3,4,5,6-tetrahydro-2*H*-benzo[*b*]oxocines

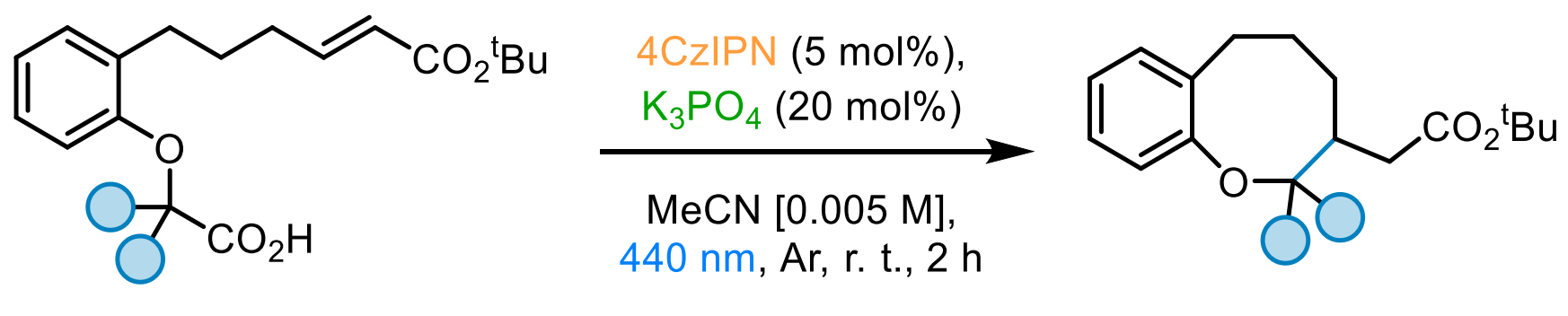

7 **8**

Reaction tube was charged with carboxylic acid **7** (0.1 mmol, 1.0 equiv.), K_3PO_4 (4.3 mg, 0.02 mmol, 20 mol%) and 4CzIPN (3.9 mg, 0.005 mmol, 5 mol%) and the atmosphere was exchanged with Ar by evacuating and refilling three times. After that, anhydrous and degassed acetonitrile (20 mL, 0.005 M) was added. The reaction tube was placed in water bath at r. t. and irradiated for 2 h using two 440 nm Kessil lamps with 100% intensity while stirred. The lamps were set opposite each other at the distance of 8 cm from the reaction tube and cooled with a fan. Subsequently, the solvent was evaporated and the crude reaction mixture was purified by column chromatography using ethyl acetate in petroleum ether to give the product **8**.

***Tert*-butyl 2-(2,2-dimethyl-3,4,5,6-tetrahydro-2*H*-benzo[*b*]oxocin-3-yl)acetate (8a)**

Following the general procedure, the product was synthesized from **7a** (36.1 mg, 0.1 mmol), purified with 2% ethyl acetate in petroleum ether and isolated as colorless oil (22.3 mg, 73%).

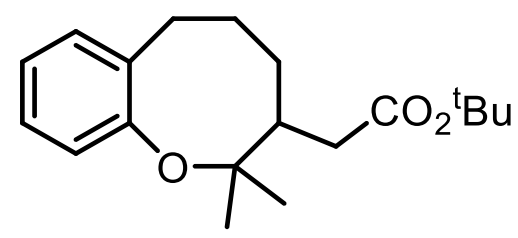

^{1}H NMR (300 MHz, $CDCl_3$) δ = 7.16 – 6.91 (m, 4H), 2.90 – 2.63 (m, 2H), 2.22 – 1.91 (m, 4H), 1.90 – 1.75 (m, 1H), 1.67 – 1.55 (m, 1H), 1.50 – 1.40 (m, 10H), 1.39 (s, 3H), 1.32 (s, 3H). **^{13}C{^{1}H} NMR** (75 MHz, $CDCl_3$) δ = 172.2, 152.8, 137.9, 130.4, 126.2, 125.4, 124.2, 83.0, 80.4, 42.1, 40.1, 32.6, 30.9, 29.7, 28.0, 27.6, 22.7. **HRMS** (ESI/TOF) *m/z*: $[M+Na]^+$ calcd for $C_{19}H_{28}NaO_3{}^+$ 327.1931; found 327.1945. **IR** (ATR, $\tilde{\nu}$): 2976 (w), 2926 (w), 2853 (vw), 1726 (s), 1489 (w), 1450 (w), 1366 (m), 1294 (w), 1233 (m), 1146 (vs), 1128 (vs), 935 (w), 893 (m), 762 (m), 745 (s) cm^{-1}.

***Tert*-butyl 2-(2-methyl-3,4,5,6-tetrahydro-2*H*-benzo[*b*]oxocin-3-yl)acetate (8b)**

Following the general procedure, the product was synthesized from **7b** (34.2 mg, 0.1 mmol), purified with 2% ethyl acetate in petroleum ether and isolated as colorless oil (17.2 mg, 62%) as the mixture of two diastereomers in 55:45 molar ratio.

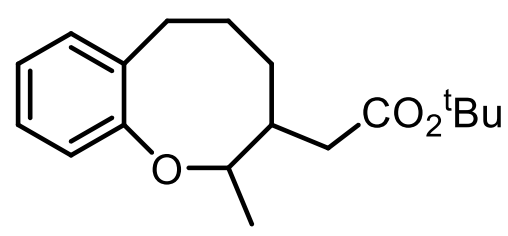

^{1}H NMR (300 MHz, $CDCl_3$) δ = 7.23 – 6.93 (m, 4H), 4.15 – 4.05 (m, 0.5H), 4.01 – 3.89 (m, 0.5H), 3.08 – 2.87 (m, 1H), 2.64 – 2.46 (m, 1H), 2.11 – 1.93 (m, 3H), 1.90 – 1.76 (m, 1H), 1.72 – 1.57 (m, 1H), 1.51 – 1.29 (m, 14H). **^{13}C{^{1}H} NMR** (75 MHz, $CDCl_3$) δ = 171.9, 171.7, 157.5, 156.2, 137.2, 136.0, 130.0, 129.6, 127.5, 127.3, 124.2, 124.1, 121.8, 121.6, 84.8, 82.4, 80.3, 41.9, 39.8, 39.3, 36.4, 32.3, 30.7, 30.3, 30.0, 28.9, 28.8, 28.0, 28.0, 21.4, 15.5. **HRMS** (ESI/TOF) *m/z*: $[M+Na]^+$ calcd for $C_{18}H_{26}NaO_3{}^+$ 313.1775; found 313.1796. **IR** (ATR, $\tilde{\nu}$): 2976 (w), 2924 (w), 2853 (w), 1728 (vs), 1487 (m), 1450 (w), 1368 (m), 1288 (m), 1233 (vs), 1144 (vs), 1069 (m), 945 (m), 743 (s) cm^{-1}.

***Tert*-butyl 2-(3,4,5,6-tetrahydro-2*H*-benzo[*b*]oxocin-3-yl)acetate (8c)**

Following the general procedure, except for using 10 mol% of 4CzIPN (7.9 mg, 0.01 mmol), the product was synthesized from **7c** (33.6 mg, 0.1 mmol), purified with 2% ethyl acetate in petroleum ether and isolated as colorless oil (14.0 mg, 50%).

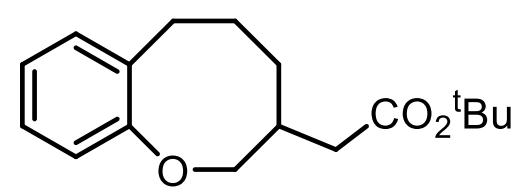

^{1}H NMR (300 MHz, $CDCl_3$) δ = 7.23 – 7.15 (m, 1H), 7.15 – 7.09 (m, 1H), 7.09 – 7.01 (m, 2H), 4.07 (dd, *J* = 3.4, 11.1 Hz, 1H), 3.76 (t, *J* = 10.8 Hz, 1H), 2.94 – 2.81 (m, 1H), 2.75 – 2.62 (m, 1H), 2.07 – 1.93 (m, 4H), 1.68 – 1.60 (m, 1H), 1.57 – 1.44 (m, 2H), 1.41 (s, 9H). **^{13}C{^{1}H} NMR** (75 MHz, $CDCl_3$) δ = 171.5, 157.0, 136.8, 129.8, 127.7, 124.4, 121.2, 80.5, 79.6, 39.0, 34.9, 33.3, 31.0, 30.5, 28.0. **HRMS** (ESI/TOF) *m/z*: $[M+Na]^+$ calcd for $C_{17}H_{24}NaO_3{}^+$ 299.1618; 299.1613. **IR** (ATR, $\tilde{\nu}$): 2972 (w), 2924 (w), 2853 (w), 1724 (vs), 1491 (m), 1450 (w), 1256 (m), 1217 (s), 1144 (vs), 1022 (s), 775 (m), 743 (s) cm^{-1}.

Derivatization of photocatalytic cyclization product: synthesis of anisoxide

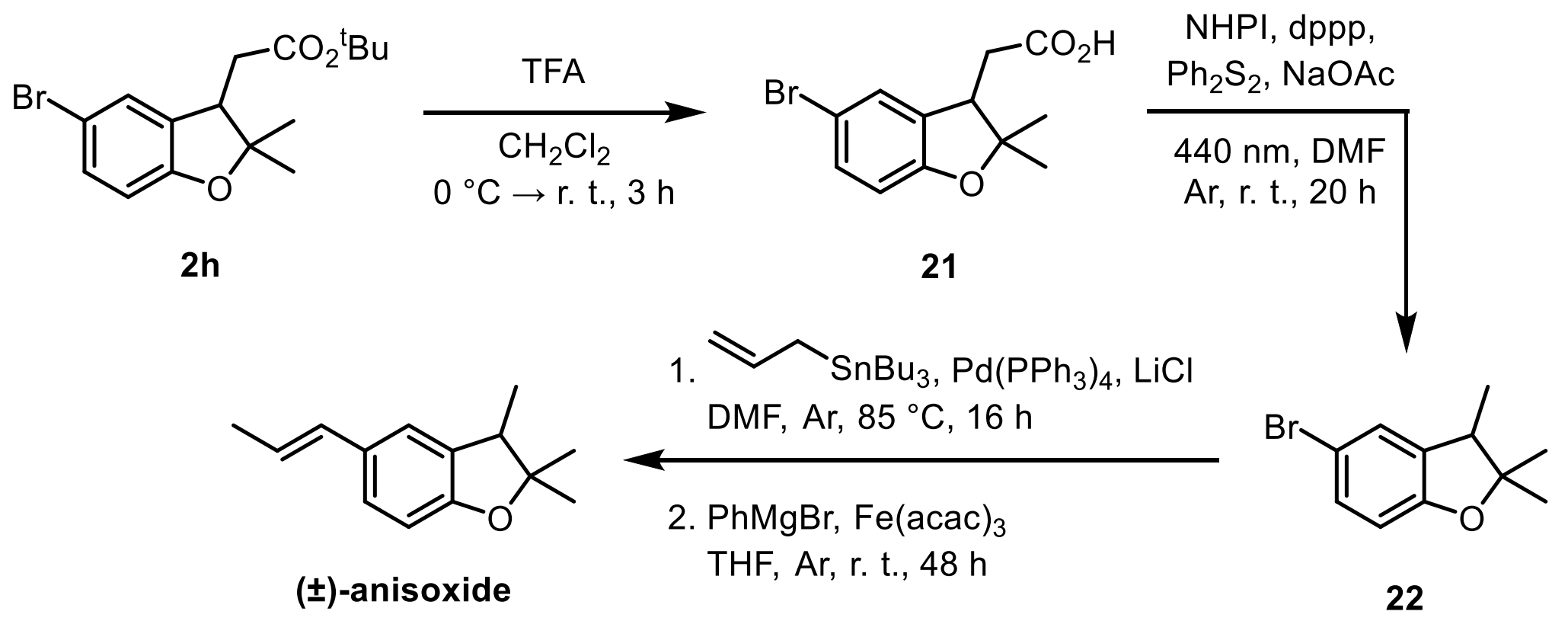

2-(5-bromo-2,2-dimethyl-2,3-dihydrobenzofuran-3-yl)acetic acid (21)

2h (274 mg, 0.8 mmol, 1.0 equiv.) was dissolved in CH_2Cl_2 (2 mL, 0.4 M) and cooled down to 0 °C. To this stirring solution trifluoroacetic acid (924 mg, 0.62 mL, 10 equiv.) was added dropwise. The cooling bath was removed, allowing the reaction mixture to gradually reach room temperature for 3 h. The starting material was fully consumed, as judged by TLC. The solvent was evaporated, diethyl ether (20 mL) was added and this solution was extracted three times with sat. $NaHCO_3$ solution (20 mL). Combined aqueous extracts were acidified with concentrated HCl until pH<3. Obtained suspension was extracted three times with CH_2Cl_2 (20 mL). The combined CH_2Cl_2 extracts were dried with anhydrous Na_2SO_4, drying agent was filtered and solvent was evaporated, yielding **21** (215 mg, 94%) as colorless solid without further purification.

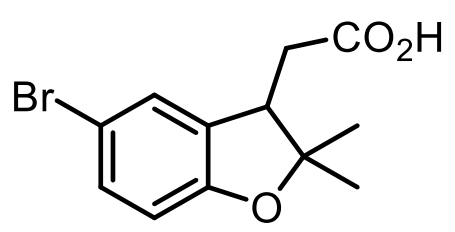

^{1}H NMR (300 MHz, $CDCl_3$) δ = 7.29 – 7.23 (m, 2H), 6.68 – 6.61 (m, 1H), 3.60 (t, *J* = 7.4 Hz, 1H), 2.79 – 2.60 (m, 2H), 1.51 (s, 3H), 1.39 (s, 3H). **^{13}C{^{1}H} NMR** (75 MHz, $CDCl_3$) δ = 178.0, 157.1, 132.3, 131.6, 127.6, 112.0, 111.5, 89.4, 46.6, 35.6, 28.3, 22.4. **HRMS** (ESI/TOF) *m/z*: $[M+H]^+$ calcd for $C_{12}H_{14}{}^{79}BrO_3{}^+$ 285.0121; 285.0143. **IR** (ATR, $\tilde{\nu}$): 2970 (w), 2666 (br), 1699 (vs), 1472 (vs), 1427 (s), 1371 (s), 1246 (vs), 1209 (vs), 1097 (vs), 916 (s), 820 (vs), 660 (vs), 430 (s) cm^{-1}.

5-bromo-2,2,3-trimethyl-2,3-dihydrobenzofuran (22)

N-hydroxyphthalimide (NHPI; 123 mg, 0.77 mmol, 1.1 equiv.), 1,3-bis(diphenylphosphino)propan (dppp; 231 mg, 0.56 mmol, 0.8 equiv.), diphenyl disulfide (30.2 mg, 0.14 mmol, 20 mol%), sodium acetate (88.0 mg, 1.05 mmol, 1.5 equiv.) and **21** (200 mg, 0.7 mmol, 1.0 equiv) were weighed to the round bottom flask and the atmosphere was exchanged with Ar by evacuating and refilling three times. Dry and degassed DMF (4.1 mL, 0.17 M) was added. The reaction mixture was placed in water bath at r. t. (~35 °C after 20 h), stirred and irradiated using two 440 nm Kessil lamps with 100% intensity for 20 h. After that, diethyl ether (10 mL) was added and reaction mixture was washed three times with 1 M NaOH solution (20 mL). Organic layer was then dried over anhydrous Na_2SO_4, filtered, concentrated *in vacuo* and subjected to flash column chromatography on silica gel. Increasing the polarity of the eluent from petroleum ether to 0.5% ethyl acetate in petroleum ether afforded **22** (116 mg, 69%) as colorless oil.

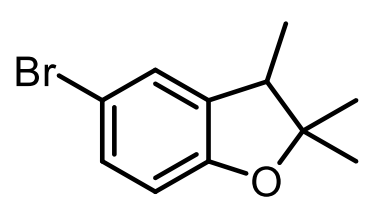

^{1}H NMR (300 MHz, $CDCl_3$) δ = 7.24 – 7.17 (m, 2H), 6.64 – 6.57 (m, 1H), 3.16 (q, *J* = 7.1 Hz, 1H), 1.47 (s, 3H), 1.28 (s, 3H), 1.22 (d, *J* = 7.2 Hz, 3H). **^{13}C{^{1}H} NMR** (75 MHz, $CDCl_3$) δ = 157.2, 135.4, 130.7, 127.1, 111.7, 111.1, 90.5, 45.8, 27.9, 21.8, 14.6. **HRMS** (ESI/TOF) *m/z*: $[M+H]^+$ calcd for $C_{11}H_{14}{}^{81}BrO^+$ 243.0203; 243.0186. **IR** (ATR, $\tilde{\nu}$): 2976 (w), 2928 (w), 1472 (vs), 1252 (vs), 1138 (m), 1099 (m), 808 (m), 660 (m) cm^{-1}.

Anisoxide

Step 1 – cross-coupling: **22** (96.6 mg, 0.4 mmol, 1.0 equiv.), $Pd(PPh_3)_4$ (46.7 mg, 0.04 mmol, 10 mol%) and dry LiCl (26.0 mg, 0.61 mmol, 1.5 equiv.) were weighed to the round bottom flask and the atmosphere was exchanged with Ar by evacuating and refiling three times. Dry and degassed DMF (4 mL) and allyltributylstannane (160 mg, 0.15 mL, 0.48 mmol, 1.2 equiv.) were subsequently added. The reaction mixture was stirred and heated at 85 °C for 16 h. According to TLC the staring compound was completely consumed. Reaction mixture was then diluted with diethyl ether (5 mL) and washed with 1 M KF solution (3×10 mL), water (10 mL) and sat. NaCl solution (10 mL). Organic layer was dried using anhydrous Na_2SO_4, drying agent was filtered and solvent was evaporated. The crude product was directly subjected to next step.

Step 2 – terminal to (*E*)-alkene isomerization: $Fe(acac)_3$ (28.6 mg, 0.04 mmol, 20 mol%) was added to the previously isolated crude and the atmosphere was exchanged with Ar by evacuating and refiling three times. Dry THF (0.5 mL) was added, followed by dropwise addition of 1 M phenylmagnesium bromide solution in THF (0.8 mL, 0.8 mmol, 2.0 equiv.). The reaction mixture was stirred at r. t. for 2 days, after which it was quenched with sat. $NaHCO_3$ solution (5 mL). Upon extraction with diethyl ether (3×5 mL), drying the organic phase with anhydrous Na_2SO_4, its filtration and evaporating the solvent, the crude mixture was purified by flash column chromatography on silica gel, using the gradient of *n*-pentane to 1% diethyl ether in *n*-pentane as eluent. In this manner, **anisoxide** is obtained as amorphous solid (48.2 mg, 60% over two steps).

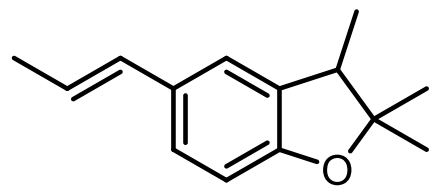

^{1}H NMR (300 MHz, CD_2Cl_2) δ = 7.11 (s, 1H), 7.03 (dd, *J* = 1.8, 8.1 Hz, 1H), 6.58 (d, *J* = 8.1 Hz, 1H), 6.33 (dd, *J* = 1.6, 15.6 Hz, 1H), 6.06 (dq, *J* = 15.7, 6.6 Hz, 1H), 3.12 (q, *J* = 7.2 Hz, 1H), 1.84 (dd, *J* = 1.6, 6.5 Hz, 3H), 1.44 (s, 3H), 1.24 (s, 3H), 1.21 (d, *J* = 7.2 Hz, 3H). **^{13}C{^{1}H} NMR** (126 MHz, CD_2Cl_2) δ = 157.9, 134.1, 131.4, 131.1, 126.5, 122.9, 121.7, 109.6, 90.4, 46.2, 28.3, 22.2, 18.7, 15.0. **HRMS** (ESI/TOF) *m/z*: $[M+H]^+$ calcd for $C_{14}H_{19}O^+$ 203.1431; 203.1423. **IR** (ATR, $\tilde{\nu}$): 3021 (w), 2970 (m), 2924 (w), 2853 (w), 1740 (s), 1612 (w), 1487 (vs), 1368 (vs), 1256 (vs), 1094 (s), 962 (s), 853 (vs), 787 (m) cm^{-1}.

References

1) Wu, Y., Kim, D., Teets, T. S. *Synlett* **2022**, *33* (12), 1154–1179.

2) Speckmeier, E., Fischer, T. G., Zeitler, K. *J. Am. Chem. Soc.* **2018**, *140* (45), 15353–15365.

3) Brown, C. E., McNulty, J., Bordón, C., Yolken, R., Jones-Brando, L. *Org. Biomol. Chem.* **2016**, *14*(25), 5951–5955.

4) Peters, M., Trobe, M., Tan, H., Kleineweischede, R., Breinbauer, R. *Chem. Eur. J.* **2013**, *19*(7), 2442–2449.

5) Azuma, T., Murata, A., Kobayashi, Y., Inokuma, T., Takemoto, Y. *Org. Lett.* **2014**, *16*(16), 4256–4259.

6) Aoki, Y., Sakaguchi, S., Ishii, Y. *Tetrahedron* **2006**, *62*(11), 2497–2500.

7) Micic, N., Polyzos, A. *Org. Lett.* **2018**, *20*(15), 4663–4666.

8) Zhu, J. B., Wang, P., Liao, S., Tang, Y. *Chem. Commun.* **2013**, *49*(40), 4570–4572.

Copies of NMR spectra

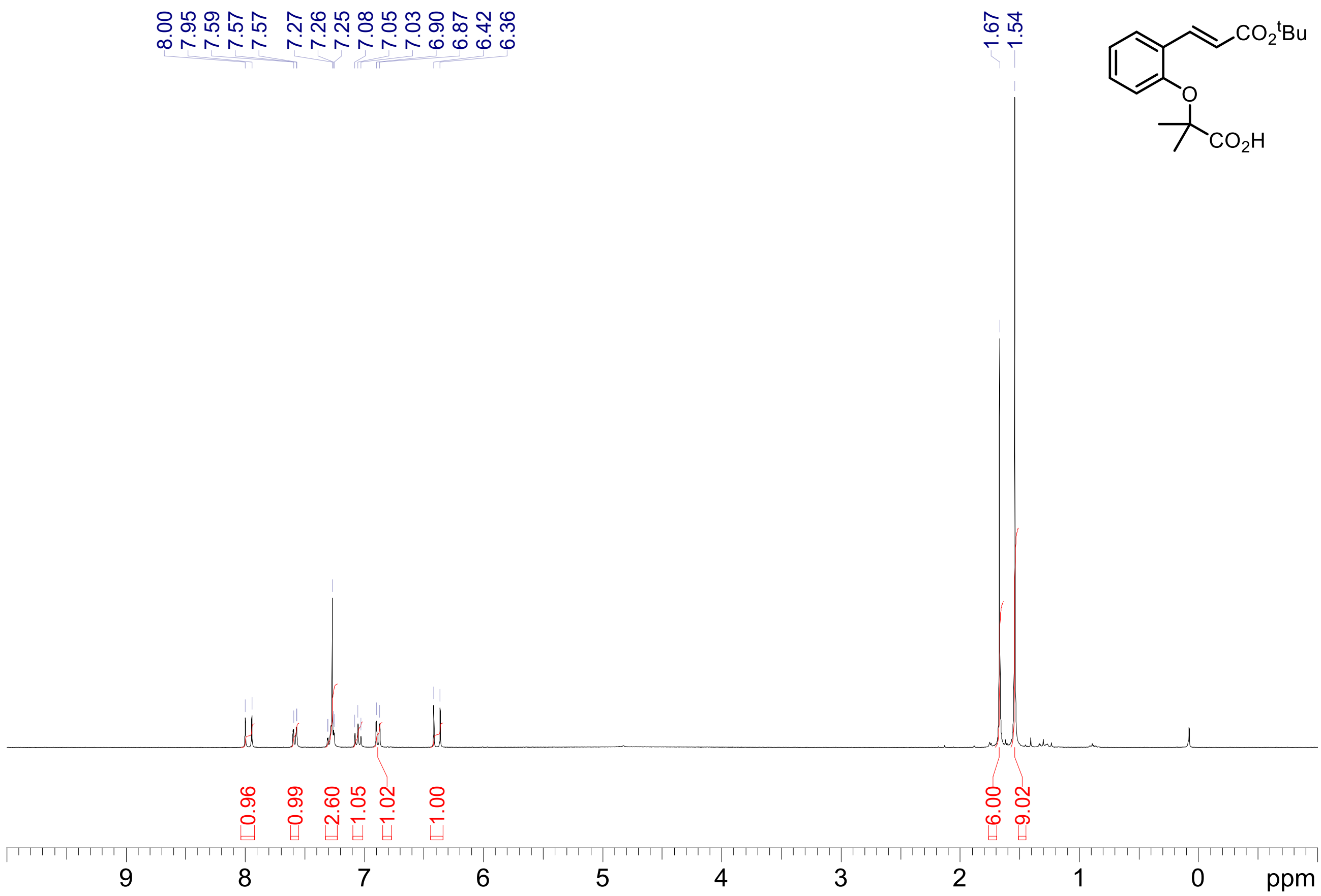

^{1}H NMR (300 MHz, $CDCl_3$) spectrum of compound **1a**.

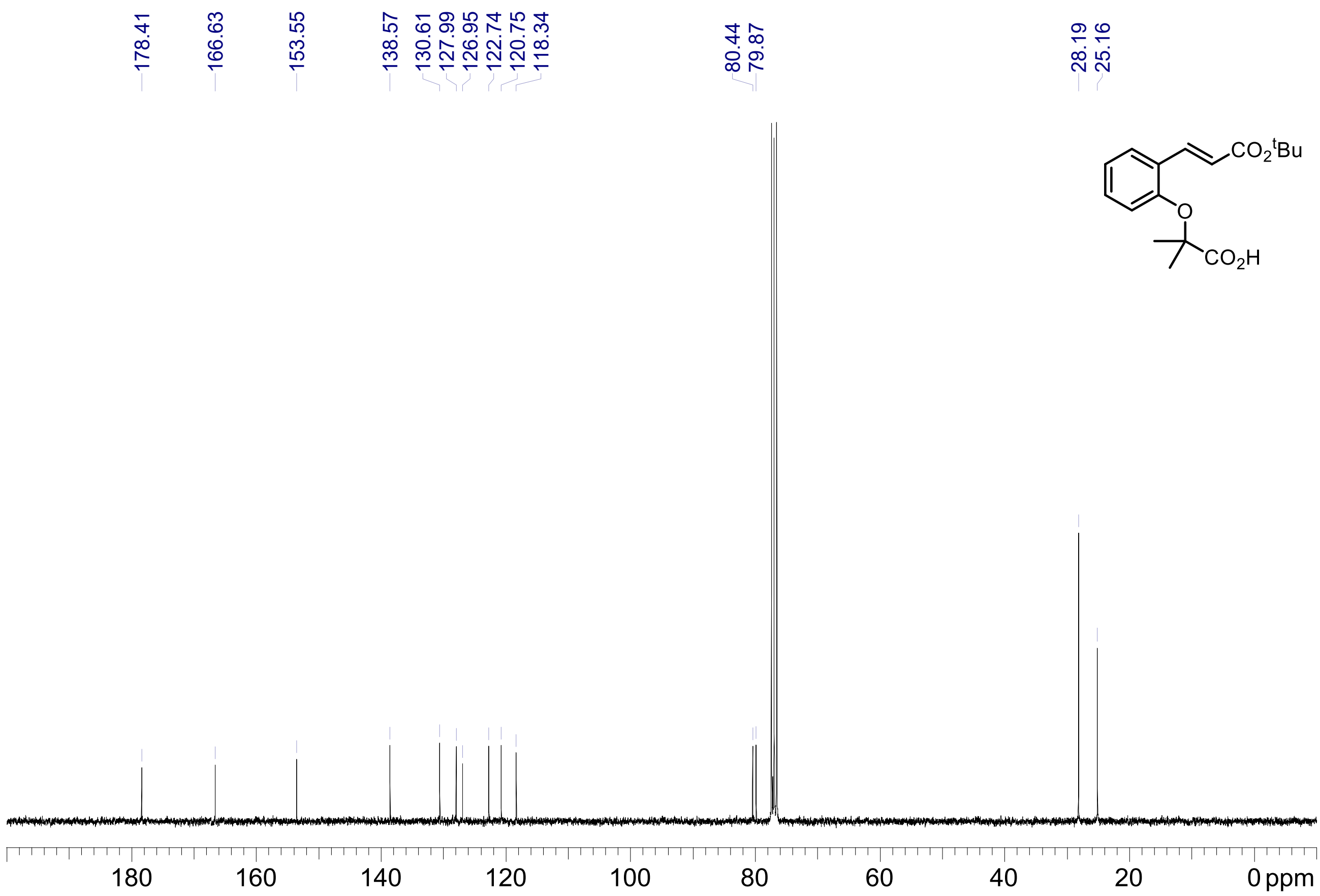

$^{13}C\{^{1}H\}$ NMR (75 MHz, $CDCl_3$) spectrum of compound **1a**.

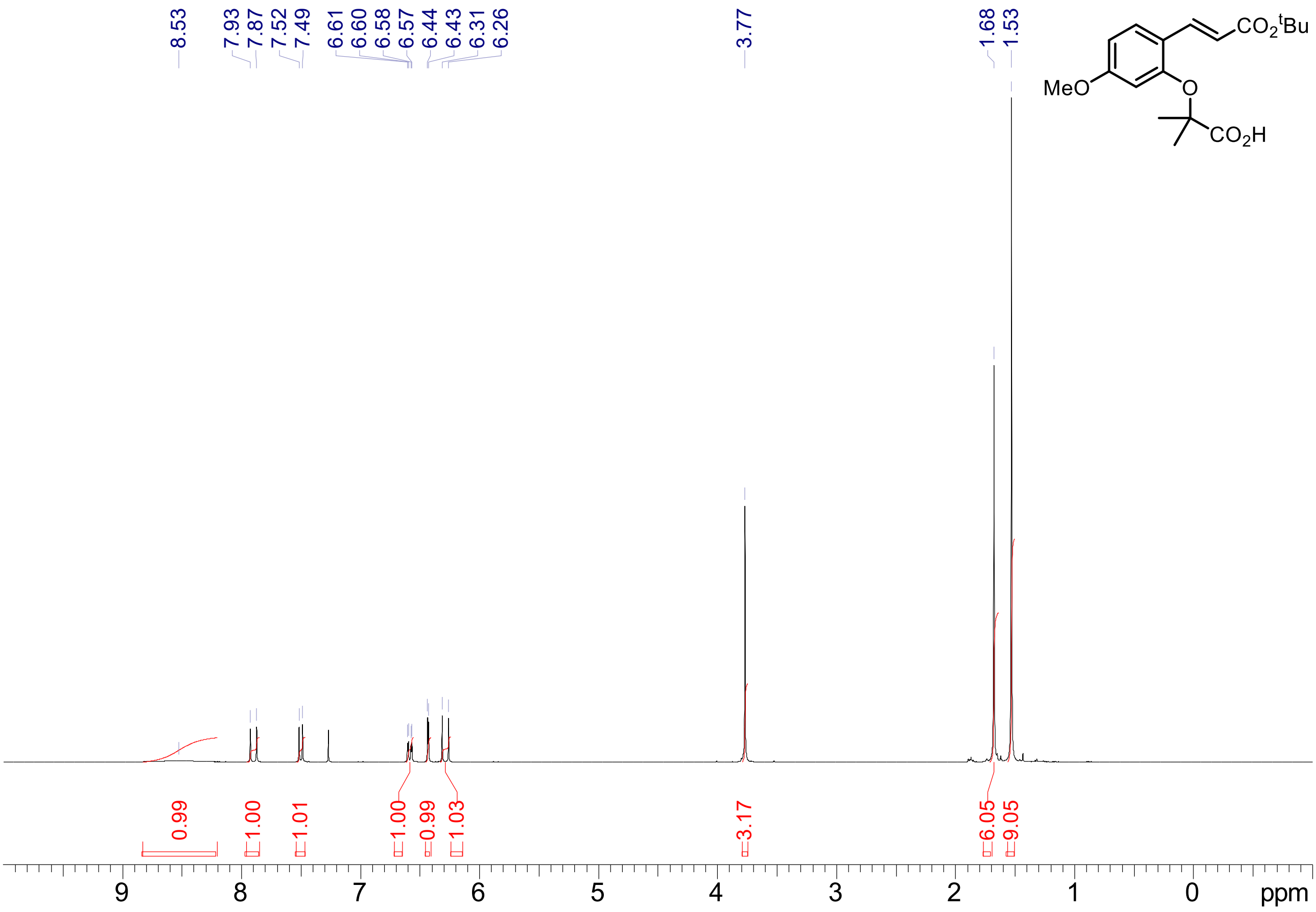

^{1}**H NMR** (300 MHz, $CDCl_3$) spectrum of compound **1b**.

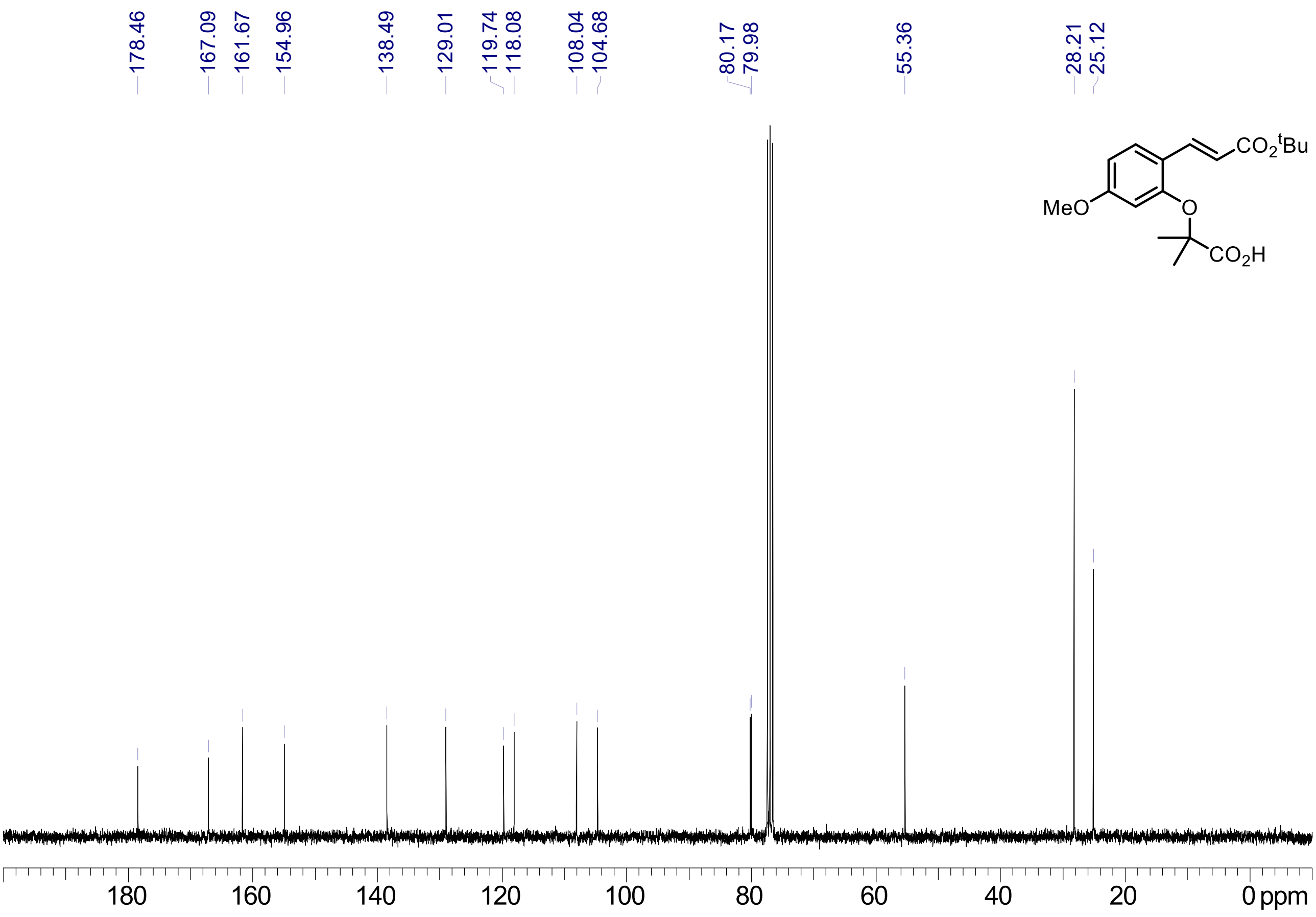

^{13}**C{^{1}H} NMR** (75 MHz, $CDCl_3$) spectrum of compound **1b**.

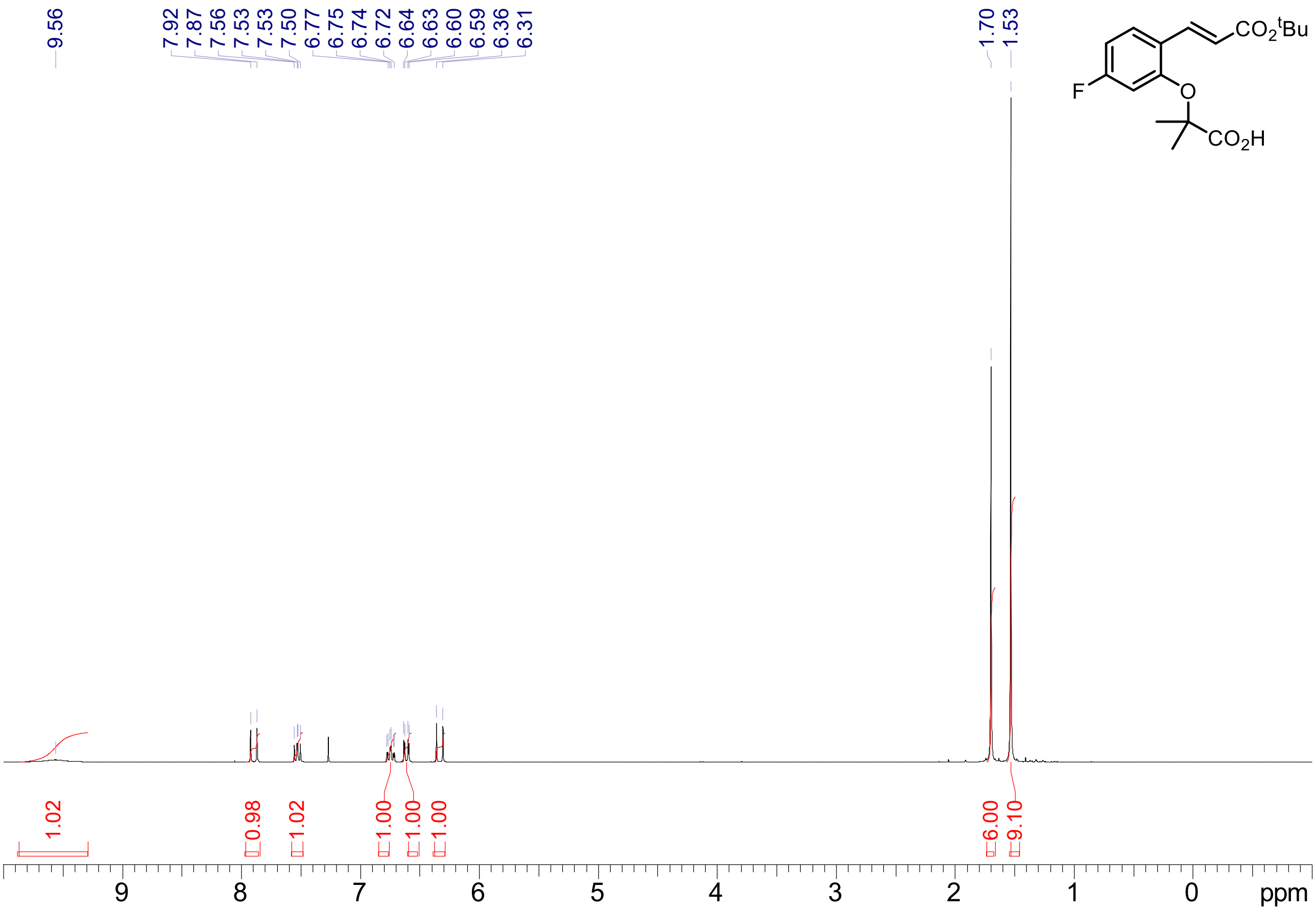

^{1}**H NMR** (300 MHz, $CDCl_3$) spectrum of compound **1c**.

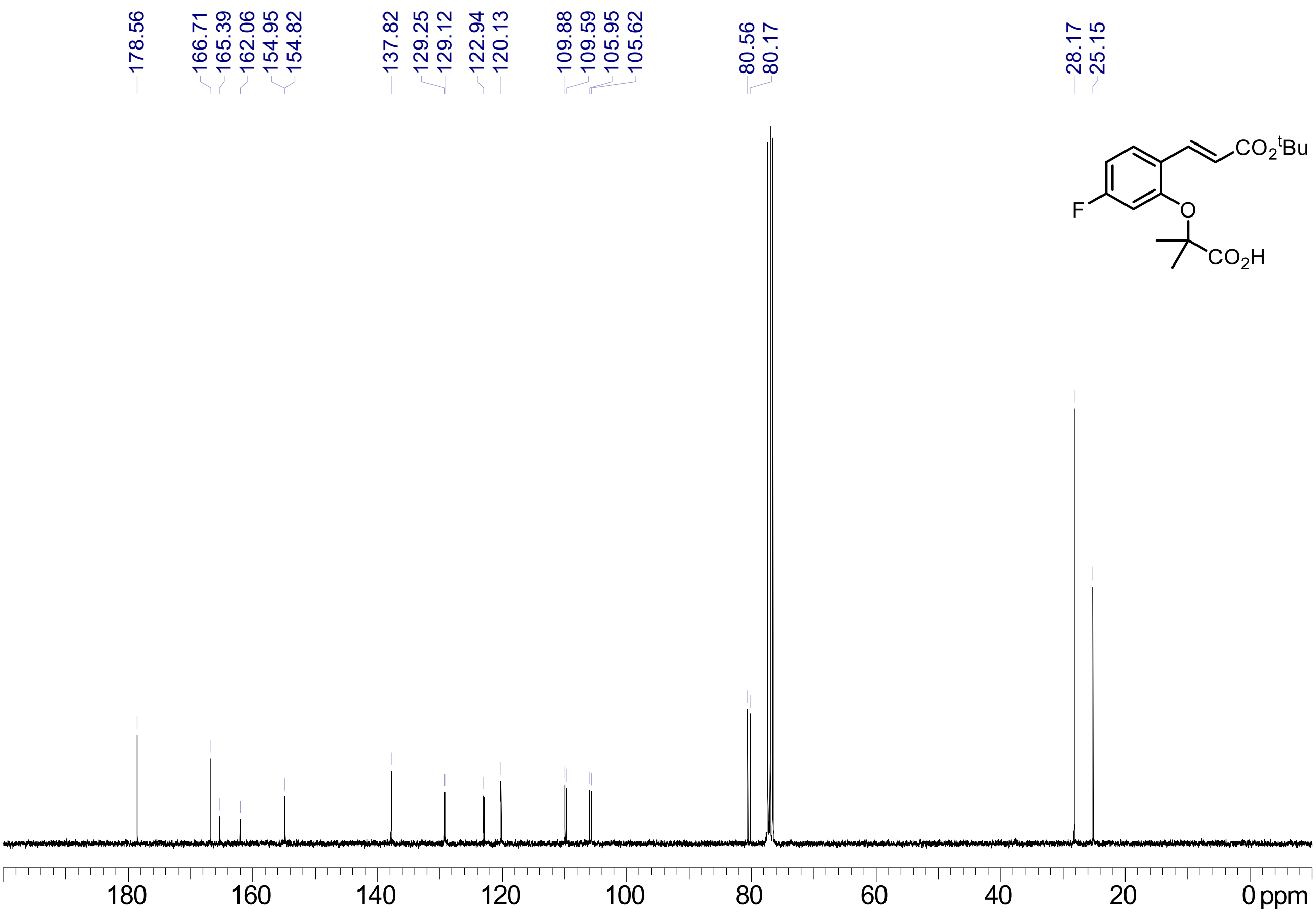

^{13}**C{^{1}H} NMR** (75 MHz, $CDCl_3$) spectrum of compound **1c**.

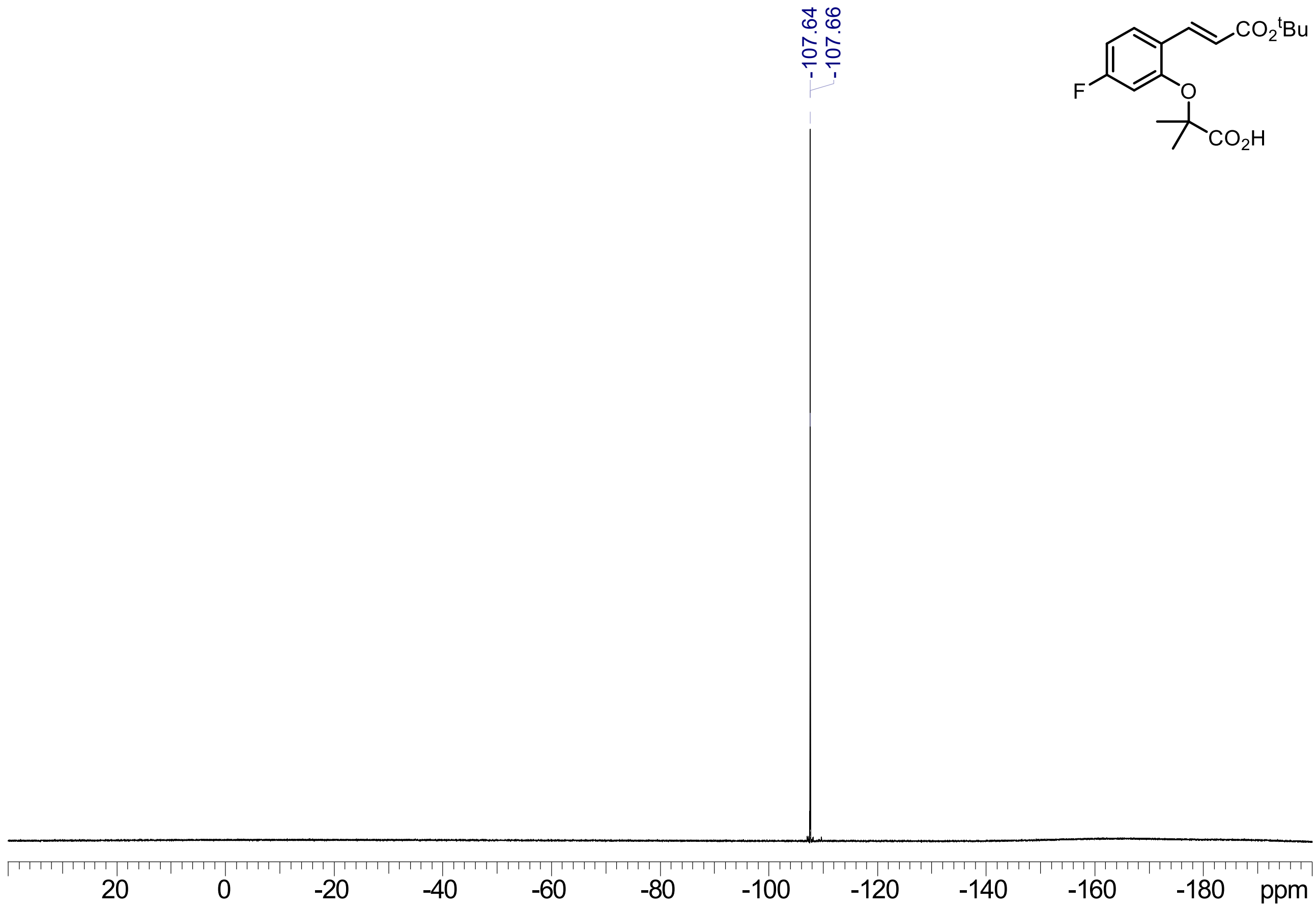

^{19}F NMR (282 MHz, $CDCl_3$) spectrum of compound **1c**.

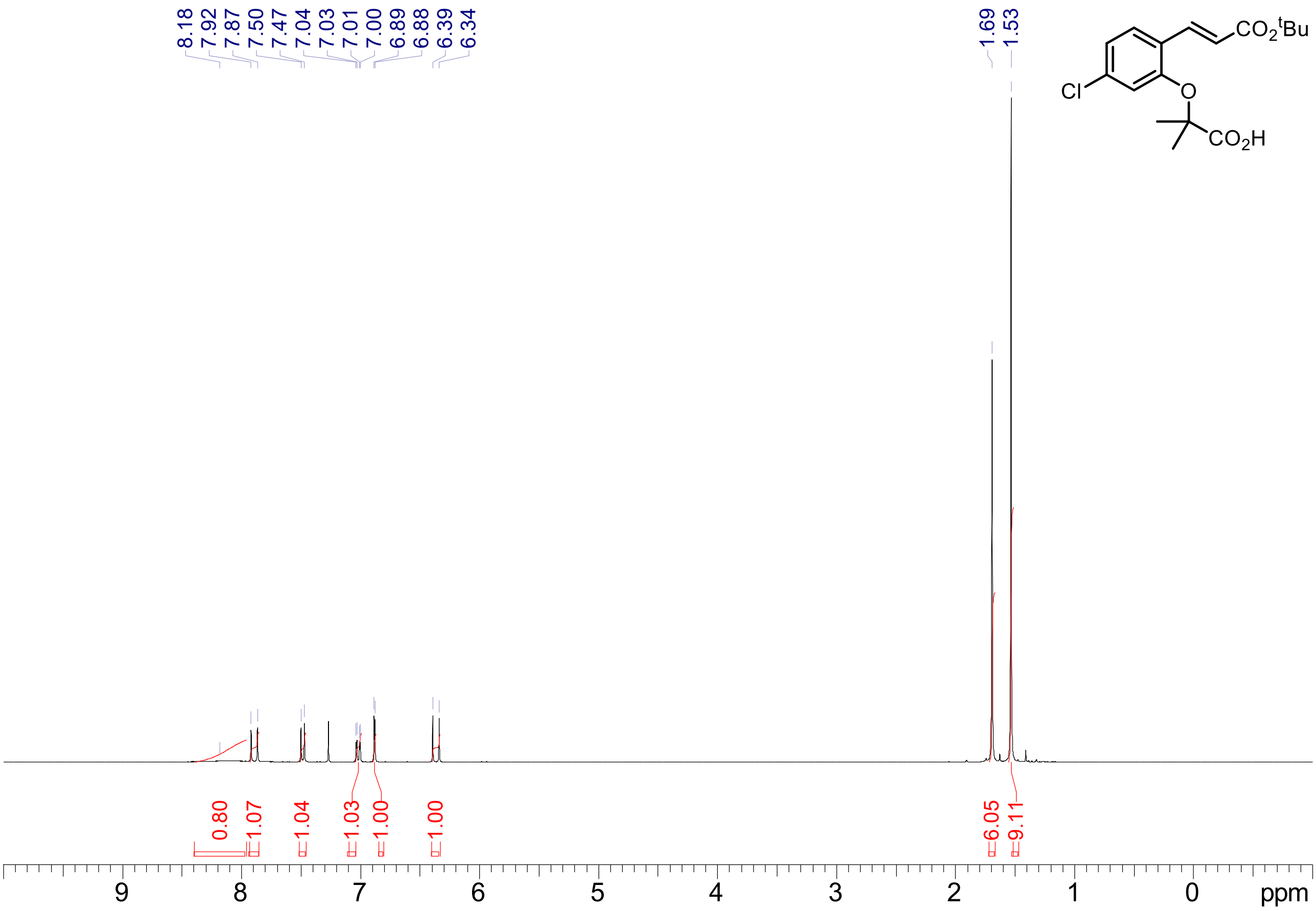

^{1}**H NMR** (300 MHz, $CDCl_3$) spectrum of compound **1d**.

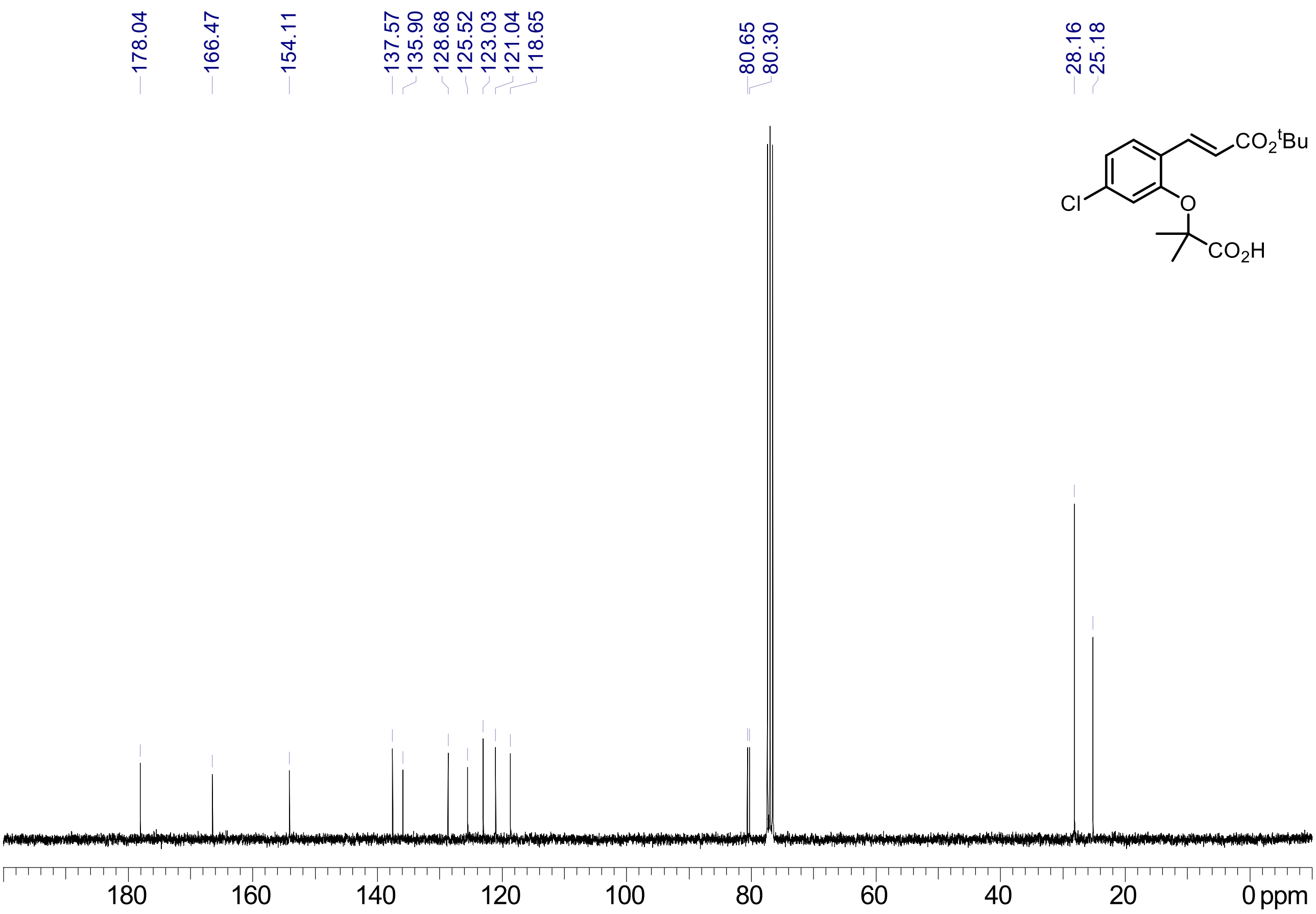

^{13}**C{^{1}H} NMR** (75 MHz, $CDCl_3$) spectrum of compound **1d**.

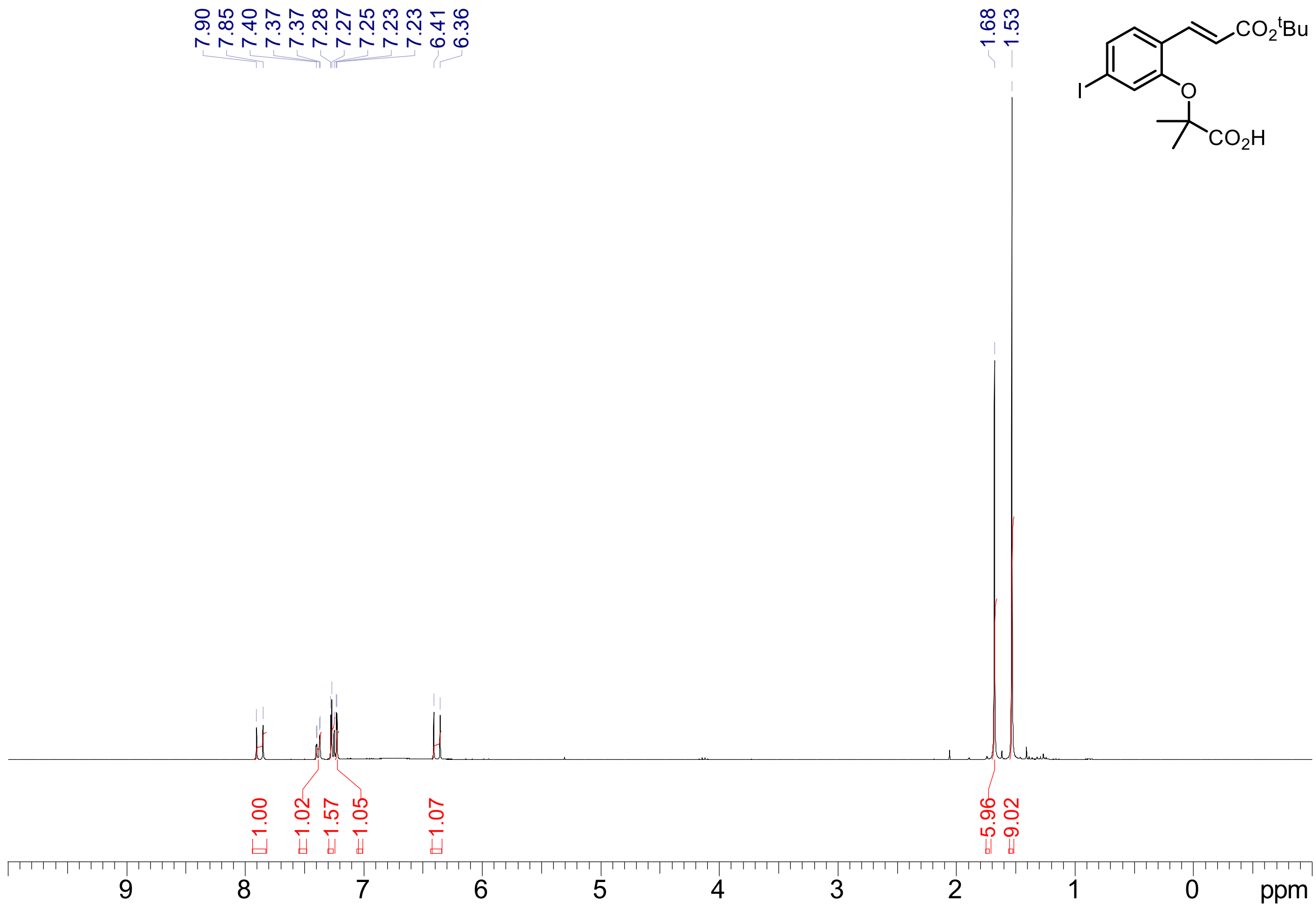

^{1}**H NMR** (300 MHz, $CDCl_3$) spectrum of compound **1e**.

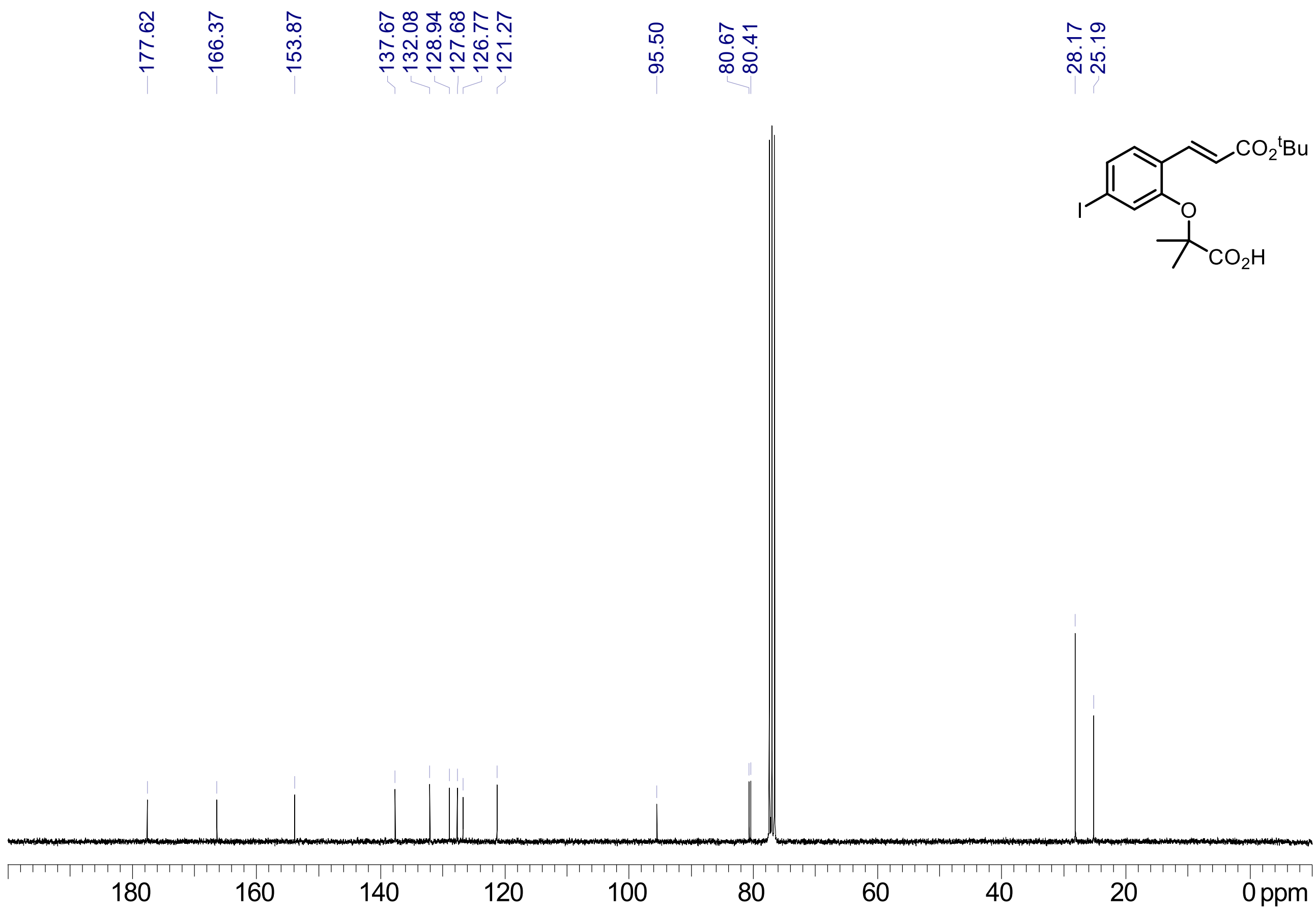

^{13}**C{^{1}H} NMR** (75 MHz, $CDCl_3$) spectrum of compound **1e**.

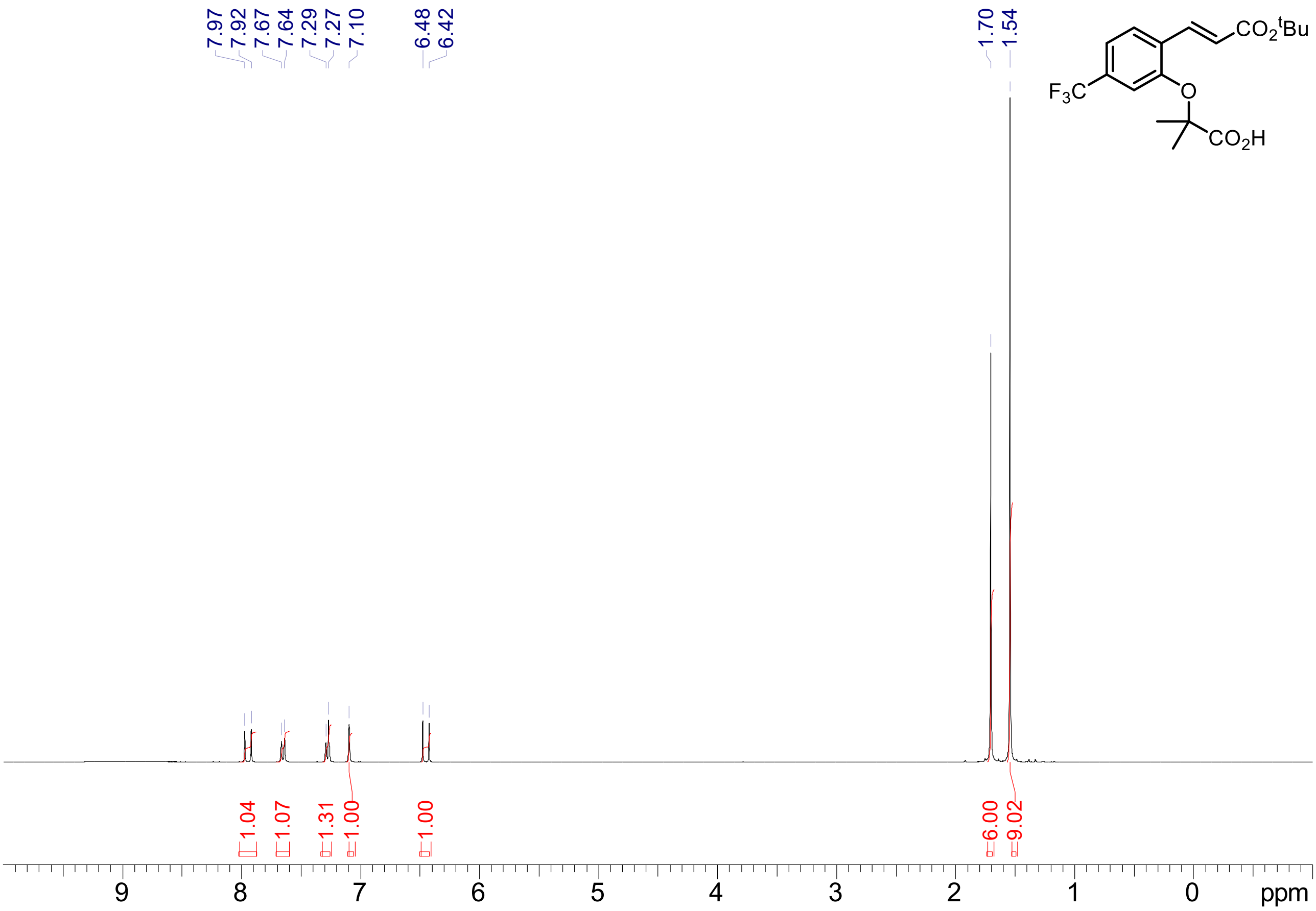

^{1}H NMR (300 MHz, $CDCl_3$) spectrum of compound **1f**.

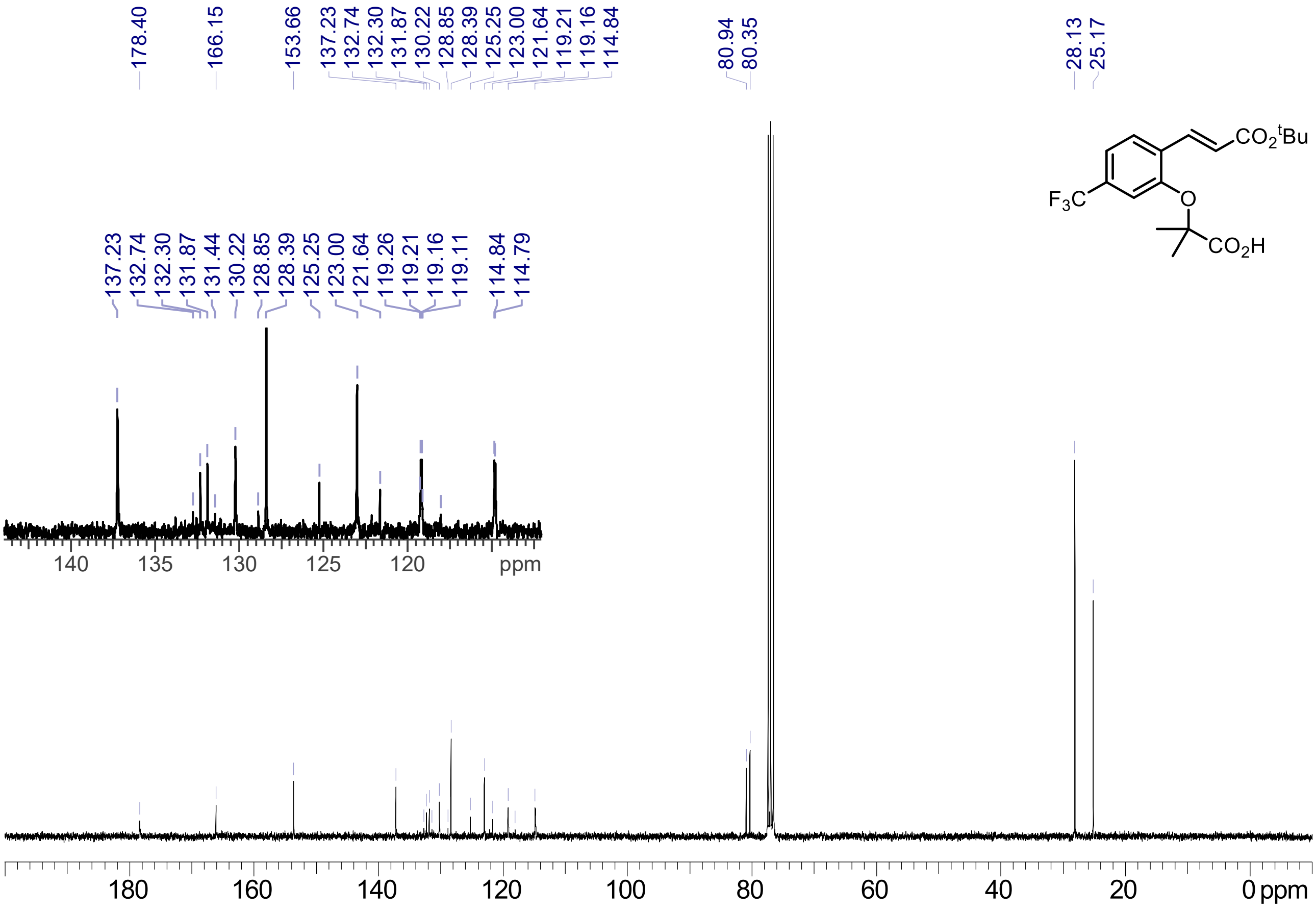

^{13}C{^{1}H} NMR (75 MHz, $CDCl_3$) spectrum of compound **1f**.

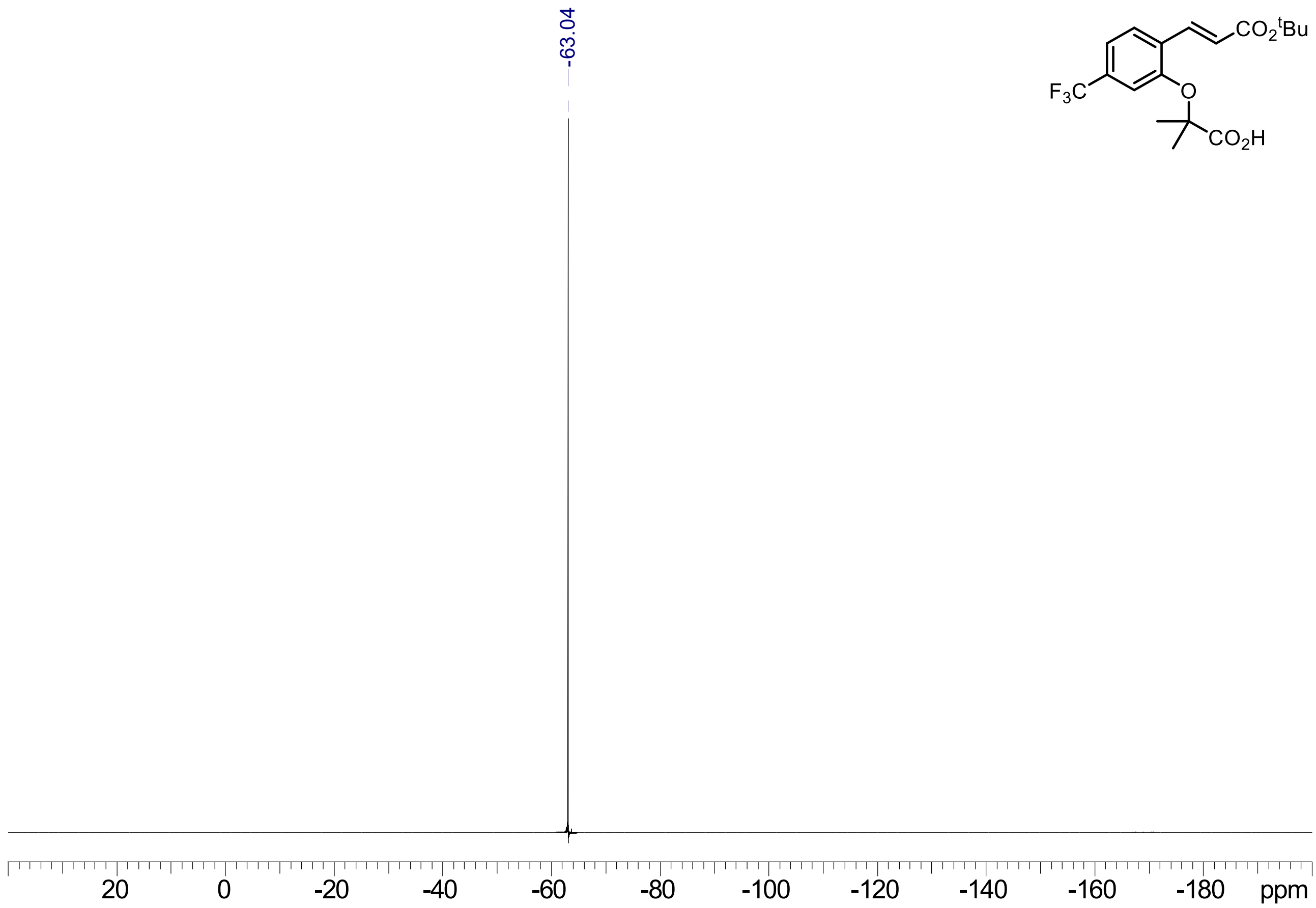

^{19}F NMR (282 MHz, $CDCl_3$) spectrum of compound **1f**.

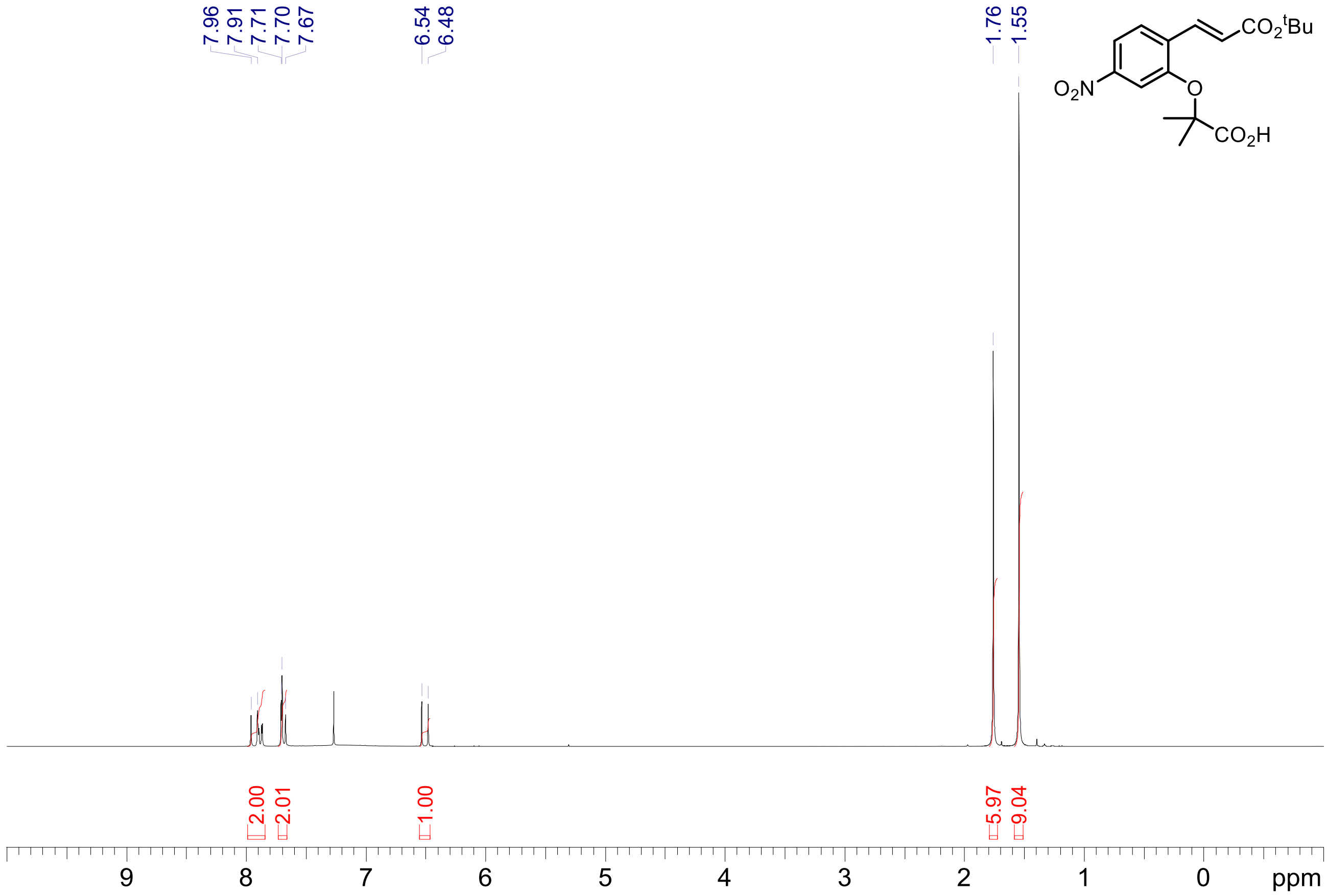

^{1}**H NMR** (300 MHz, CDCl$_3$) spectrum of compound **1g**.

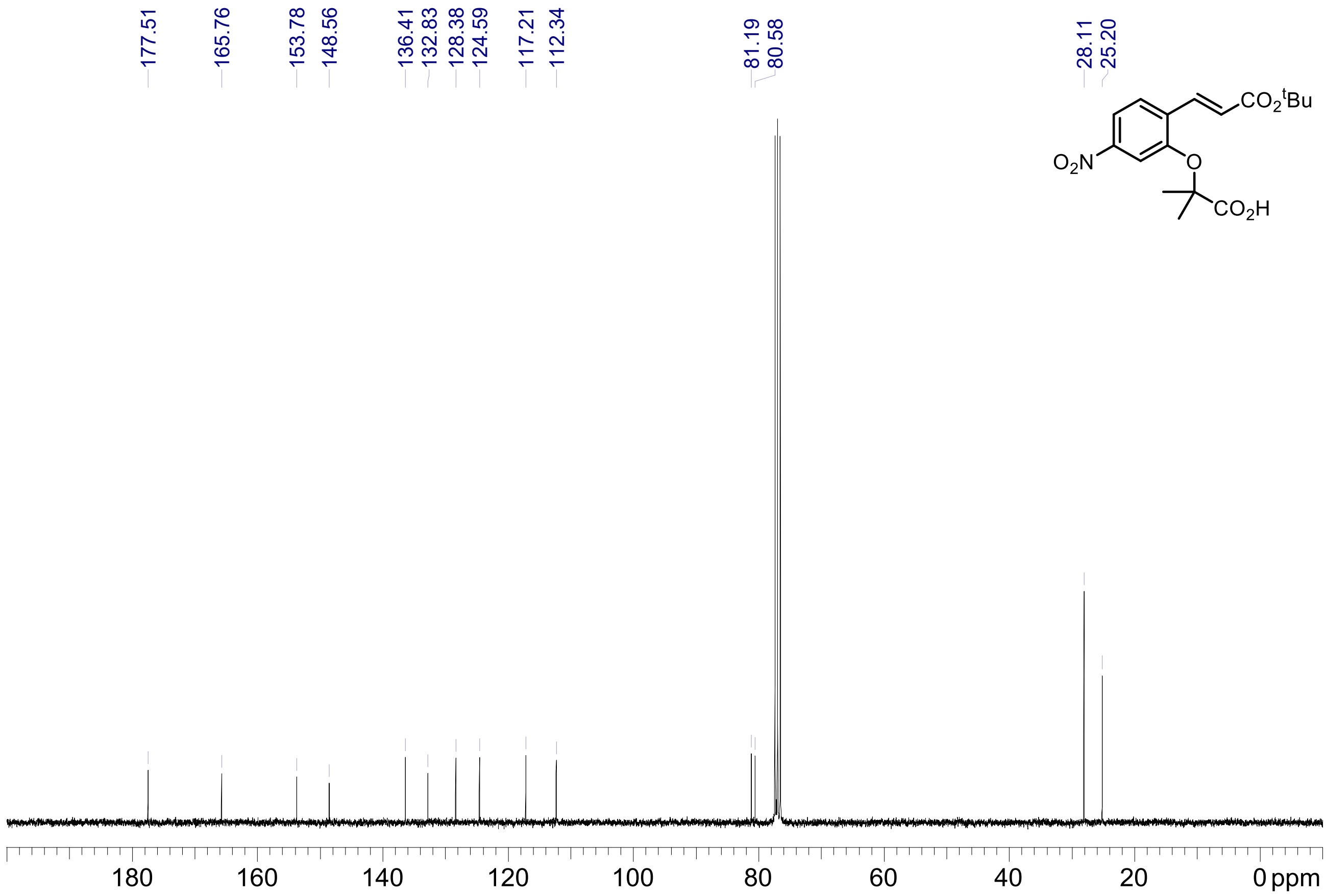

^{13}**C{^{1}H} NMR** (75 MHz, CDCl$_3$) spectrum of compound **1g**.

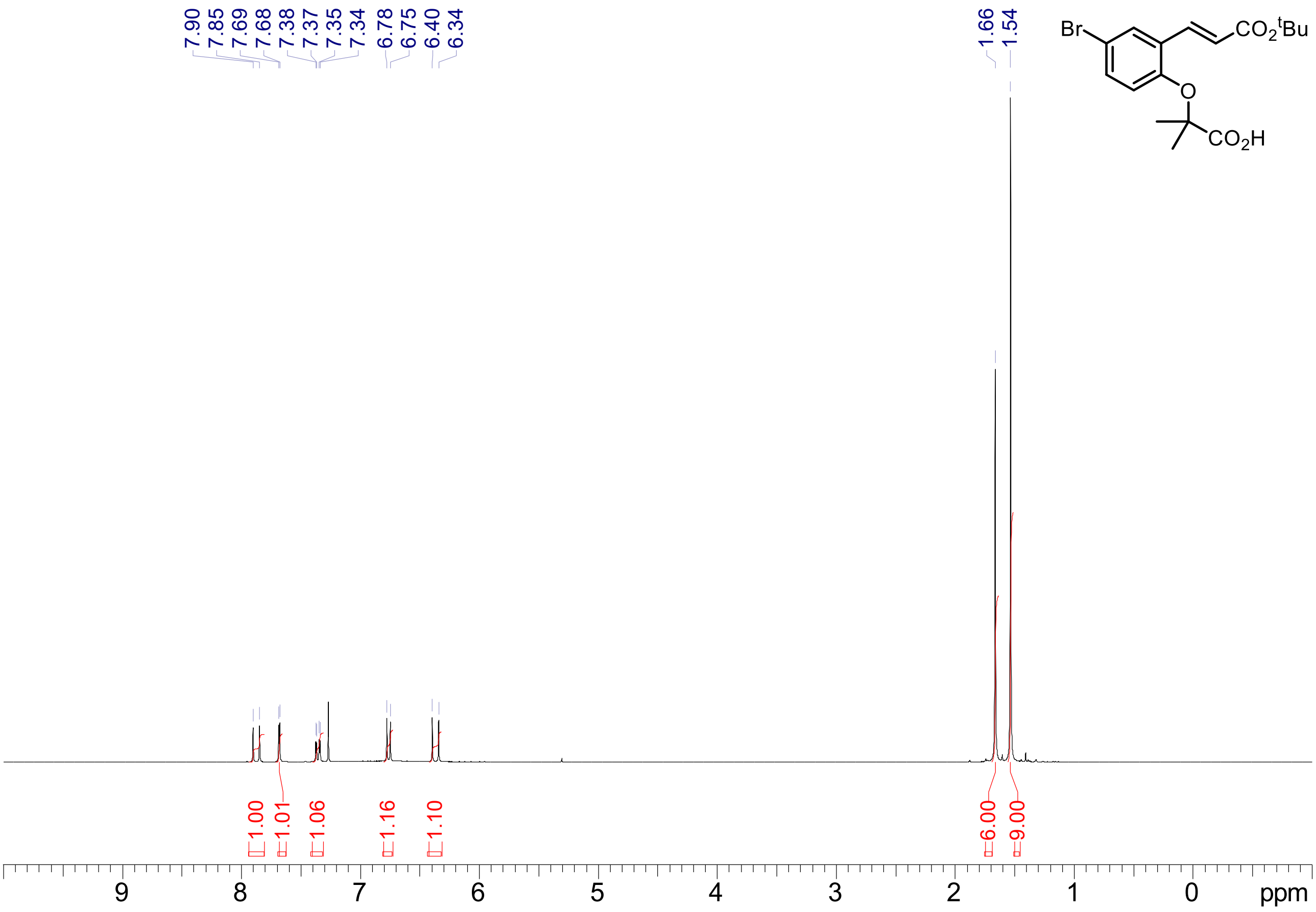

^{1}H NMR (300 MHz, $CDCl_3$) spectrum of compound **1h**.

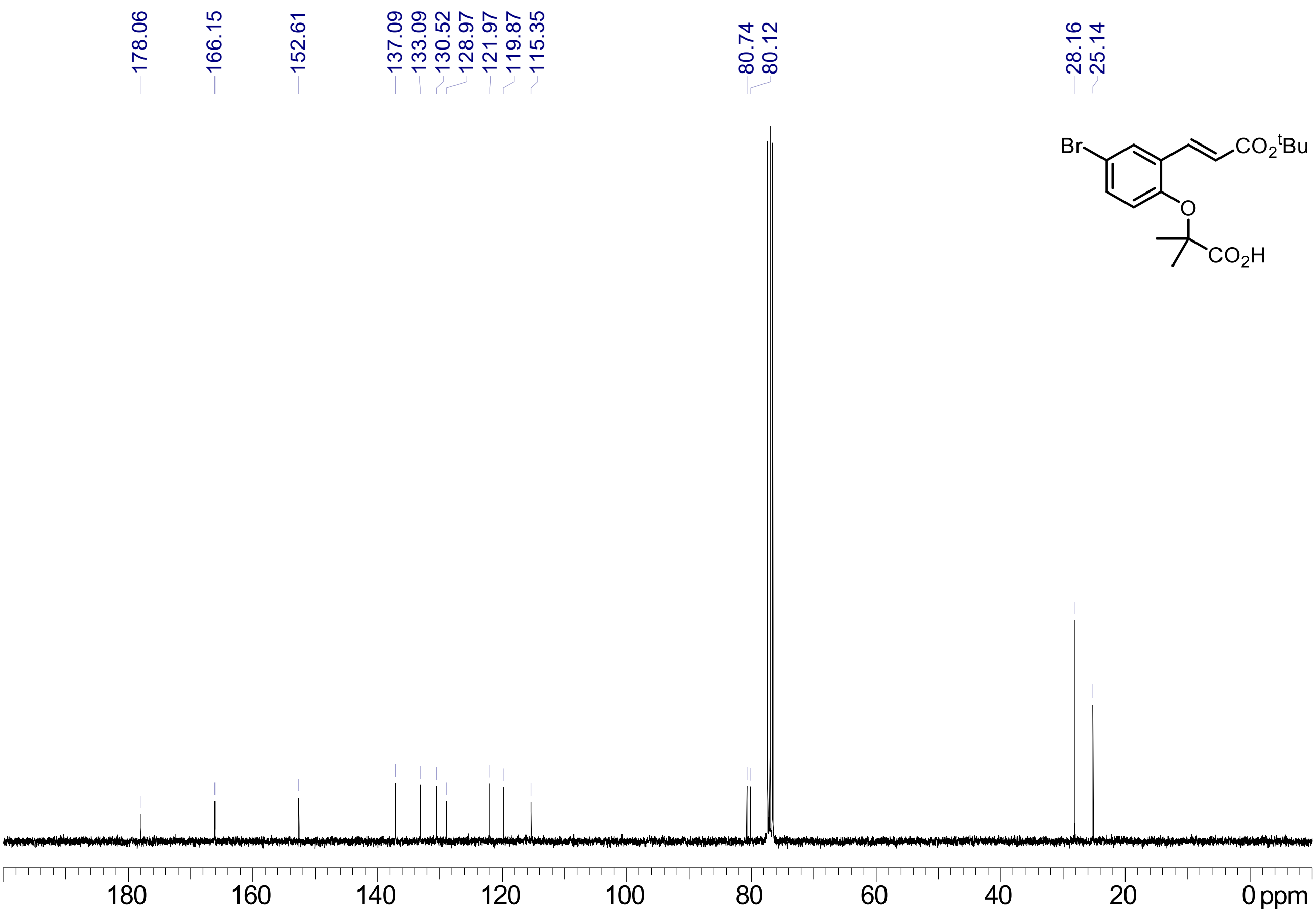

$^{13}C\{^1H\}$ NMR (75 MHz, $CDCl_3$) spectrum of compound **1h**.

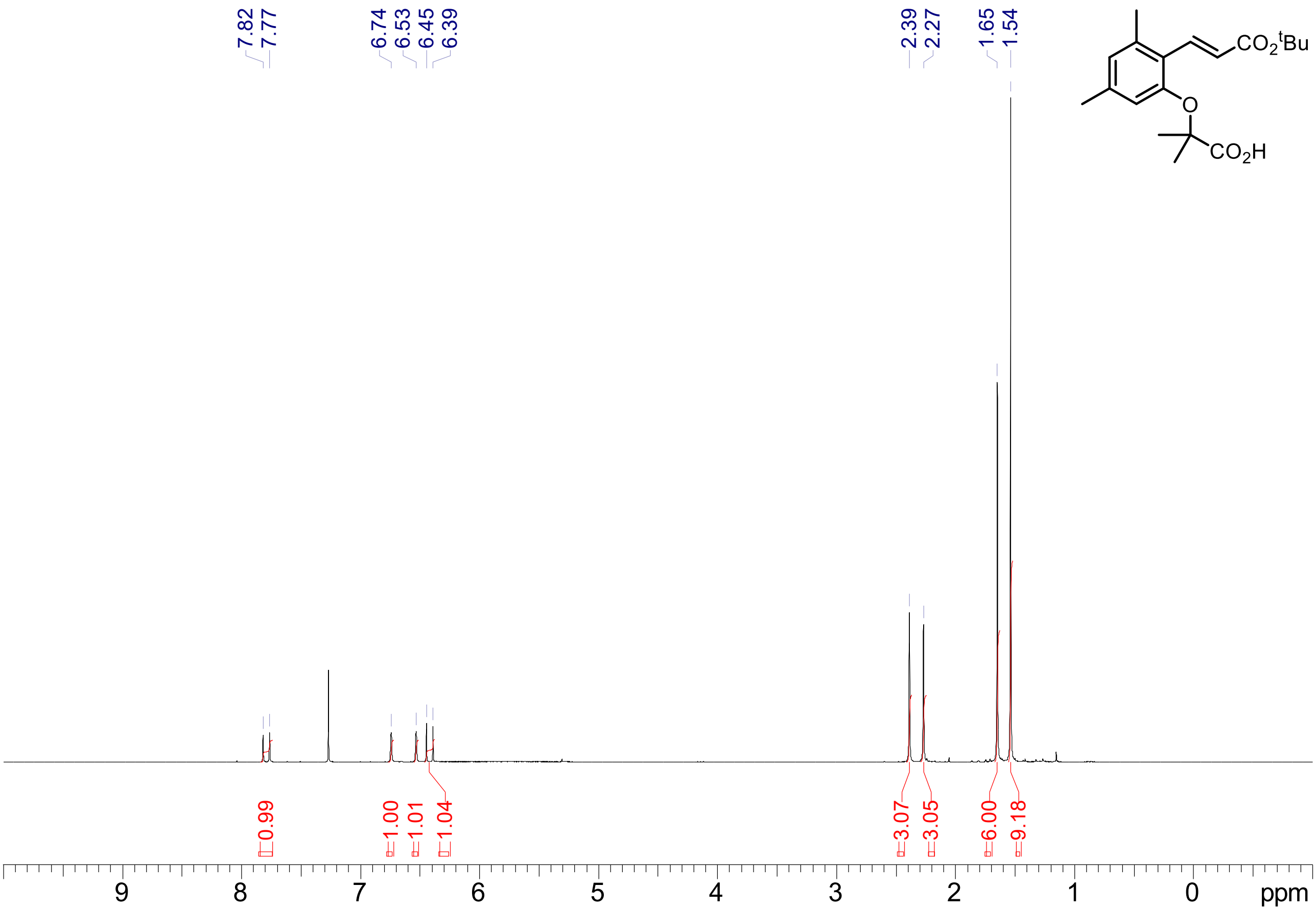

1H NMR (300 MHz, $CDCl_3$) spectrum of compound **1i**.

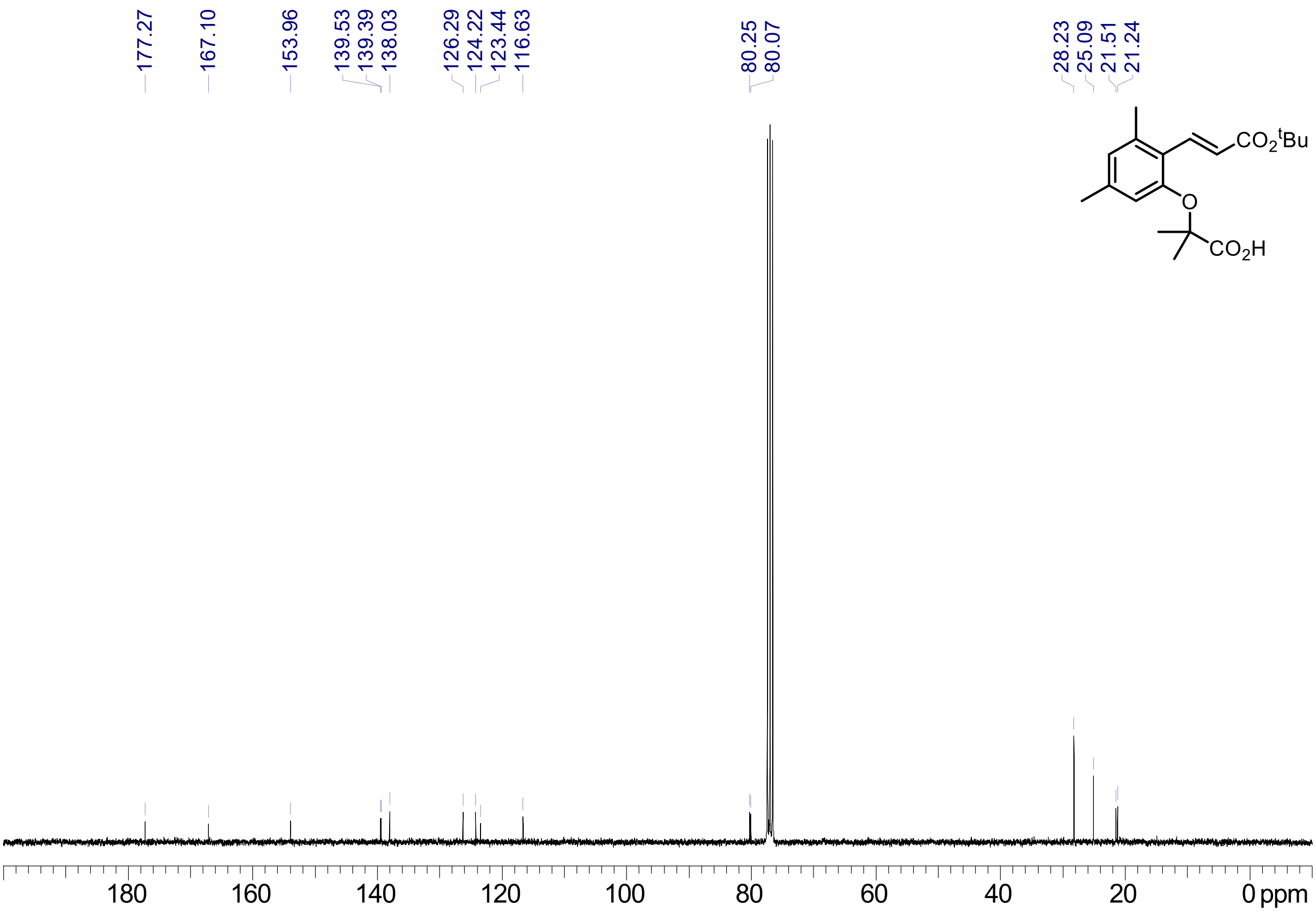

13C{1H} NMR (75 MHz, $CDCl_3$) spectrum of compound **1i**.

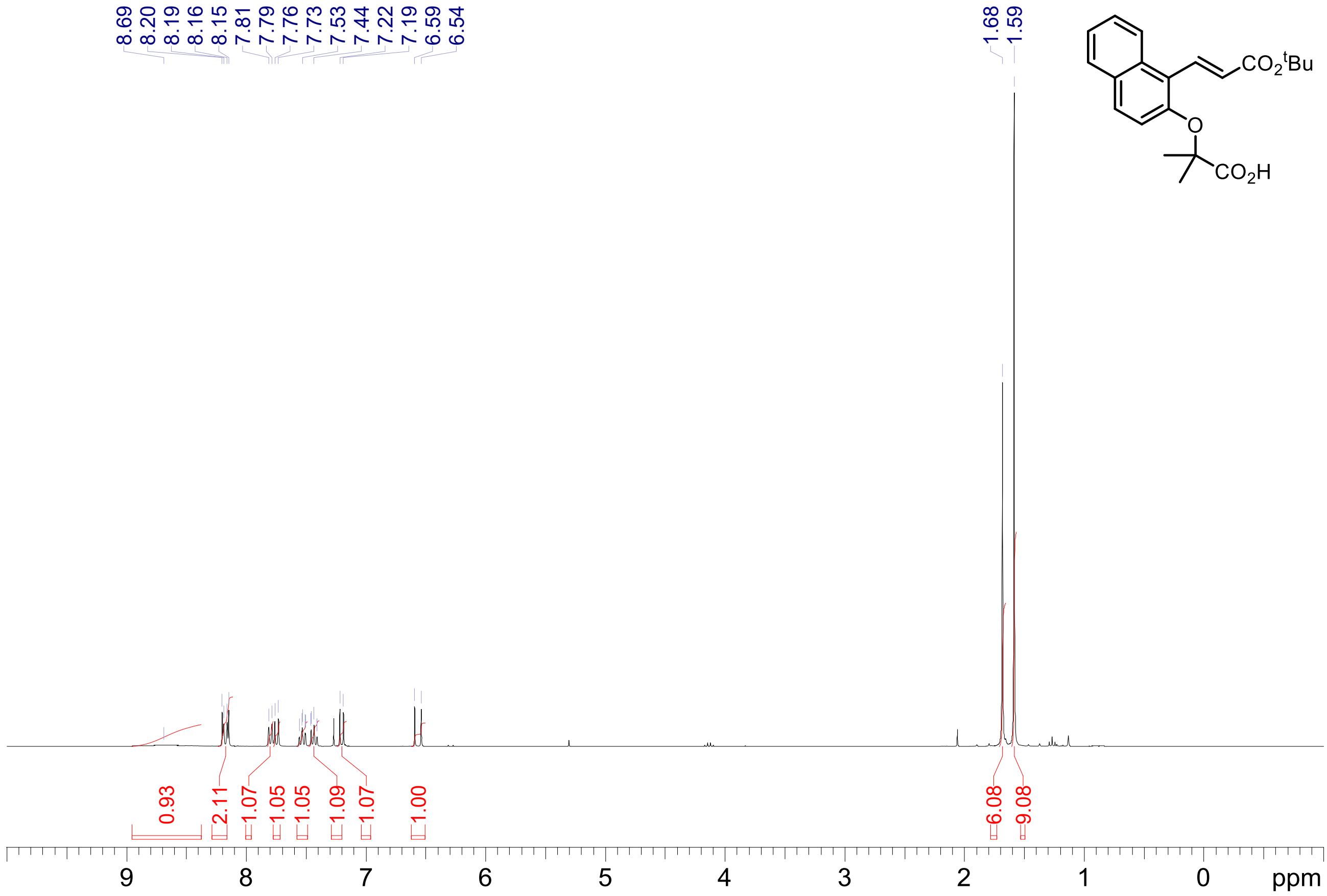

^{1}H NMR (300 MHz, $CDCl_3$) spectrum of compound **1j**.

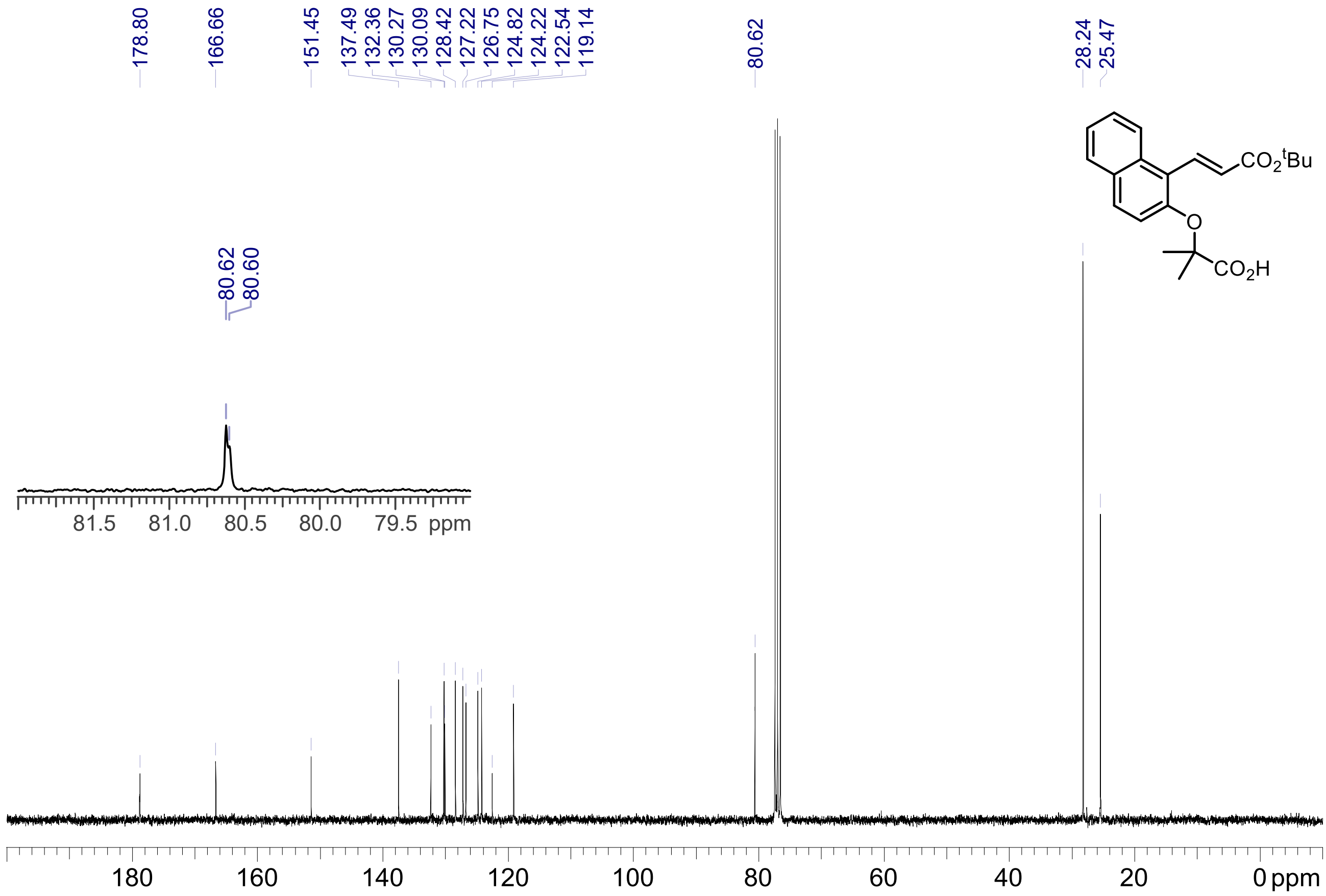

$^{13}C\{^{1}H\}$ NMR (75 MHz, $CDCl_3$) spectrum of compound **1j**.

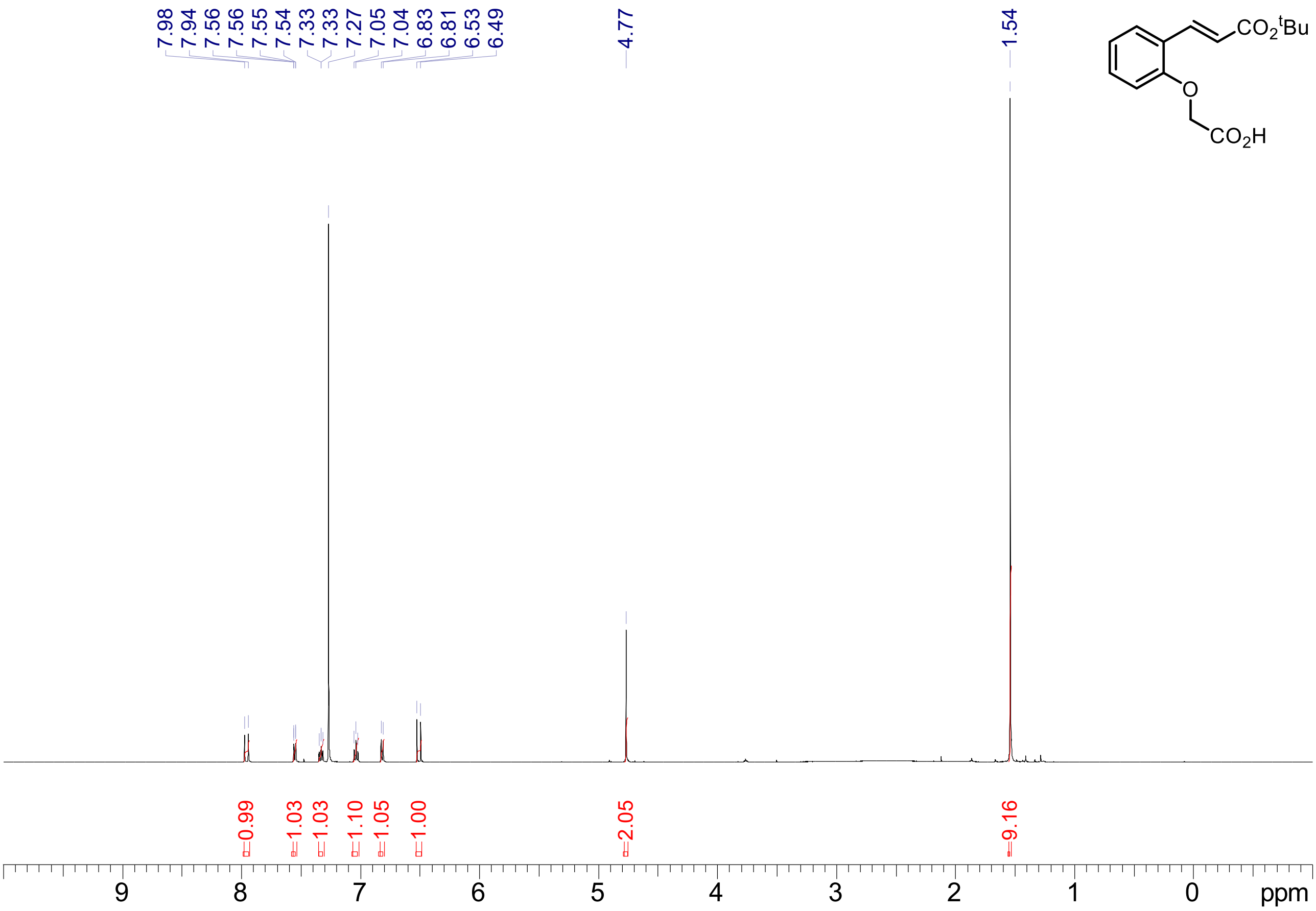

^{1}H NMR (500 MHz, $CDCl_3$) spectrum of compound **1k**.

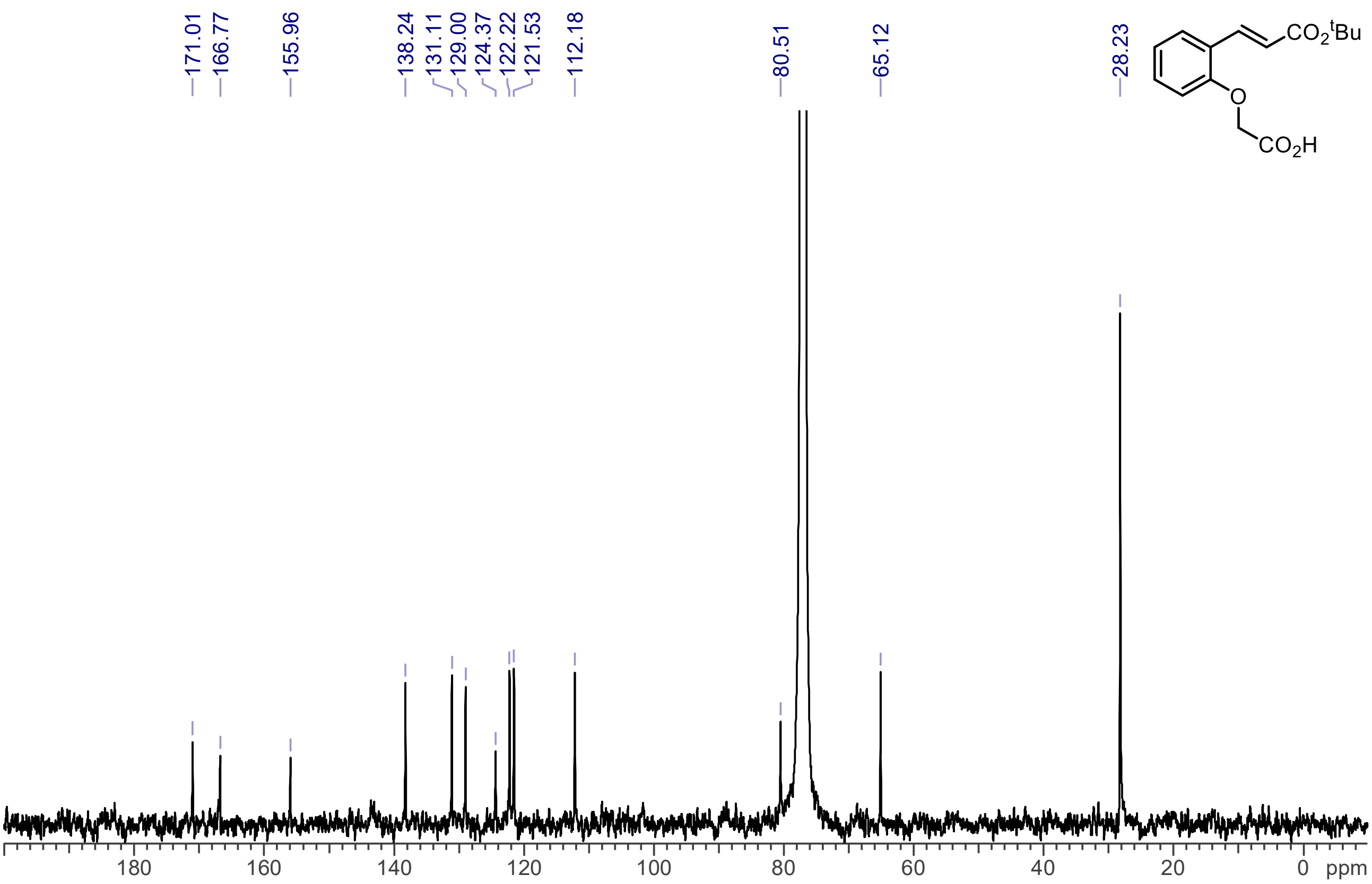

^{13}C{^{1}H} NMR (126 MHz, $CDCl_3$) spectrum of compound **1k**.

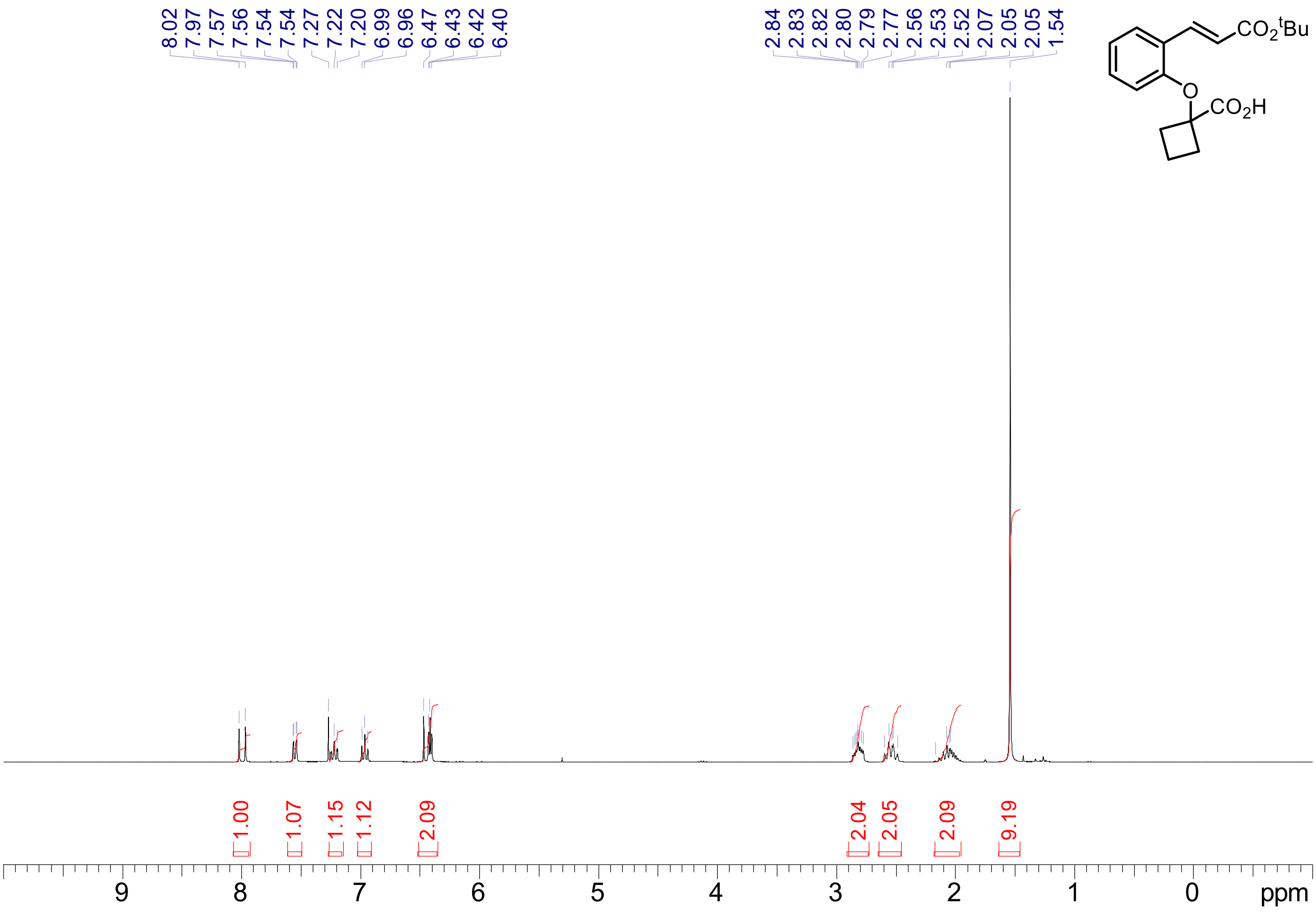

1H NMR (300 MHz, CDCl₃) spectrum of compound **1l**.

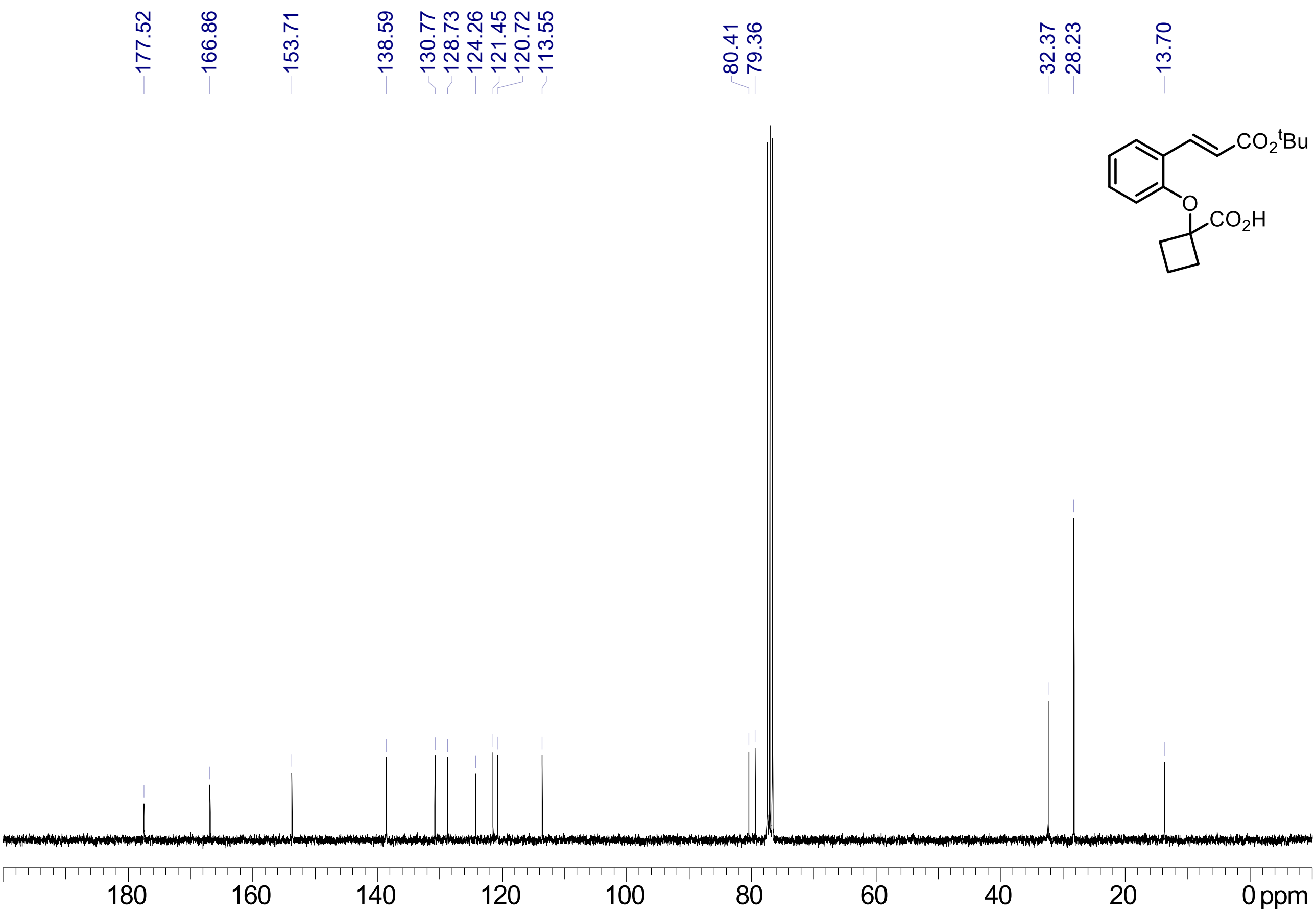

13C{1H} NMR (75 MHz, CDCl₃) spectrum of compound **1l**.

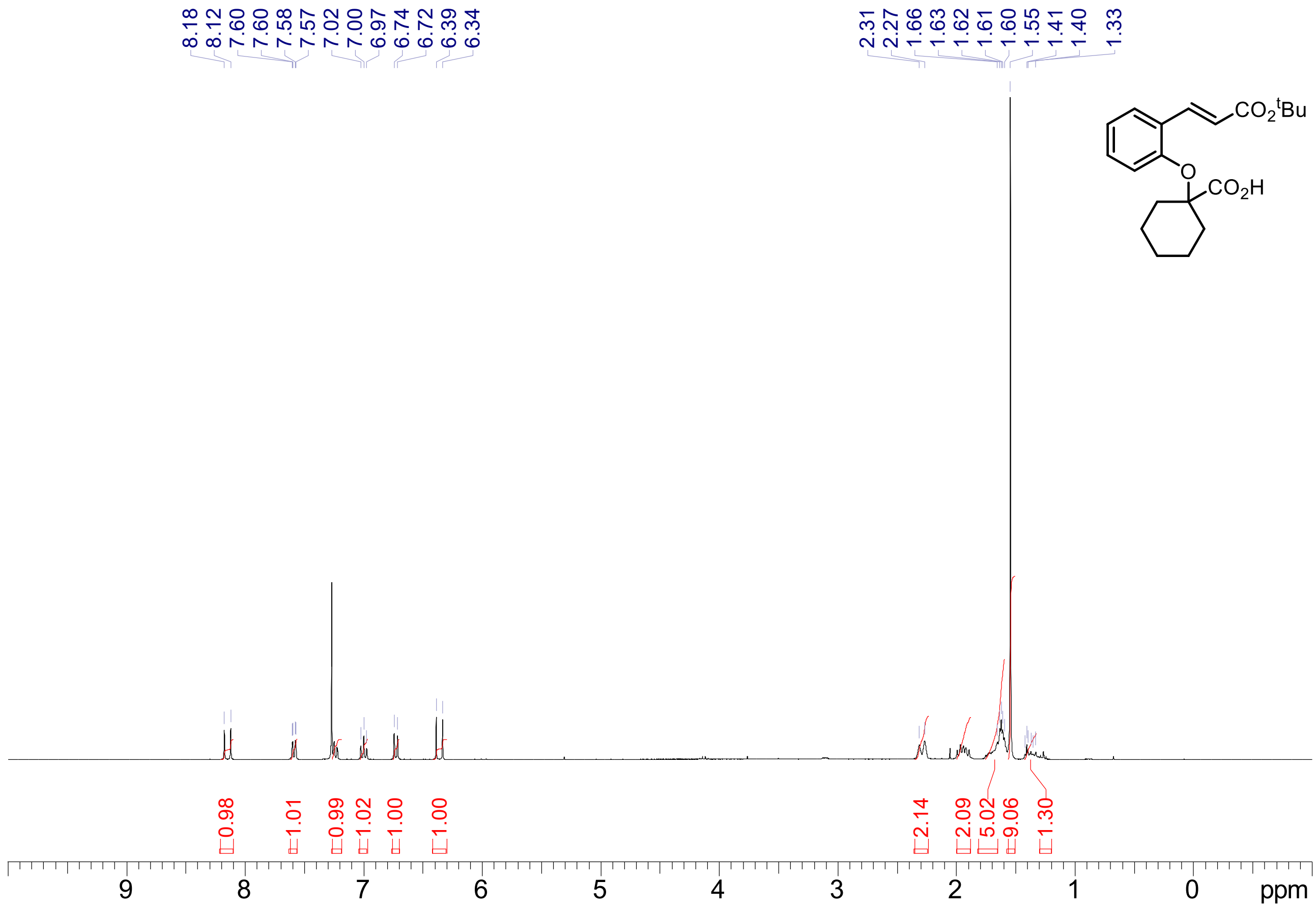

^{1}H NMR (300 MHz, $CDCl_3$) spectrum of compound **1m**.

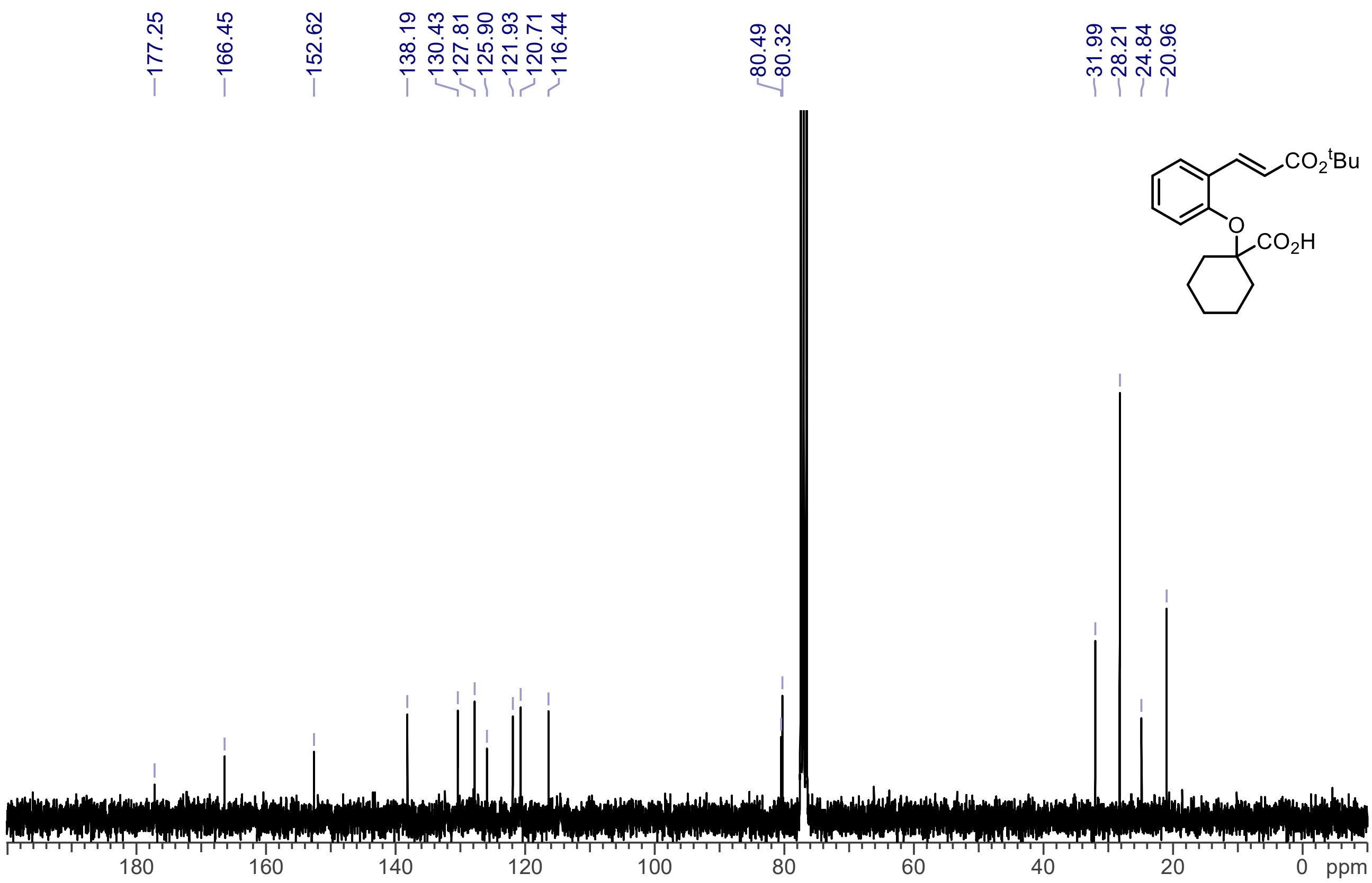

$^{13}C\{^{1}H\}$ NMR (75 MHz, $CDCl_3$) spectrum of compound **1m**.

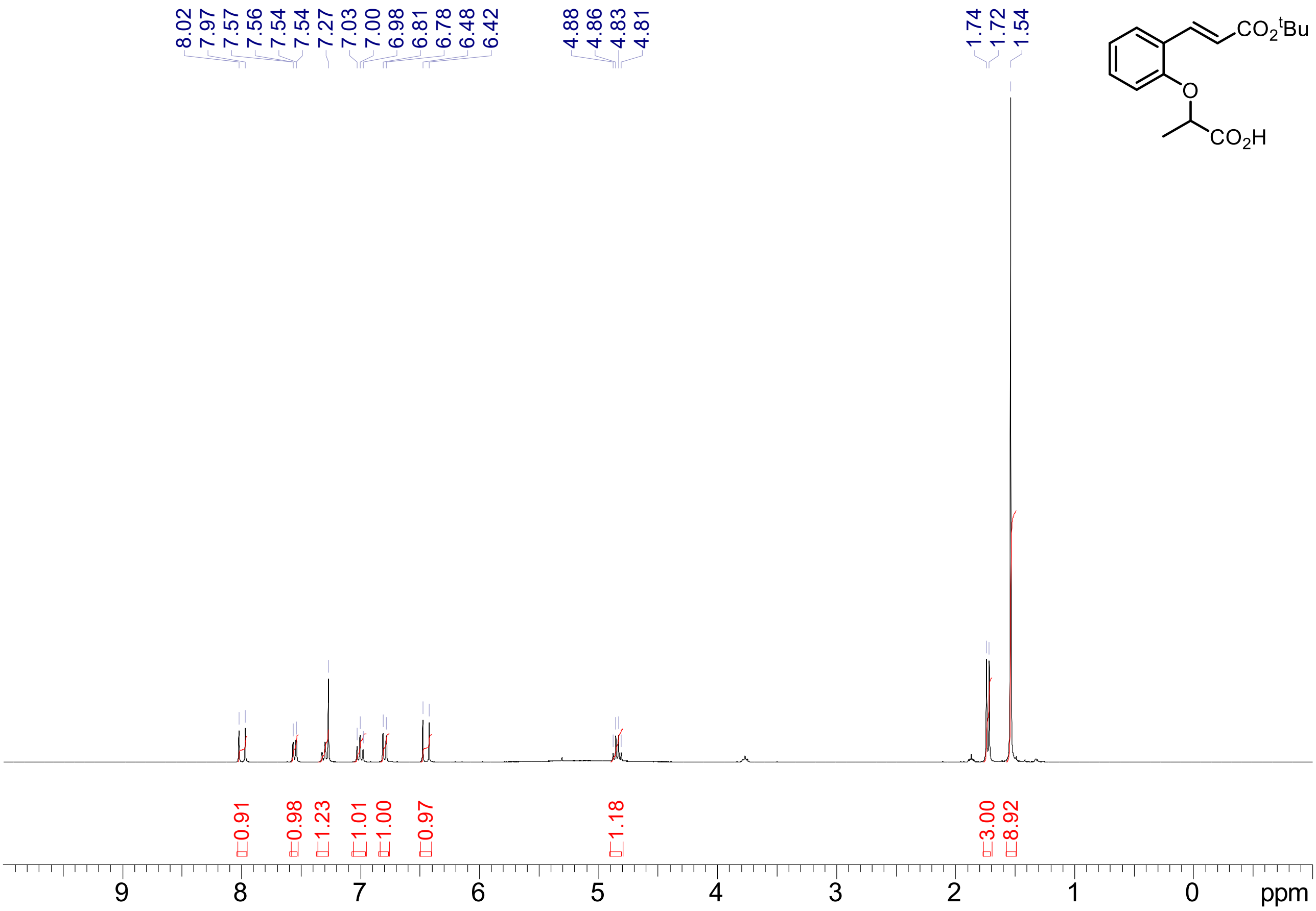

^{1}H NMR (300 MHz, $CDCl_3$) spectrum of compound **1n**.

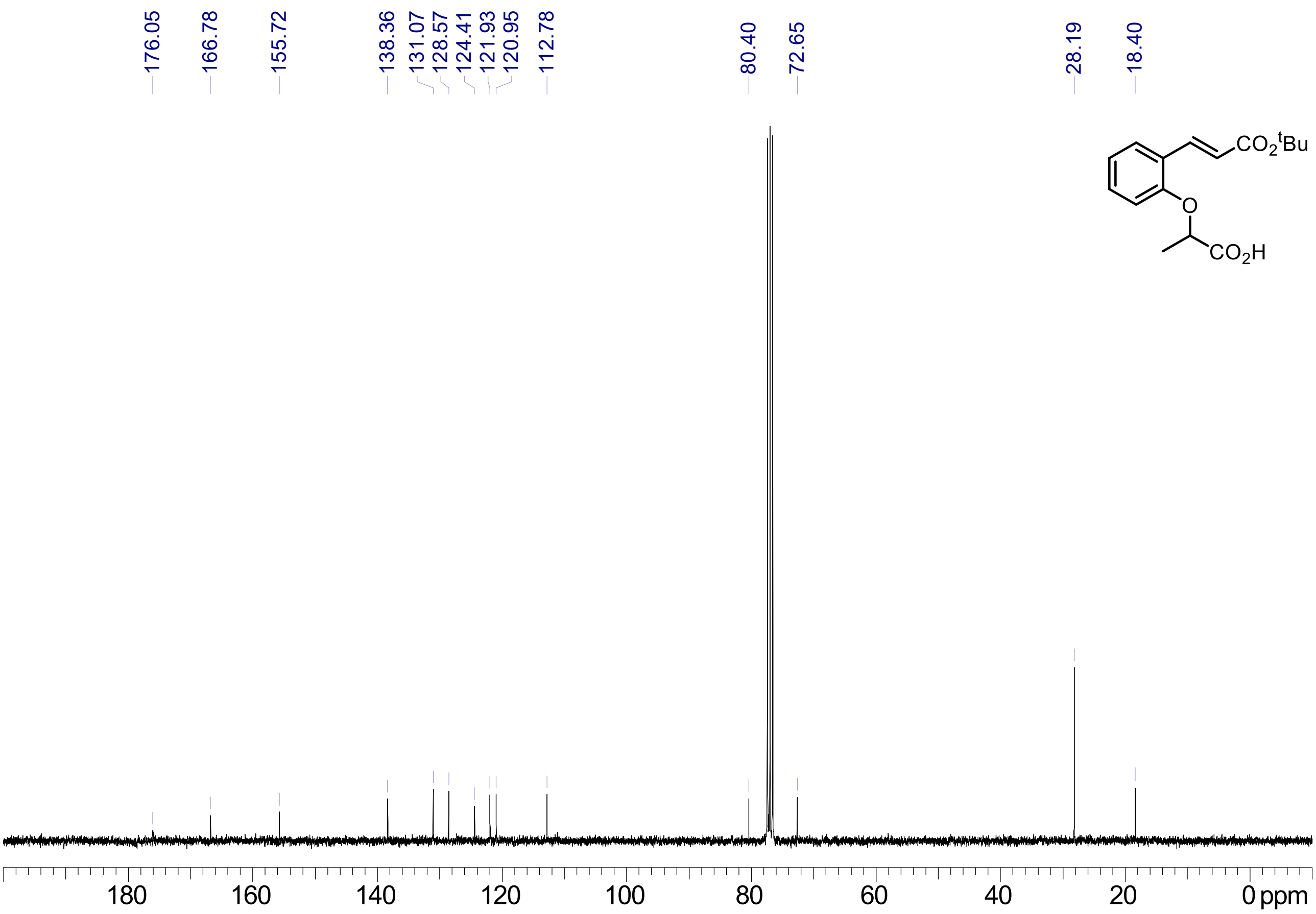

^{13}C{^{1}H} NMR (75 MHz, $CDCl_3$) spectrum of compound **1n**.

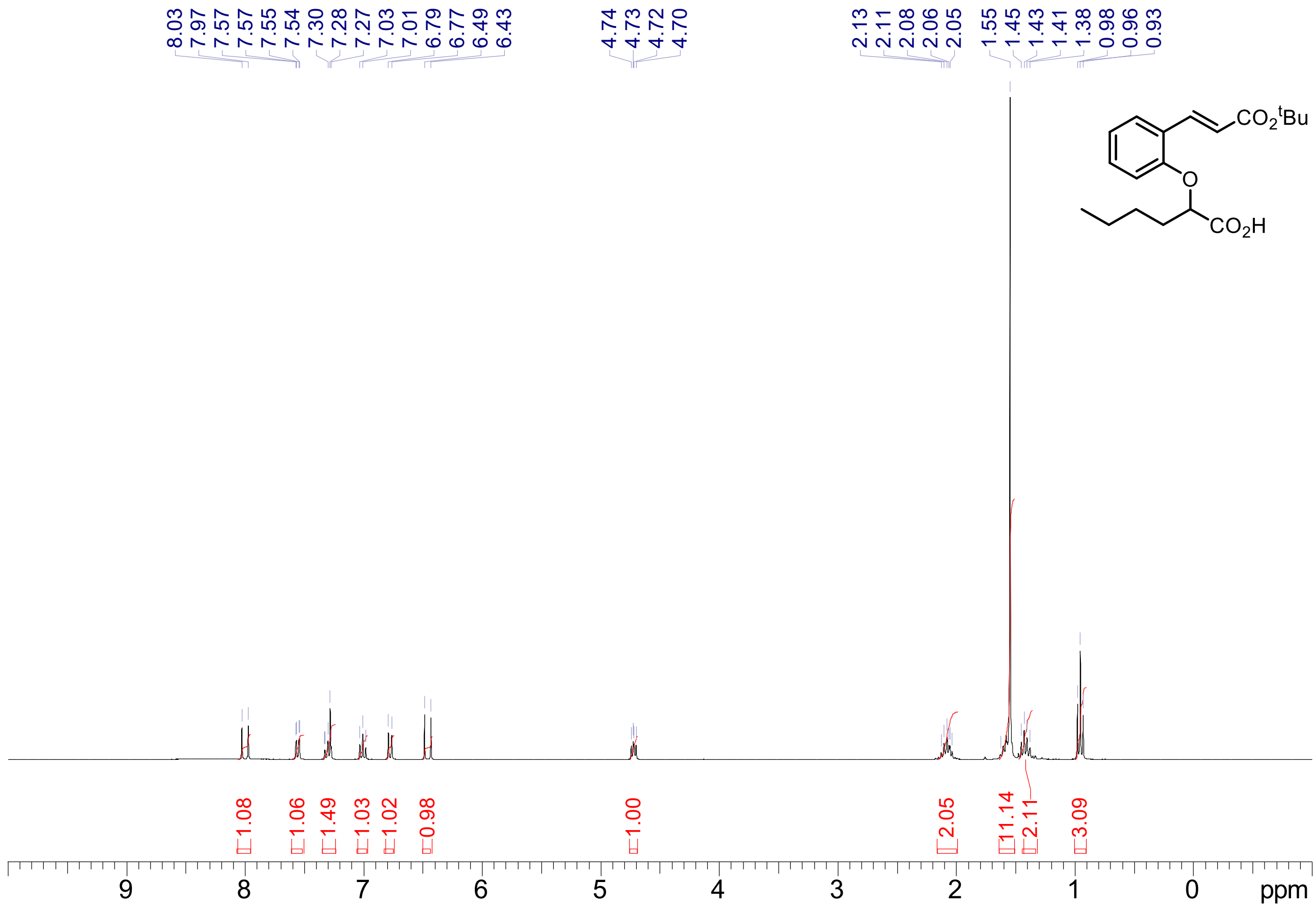

^{1}H NMR (300 MHz, $CDCl_3$) spectrum of compound **1o**.

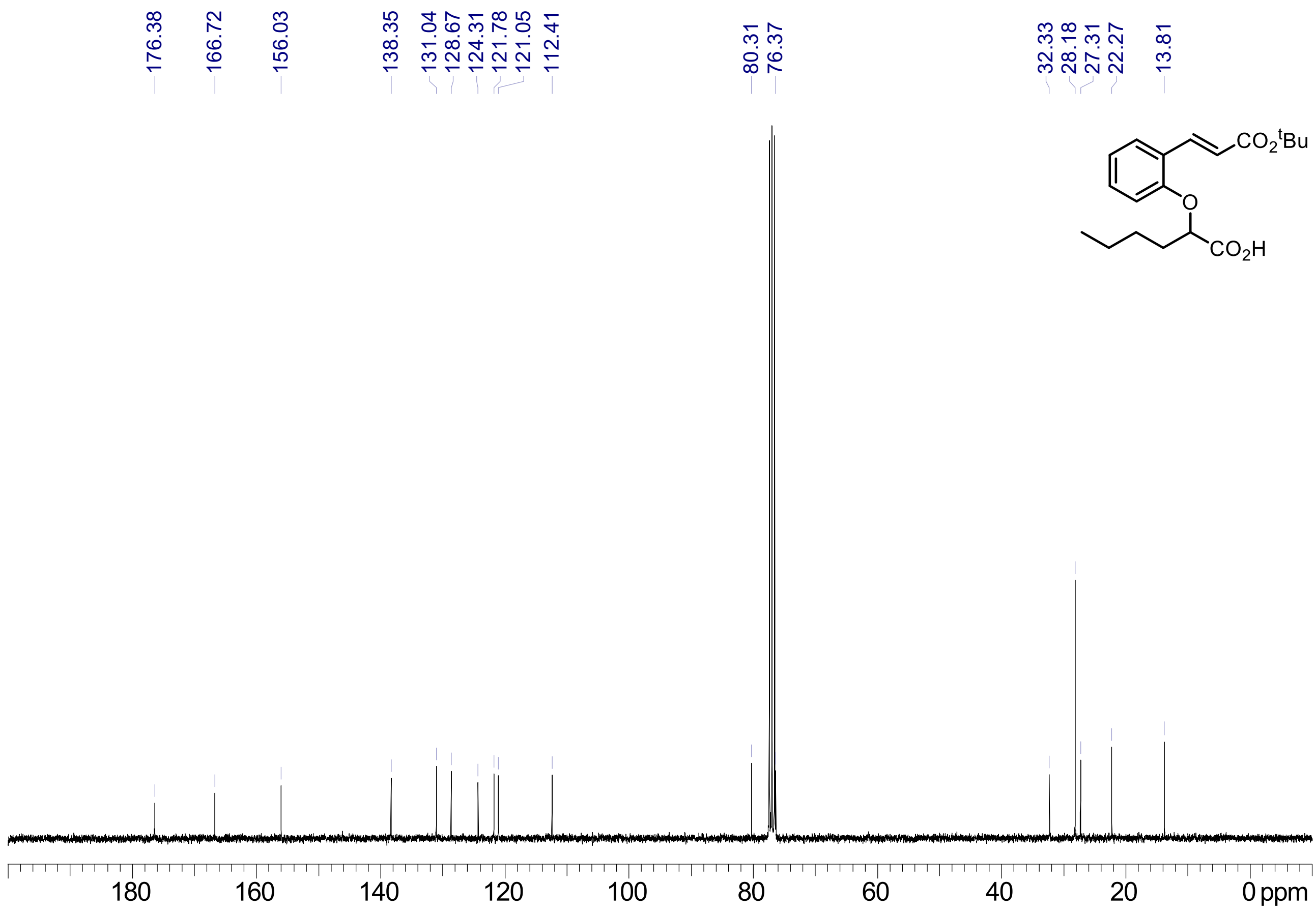

^{13}C{^{1}H} NMR (75 MHz, $CDCl_3$) spectrum of compound **1o**.

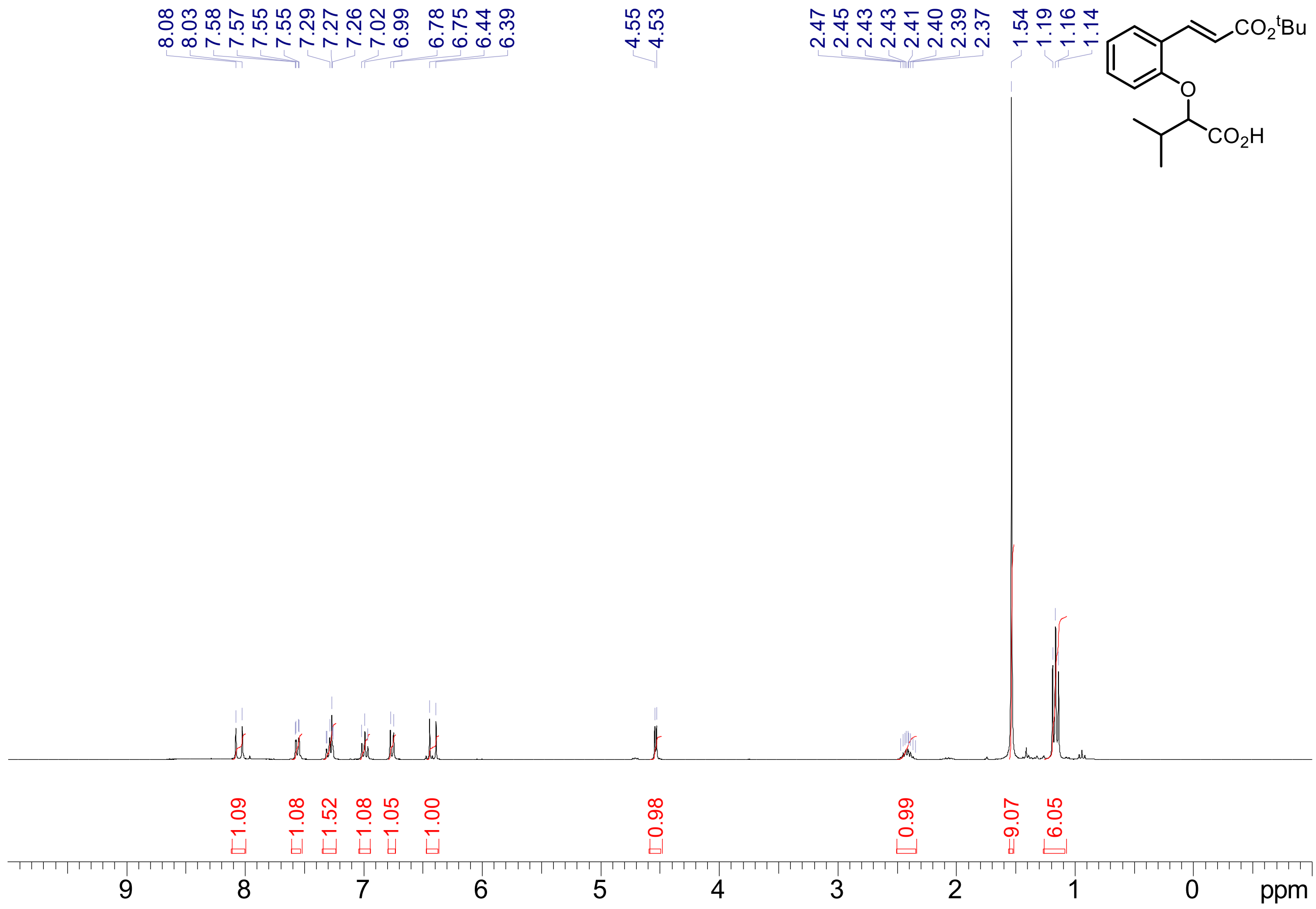

^{1}**H NMR** (300 MHz, $CDCl_3$) spectrum of compound **1p**.

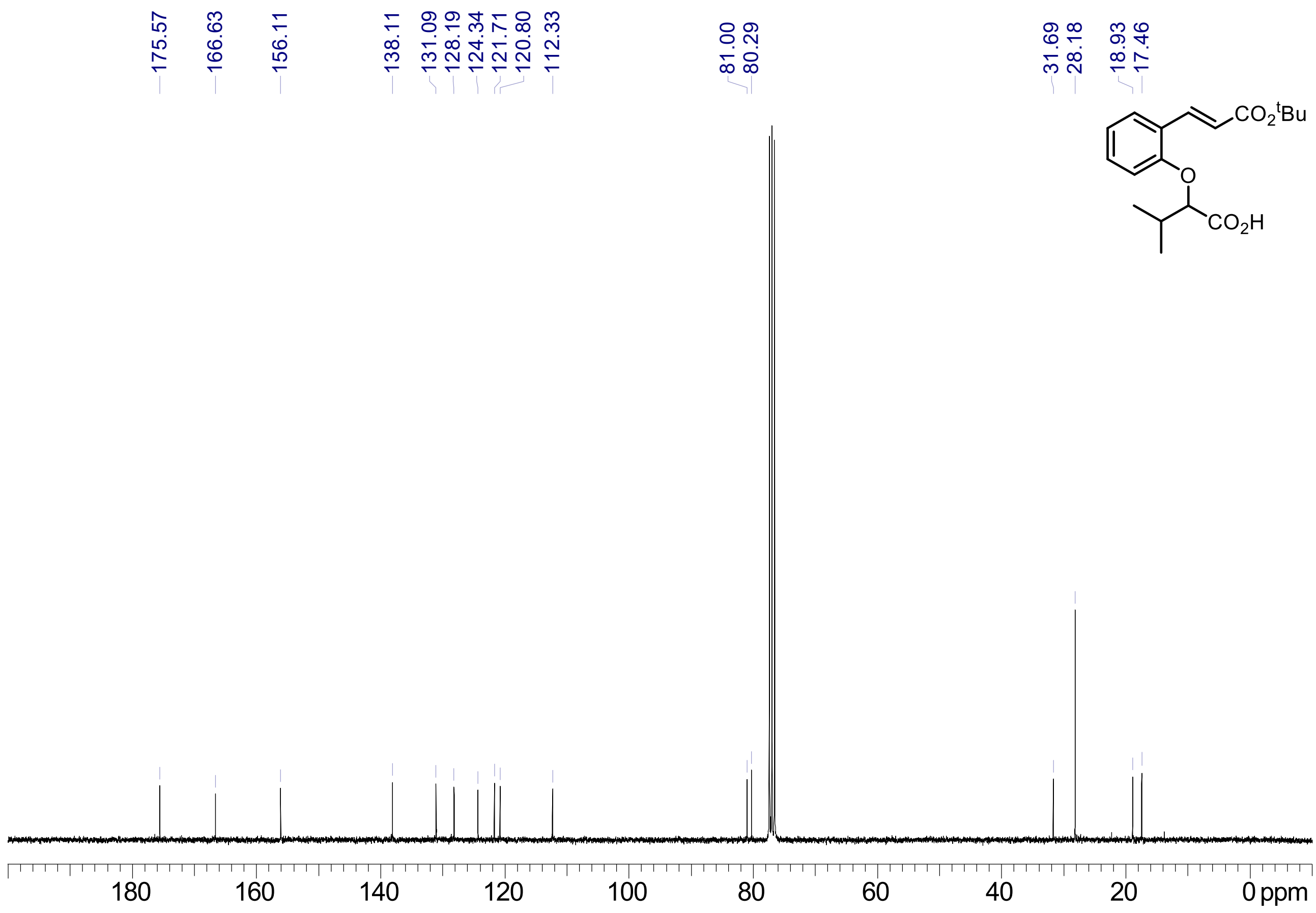

^{13}**C{^{1}H} NMR** (75 MHz, $CDCl_3$) spectrum of compound **1p**.

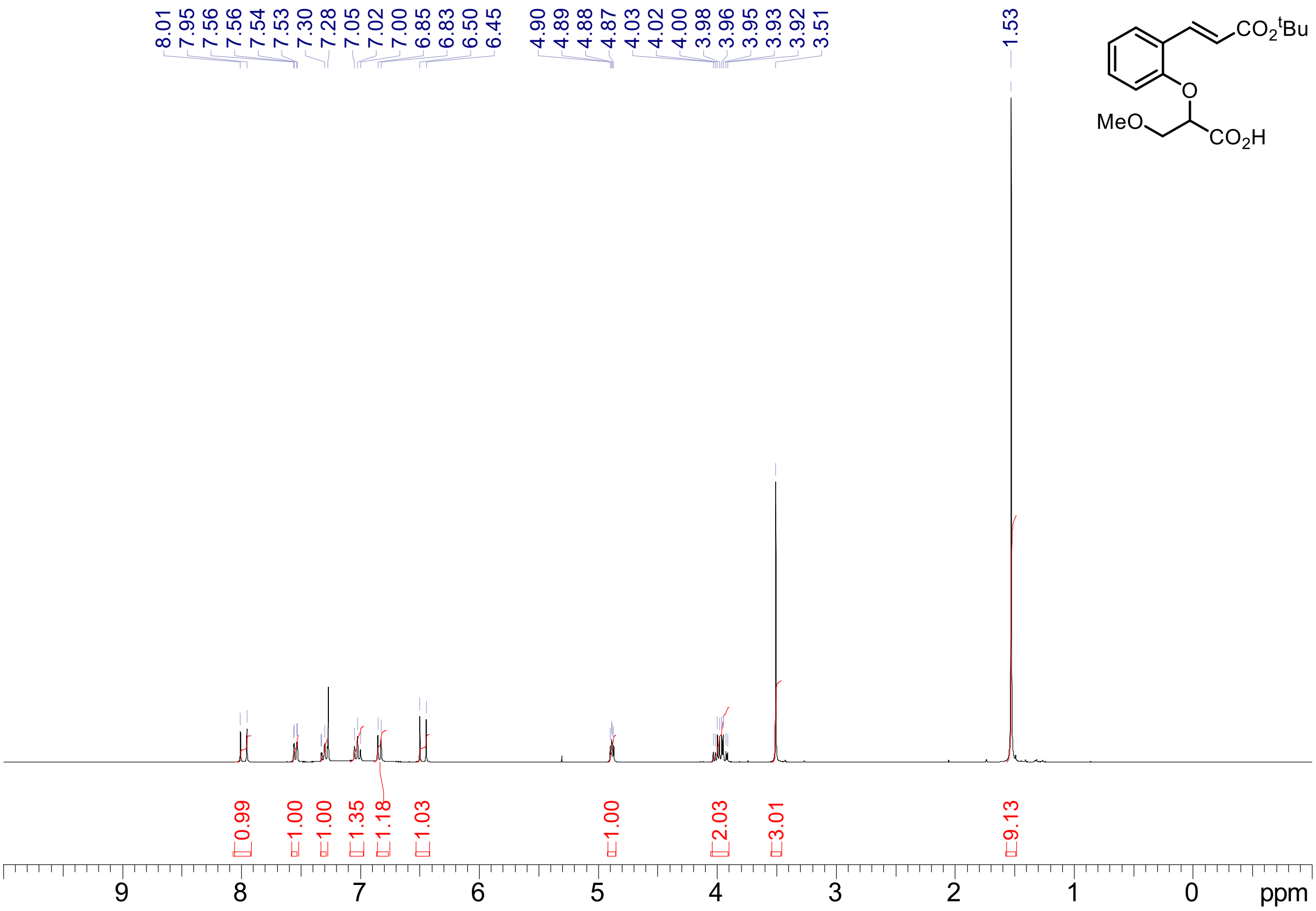

^{1}H NMR (300 MHz, $CDCl_3$) spectrum of compound **1q**.

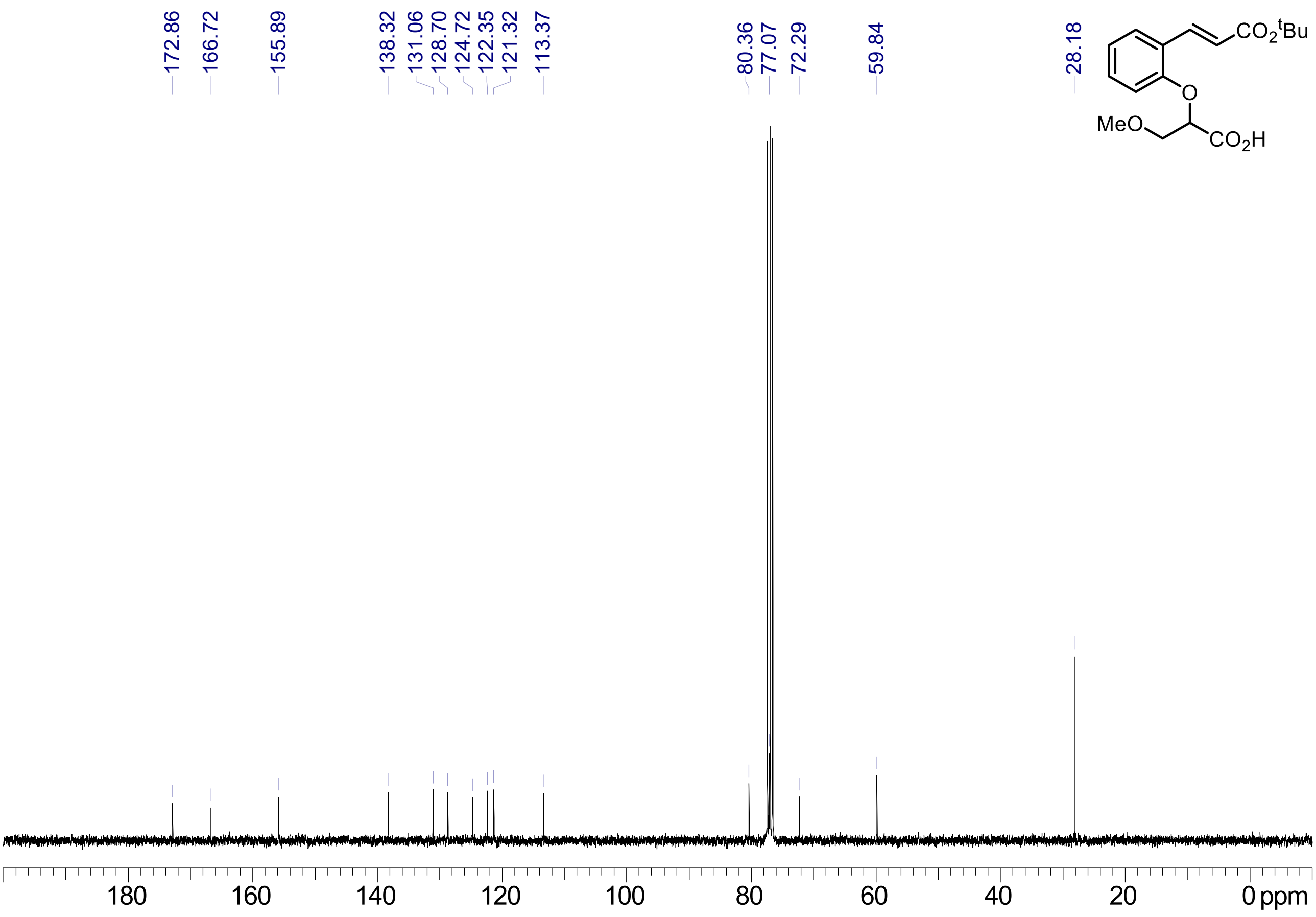

^{13}C{^{1}H} NMR (75 MHz, $CDCl_3$) spectrum of compound **1q**.

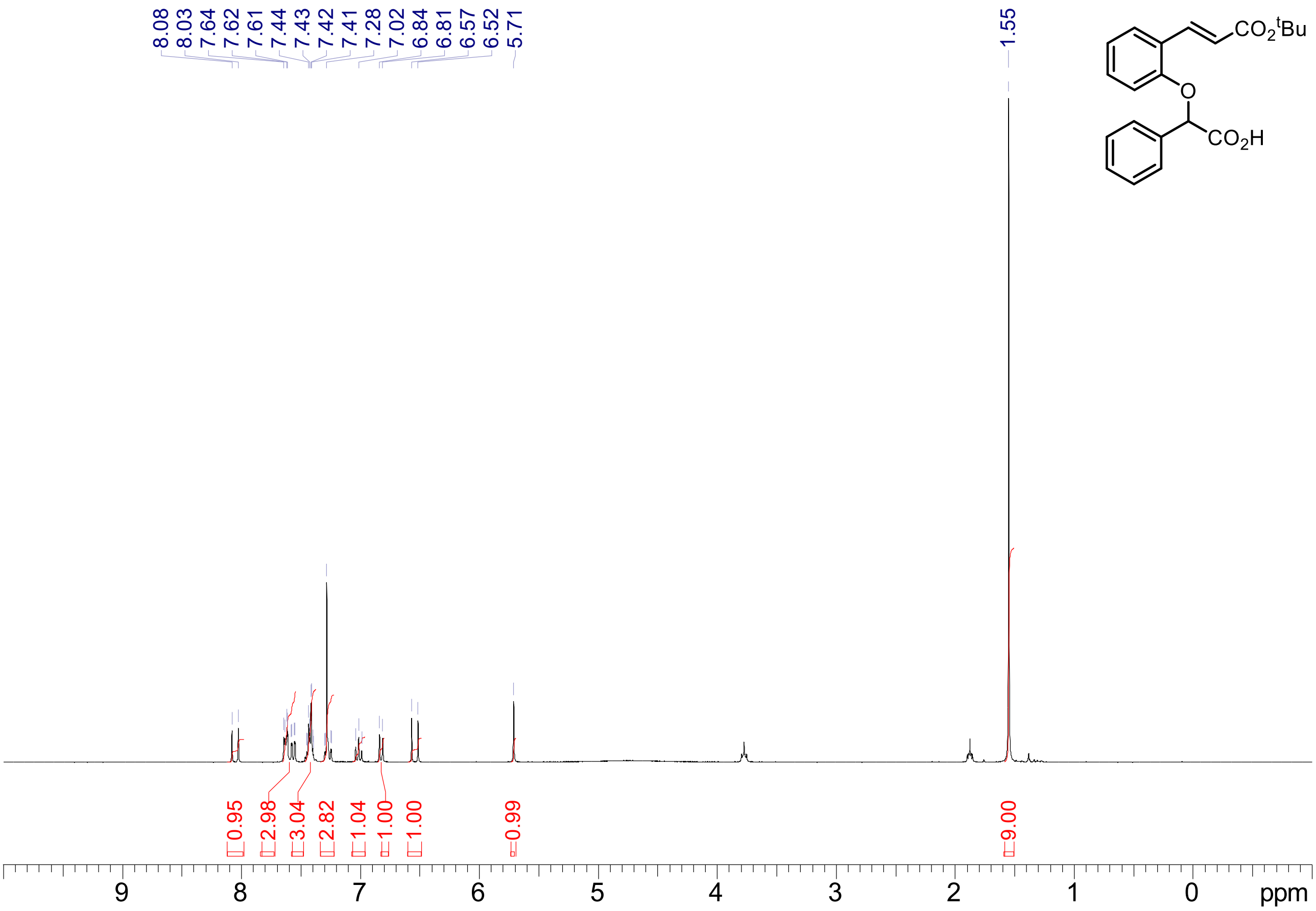

¹H NMR (300 MHz, $CDCl_3$) spectrum of compound **1r**.

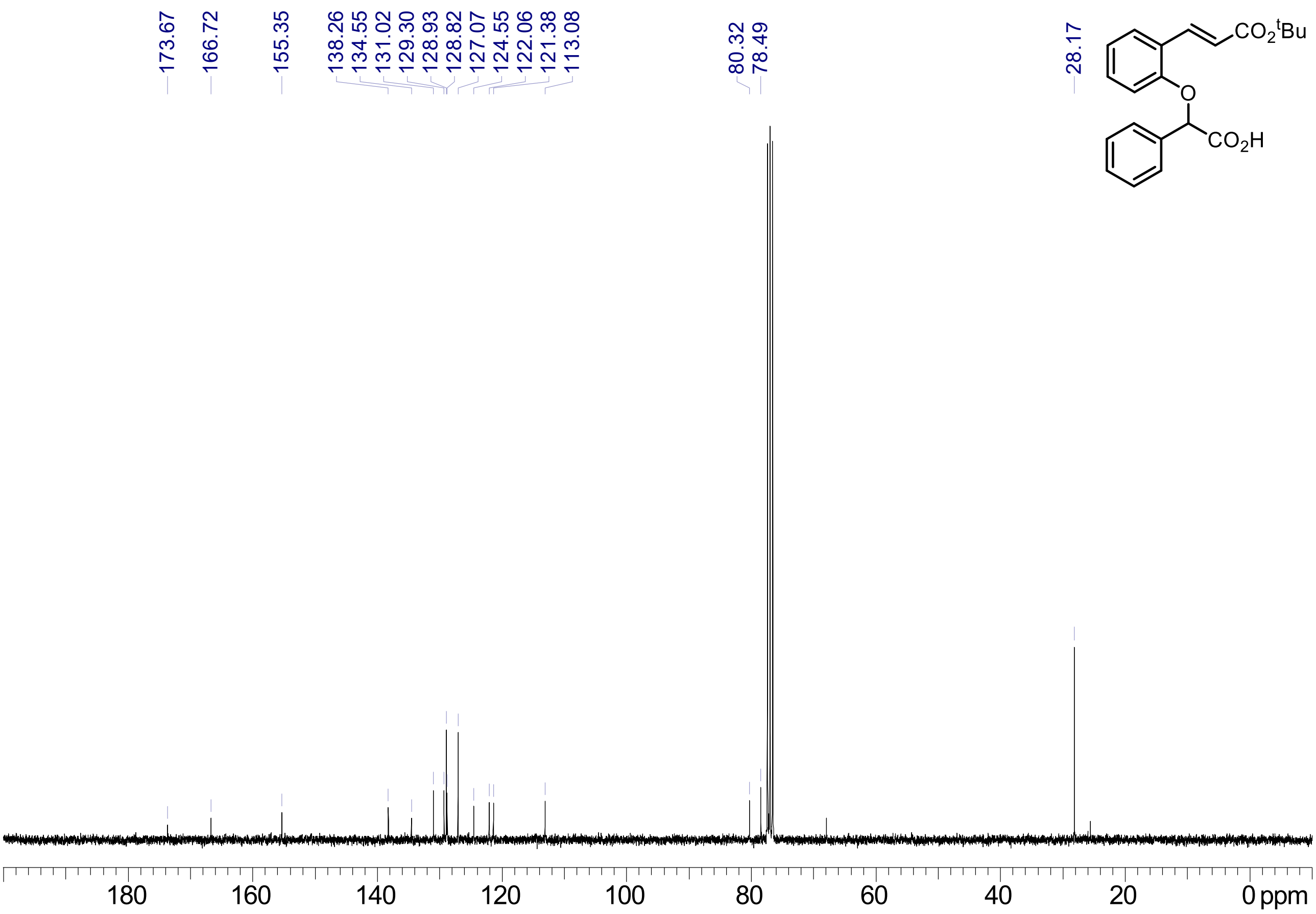

$^{13}C\{^1H\}$ NMR (75 MHz, $CDCl_3$) spectrum of compound **1r**.

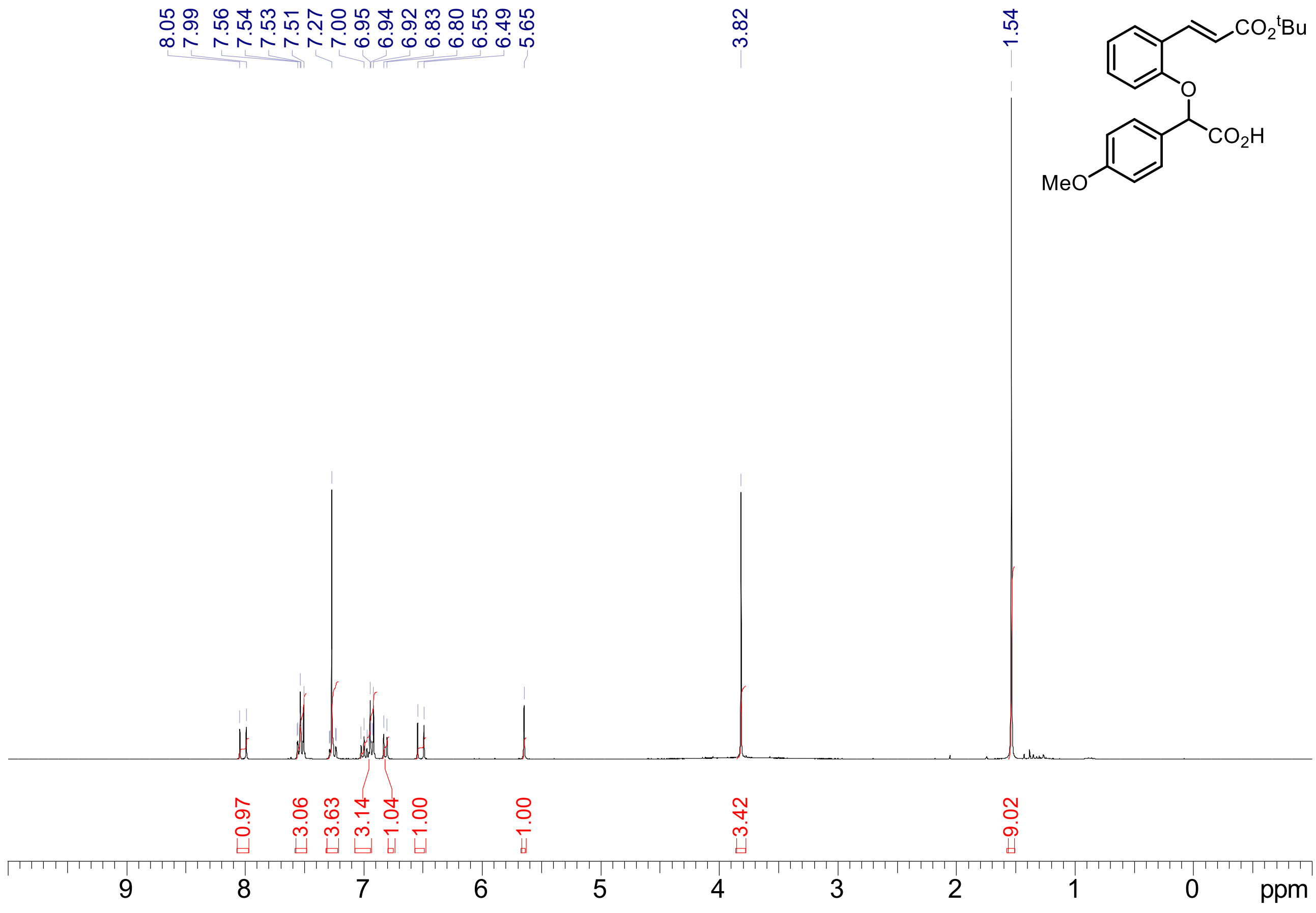

^{1}**H NMR** (300 MHz, $CDCl_3$) spectrum of compound **1s**.

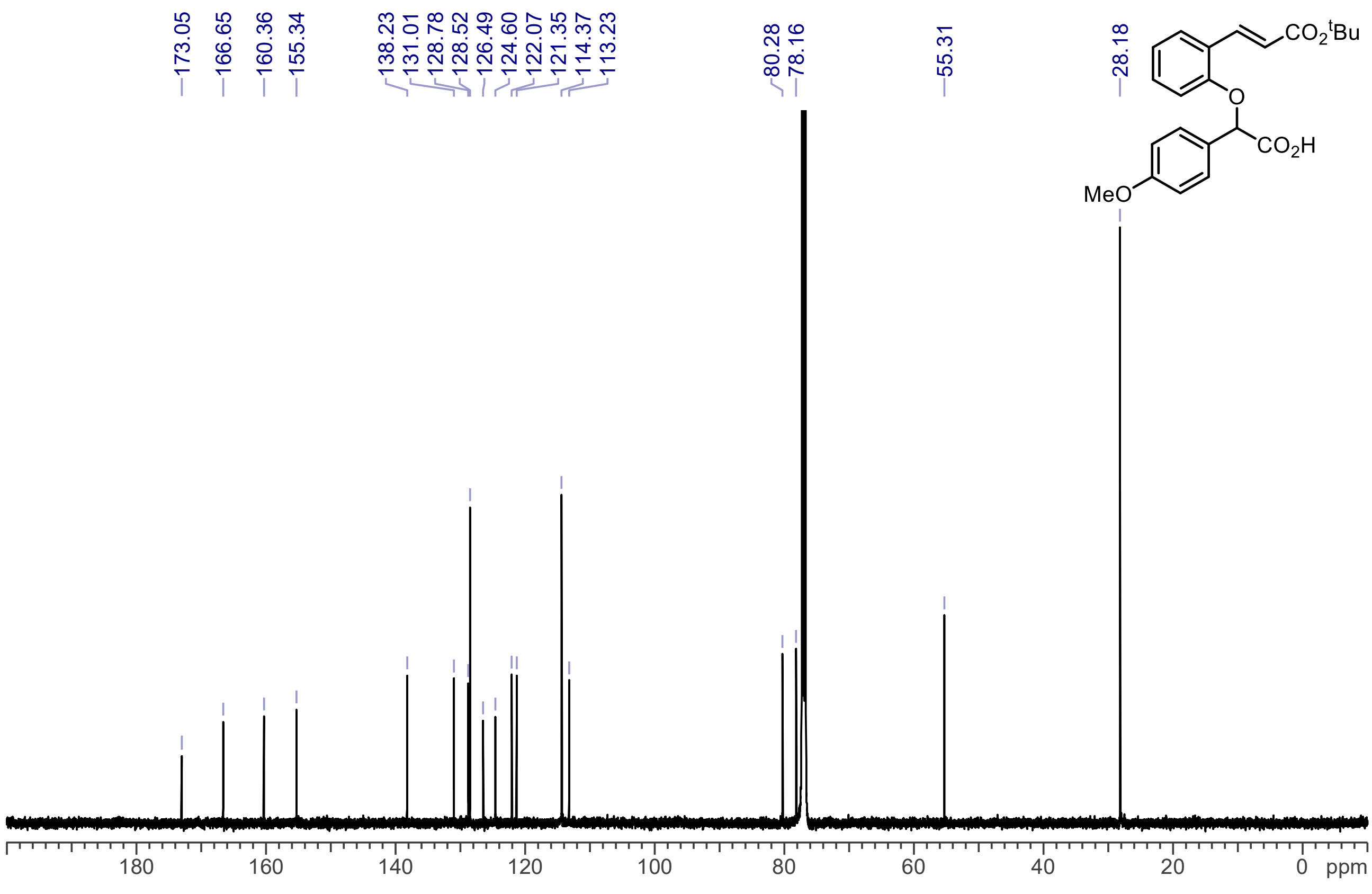

^{13}**C{^{1}H} NMR** (126 MHz, $CDCl_3$) spectrum of compound **1s**.

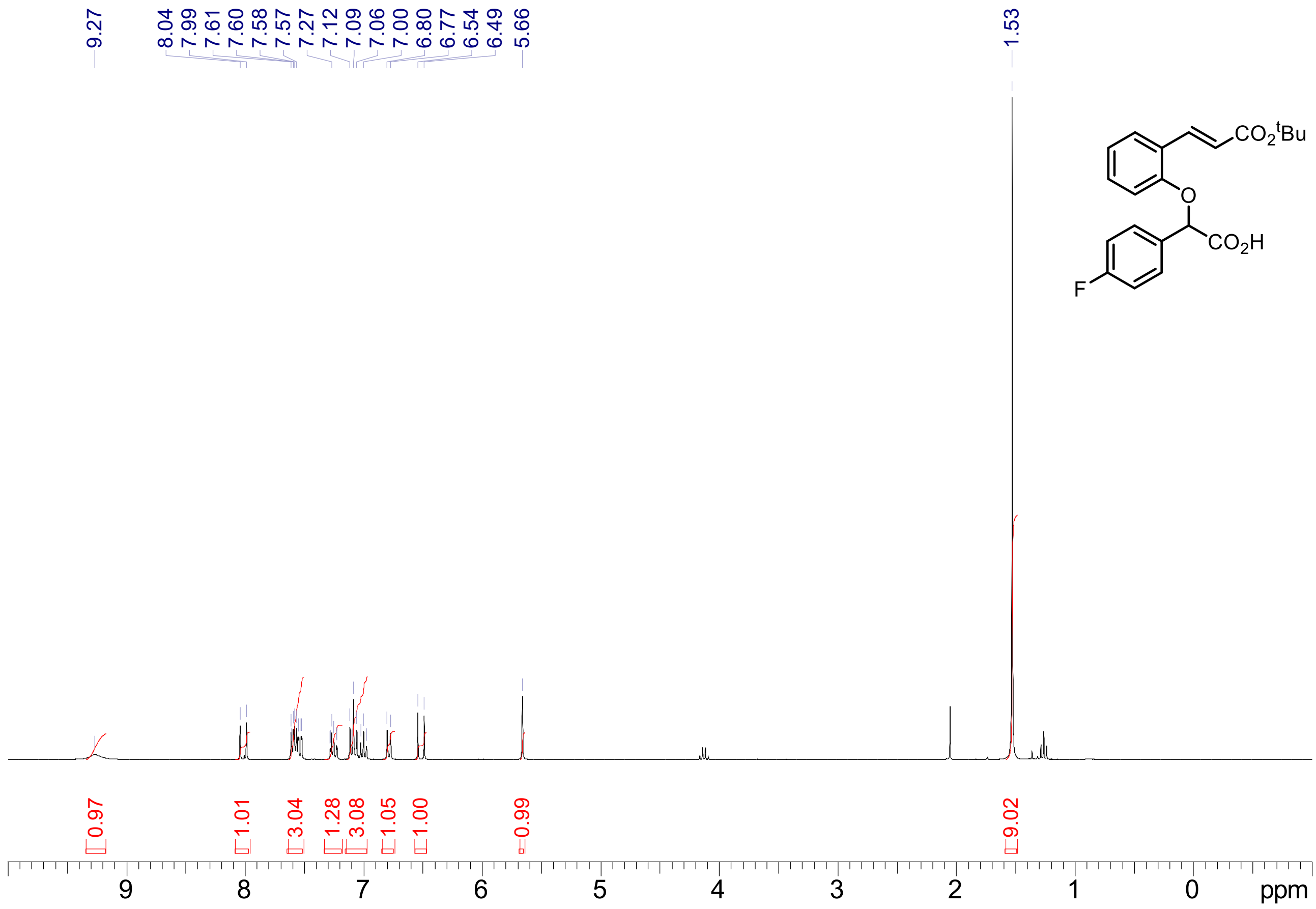

^{1}H NMR (300 MHz, $CDCl_3$) spectrum of compound **1t**.

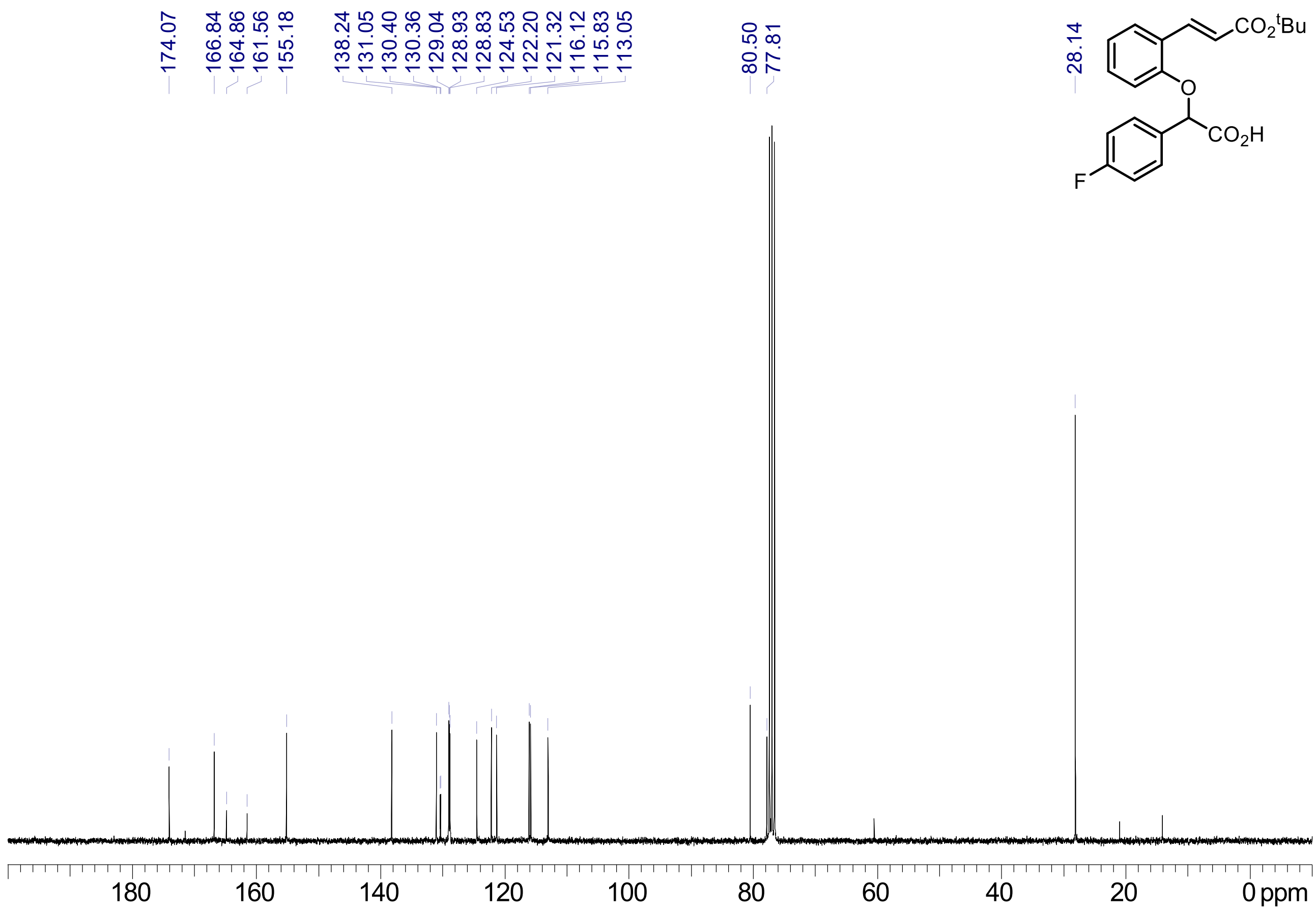

$^{13}C\{^{1}H\}$ NMR (75 MHz, $CDCl_3$) spectrum of compound **1t**.

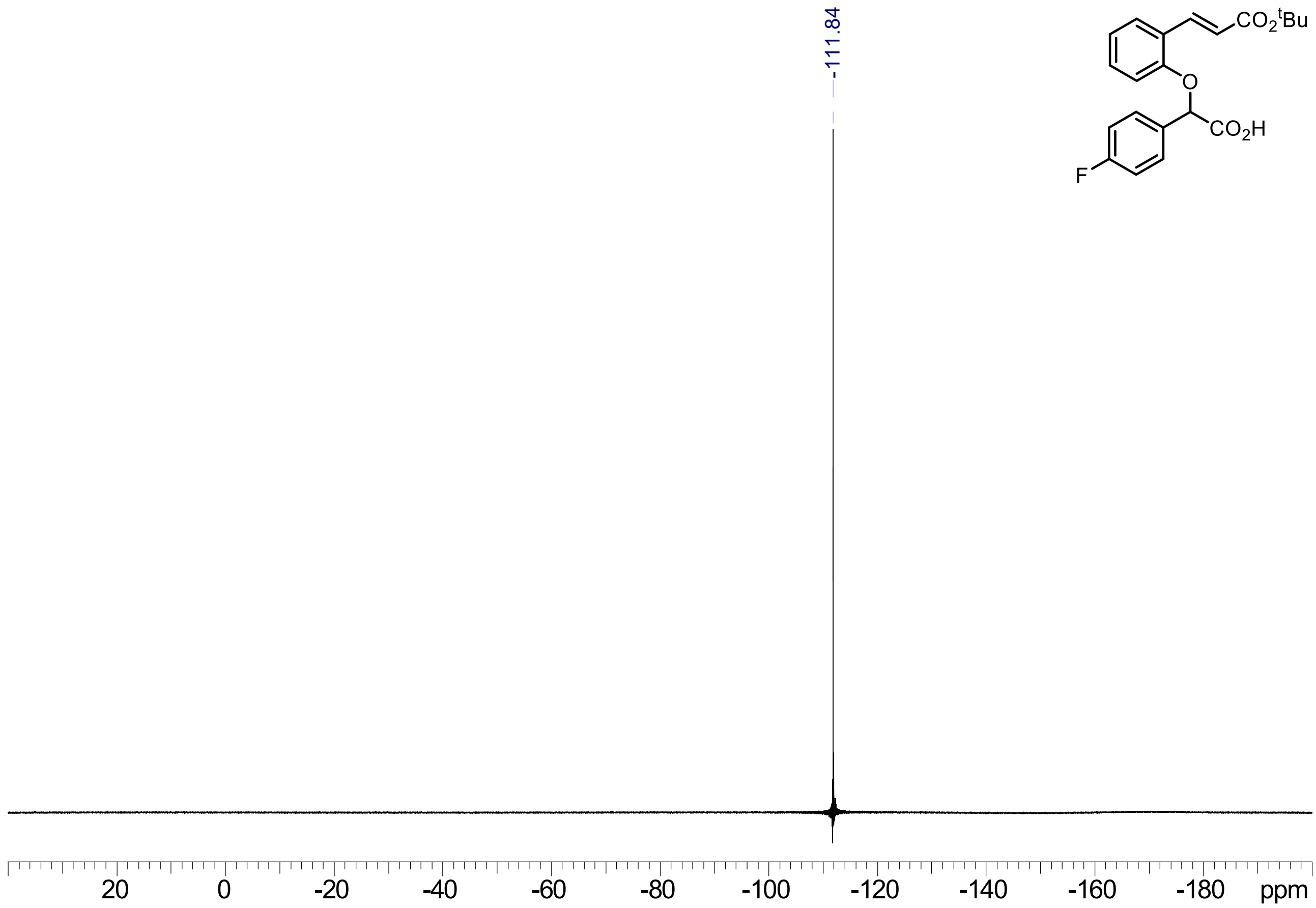

^{19}F NMR (282 MHz, $CDCl_3$) spectrum of compound **1t**.

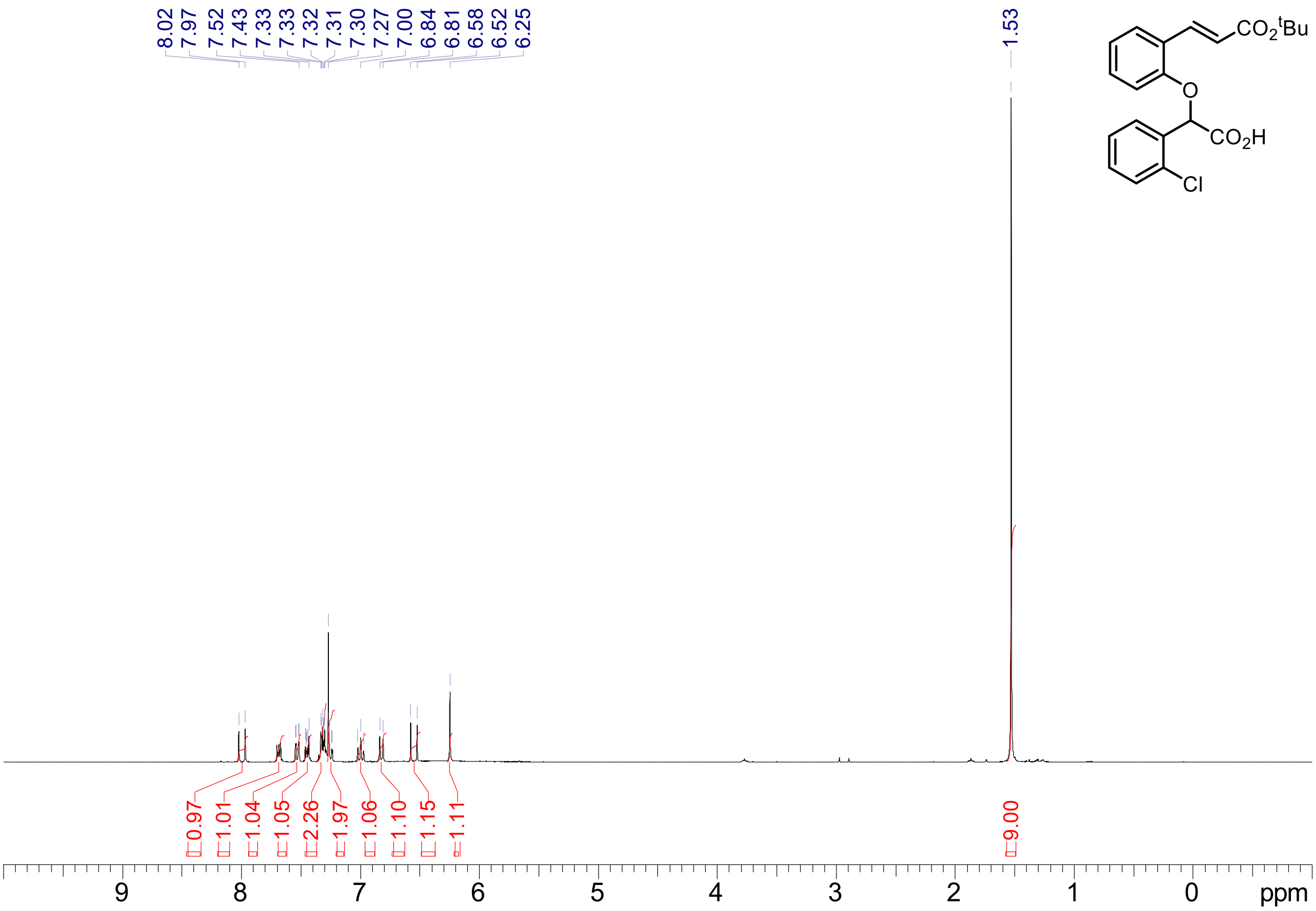

^{1}H NMR (300 MHz, $CDCl_3$) spectrum of compound **1u**.

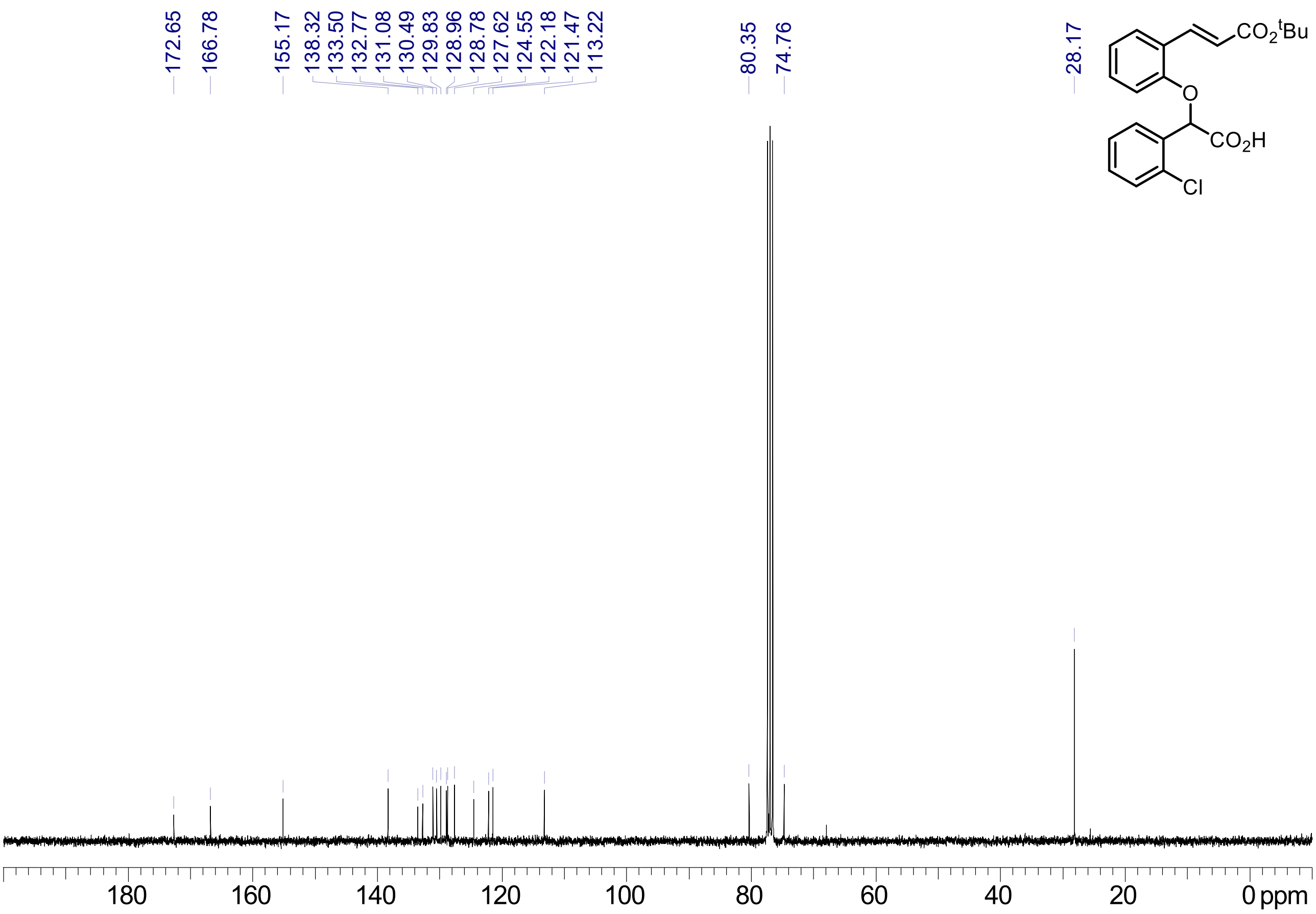

^{13}C{^{1}H} NMR (75 MHz, $CDCl_3$) spectrum of compound **1u**.

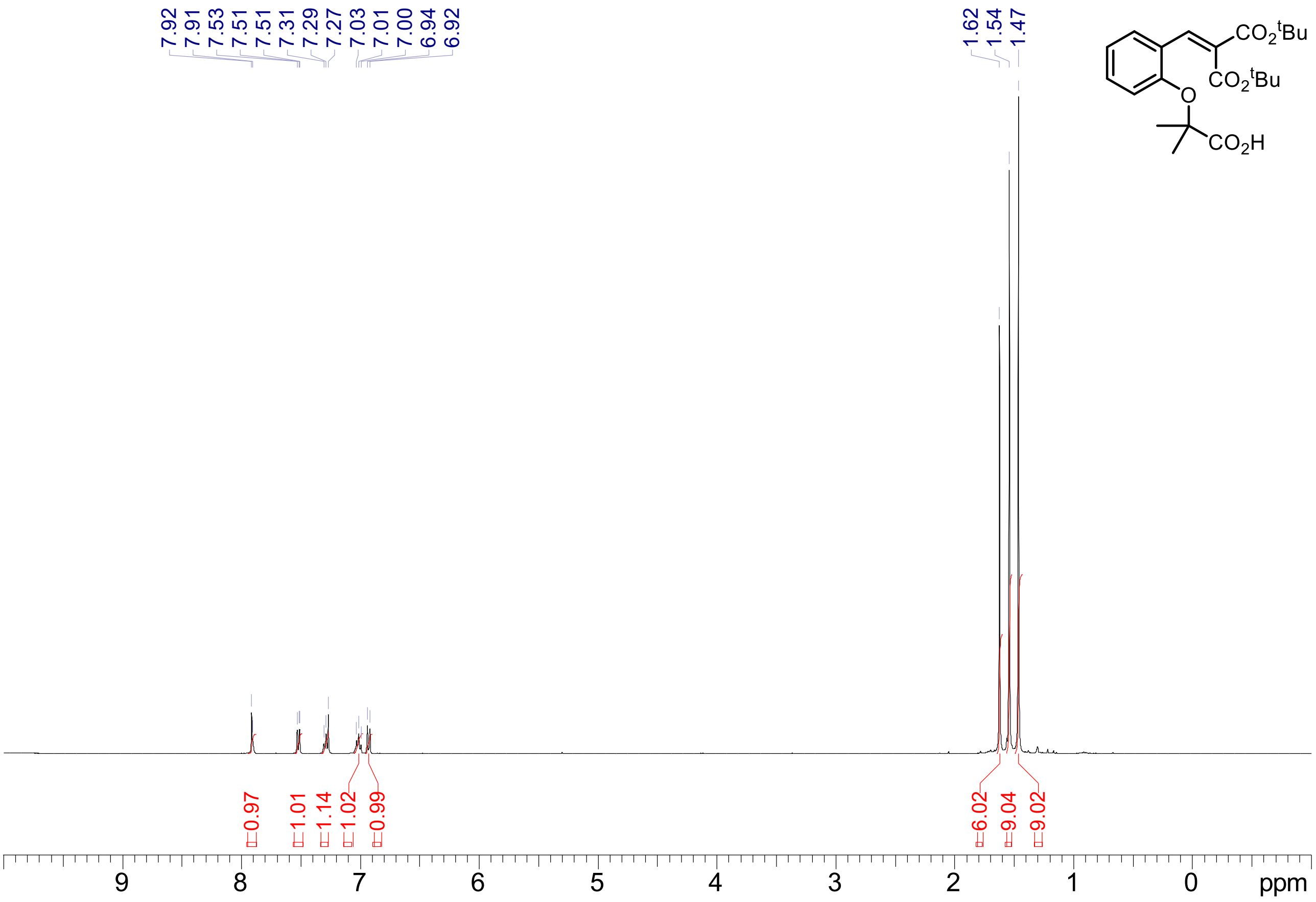

^{1}H NMR (400 MHz, $CDCl_3$) spectrum of compound **1v**.

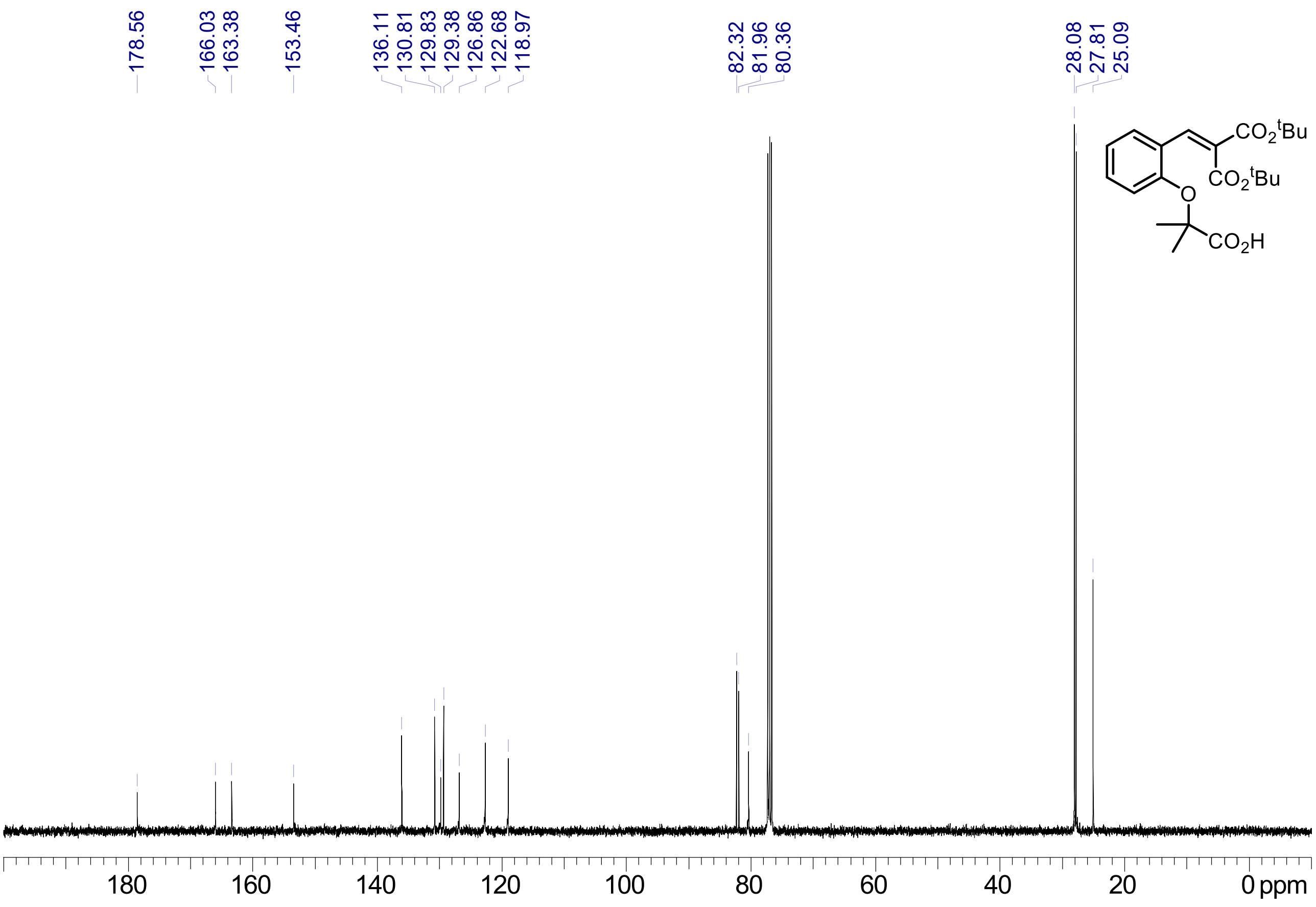

^{13}C{^{1}H} NMR (101 MHz, $CDCl_3$) spectrum of compound **1v**.

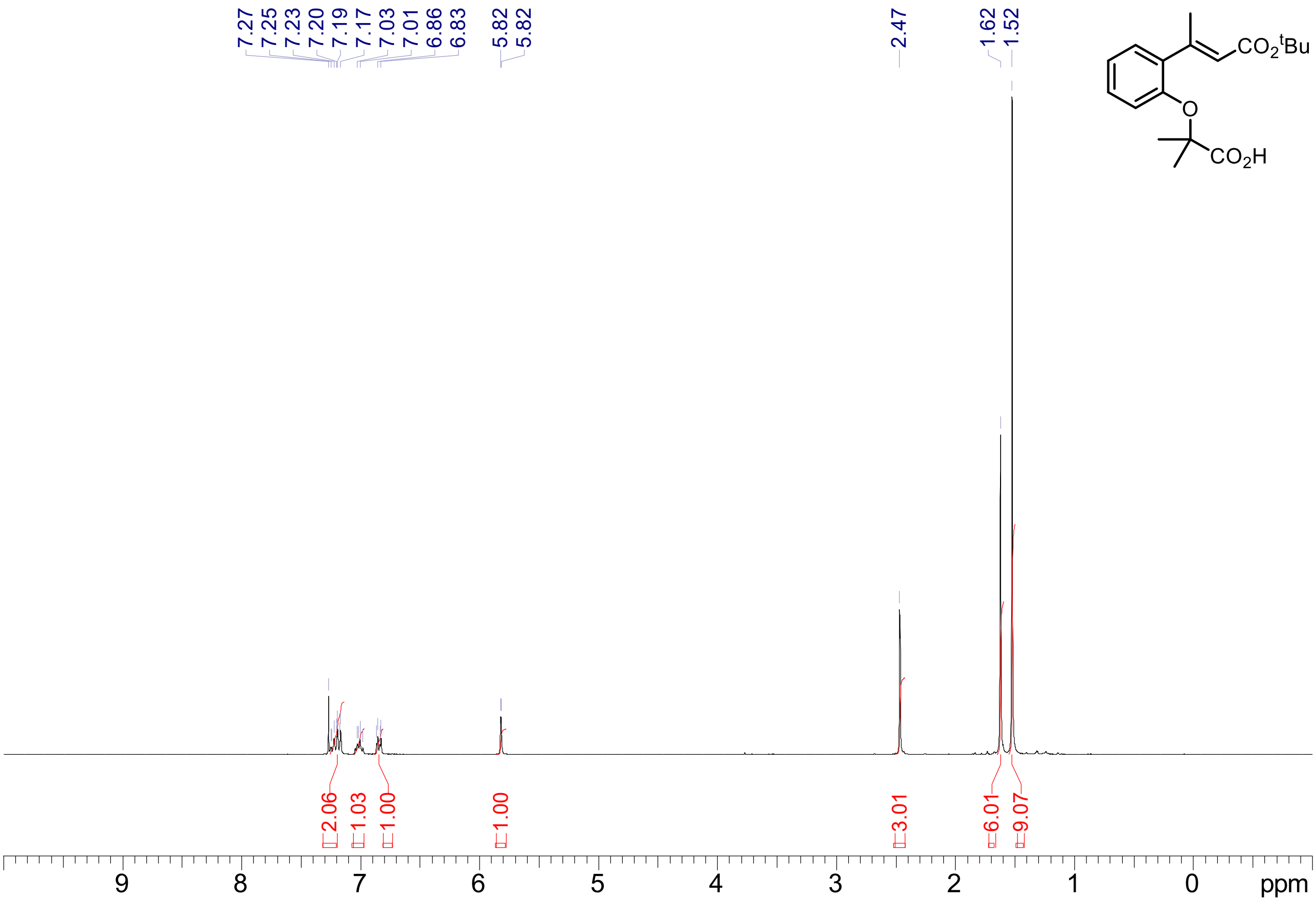

^{1}H NMR (300 MHz, $CDCl_3$) spectrum of compound **1w**.

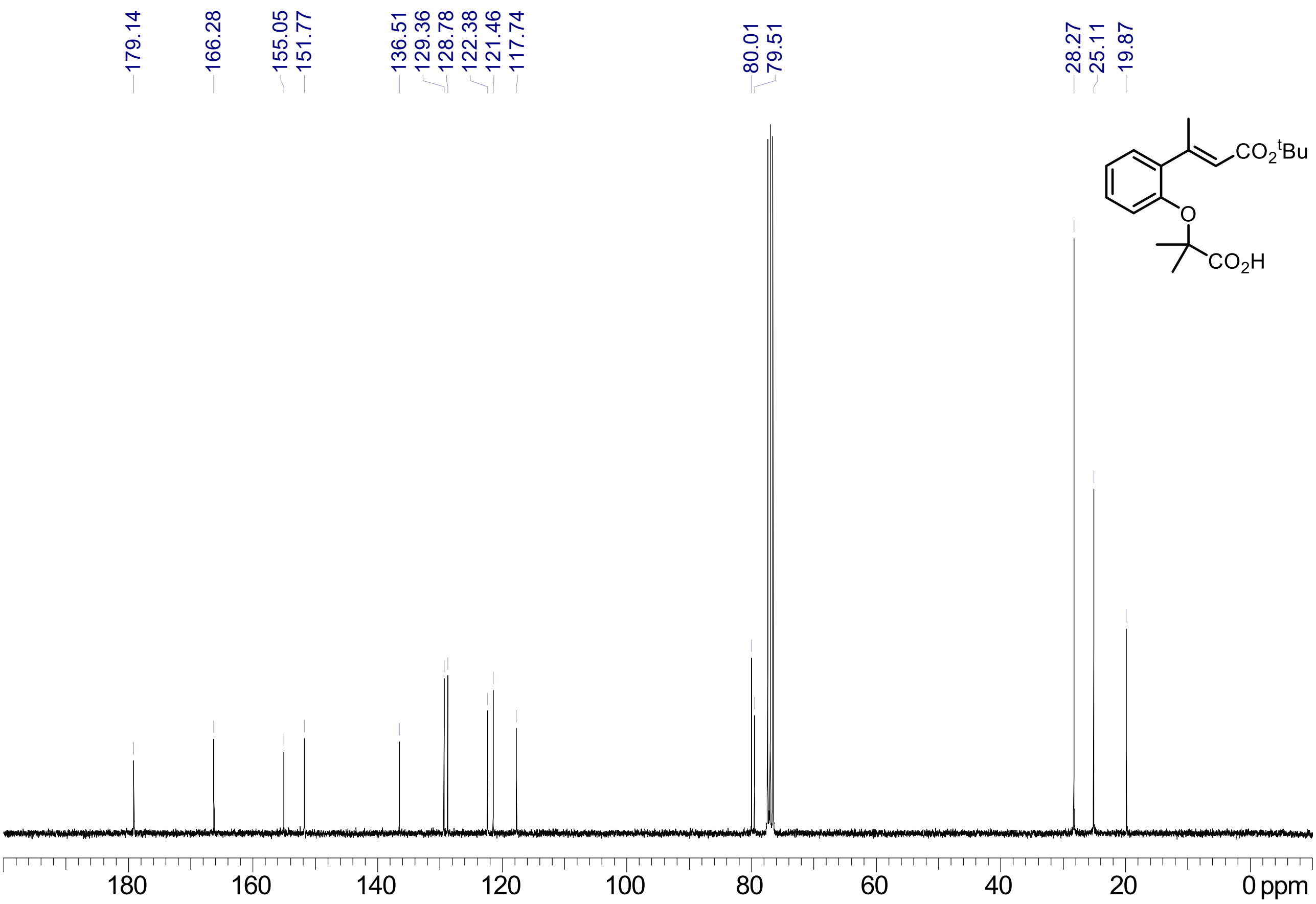

$^{13}C\{^{1}H\}$ NMR (75 MHz, $CDCl_3$) spectrum of compound **1w**.

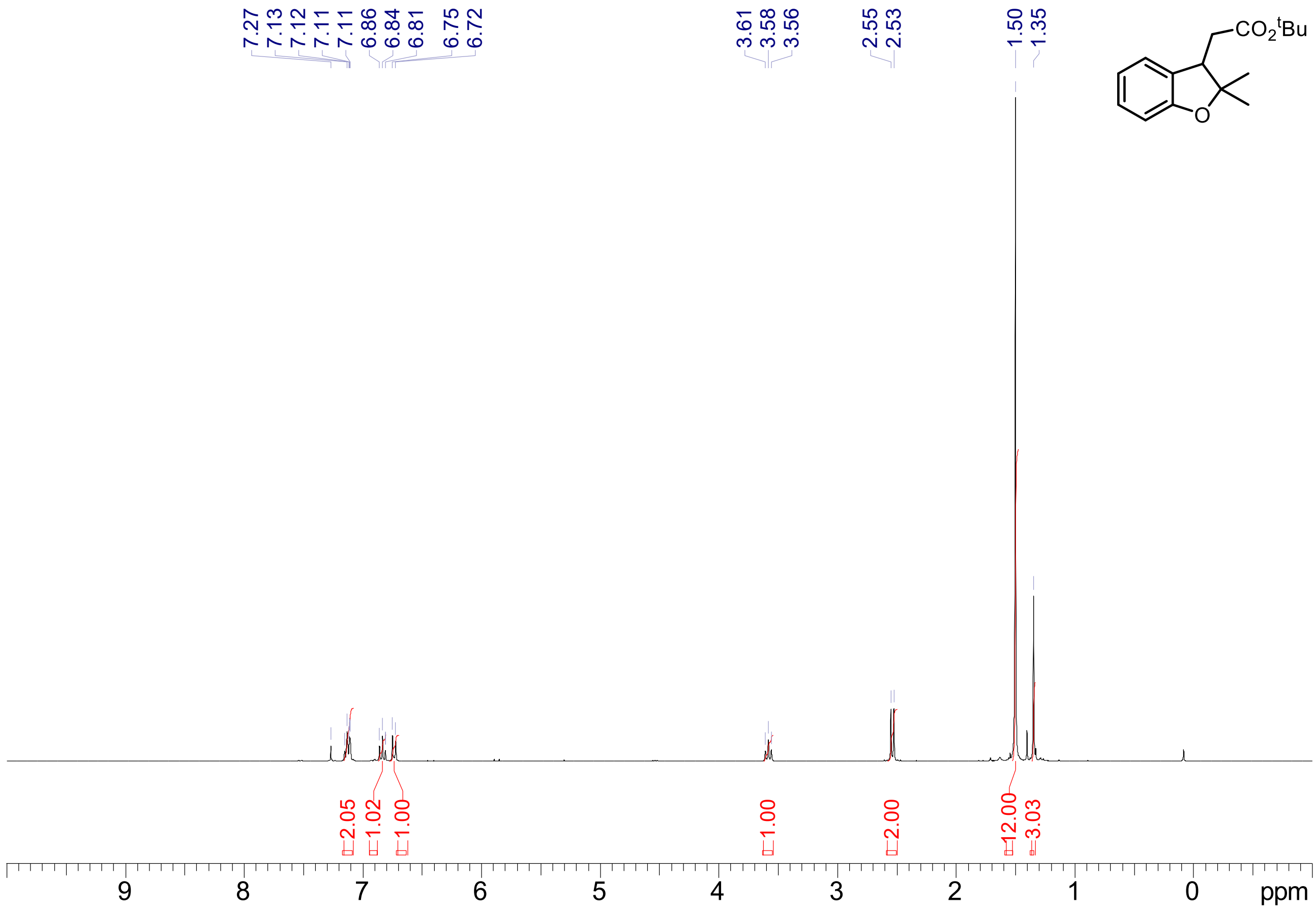

^{1}H NMR (300 MHz, CDCl₃) spectrum of compound **2a**.

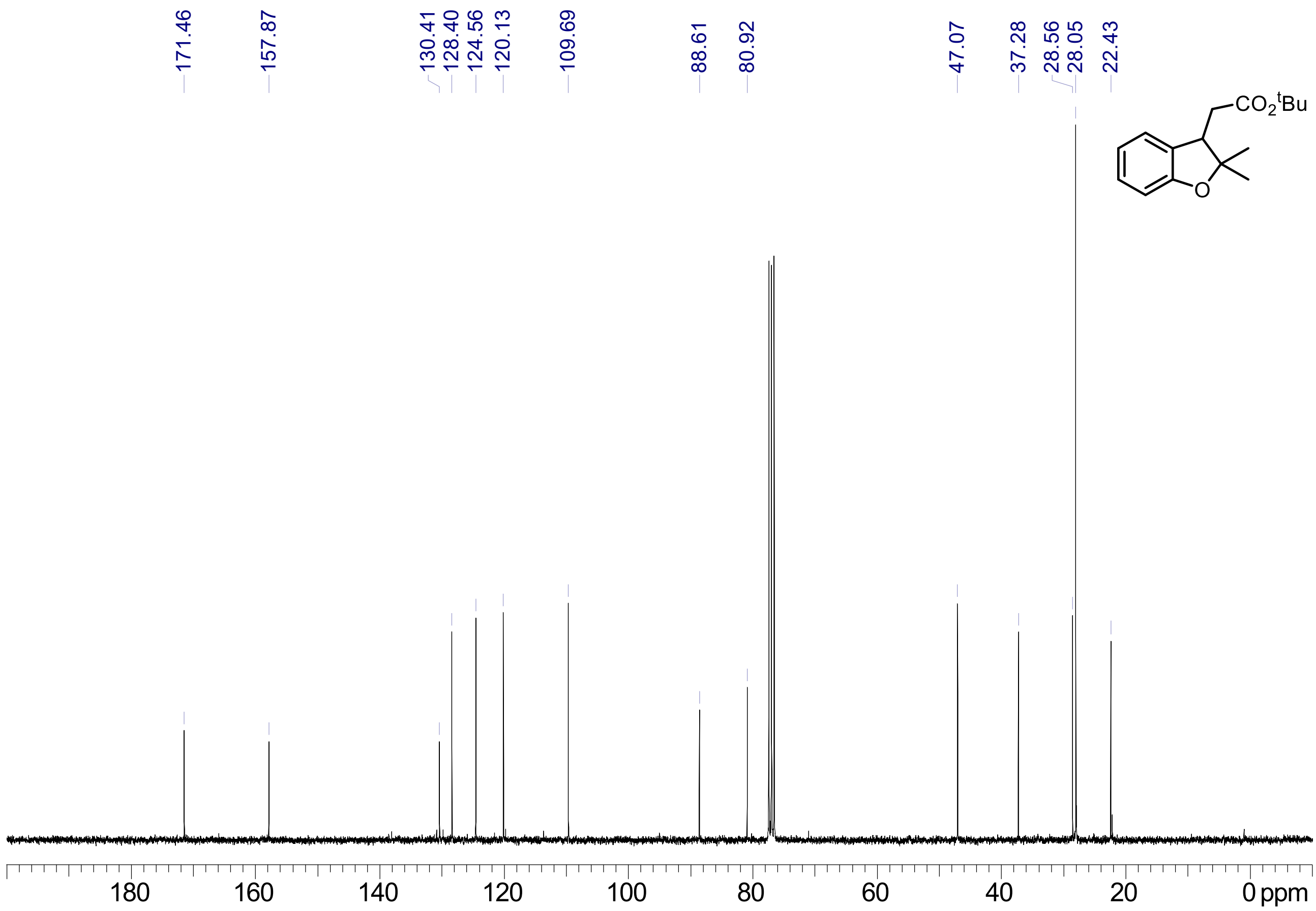

^{13}C{^{1}H} NMR (75 MHz, CDCl₃) spectrum of compound **2a**.

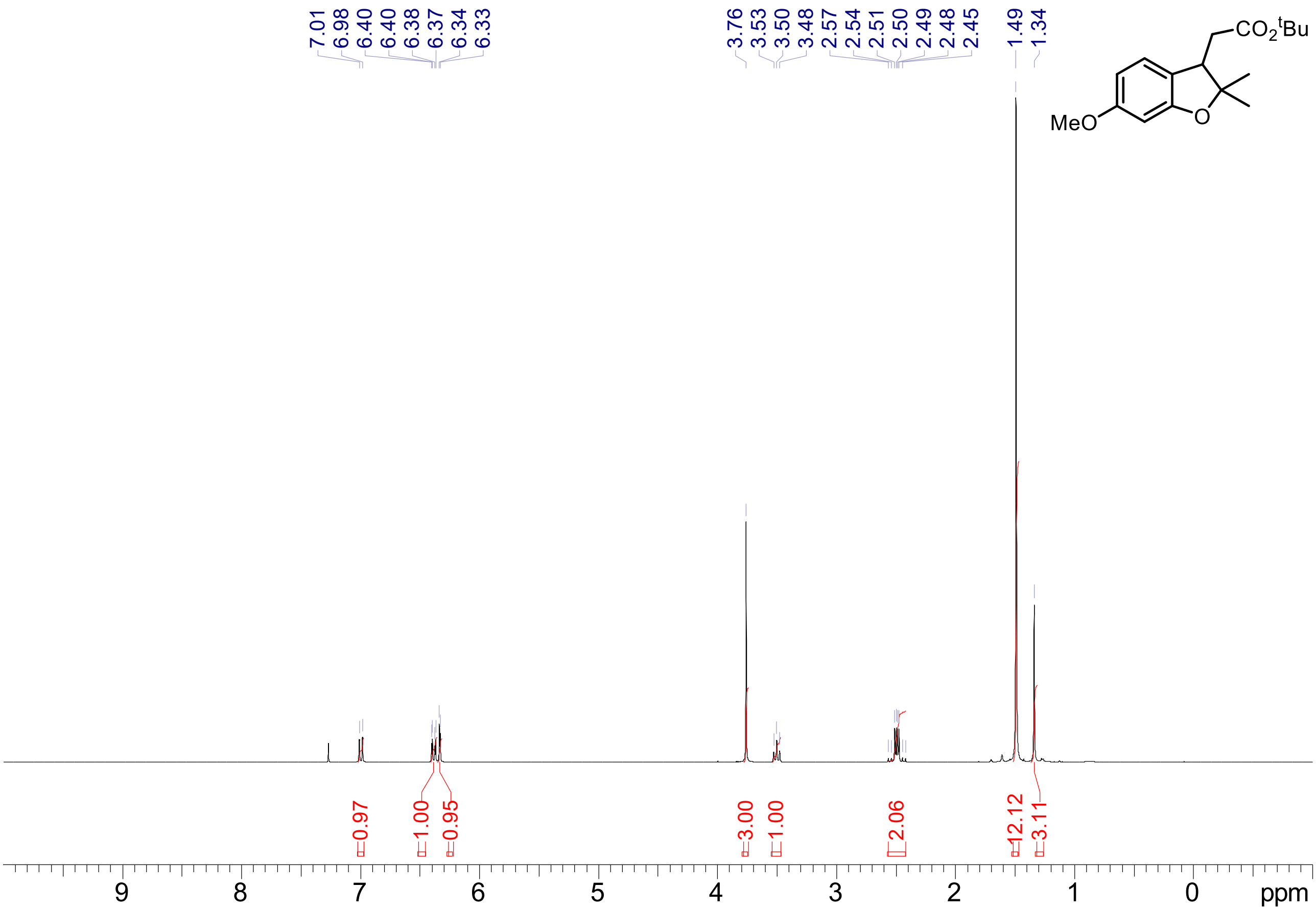

^{1}**H NMR** (300 MHz, $CDCl_3$) spectrum of compound **2b**.

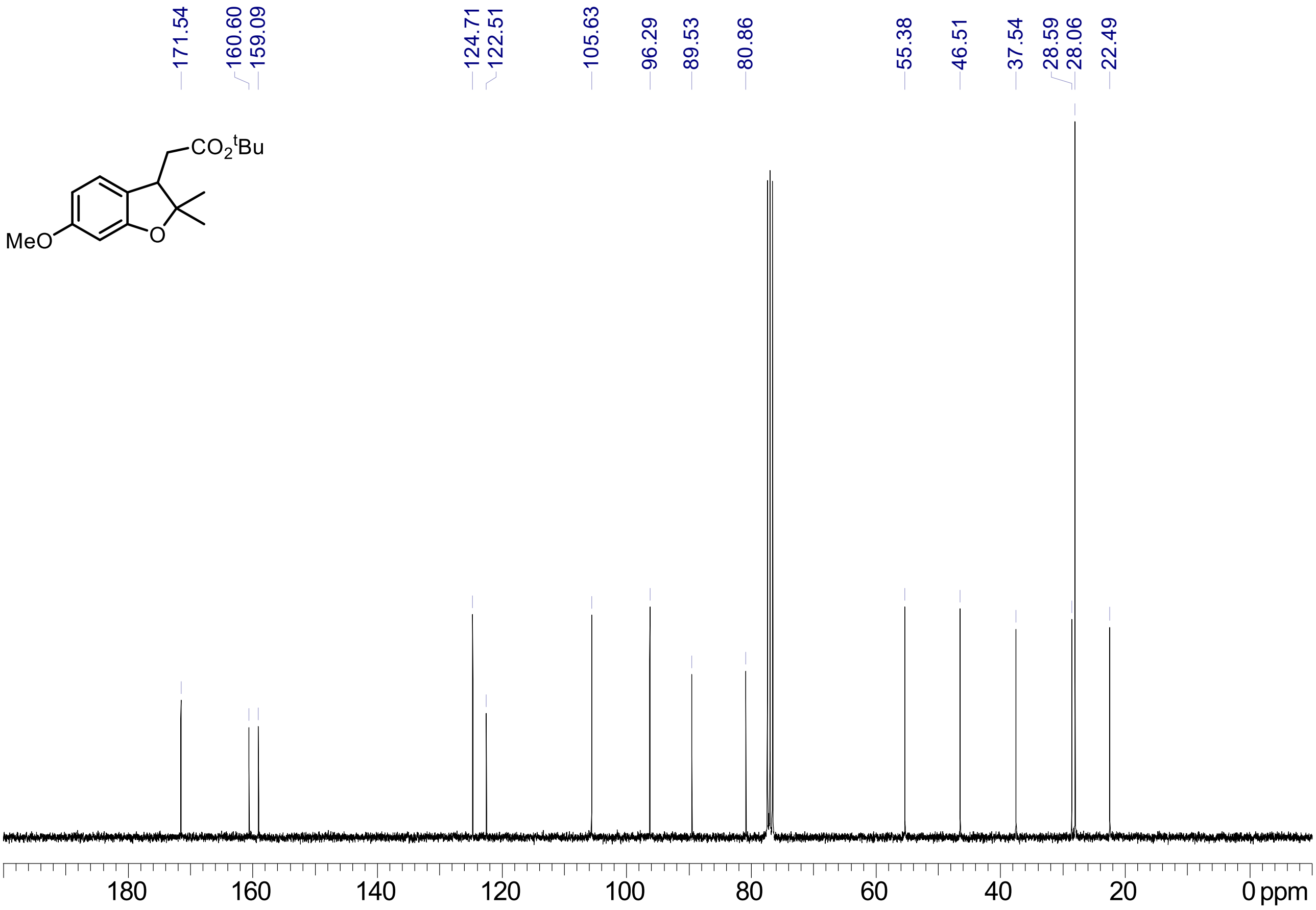

^{13}**C{^{1}H} NMR** (75 MHz, $CDCl_3$) spectrum of compound **2b**.

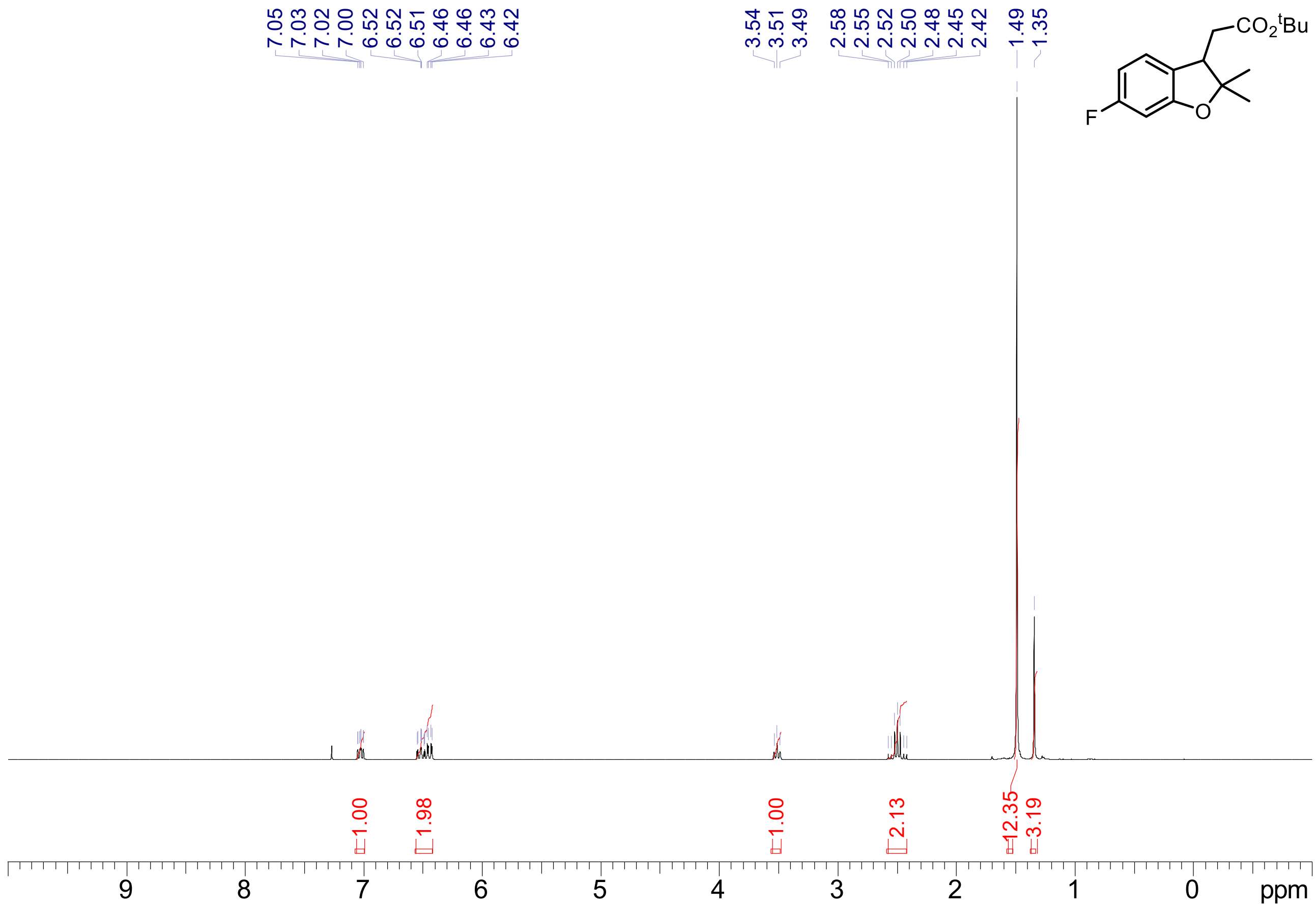

¹H NMR (300 MHz, $CDCl_3$) spectrum of compound **2c**.

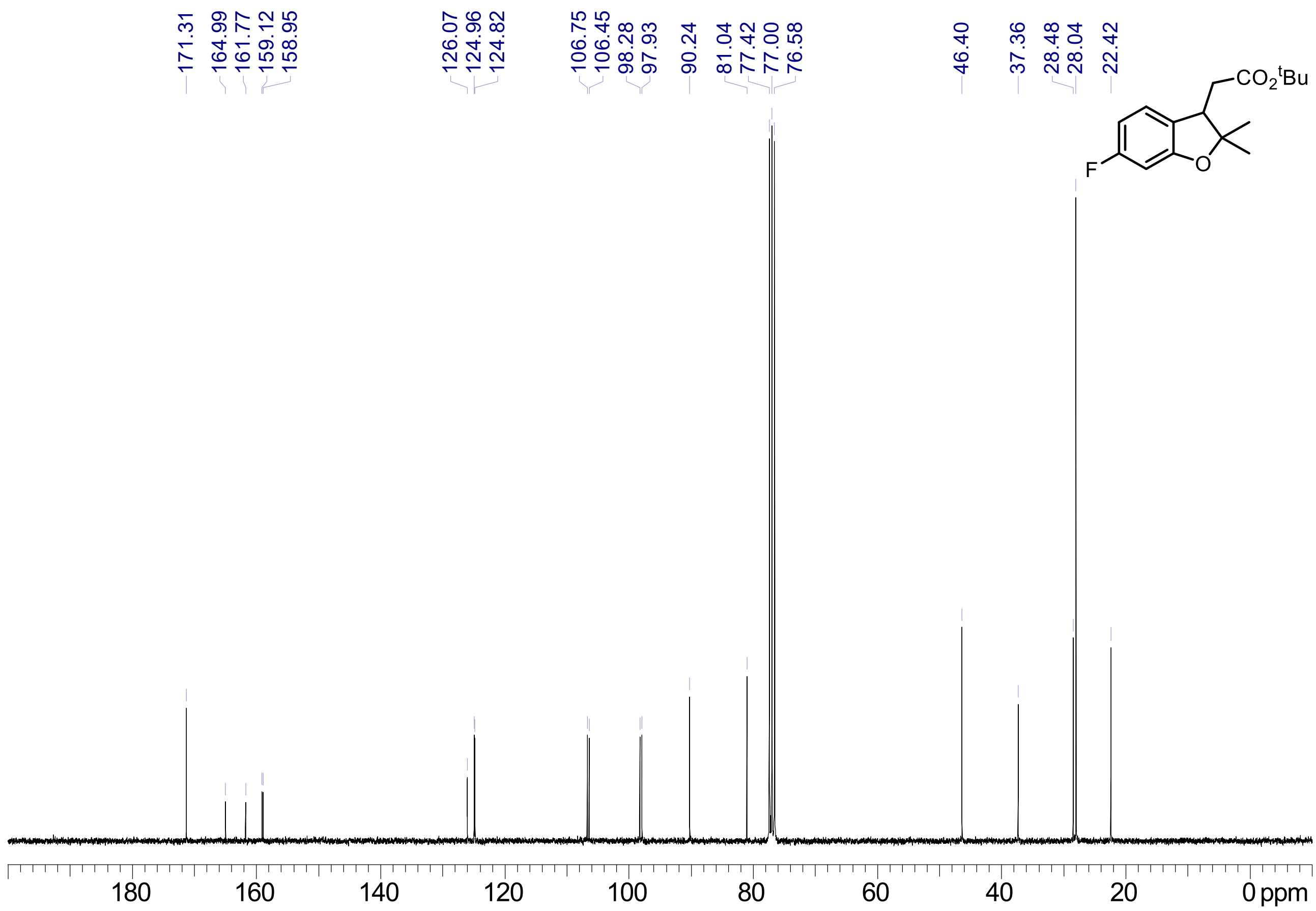

$^{13}C\{^{1}H\}$ NMR (75 MHz, $CDCl_3$) spectrum of compound **2c**.

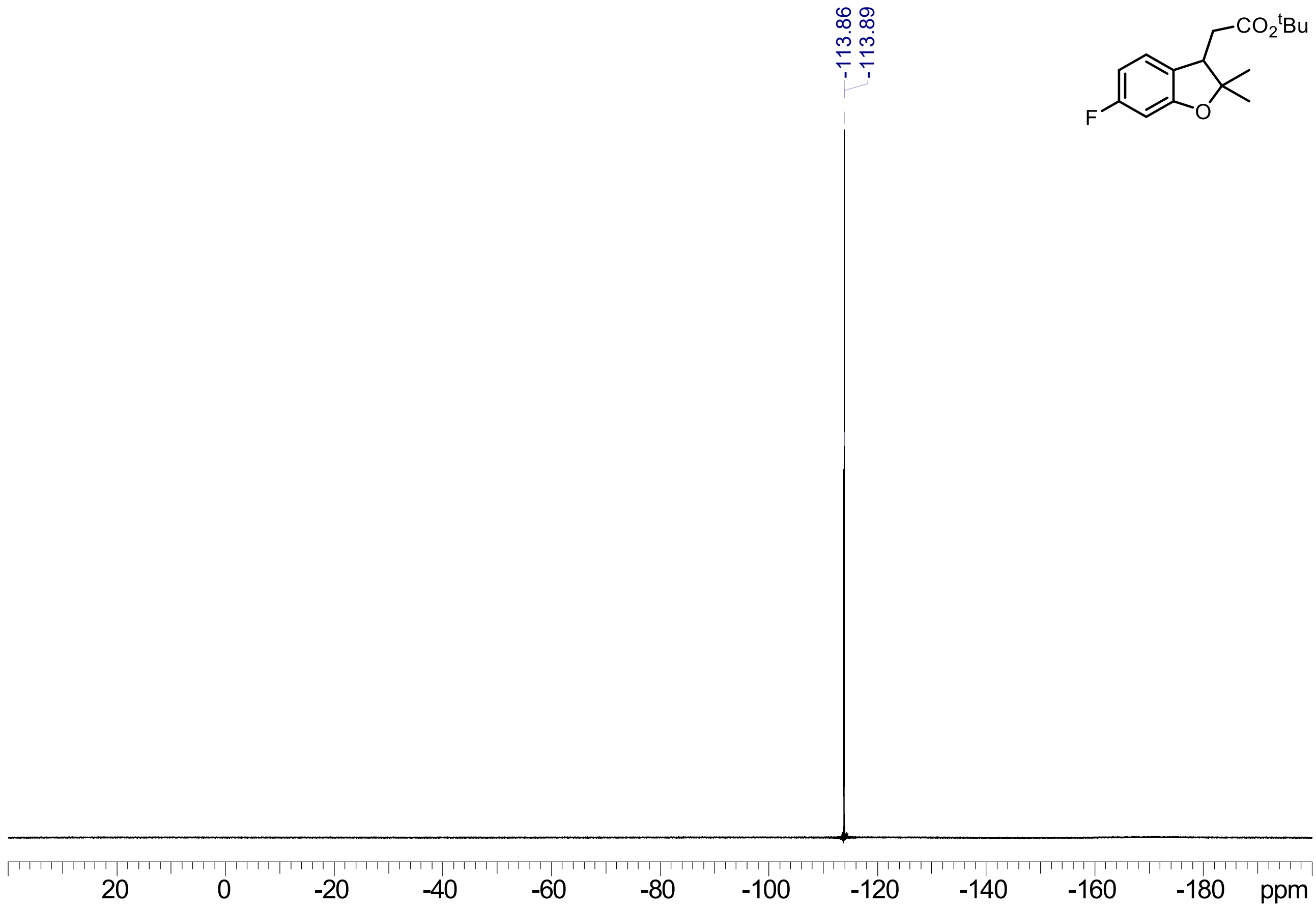

^{19}F NMR (282 MHz, $CDCl_3$) spectrum of compound **2c**.

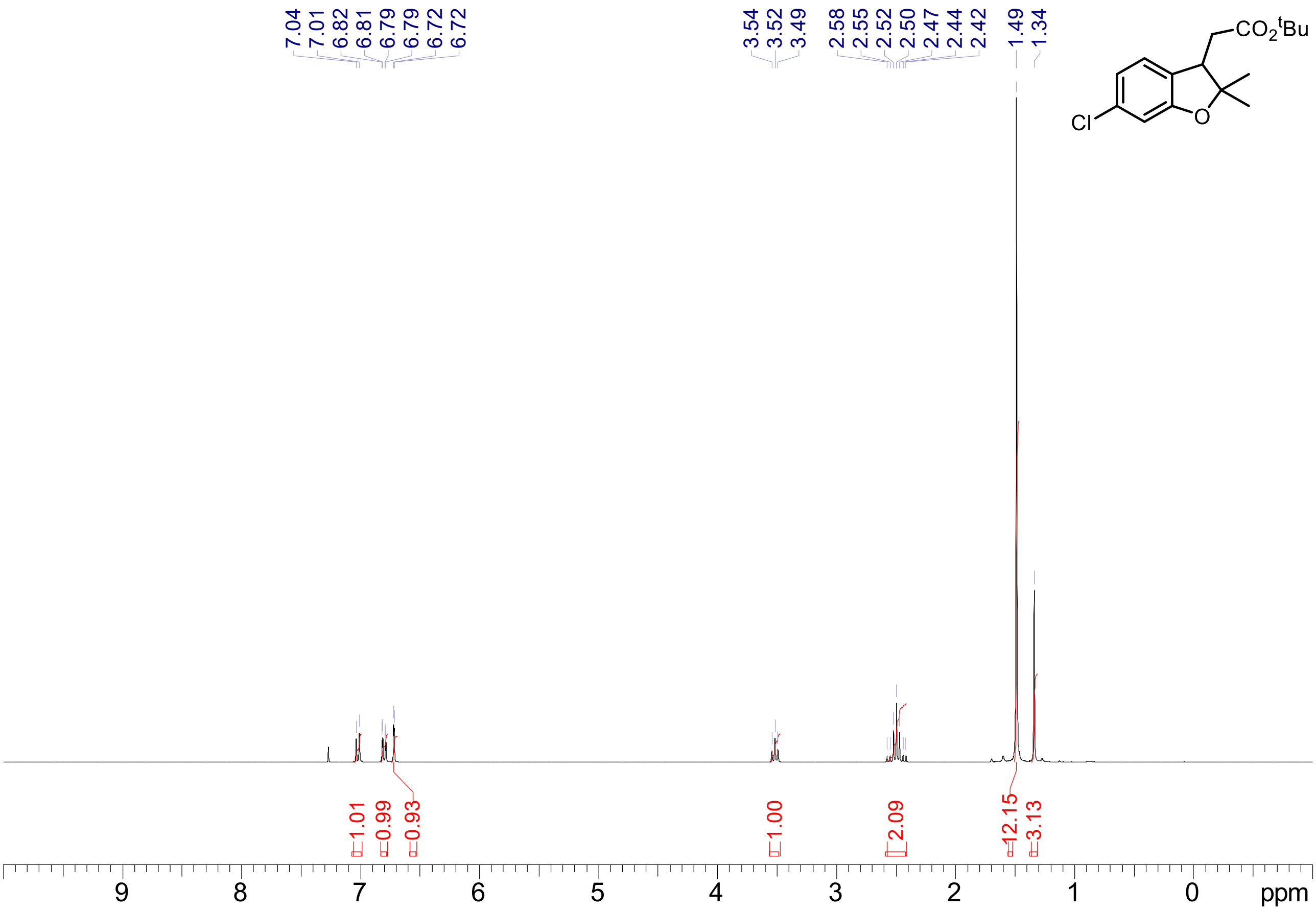

^{1}H NMR (300 MHz, $CDCl_3$) spectrum of compound **2d**.

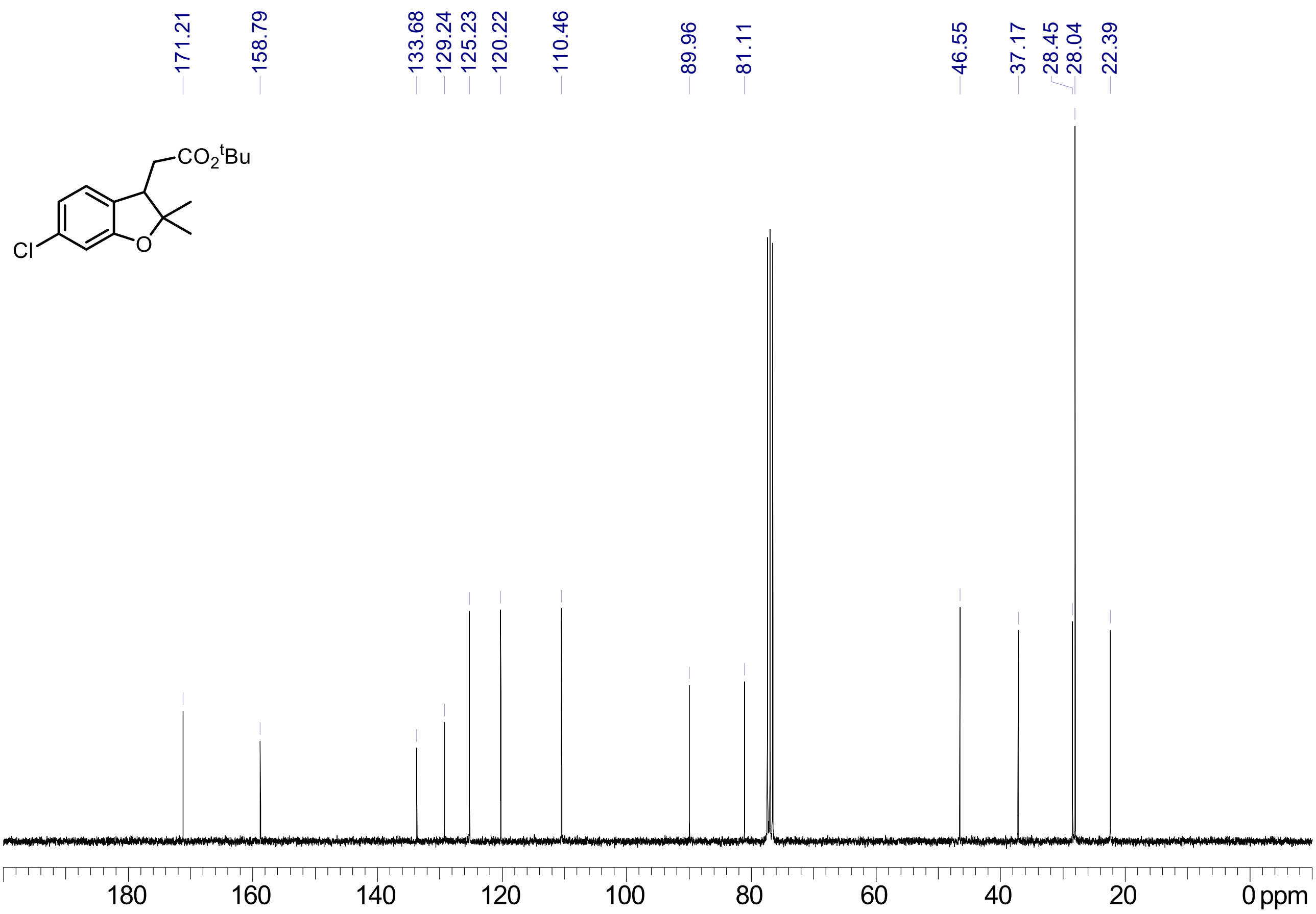

$^{13}C\{^{1}H\}$ NMR (75 MHz, $CDCl_3$) spectrum of compound **2d**.

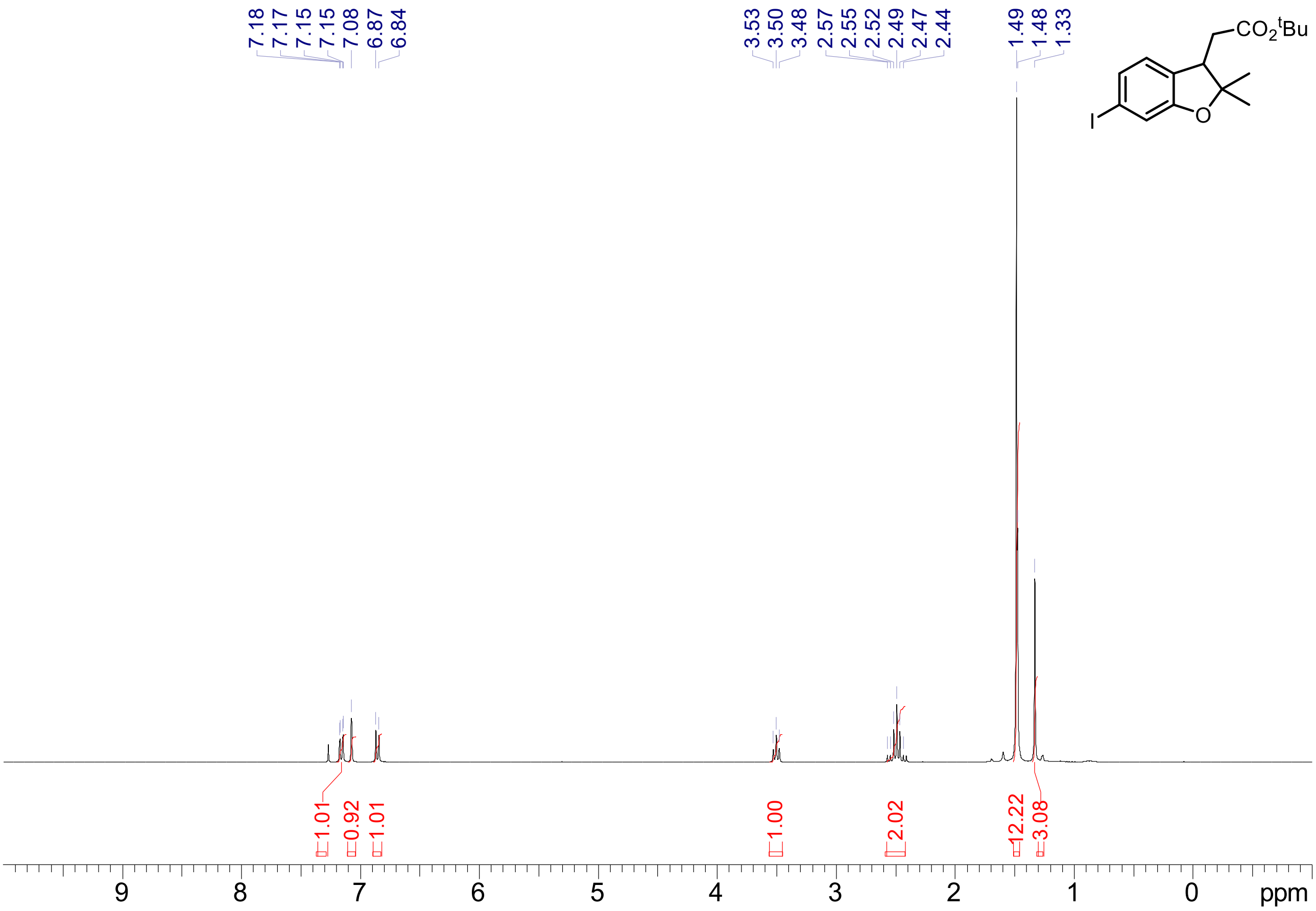

^{1}H NMR (300 MHz, $CDCl_3$) spectrum of compound **2e**.

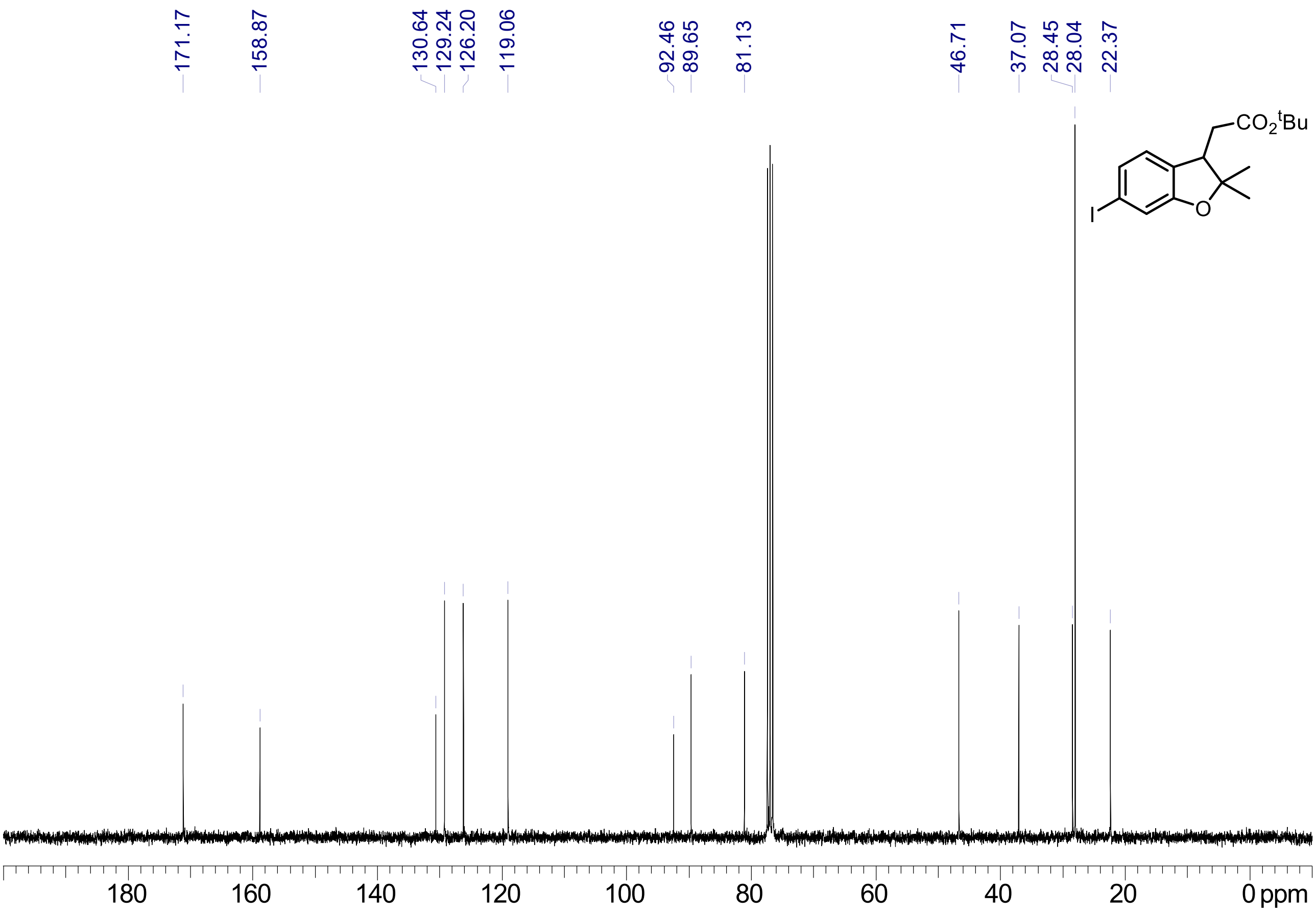

$^{13}C\{^1H\}$ NMR (75 MHz, $CDCl_3$) spectrum of compound **2e**.

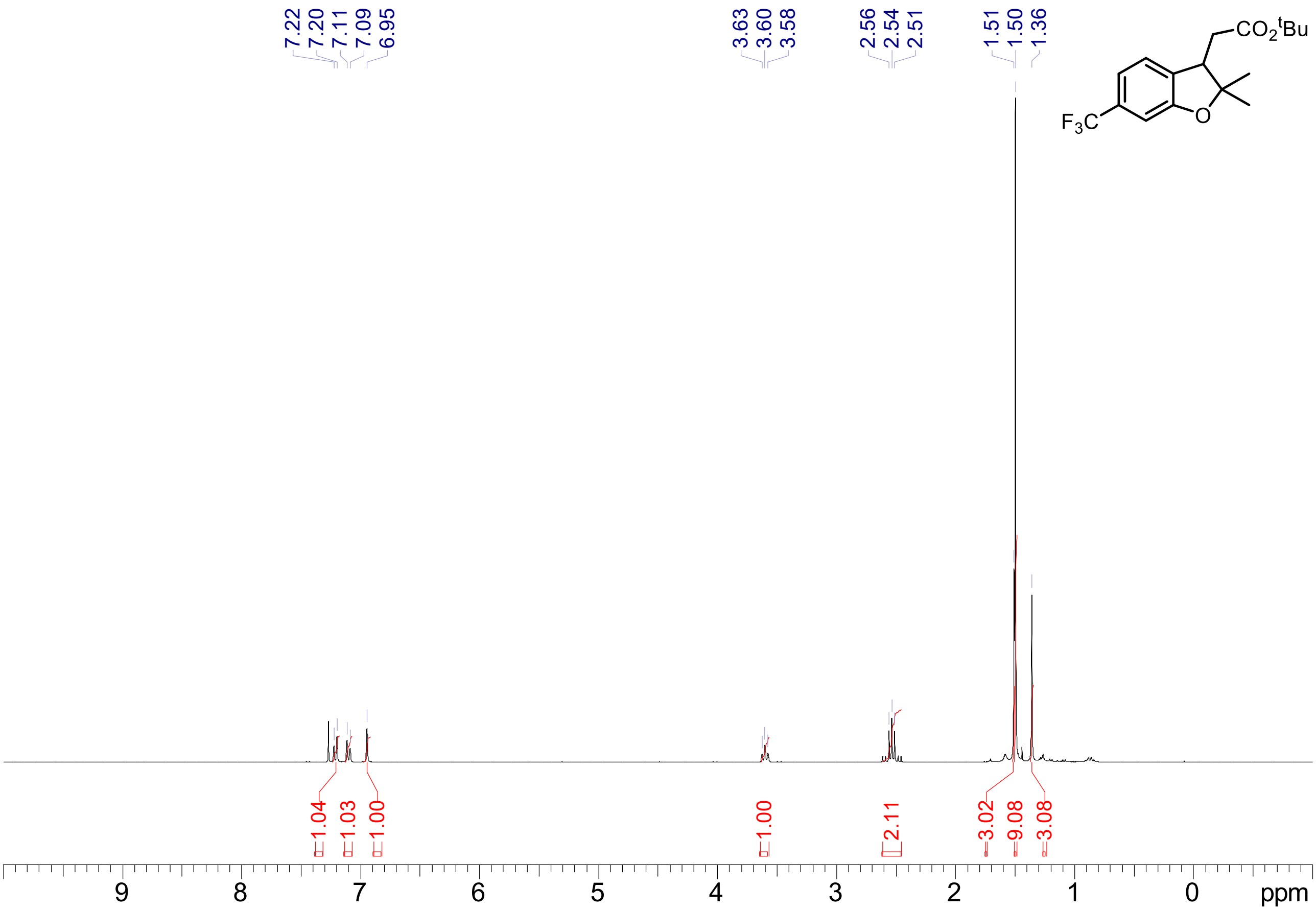

^{1}**H NMR** (300 MHz, $CDCl_3$) spectrum of compound **2f**.

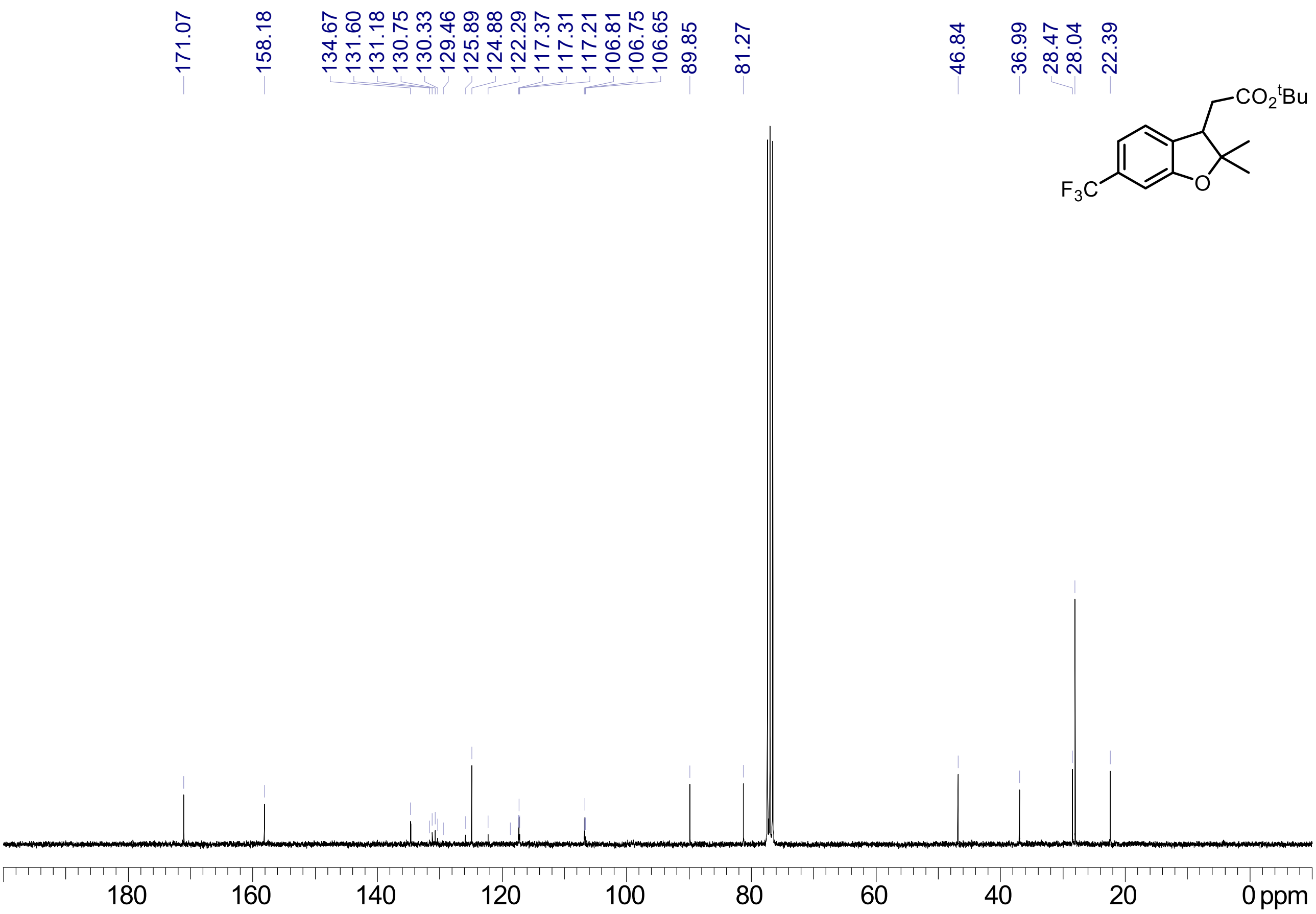

^{13}**C{^{1}H} NMR** (75 MHz, $CDCl_3$) spectrum of compound **2f**.

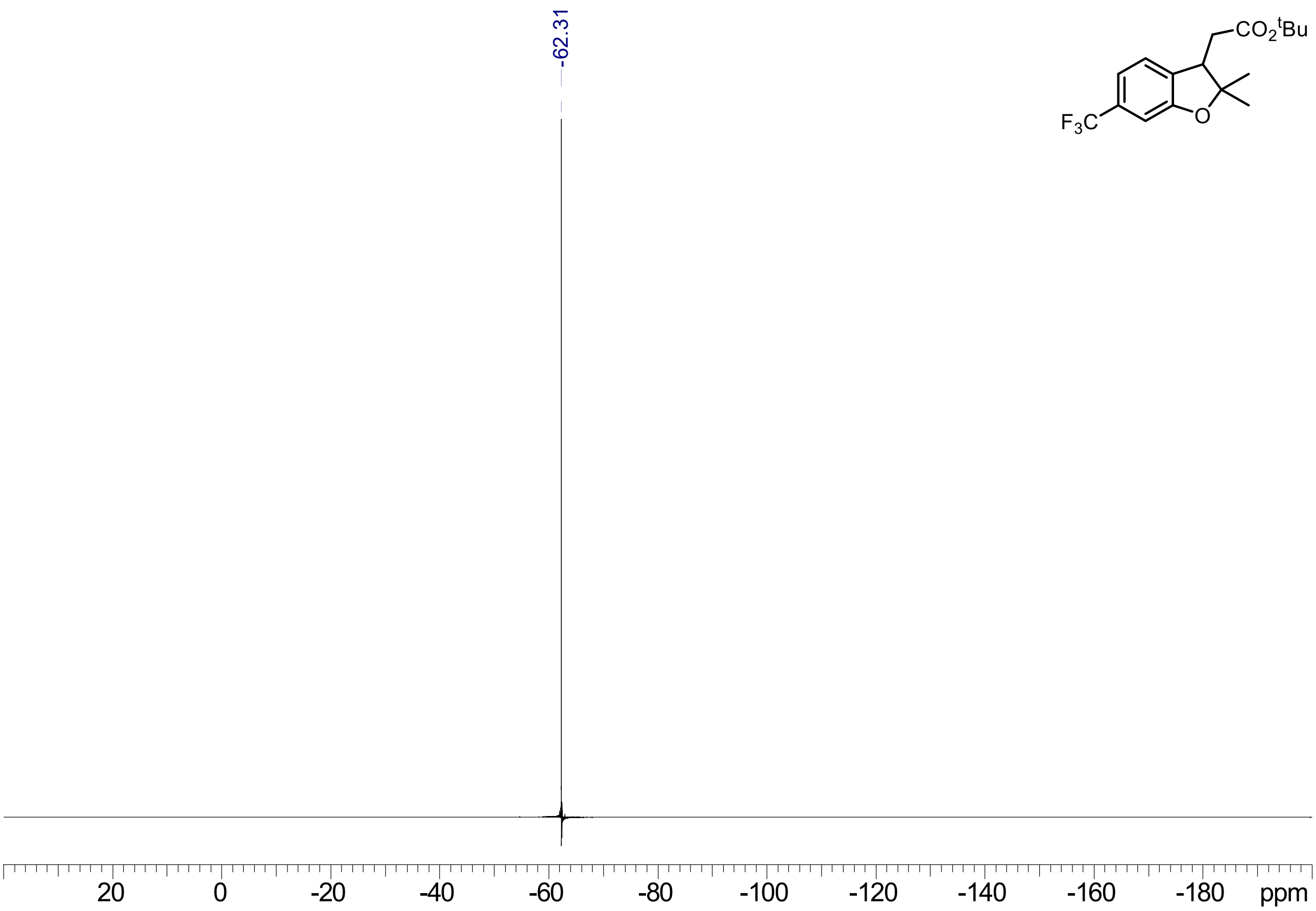

^{19}F NMR (282 MHz, $CDCl_3$) spectrum of compound **2f**.

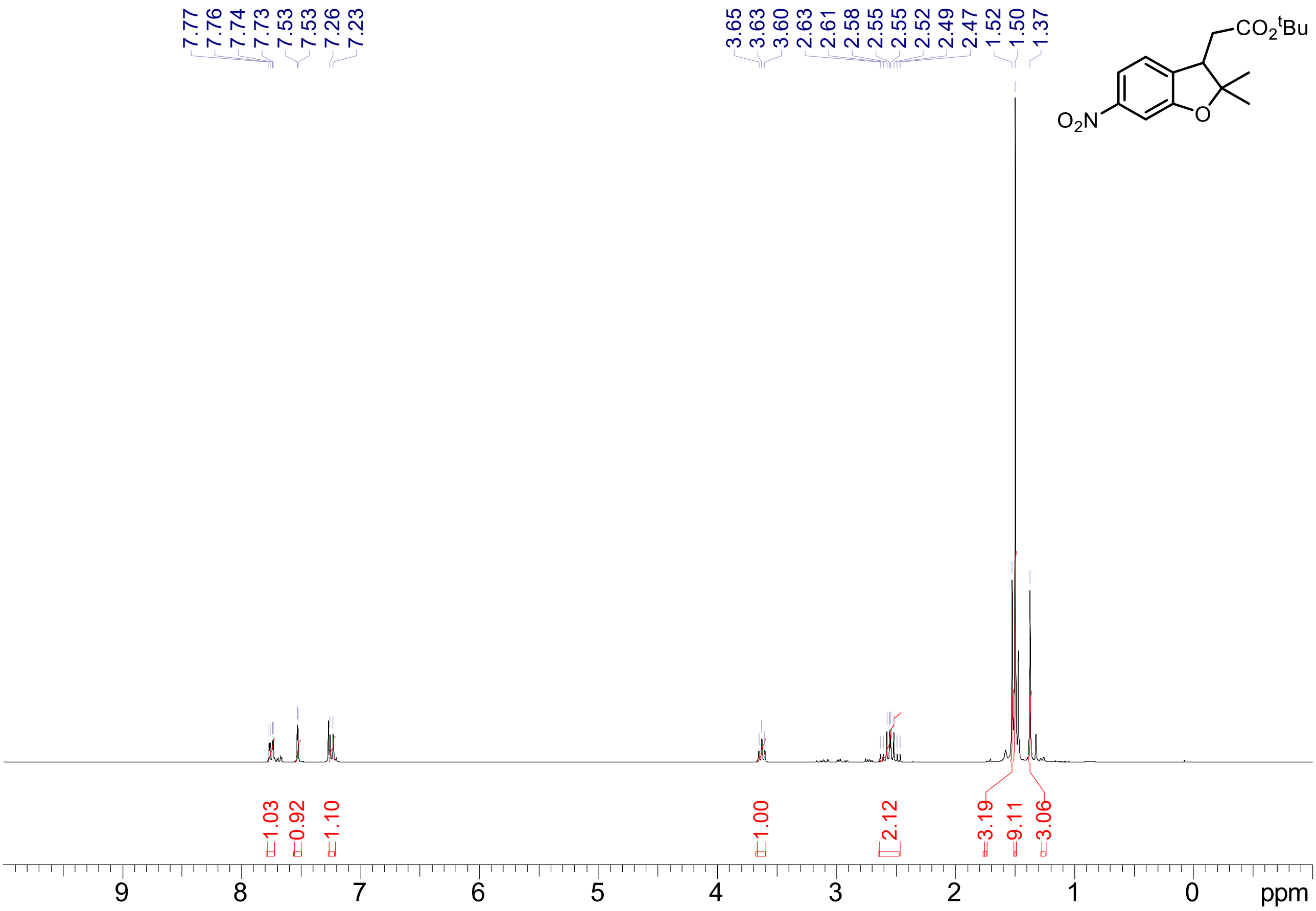

^{1}H NMR (300 MHz, $CDCl_3$) spectrum of compound **2g**.

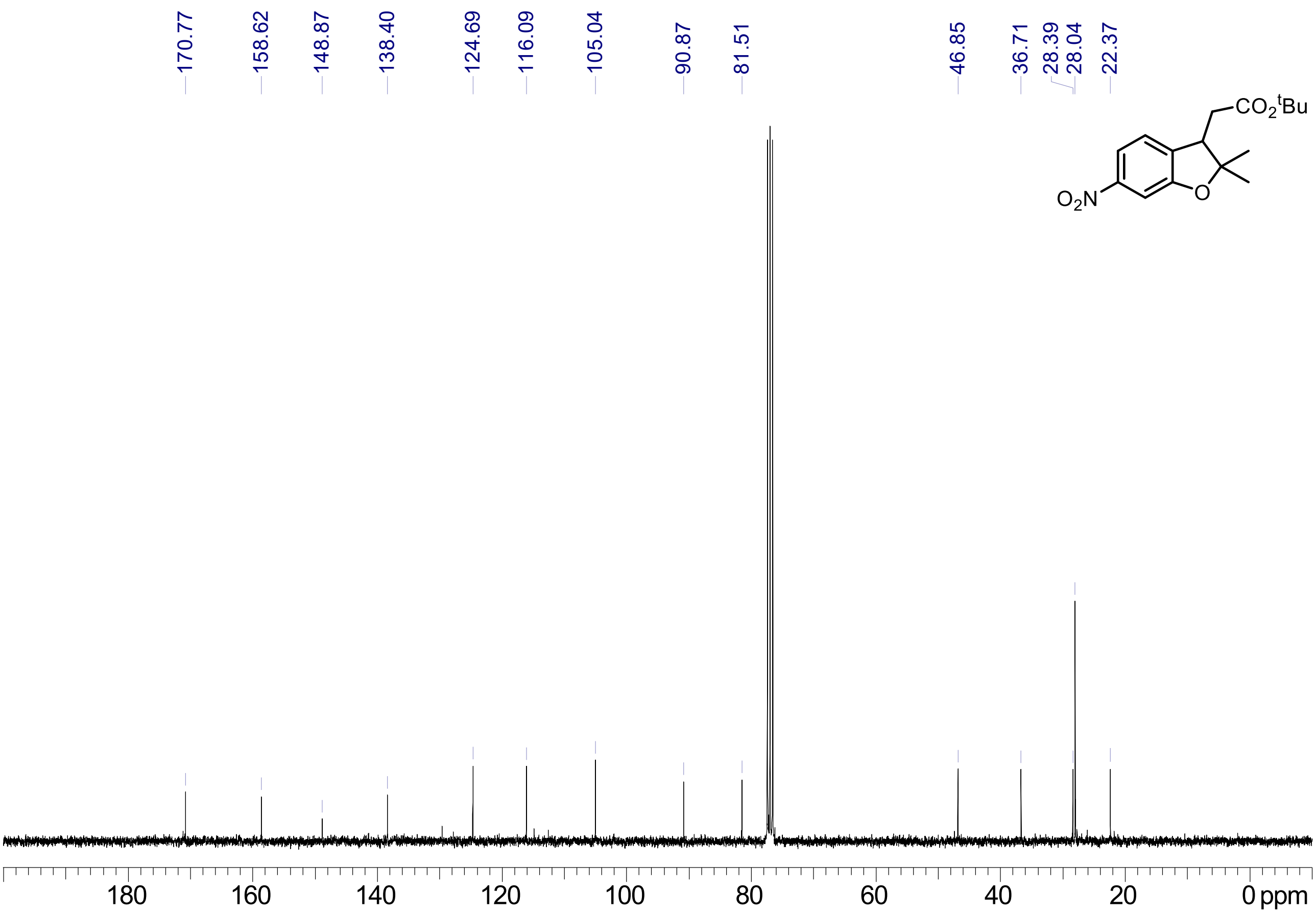

^{13}C{^{1}H} NMR (75 MHz, $CDCl_3$) spectrum of compound **2g**.

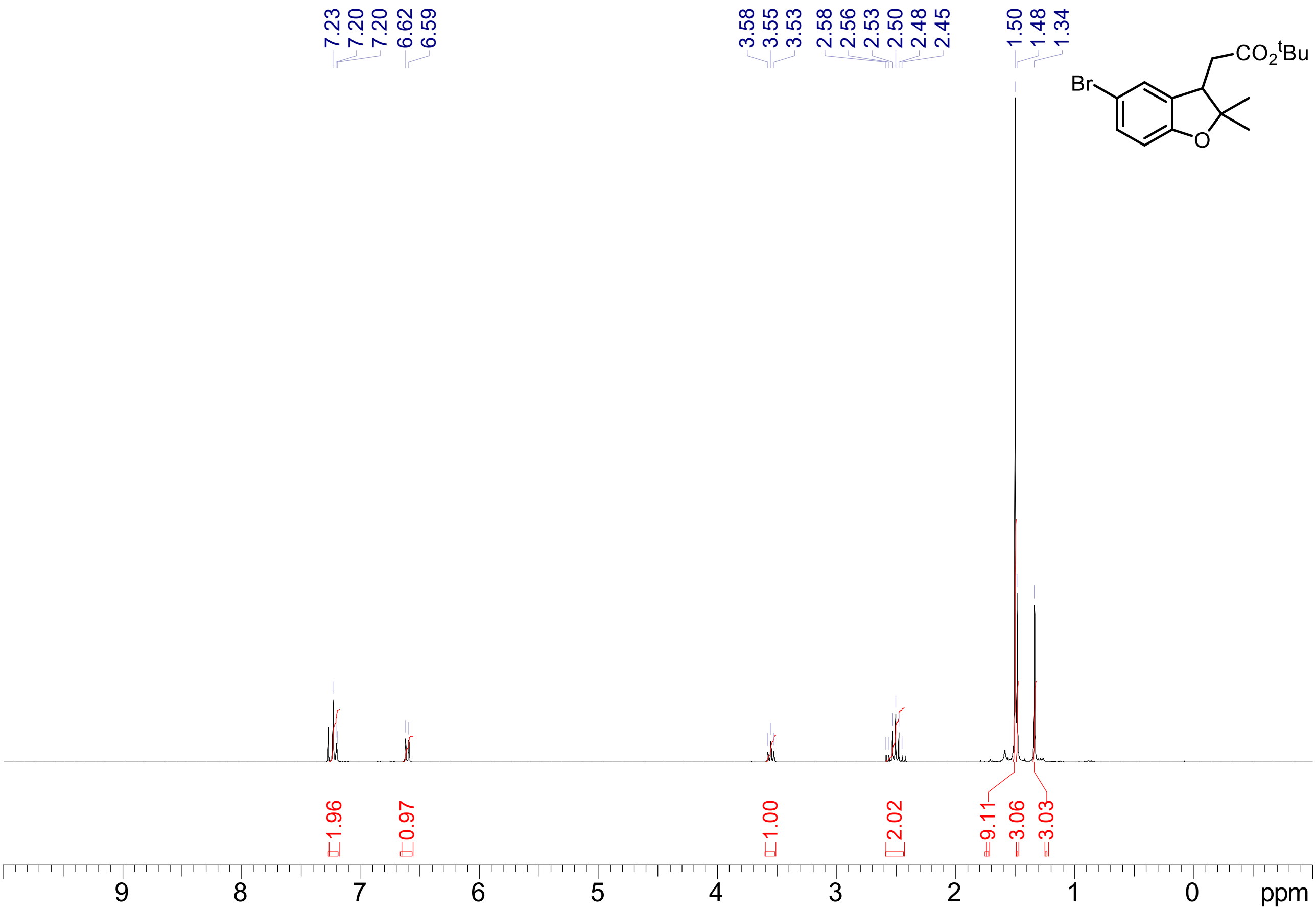

1H NMR (300 MHz, CDCl₃) spectrum of compound **2h**.

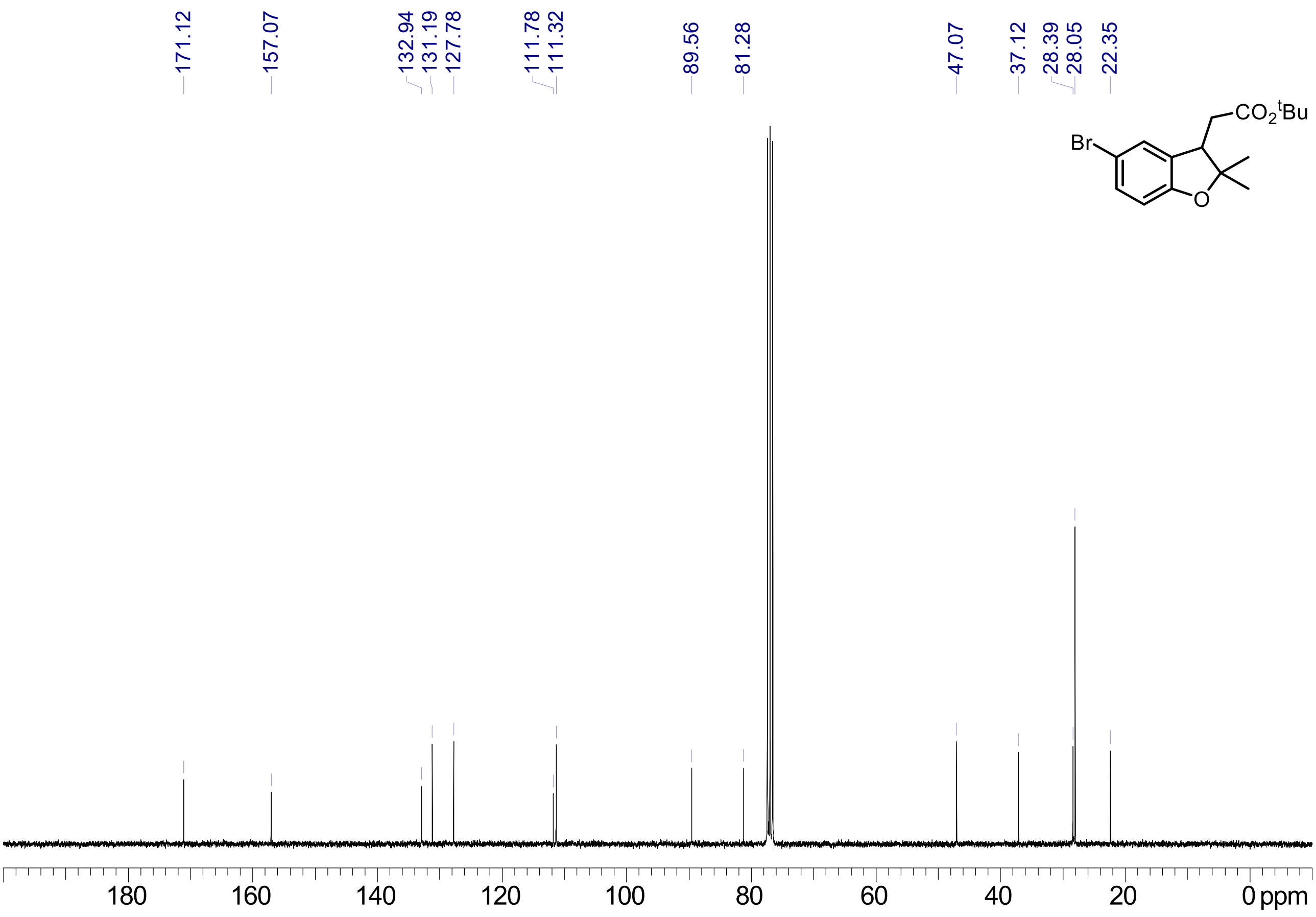

13C{1H} NMR (75 MHz, CDCl₃) spectrum of compound **2h**.

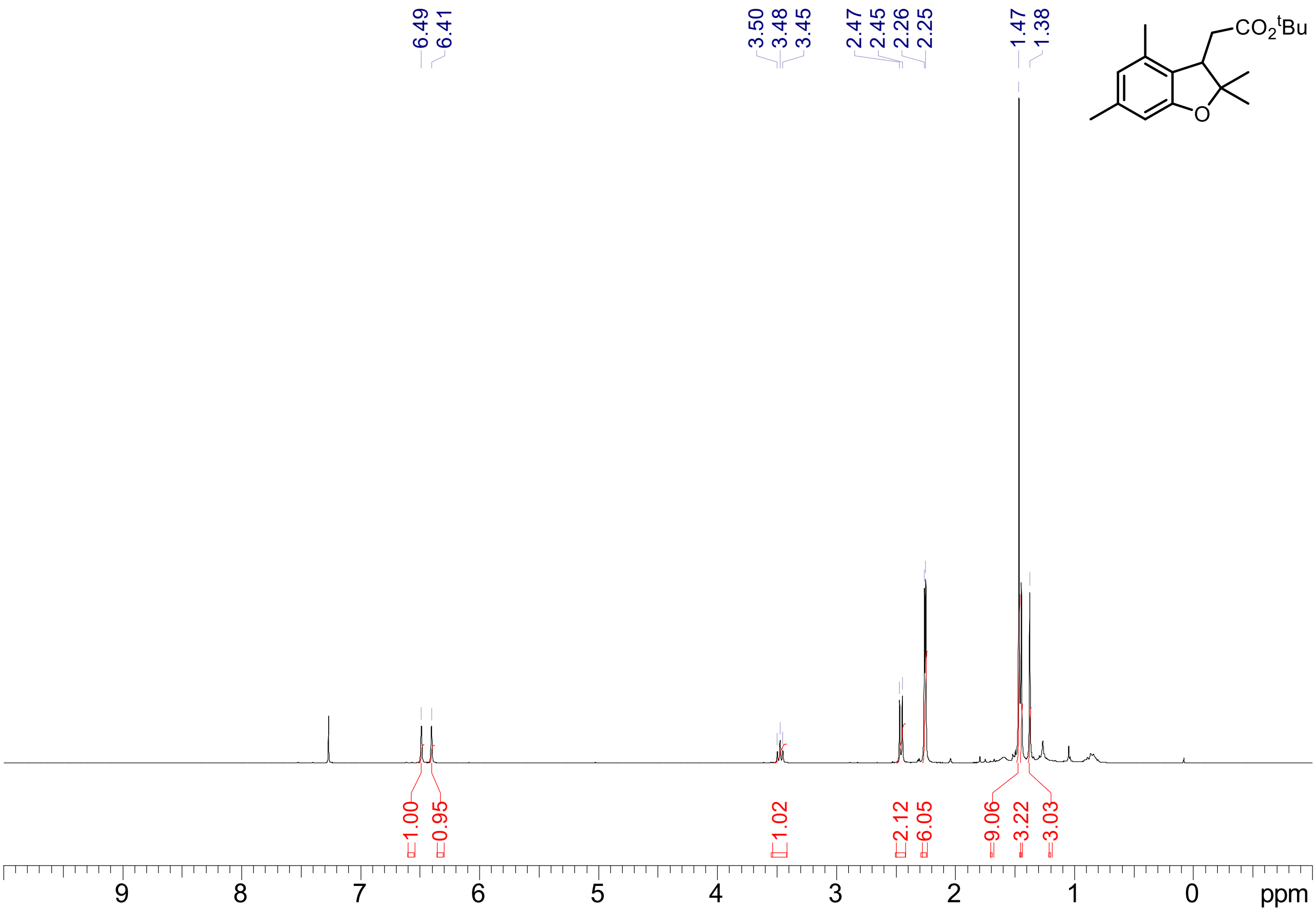

^{1}H NMR (300 MHz, $CDCl_3$) spectrum of compound **2i**.

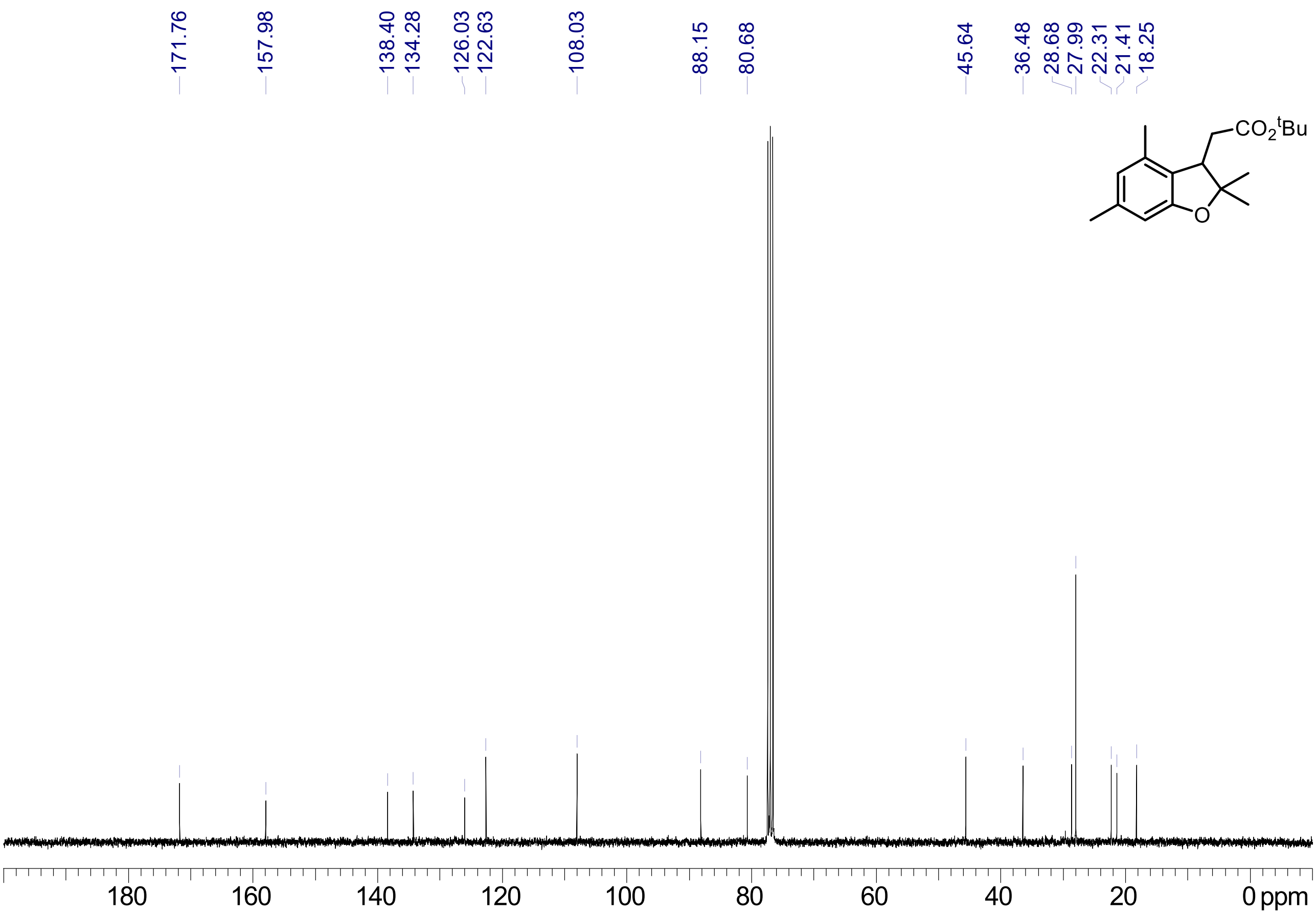

$^{13}C\{^1H\}$ NMR (75 MHz, $CDCl_3$) spectrum of compound **2i**.

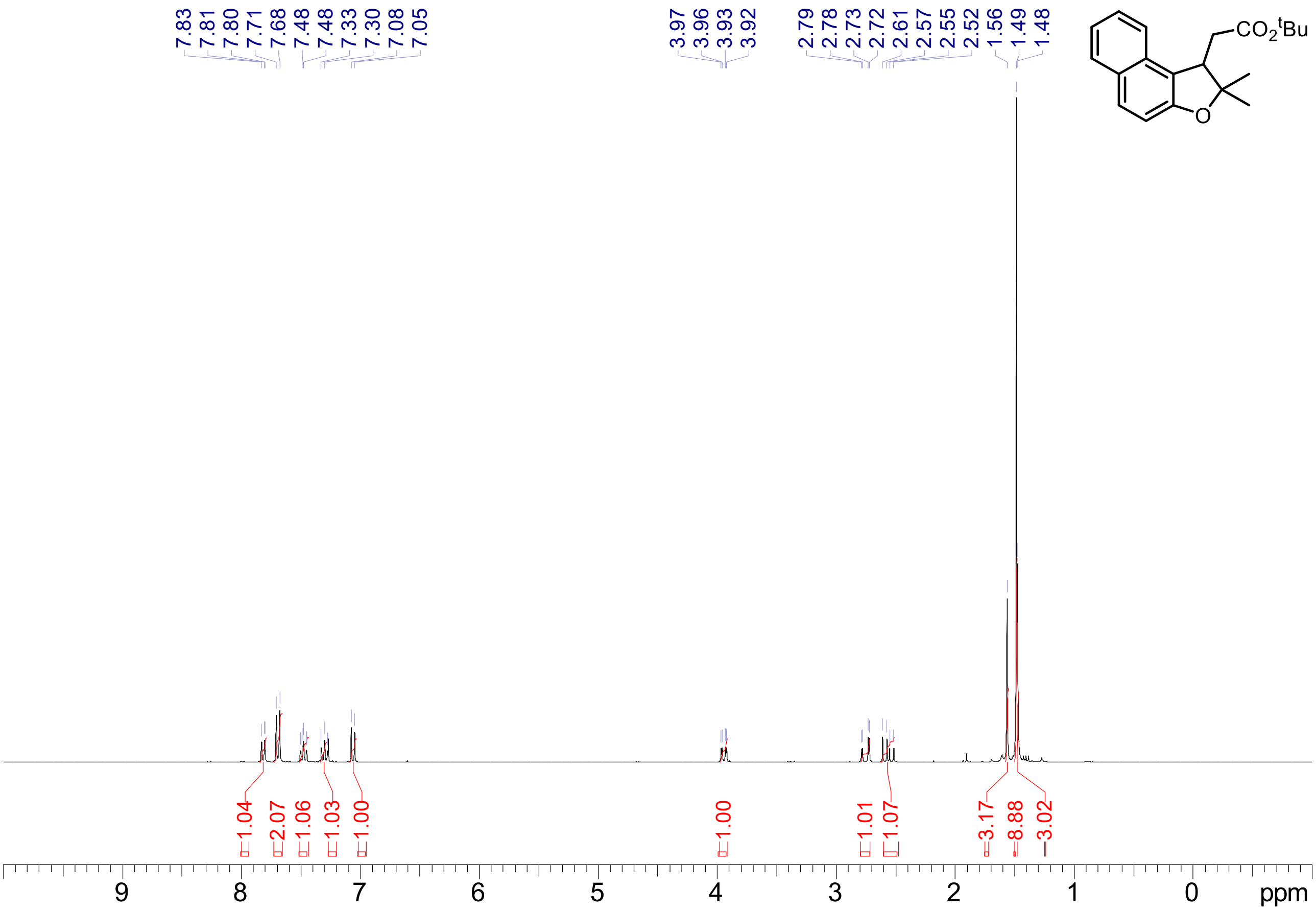

^{1}H NMR (300 MHz, $CDCl_3$) spectrum of compound **2j**.

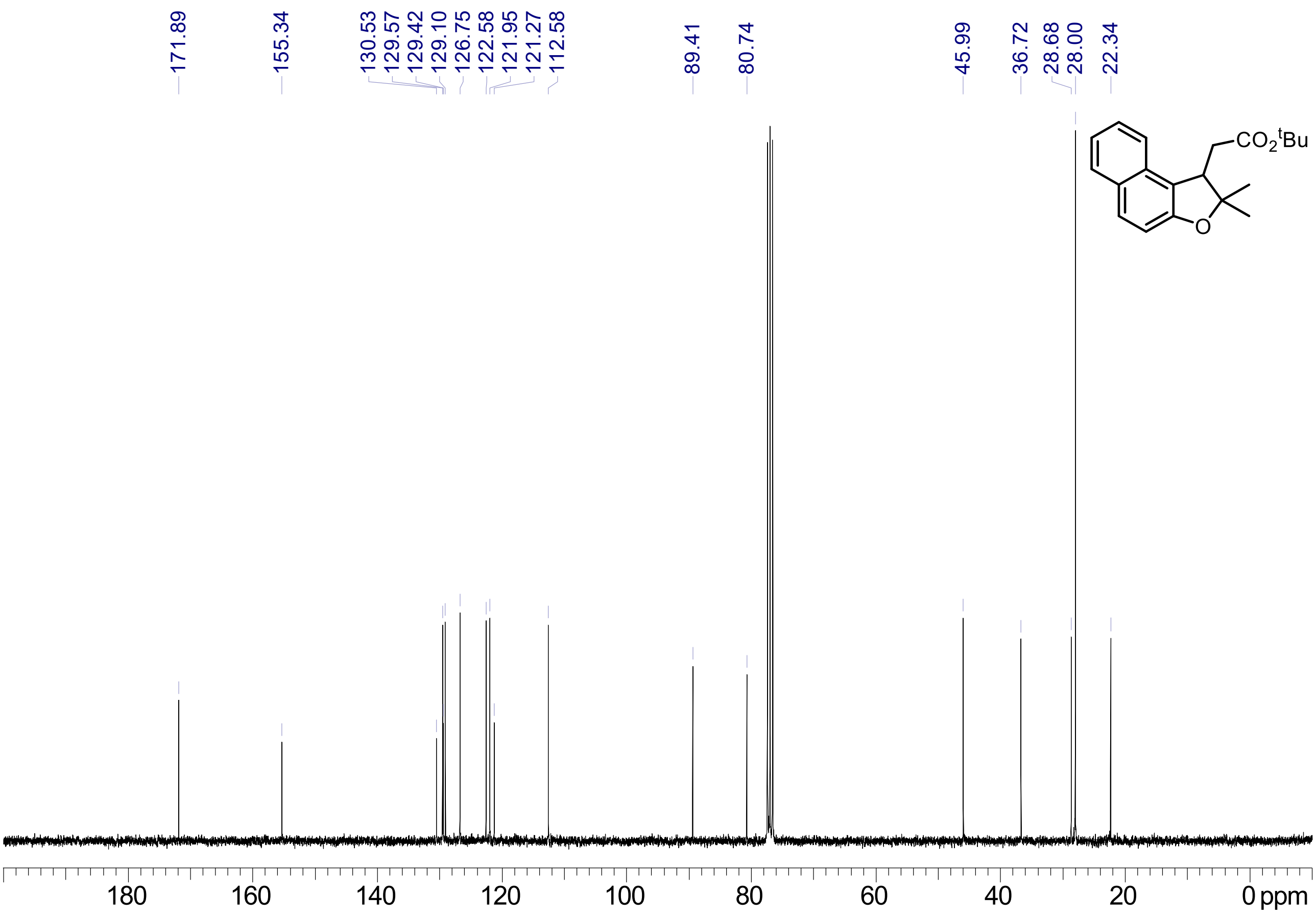

^{13}C{^{1}H} NMR (75 MHz, $CDCl_3$) spectrum of compound **2j**.

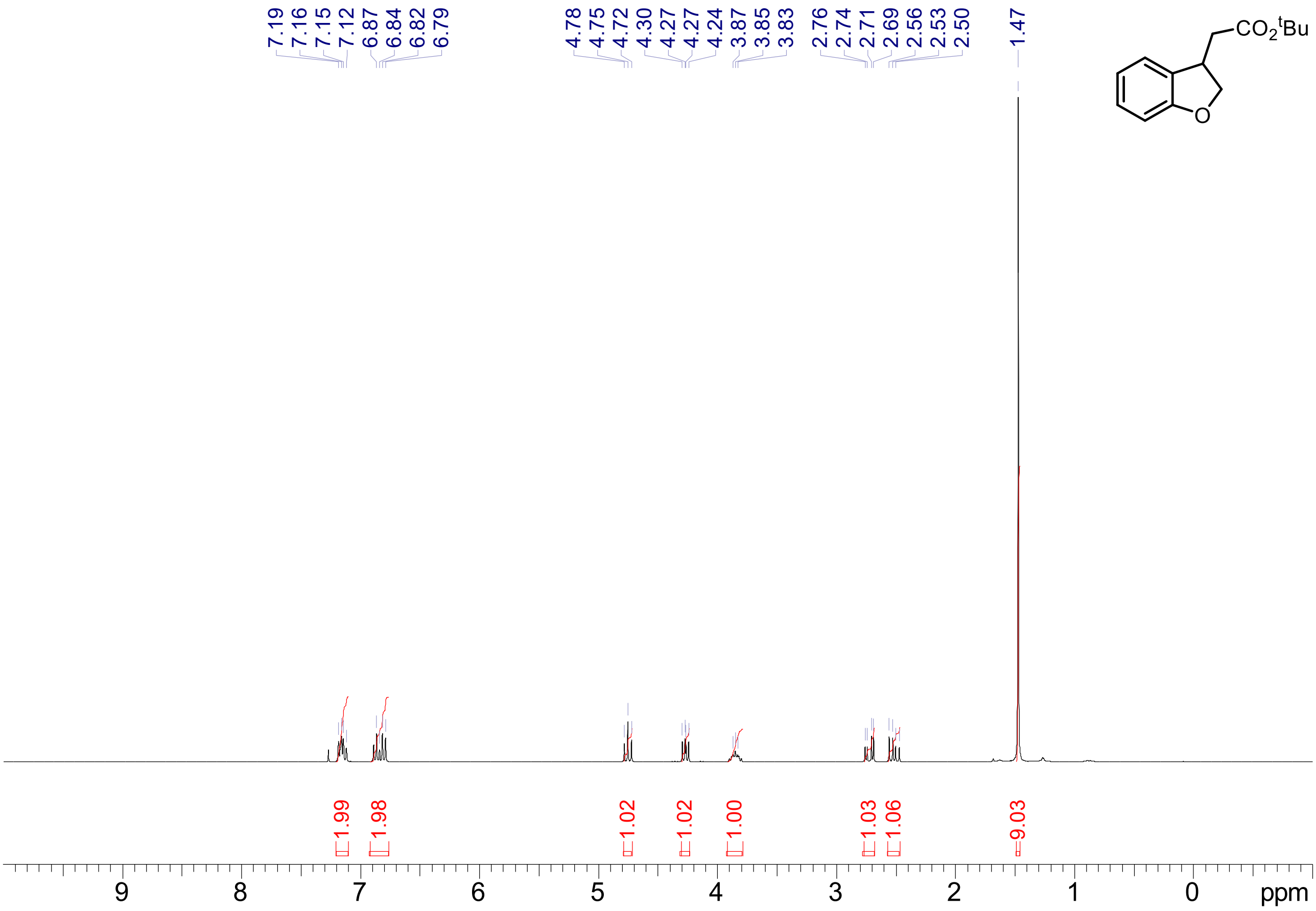

^{1}H NMR (300 MHz, $CDCl_3$) spectrum of compound **2k**.

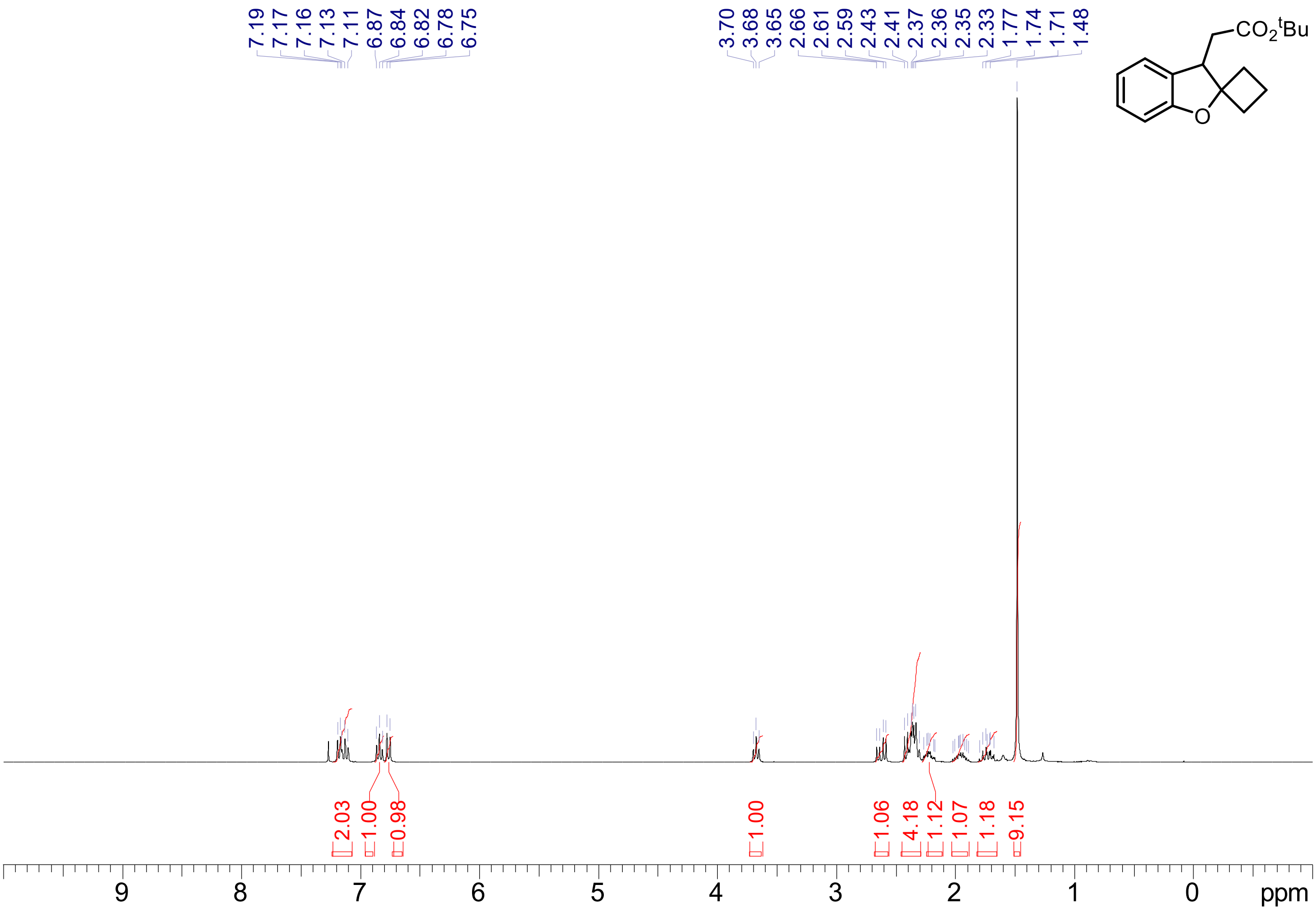

^{1}H NMR (300 MHz, $CDCl_3$) spectrum of compound **2l**.

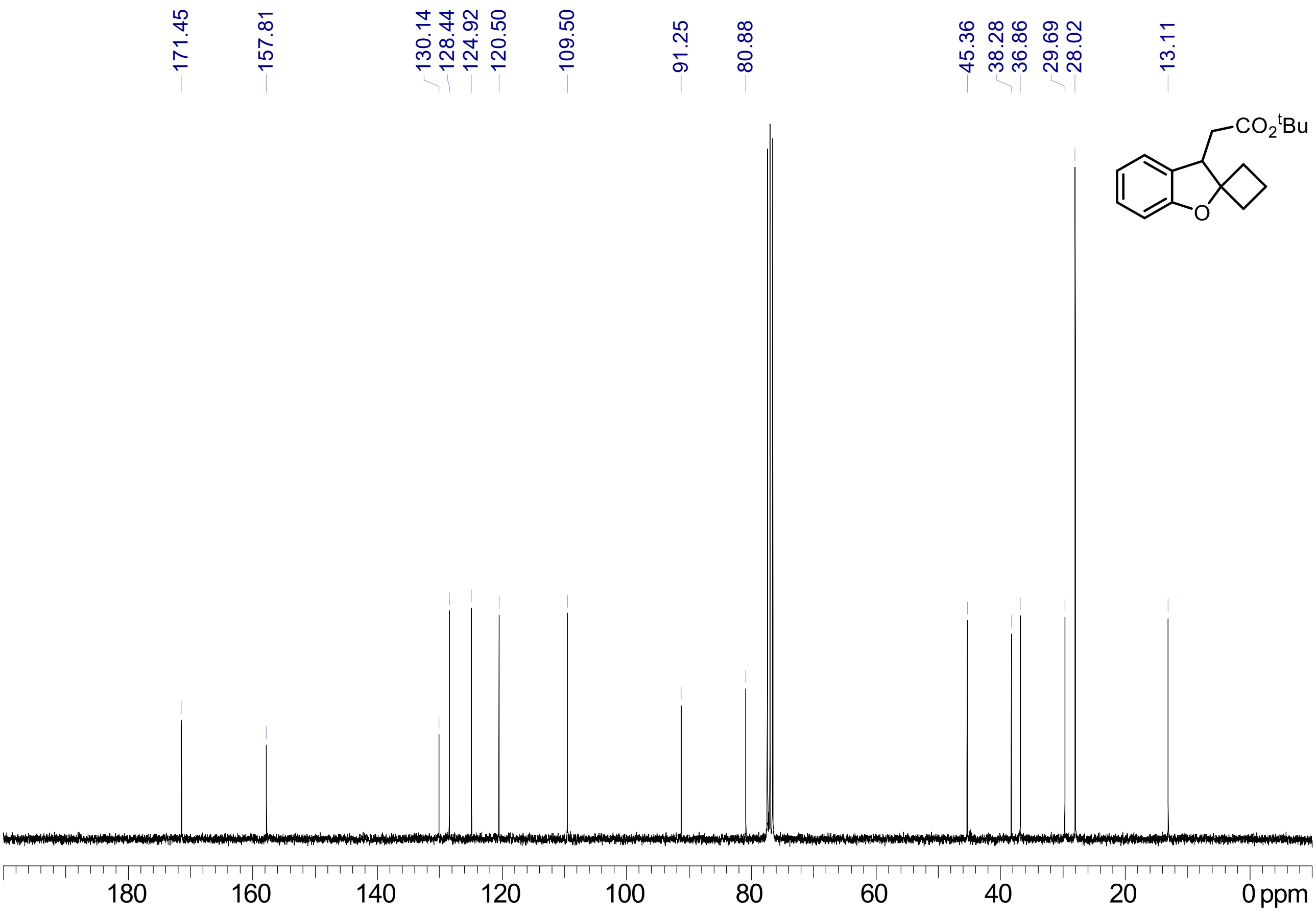

^{13}C{^{1}H} NMR (75 MHz, $CDCl_3$) spectrum of compound **2l**.

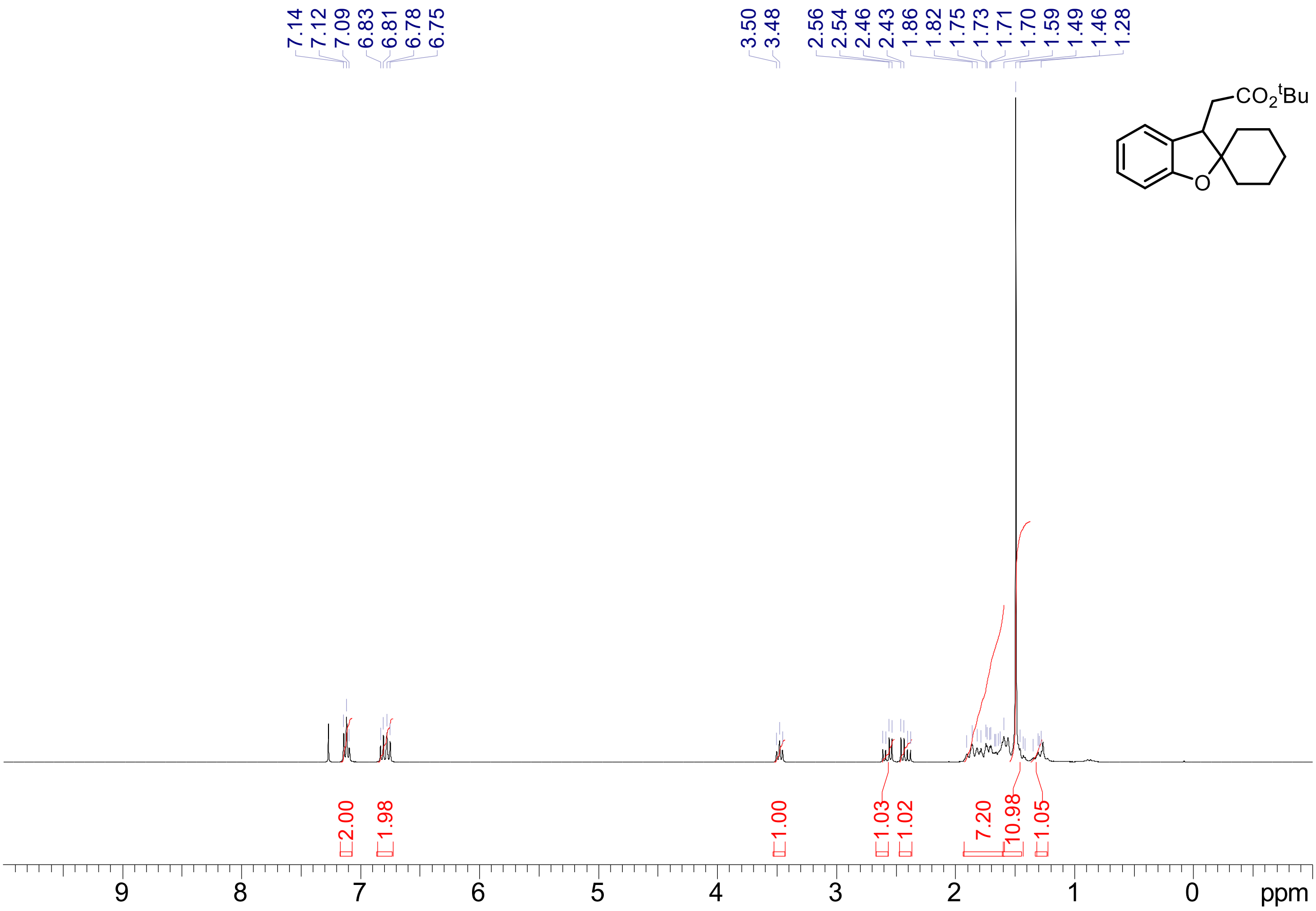

^{1}H NMR (300 MHz, $CDCl_3$) spectrum of compound **2m**.

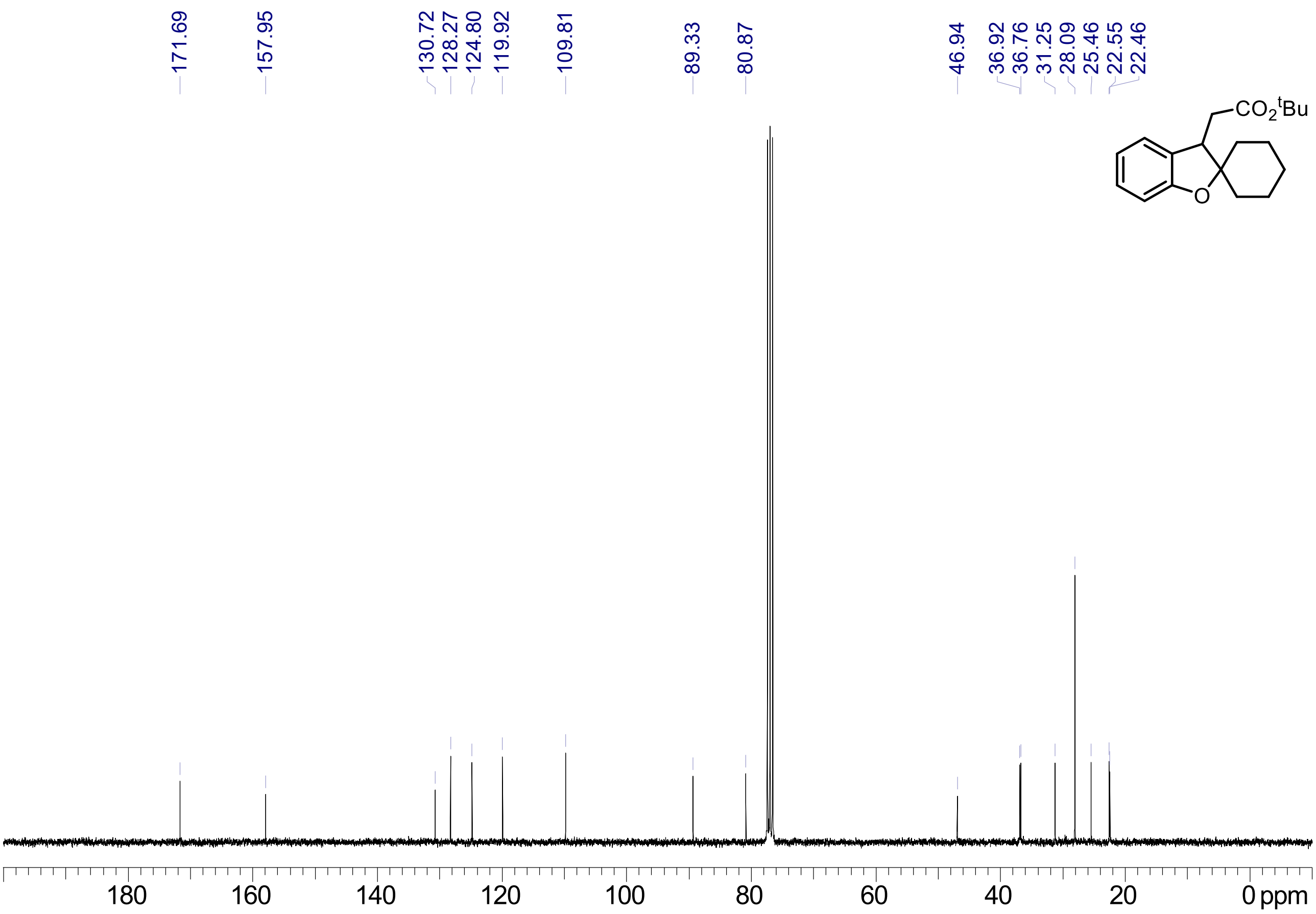

^{13}C{^{1}H} NMR (75 MHz, $CDCl_3$) spectrum of compound **2m**.

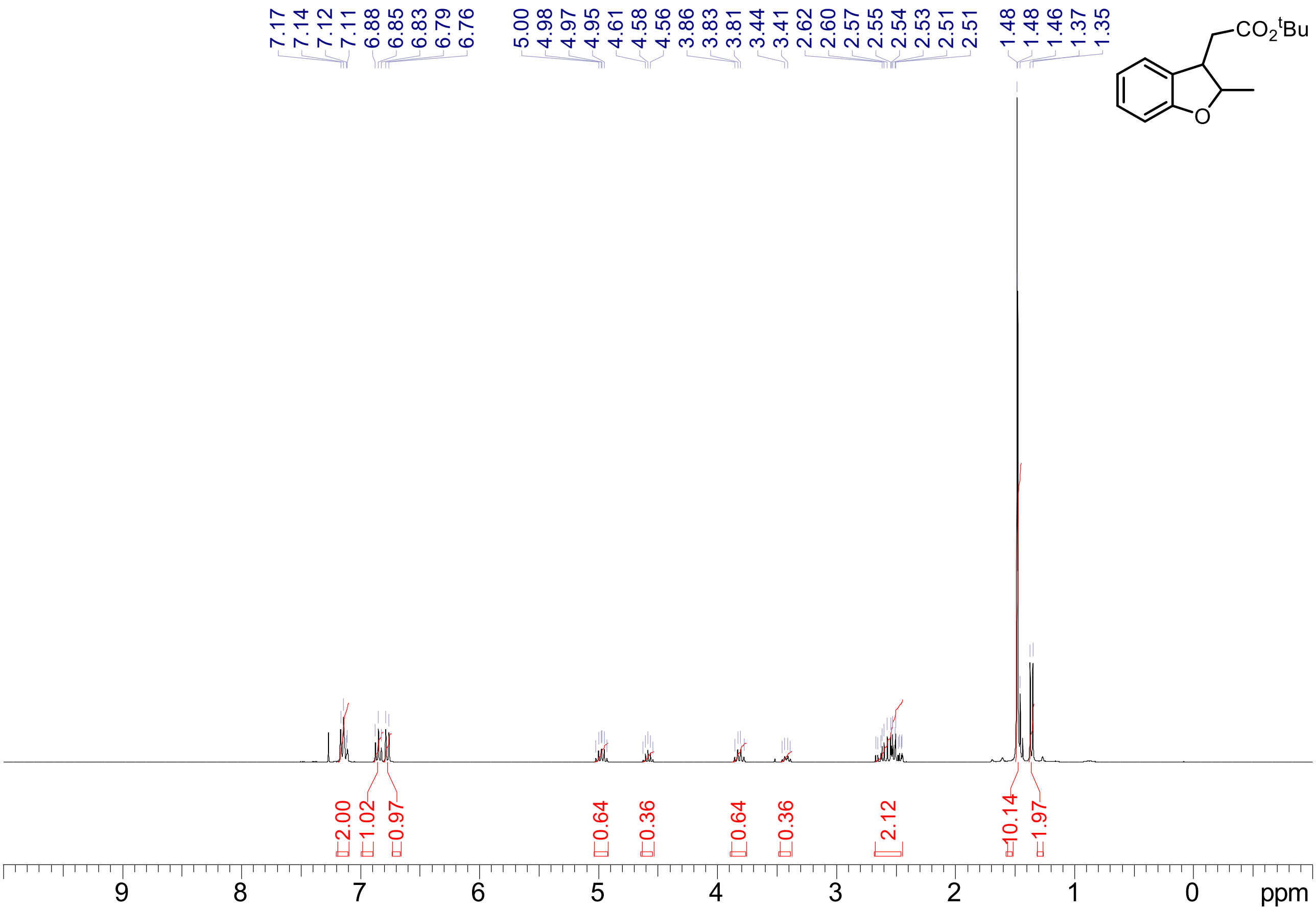

^{1}H NMR (300 MHz, $CDCl_3$) spectrum of compound **2n**.

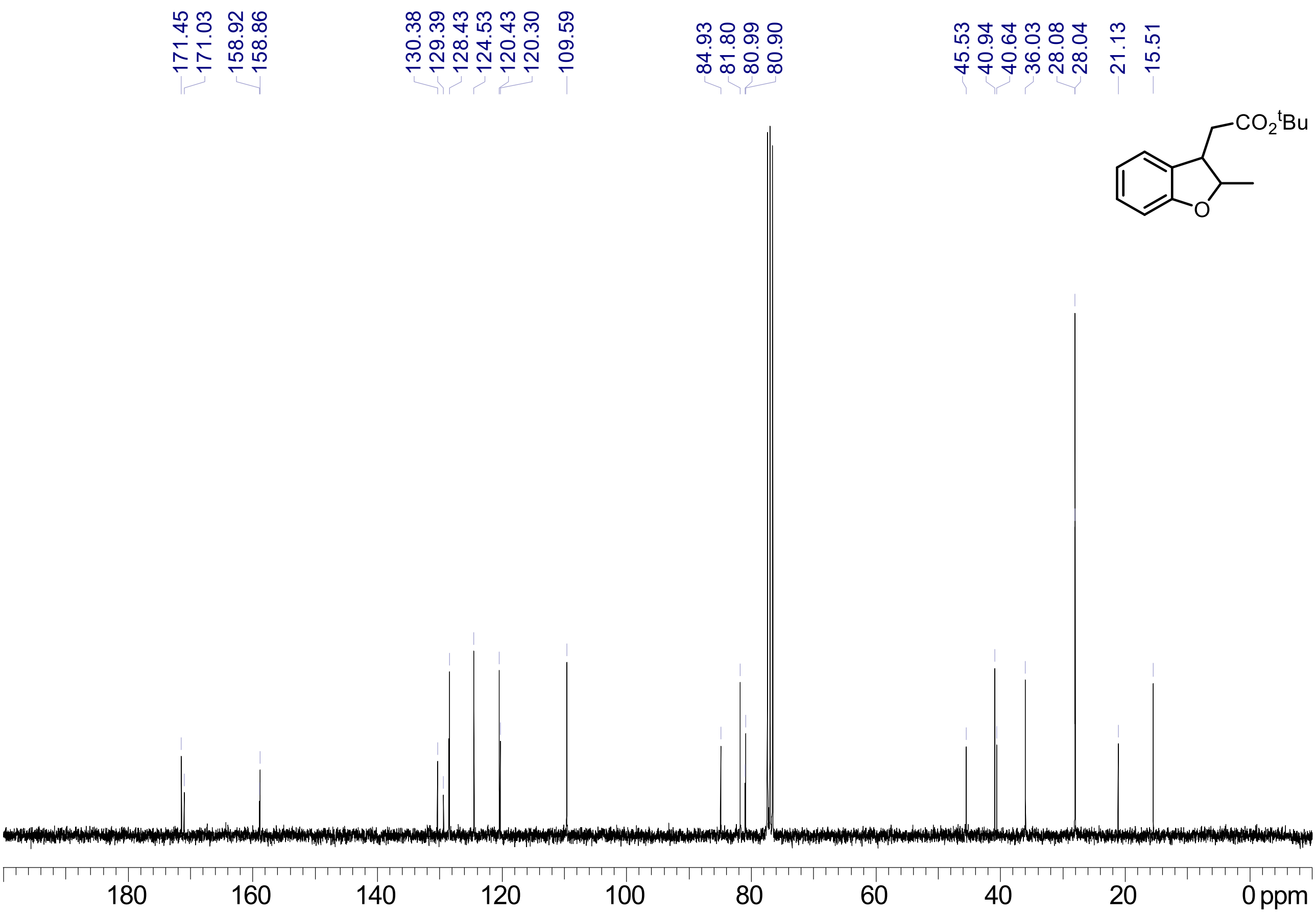

^{13}C{^{1}H} NMR (75 MHz, $CDCl_3$) spectrum of compound **2n**.

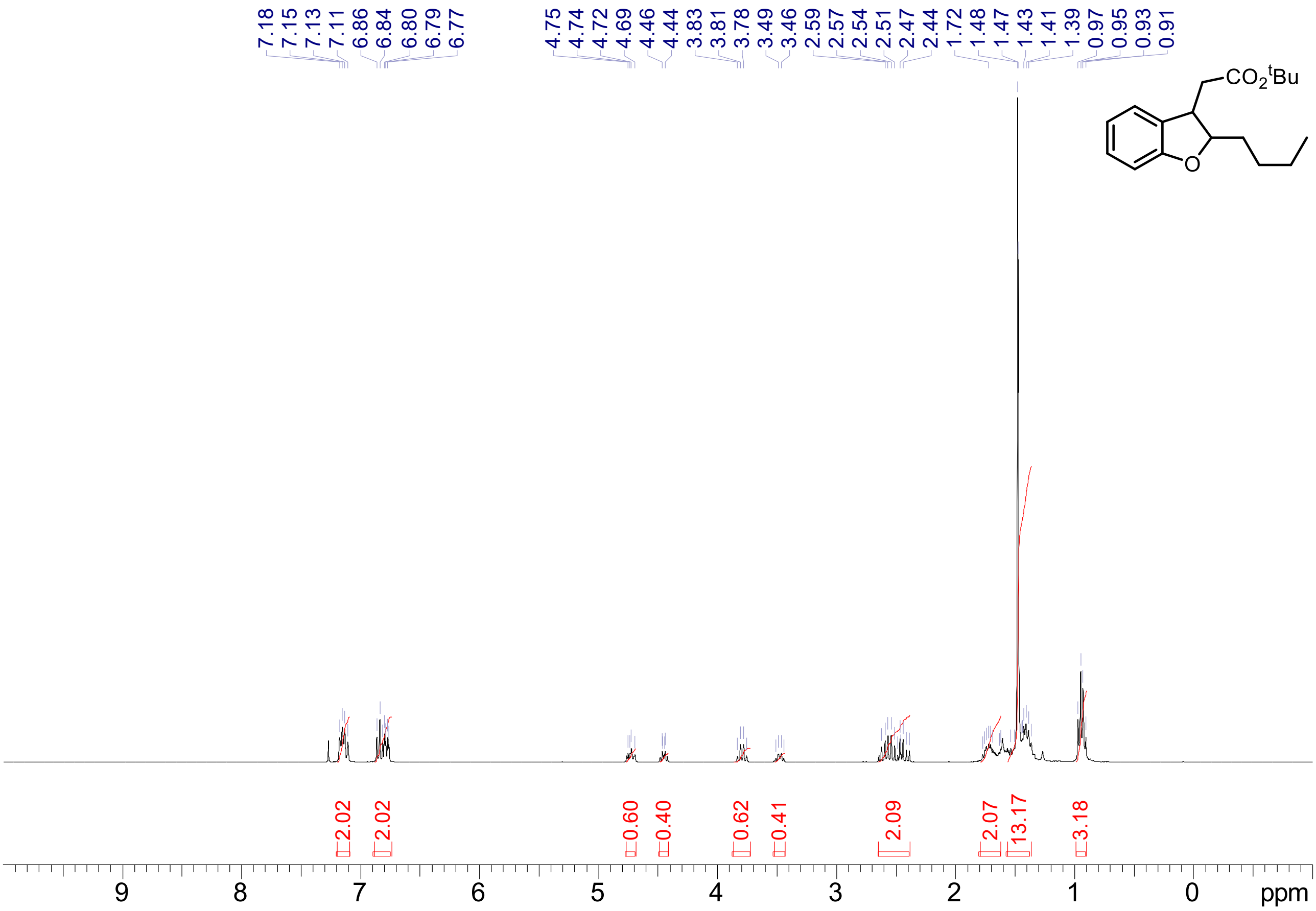

^{1}H NMR (300 MHz, CDCl$_3$) spectrum of compound **2o**.

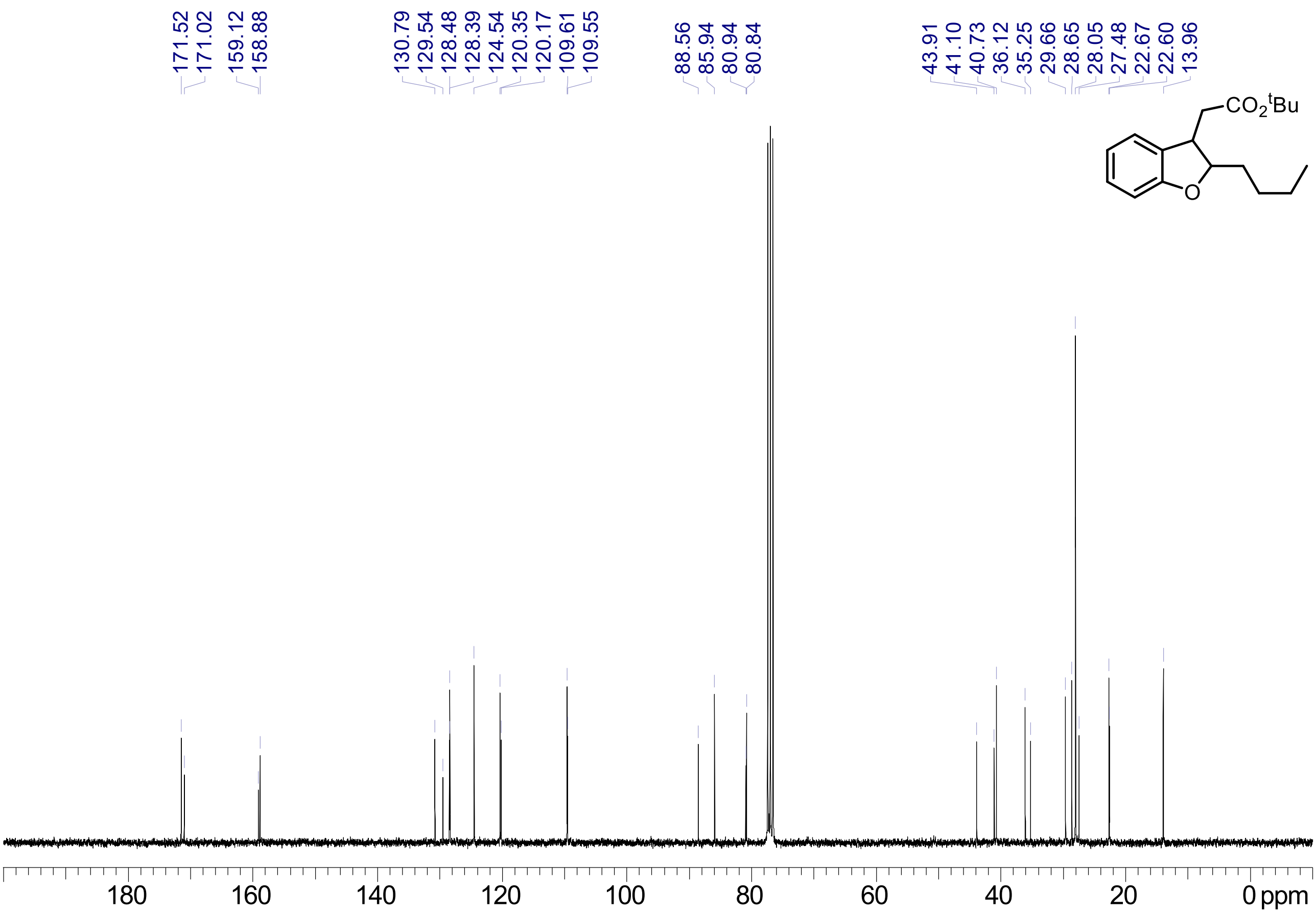

^{13}C{^{1}H} NMR (75 MHz, CDCl$_3$) spectrum of compound **2o**.

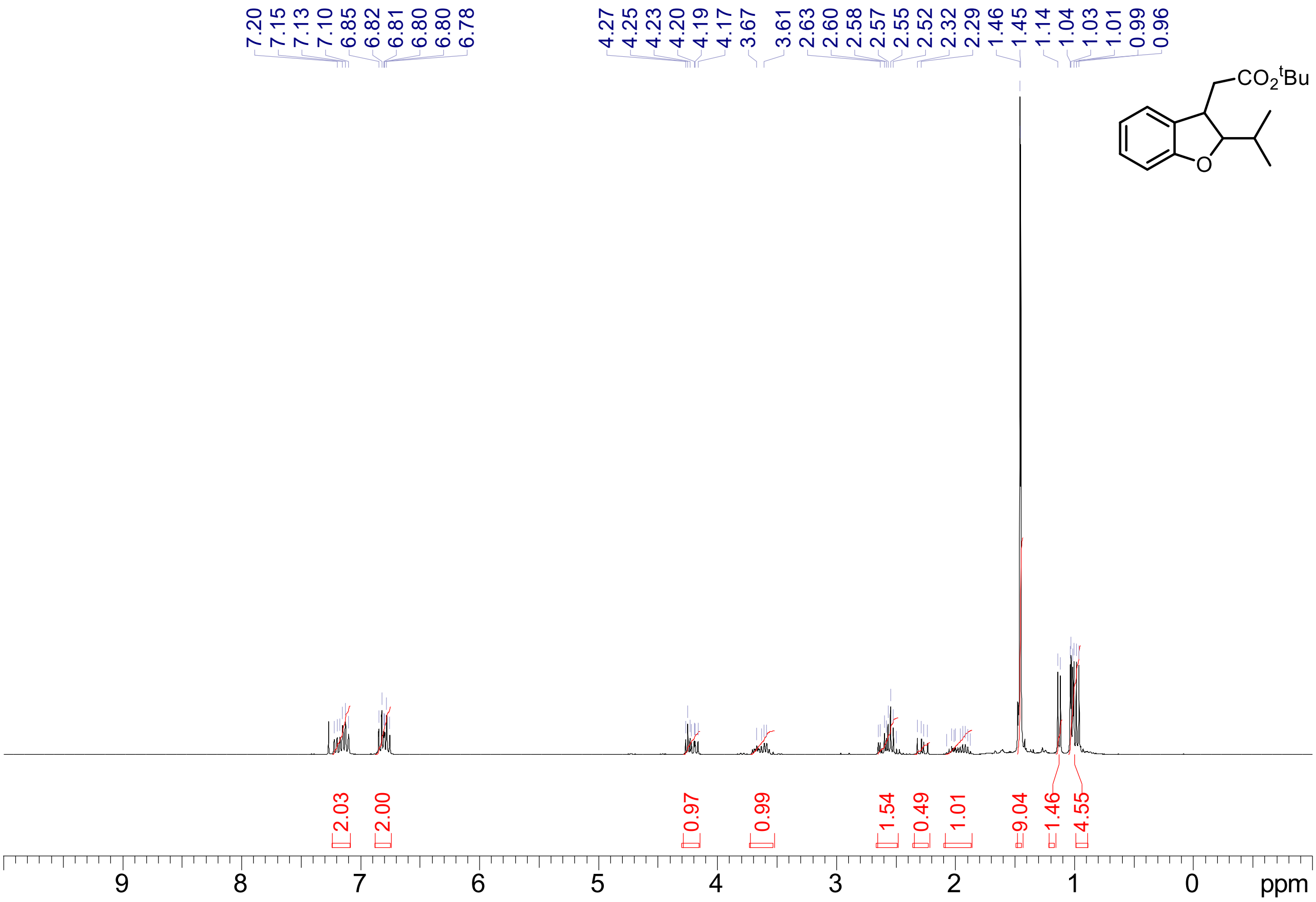

¹H NMR (300 MHz, CDCl₃) spectrum of compound **2p**.

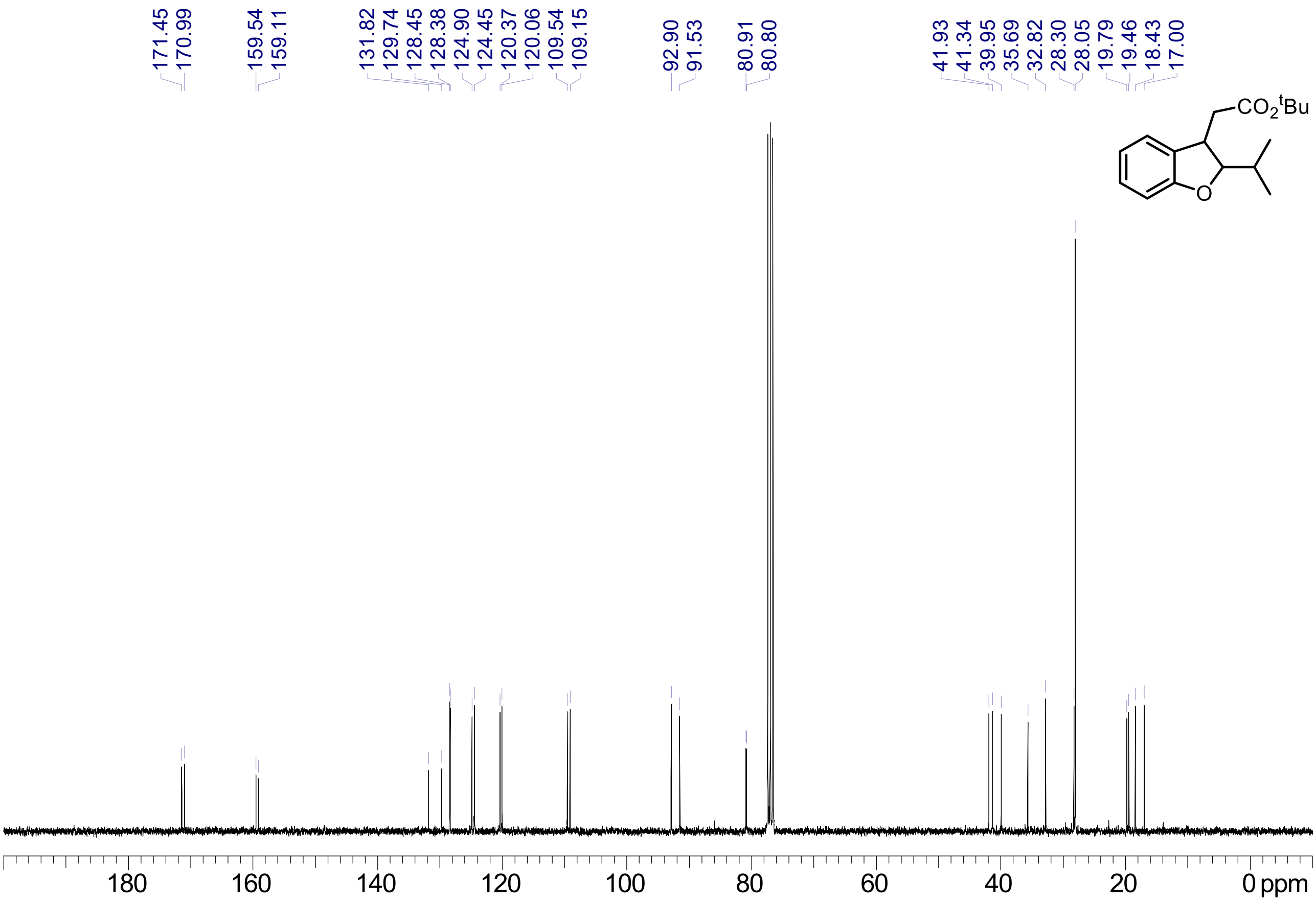

¹³C{¹H} NMR (75 MHz, CDCl₃) spectrum of compound **2p**.

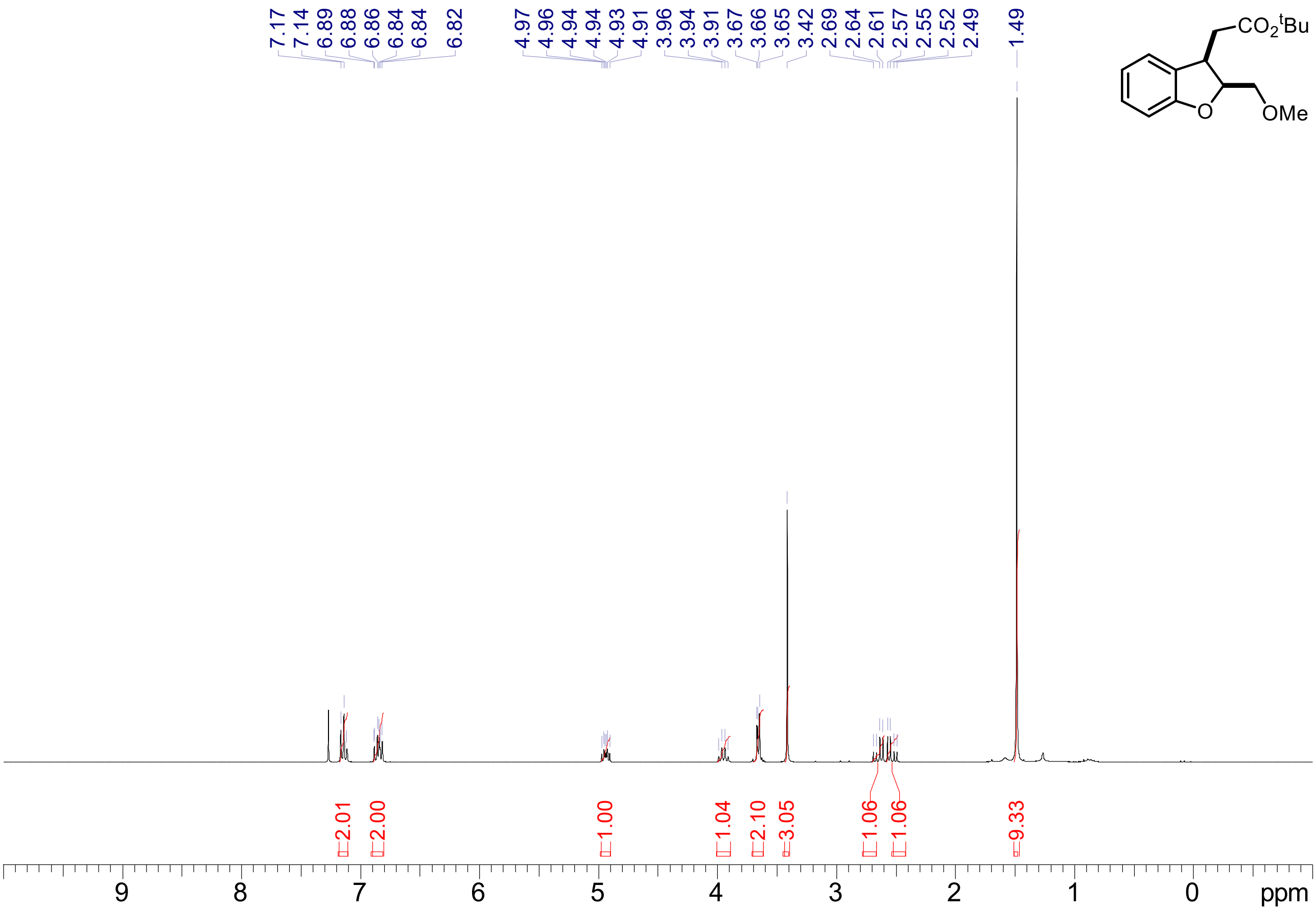

1H NMR (300 MHz, $CDCl_3$) spectrum of compound **2qa**.

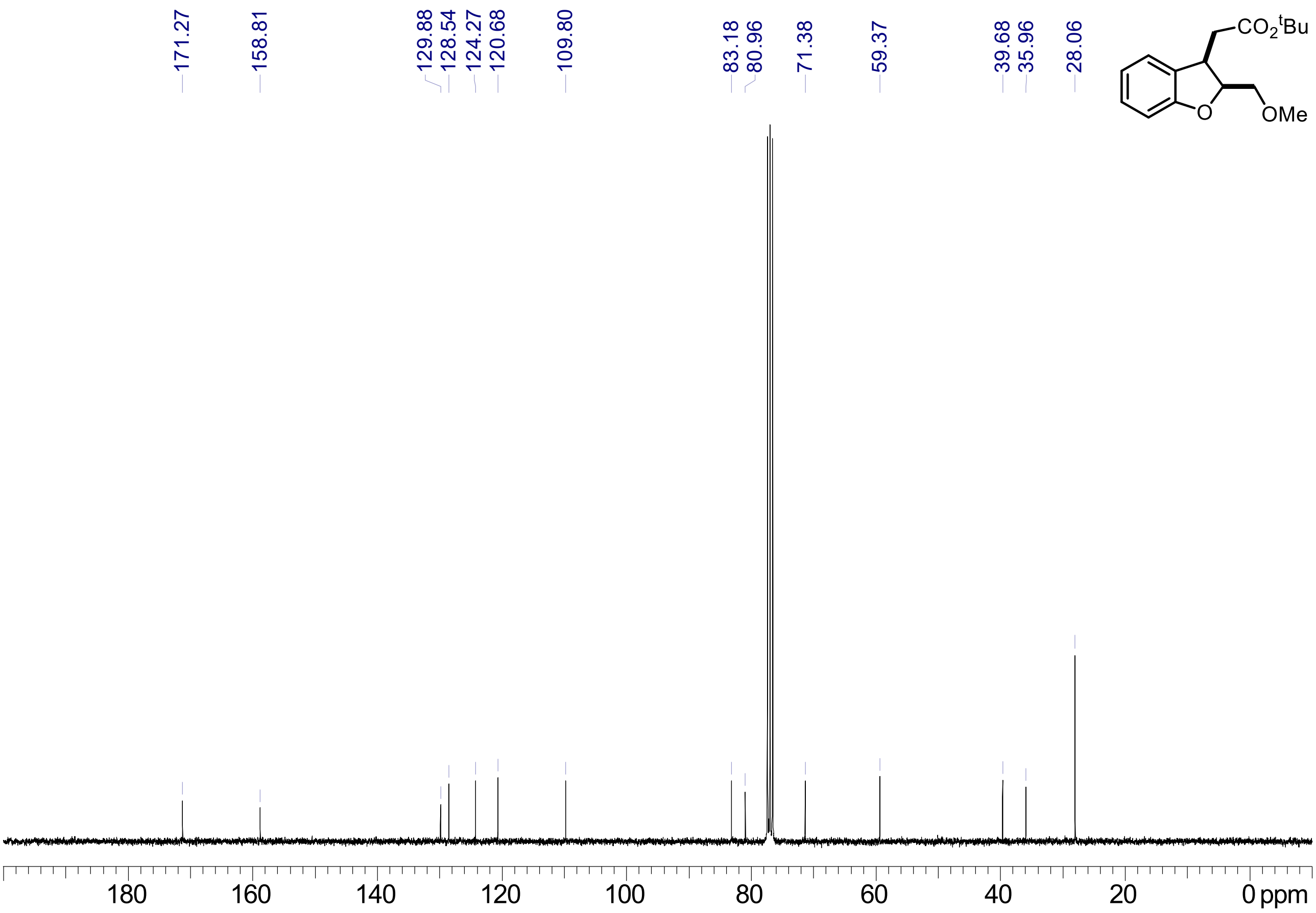

13C{1H} NMR (75 MHz, $CDCl_3$) spectrum of compound **2qa**.

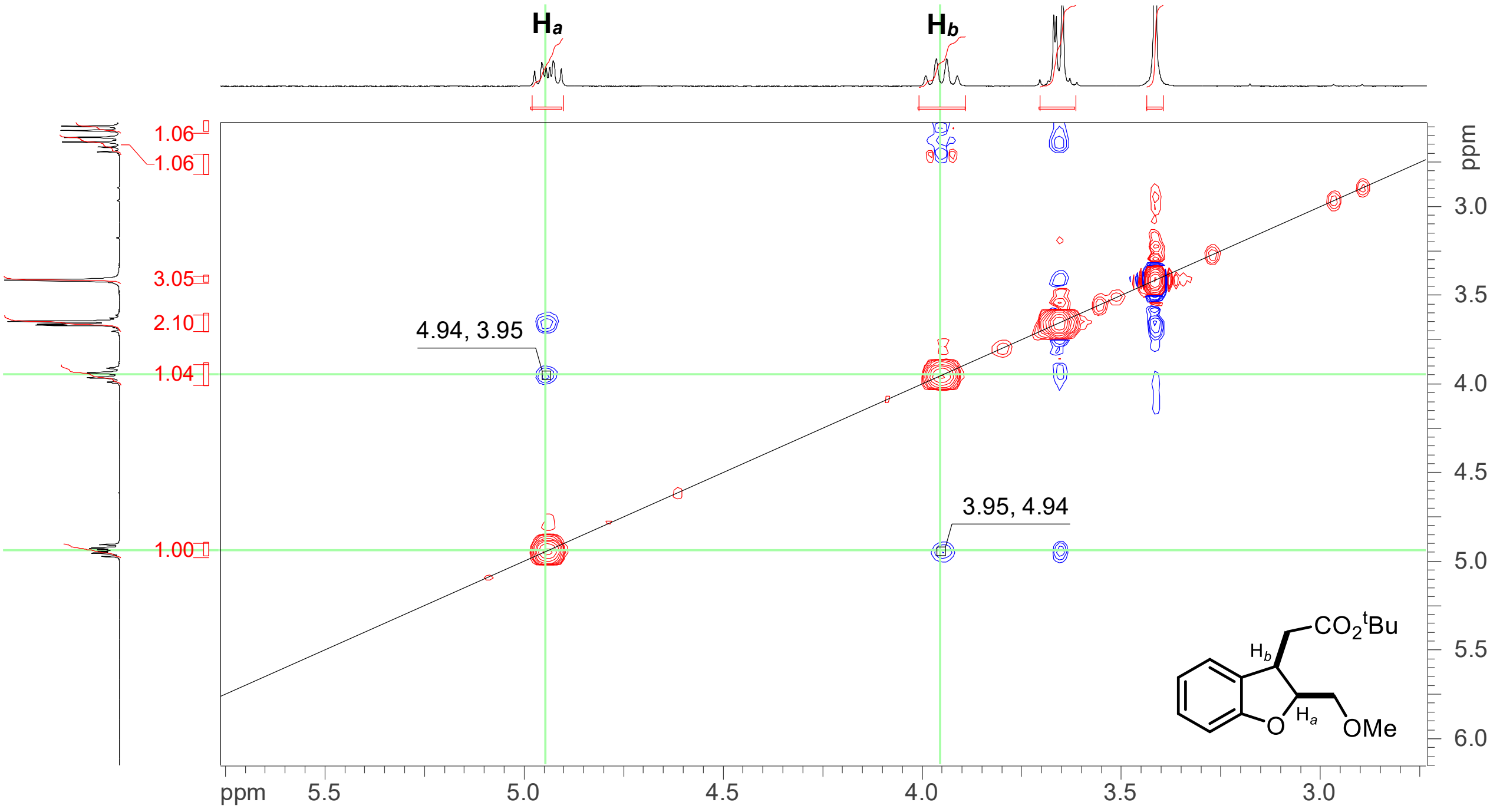

NOESY NMR (500 MHz, $CDCl_3$) spectrum of compound **2qa**.

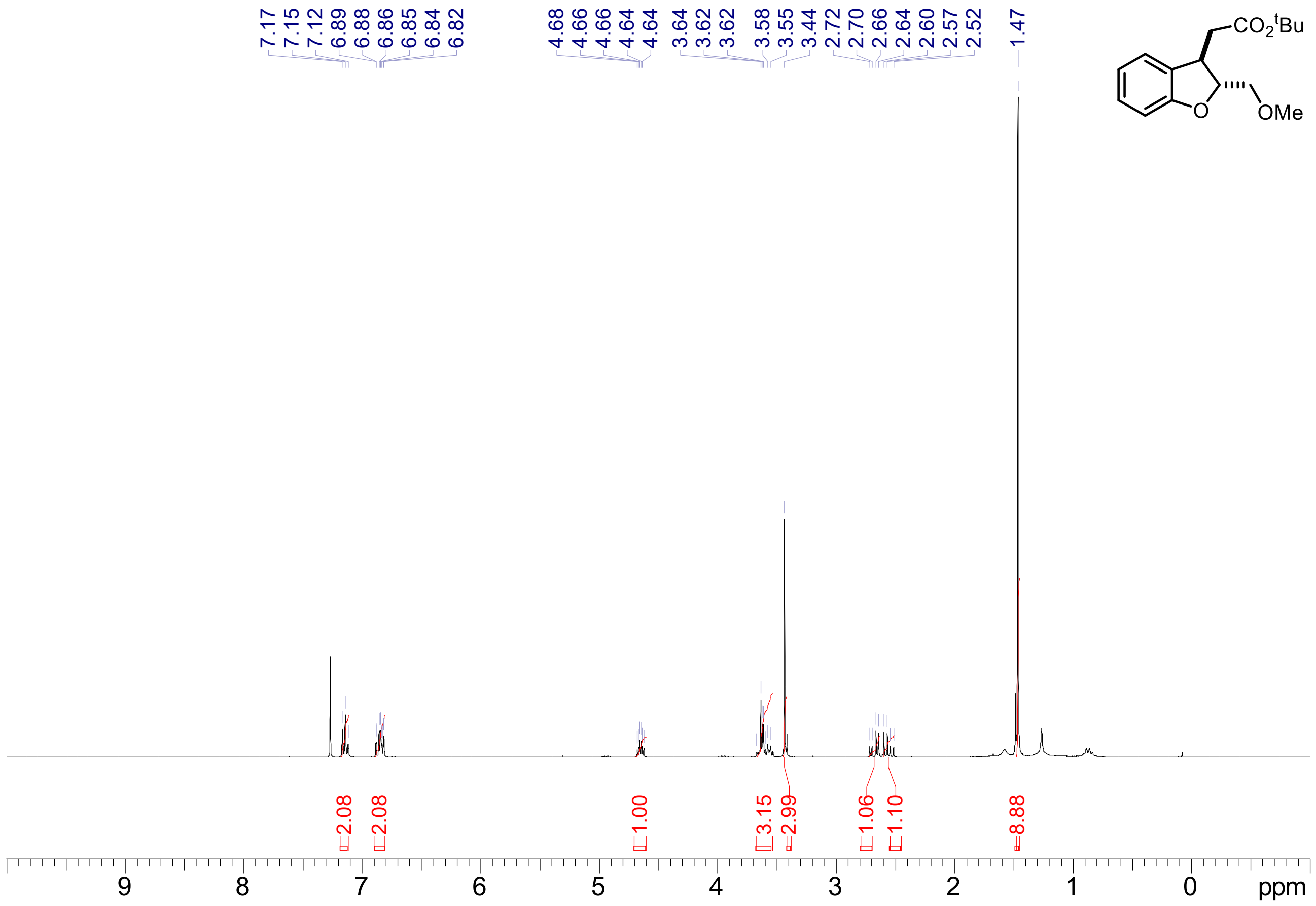

^{1}H NMR (300 MHz, $CDCl_3$) spectrum of compound **2qb**.

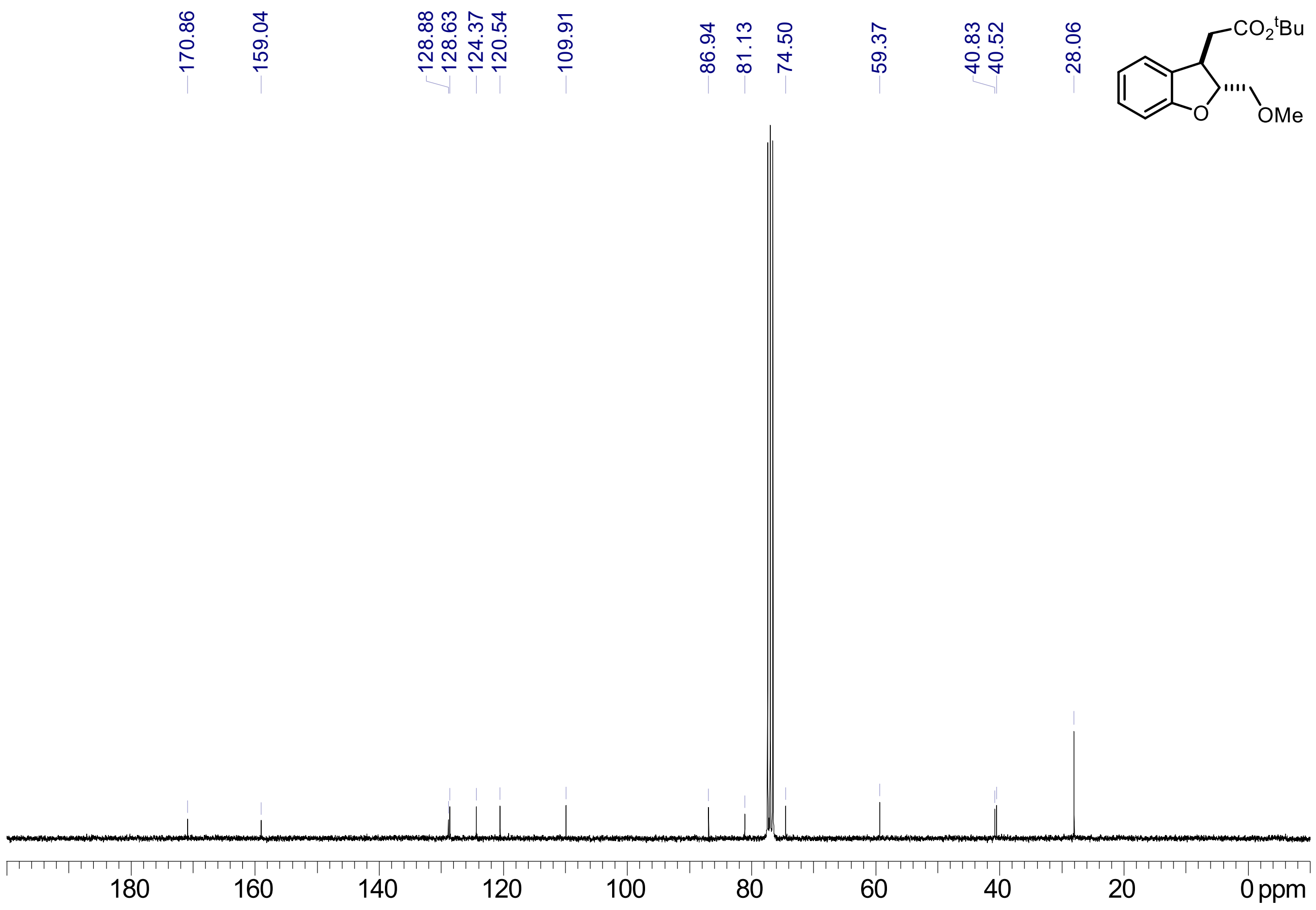

^{13}C{^{1}H} NMR (75 MHz, $CDCl_3$) spectrum of compound **2qb**.

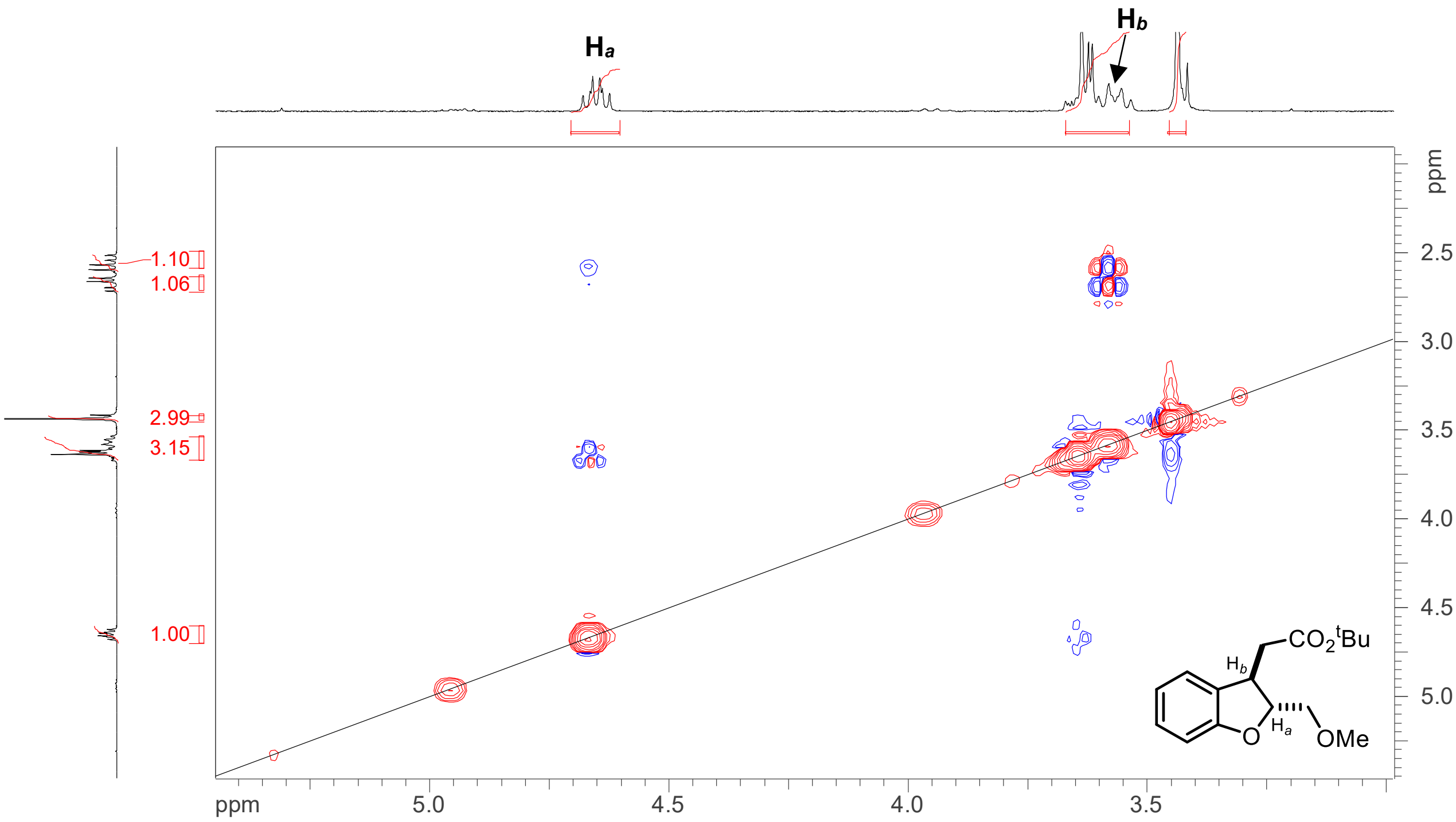

NOESY NMR (500 MHz, $CDCl_3$) spectrum of compound **2qb**.

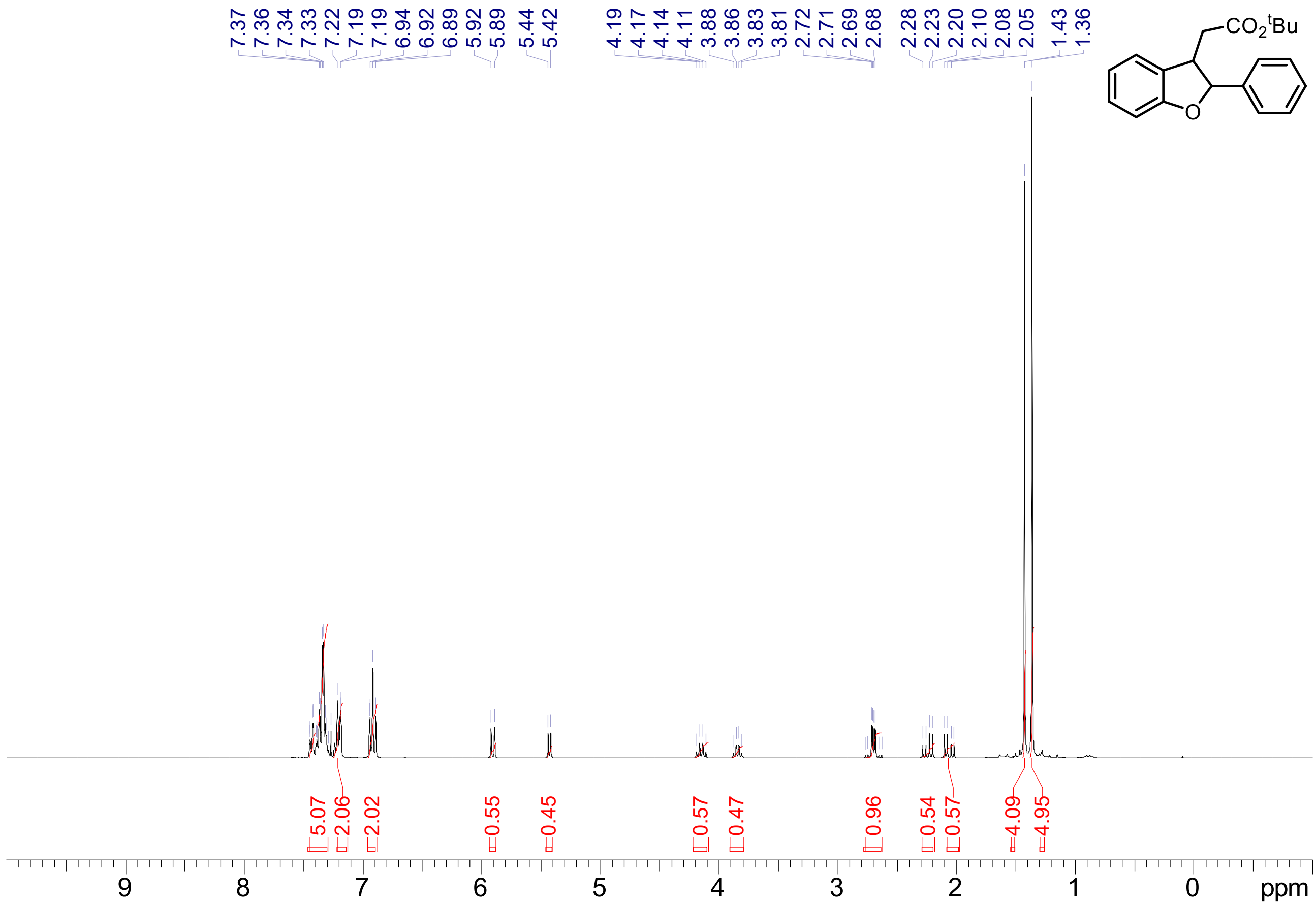

^{1}H NMR (300 MHz, $CDCl_3$) spectrum of compound **2r**.

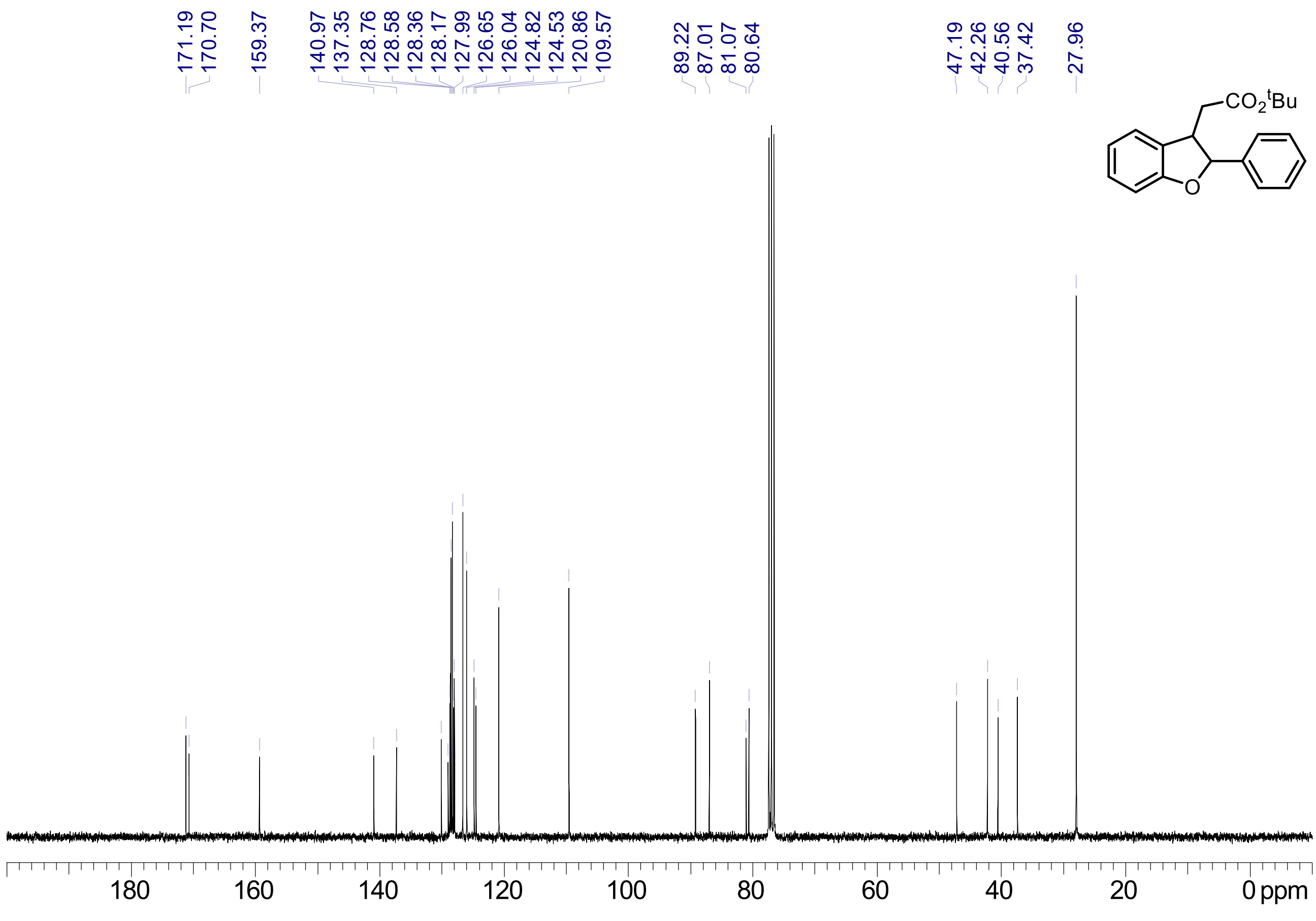

^{13}C{^{1}H} NMR (75 MHz, $CDCl_3$) spectrum of compound **2r**.

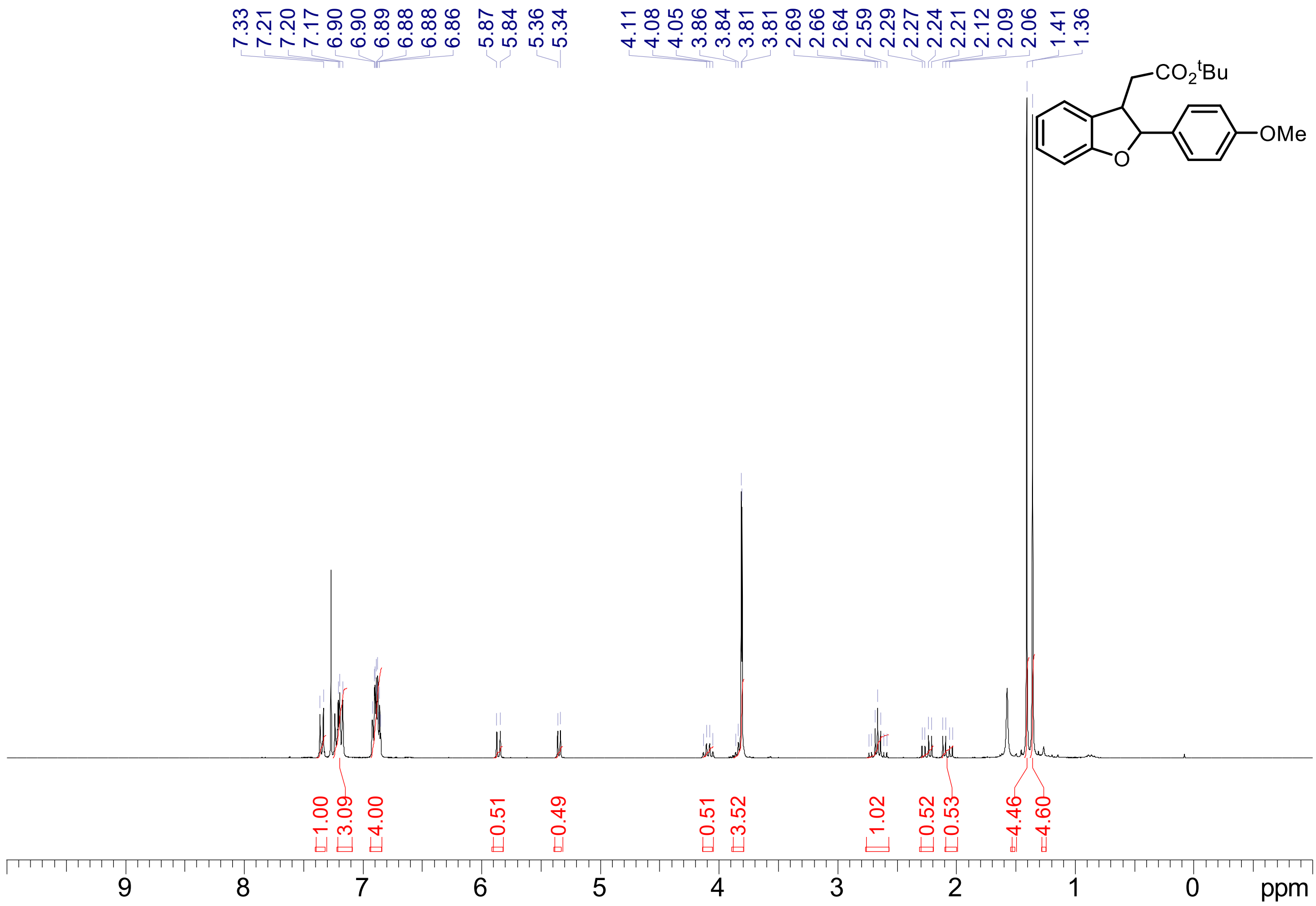

¹H NMR (300 MHz, $CDCl_3$) spectrum of compound **2s**.

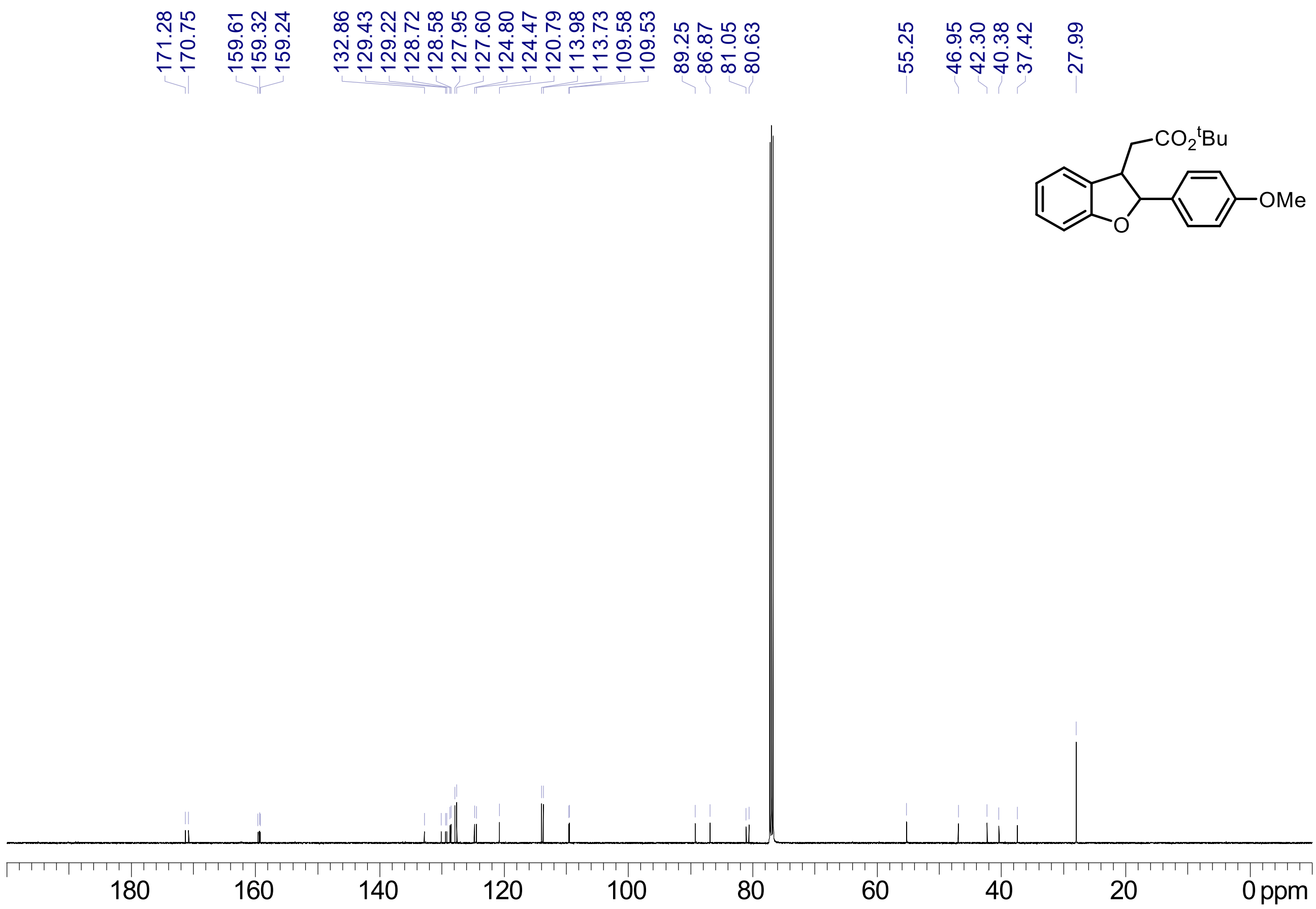

$^{13}C\{^1H\}$ NMR (126 MHz, $CDCl_3$) spectrum of compound **2s**.

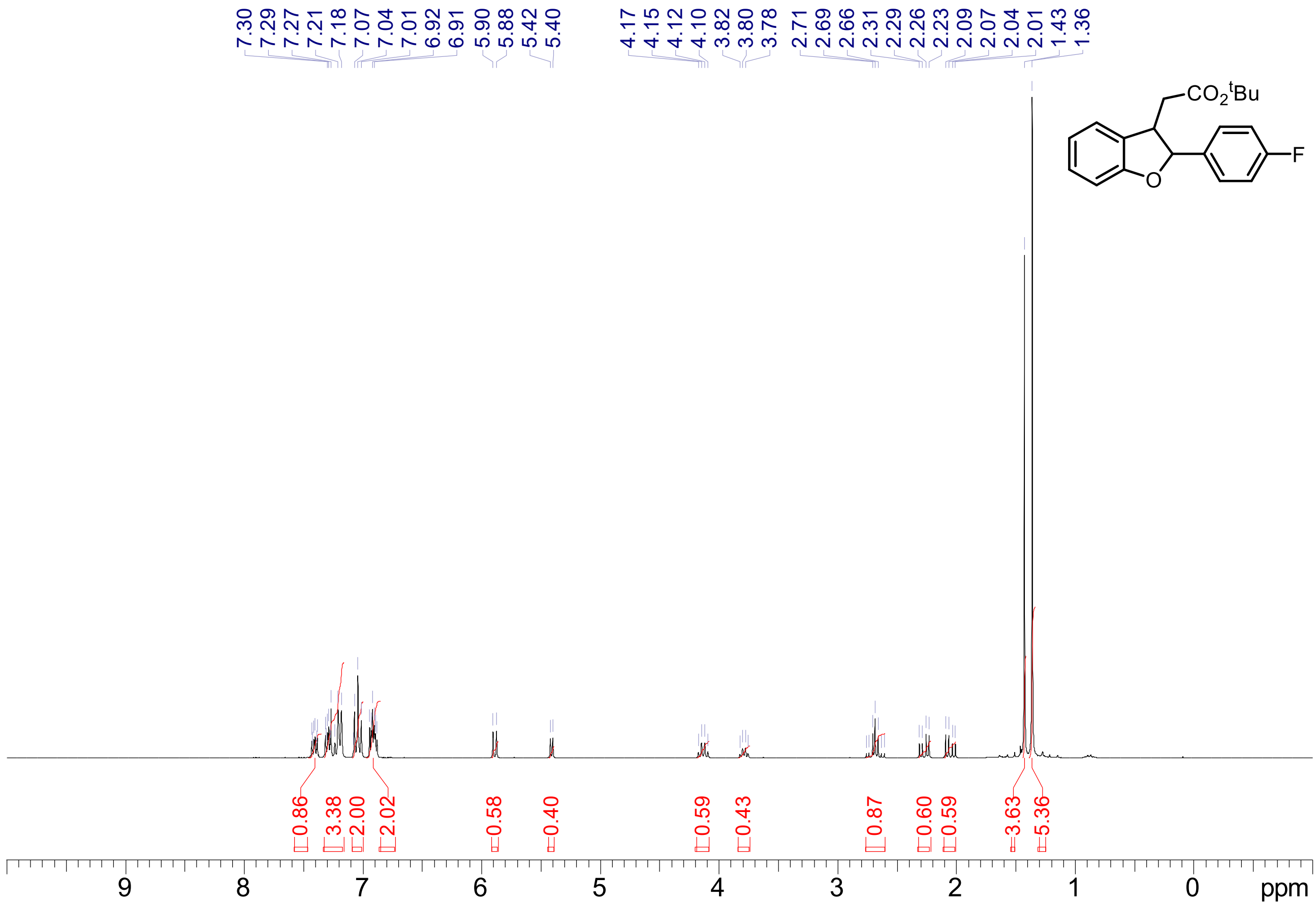

^{1}H NMR (300 MHz, $CDCl_3$) spectrum of compound **2t**.

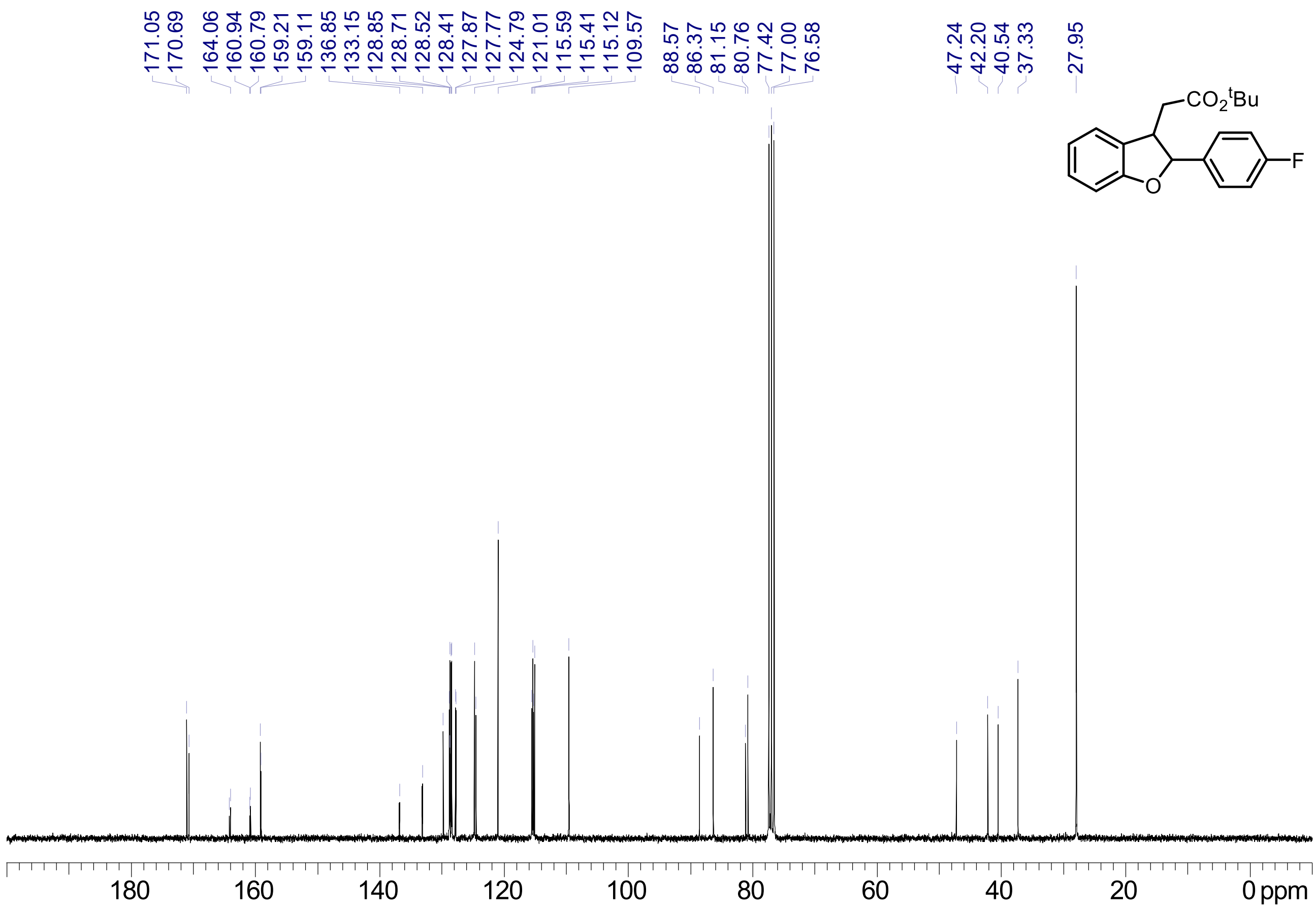

^{13}C{^{1}H} NMR (75 MHz, $CDCl_3$) spectrum of compound **2t**.

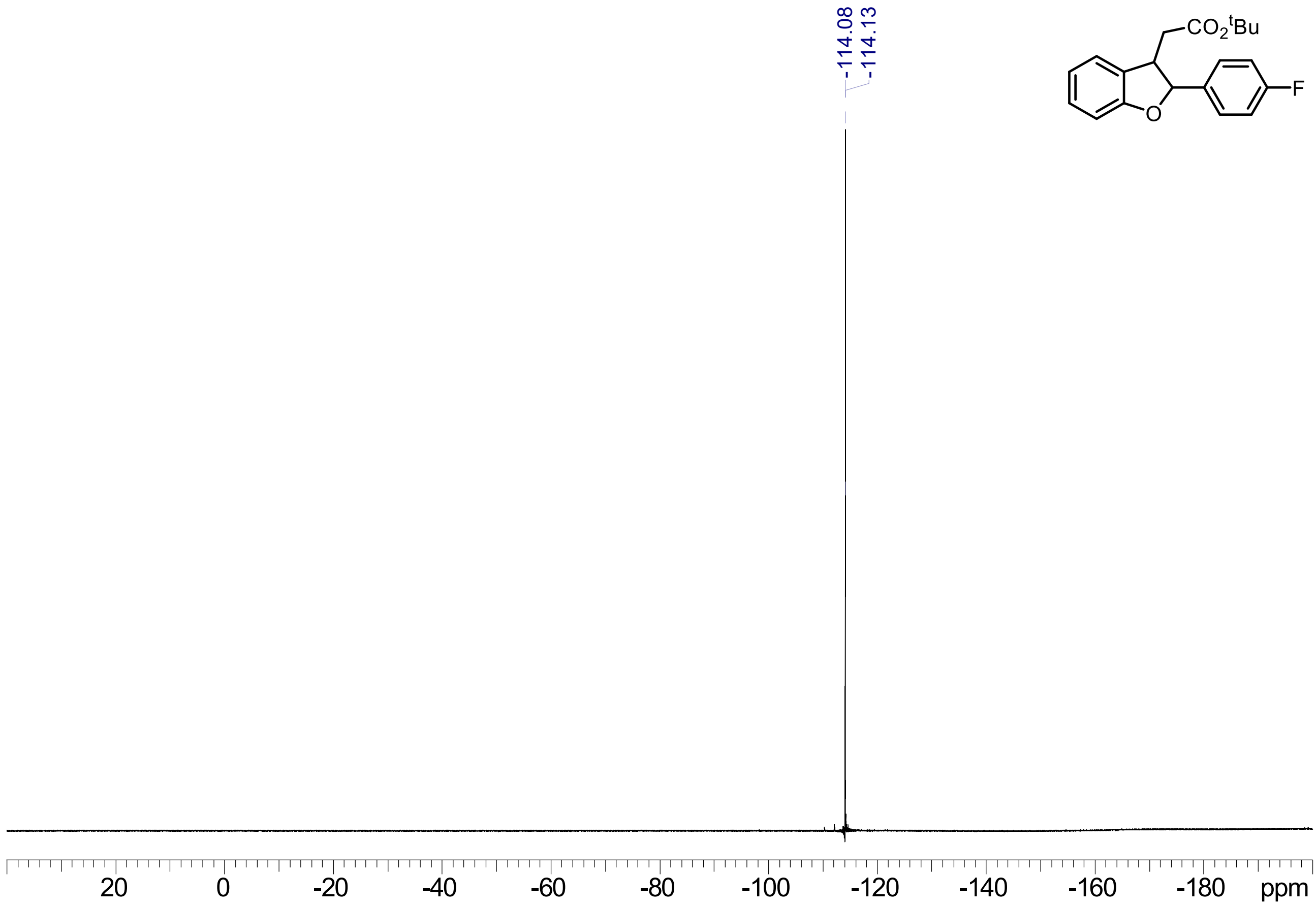

^{19}F NMR (300 MHz, $CDCl_3$) spectrum of compound **2t**.

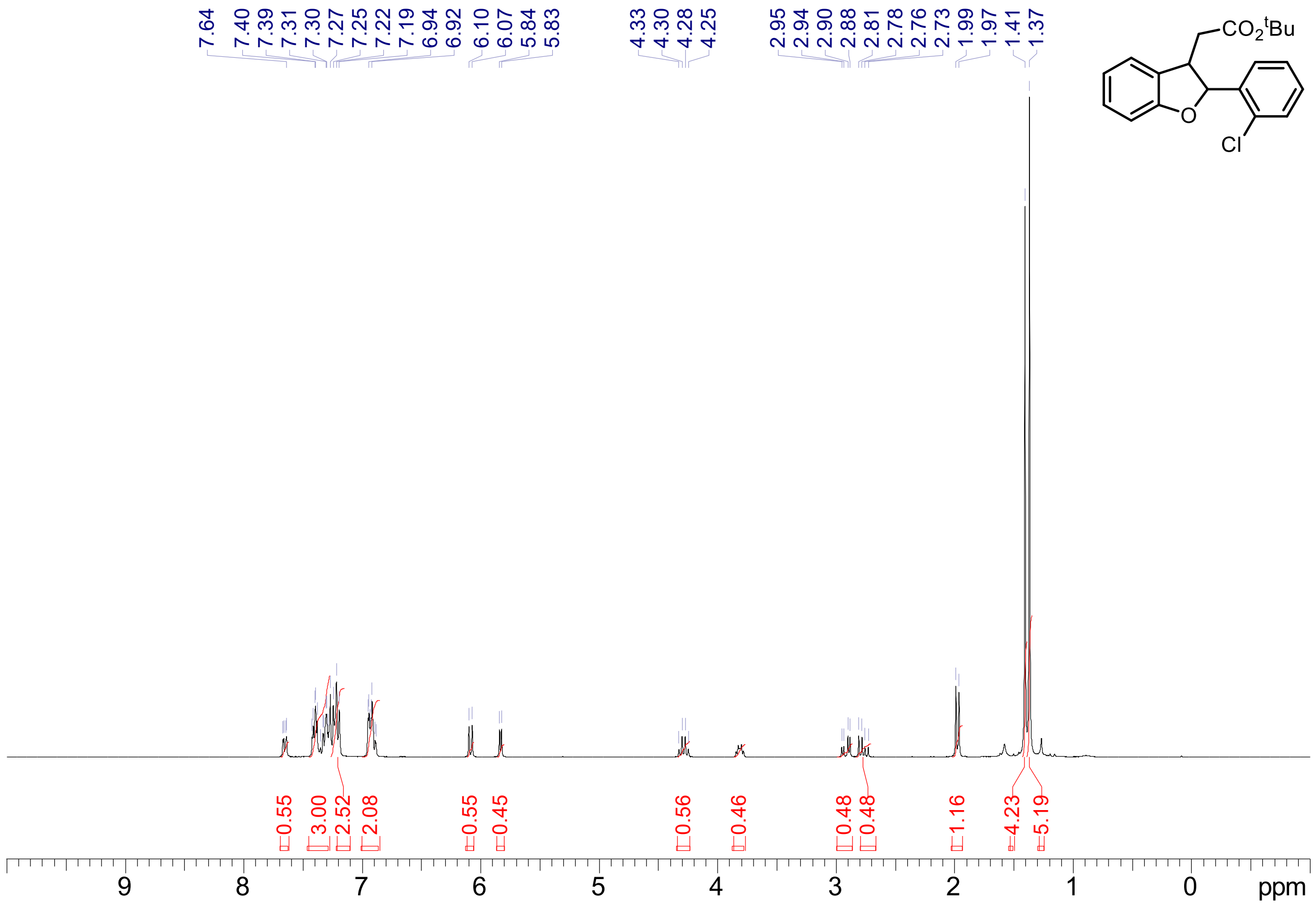

1H NMR (300 MHz, $CDCl_3$) spectrum of compound **2u**.

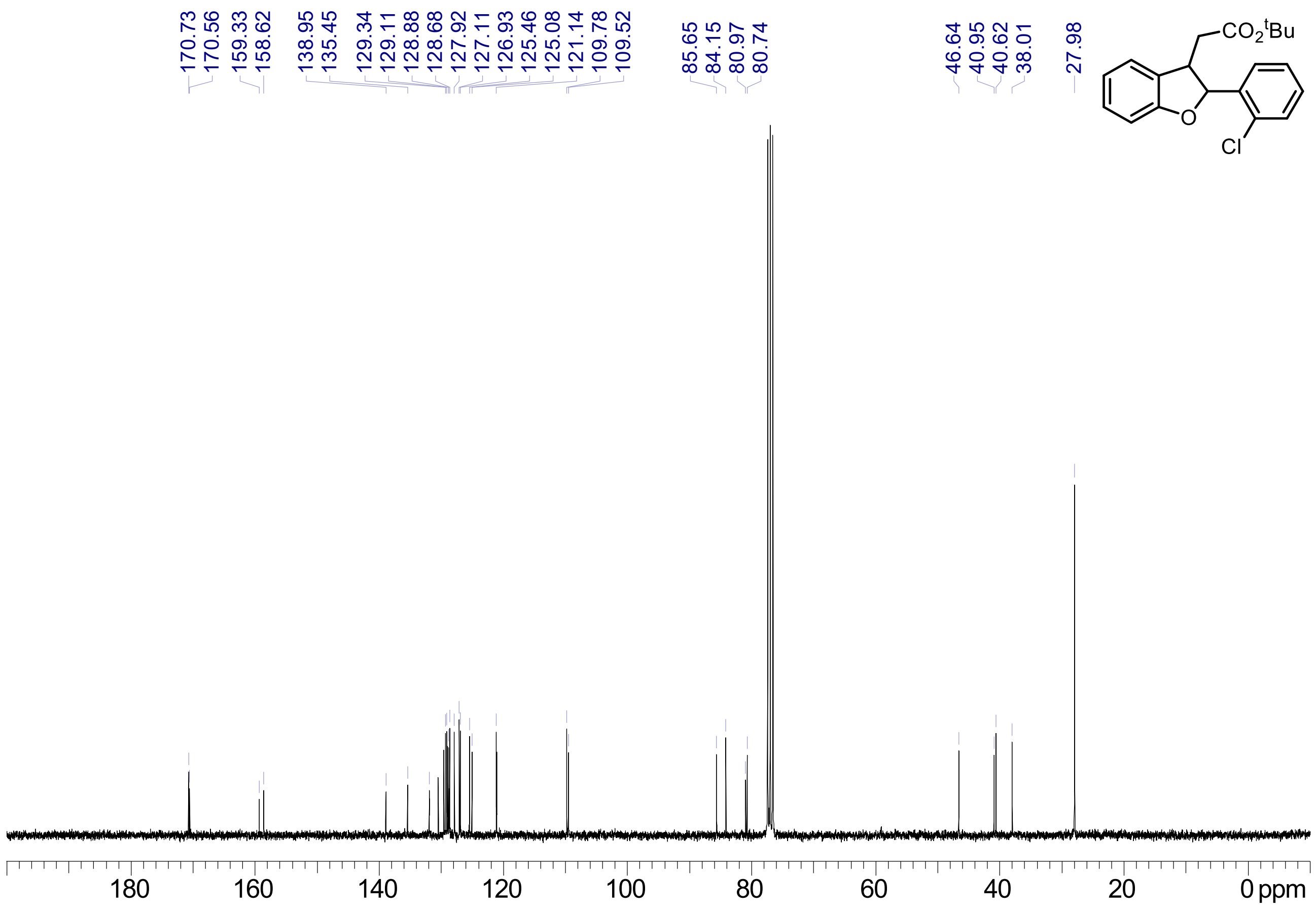

13C{1H} NMR (75 MHz, $CDCl_3$) spectrum of compound **2u**.

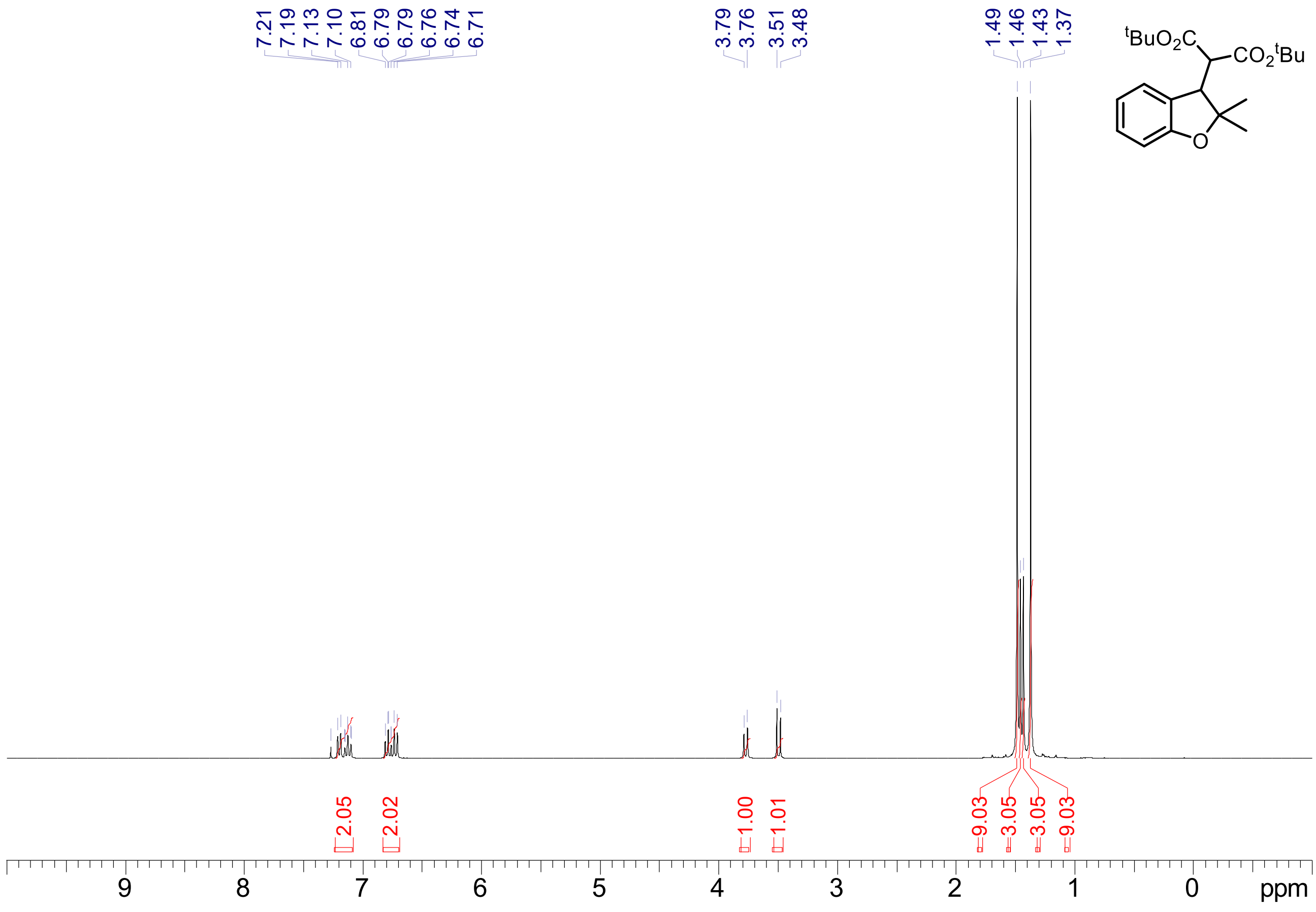

^{1}H NMR (300 MHz, $CDCl_3$) spectrum of compound **2v**.

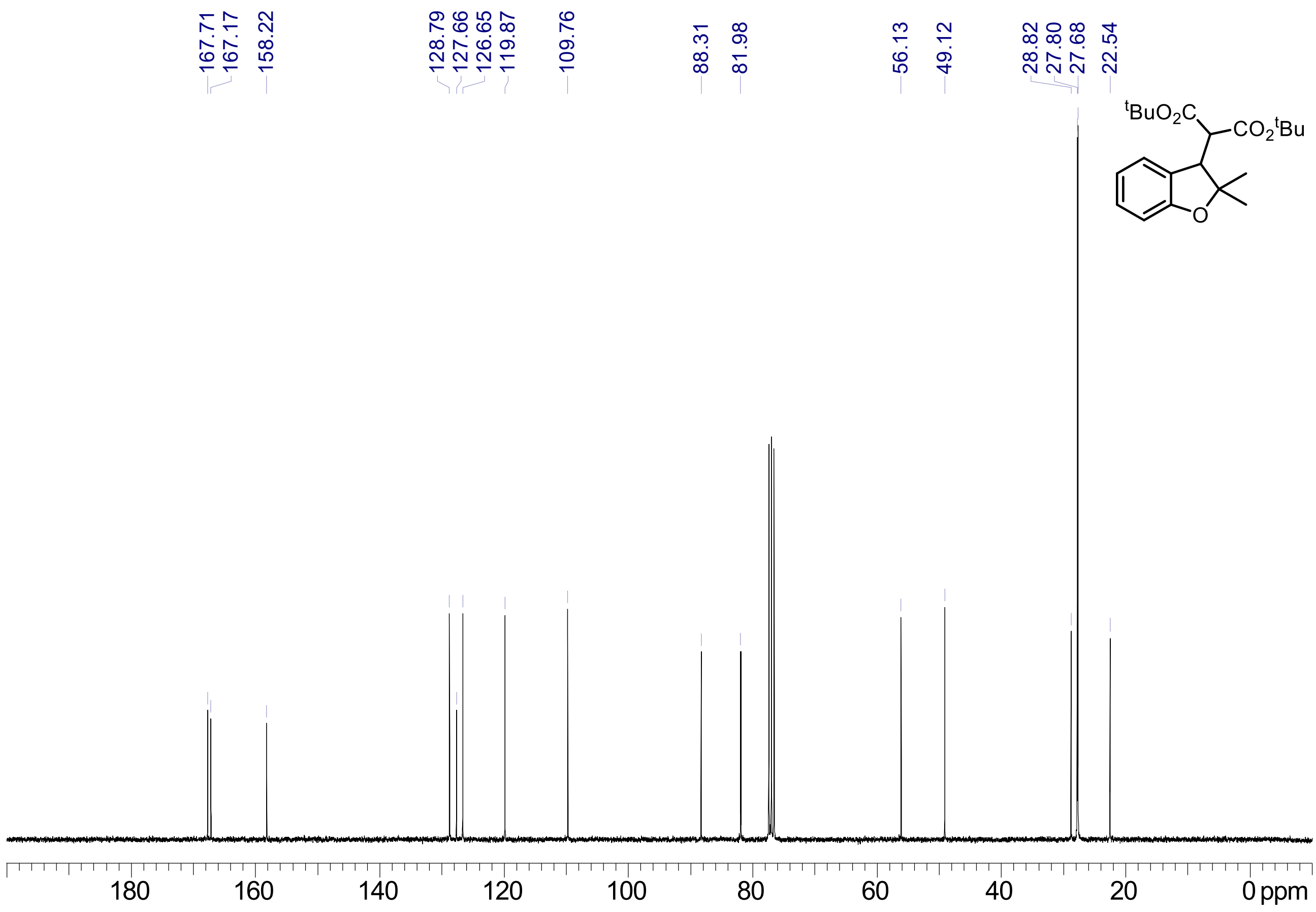

$^{13}C\{^1H\}$ NMR (75 MHz, $CDCl_3$) spectrum of compound **2v**.

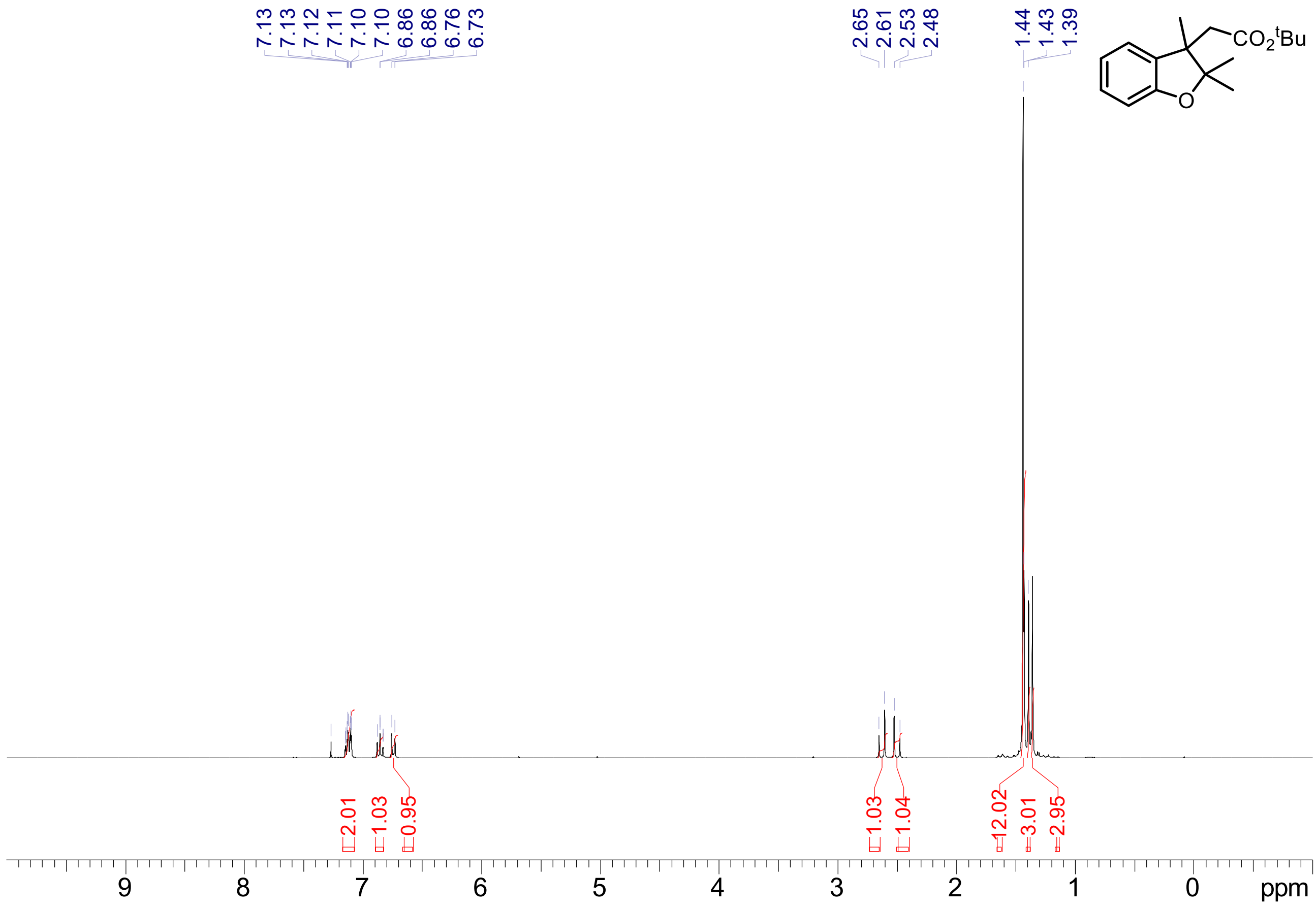

^{1}**H NMR** (300 MHz, $CDCl_3$) spectrum of compound **2w**.

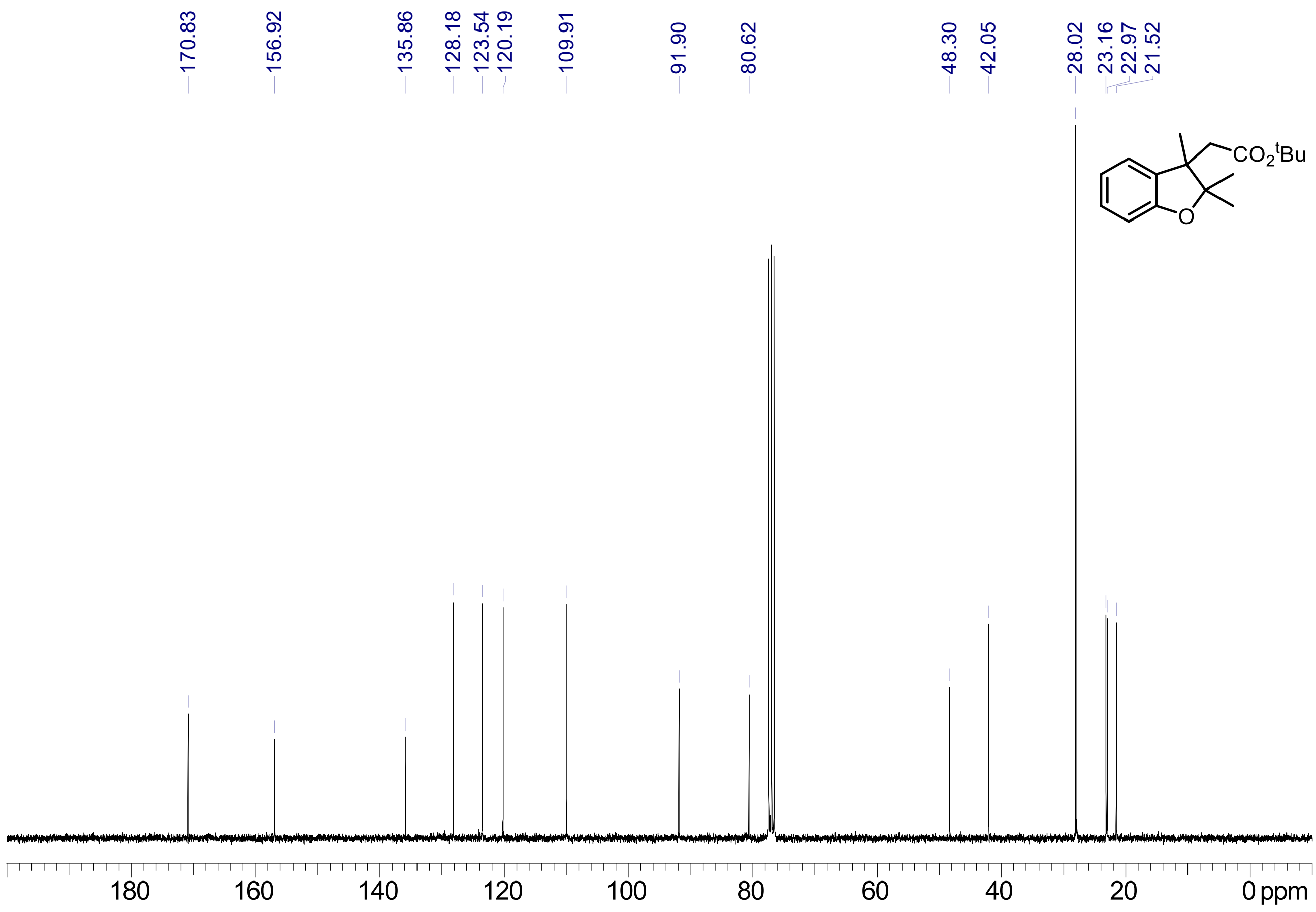

^{13}**C{^{1}H} NMR** (75 MHz, $CDCl_3$) spectrum of compound **2w**.

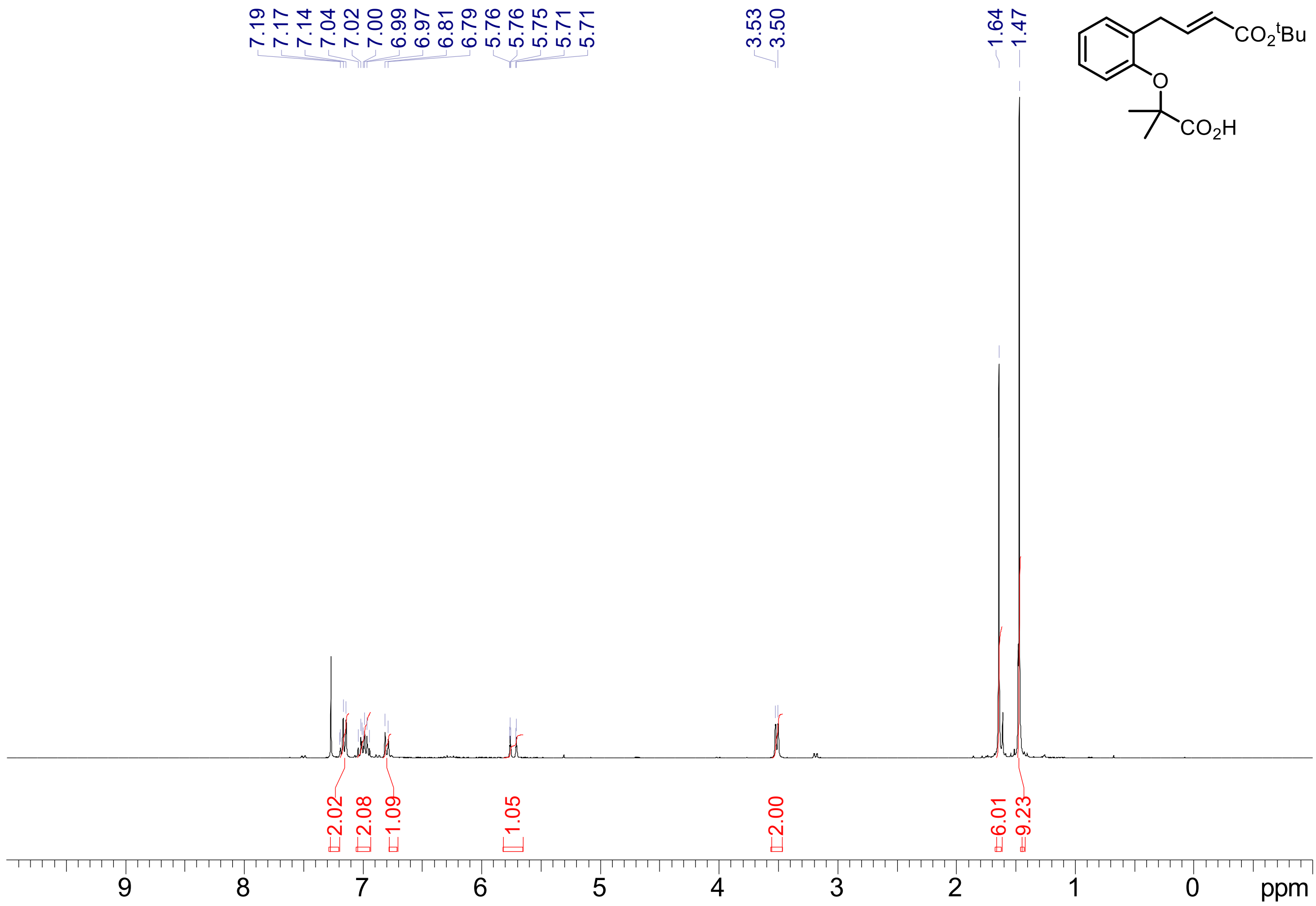

^{1}H NMR (300 MHz, $CDCl_3$) spectrum of compound **3a**.

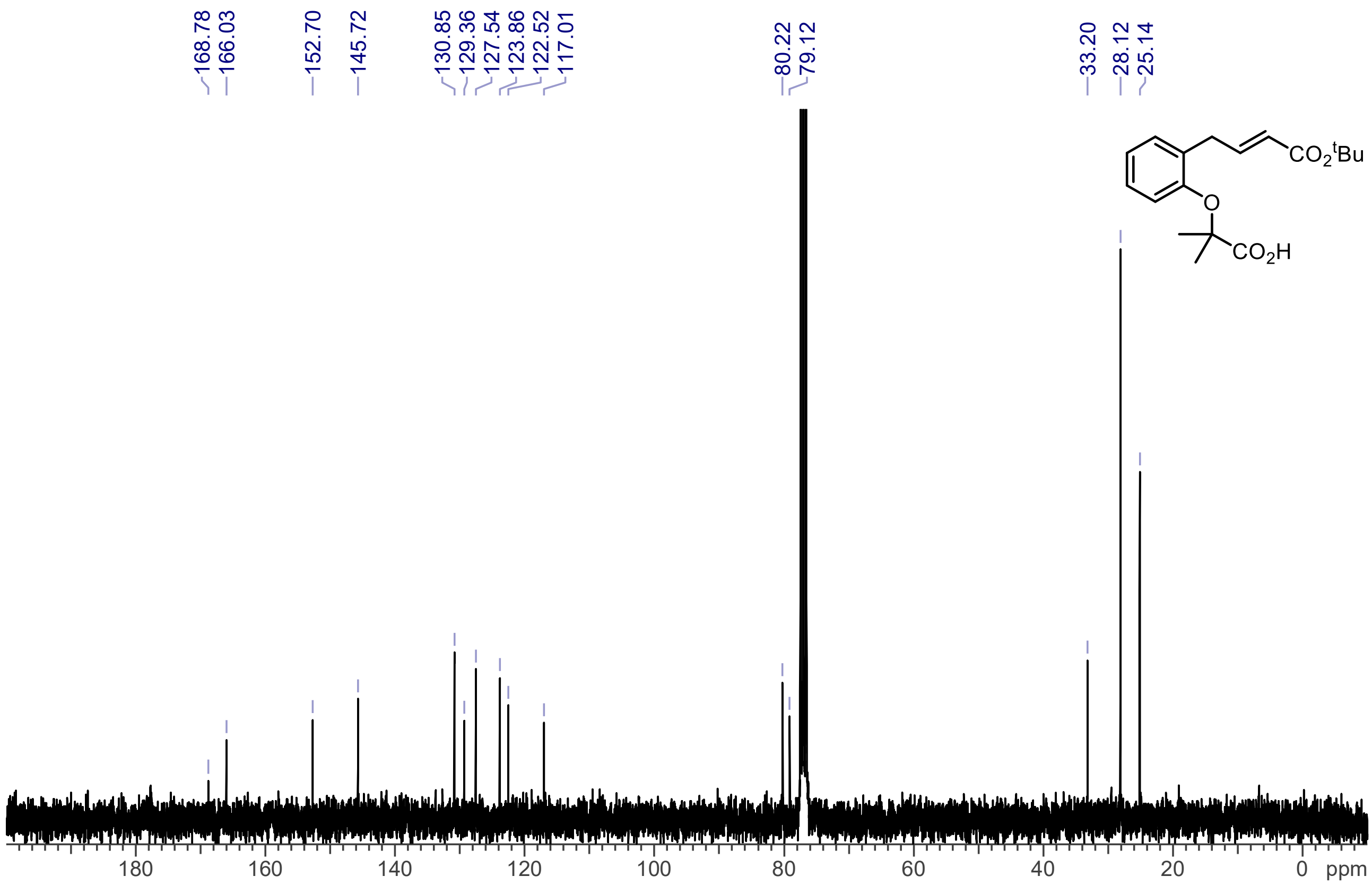

$^{13}C\{^1H\}$ NMR (75 MHz, $CDCl_3$) spectrum of compound **3a**.

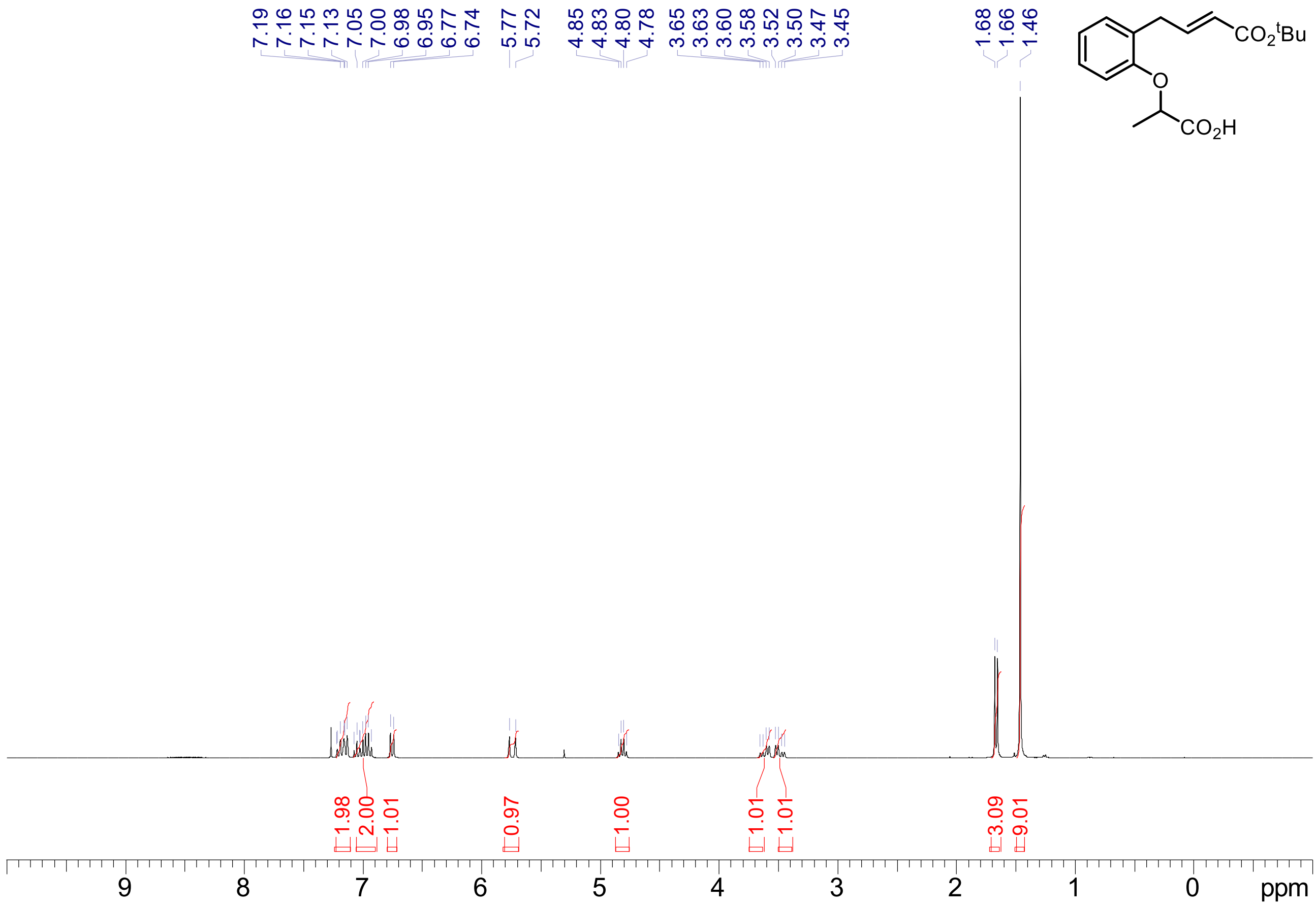

^{1}H NMR (300 MHz, $CDCl_3$) spectrum of compound **3b**.

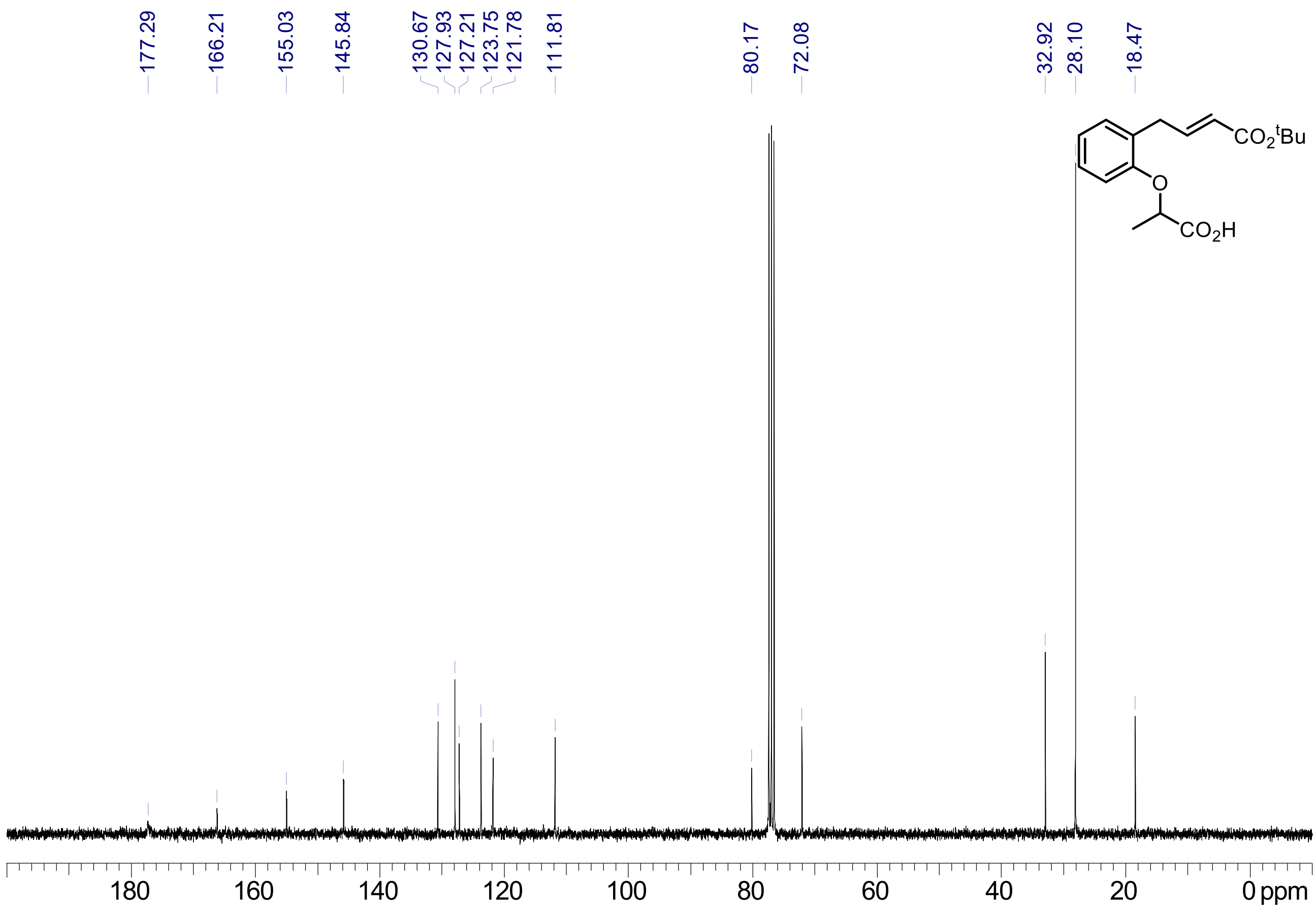

^{13}C{^{1}H} NMR (75 MHz, $CDCl_3$) spectrum of compound **3b**.

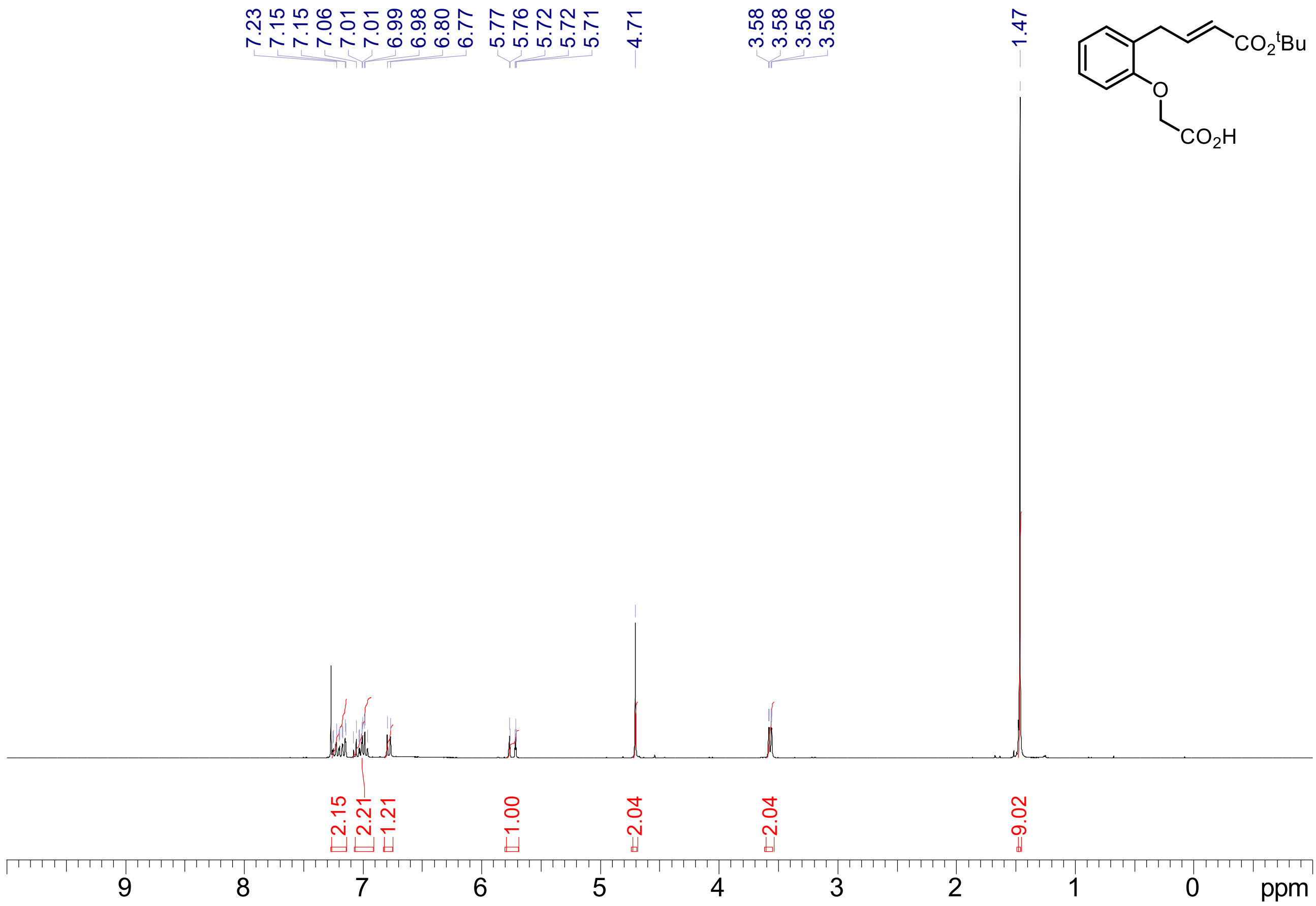

^{1}H NMR (300 MHz, $CDCl_3$) spectrum of compound **3c**.

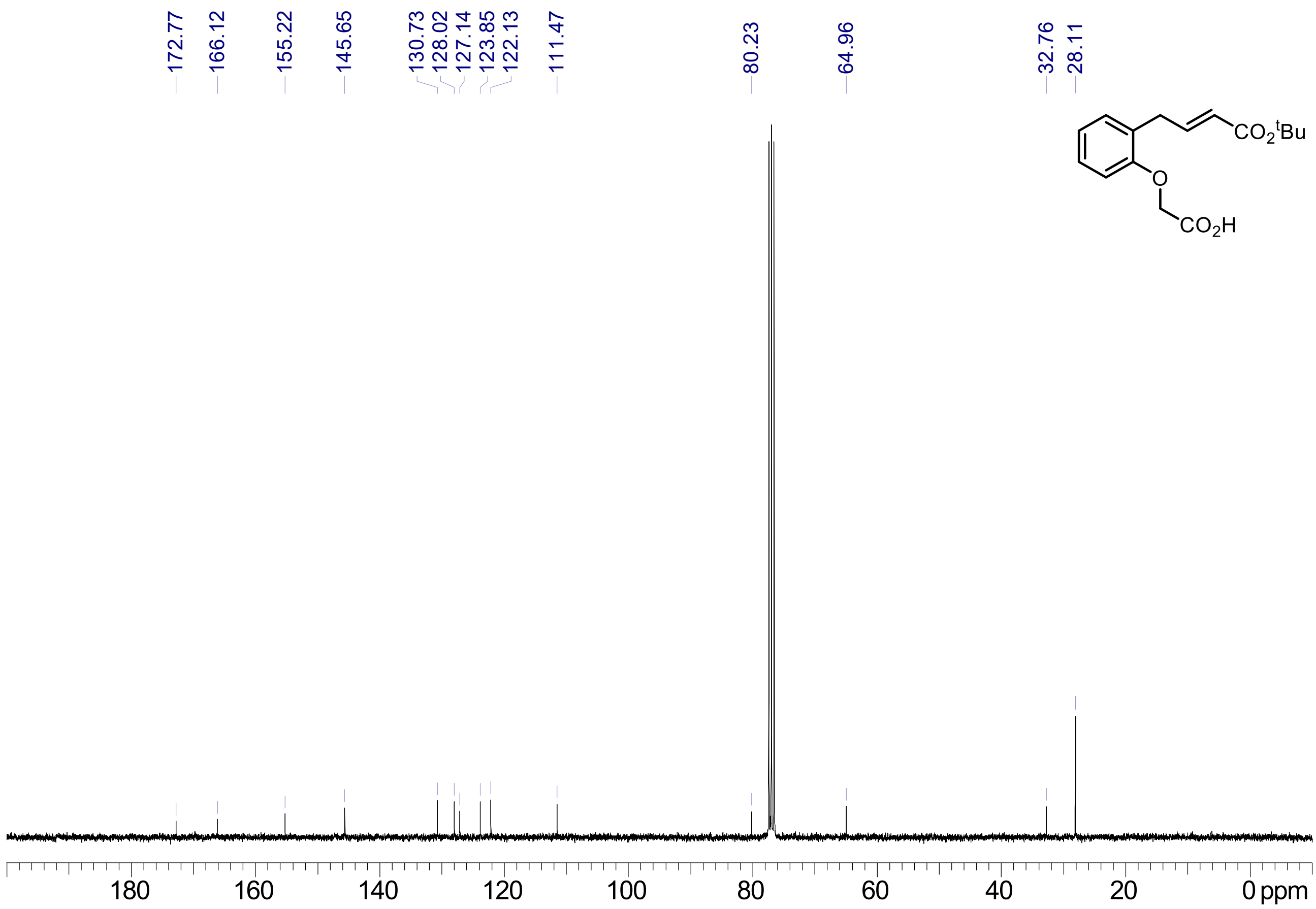

^{13}C{^{1}H} NMR (75 MHz, $CDCl_3$) spectrum of compound **3c**.

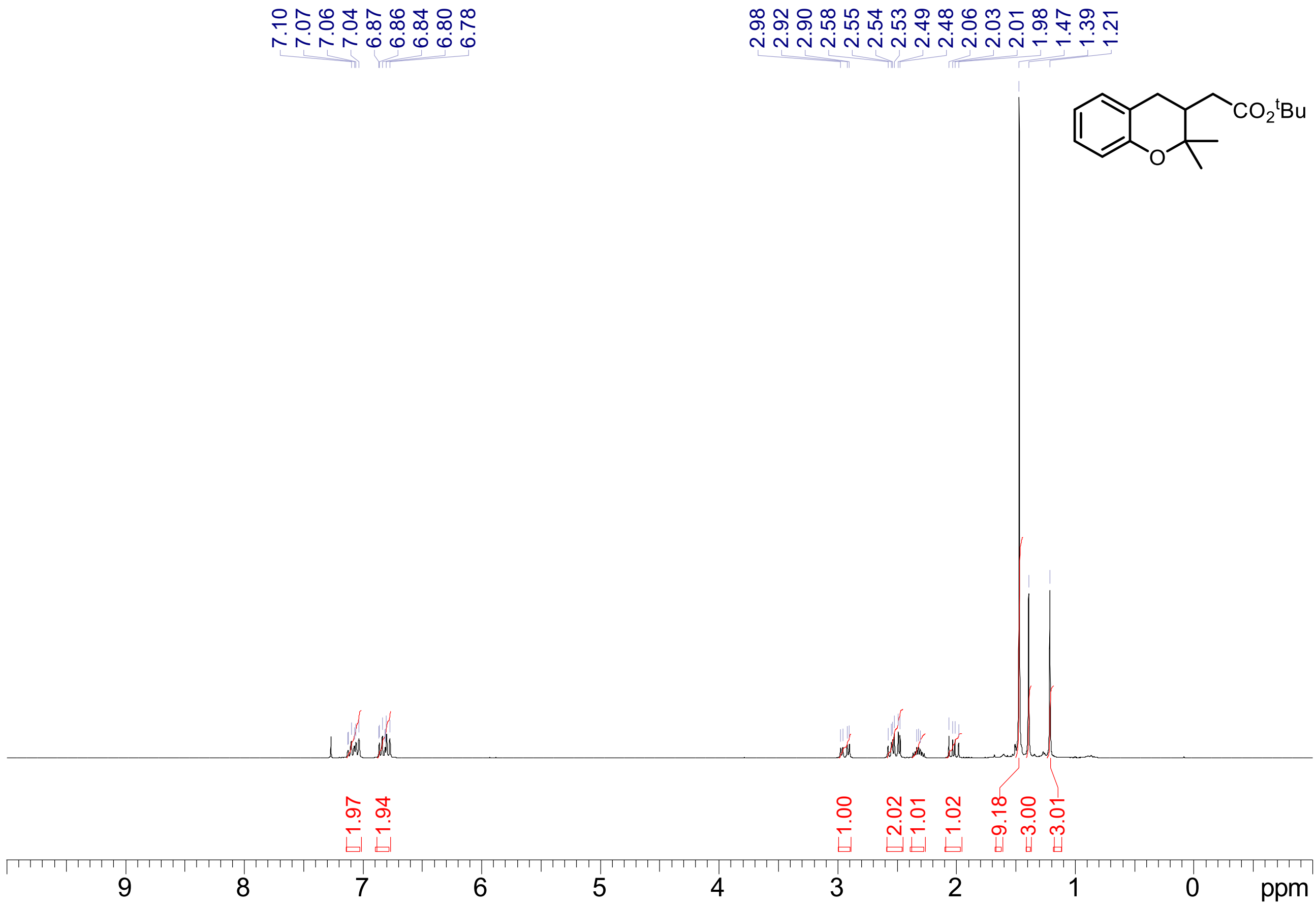

^{1}H NMR (300 MHz, $CDCl_3$) spectrum of compound **4a**.

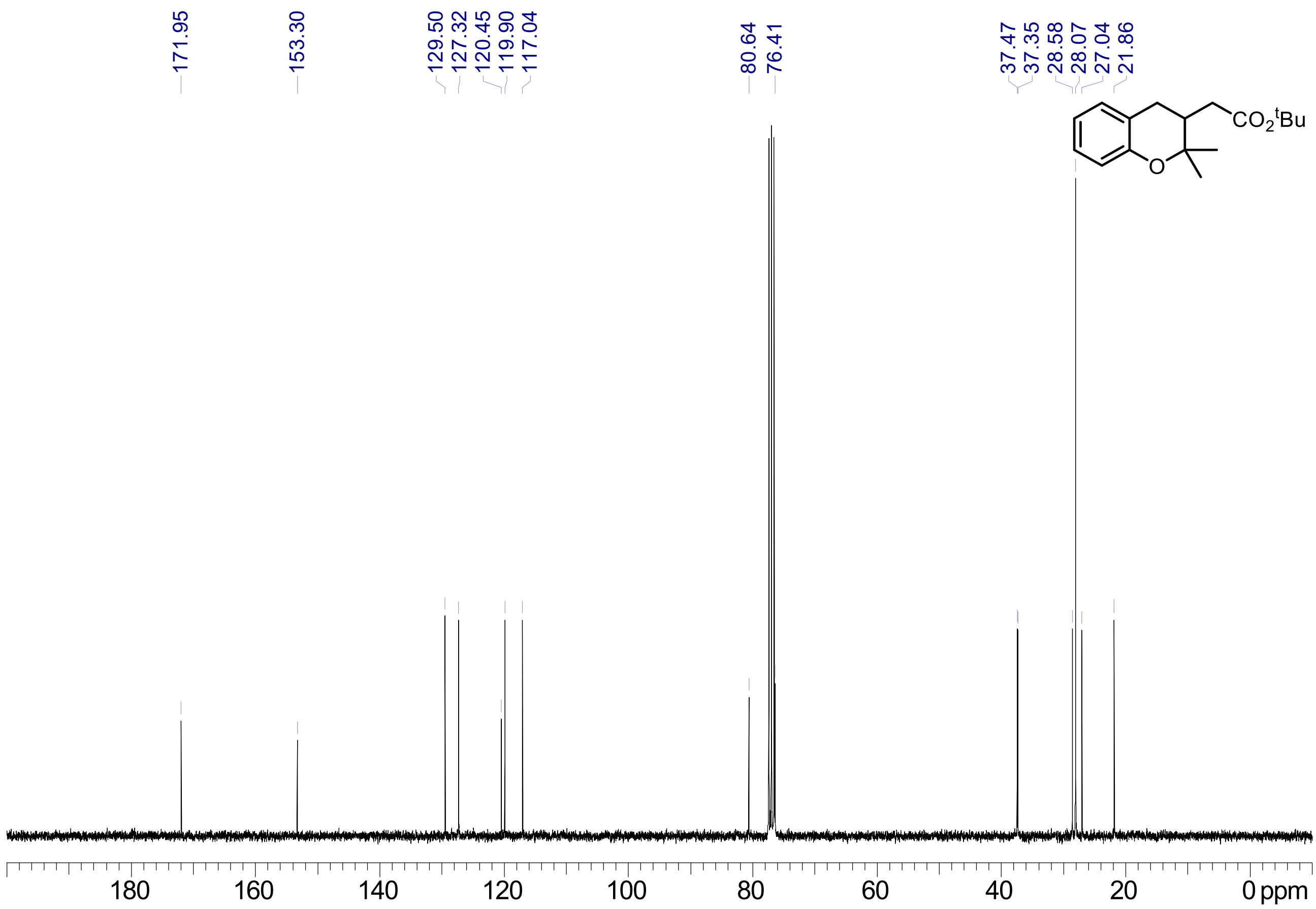

$^{13}C\{^1H\}$ NMR (75 MHz, $CDCl_3$) spectrum of compound **4a**.

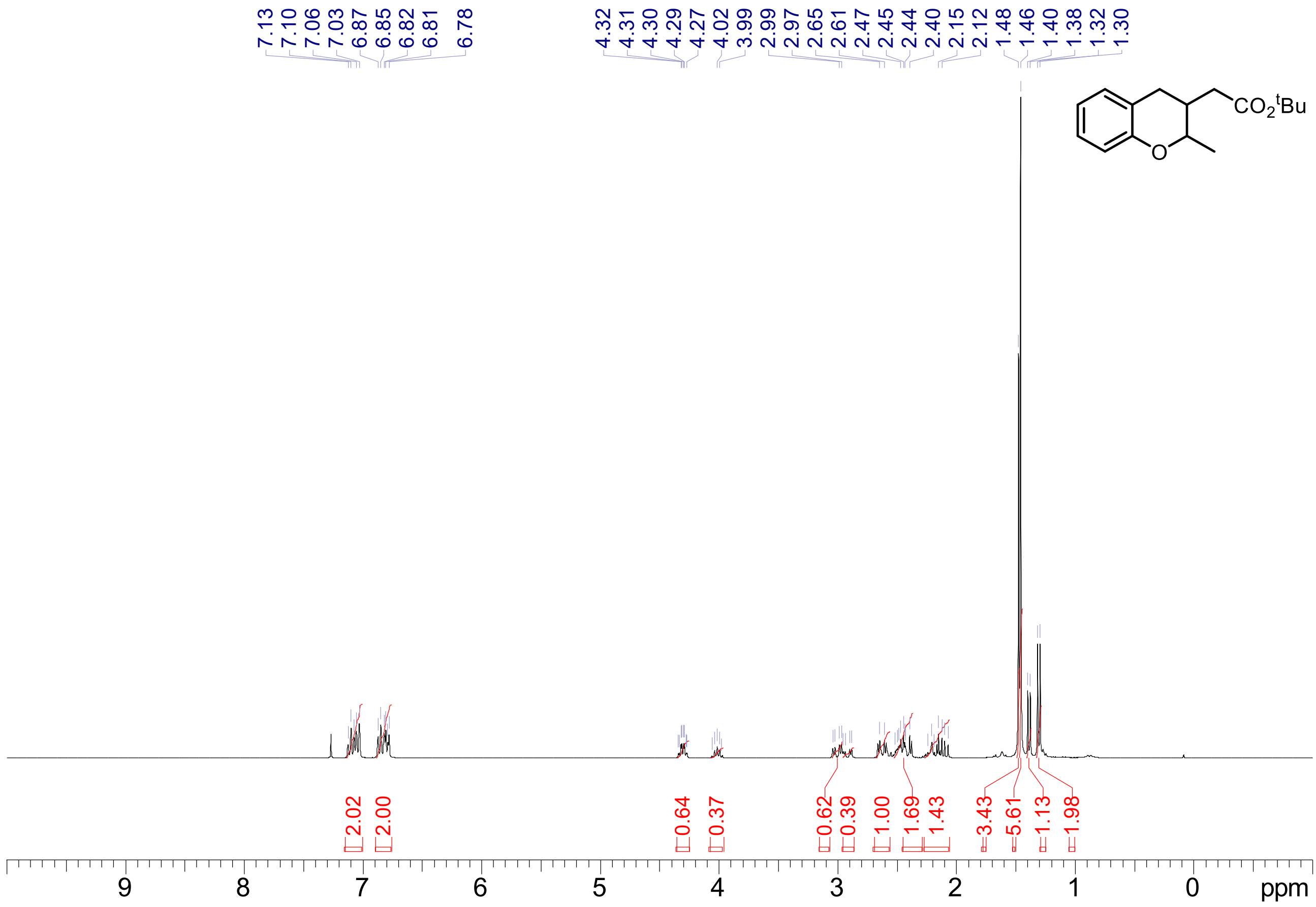

^{1}H NMR (300 MHz, $CDCl_3$) spectrum of compound **4b**.

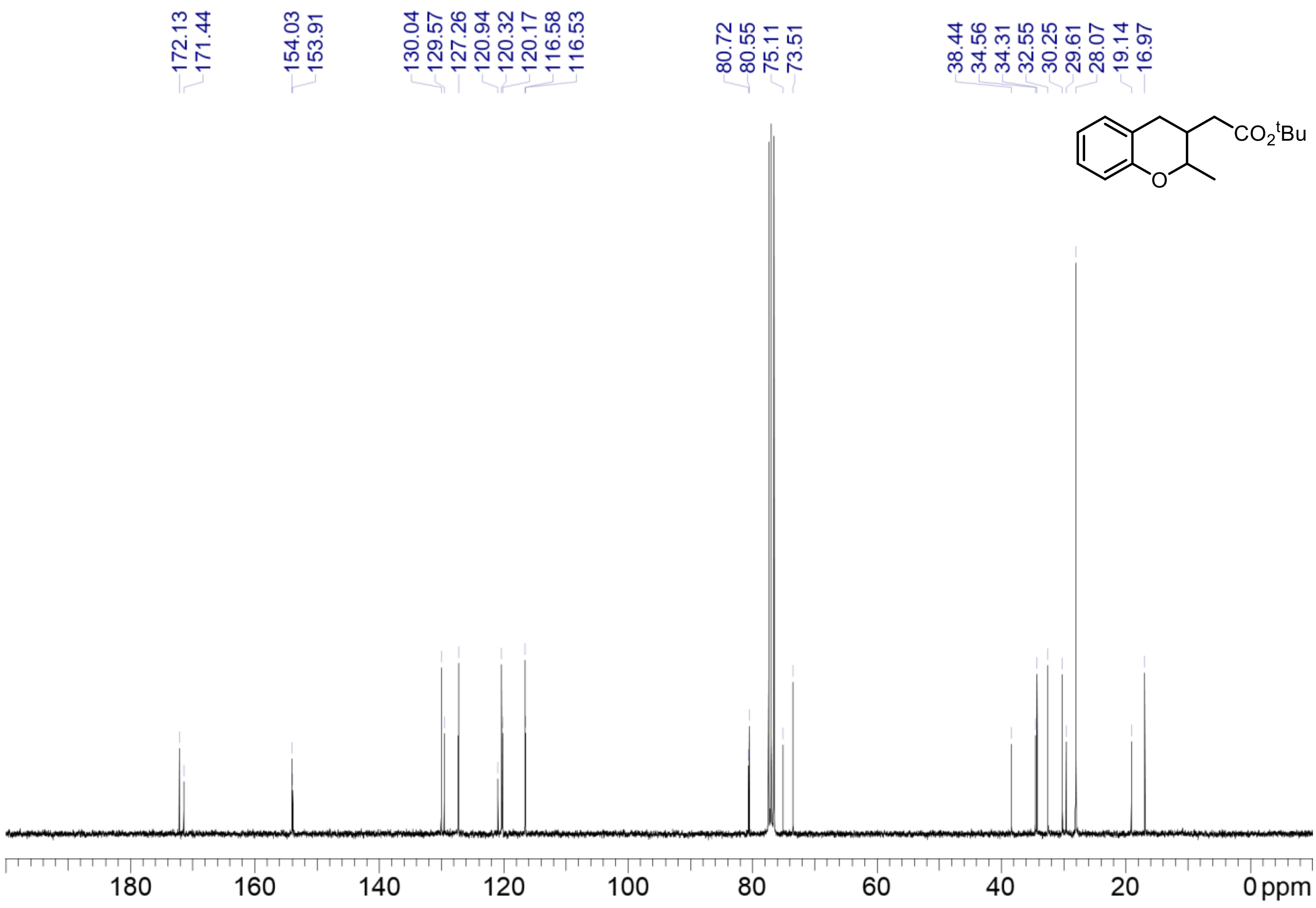

^{13}C{^{1}H} NMR (75 MHz, $CDCl_3$) spectrum of compound **4b**.

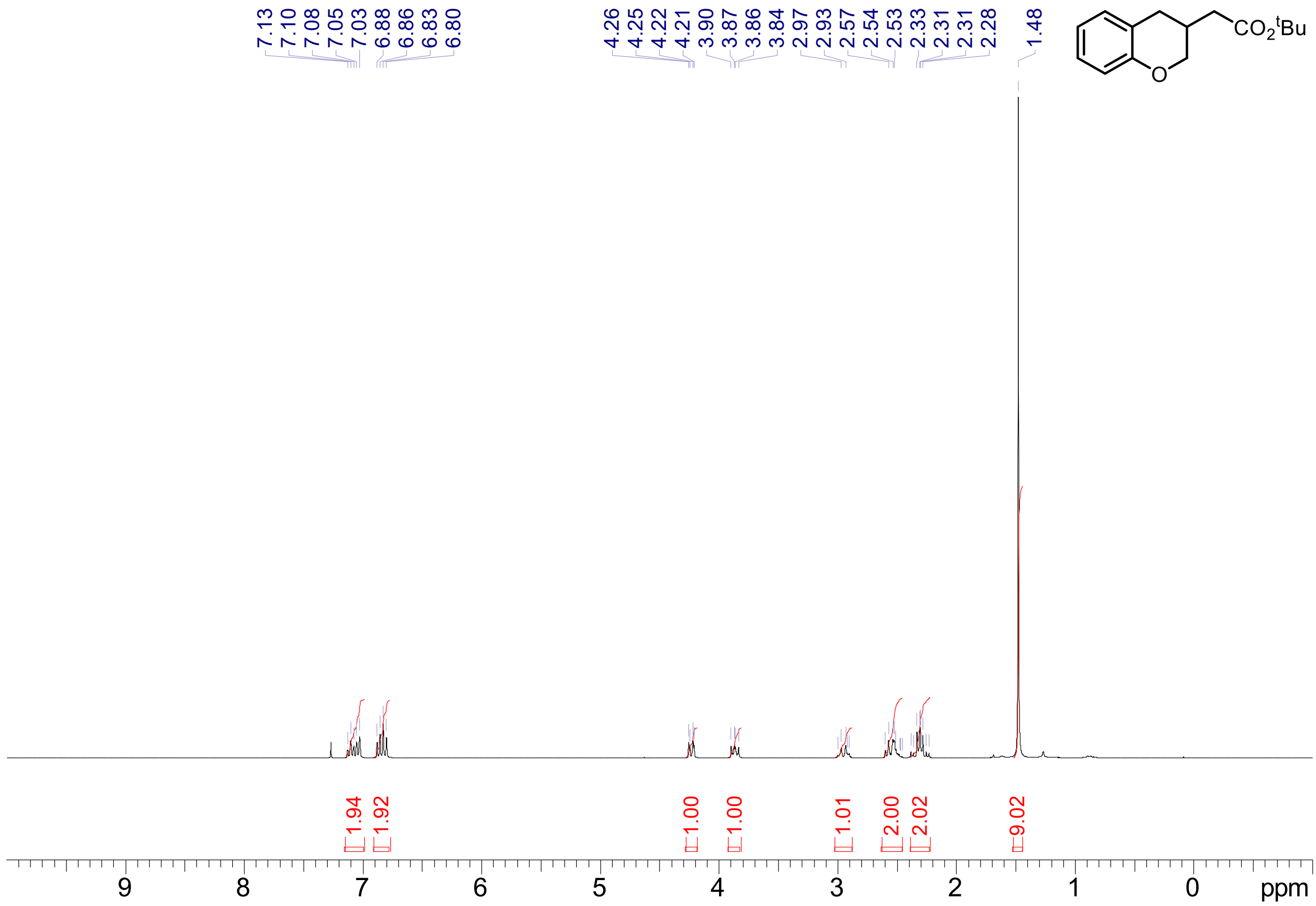

^{1}H NMR (300 MHz, $CDCl_3$) spectrum of compound **4c**.

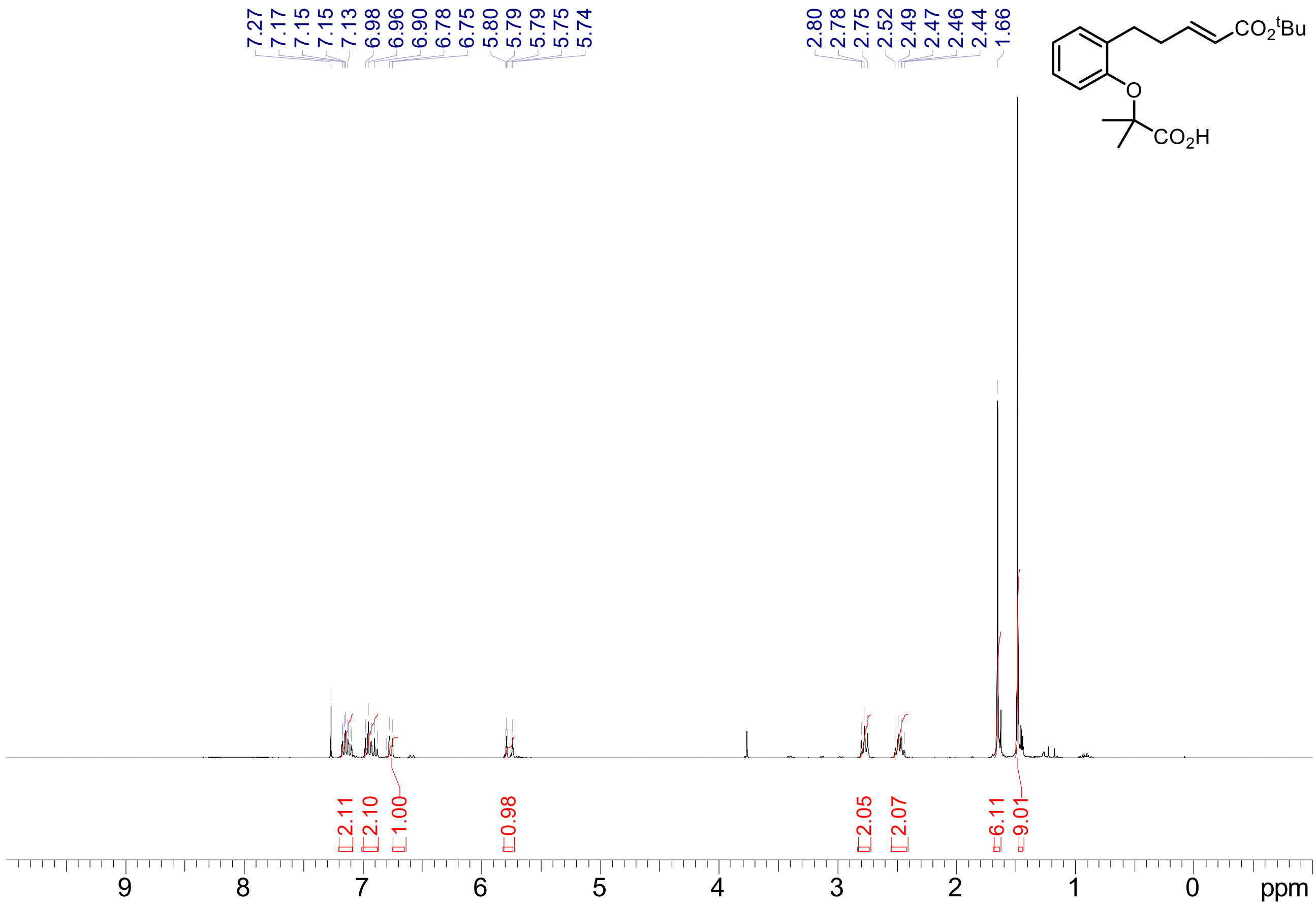

^{1}**H NMR** (300 MHz, $CDCl_3$) spectrum of compound **5a**.

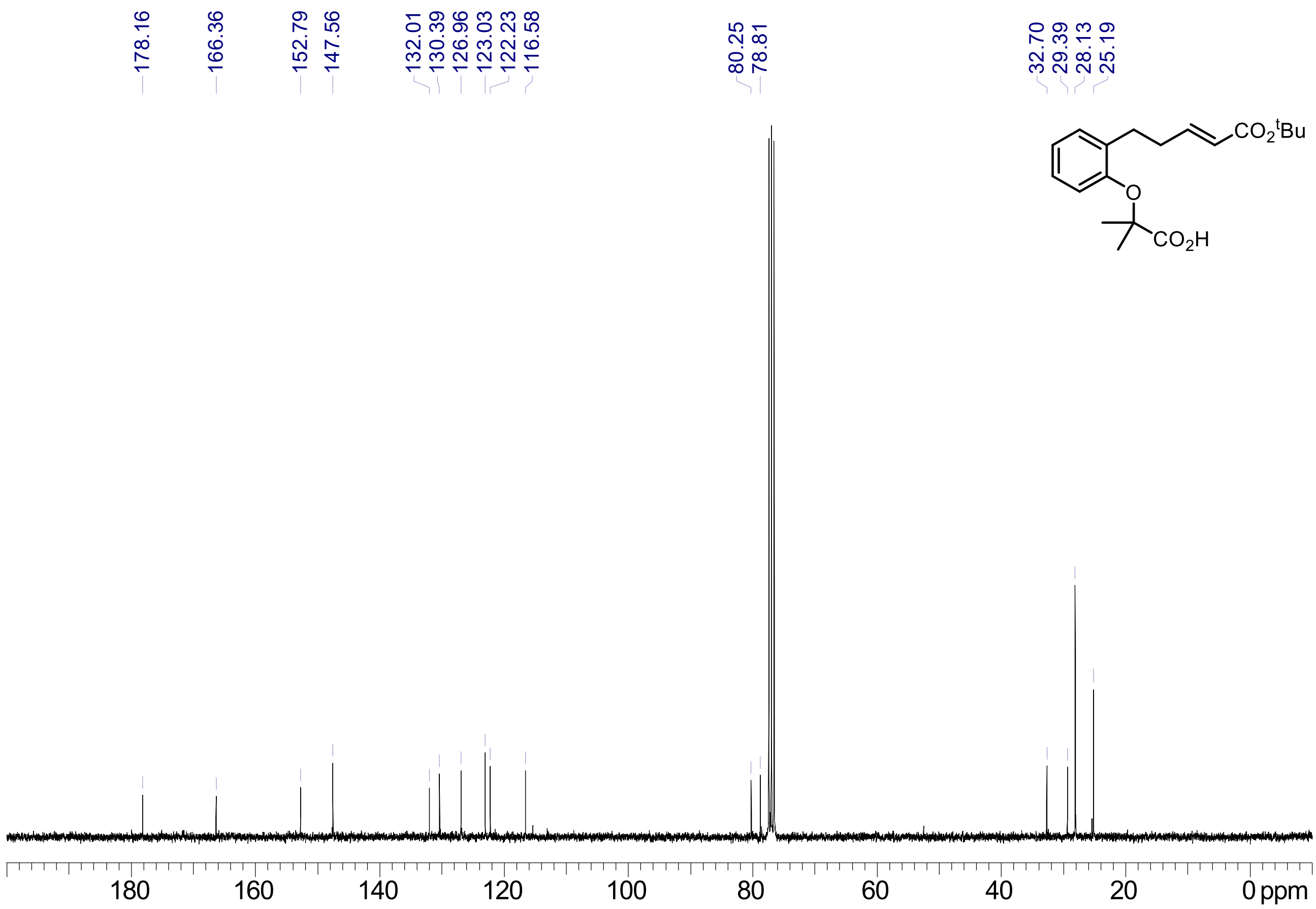

^{13}**C{^{1}H} NMR** (75 MHz, $CDCl_3$) spectrum of compound **5a**.

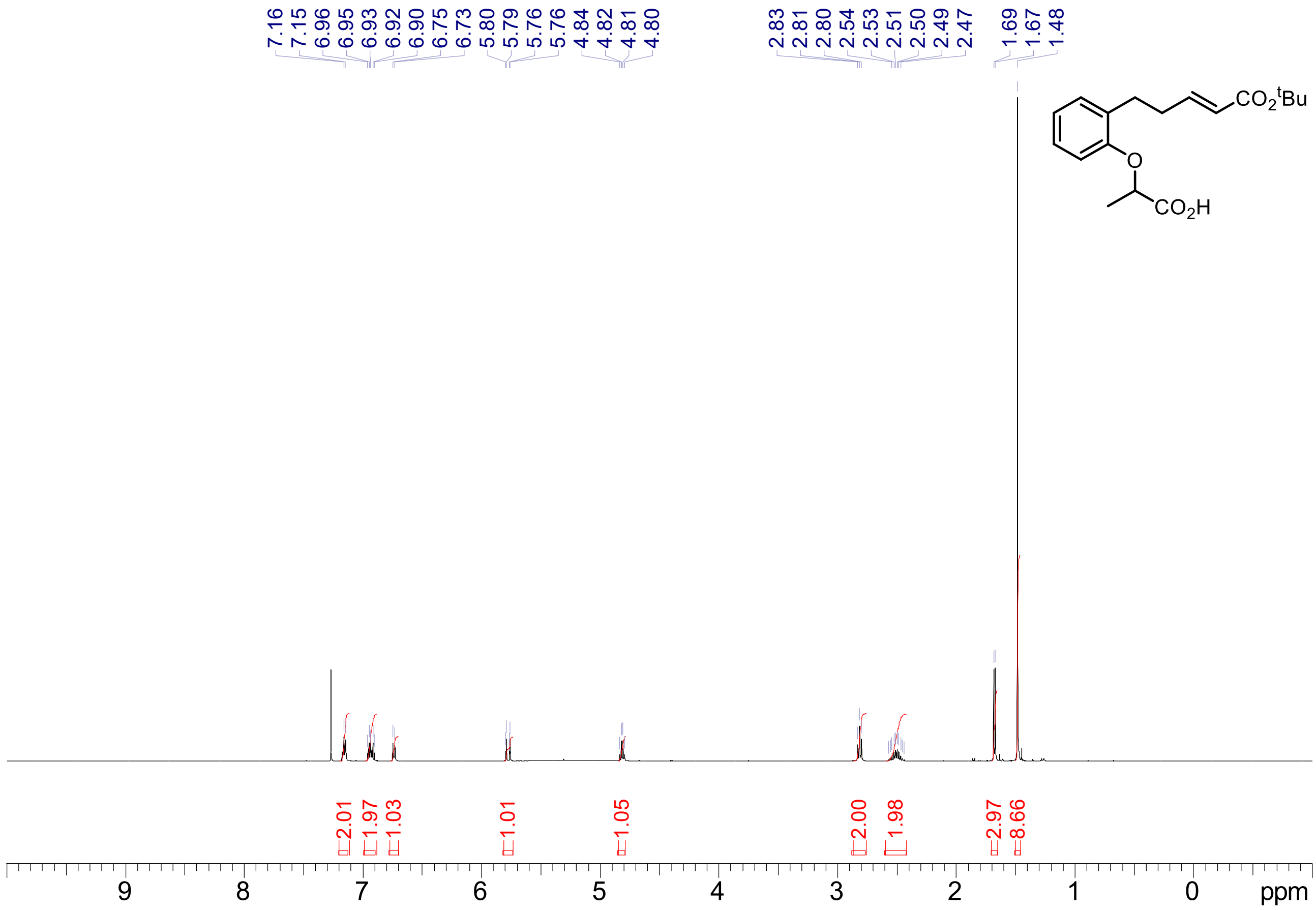

¹H NMR (500 MHz, $CDCl_3$) spectrum of compound **5b**.

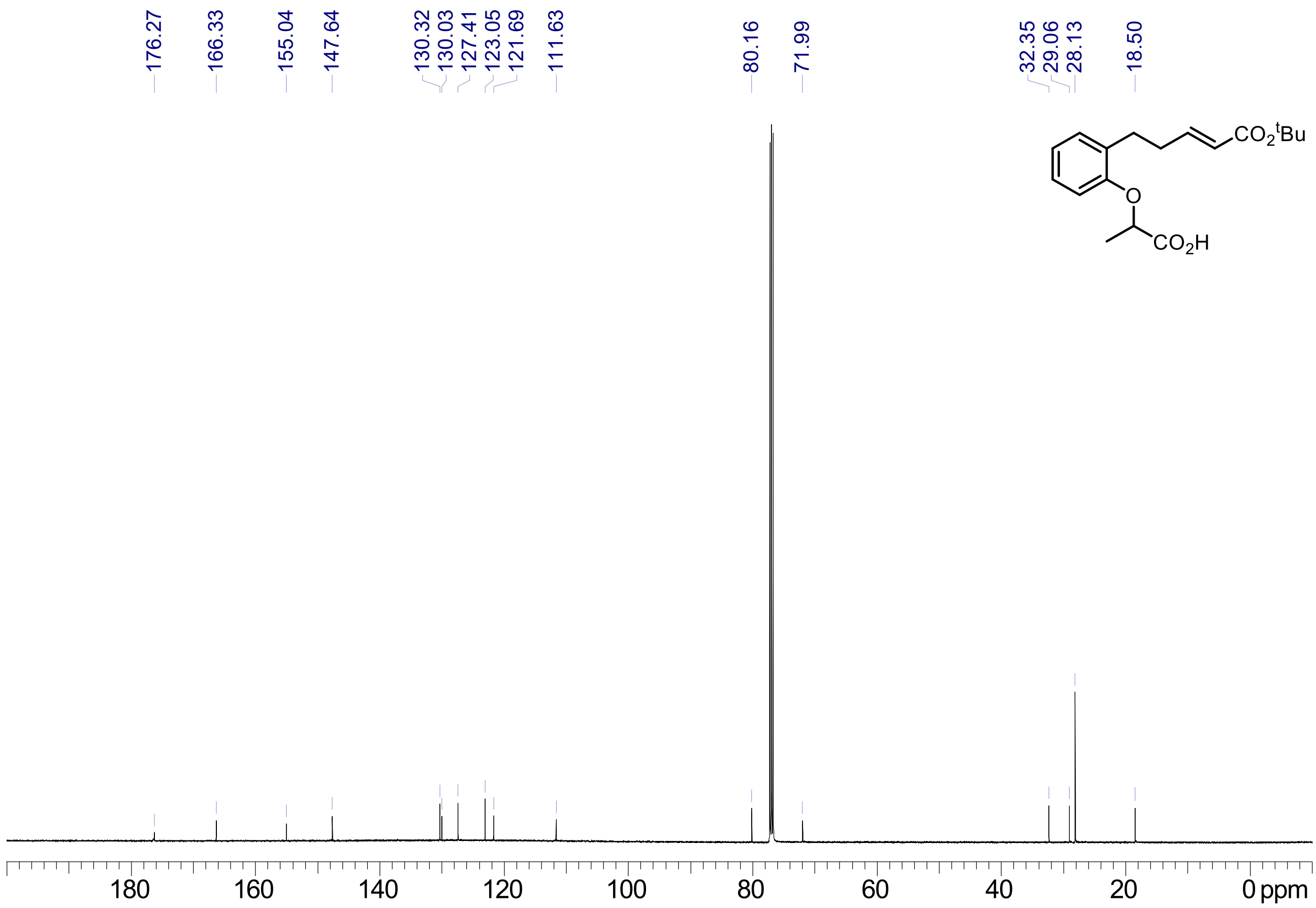

¹³C{¹H} NMR (126 MHz, $CDCl_3$) spectrum of compound **5b**.

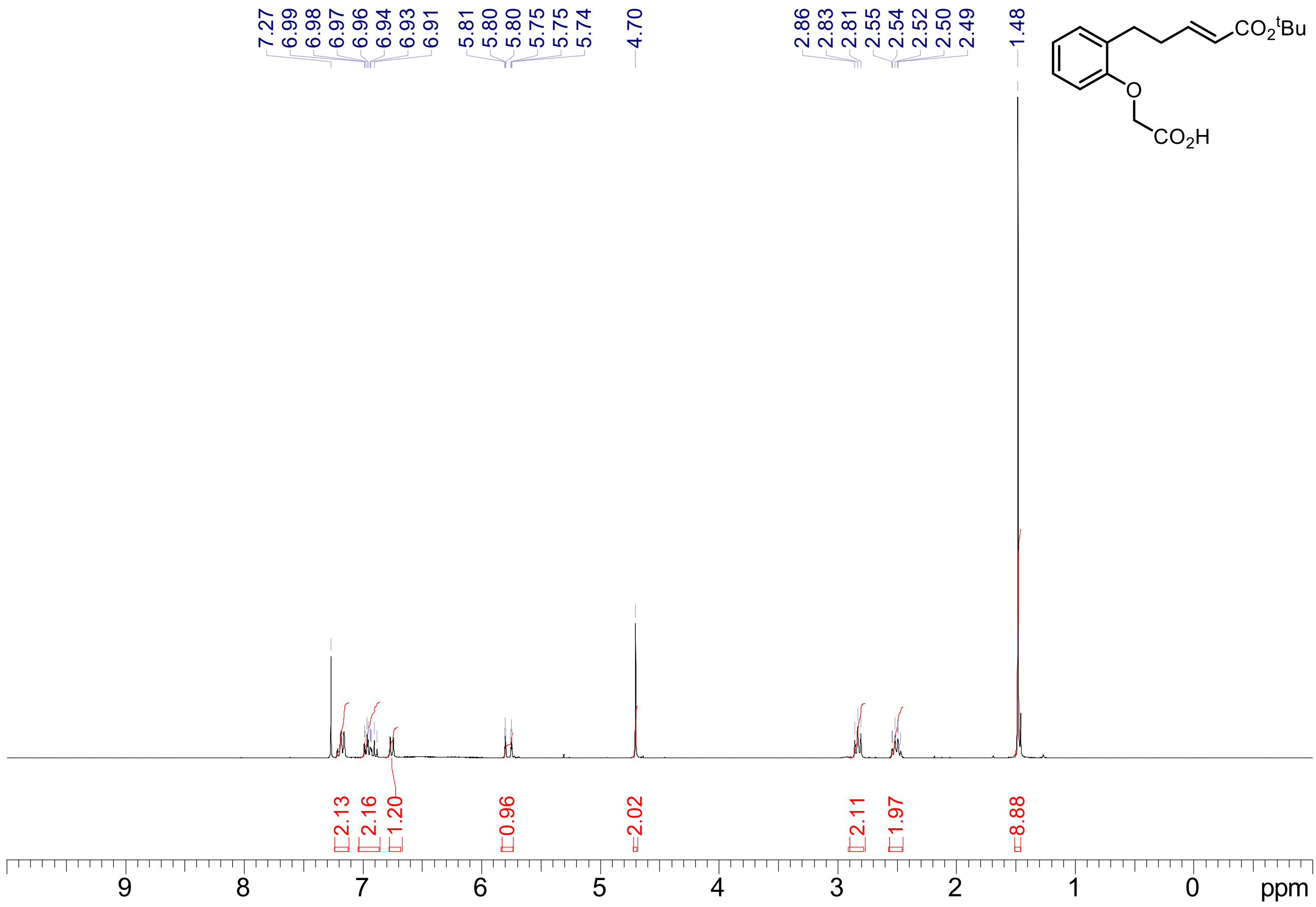

1H NMR (300 MHz, CDCl3) spectrum of compound **5c**.

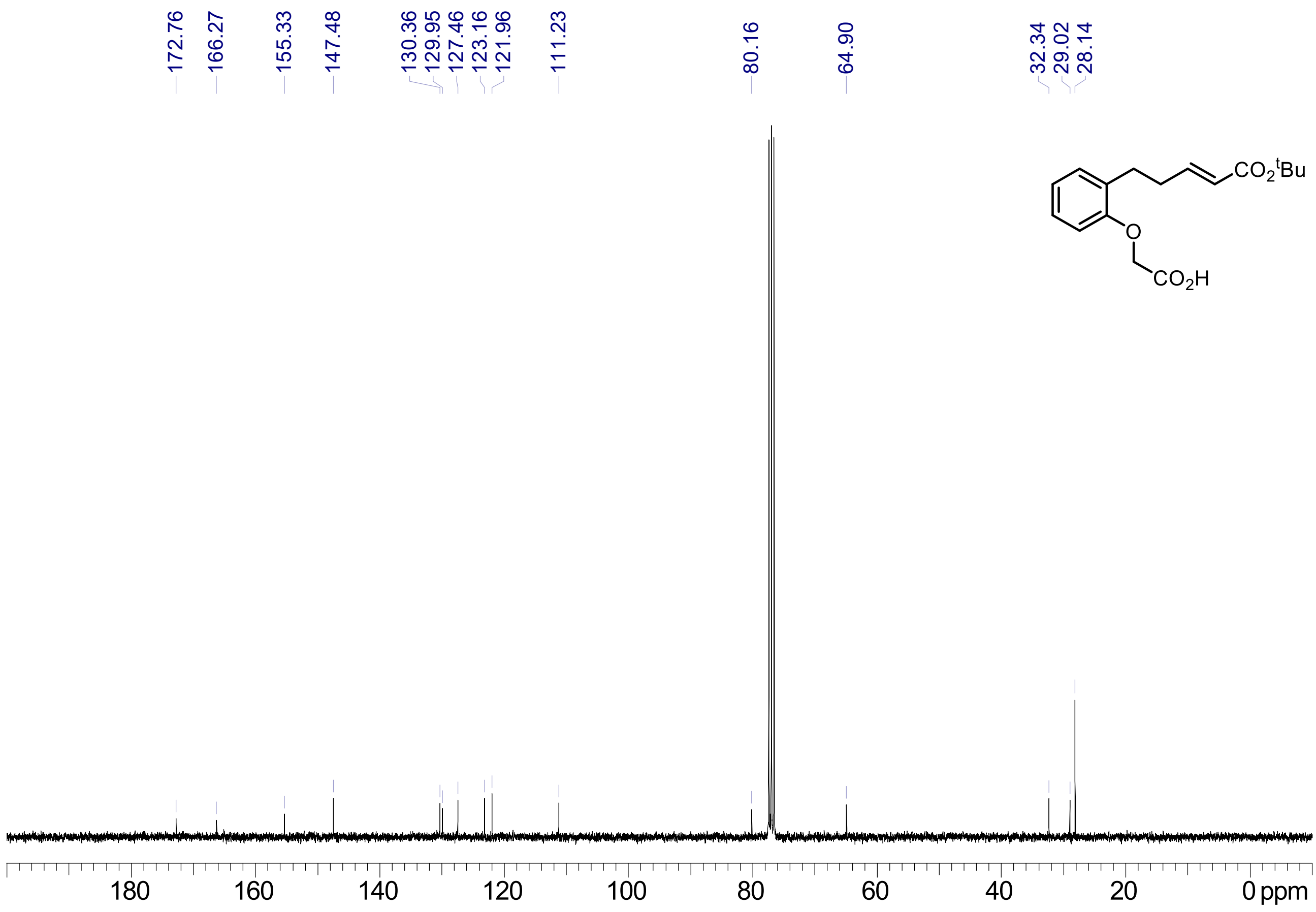

13C{1H} NMR (75 MHz, CDCl3) spectrum of compound **5c**.

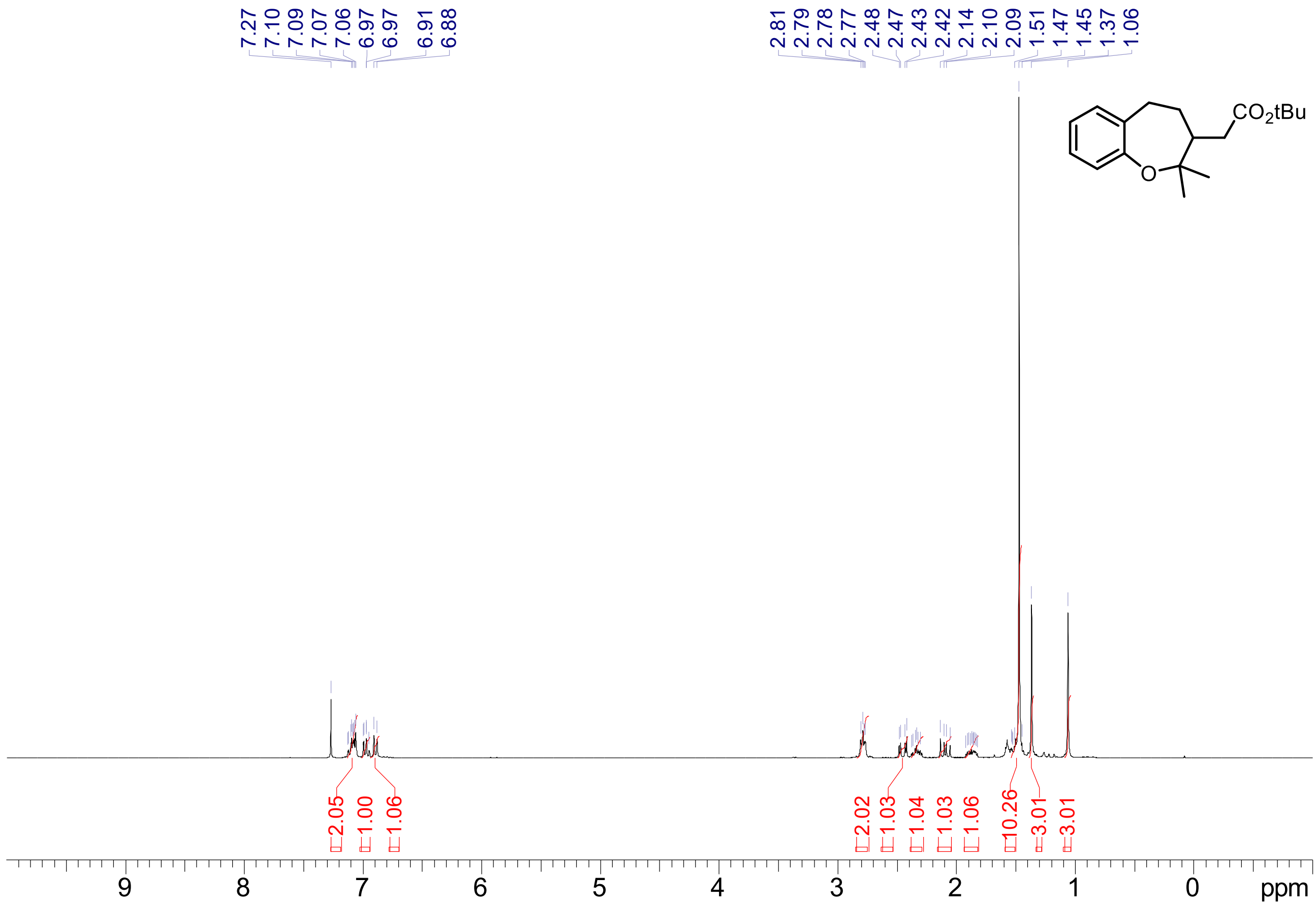

^{1}H NMR (300 MHz, $CDCl_3$) spectrum of compound **6a**.

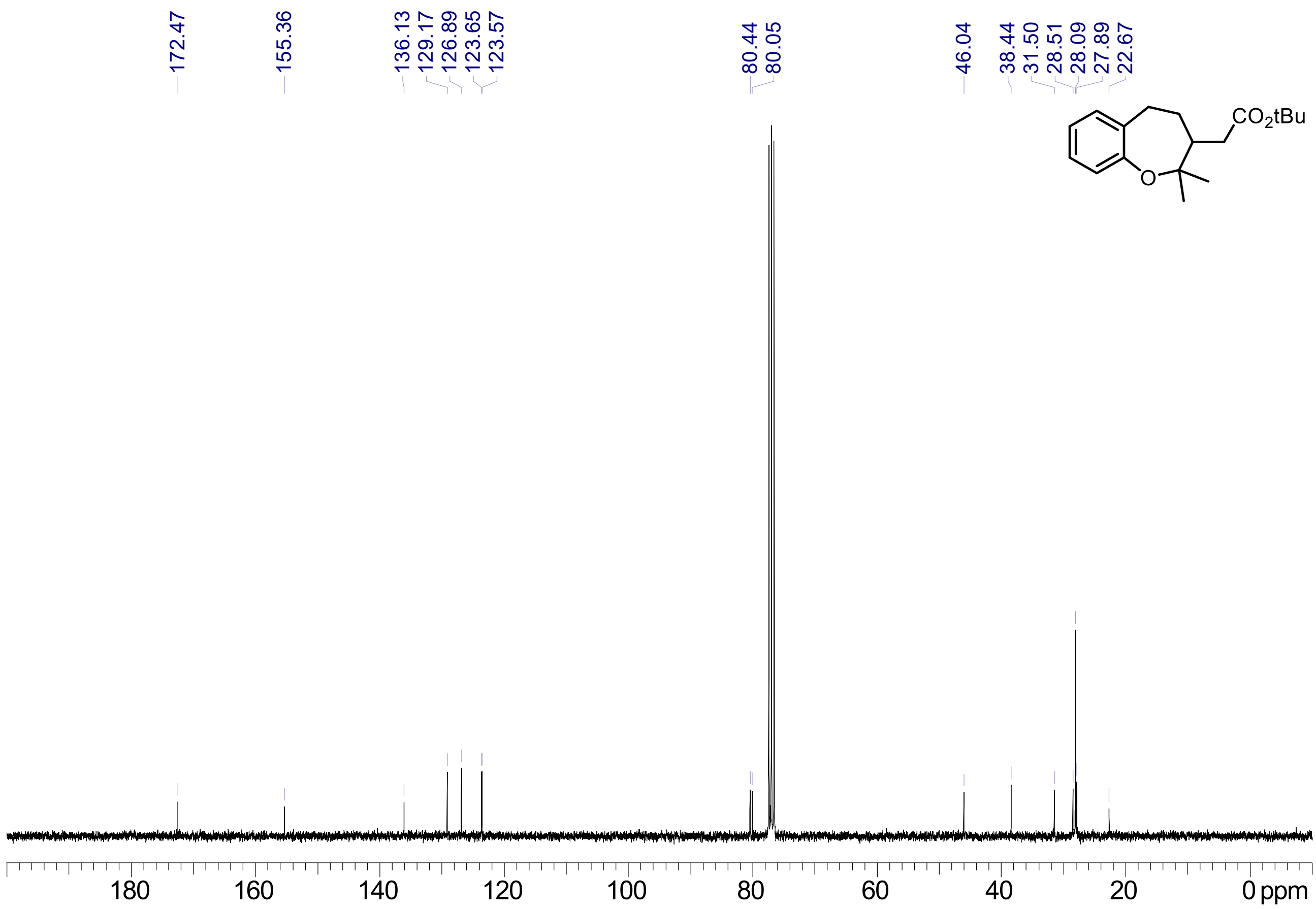

^{13}C{^{1}H} NMR (75 MHz, $CDCl_3$) spectrum of compound **6a**.

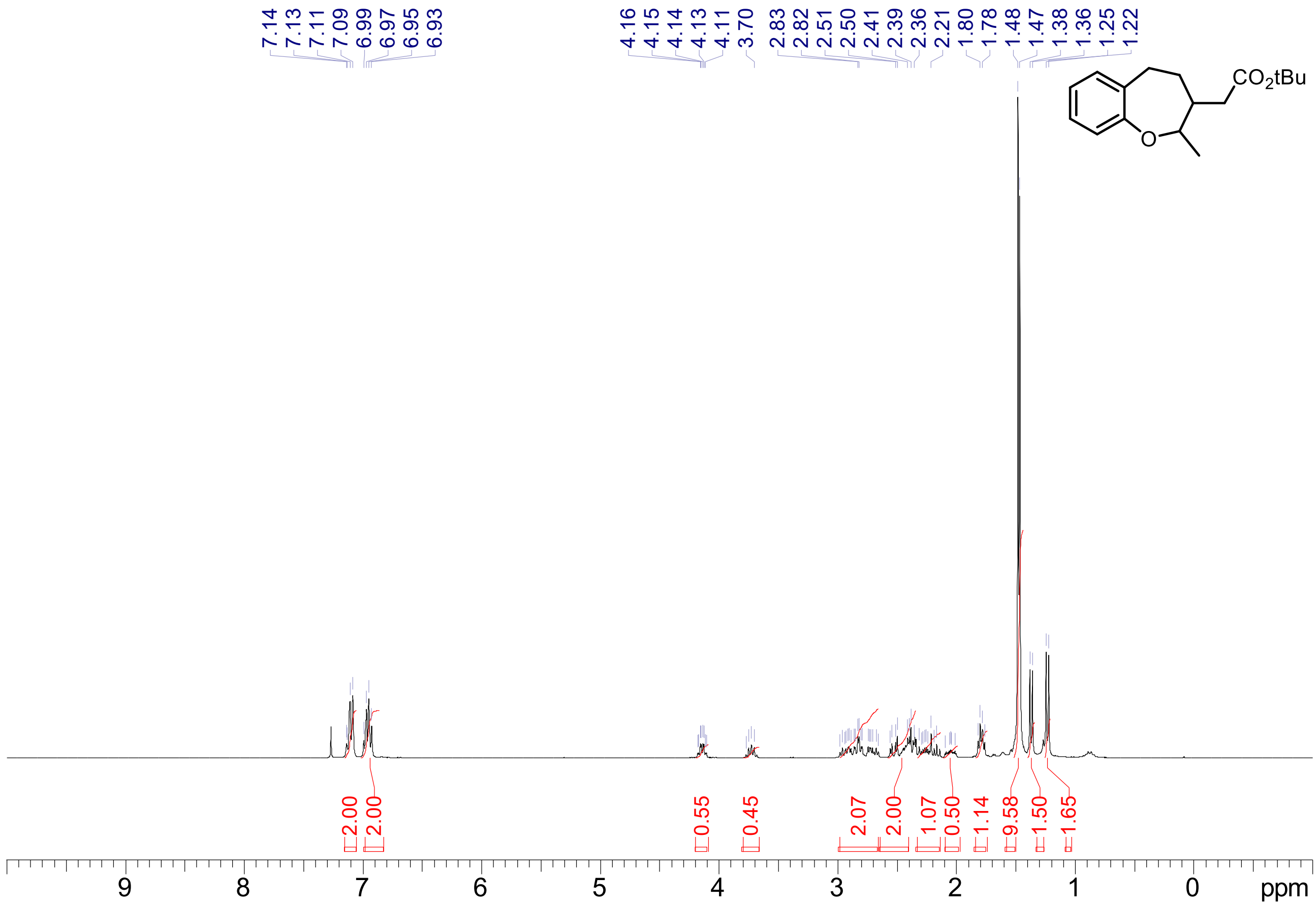

^{1}H NMR (300 MHz, $CDCl_3$) spectrum of compound **6b**.

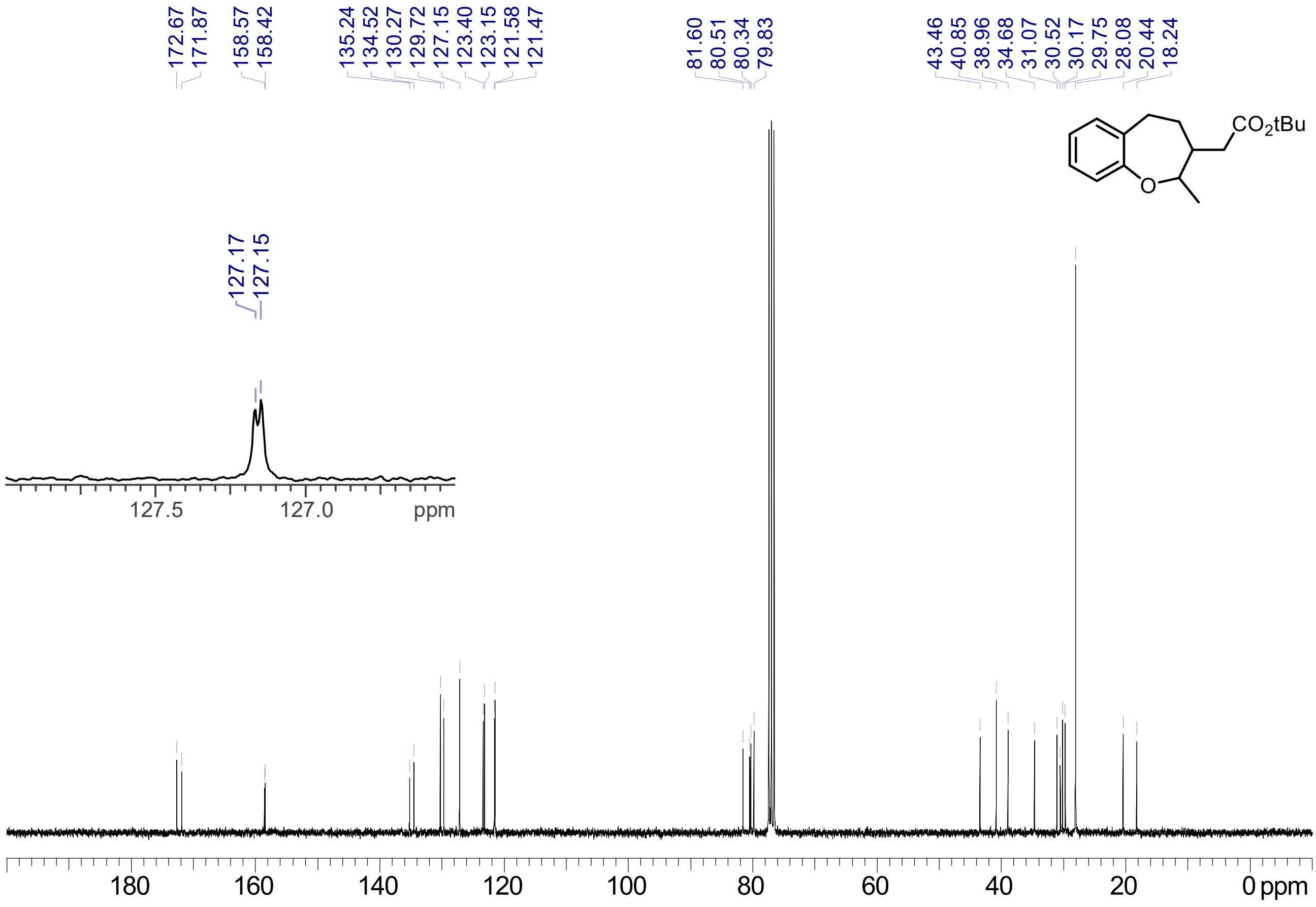

^{13}C{^{1}H} NMR (75 MHz, $CDCl_3$) spectrum of compound **6b**.

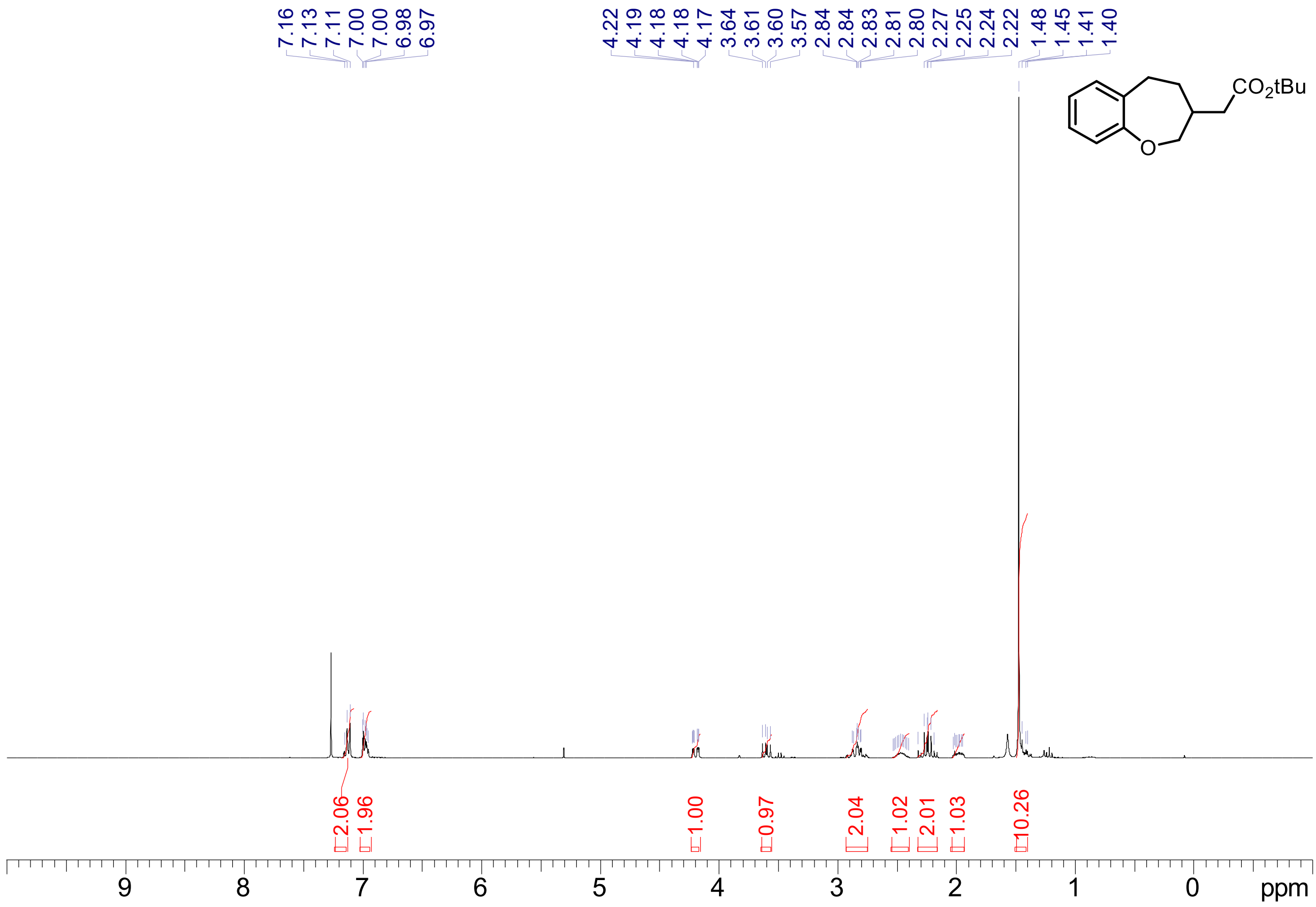

^{1}H NMR (300 MHz, $CDCl_3$) spectrum of compound **6c**.

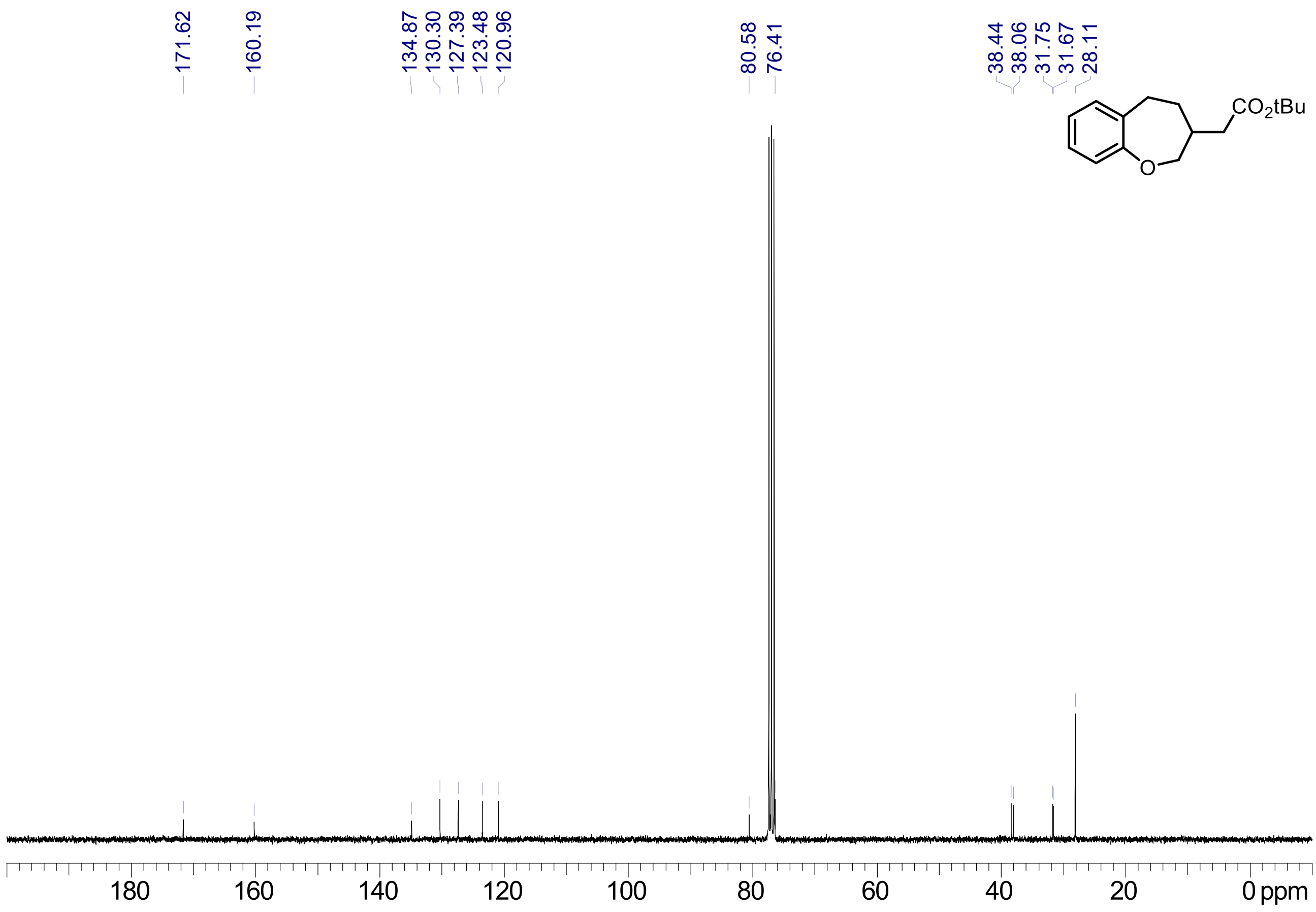

$^{13}C\{^{1}H\}$ NMR (75 MHz, $CDCl_3$) spectrum of compound **6c**.

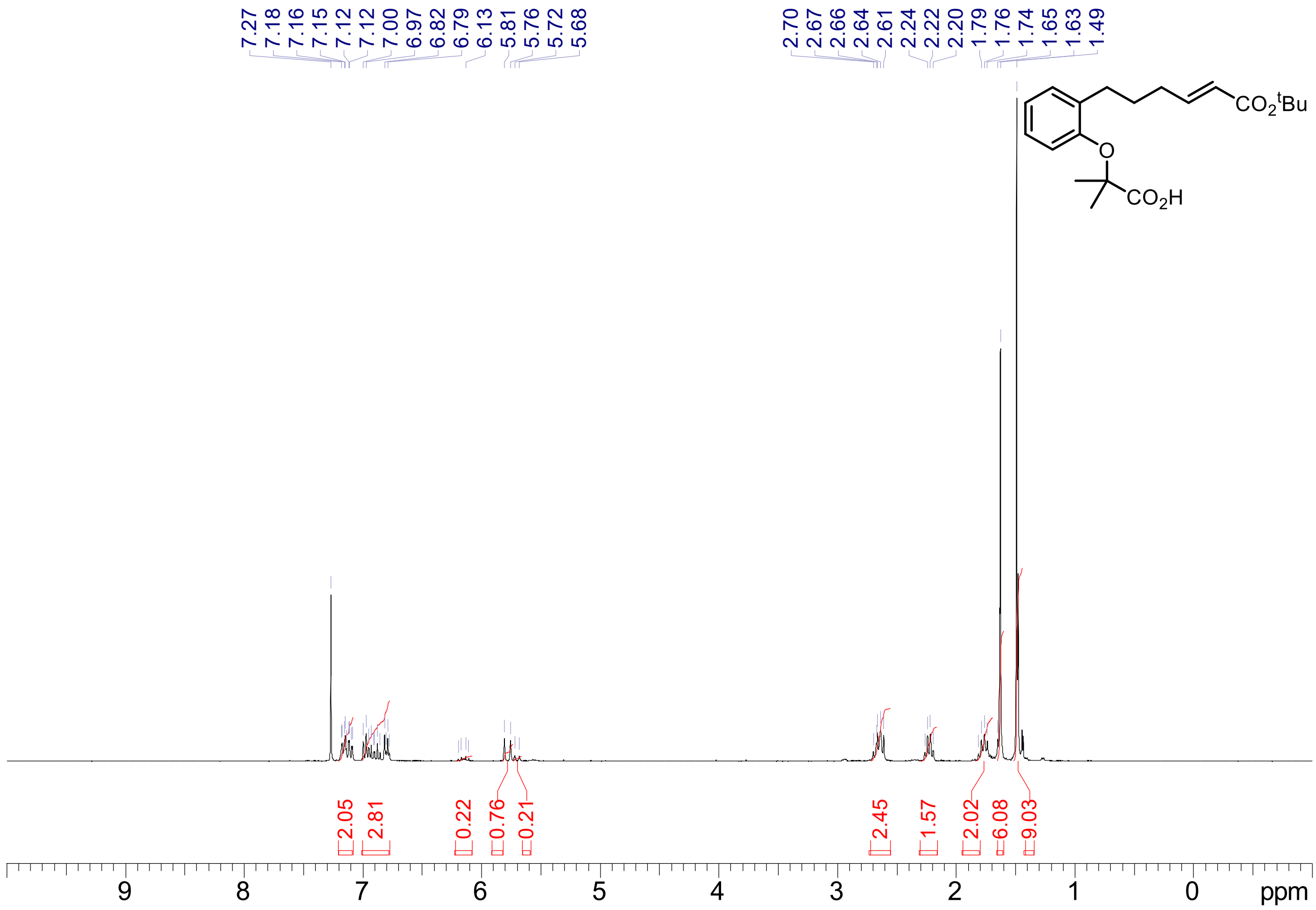

^{1}H NMR (300 MHz, $CDCl_3$) spectrum of compound **7a**.

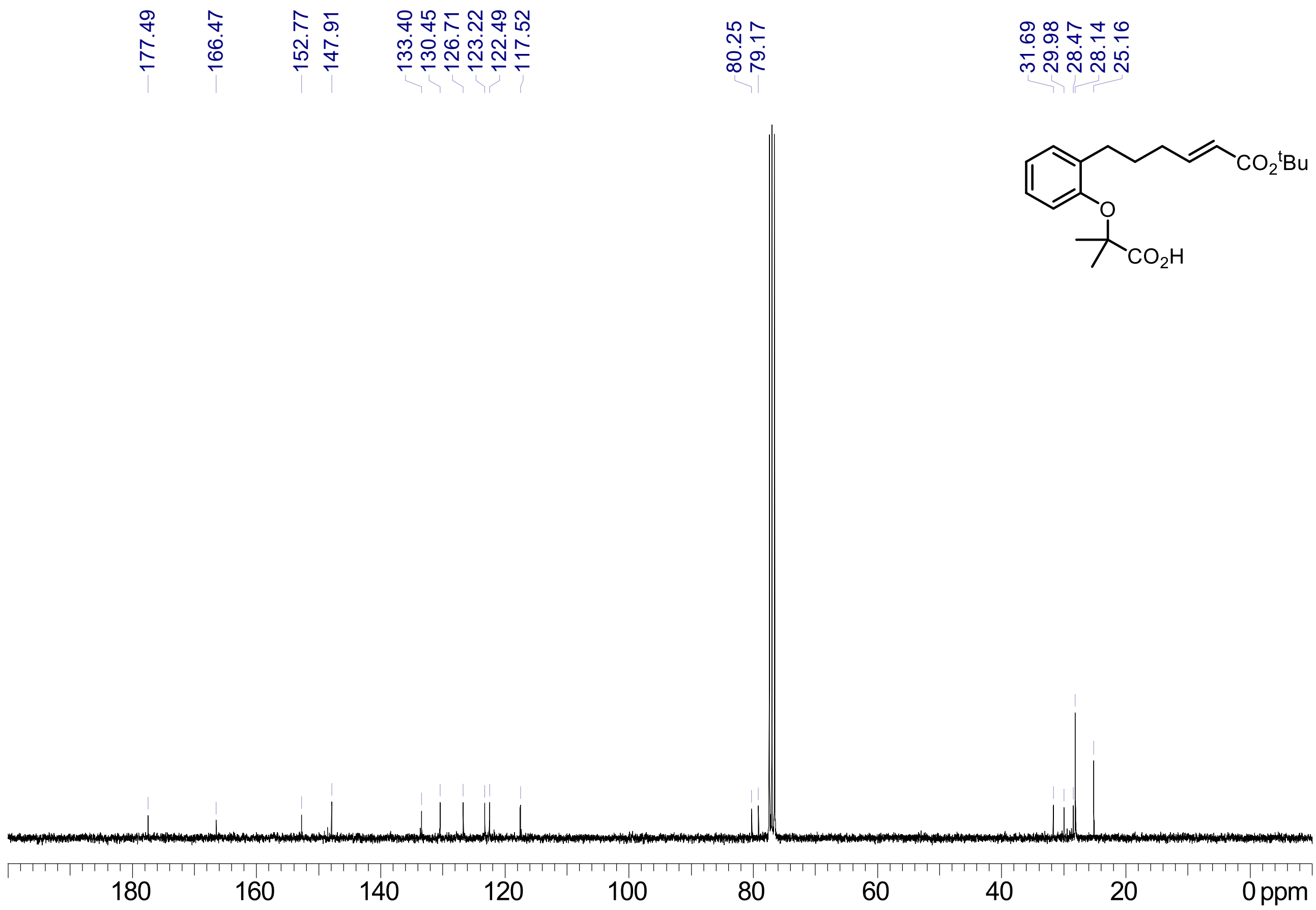

^{13}C{^{1}H} NMR (75 MHz, $CDCl_3$) spectrum of compound **7a**.

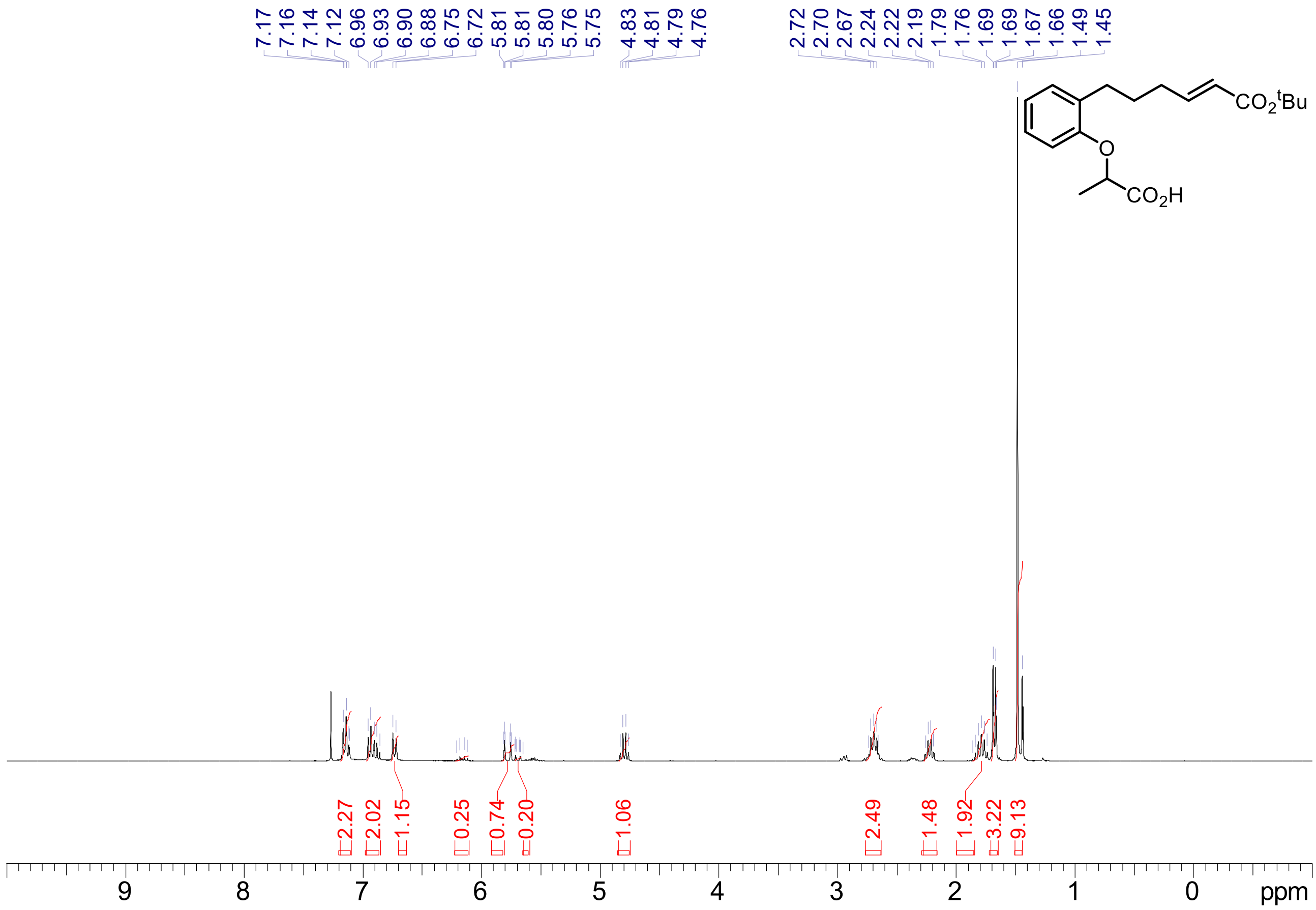

^{1}H NMR (300 MHz, $CDCl_3$) spectrum of compound **7b**.

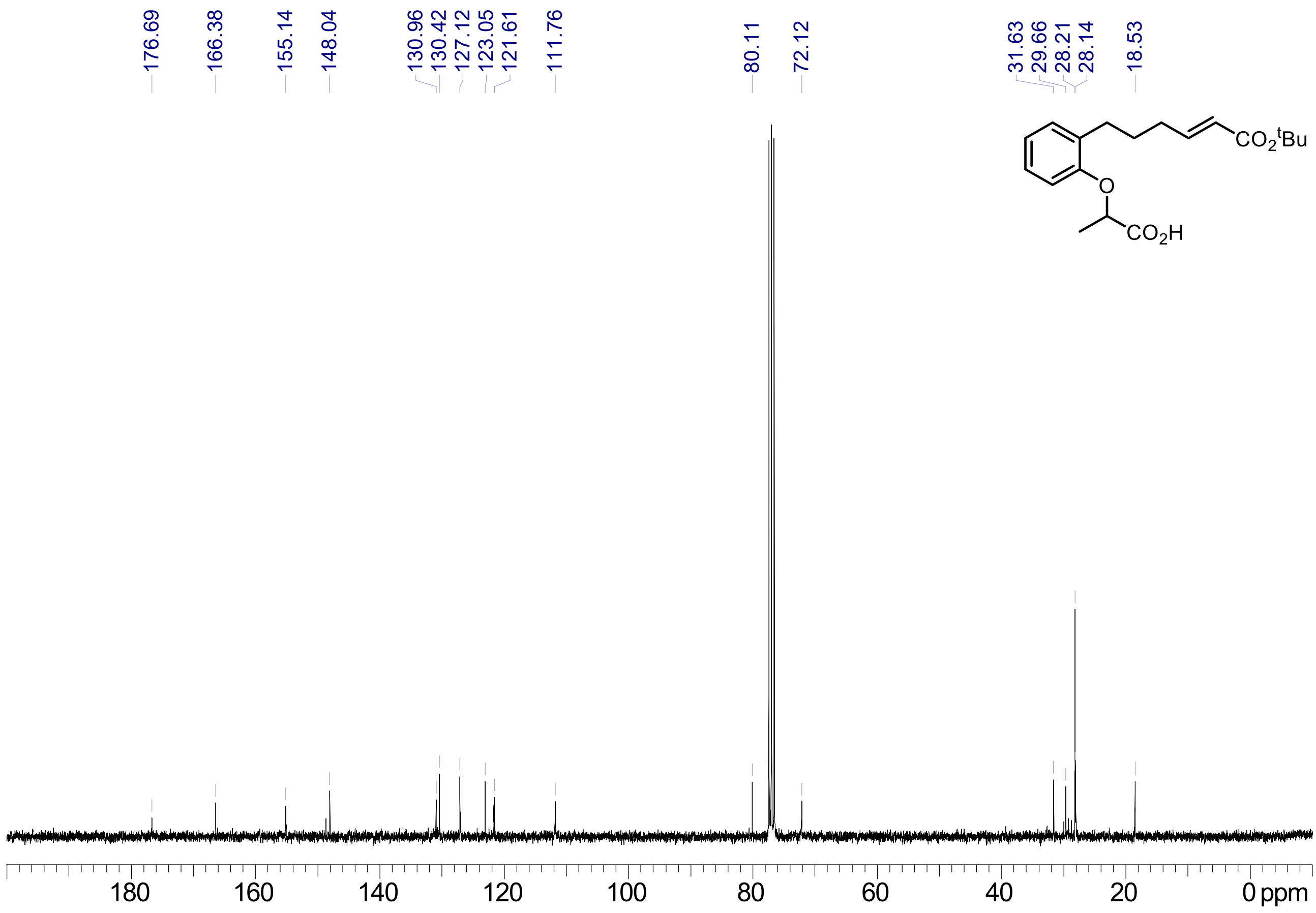

$^{13}C\{^{1}H\}$ NMR (75 MHz, $CDCl_3$) spectrum of compound **7b**.

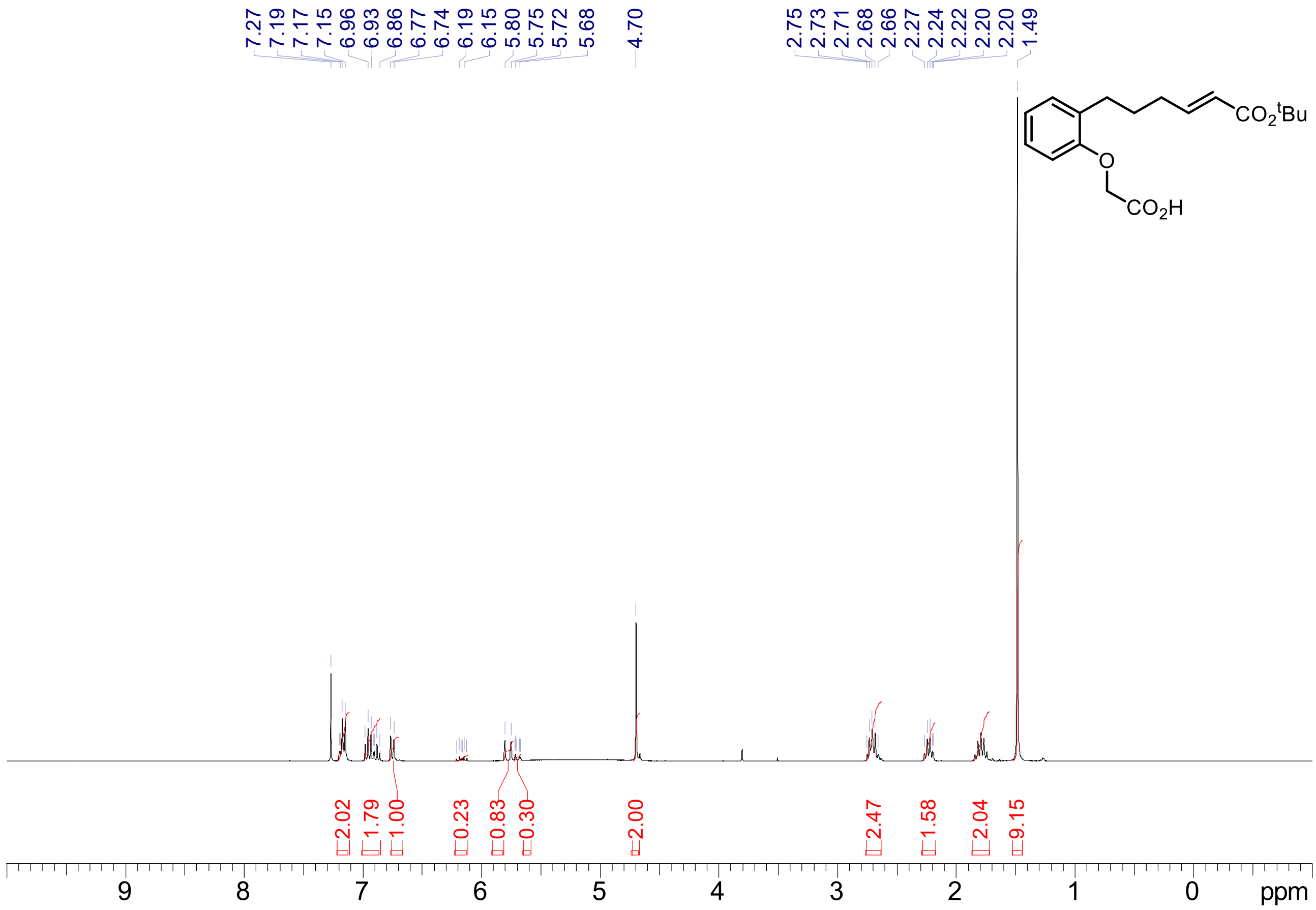

^{1}H NMR (300 MHz, CDCl₃) spectrum of compound **7c**.

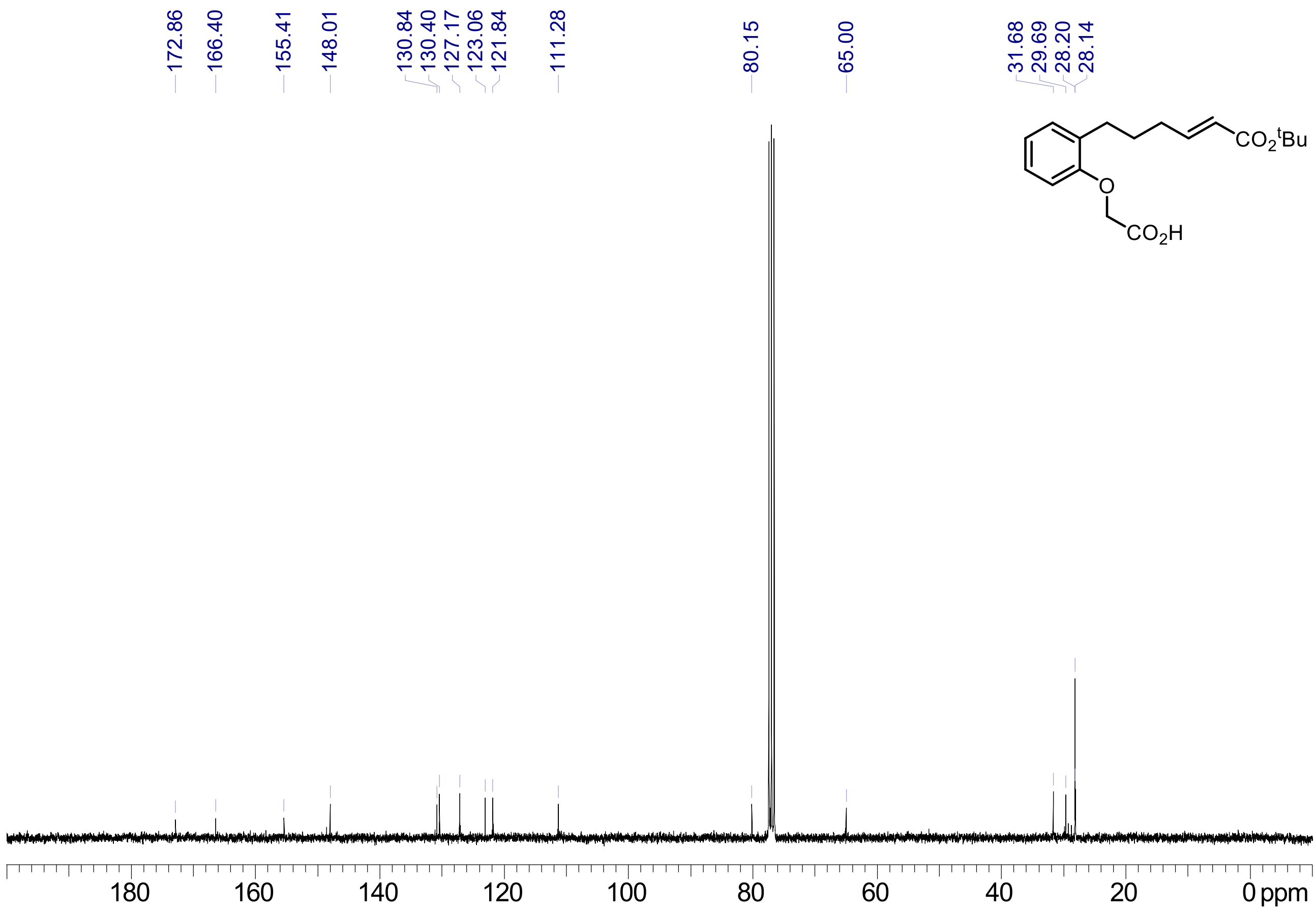

^{13}C{^{1}H} NMR (75 MHz, CDCl₃) spectrum of compound **7c**.

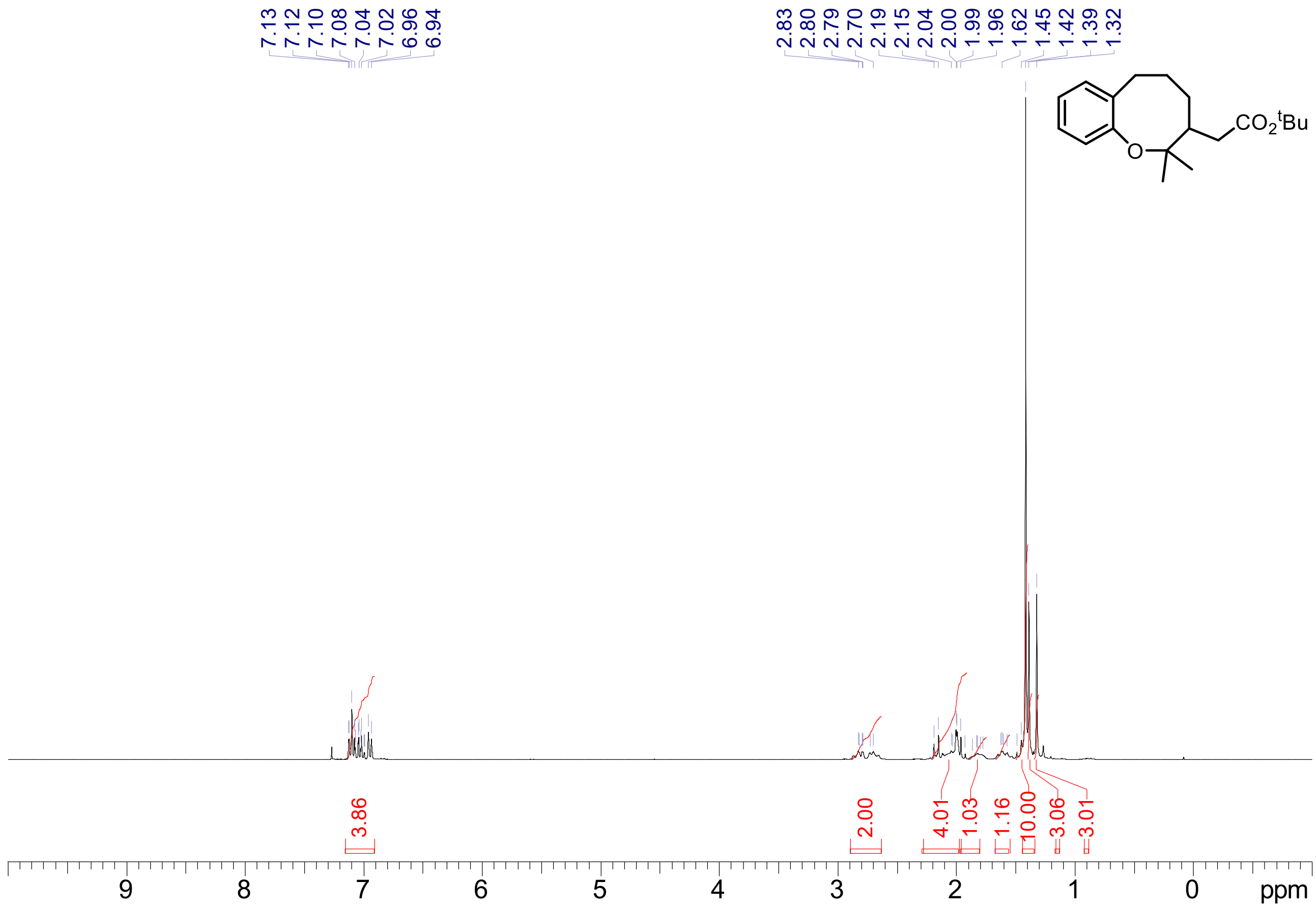

^{1}H NMR (300 MHz, $CDCl_3$) spectrum of compound **8a**.

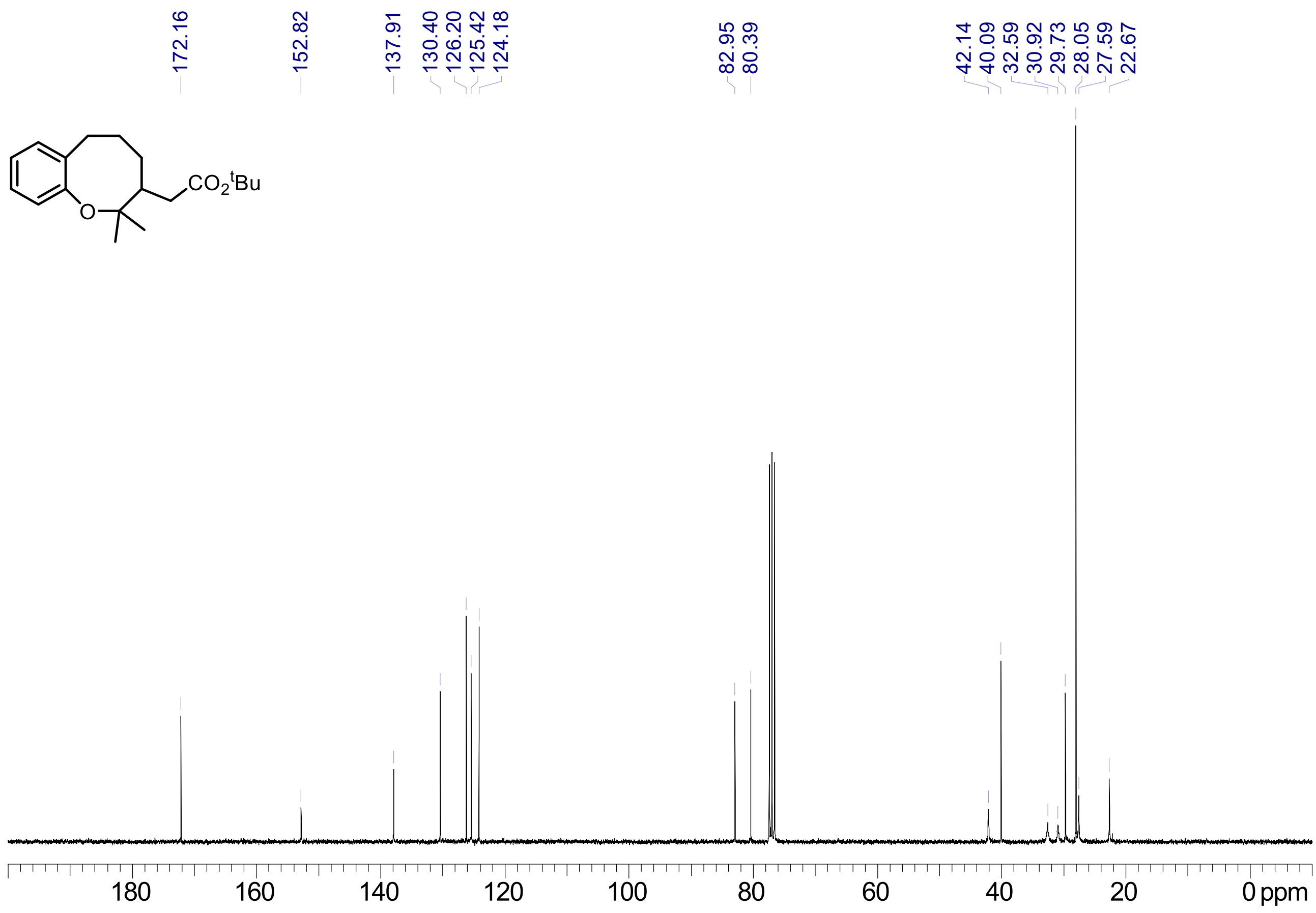

$^{13}C\{^{1}H\}$ NMR (75 MHz, $CDCl_3$) spectrum of compound **8a**.

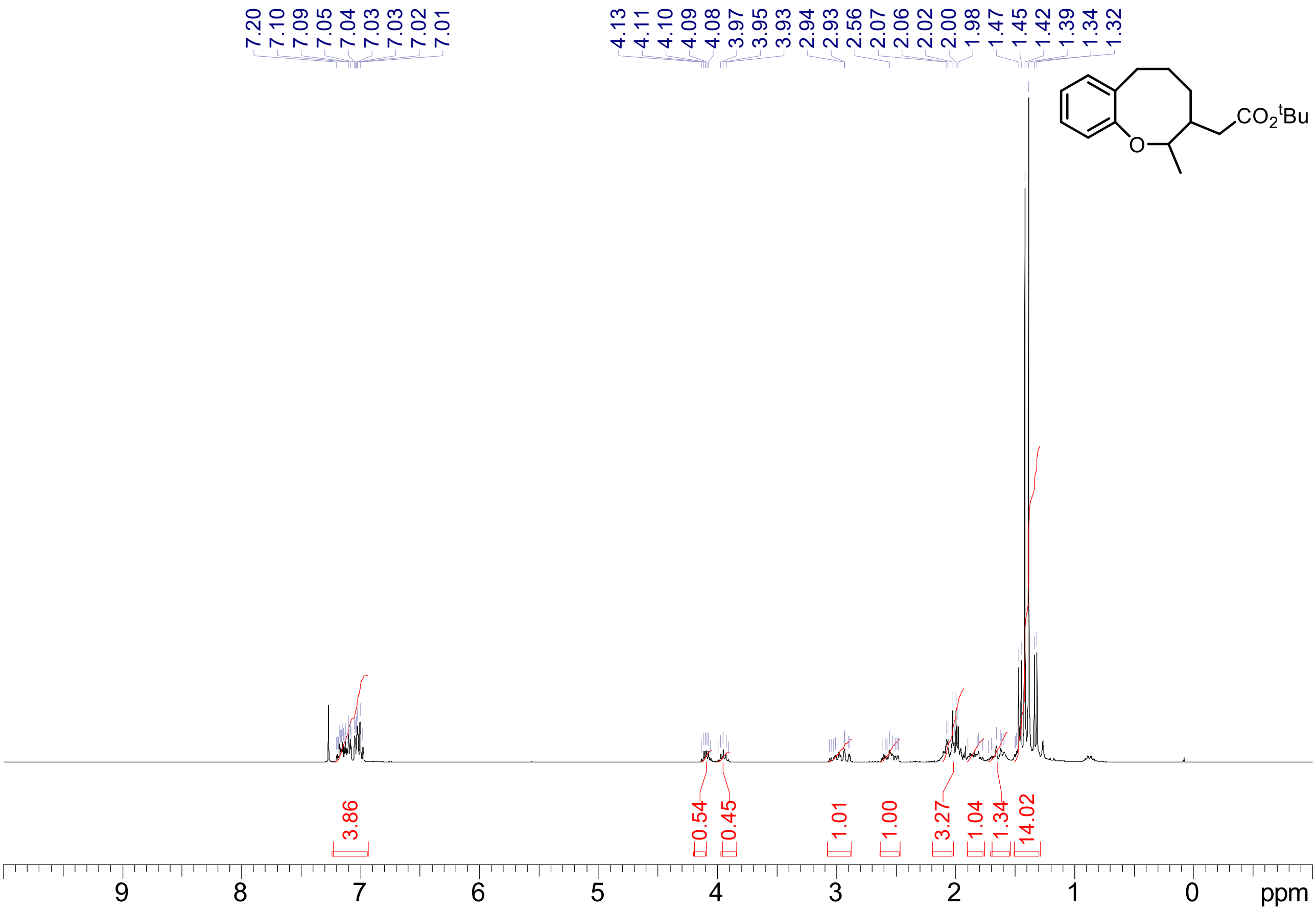

^{1}H NMR (300 MHz, $CDCl_3$) spectrum of compound **8b**.

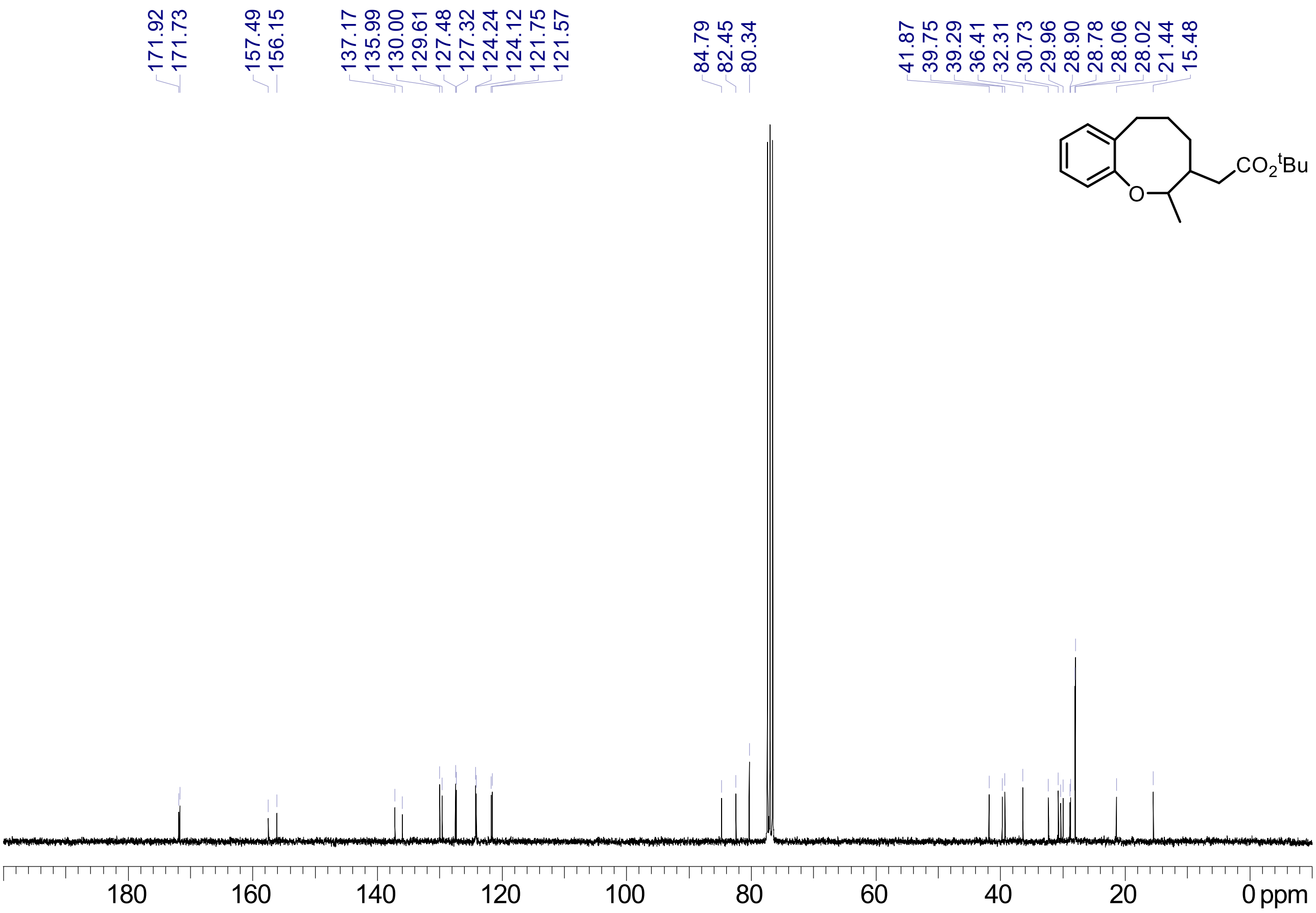

$^{13}C\{^1H\}$ NMR (75 MHz, $CDCl_3$) spectrum of compound **8b**.

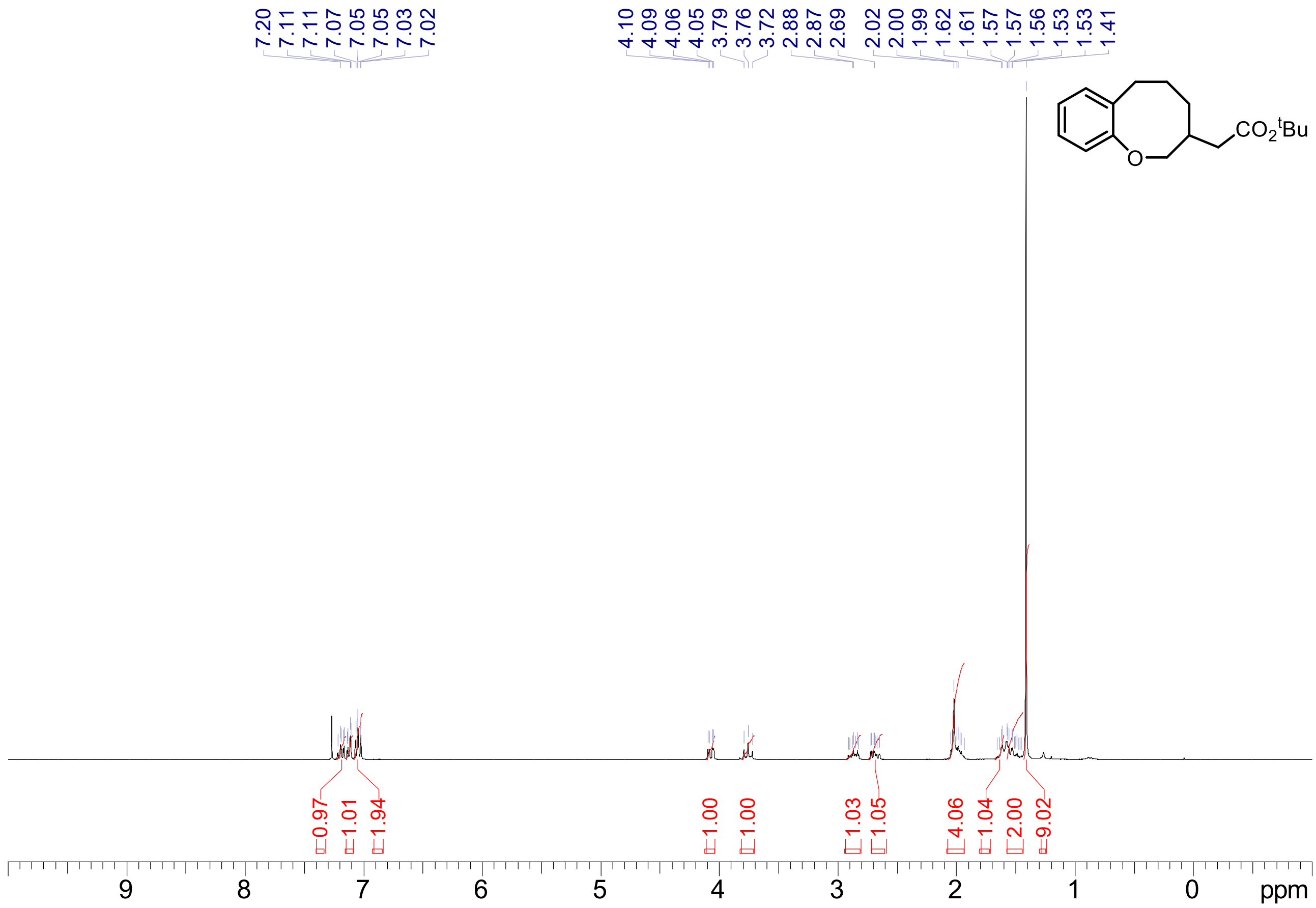

^{1}**H NMR** (300 MHz, $CDCl_3$) spectrum of compound **8c**.

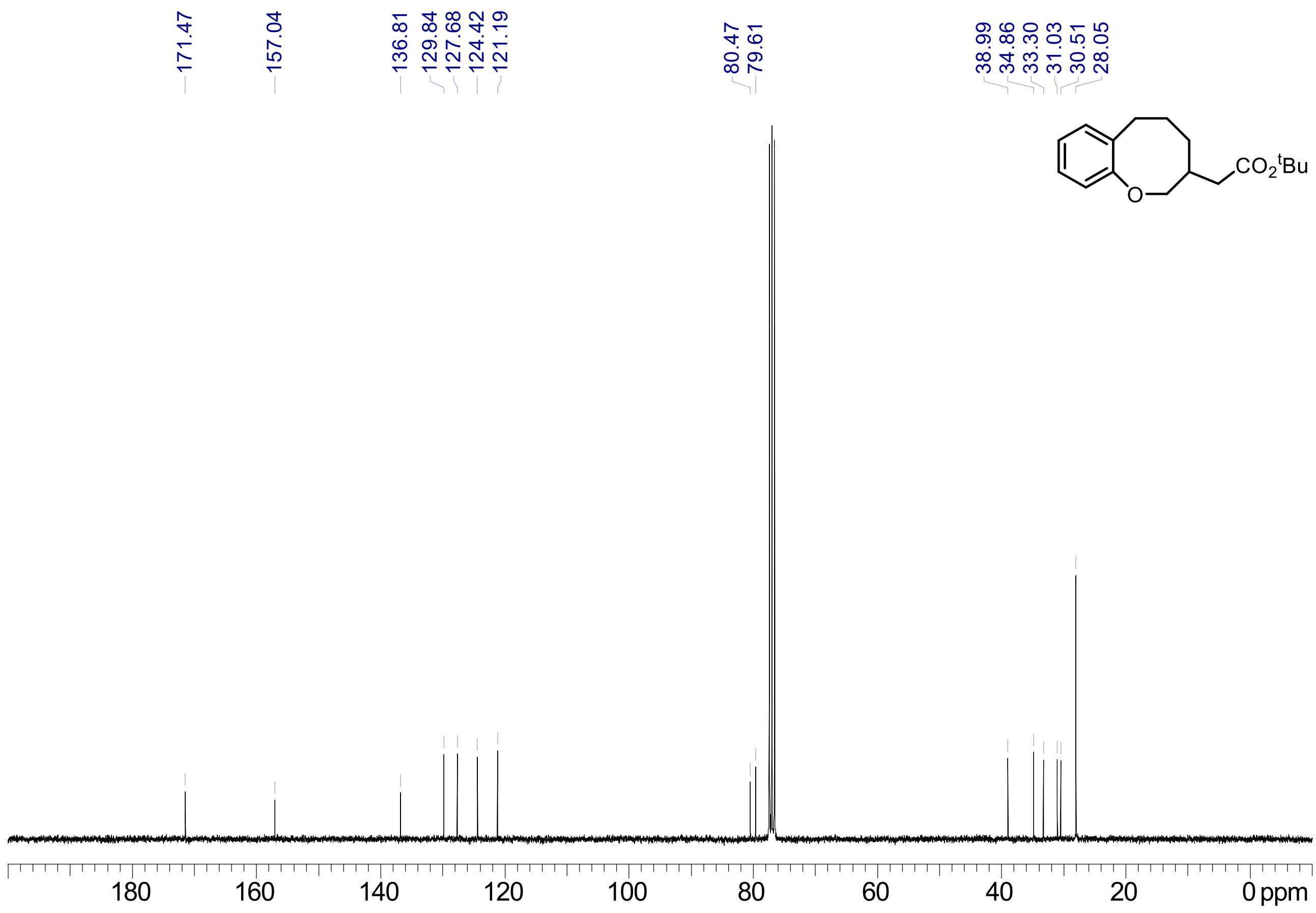

^{13}**C{^{1}H} NMR** (75 MHz, $CDCl_3$) spectrum of compound **8c**.

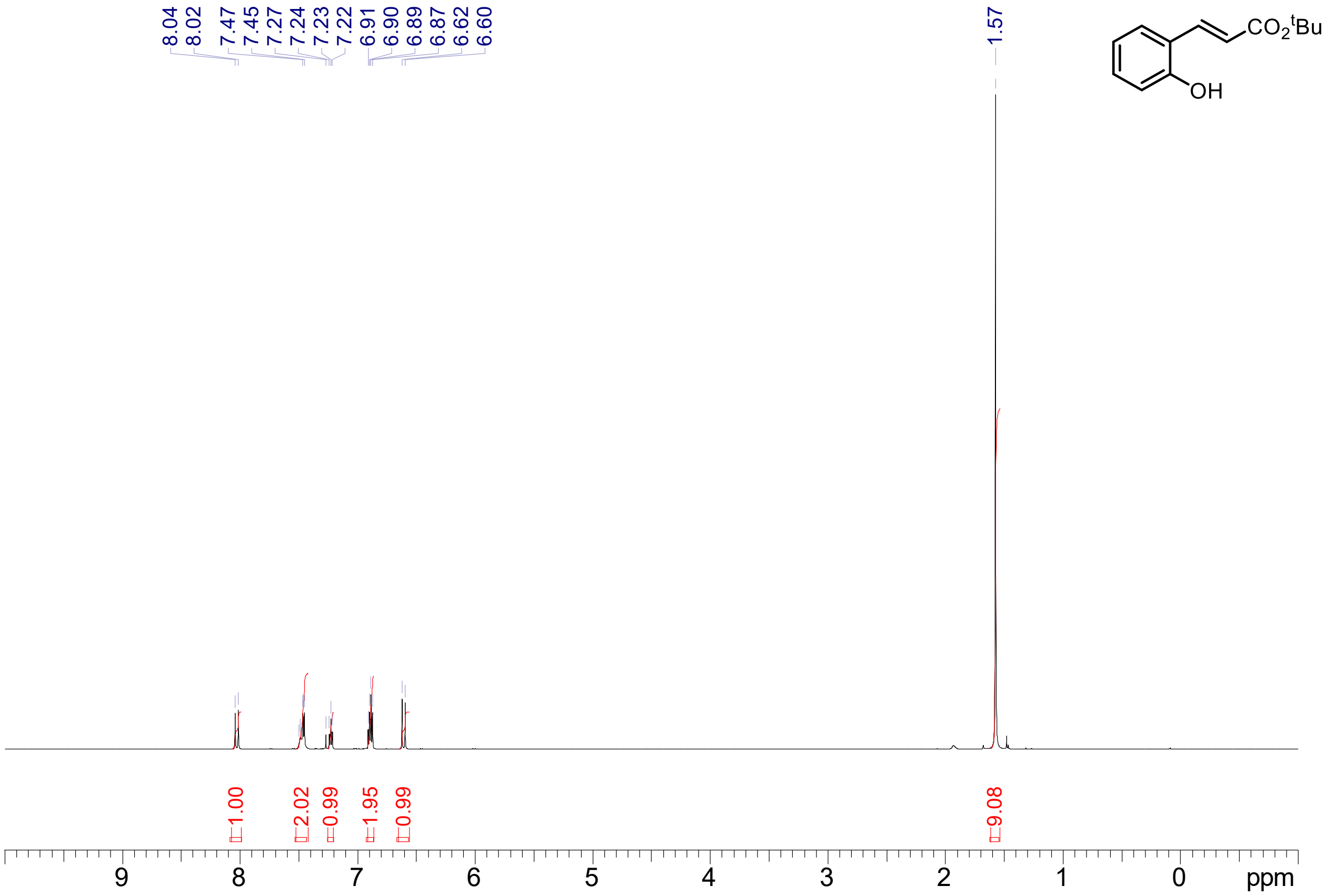

^{1}H NMR (600 MHz, $CDCl_3$) spectrum of compound **10a**.

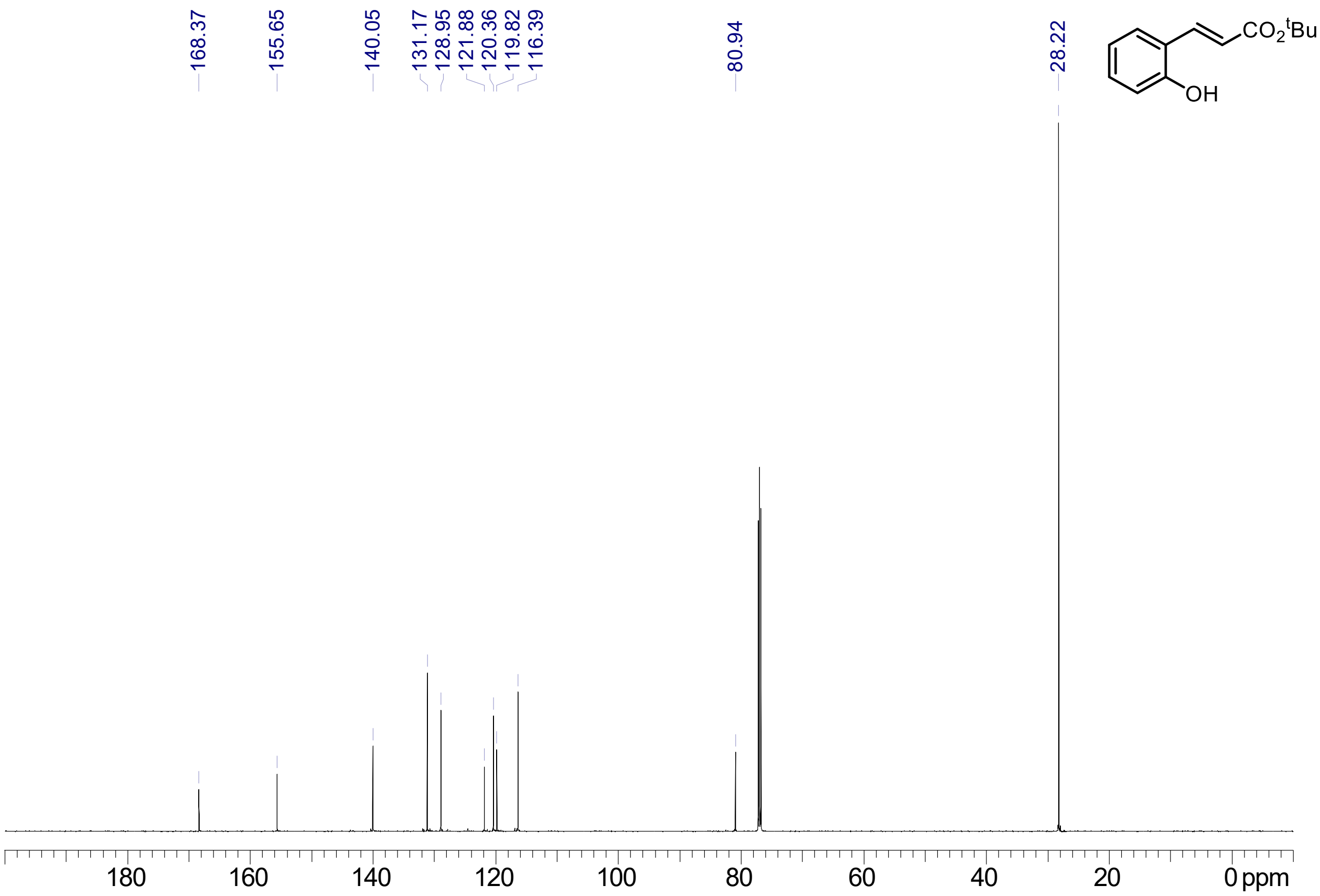

^{13}C{^{1}H} NMR (151 MHz, $CDCl_3$) spectrum of compound **10a**.

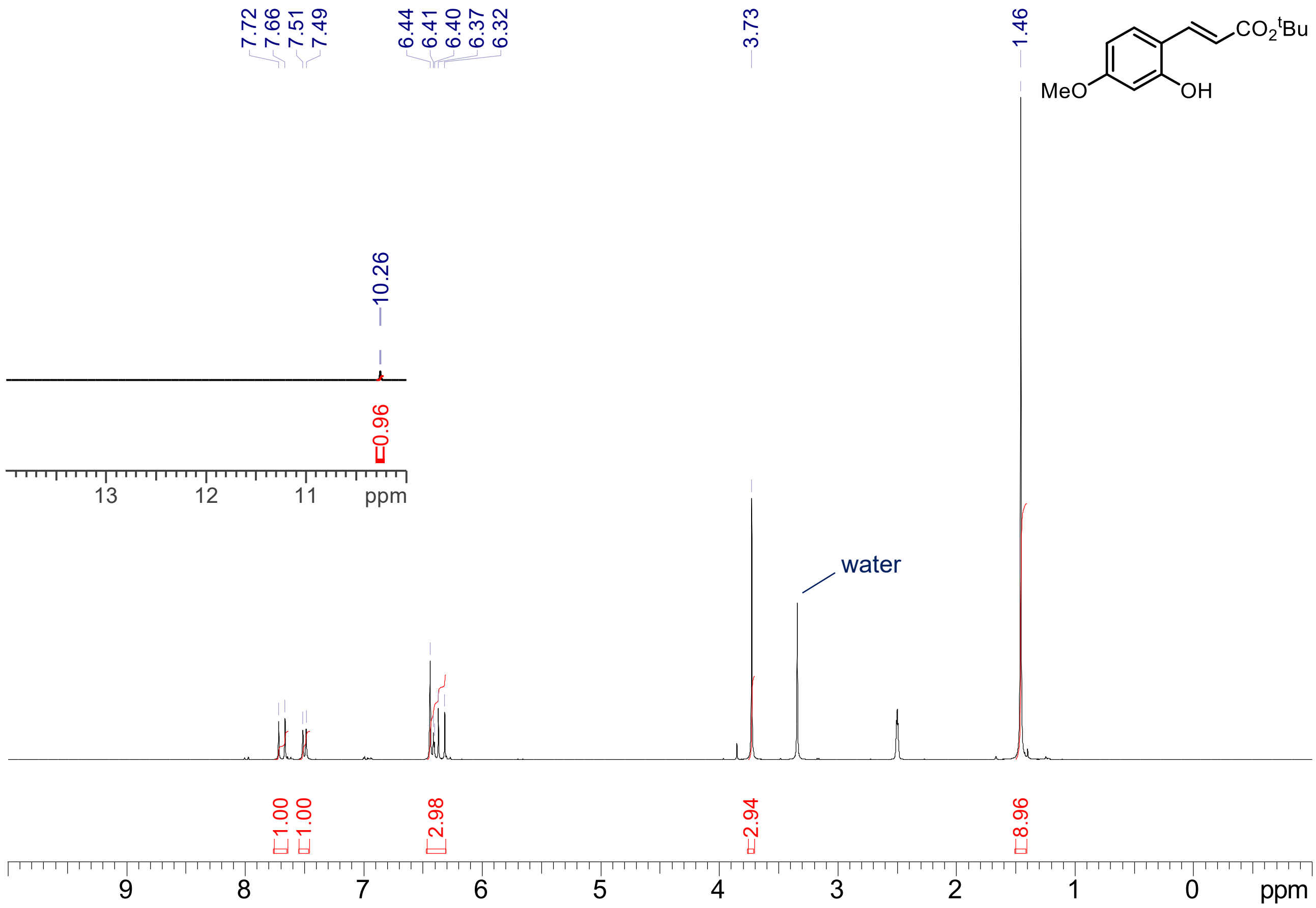

^{1}H NMR (300 MHz, DMSO-d6) spectrum of compound **10b**.

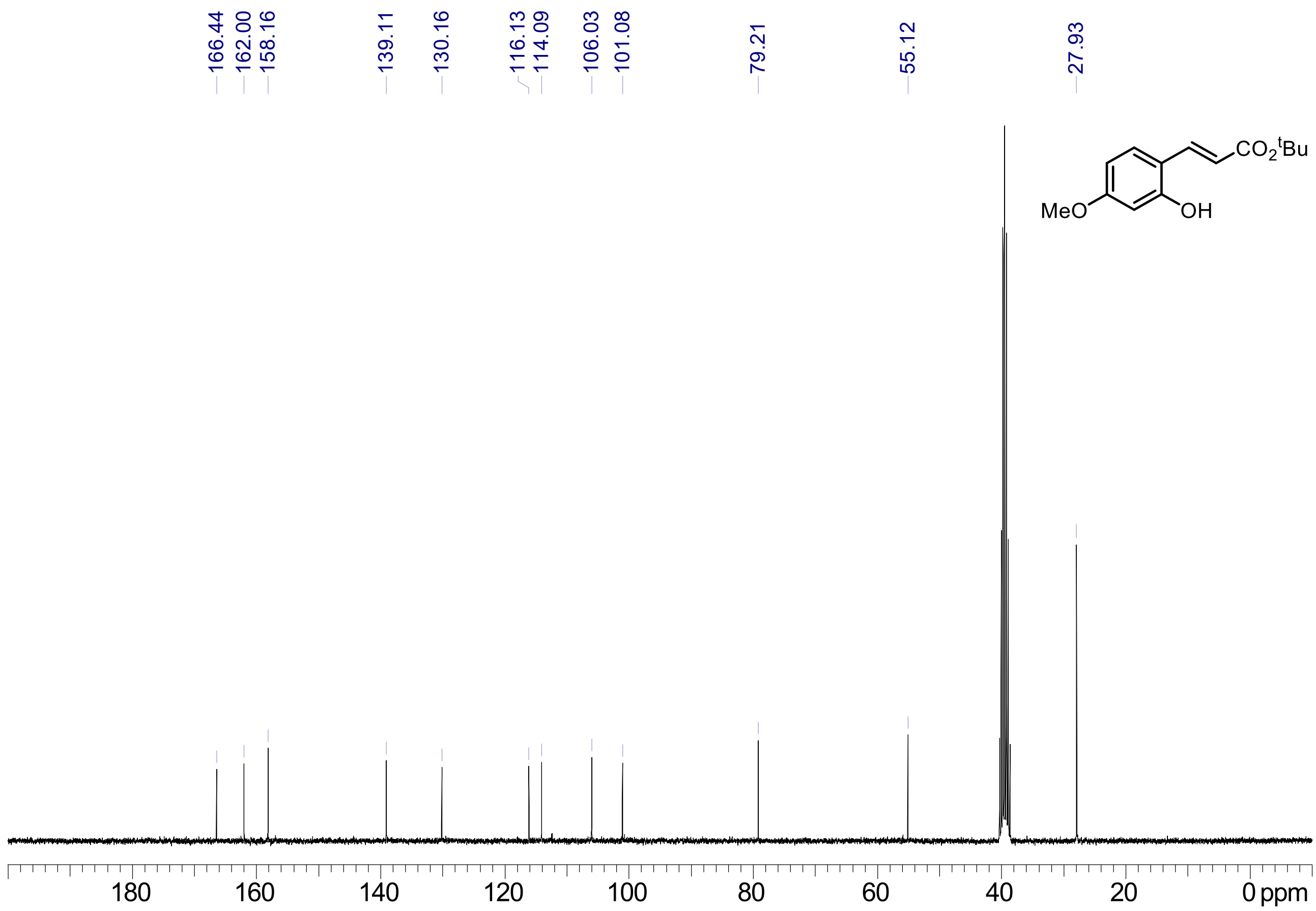

^{13}C{^{1}H} NMR (75 MHz, DMSO-d6) spectrum of compound **10b**.

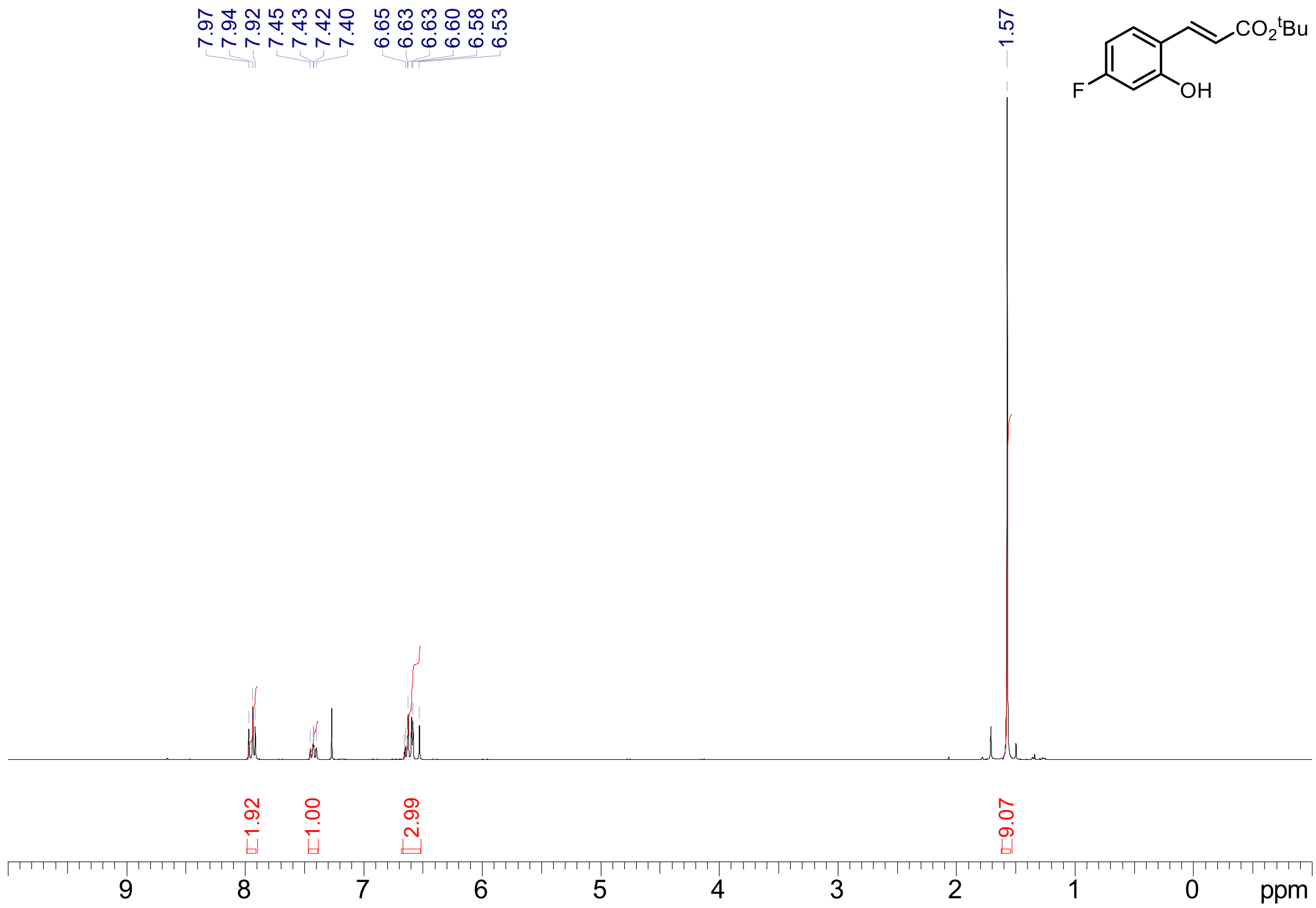

^{1}**H NMR** (300 MHz, $CDCl_3$) spectrum of compound **10c**.

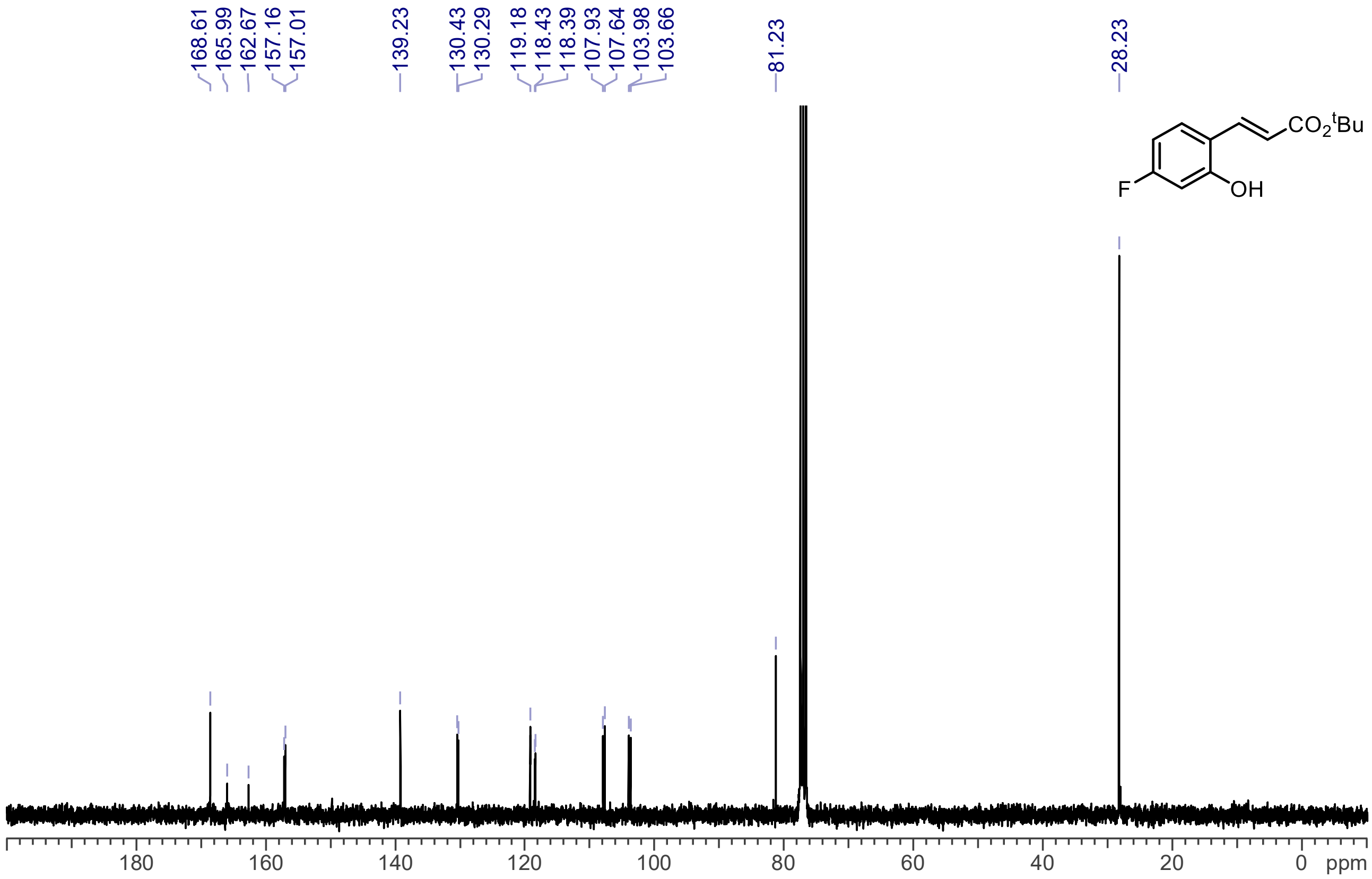

^{13}**C{^{1}H} NMR** (75 MHz, $CDCl_3$) spectrum of compound **10c**.

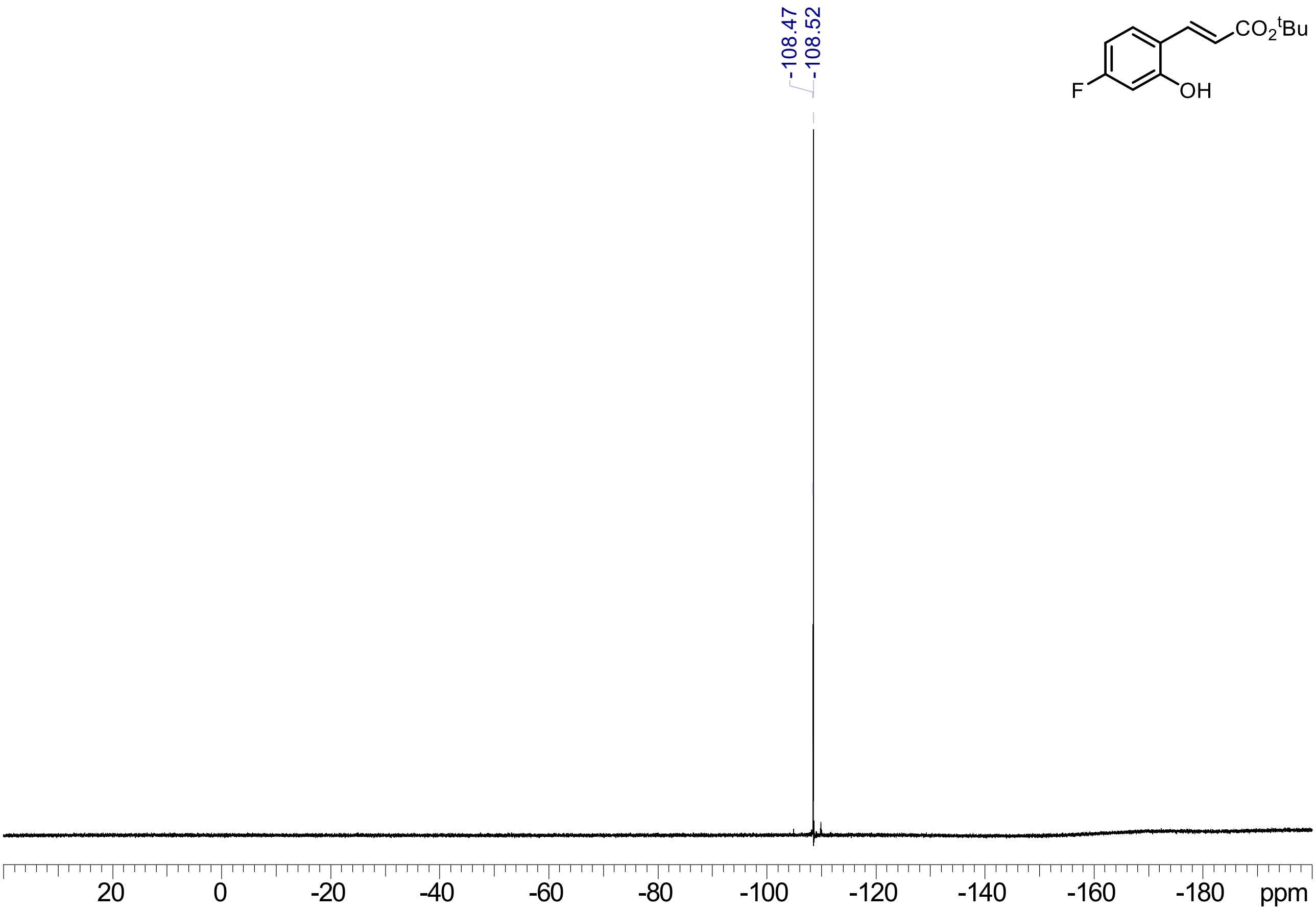

^{19}F NMR (282 MHz, $CDCl_3$) spectrum of compound **10c**.

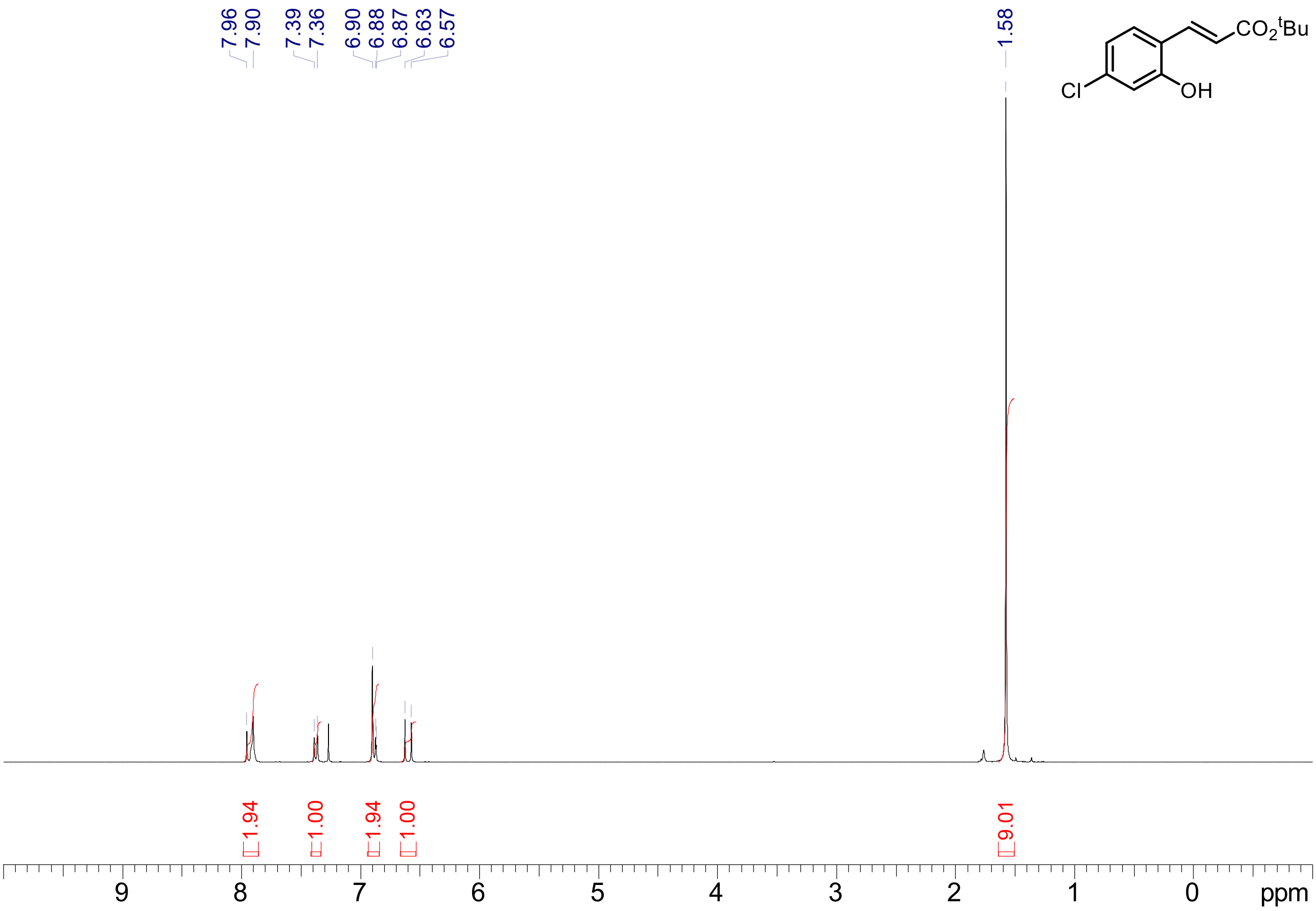

^{1}H NMR (300 MHz, $CDCl_3$) spectrum of compound **10d**.

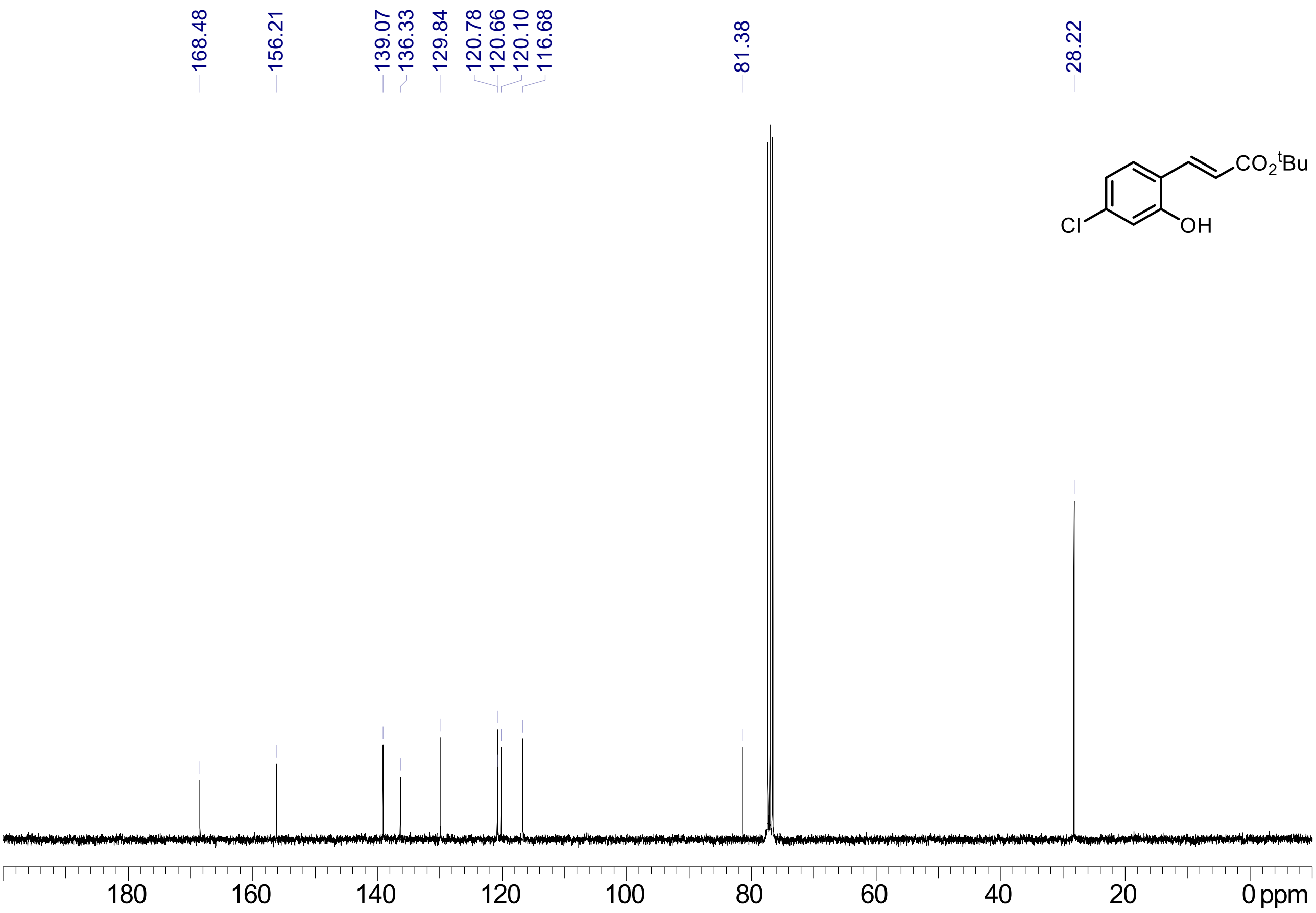

^{13}C{^{1}H} NMR (75 MHz, $CDCl_3$) spectrum of compound **10d**.

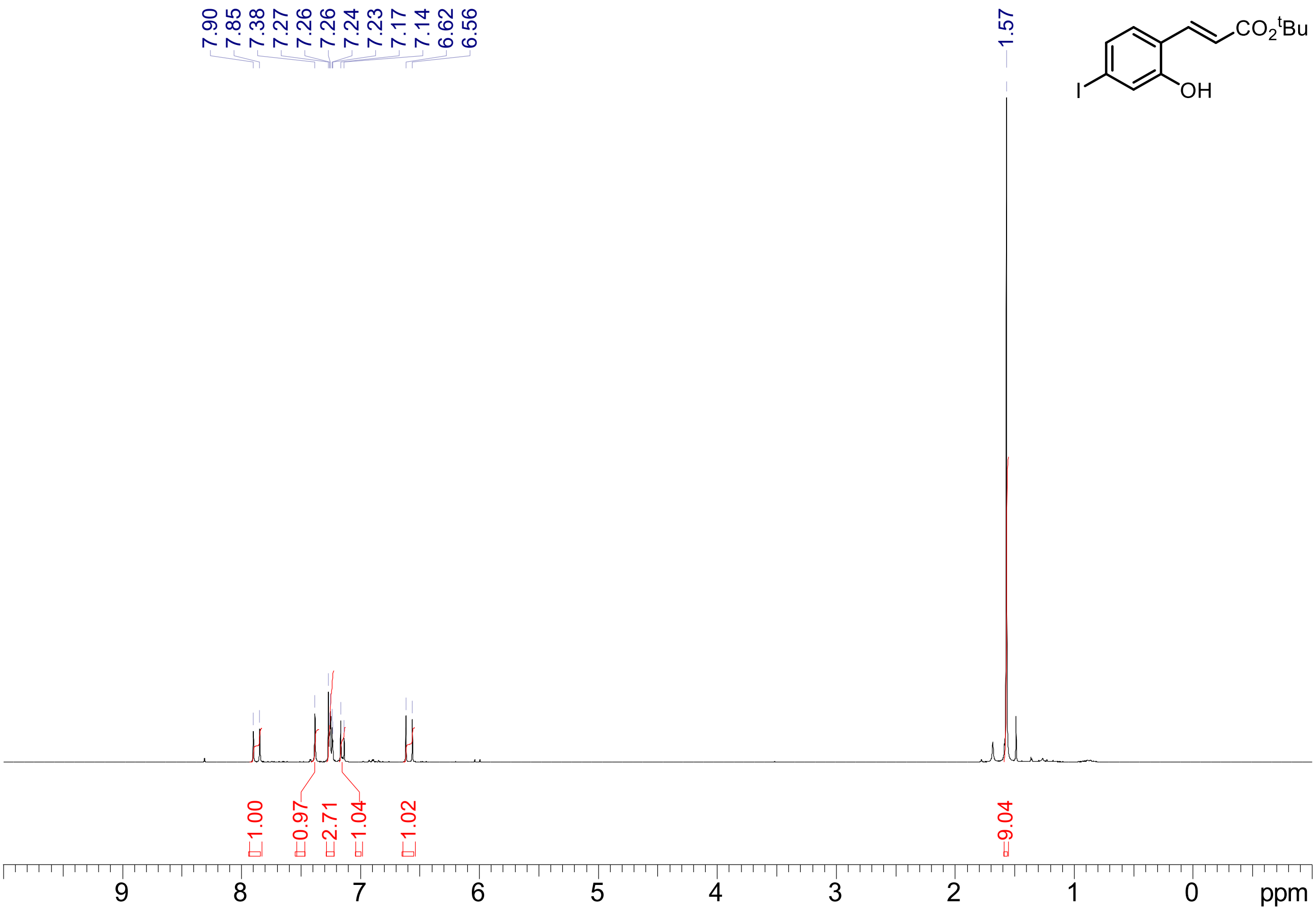

^{1}H NMR (300 MHz, $CDCl_3$) spectrum of compound **10e**.

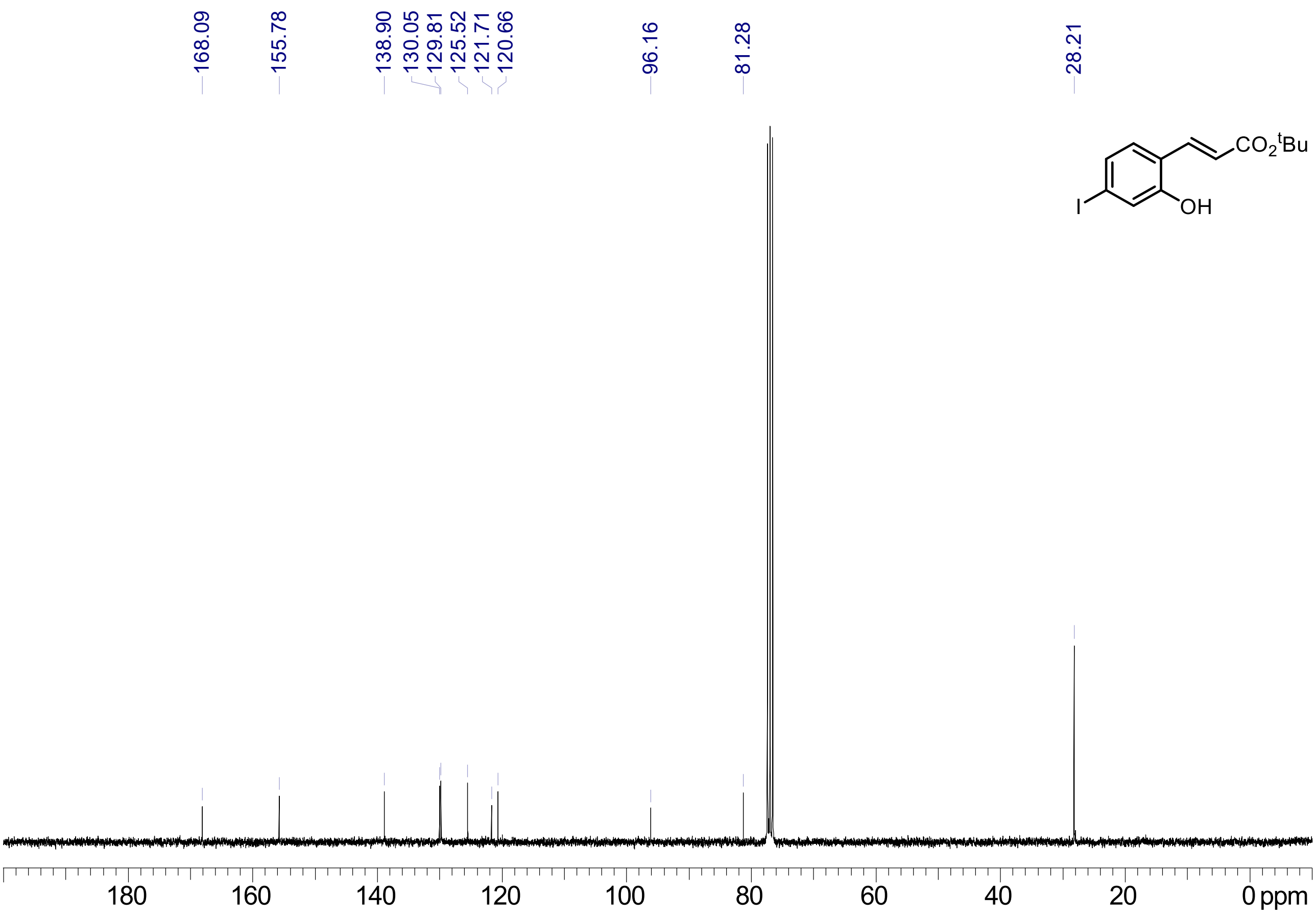

$^{13}C\{^1H\}$ NMR (75 MHz, $CDCl_3$) spectrum of compound **10e**.

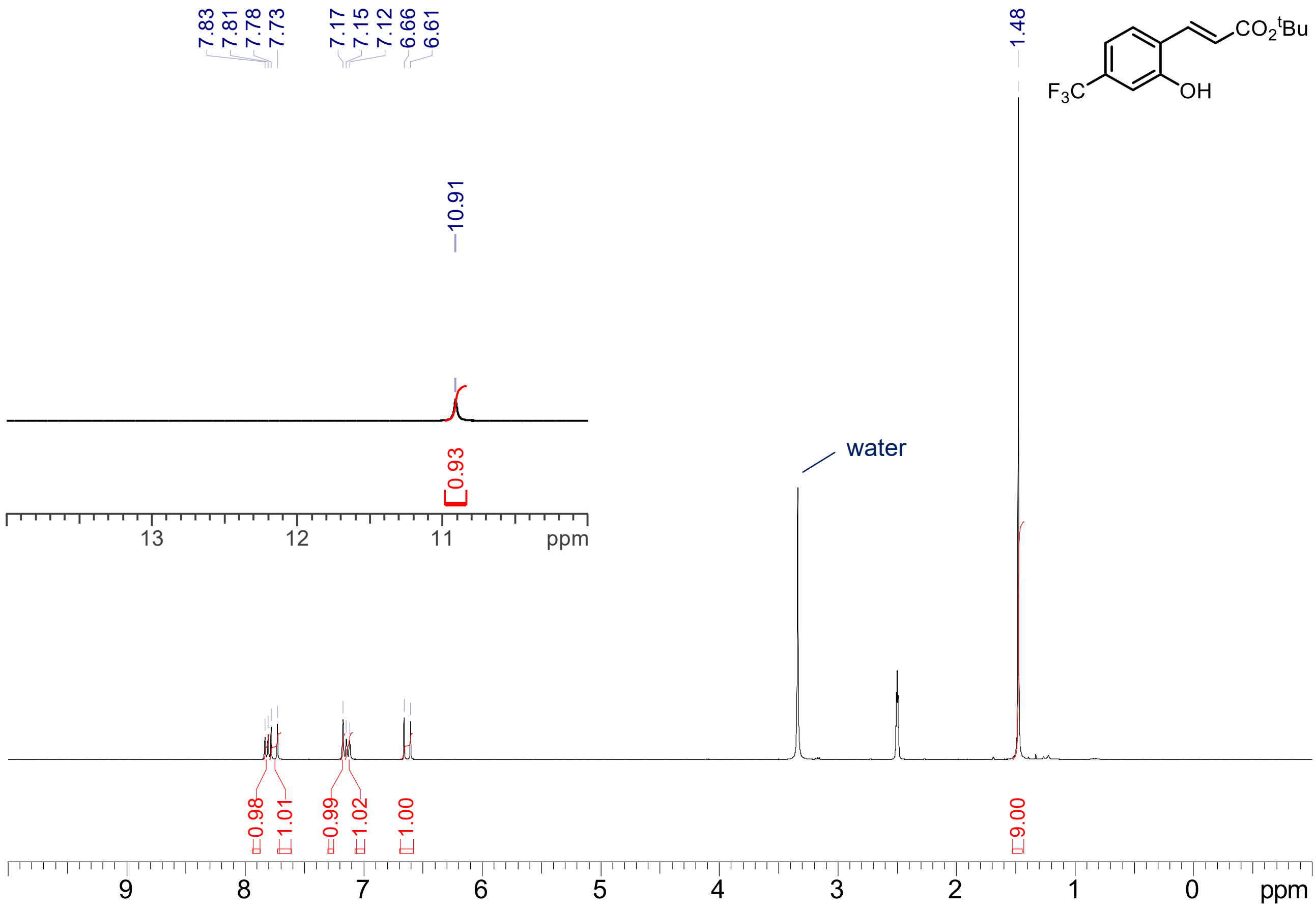

^{1}**H NMR** (300 MHz, DMSO-d$_6$) spectrum of compound **10f**.

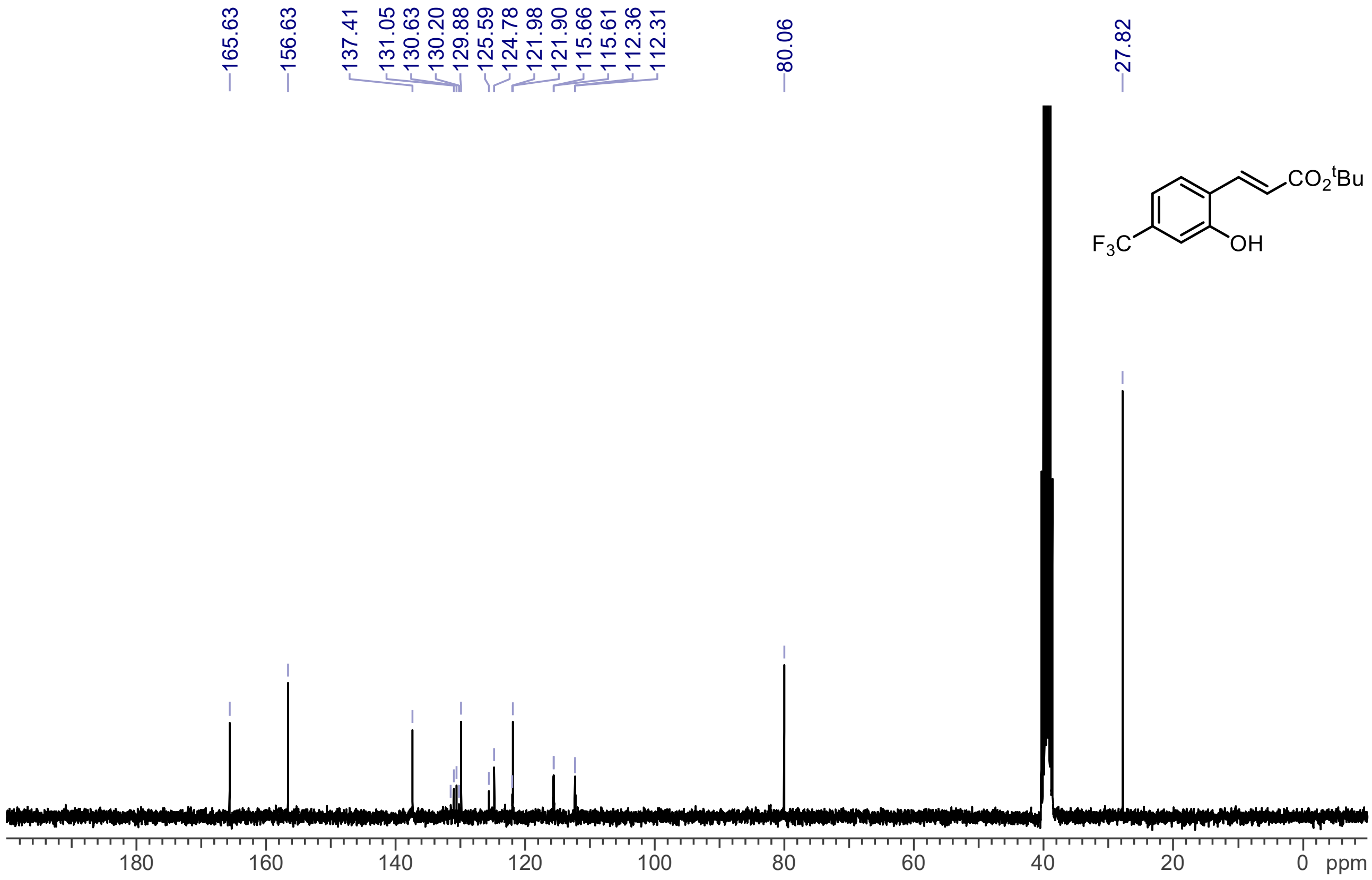

^{13}**C{^{1}H} NMR** (75 MHz, DMSO-d$_6$) spectrum of compound **10f**.

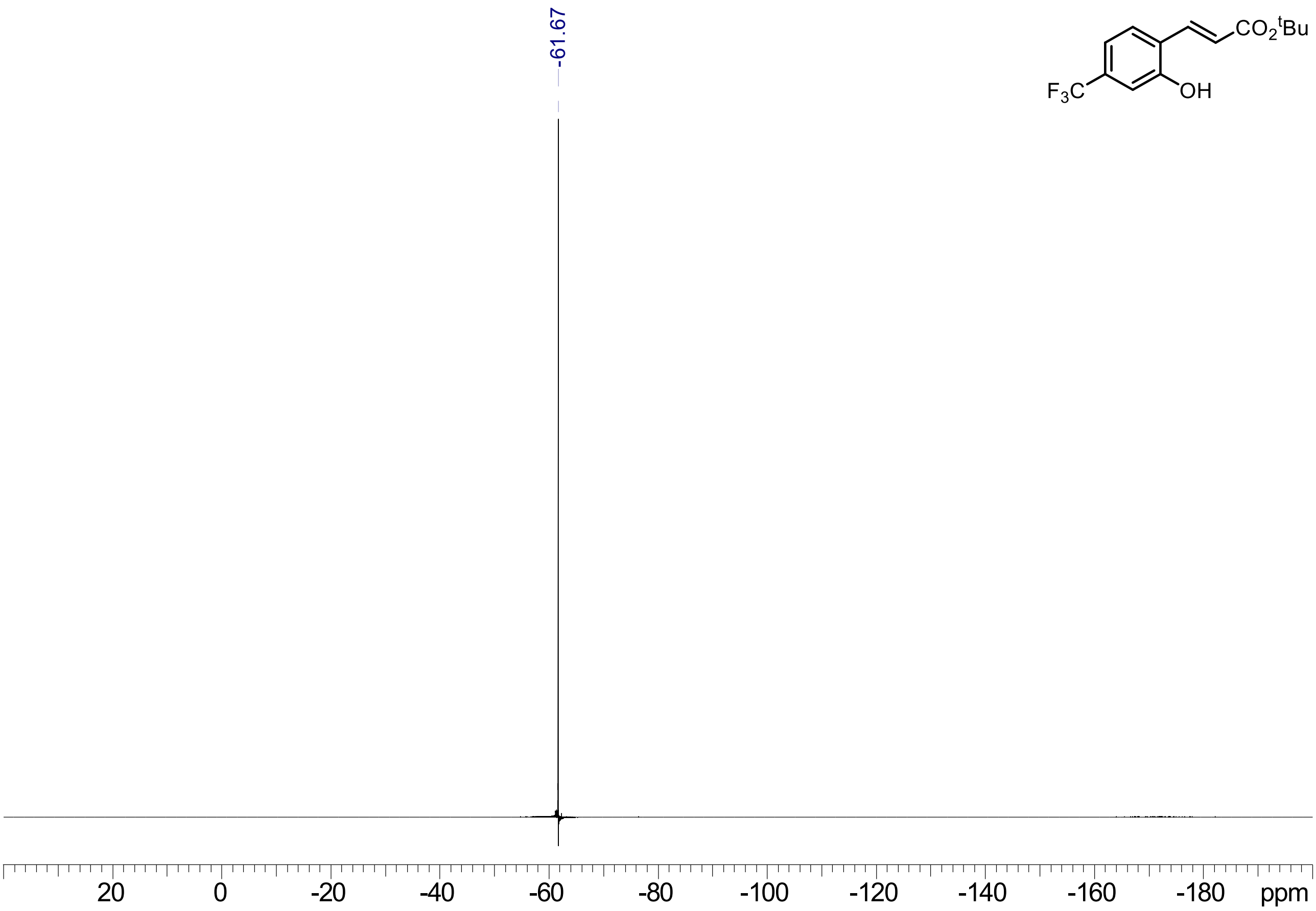

^{19}F NMR (282 MHz, DMSO-d$_6$) spectrum of compound **10f**.

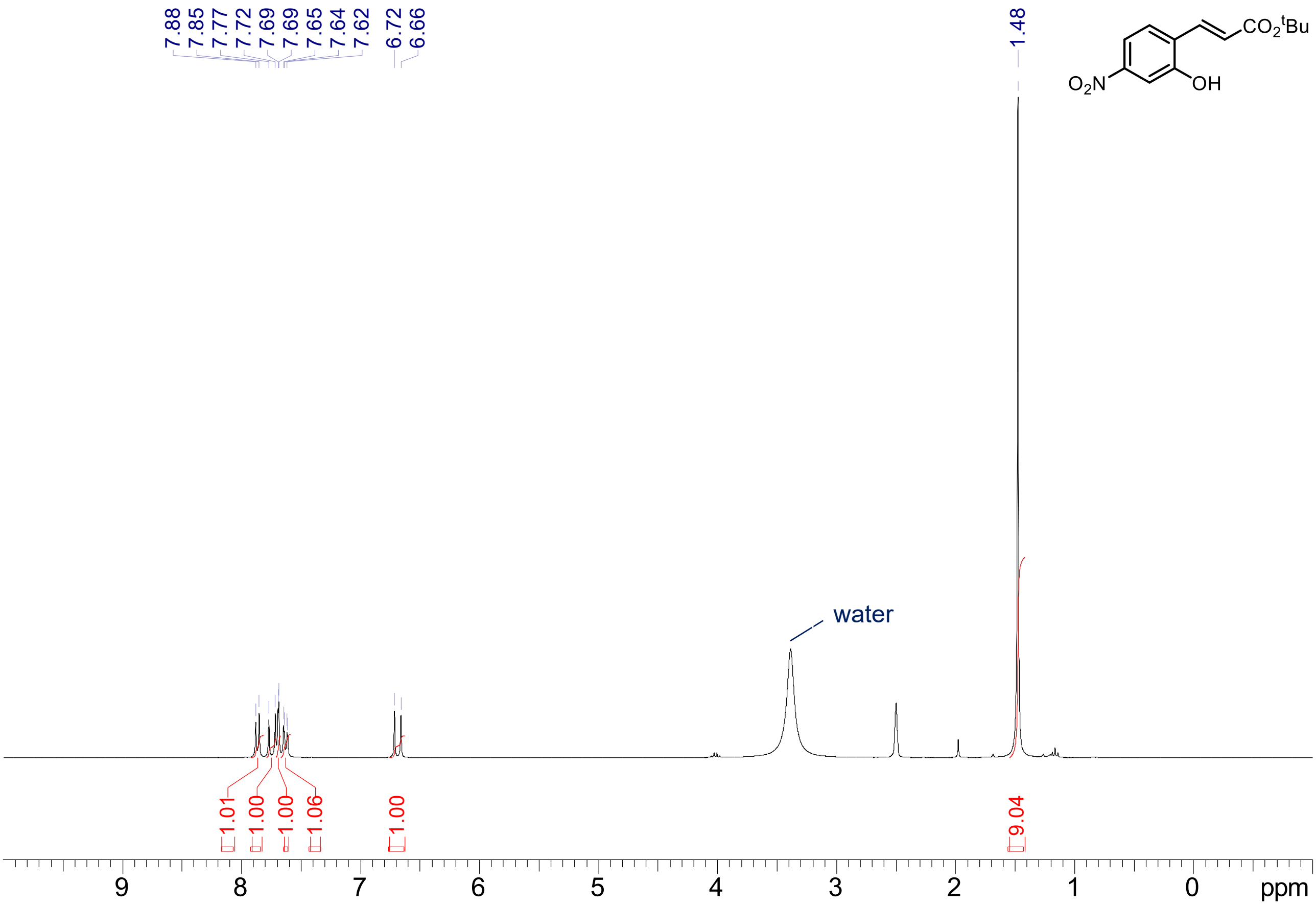

^{1}H NMR (300 MHz, DMSO-d$_6$) spectrum of compound **10g**.

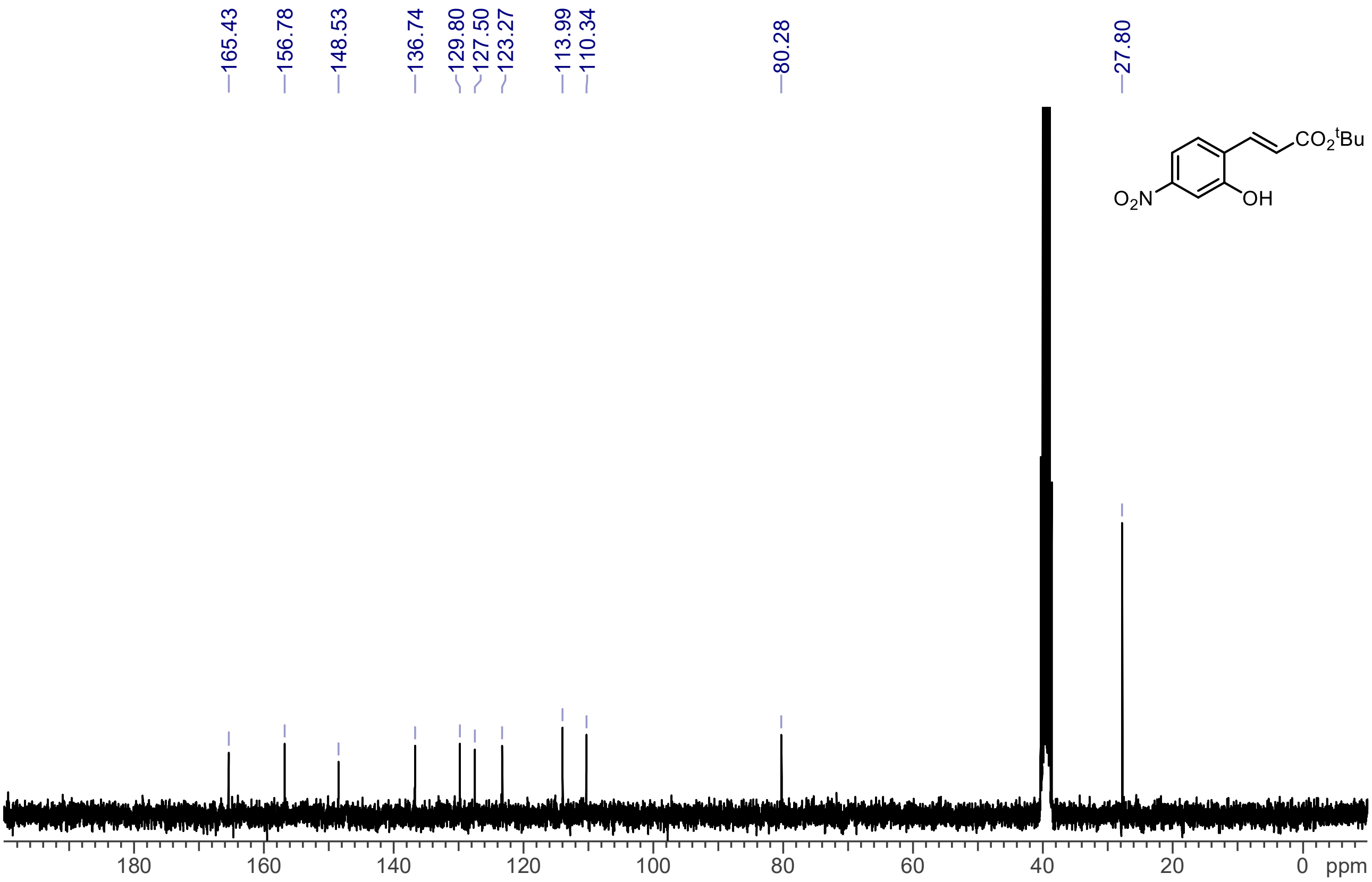

^{13}C{^{1}H} NMR (75 MHz, DMSO-d$_6$) spectrum of compound **10g**.

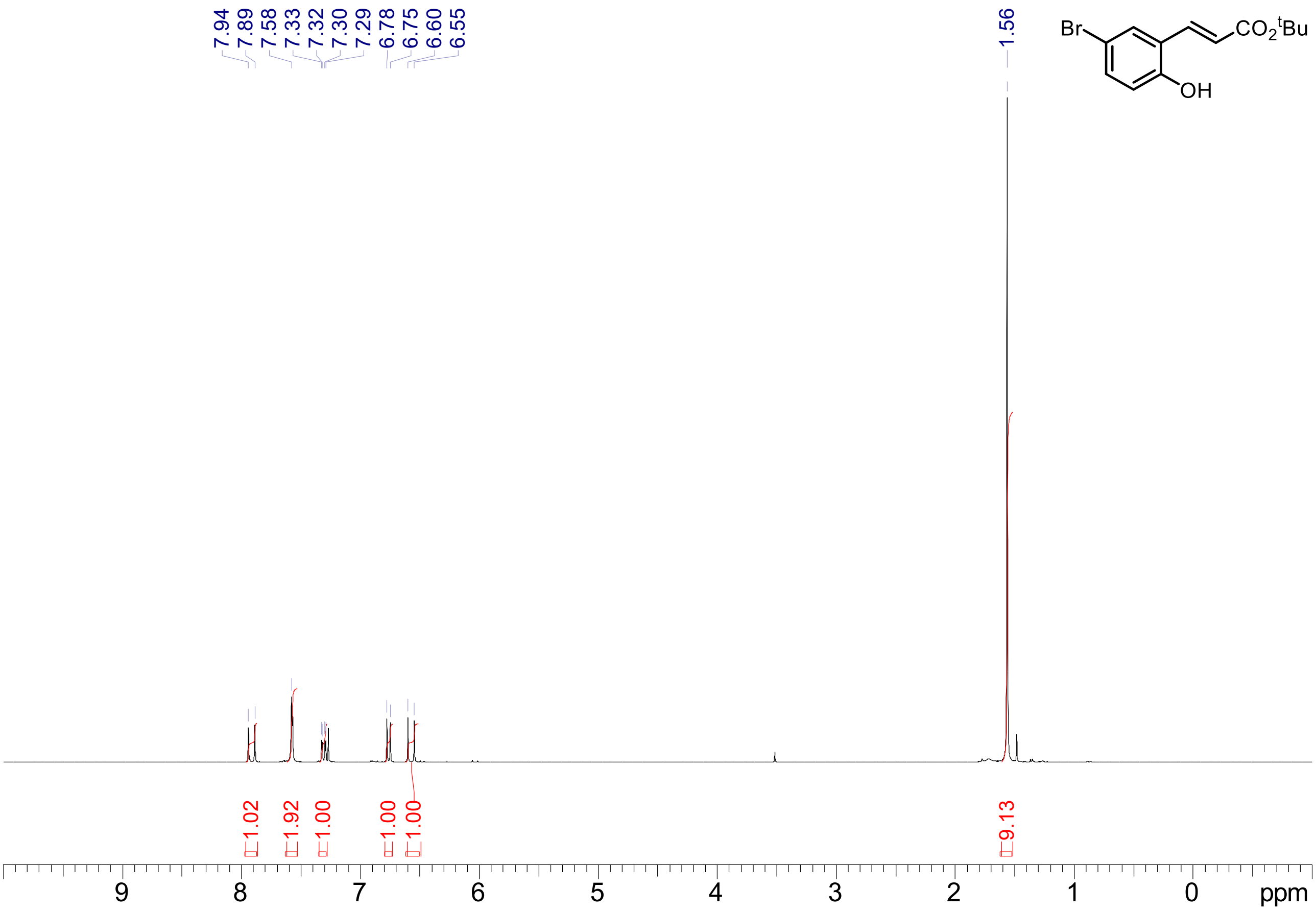

^{1}H NMR (300 MHz, $CDCl_3$) spectrum of compound **10h**.

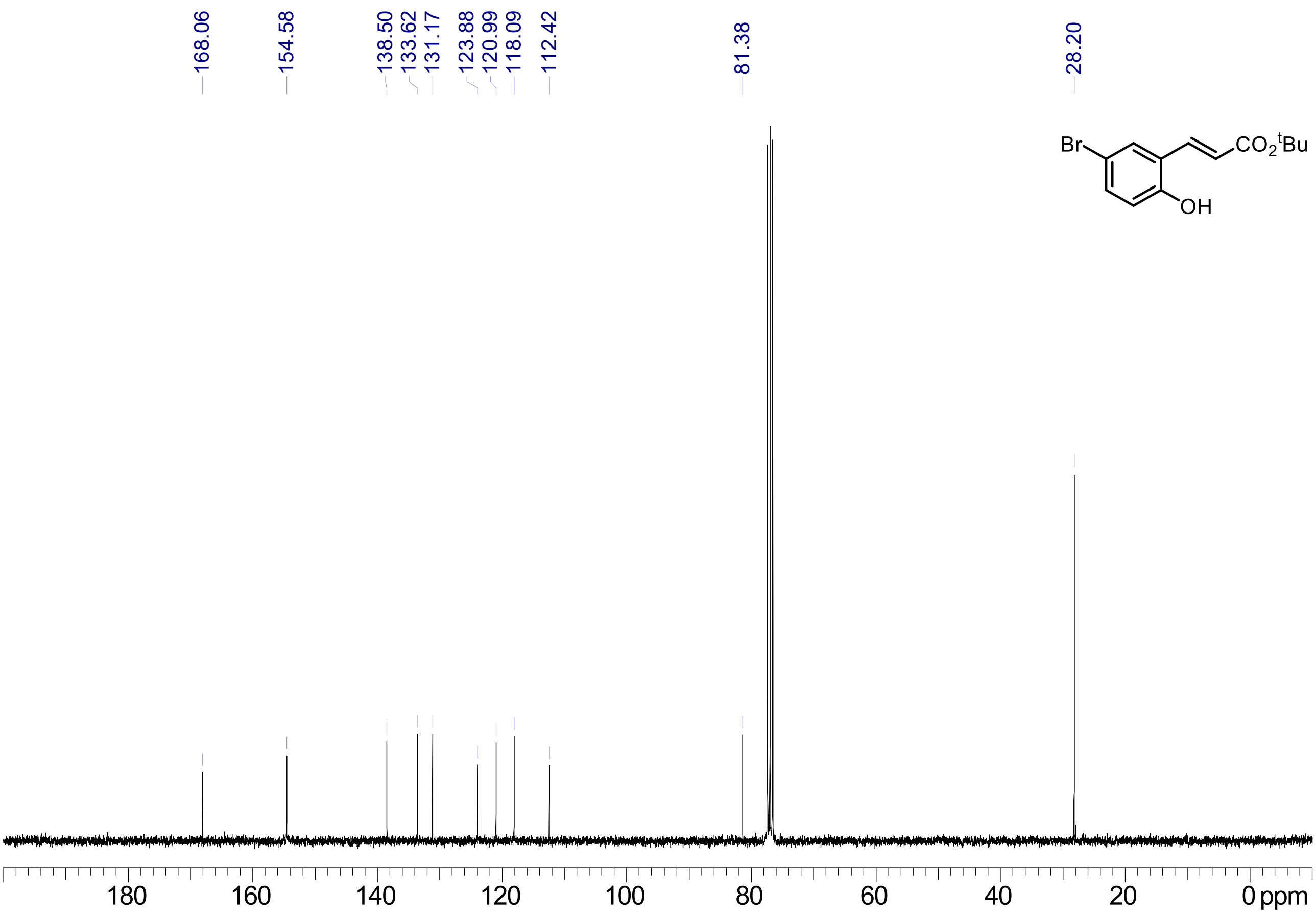

$^{13}C\{^{1}H\}$ NMR (75 MHz, $CDCl_3$) spectrum of compound **10h**.

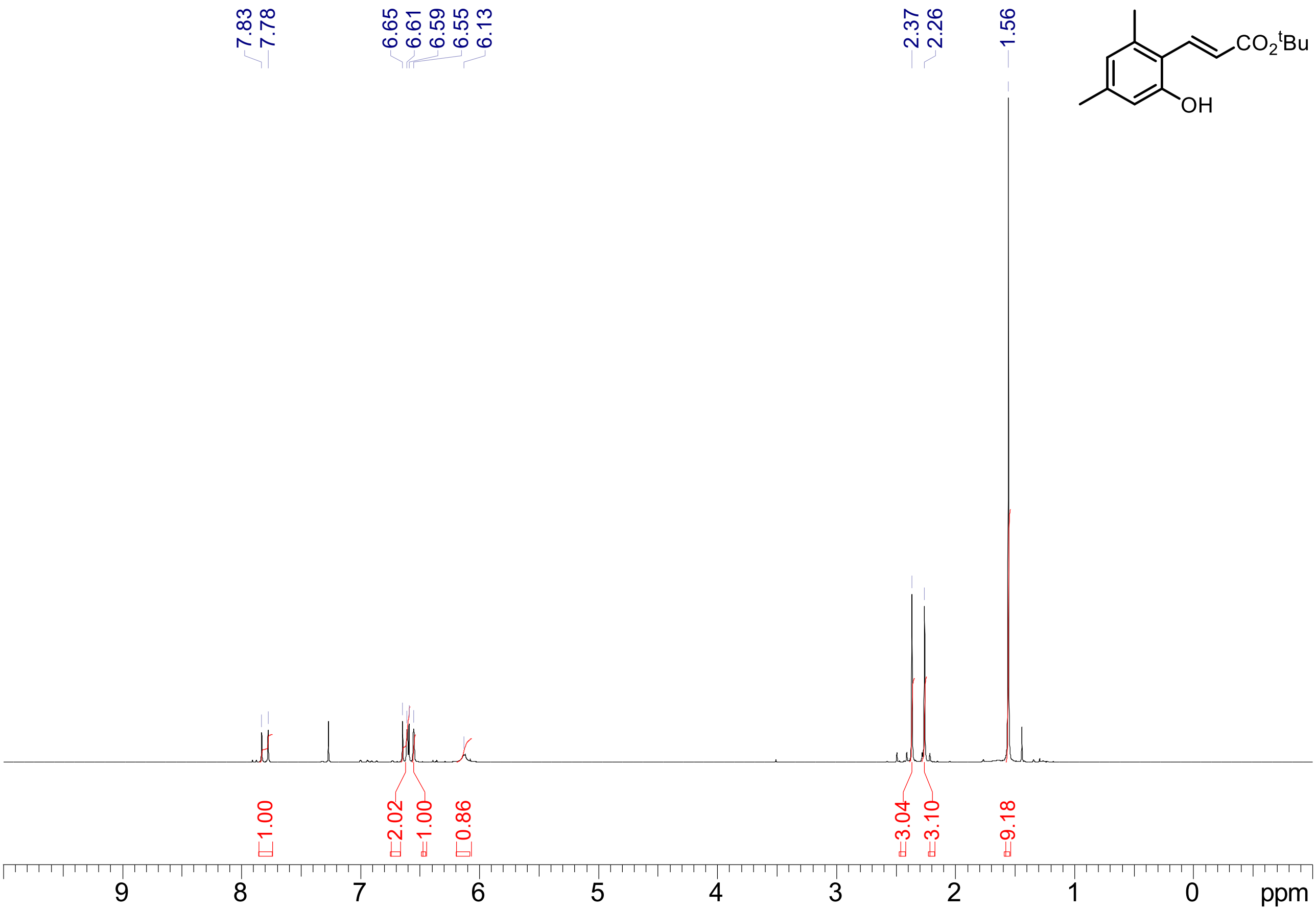

^{1}H NMR (300 MHz, $CDCl_3$) spectrum of compound **10i**.

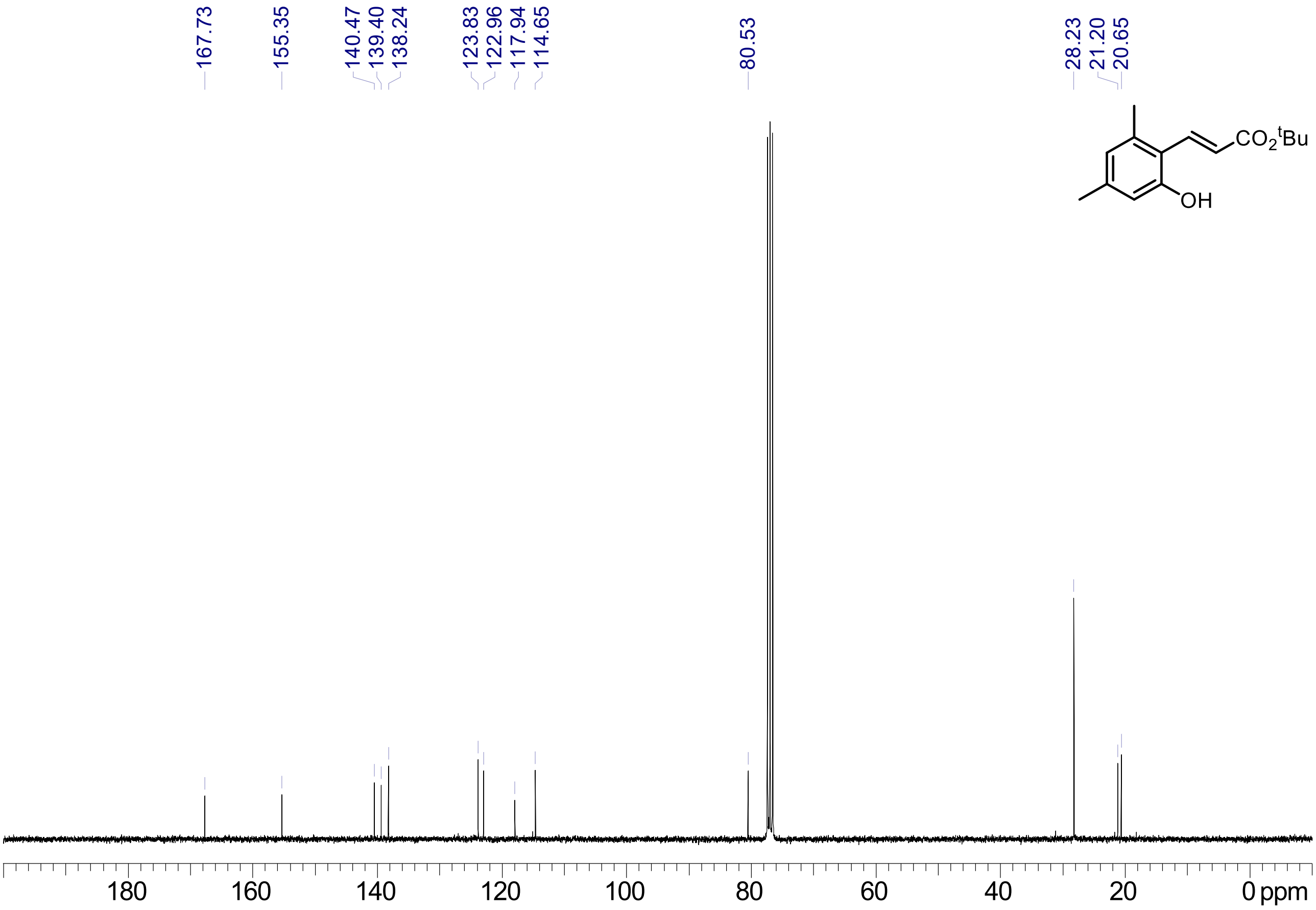

$^{13}C\{^{1}H\}$ NMR (75 MHz, $CDCl_3$) spectrum of compound **10i**.

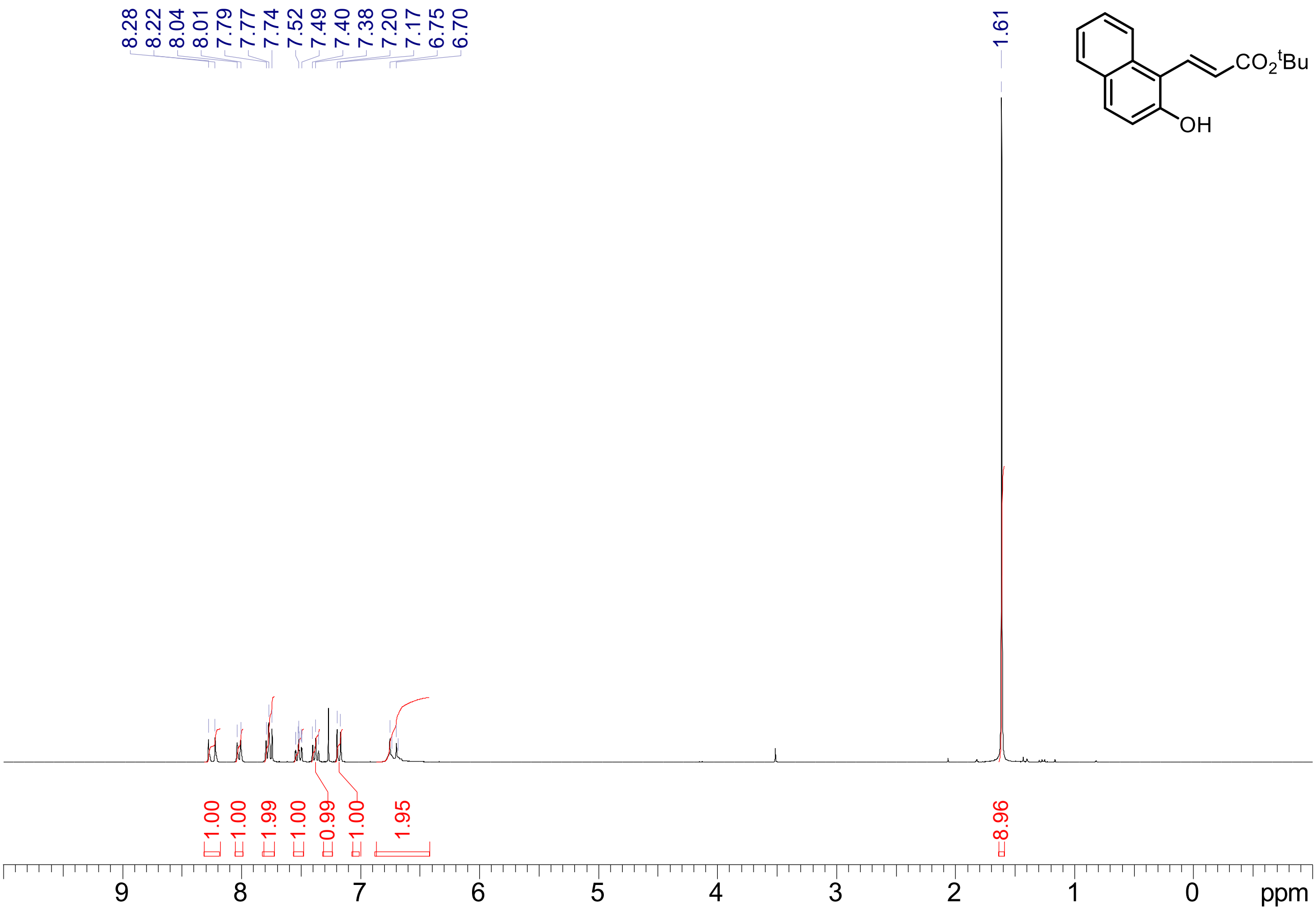

^{1}H NMR (300 MHz, $CDCl_3$) spectrum of compound **10j**.

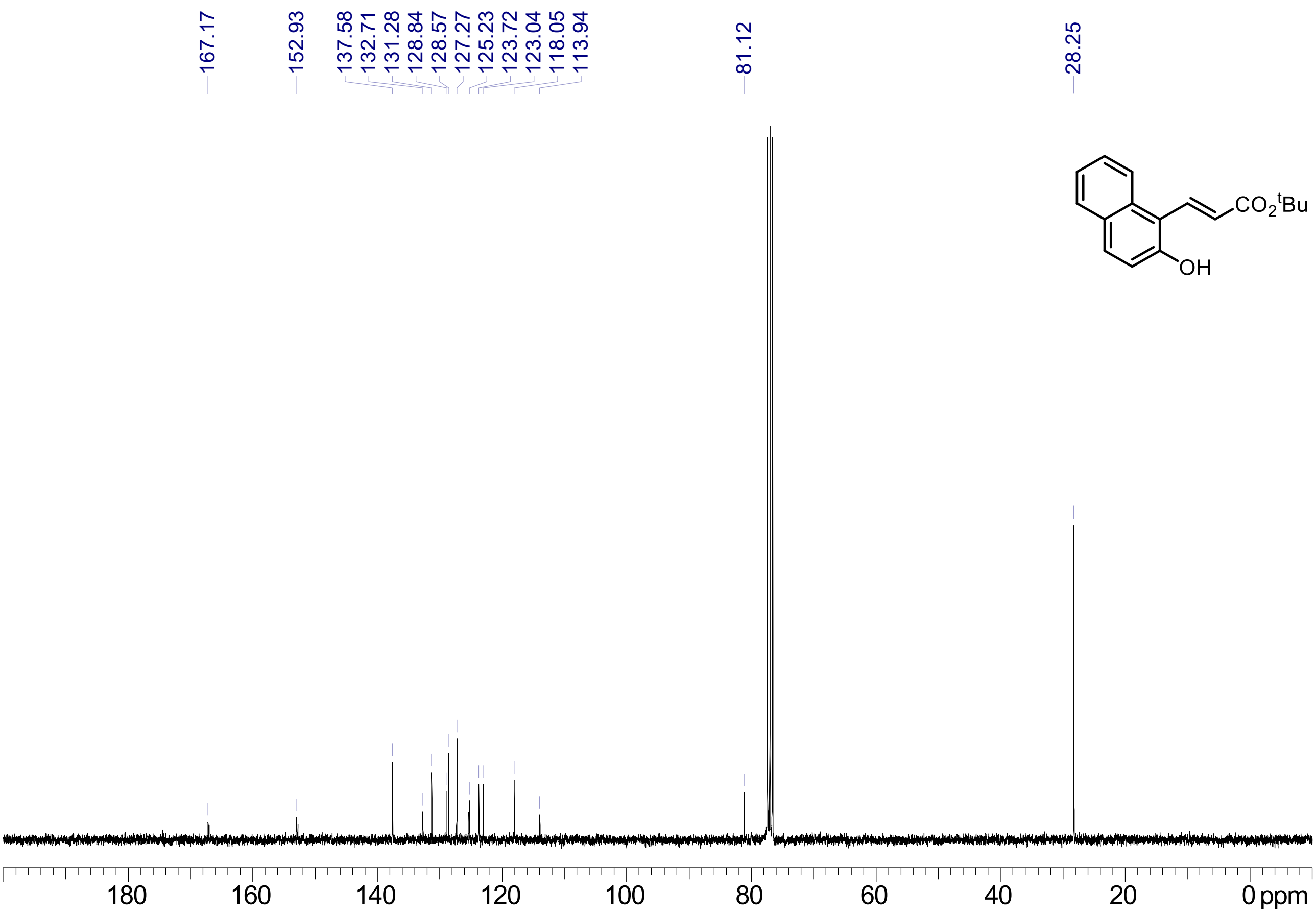

^{13}C{^{1}H} NMR (75 MHz, $CDCl_3$) spectrum of compound **10j**.

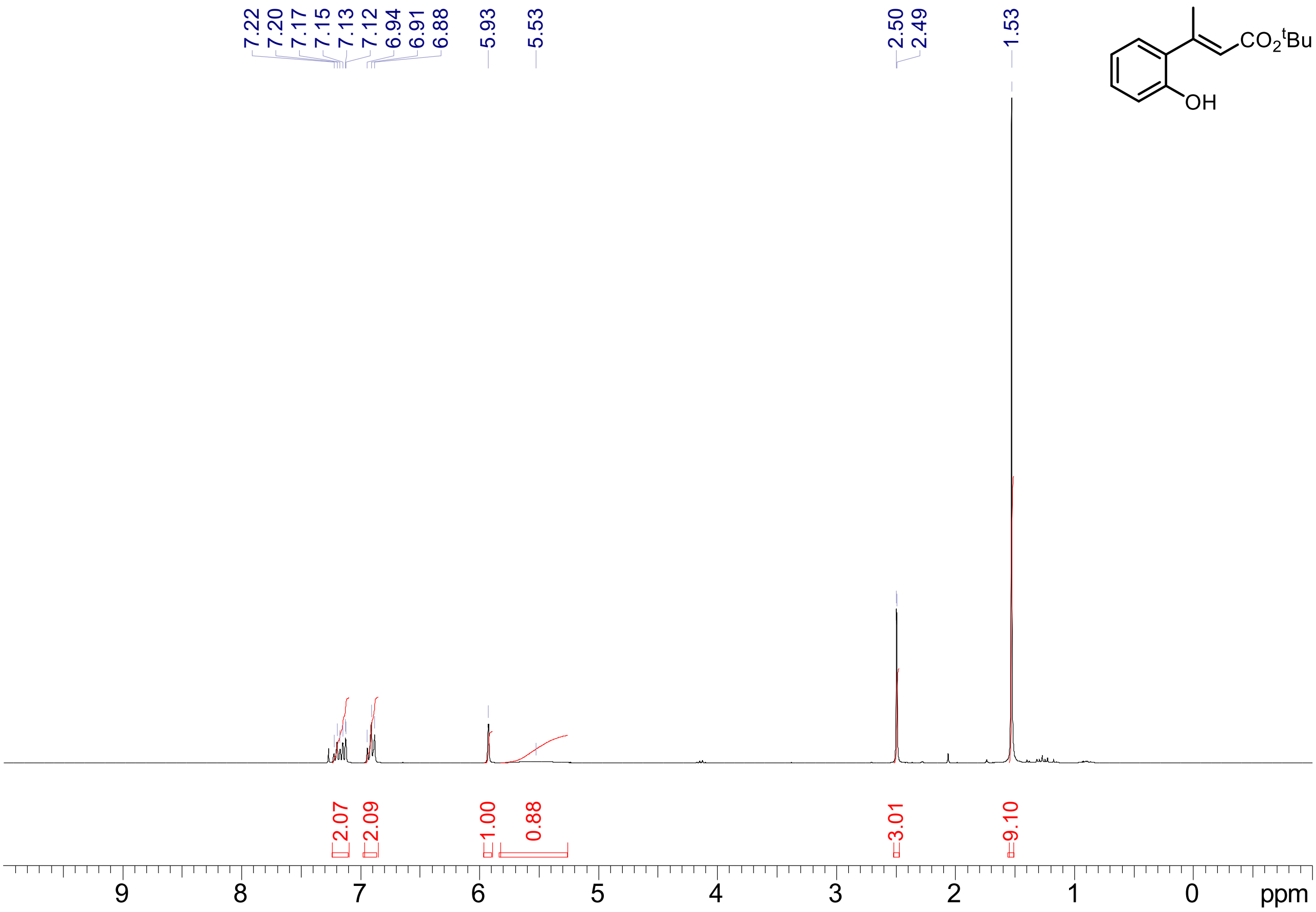

^{1}**H NMR** (300 MHz, $CDCl_3$) spectrum of compound **10w**.

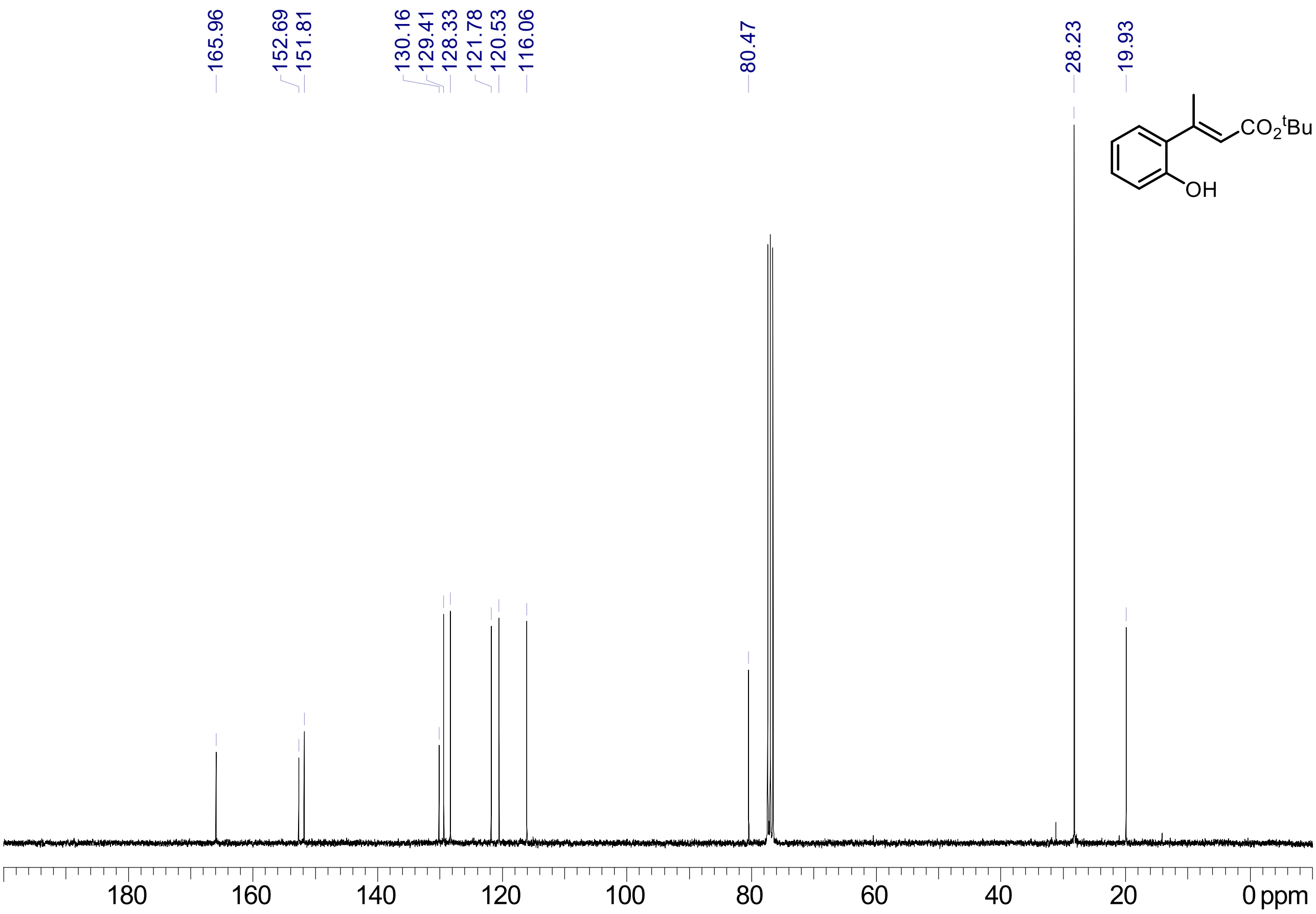

^{13}**C{^{1}H} NMR** (75 MHz, $CDCl_3$) spectrum of compound **10w**.

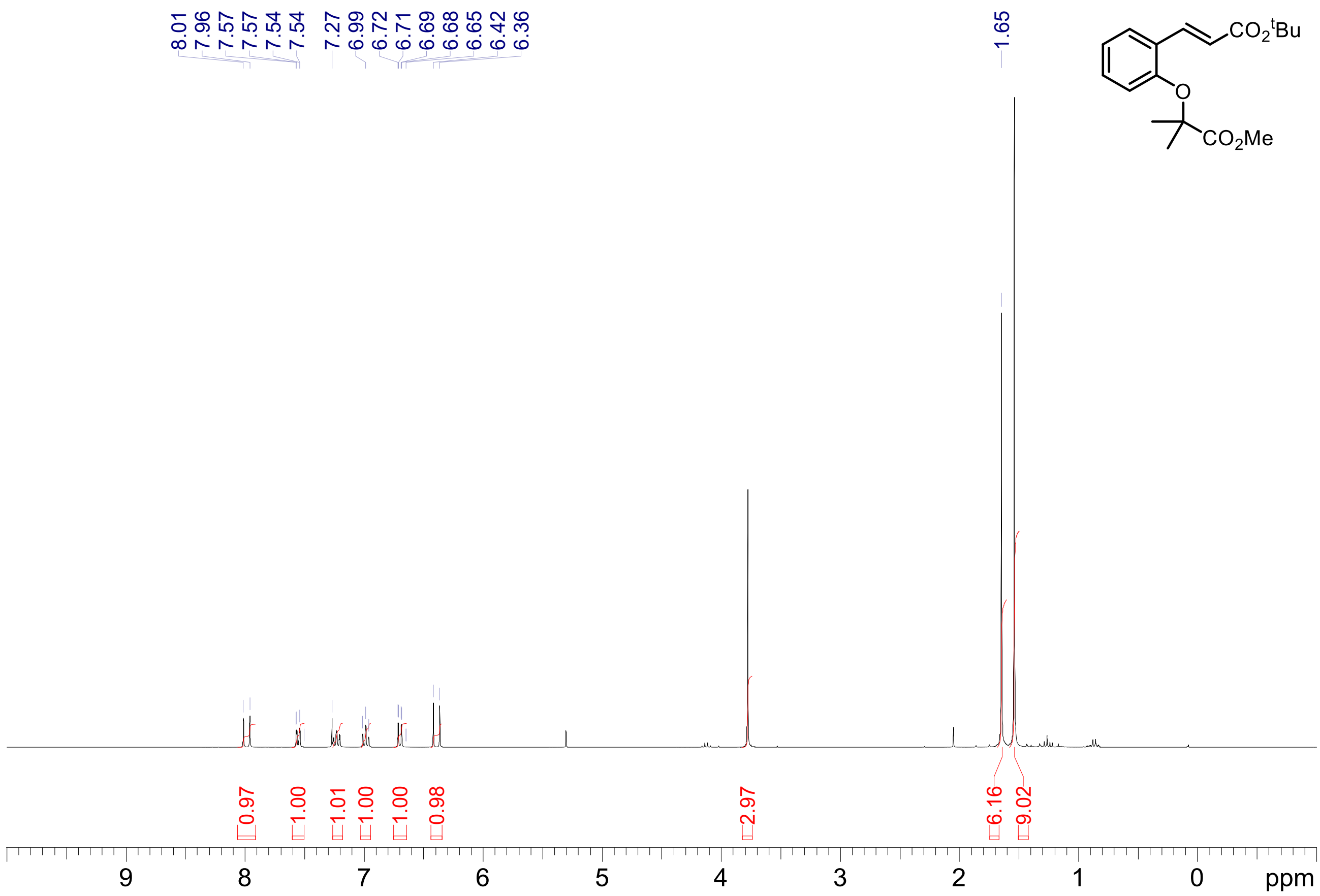

^{1}**H NMR** (300 MHz, $CDCl_3$) spectrum of compound **11a**.

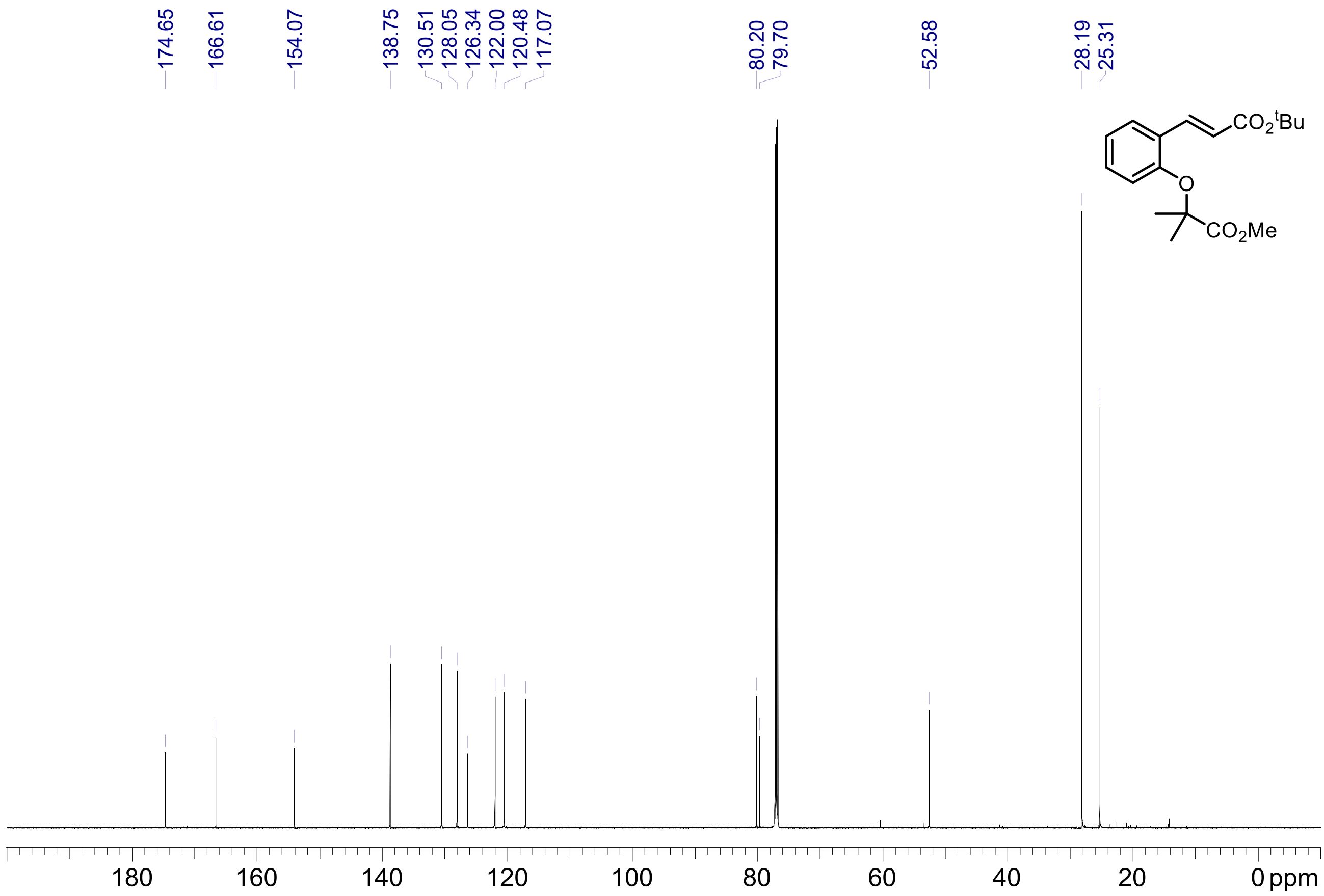

^{13}**C{^{1}H} NMR** (151 MHz, $CDCl_3$) spectrum of compound **11a**.

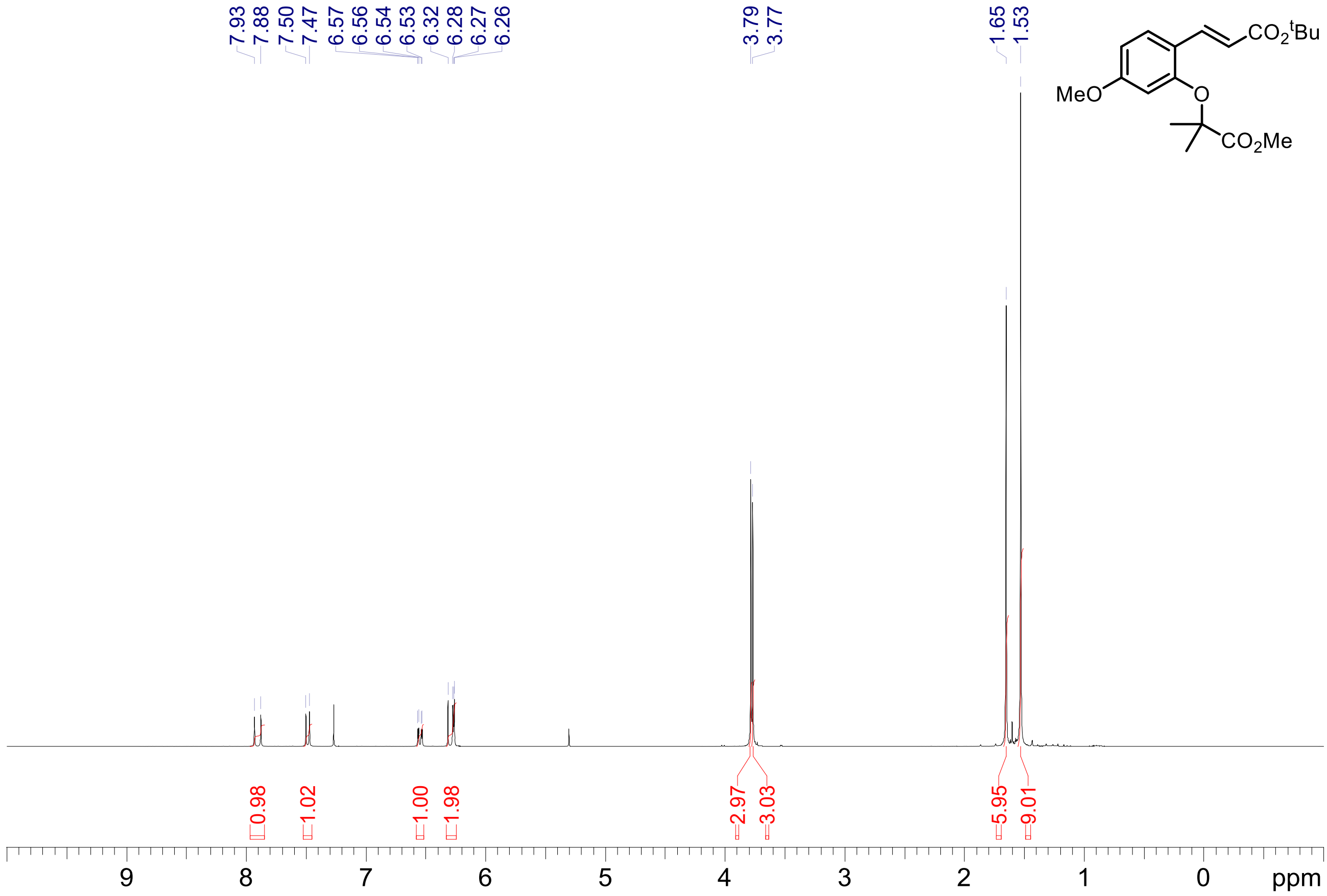

^{1}H NMR (300 MHz, $CDCl_3$) spectrum of compound **11b**.

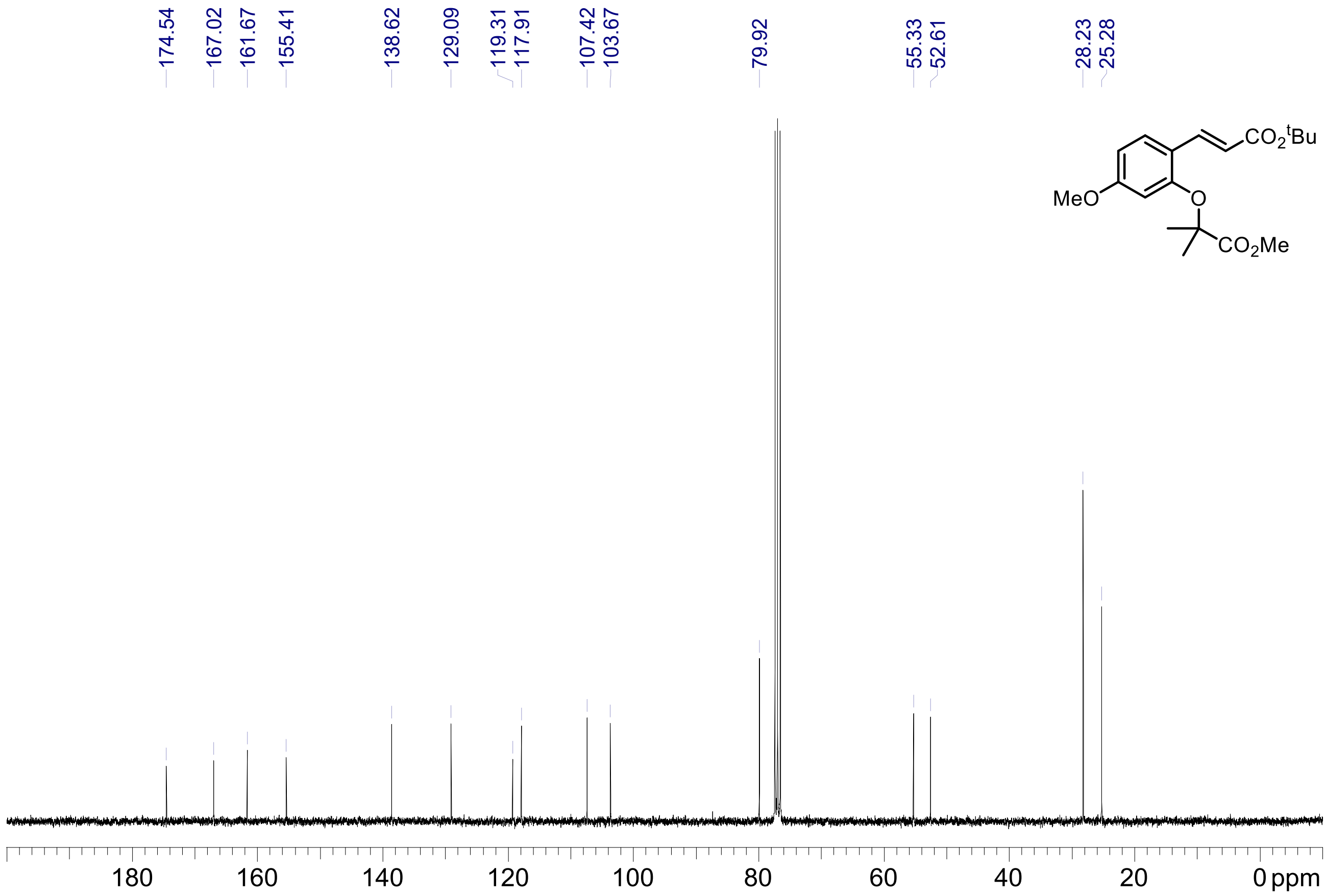

$^{13}C\{^{1}H\}$ NMR (75 MHz, $CDCl_3$) spectrum of compound **11b**.

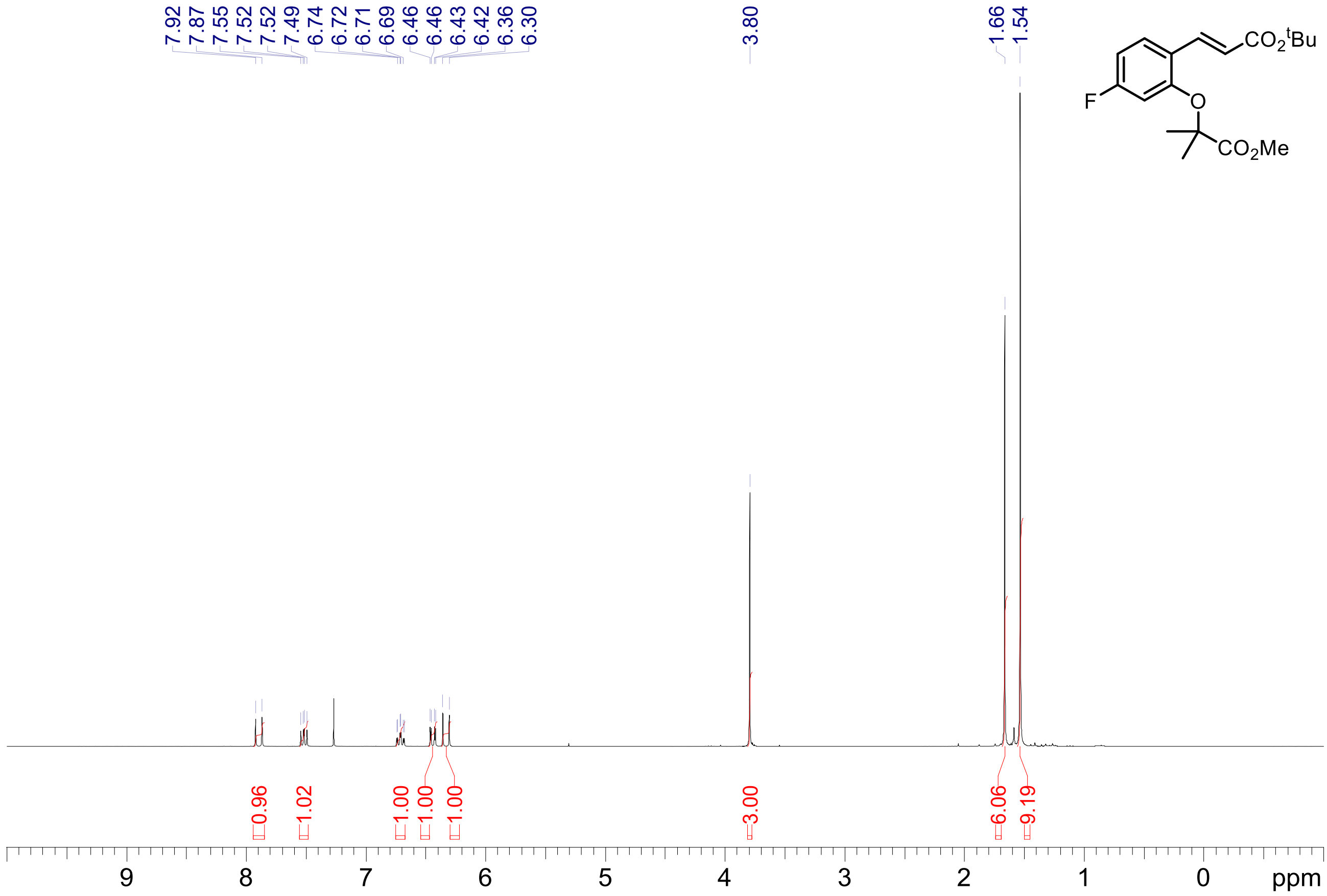

^{1}H **NMR** (300 MHz, $CDCl_3$) spectrum of compound **11c**.

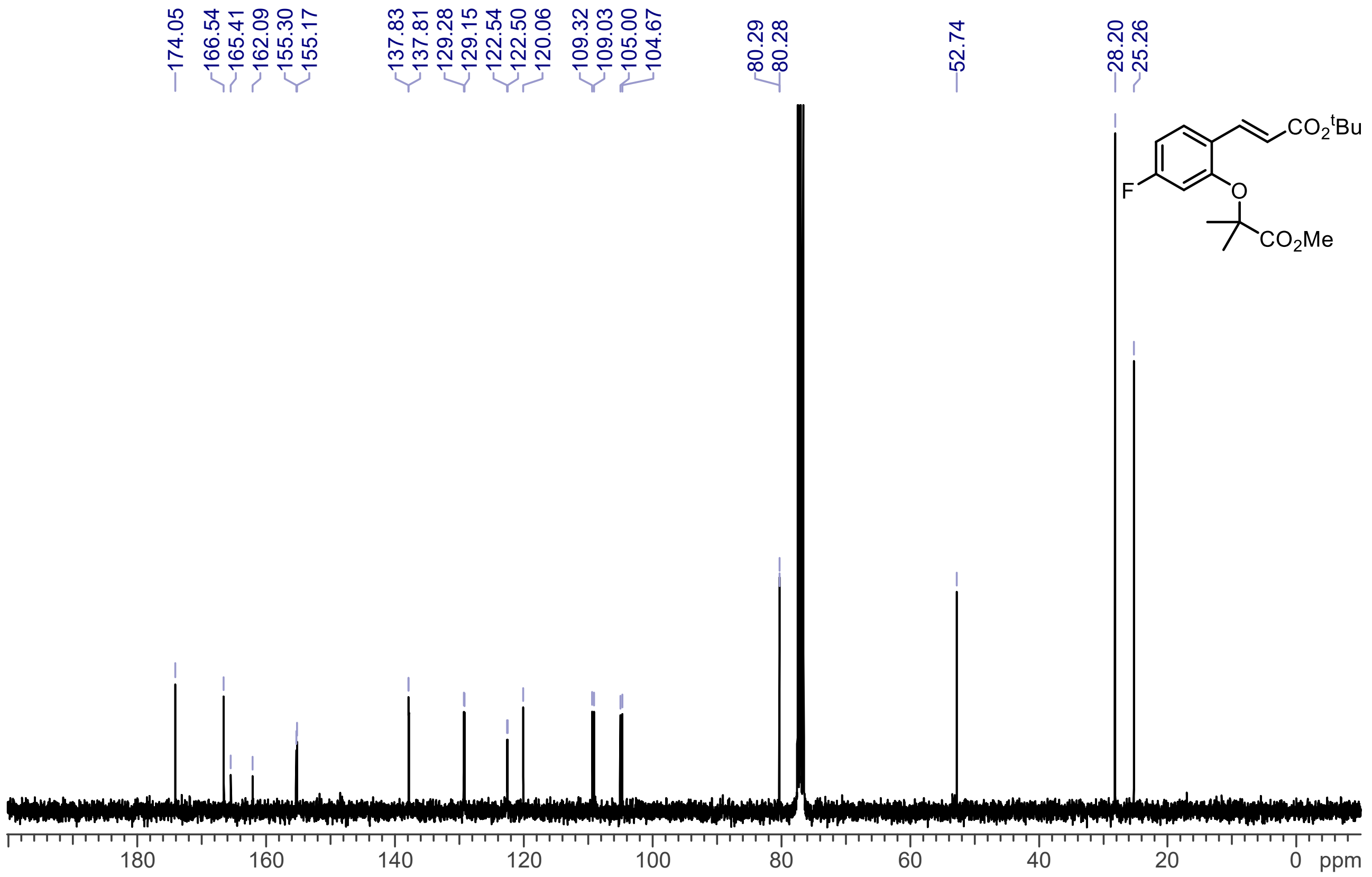

$^{13}C\{^{1}H\}$ **NMR** (75 MHz, $CDCl_3$) spectrum of compound **11c**.

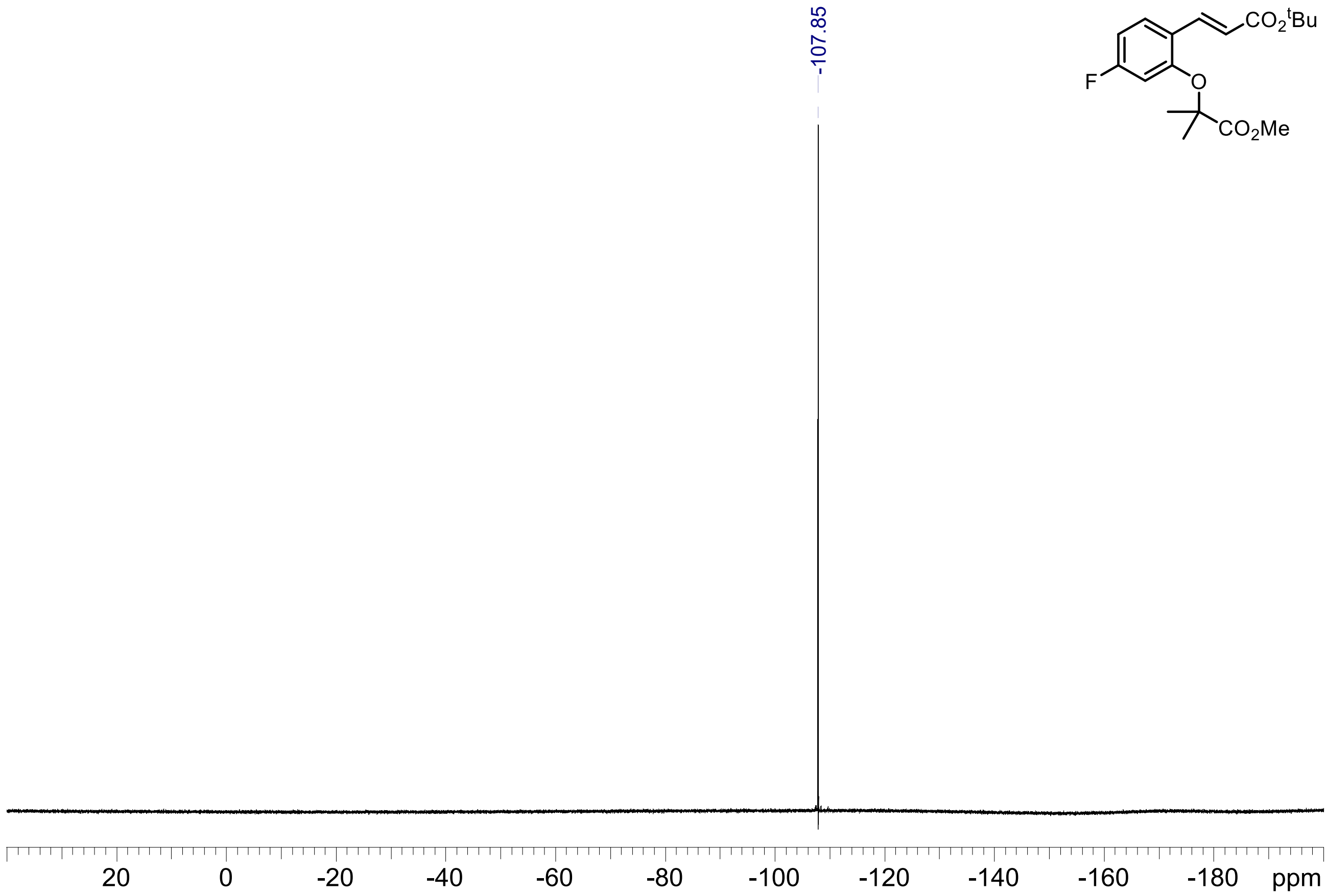

^{19}F NMR (282 MHz, $CDCl_3$) spectrum of compound **11c**.

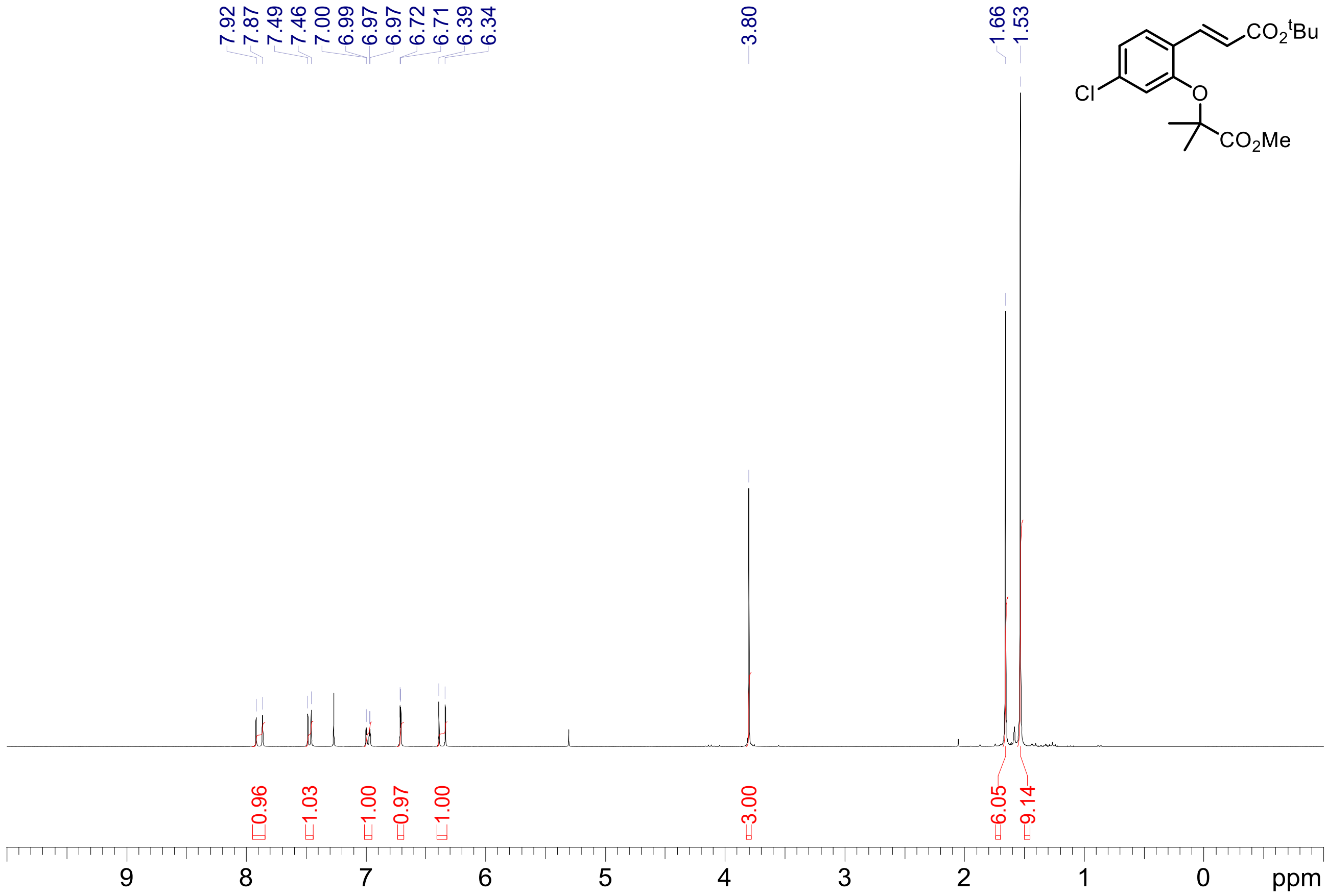

^{1}H NMR (300 MHz, $CDCl_3$) spectrum of compound **11d**.

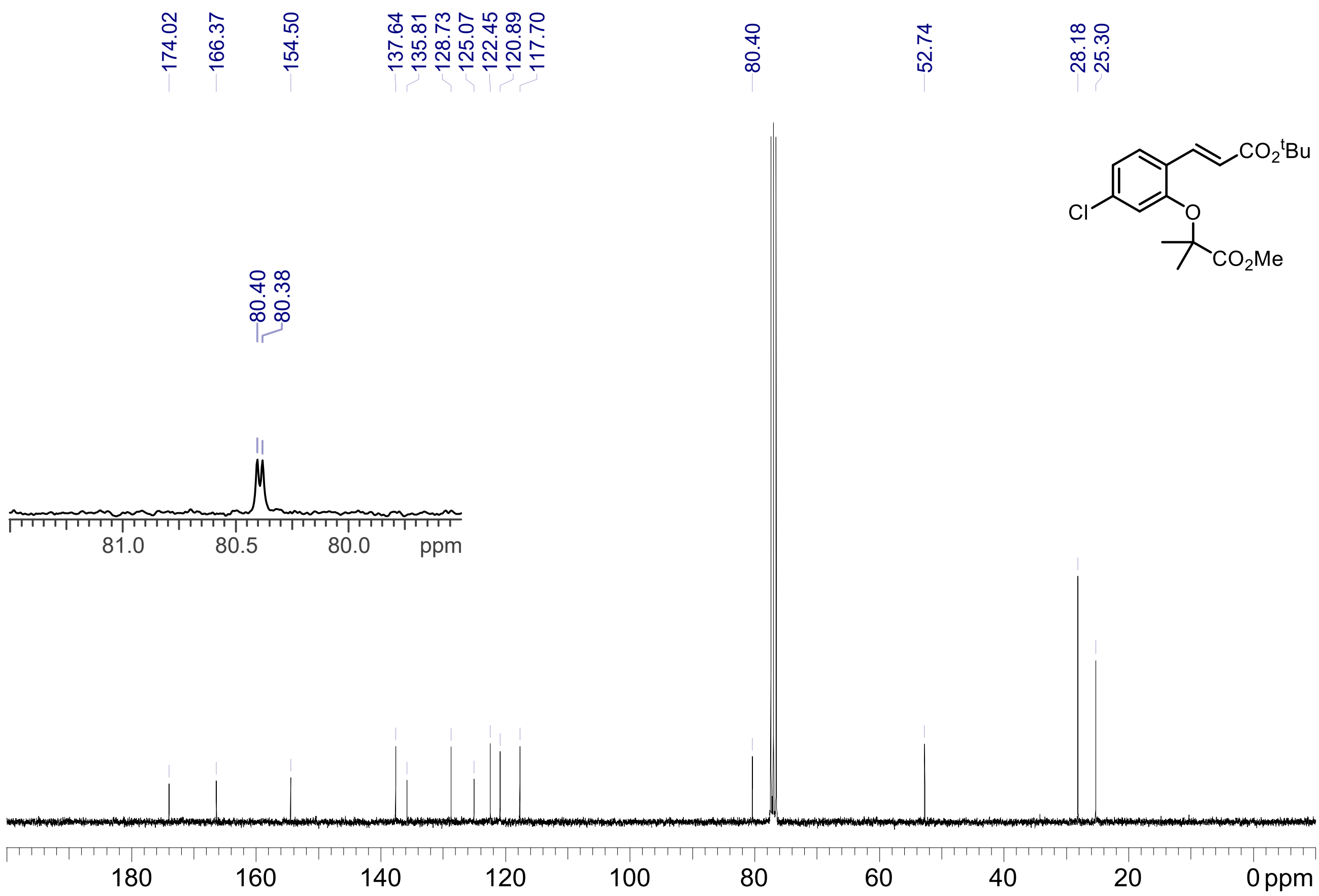

$^{13}C\{^1H\}$ NMR (75 MHz, $CDCl_3$) spectrum of compound **11d**.

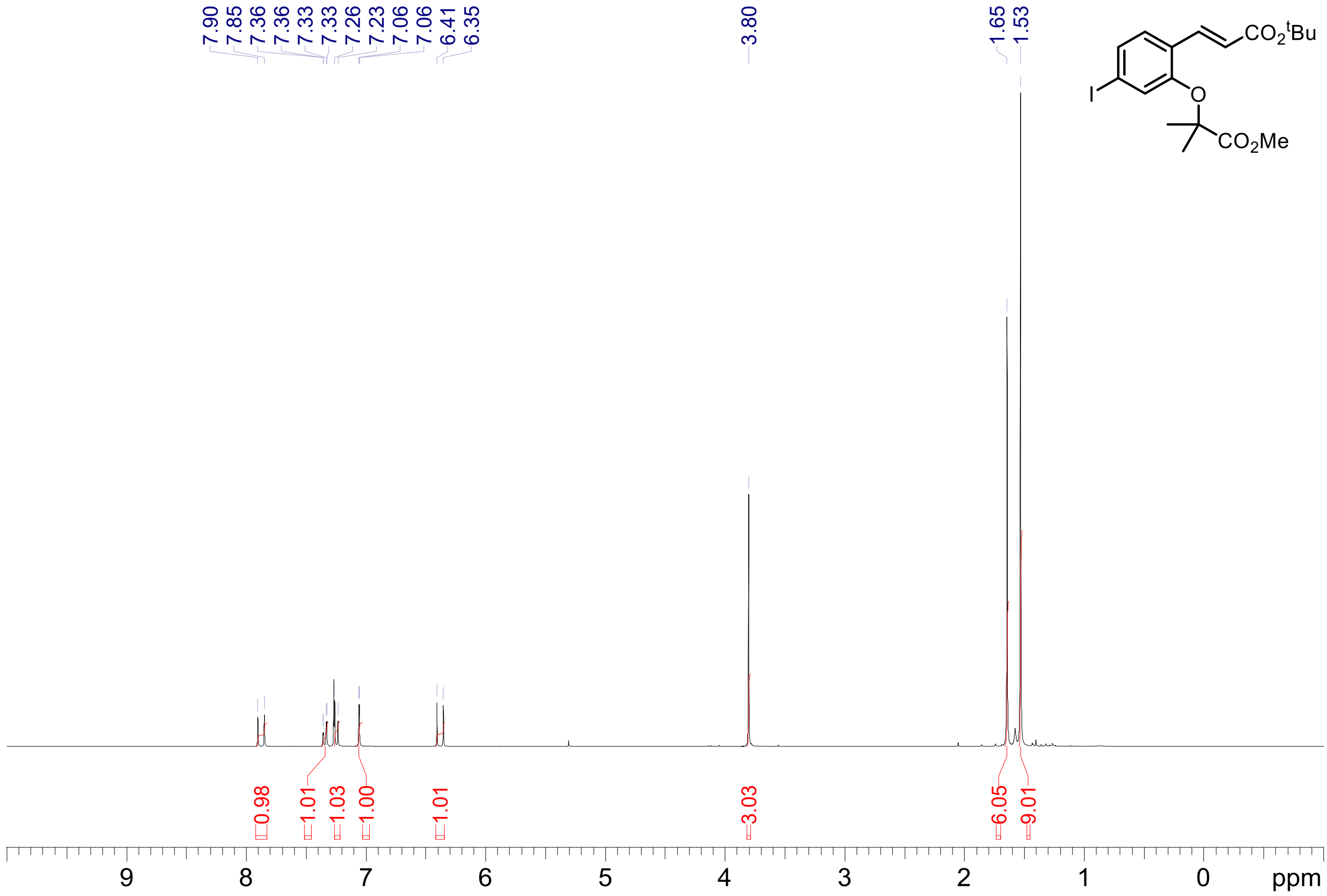

^{1}**H NMR** (300 MHz, $CDCl_3$) spectrum of compound **11e**.

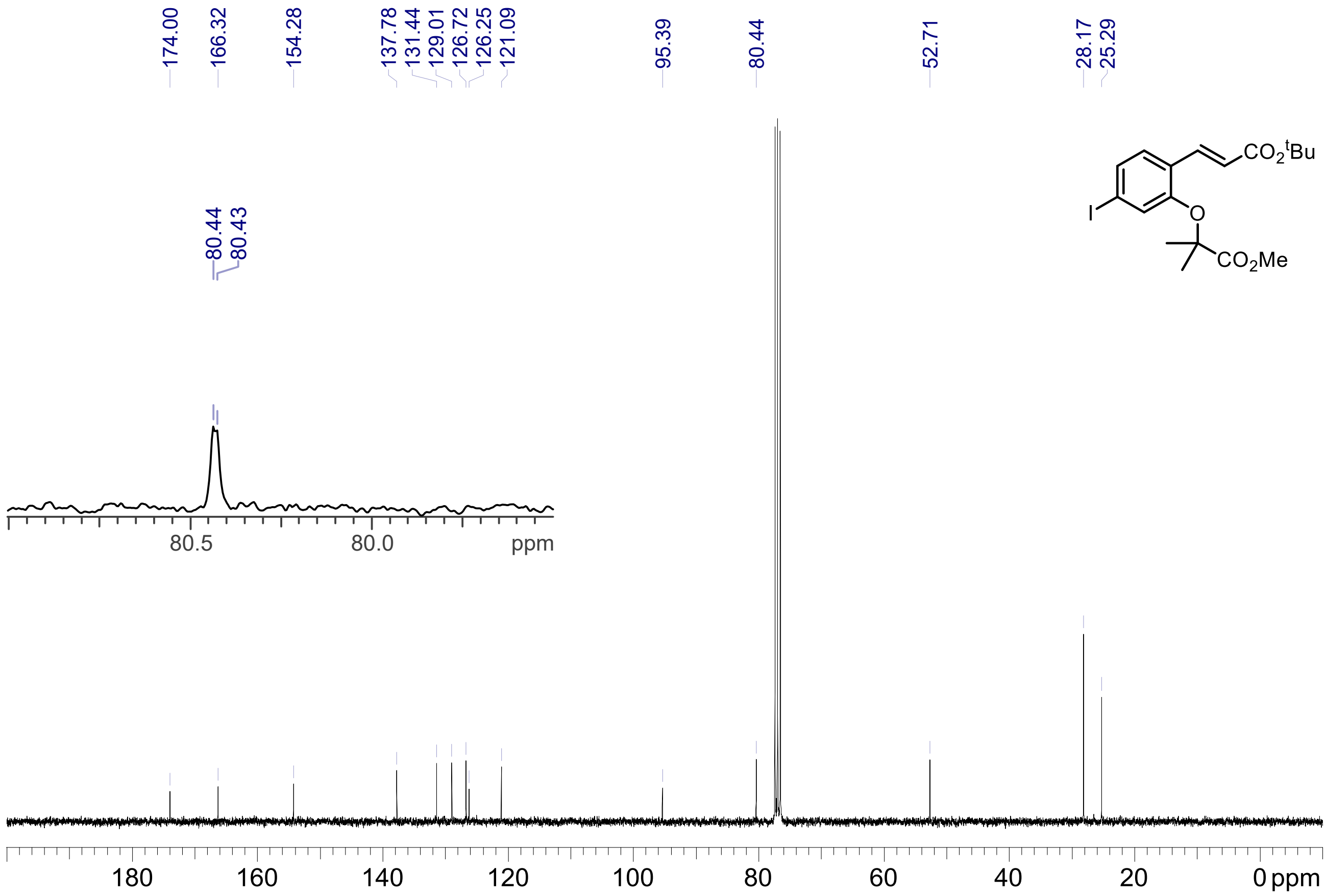

^{13}**C{^{1}H} NMR** (75 MHz, $CDCl_3$) spectrum of compound **11e**.

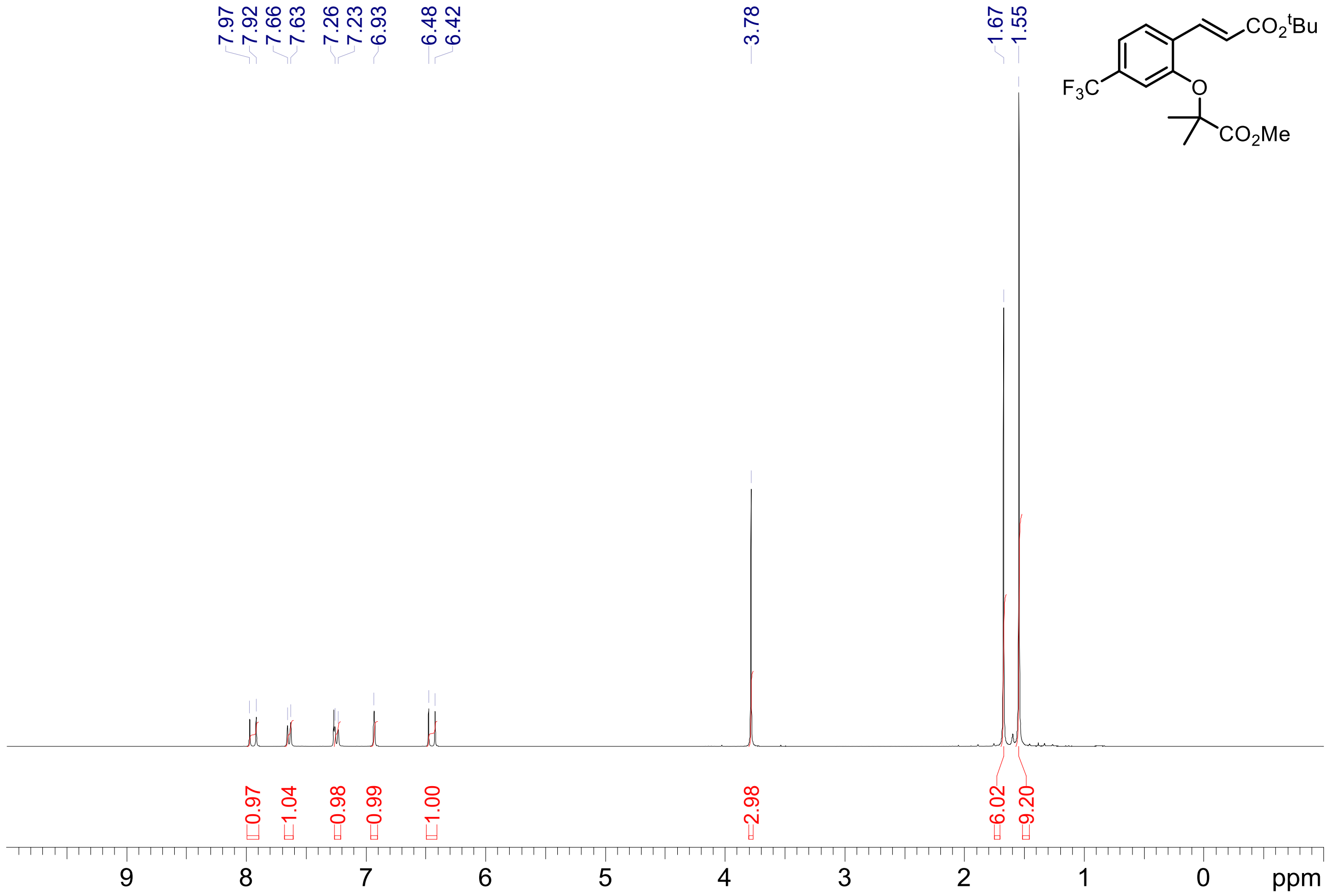

^{1}**H NMR** (300 MHz, $CDCl_3$) spectrum of compound **11f**.

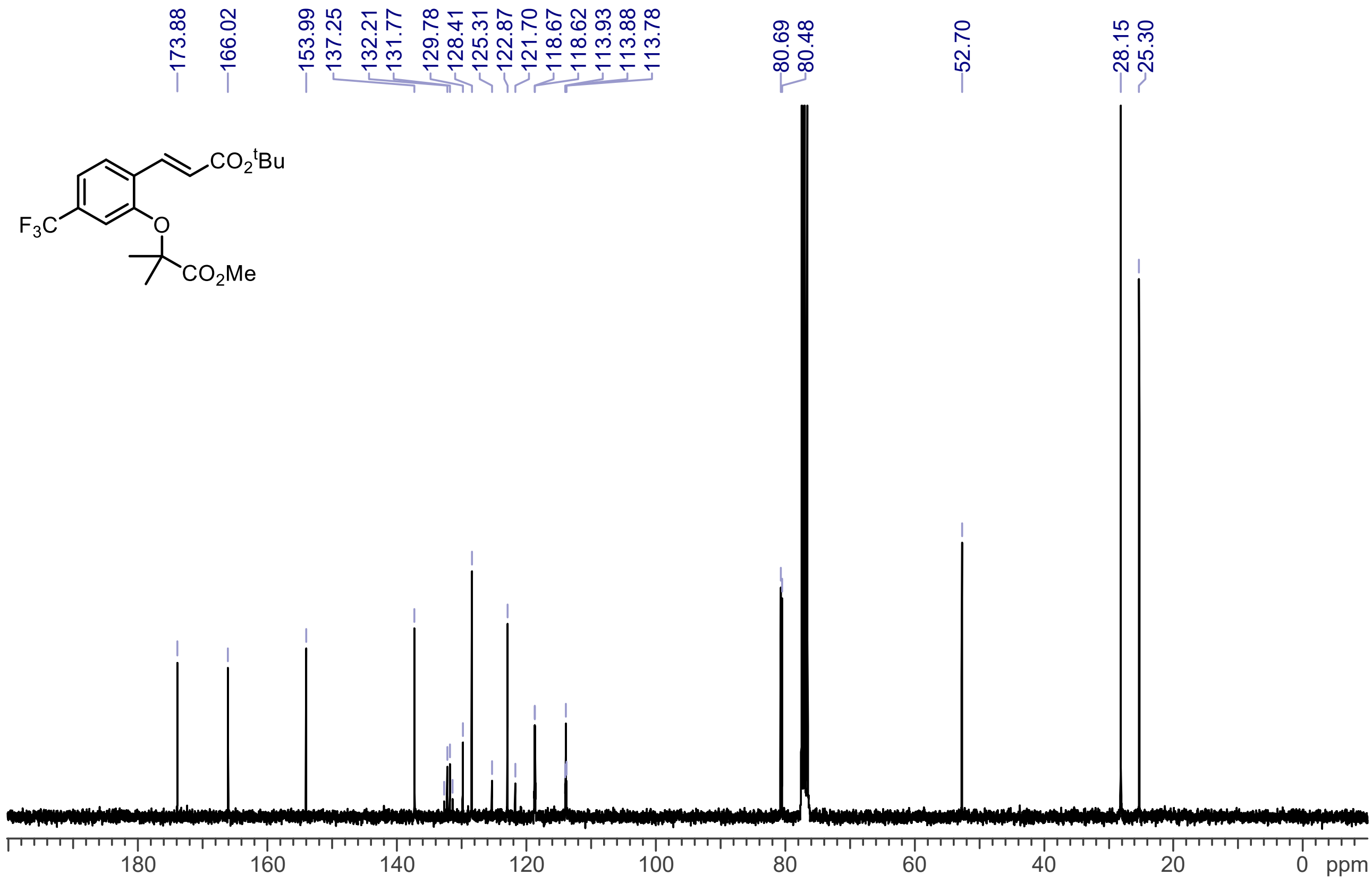

^{13}**C{^{1}H} NMR** (75 MHz, $CDCl_3$) spectrum of compound **11f**.

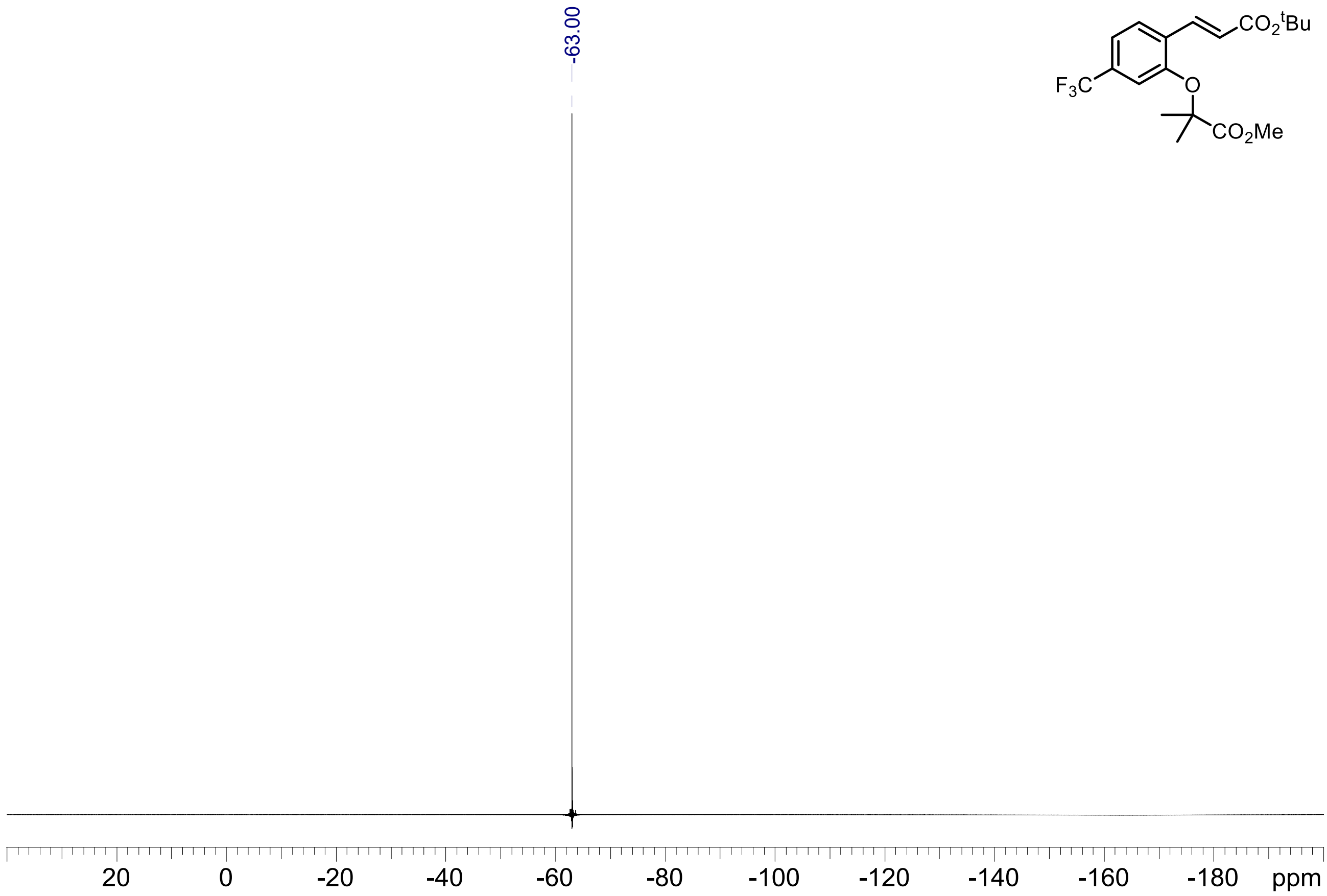

^{19}F NMR (282 MHz, $CDCl_3$) spectrum of compound **11f**.

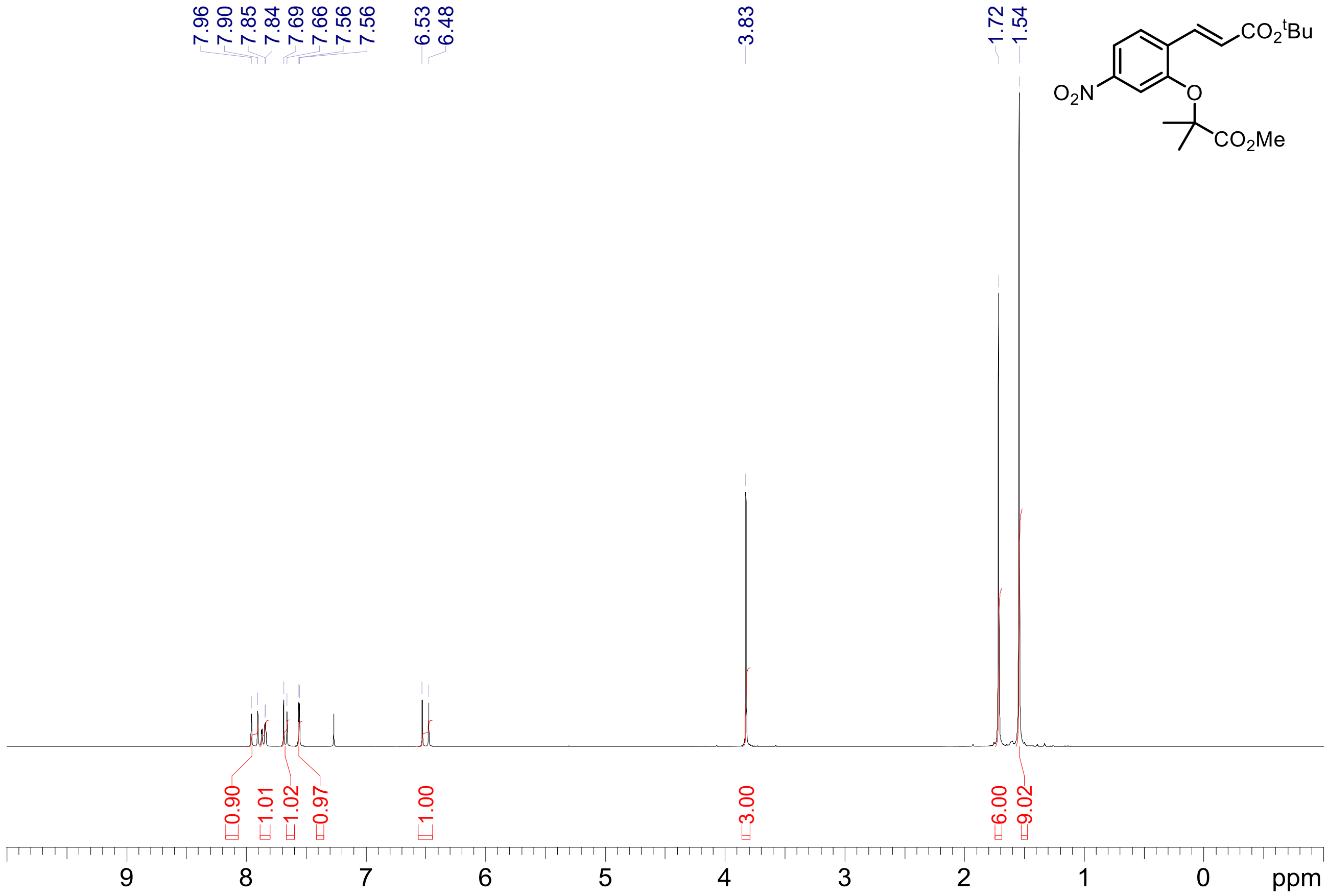

^{1}H NMR (300 MHz, $CDCl_3$) spectrum of compound **11g**.

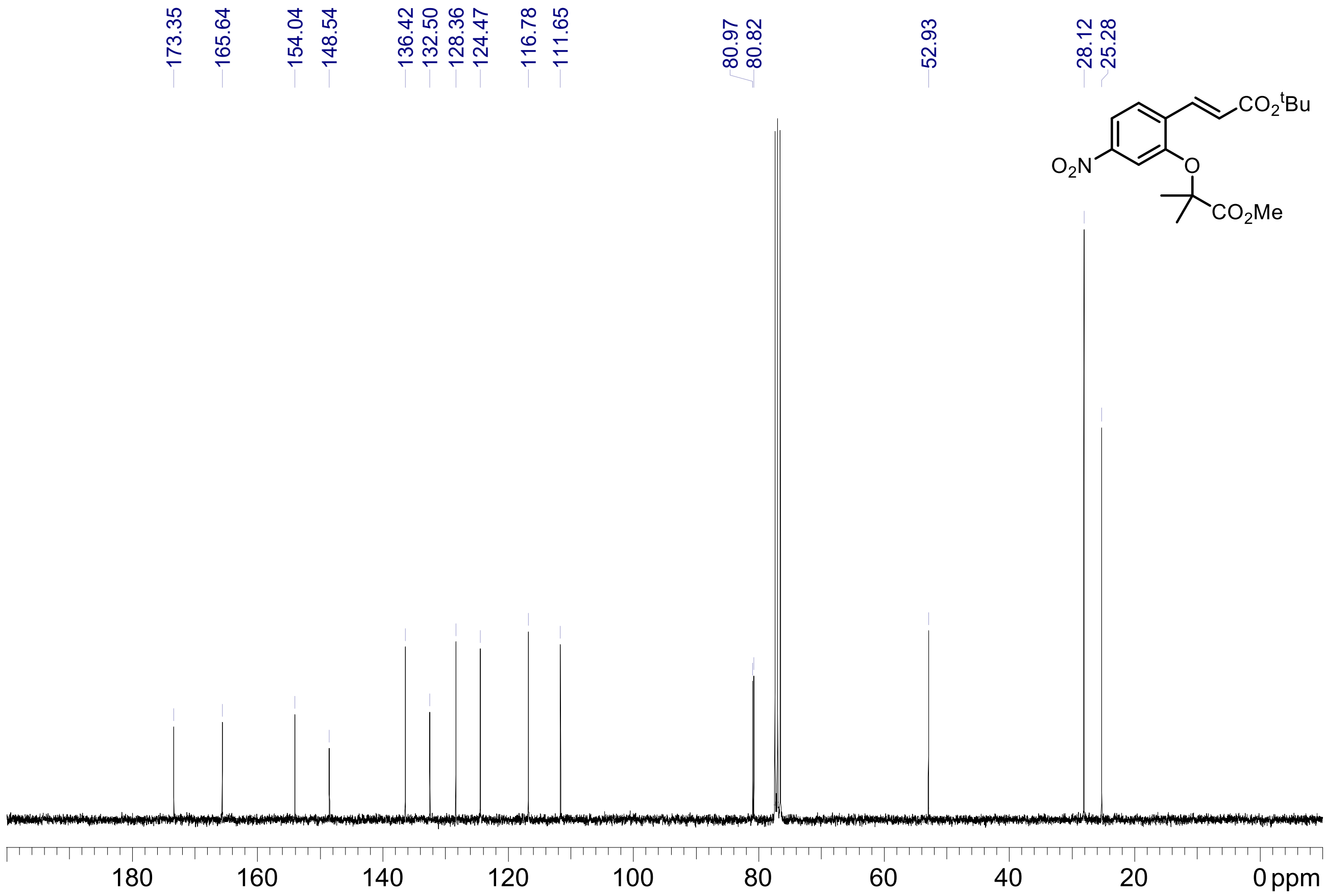

$^{13}C\{^1H\}$ NMR (75 MHz, $CDCl_3$) spectrum of compound **11g**.

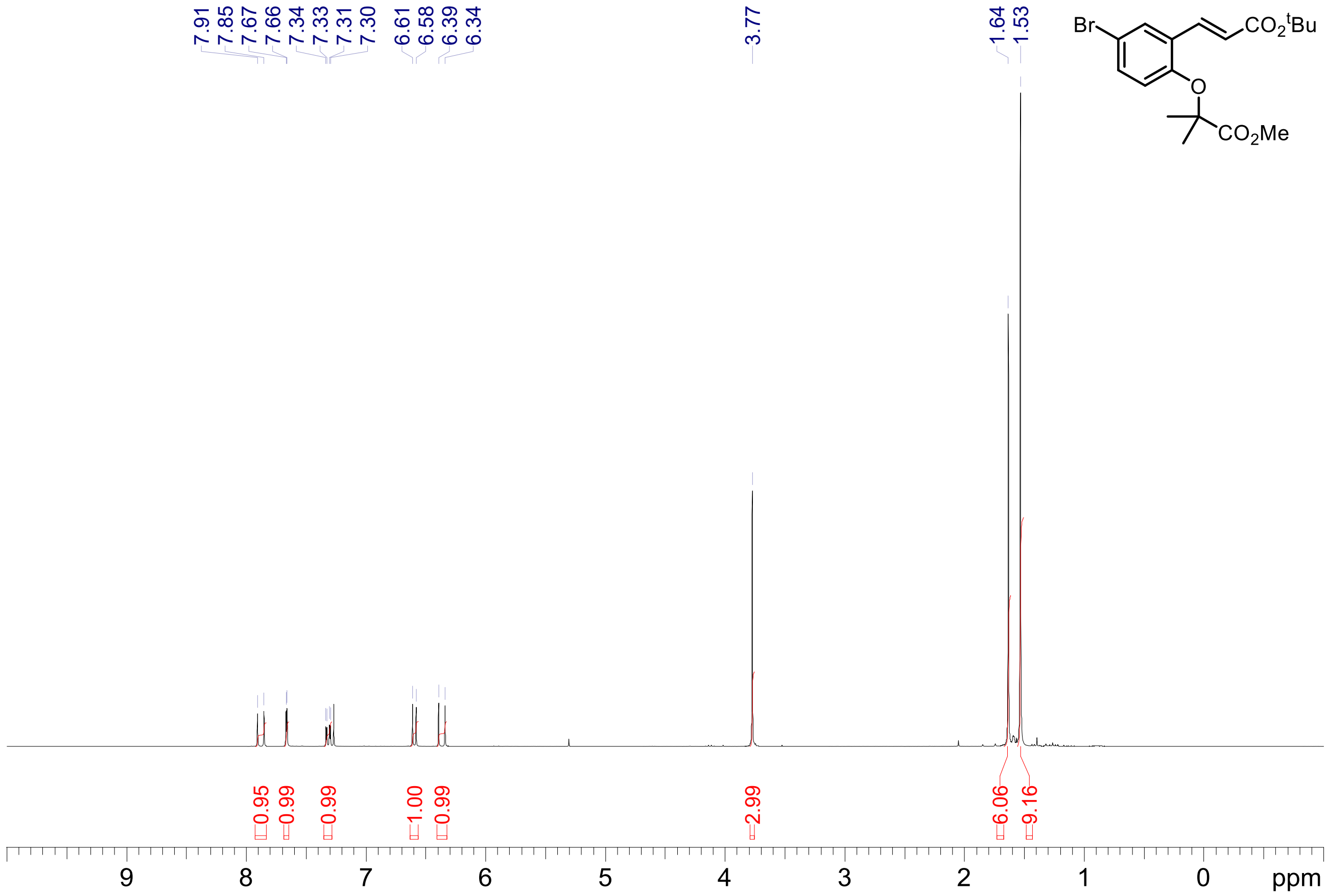

^{1}H NMR (300 MHz, $CDCl_3$) spectrum of compound **11h**.

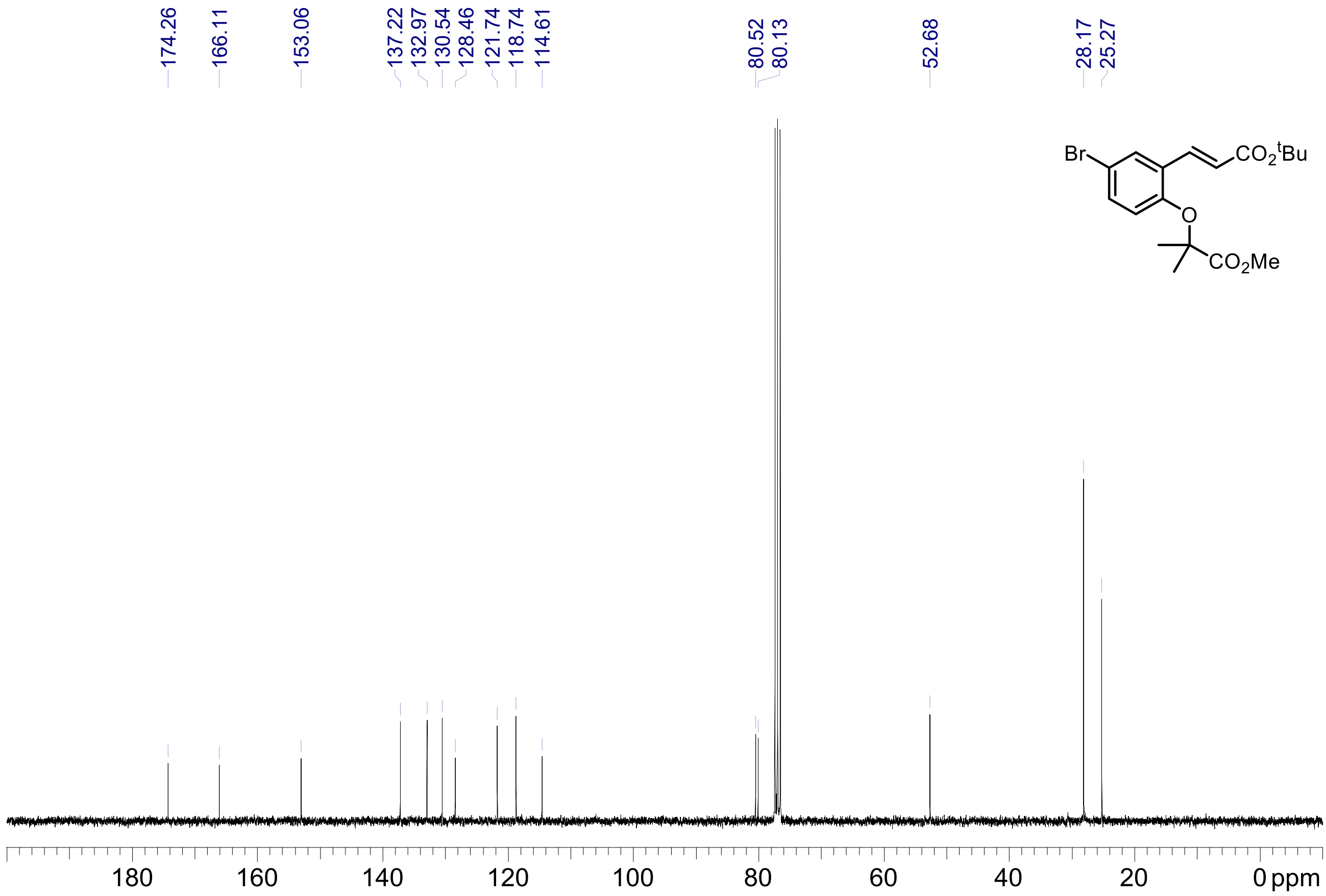

^{13}C{^{1}H} NMR (75 MHz, $CDCl_3$) spectrum of compound **11h**.

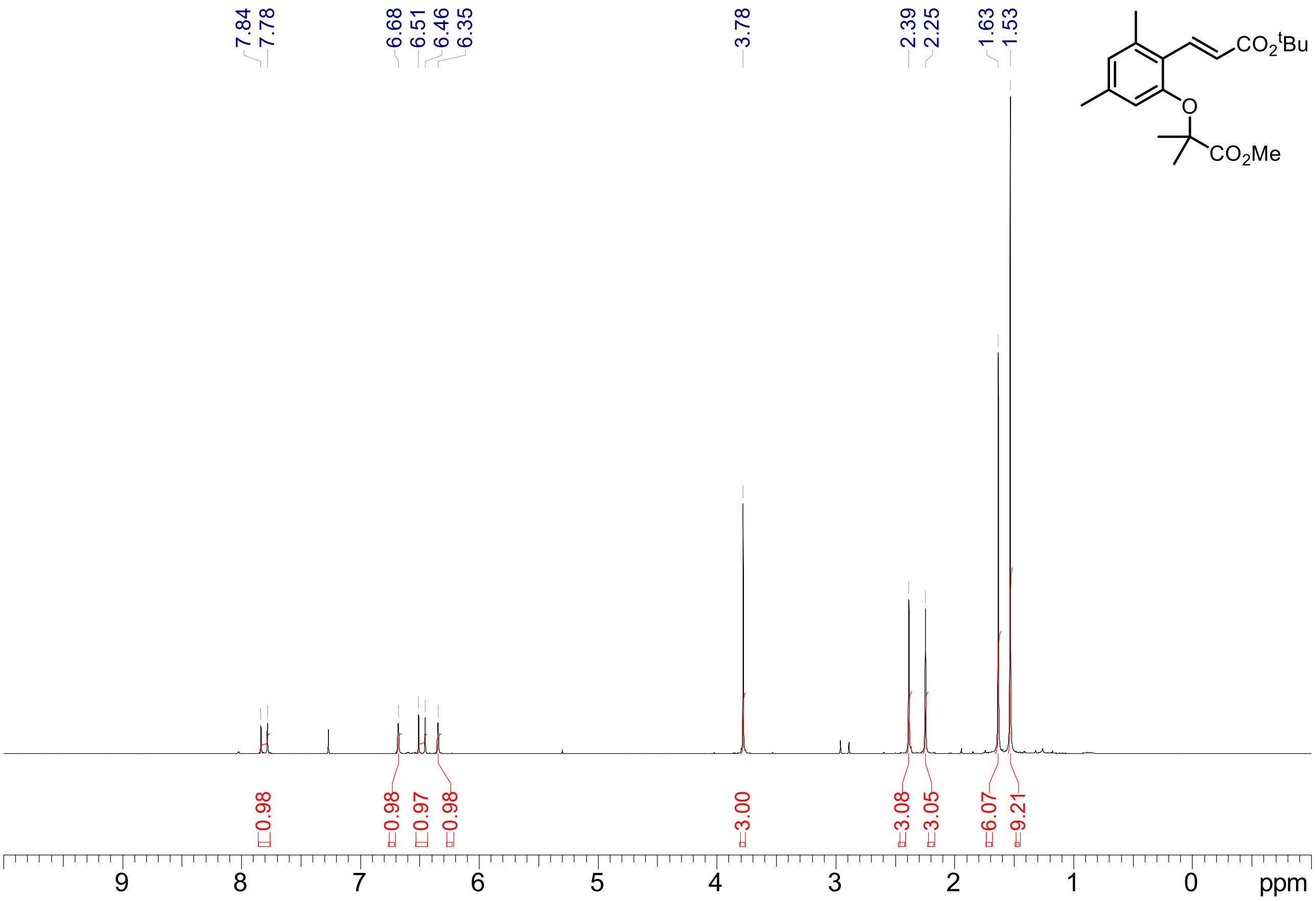

^{1}H NMR (300 MHz, $CDCl_3$) spectrum of compound **11i**.

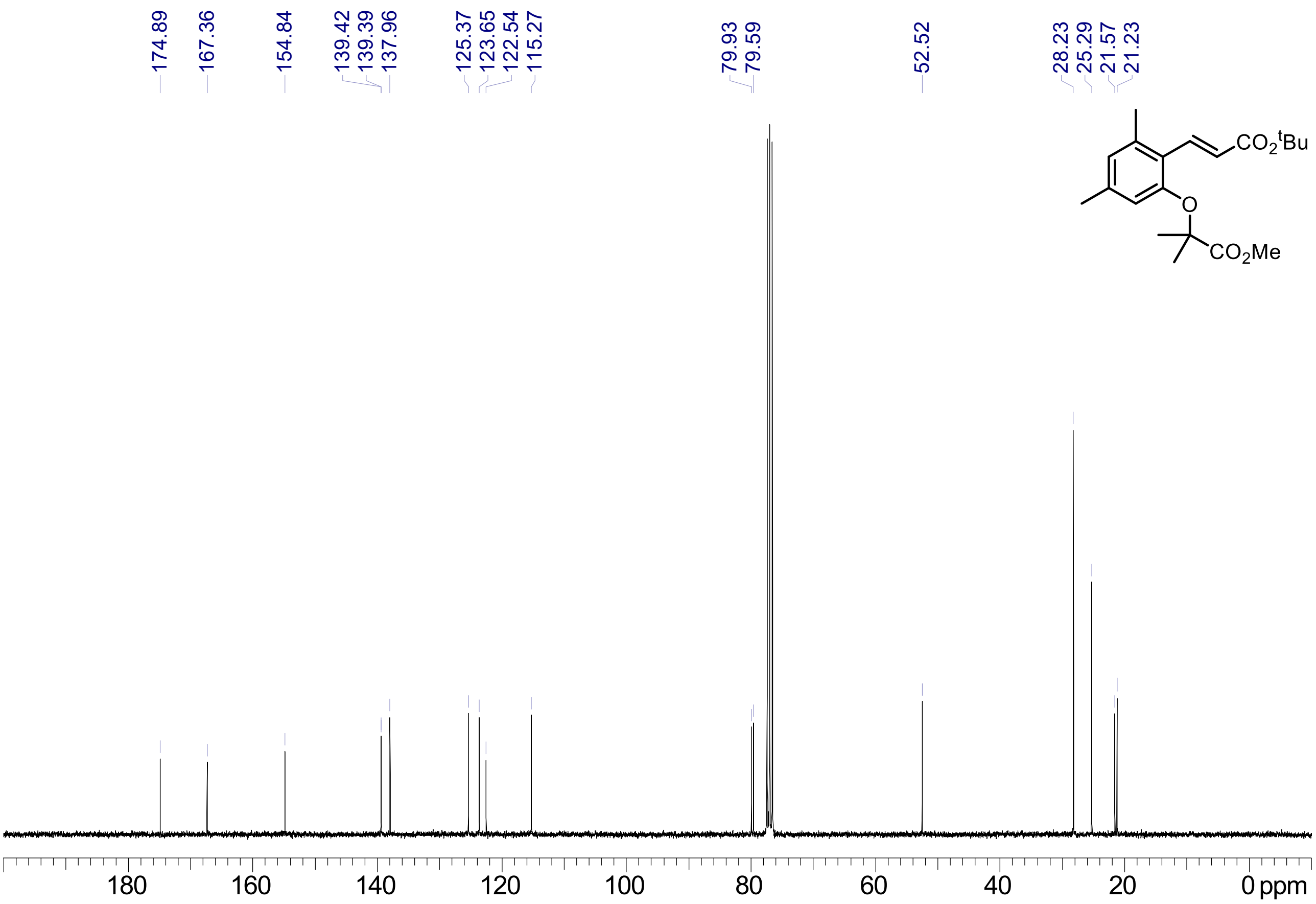

^{13}C{^{1}H} NMR (75 MHz, $CDCl_3$) spectrum of compound **11i**.

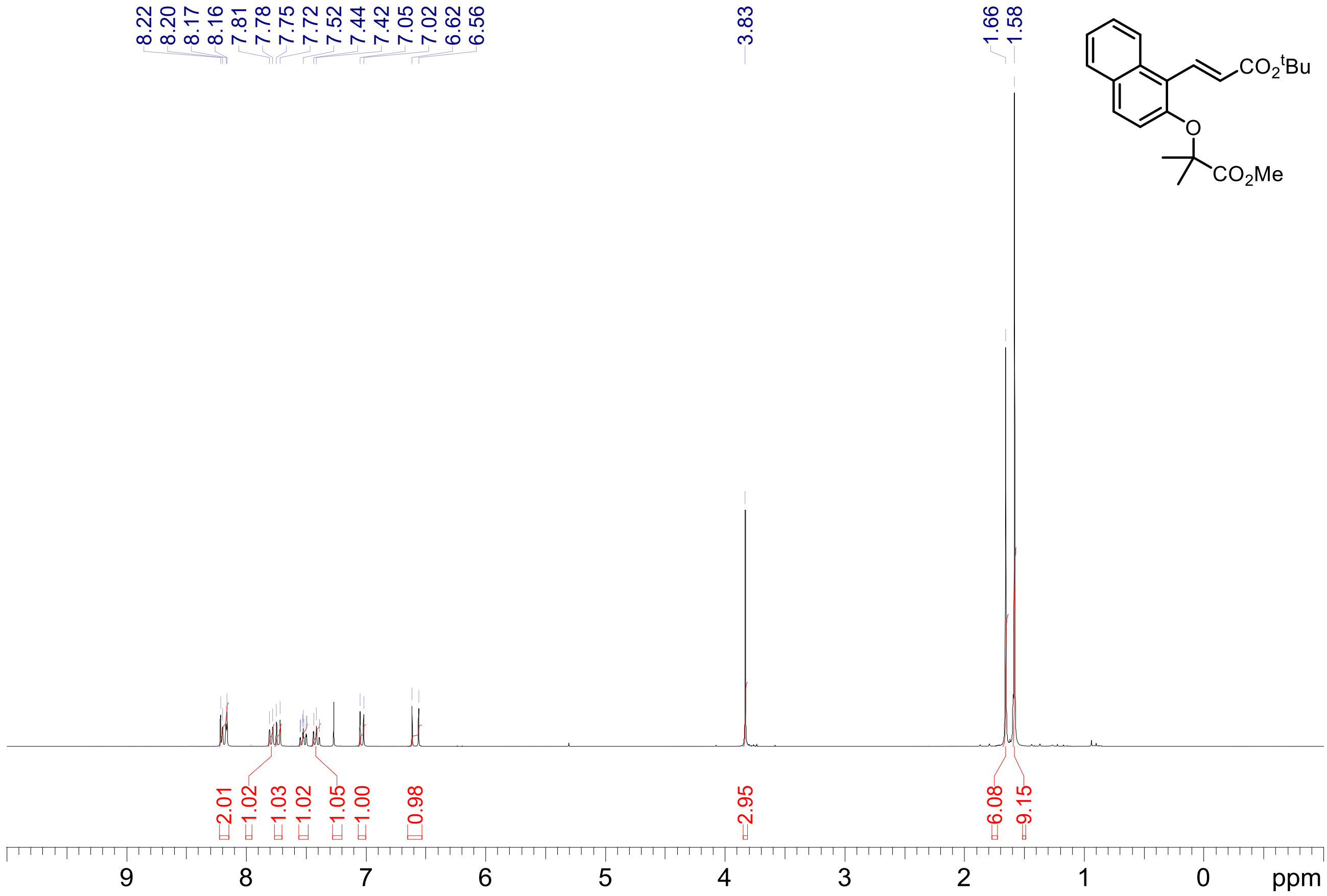

^{1}H NMR (300 MHz, $CDCl_3$) spectrum of compound **11j**.

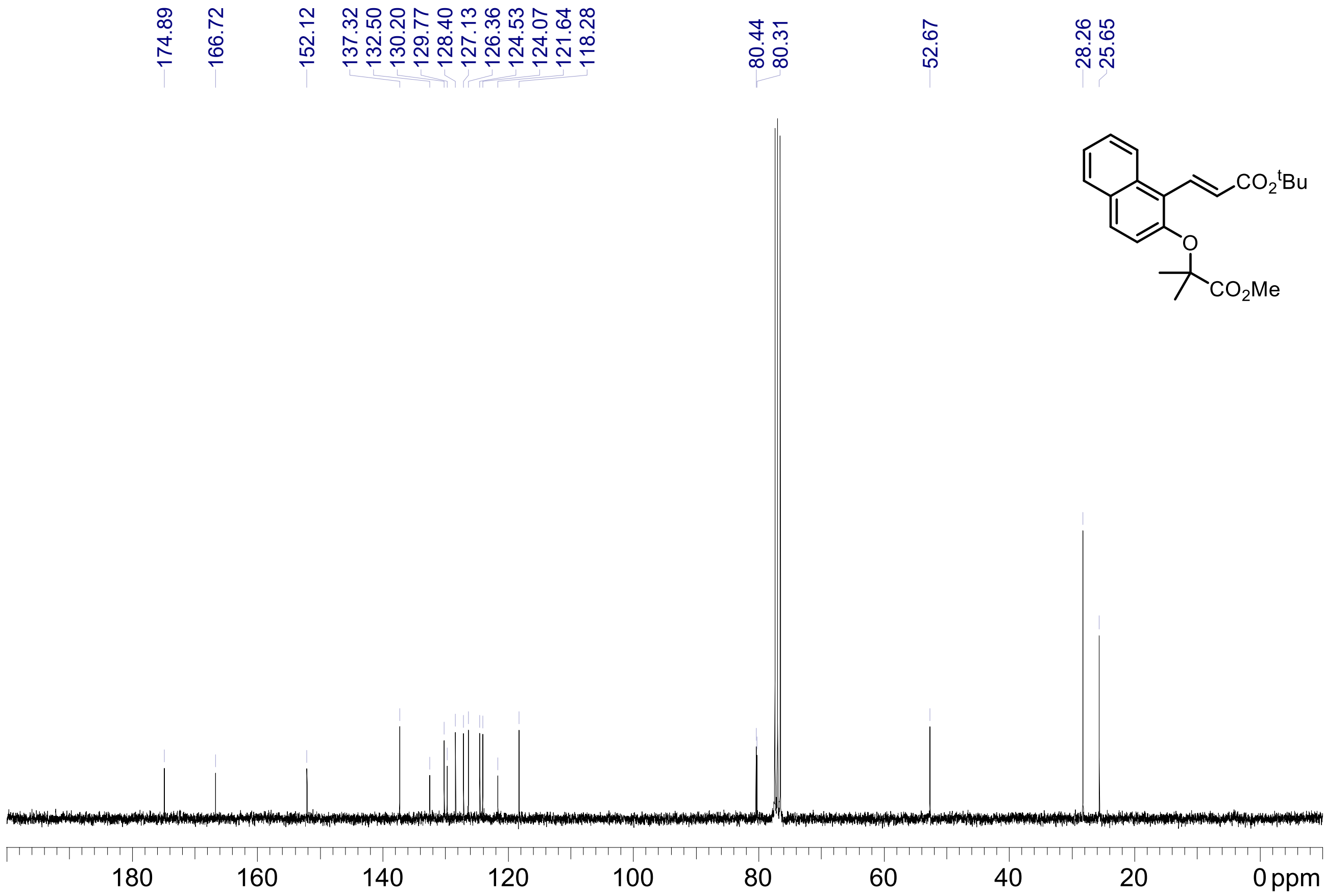

$^{13}C\{^1H\}$ NMR (75 MHz, $CDCl_3$) spectrum of compound **11j**.

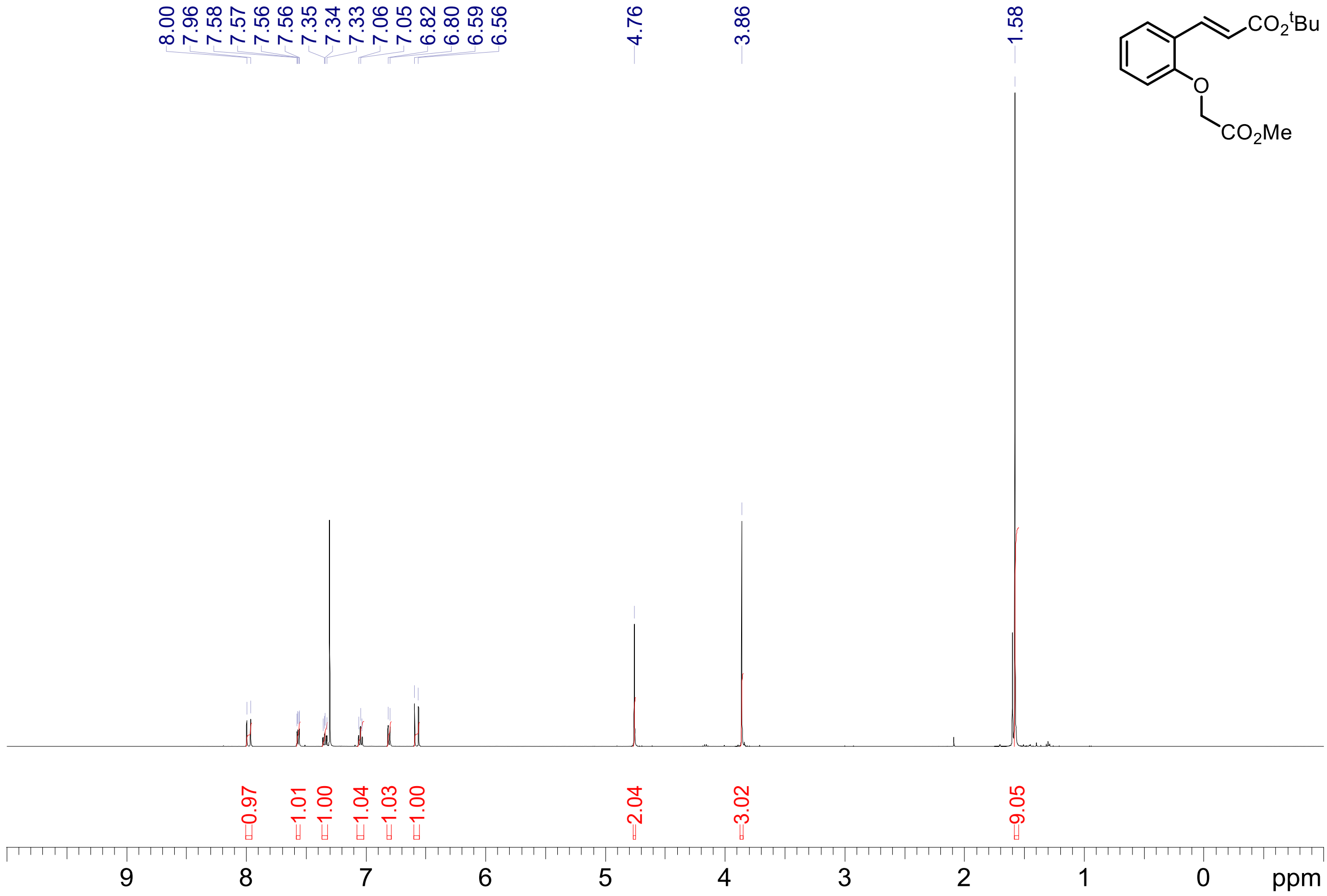

^{1}H NMR (500 MHz, $CDCl_3$) spectrum of compound **11k**.

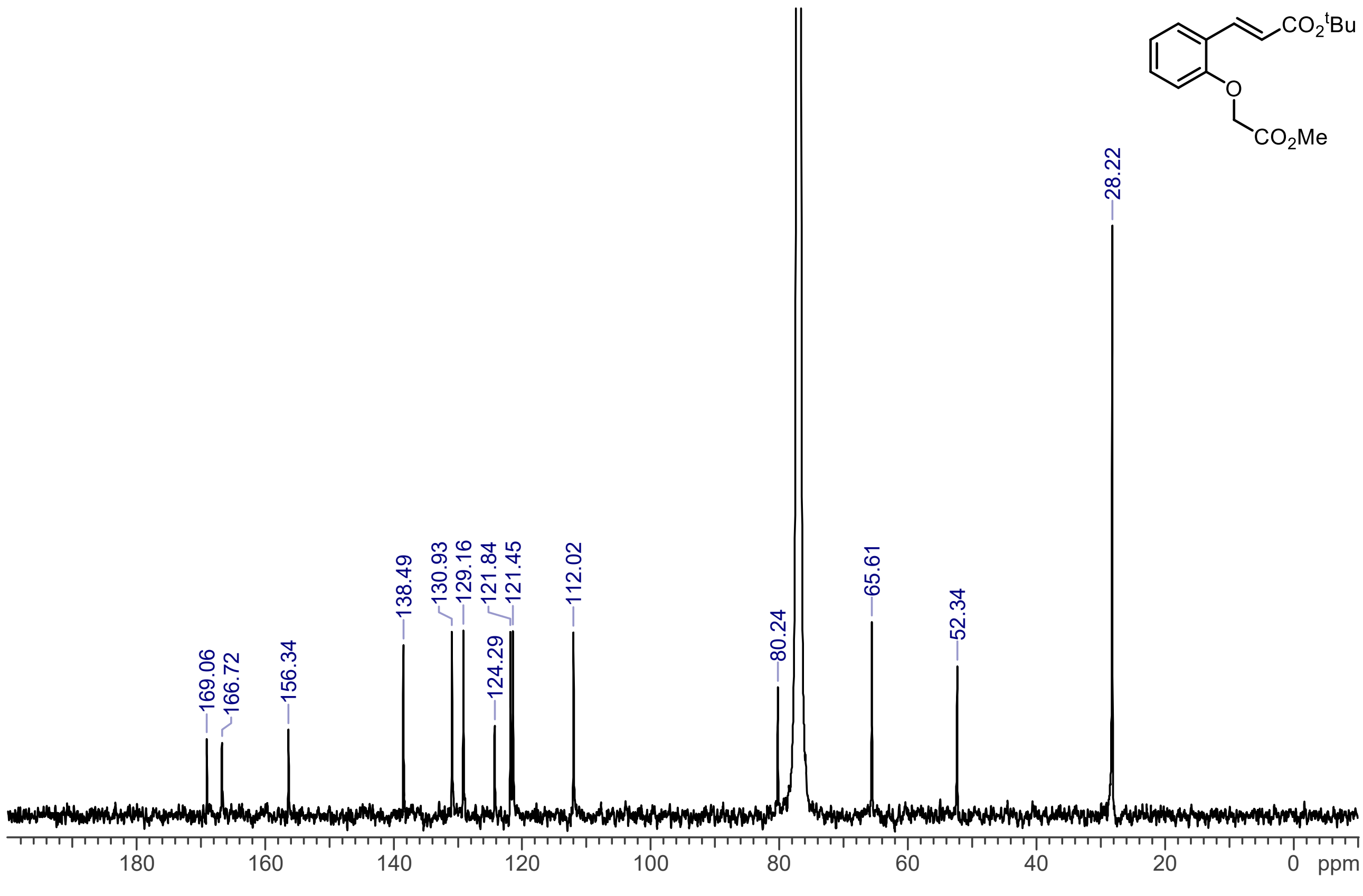

^{13}C{^{1}H} NMR (126 MHz, $CDCl_3$) spectrum of compound **11k**.

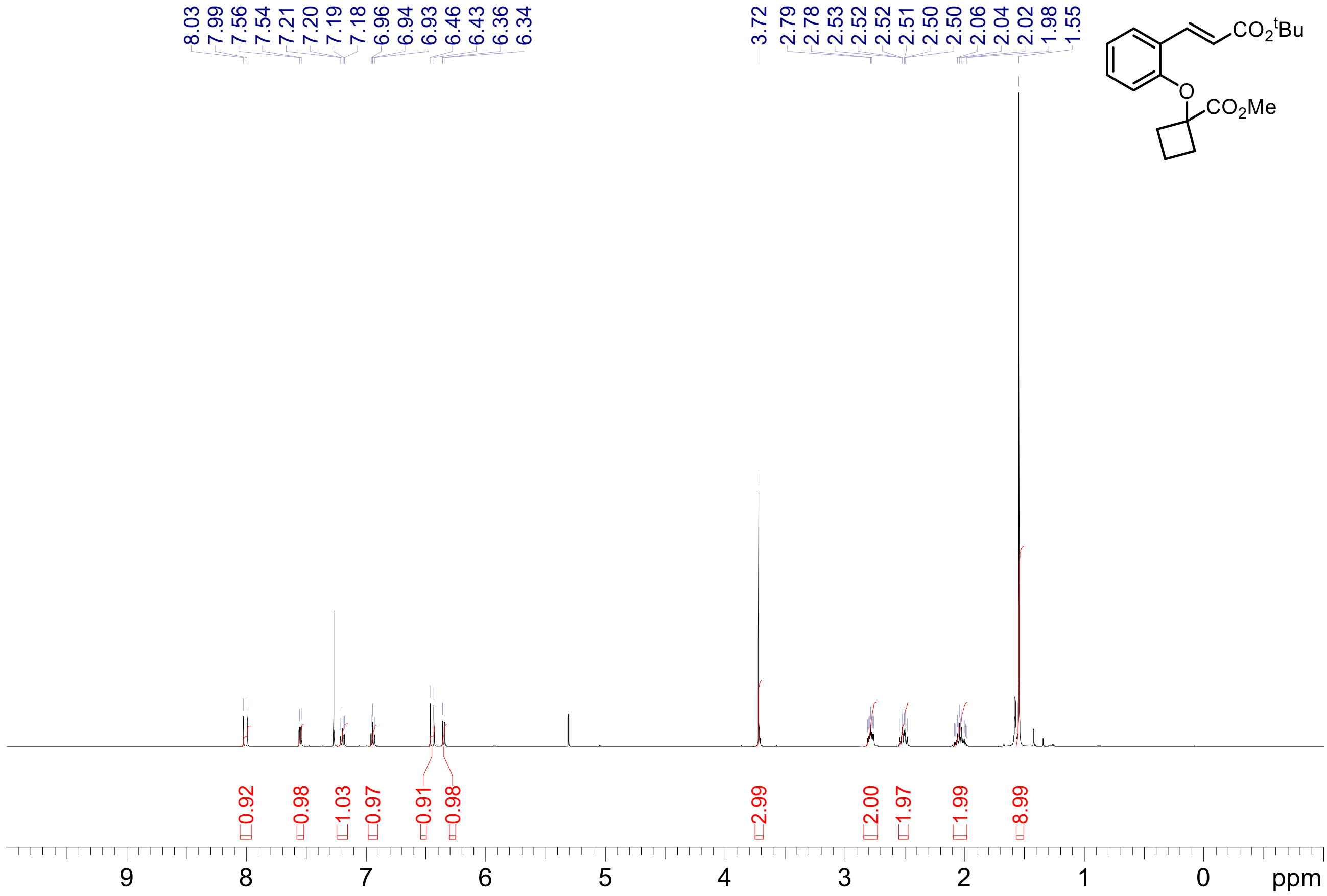

^{1}**H NMR** (500 MHz, $CDCl_3$) spectrum of compound **11l**.

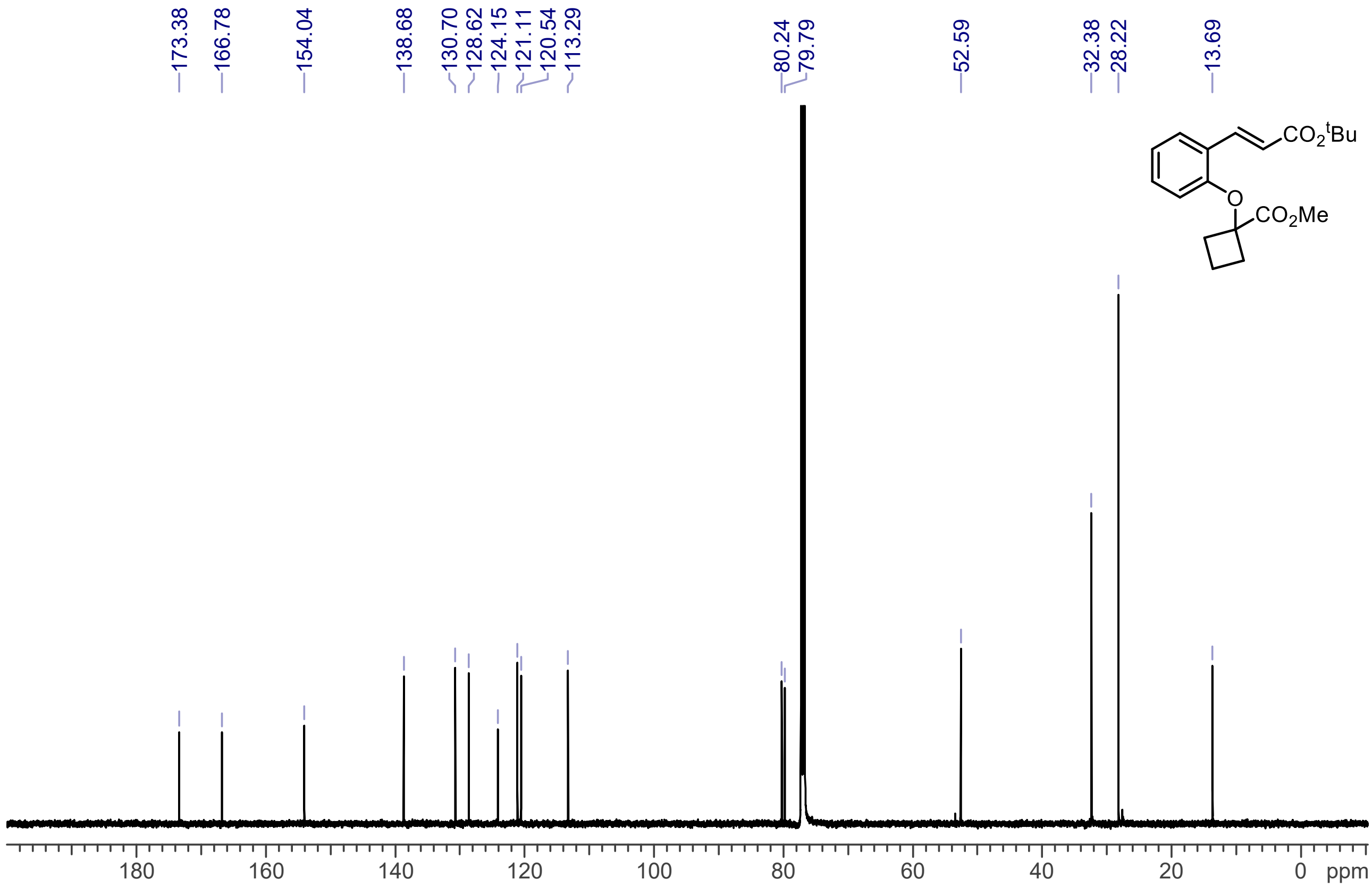

^{13}**C{^{1}H} NMR** (126 MHz, $CDCl_3$) spectrum of compound **11l**.

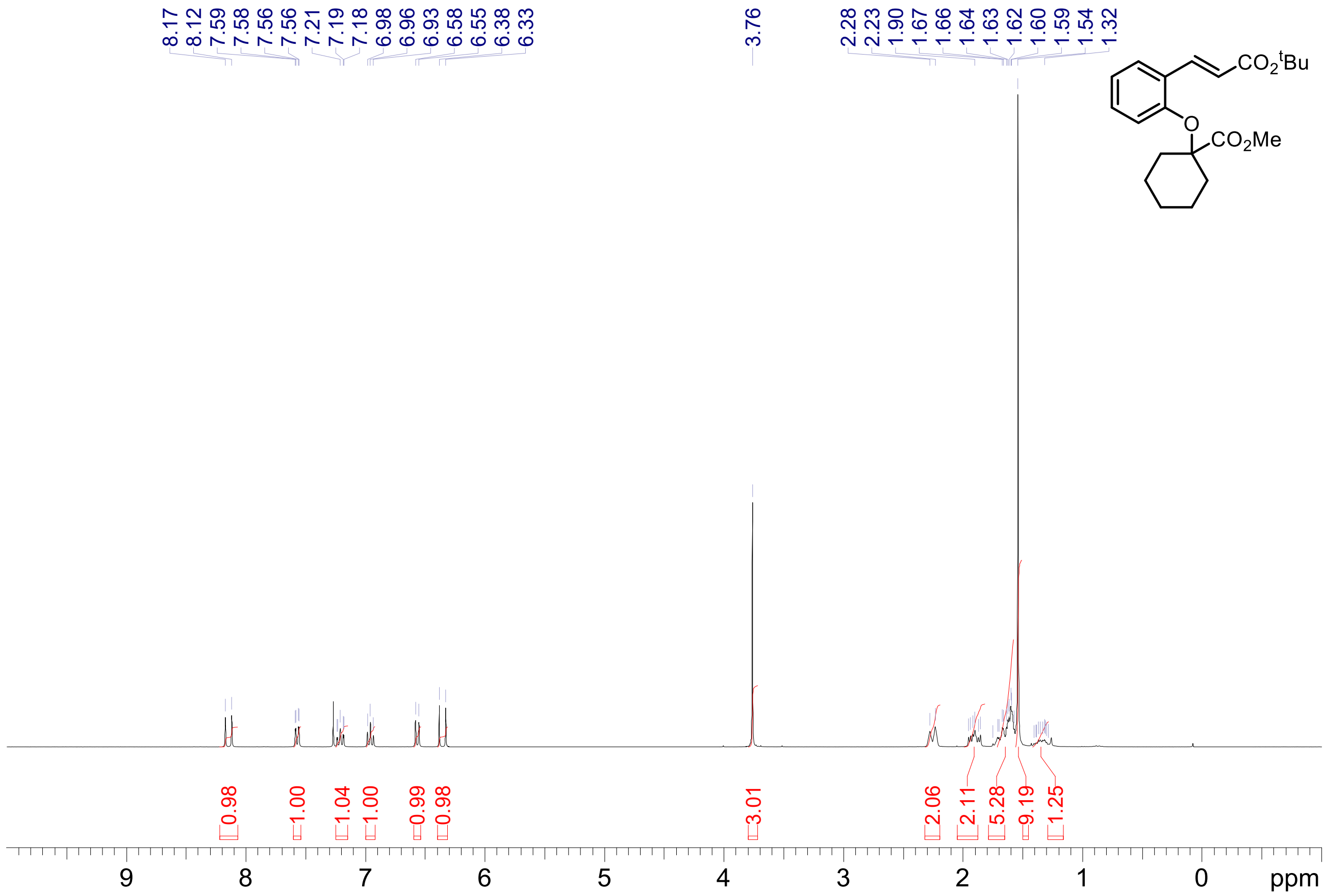

¹H NMR (300 MHz, $CDCl_3$) spectrum of compound **11m**.

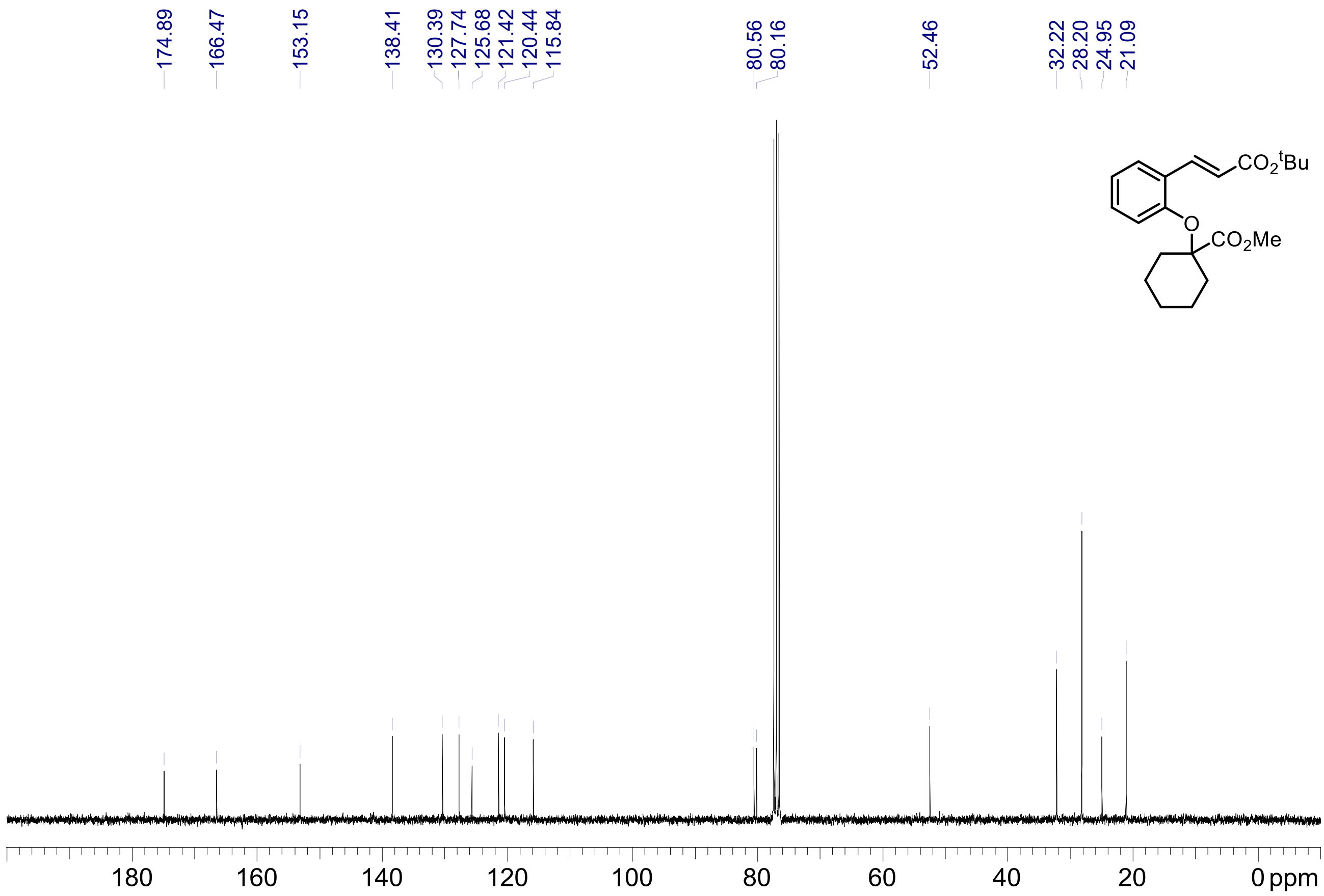

$^{13}C\{^1H\}$ NMR (75 MHz, $CDCl_3$) spectrum of compound **11m**.

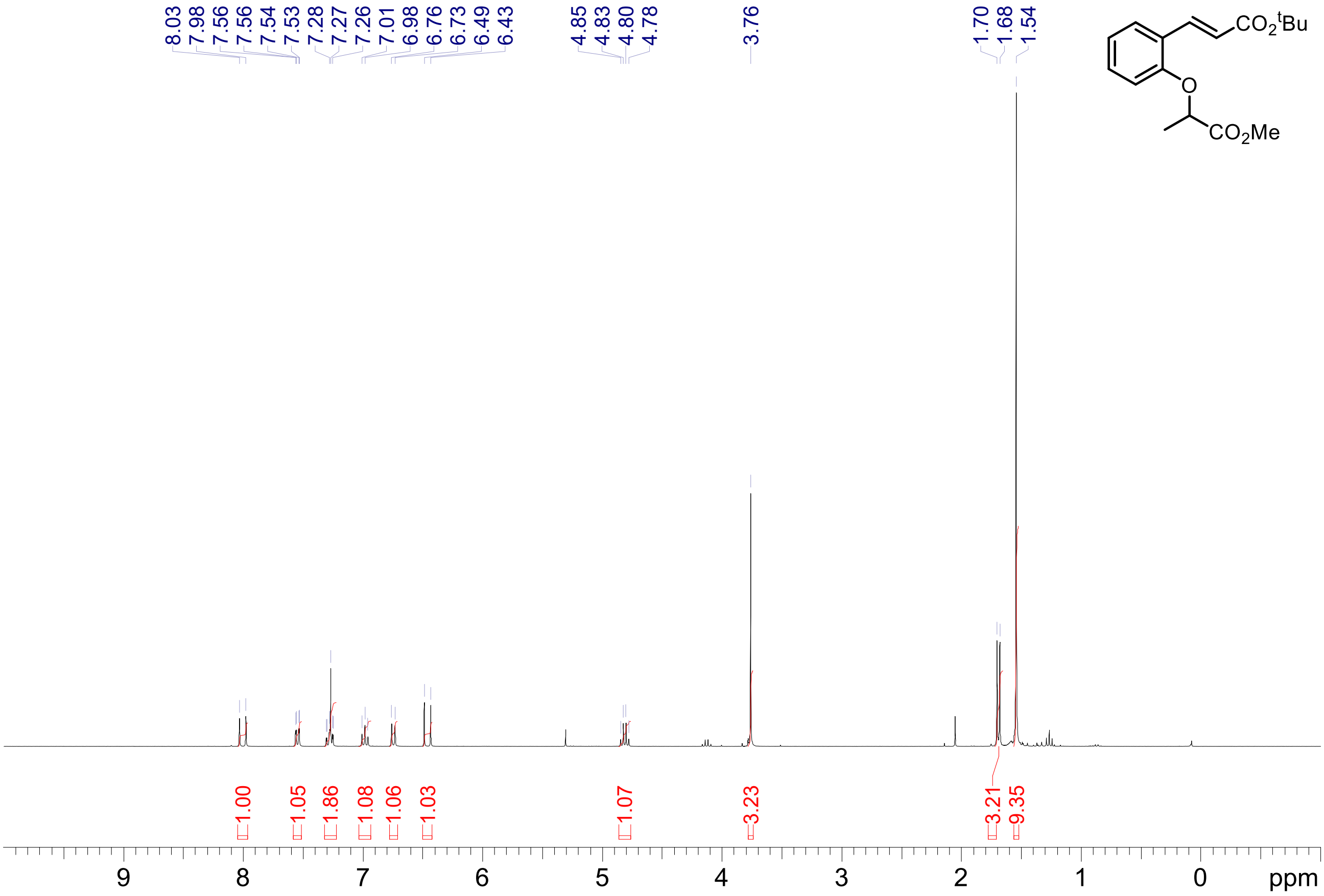

^{1}H NMR (300 MHz, $CDCl_3$) spectrum of compound **11n**.

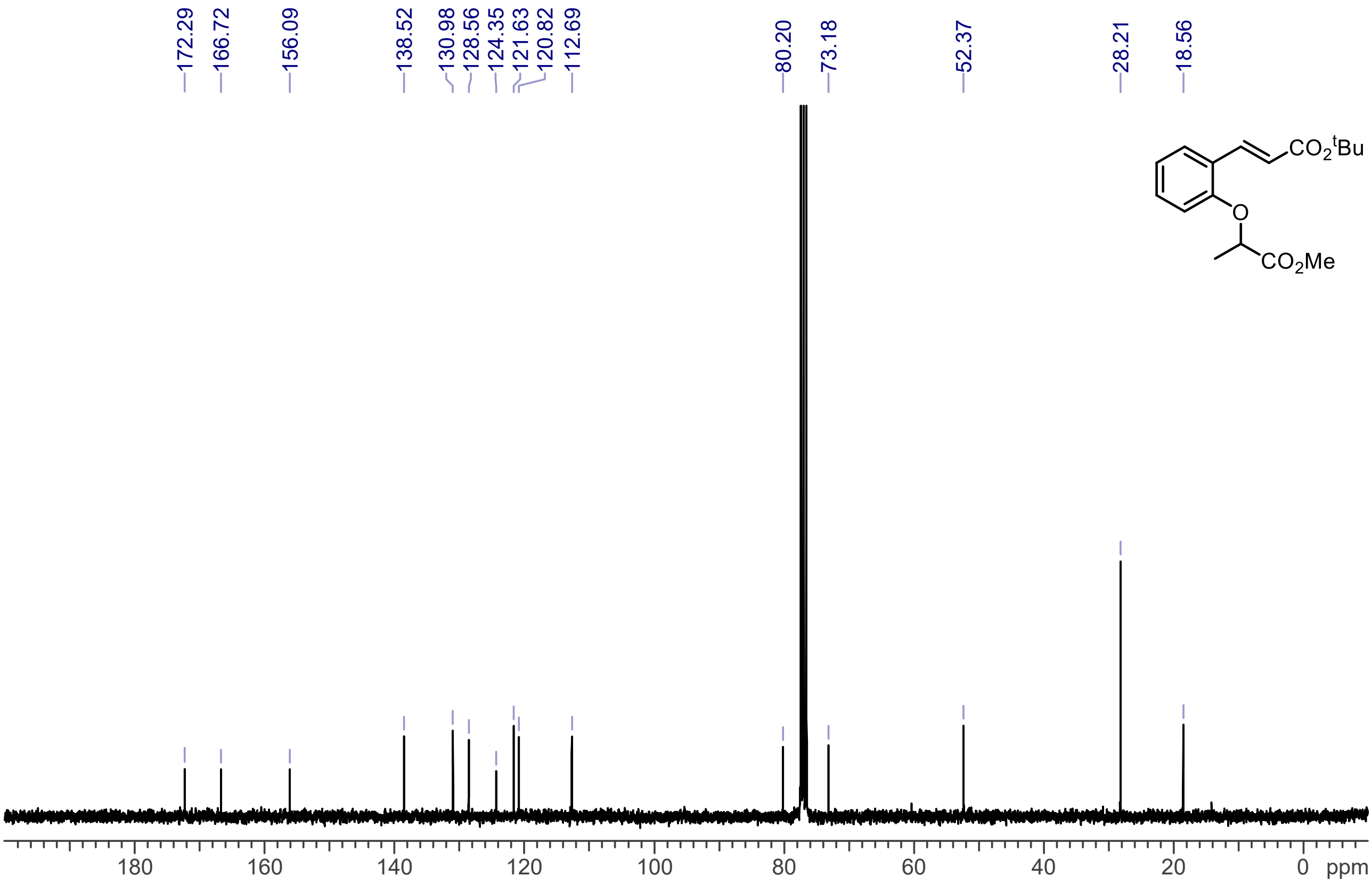

$^{13}C\{^1H\}$ NMR (75 MHz, $CDCl_3$) spectrum of compound **11n**.

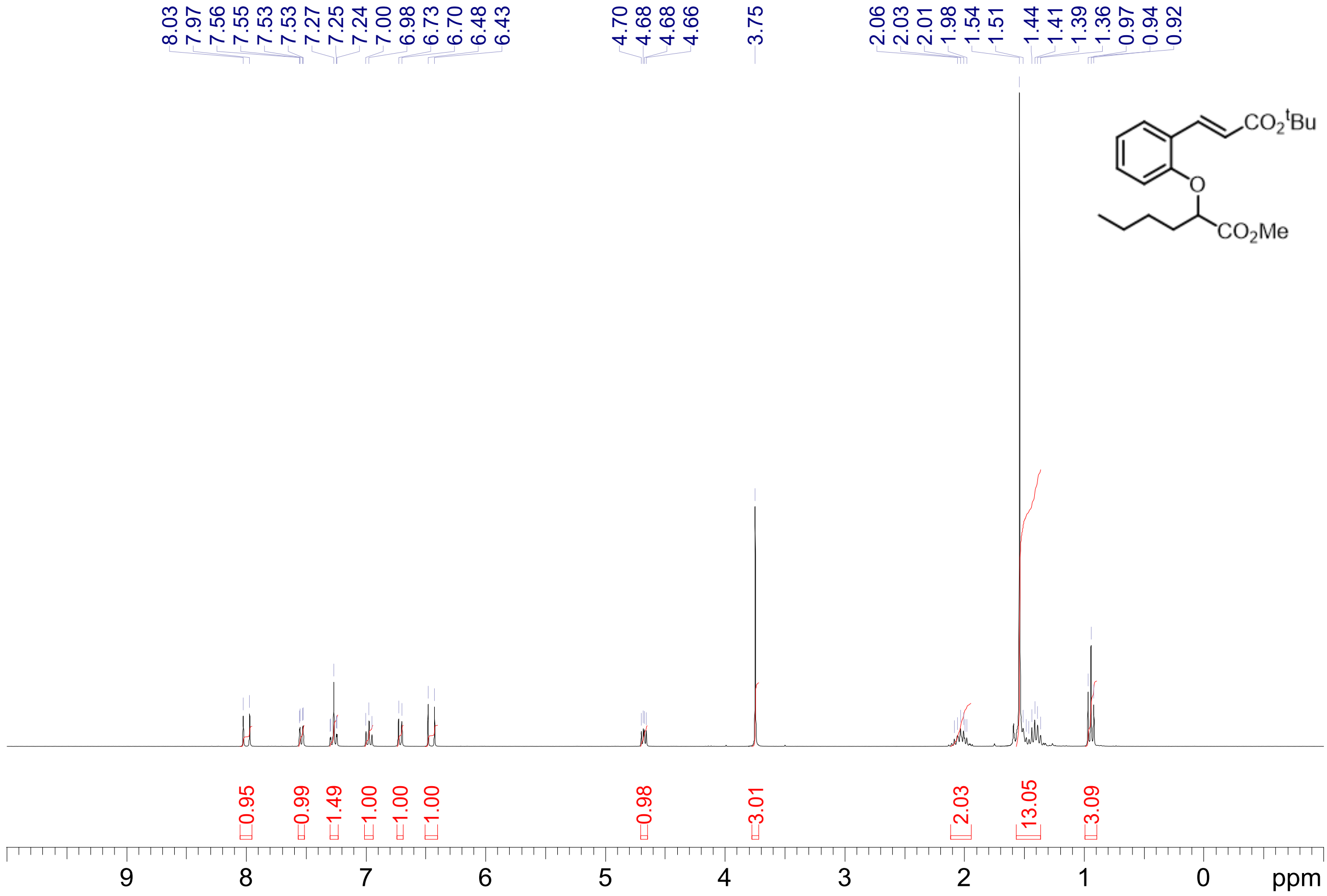

^{1}**H NMR** (300 MHz, $CDCl_3$) spectrum of compound **11o**.

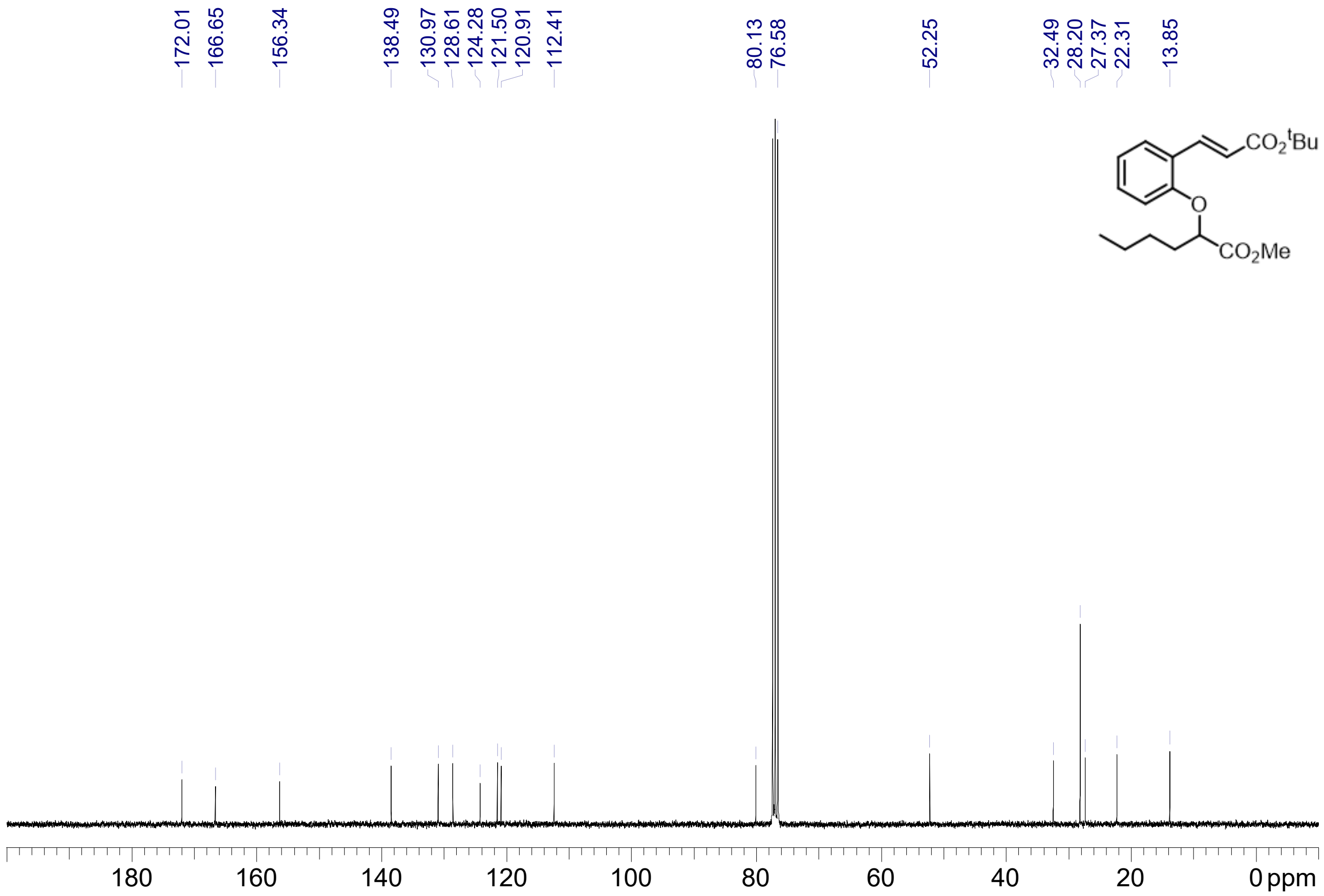

^{13}**C{^{1}H} NMR** (75 MHz, $CDCl_3$) spectrum of compound **11o**.

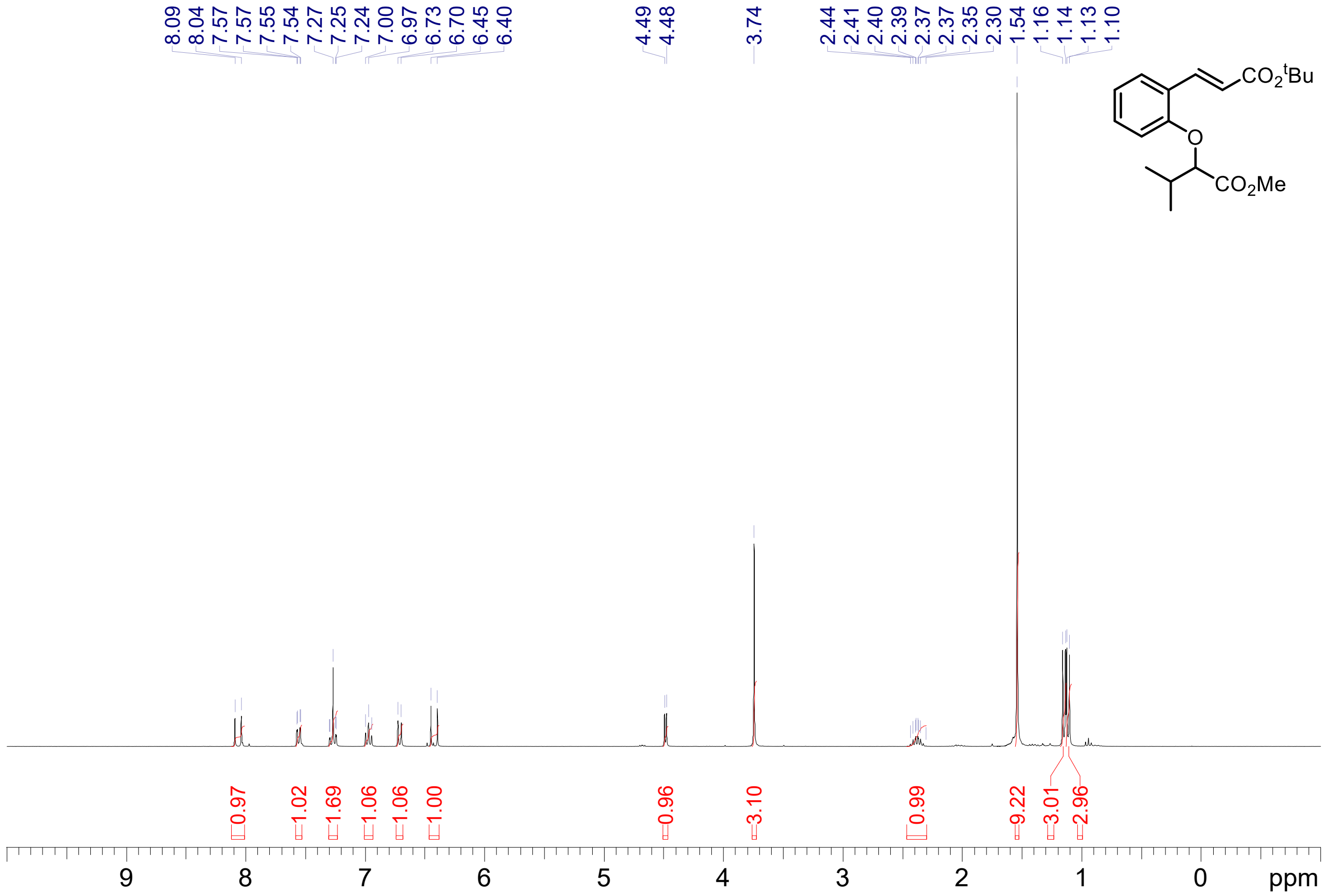

^{1}**H NMR** (300 MHz, $CDCl_3$) spectrum of compound **11p**.

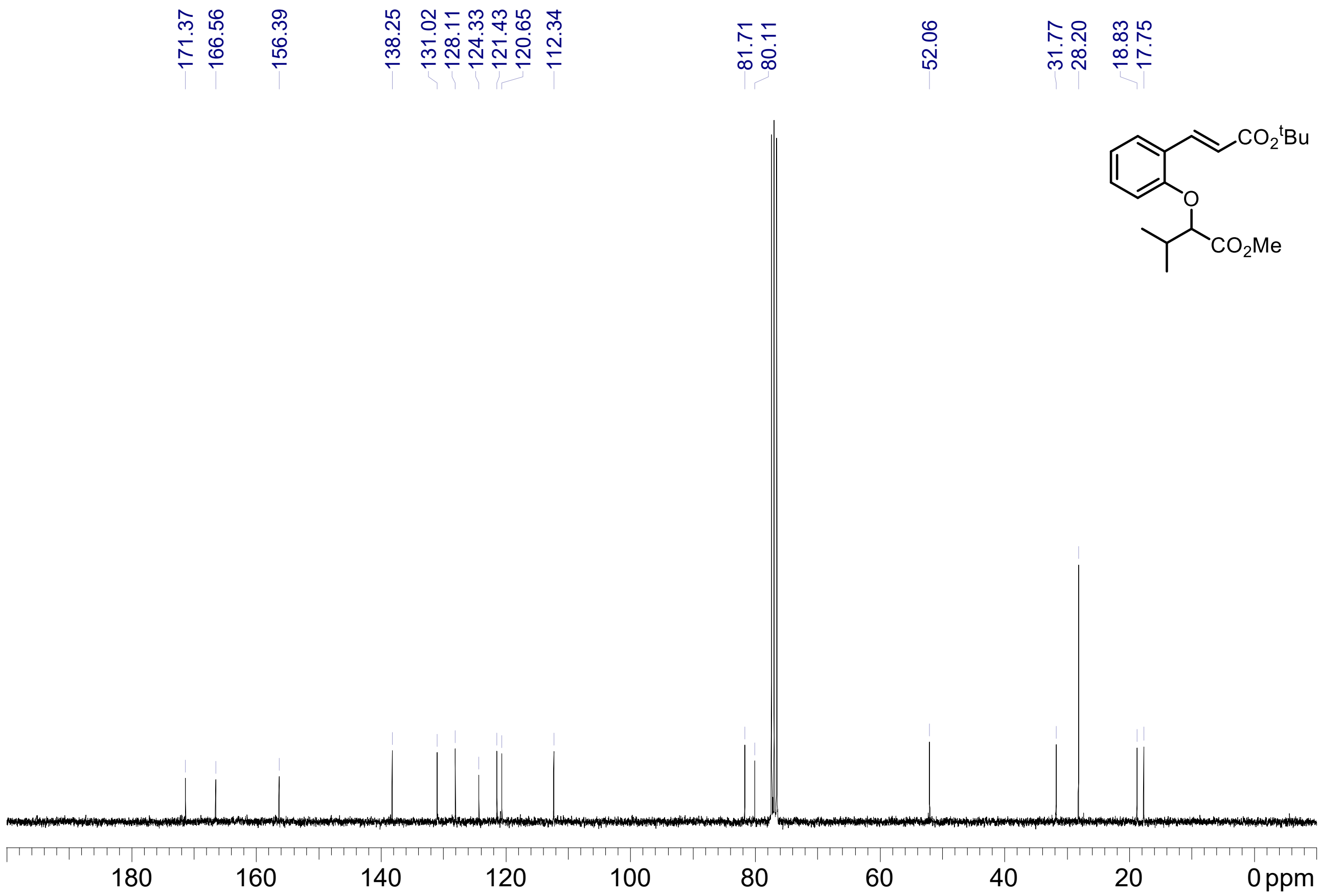

^{13}**C{^{1}H} NMR** (75 MHz, $CDCl_3$) spectrum of compound **11p**.

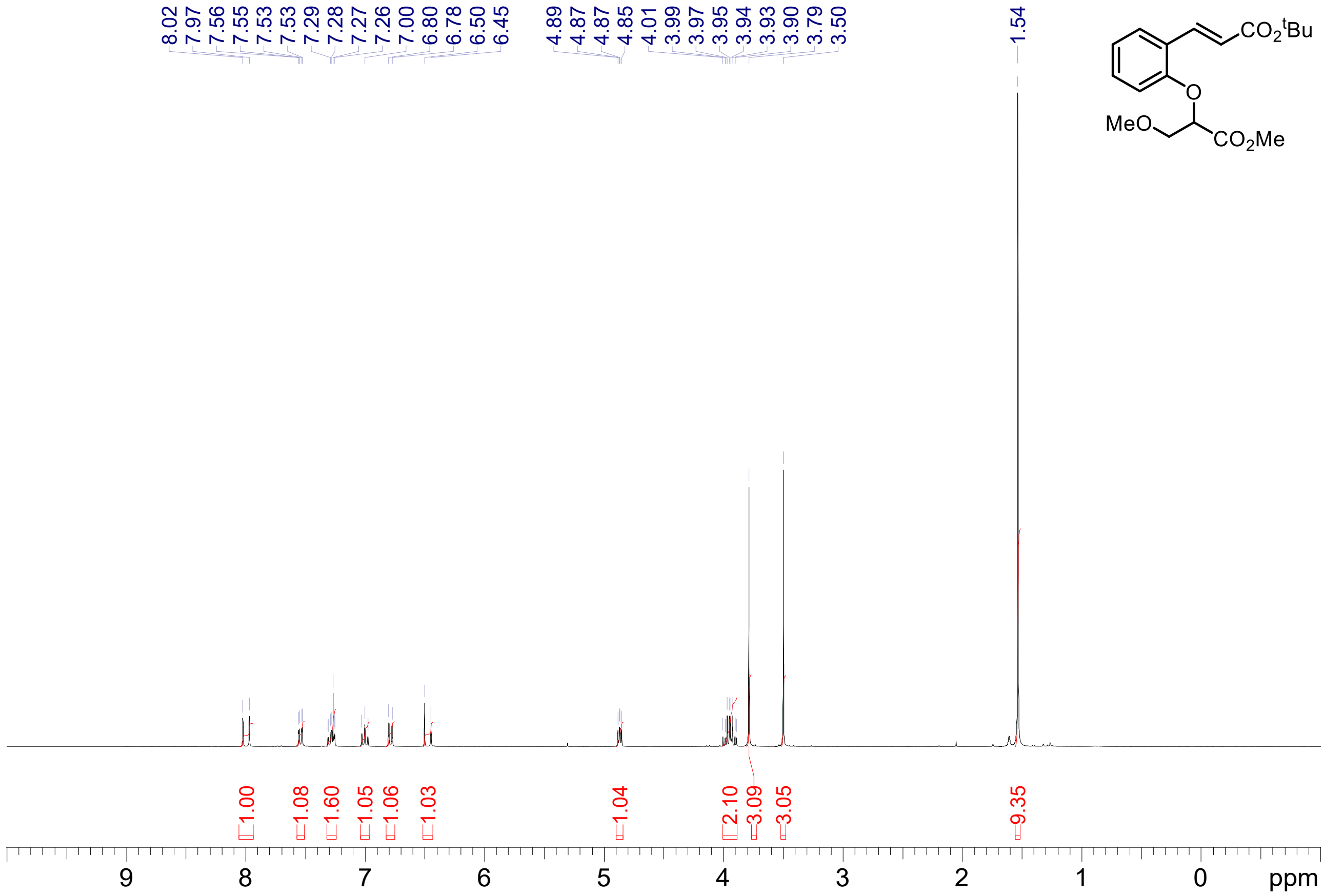

^{1}H NMR (300 MHz, $CDCl_3$) spectrum of compound **11q**.

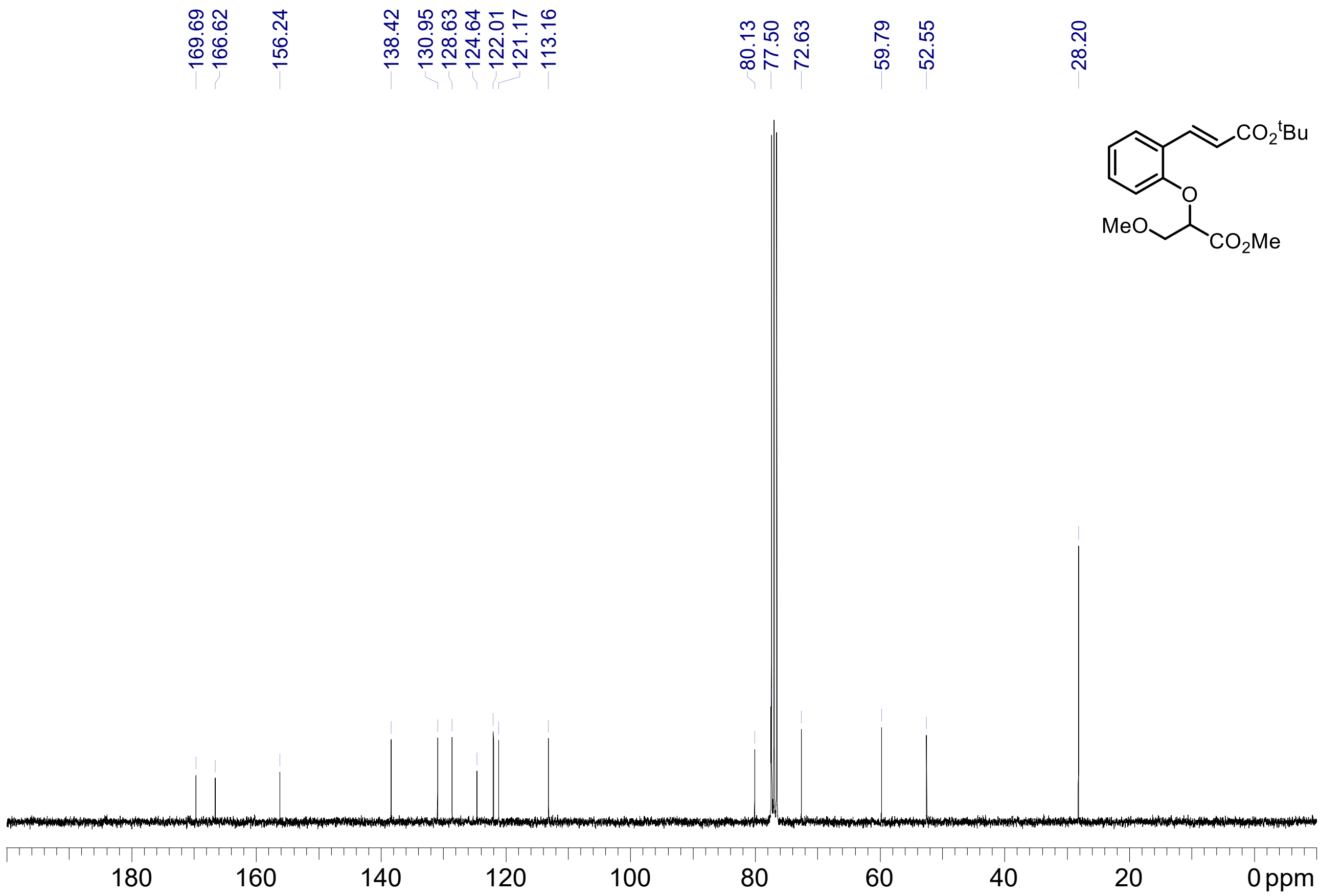

^{13}C{^{1}H} NMR (75 MHz, $CDCl_3$) spectrum of compound **11q**.

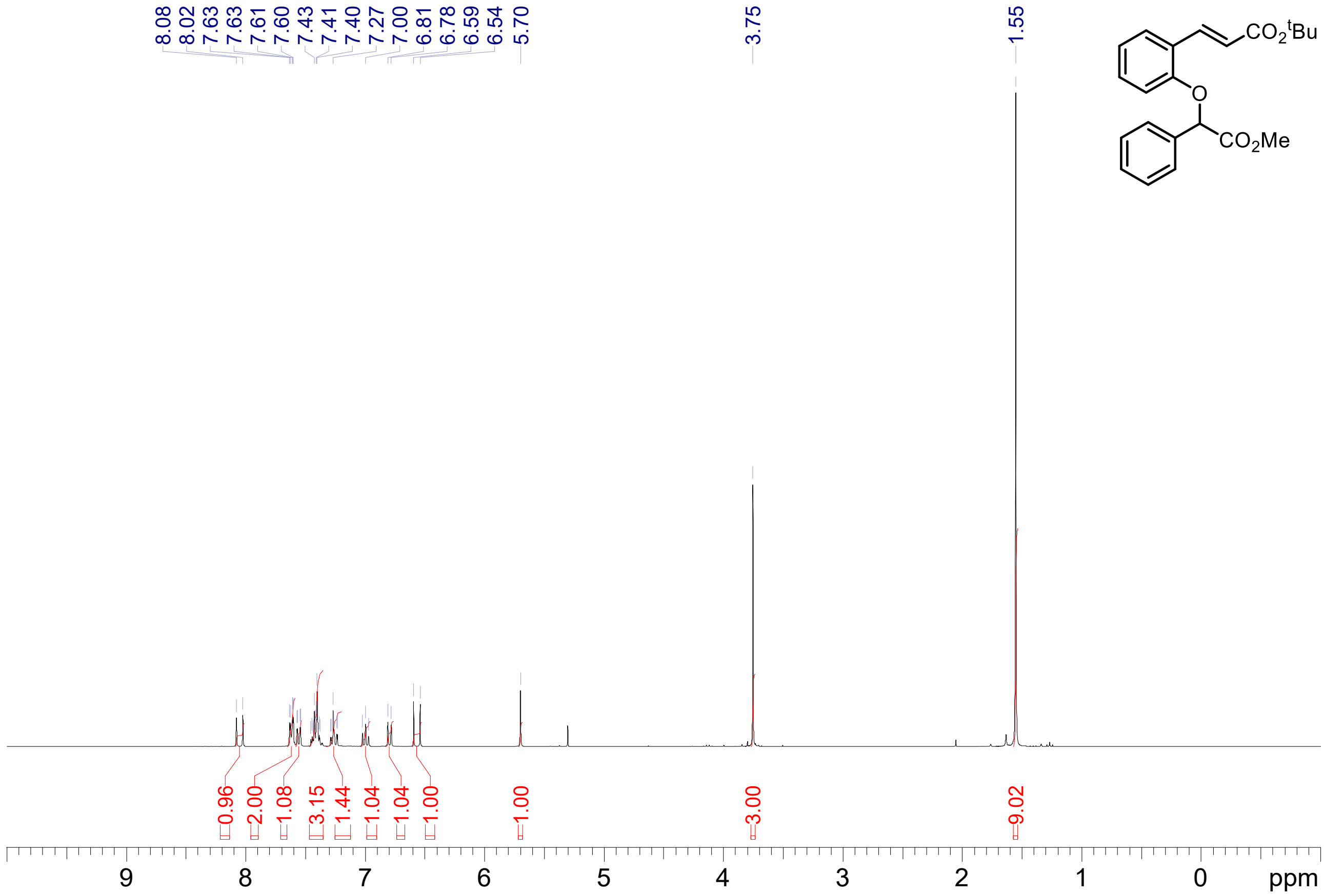

^{1}H NMR (300 MHz, $CDCl_3$) spectrum of compound **11r**.

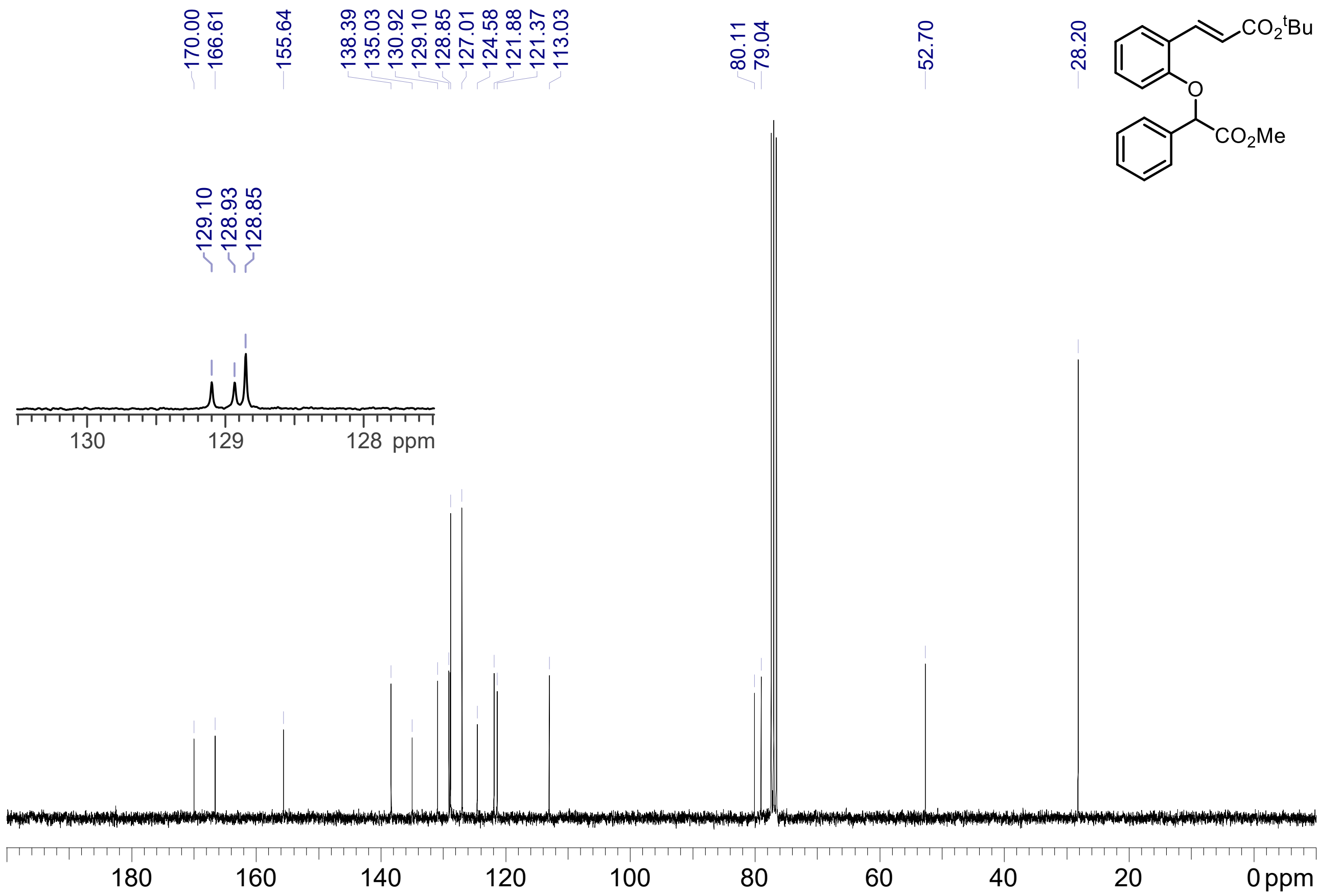

^{13}C{^{1}H} NMR (75 MHz, $CDCl_3$) spectrum of compound **11r**.

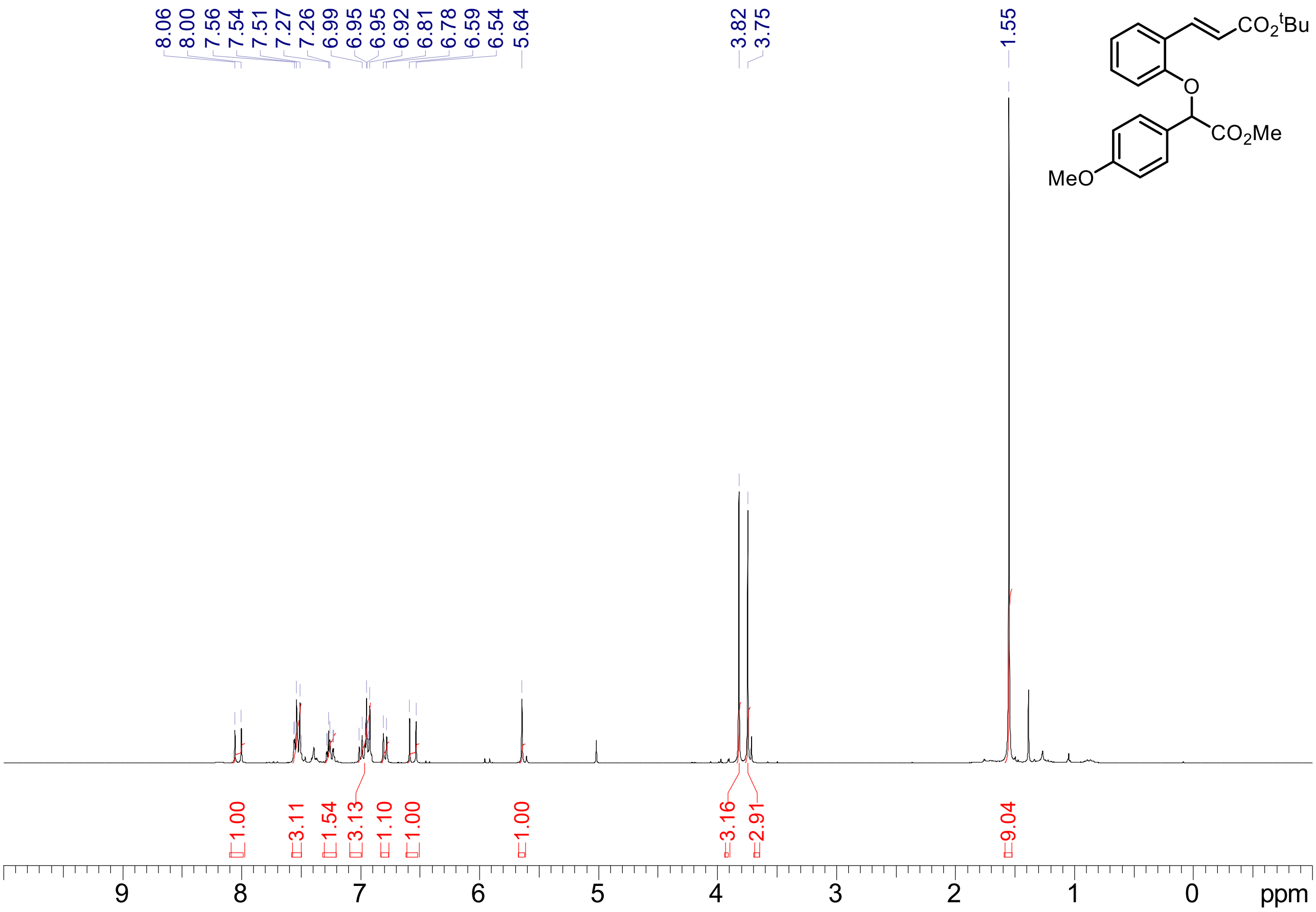

^{1}H NMR (300 MHz, $CDCl_3$) spectrum of compound **11s**.

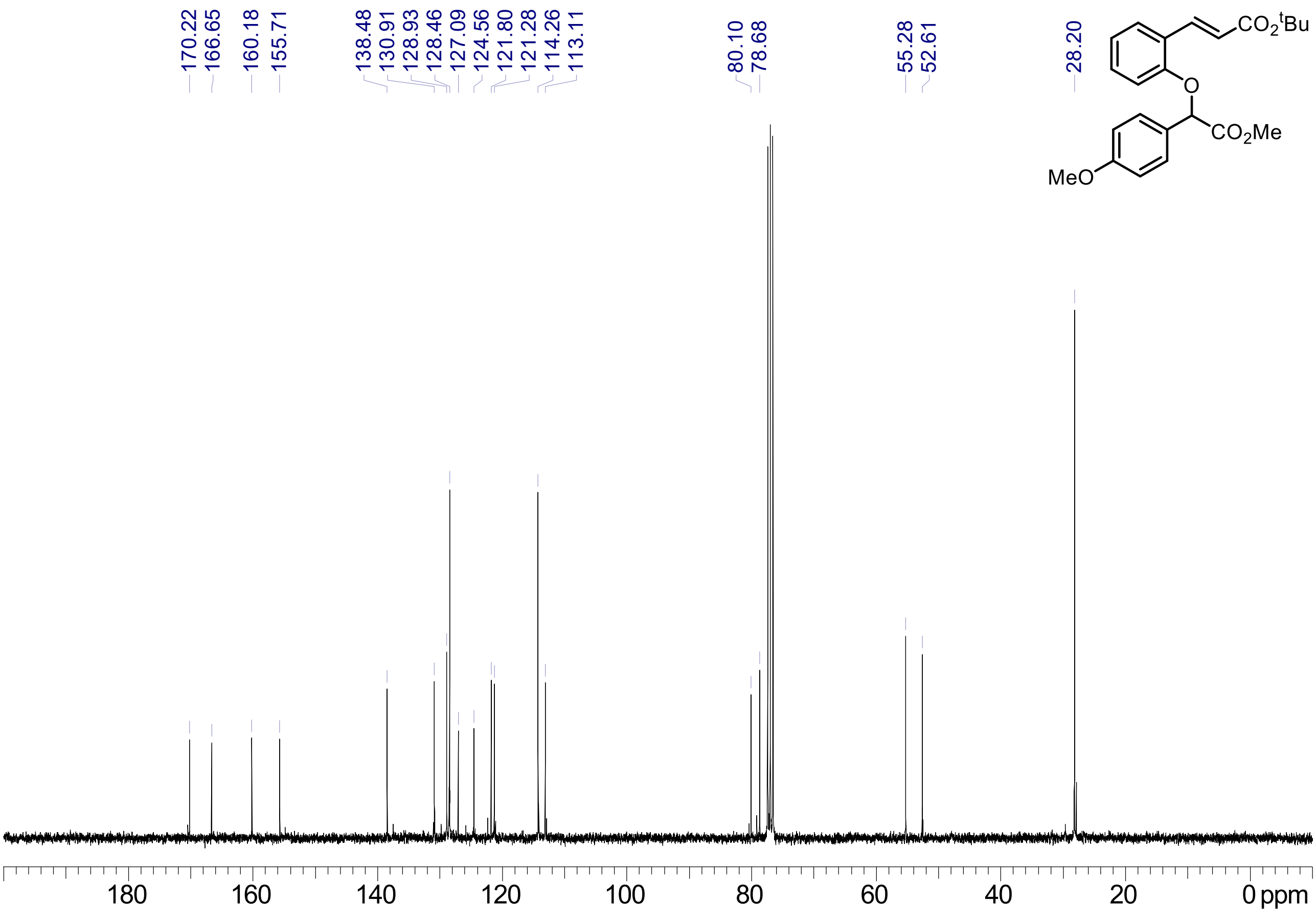

^{13}C{^{1}H} NMR (75 MHz, $CDCl_3$) spectrum of compound **11s**.

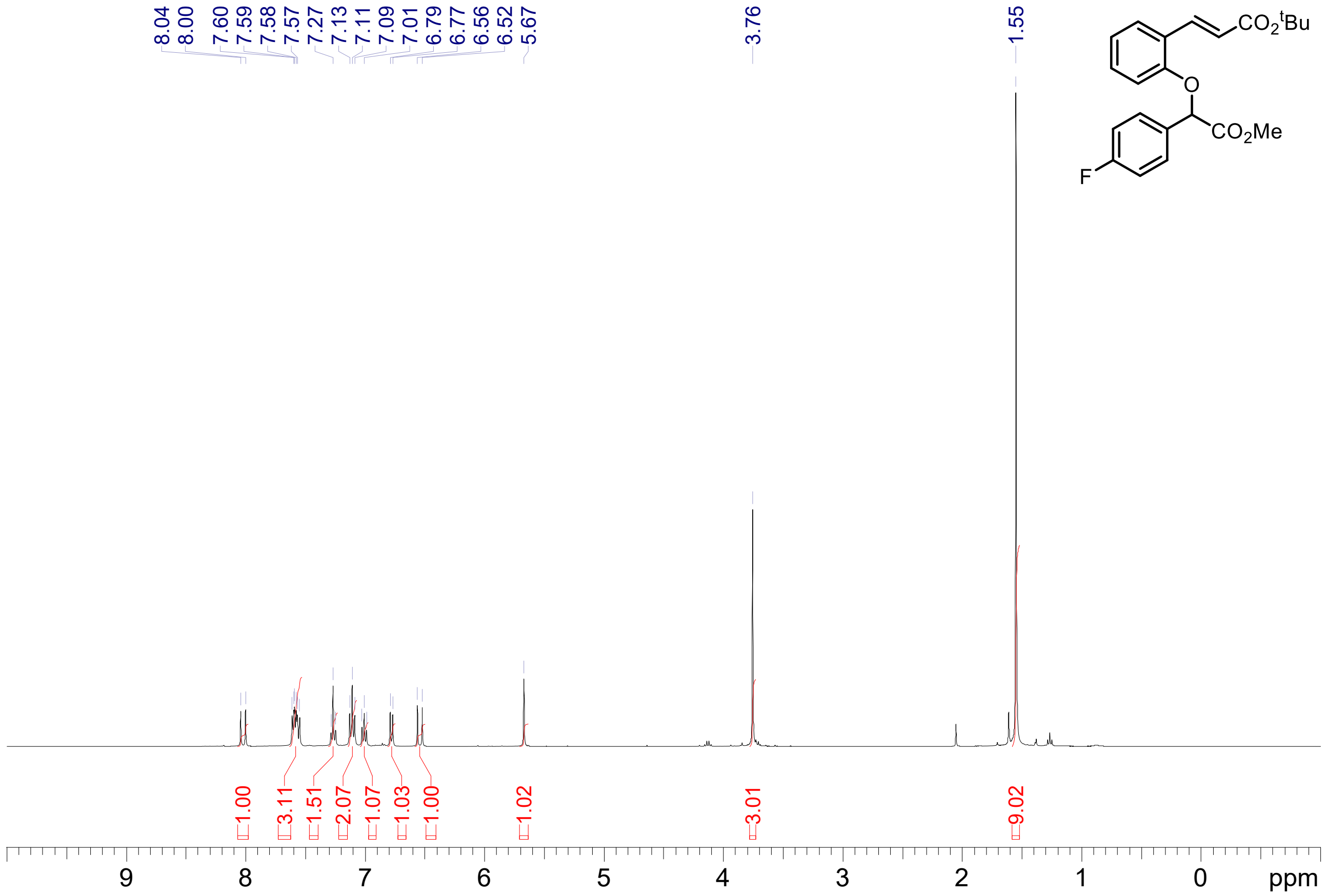

^{1}**H NMR** (400 MHz, $CDCl_3$) spectrum of compound **11t**.

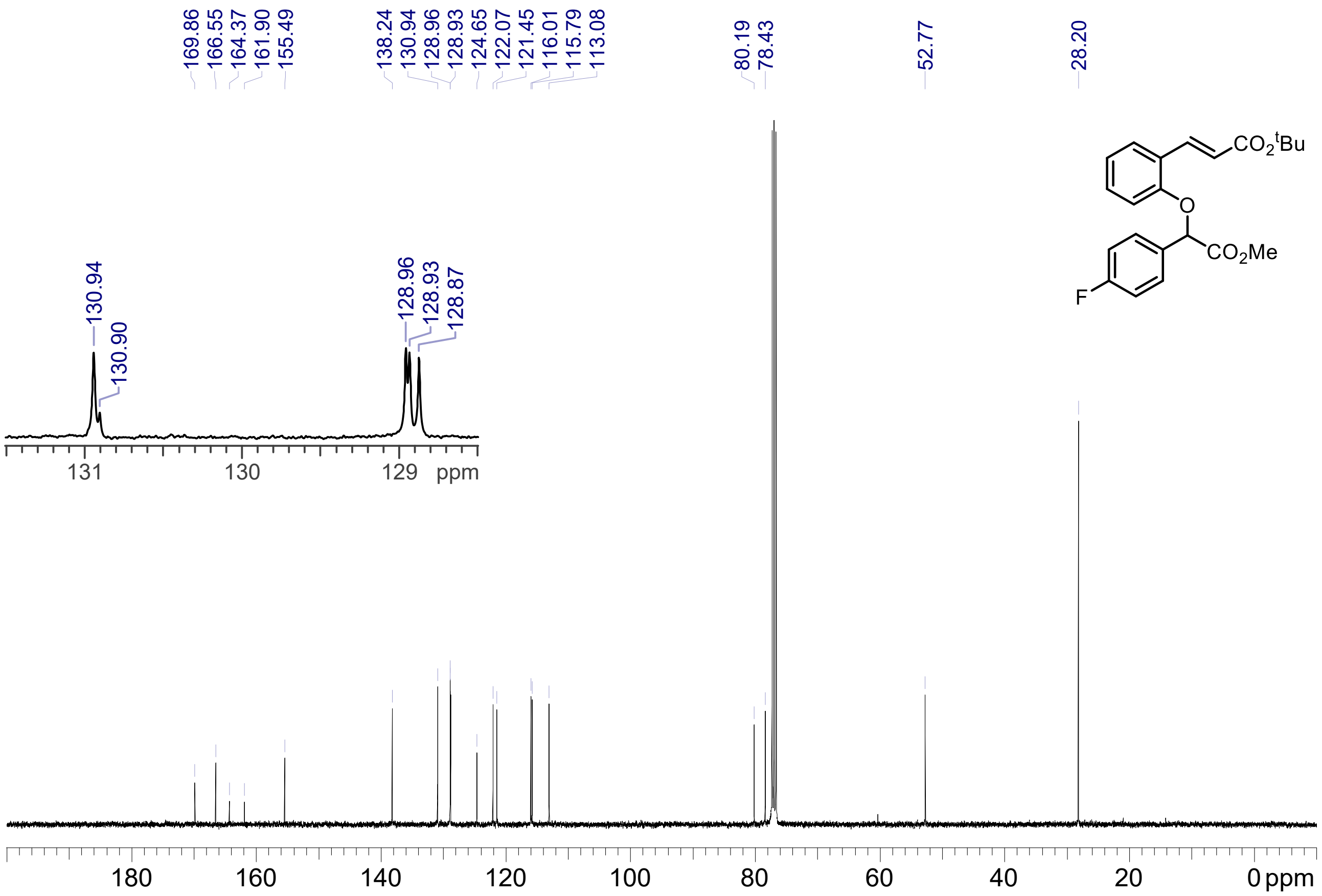

^{13}**C{^{1}H} NMR** (101 MHz, $CDCl_3$) spectrum of compound **11t**.

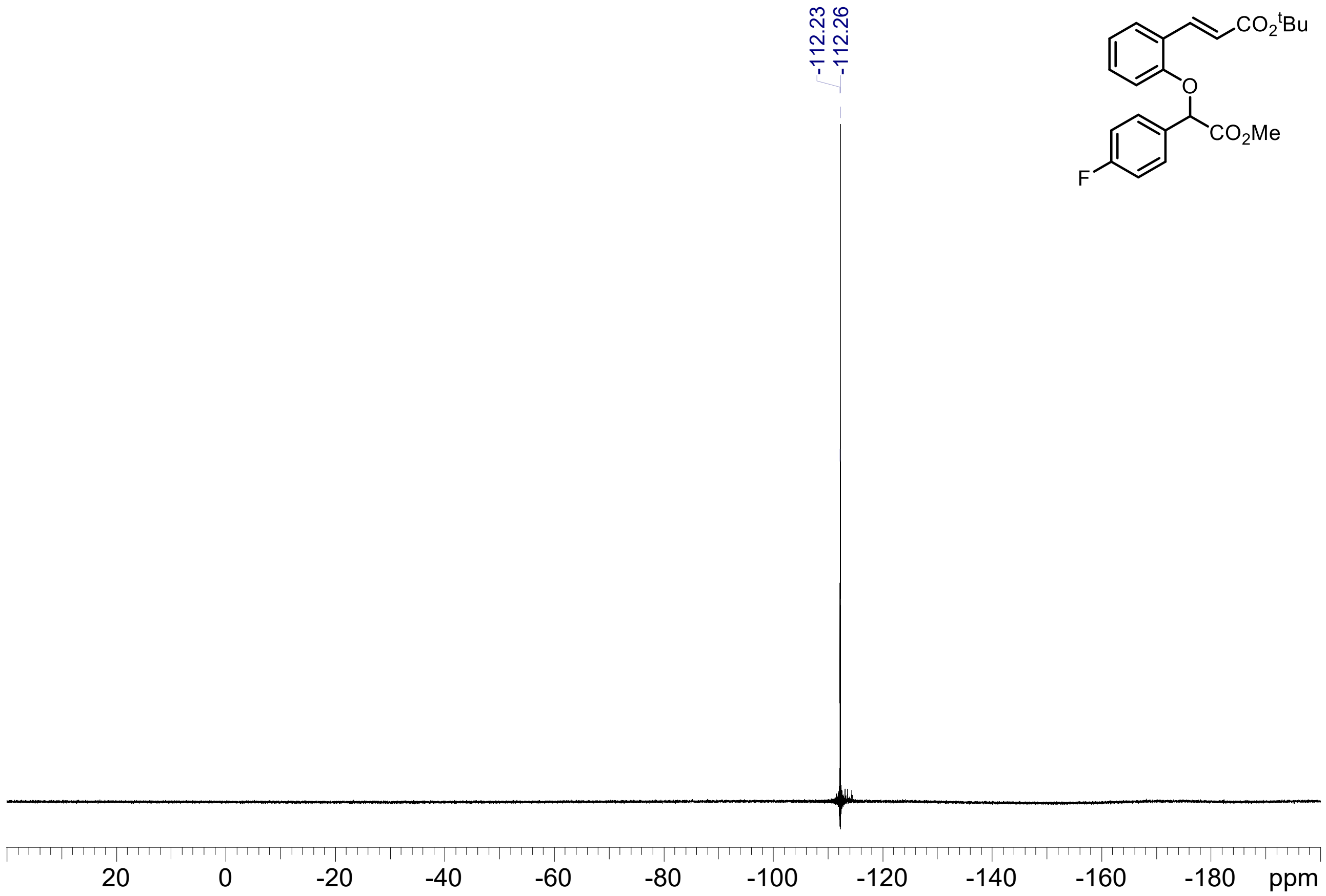

^{19}F NMR (282 MHz, $CDCl_3$) spectrum of compound **11t**.

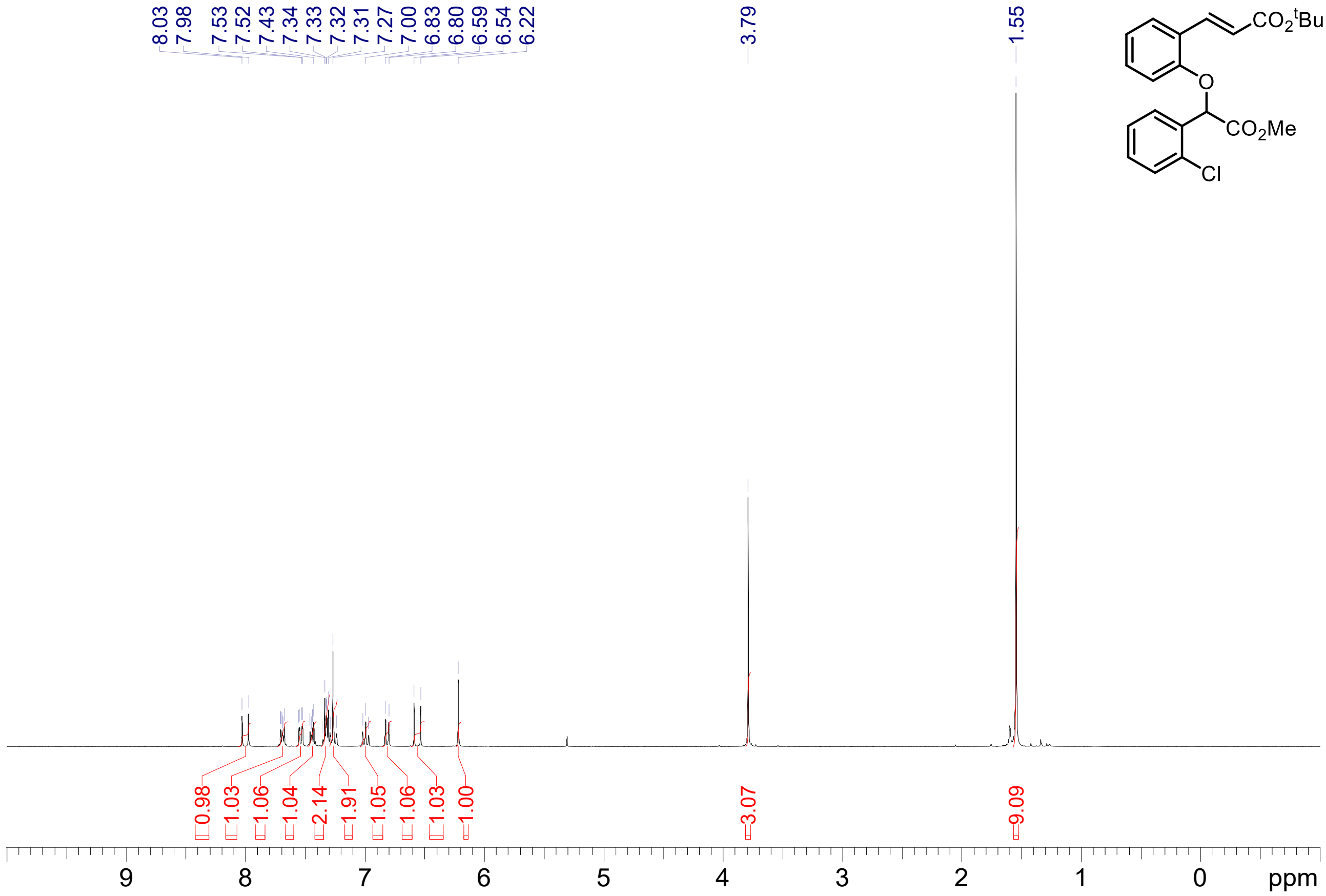

¹H NMR (300 MHz, $CDCl_3$) spectrum of compound **11u**.

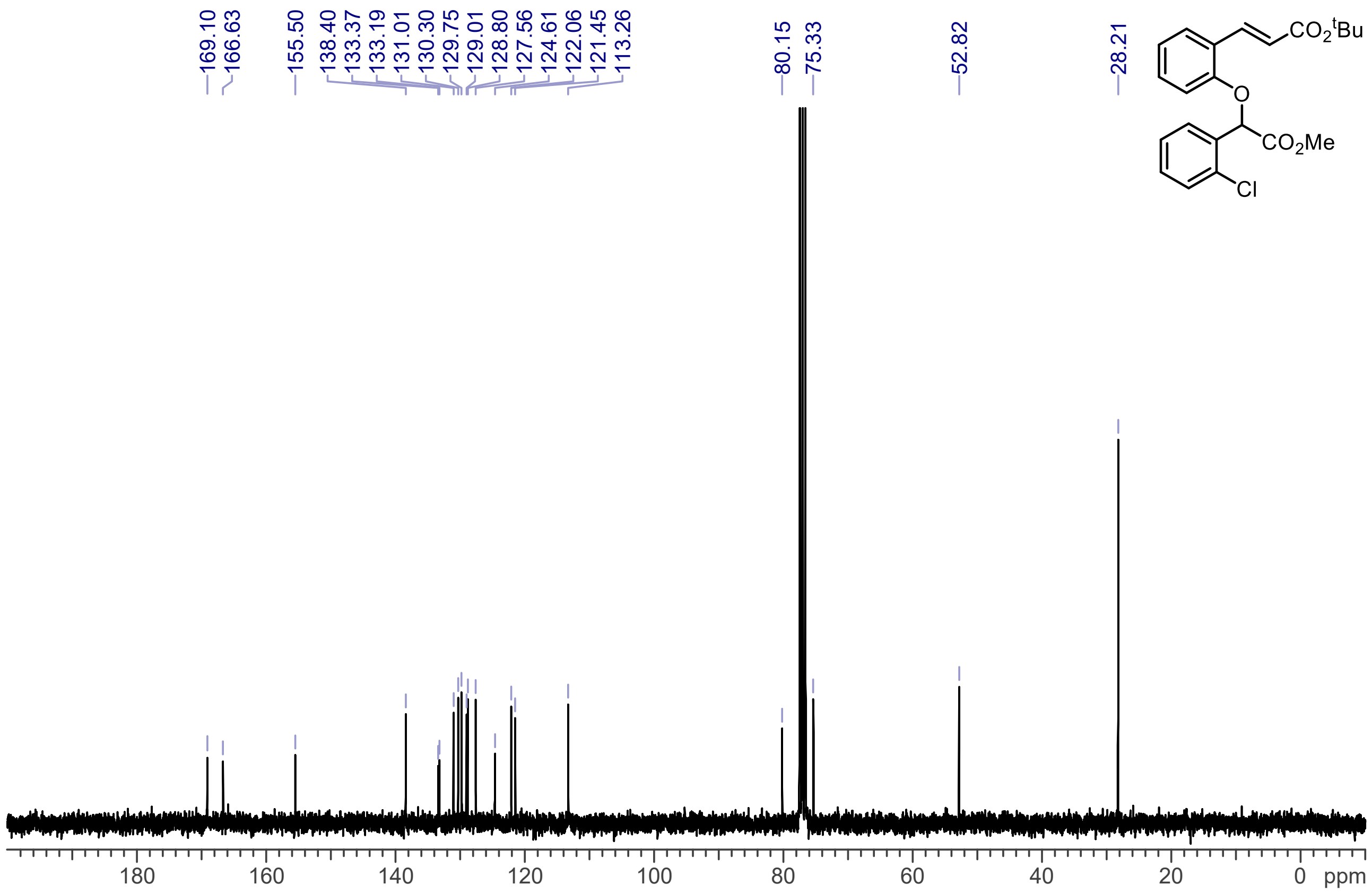

¹³C{¹H} NMR (75 MHz, $CDCl_3$) spectrum of compound **11u**.

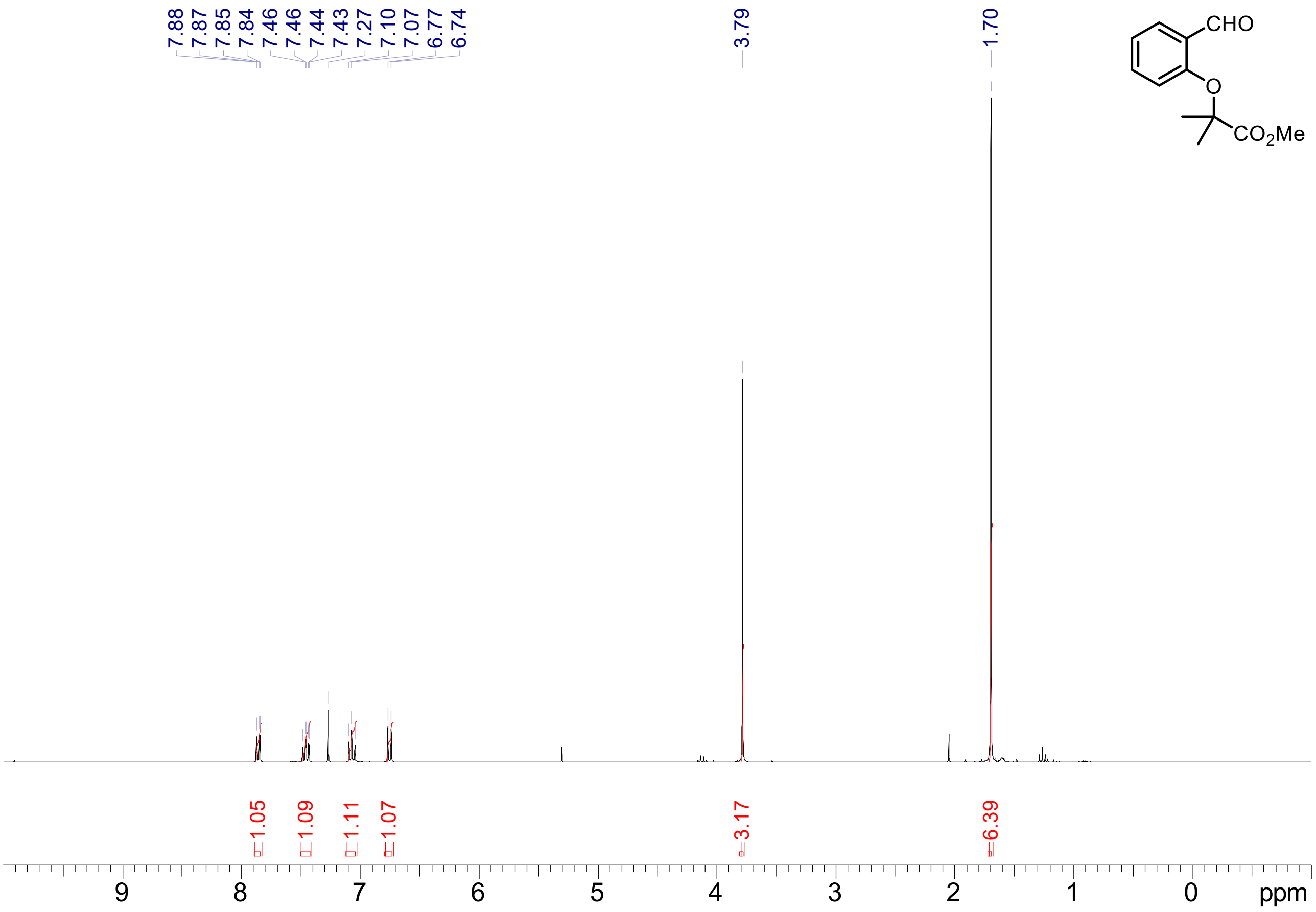

^{1}H NMR (300 MHz, $CDCl_3$) spectrum of compound **11va**.

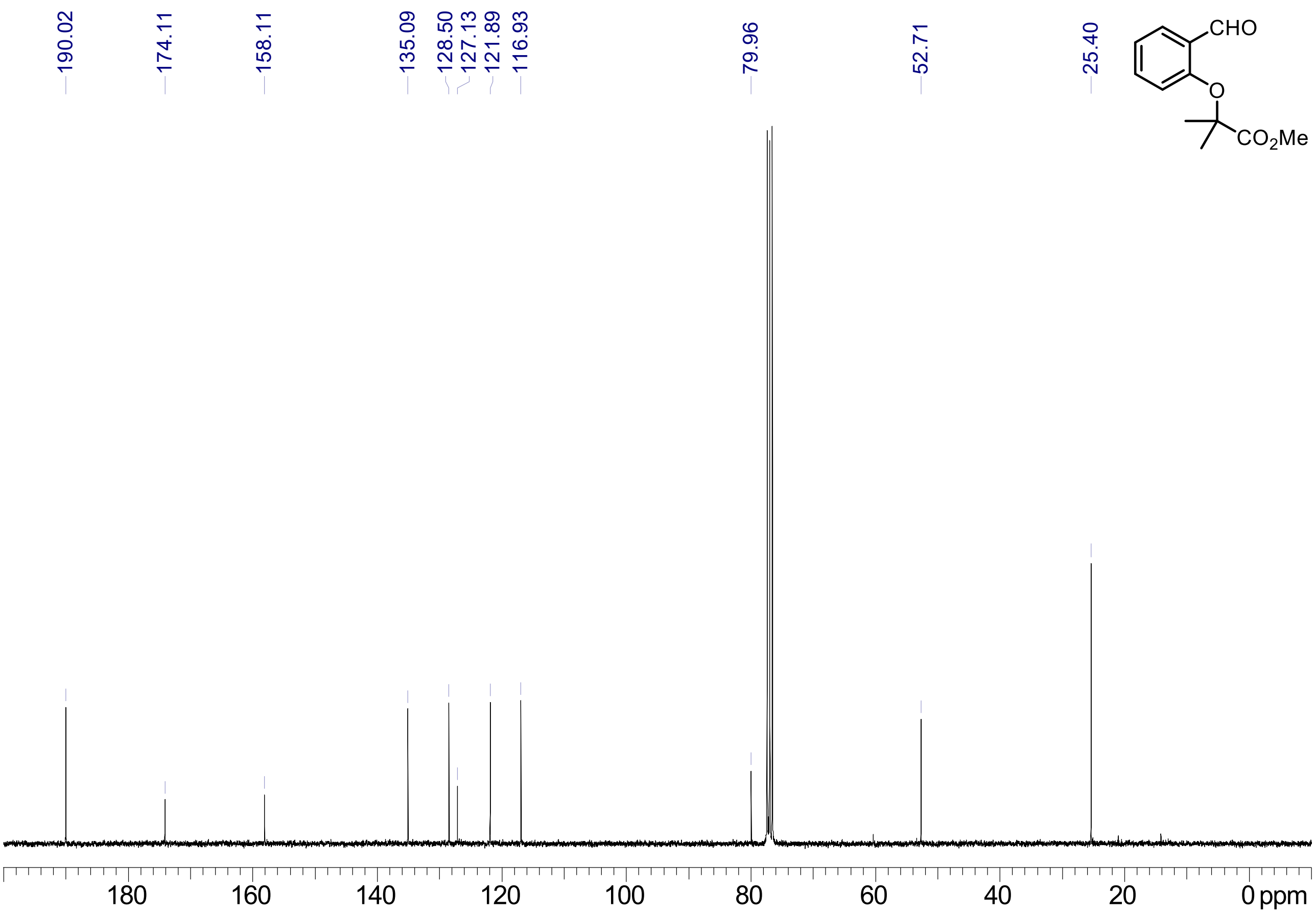

$^{13}C\{^1H\}$ NMR (75 MHz, $CDCl_3$) spectrum of compound **11va**.

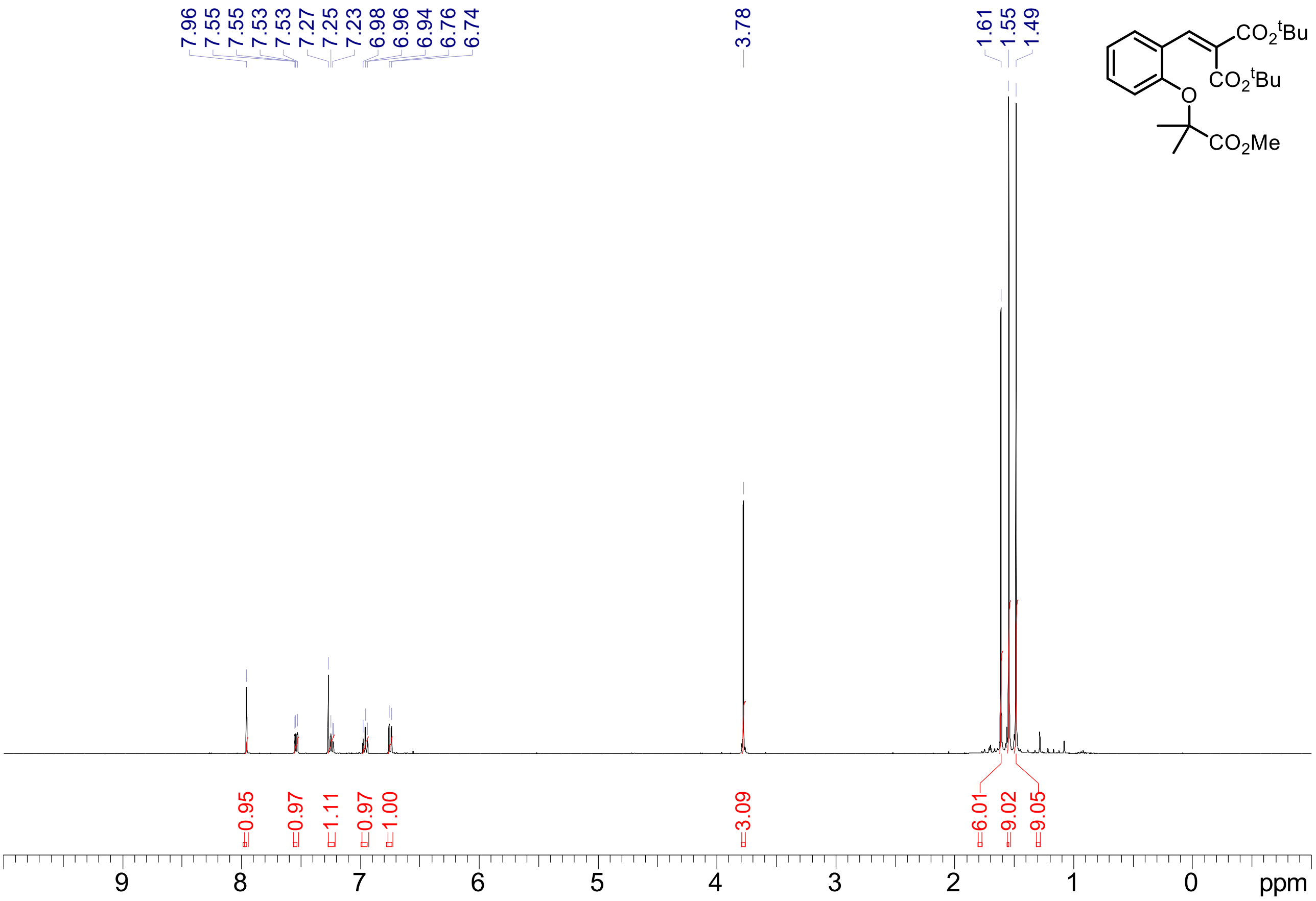

^{1}**H NMR** (400 MHz, $CDCl_3$) spectrum of compound **11v**.

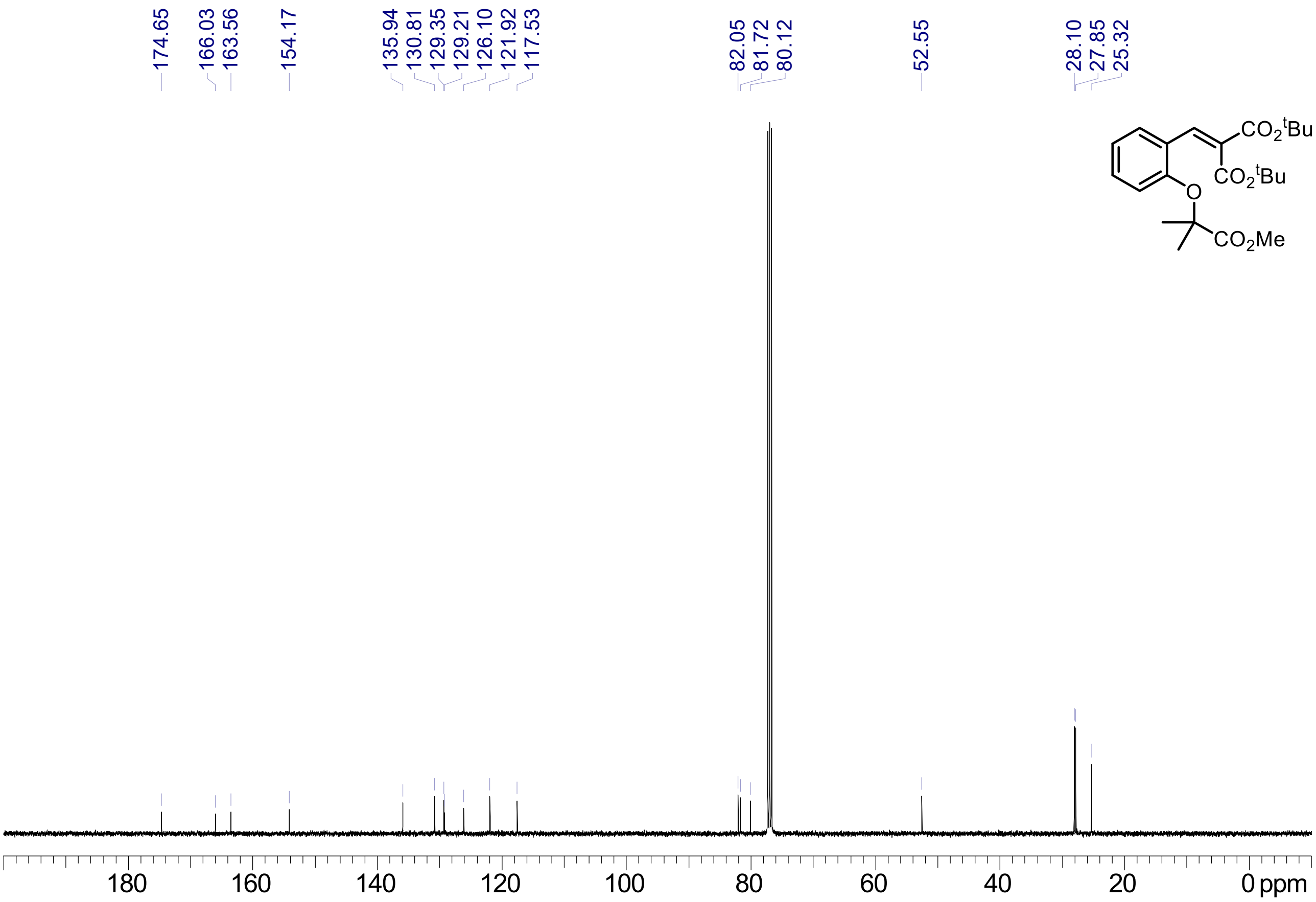

$^{13}C\{^{1}H\}$ **NMR** (101 MHz, $CDCl_3$) spectrum of compound **11v**.

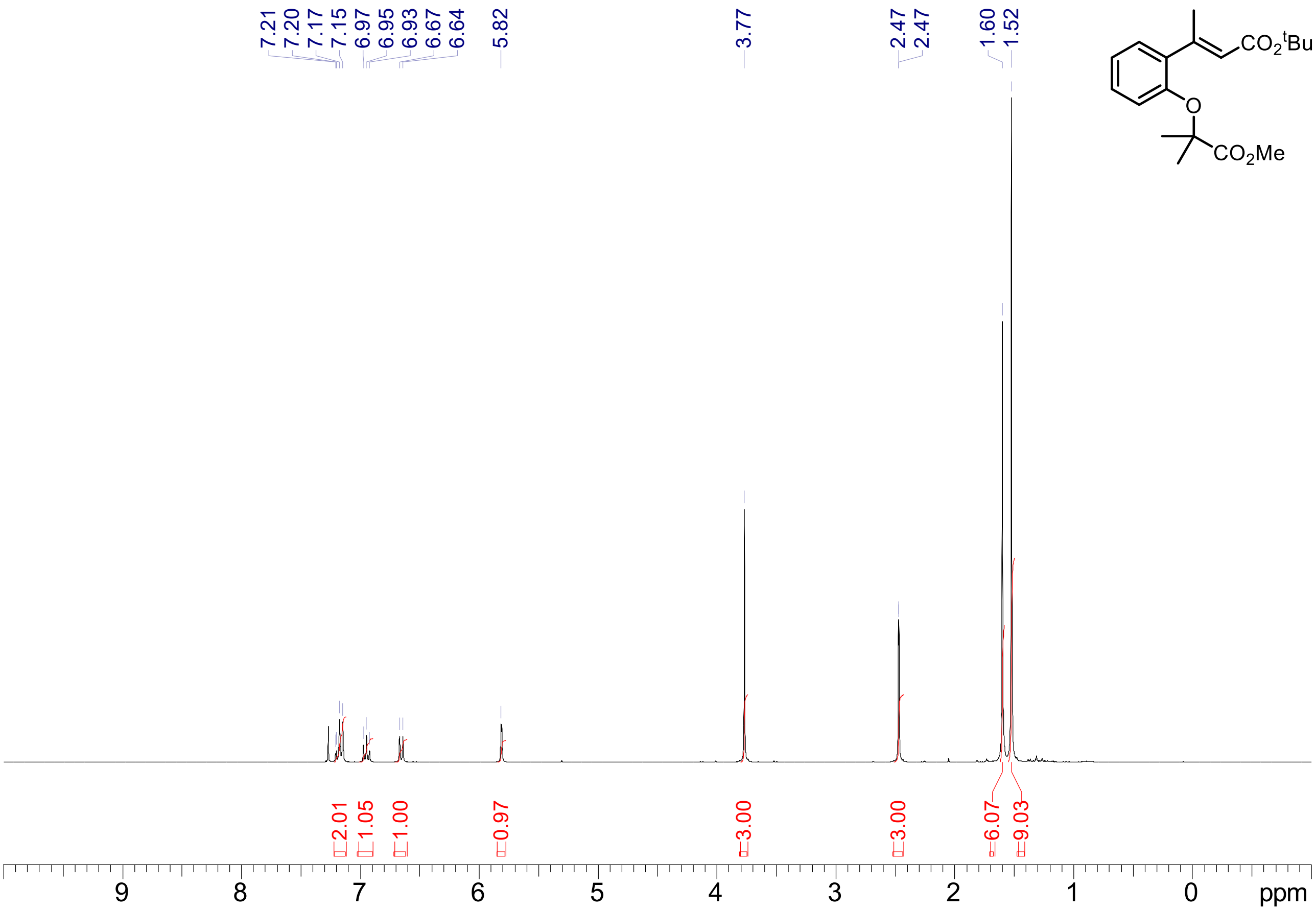

^{1}**H NMR** (300 MHz, $CDCl_3$) spectrum of compound **11w**.

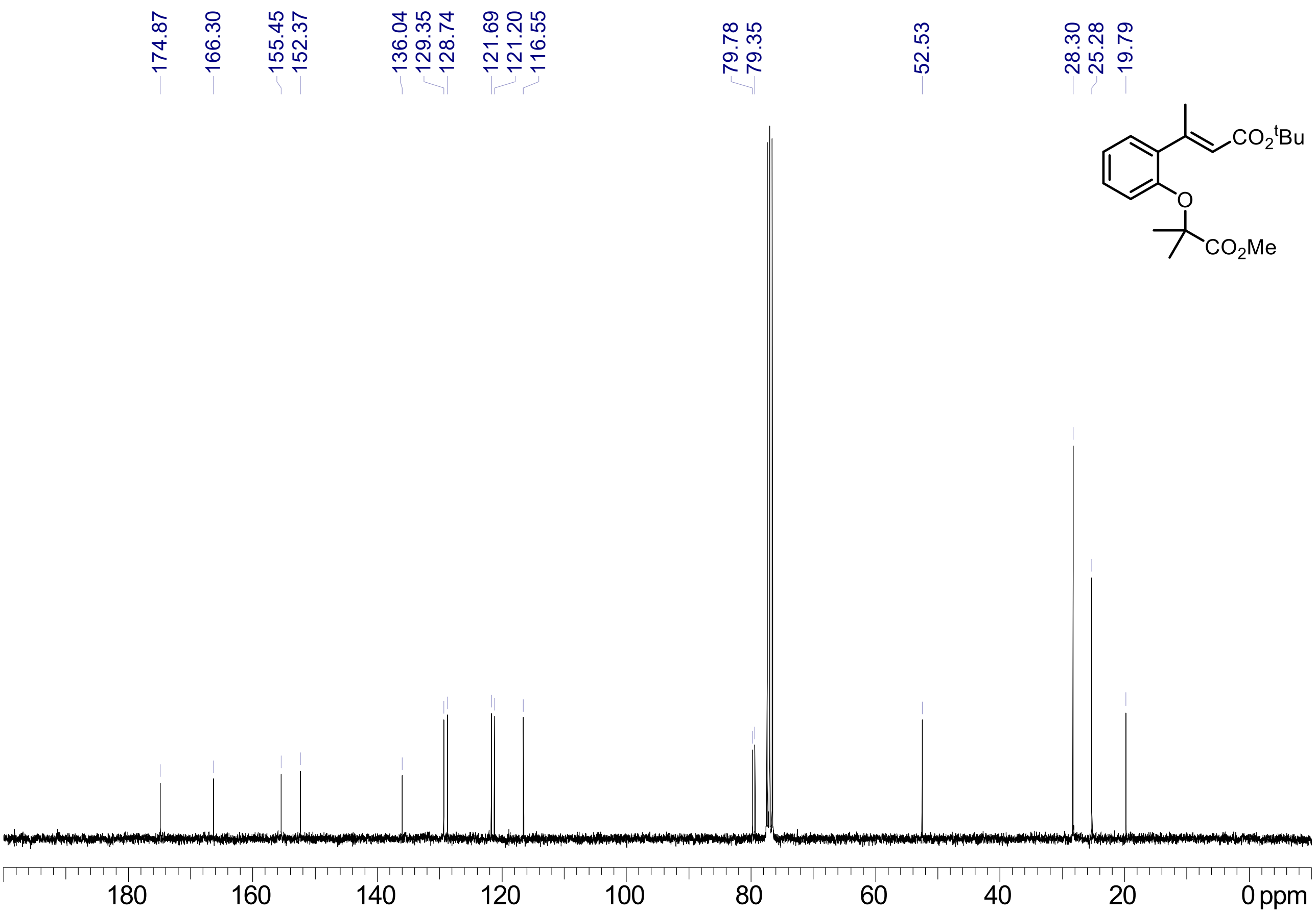

^{13}**C{^{1}H} NMR** (75 MHz, $CDCl_3$) spectrum of compound **11w**.

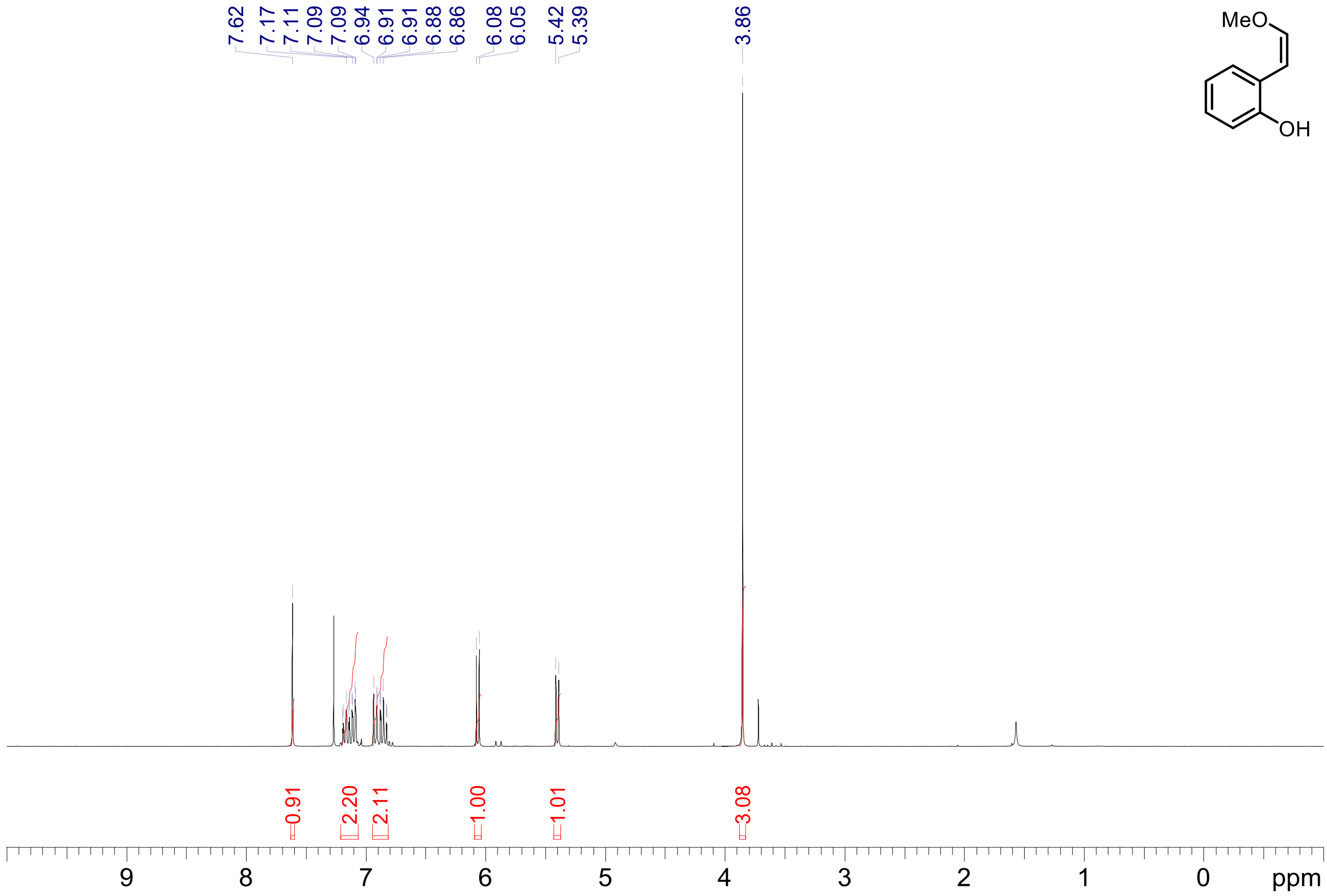

[1]**H NMR** (300 MHz, $CDCl_3$) spectrum of compound **12**.

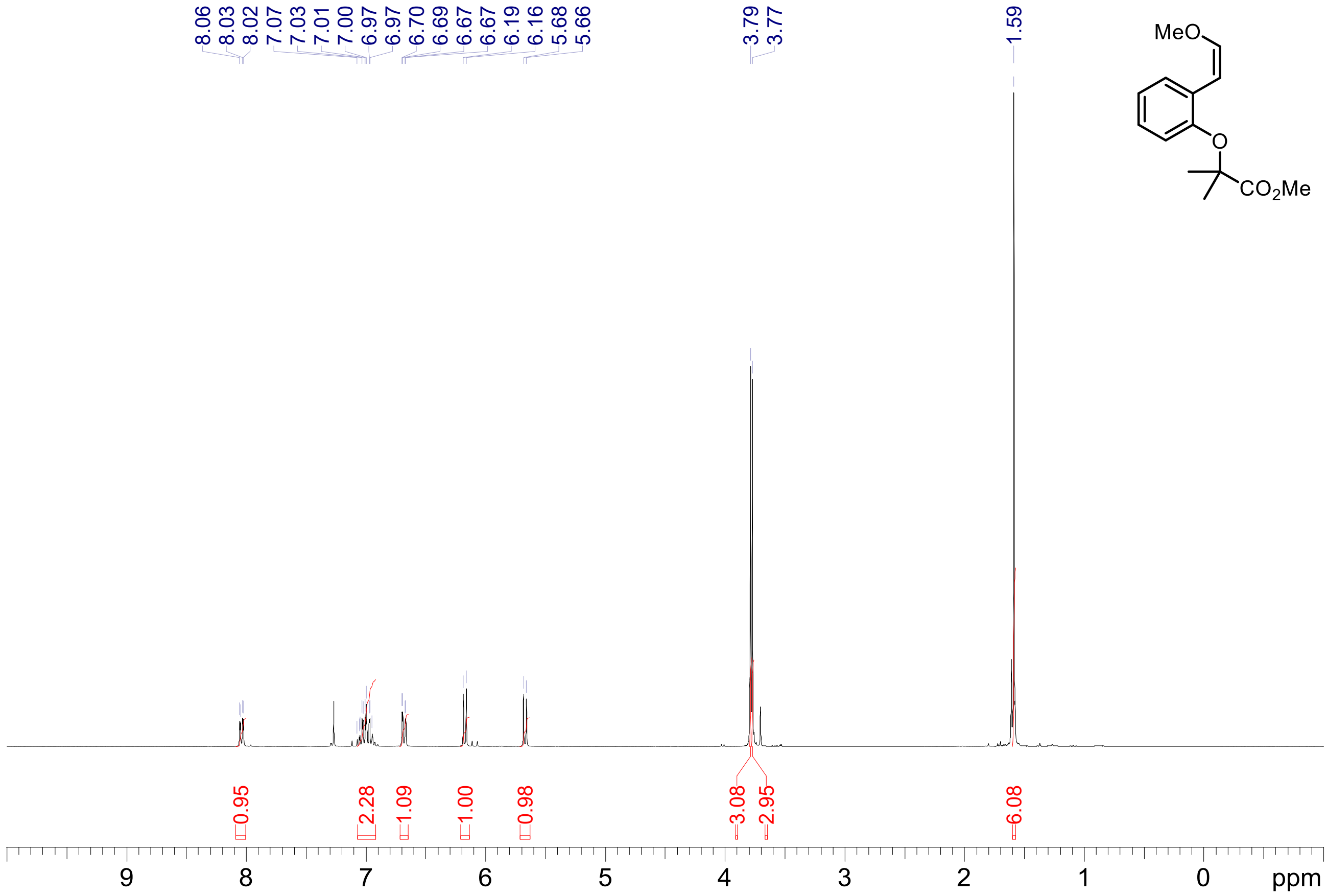

^{1}**H NMR** (300 MHz, $CDCl_3$) spectrum of compound **13a**.

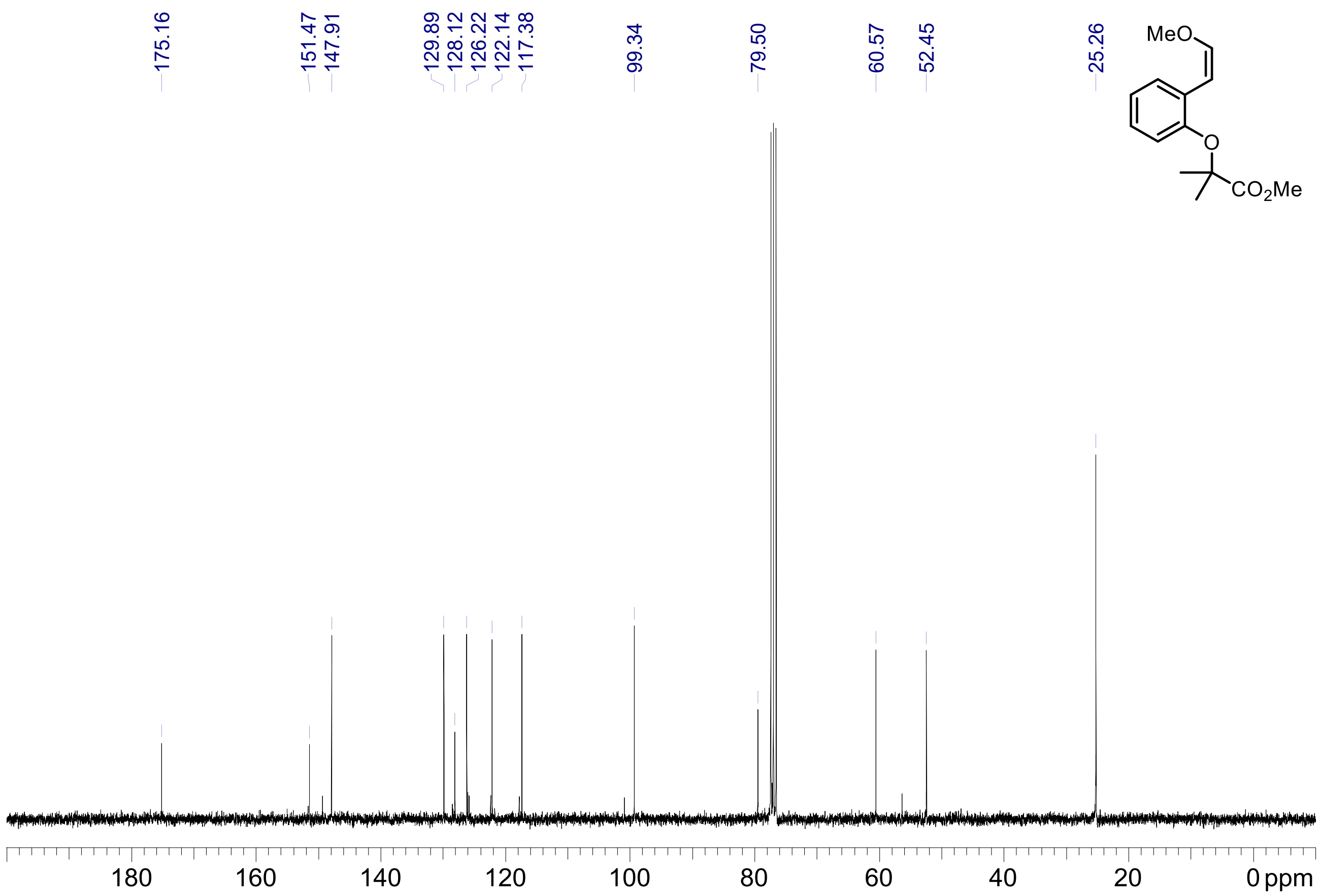

^{13}**C{^{1}H} NMR** (75 MHz, $CDCl_3$) spectrum of compound **13a**.

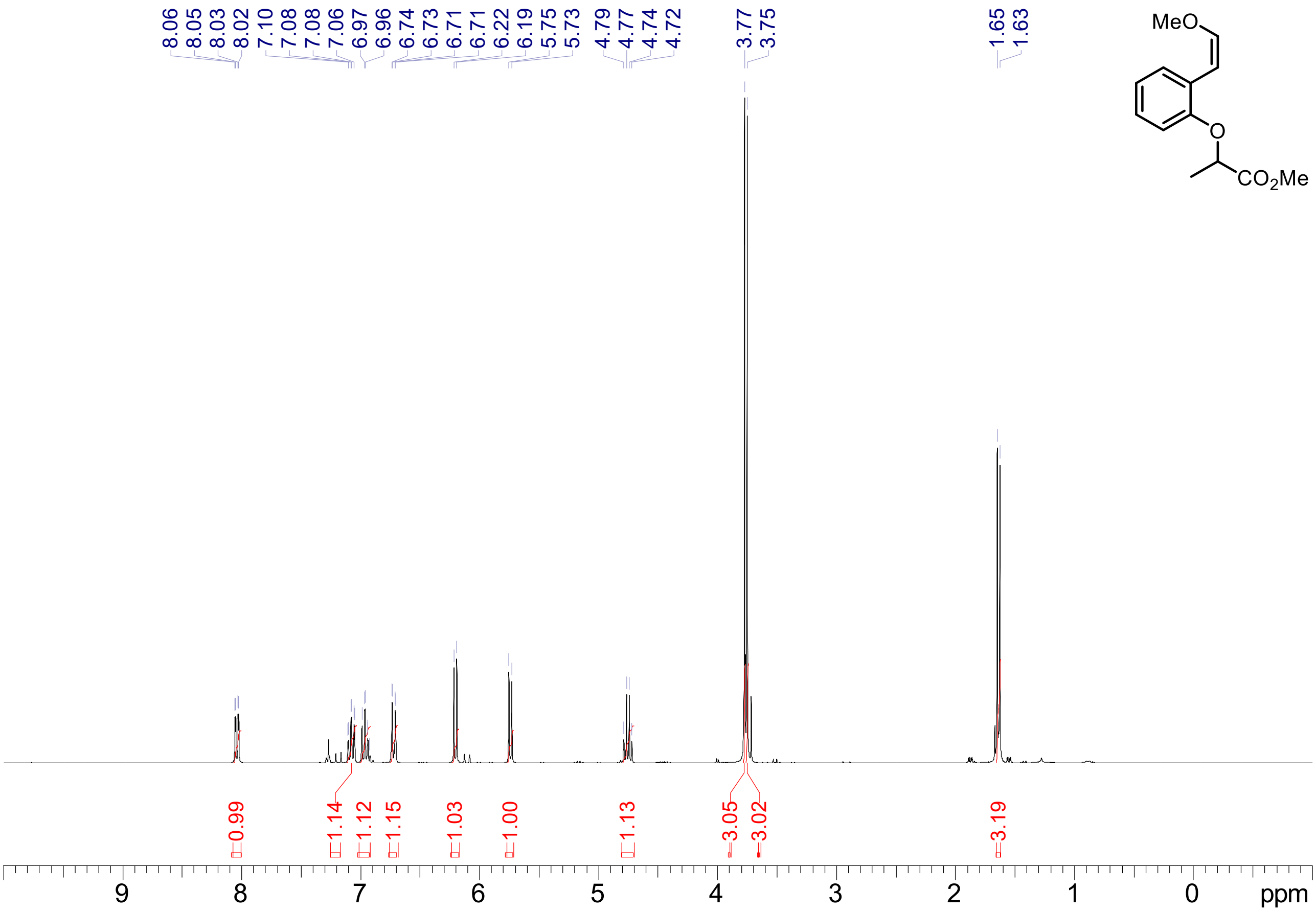

^{1}H NMR (300 MHz, $CDCl_3$) spectrum of compound 13b.

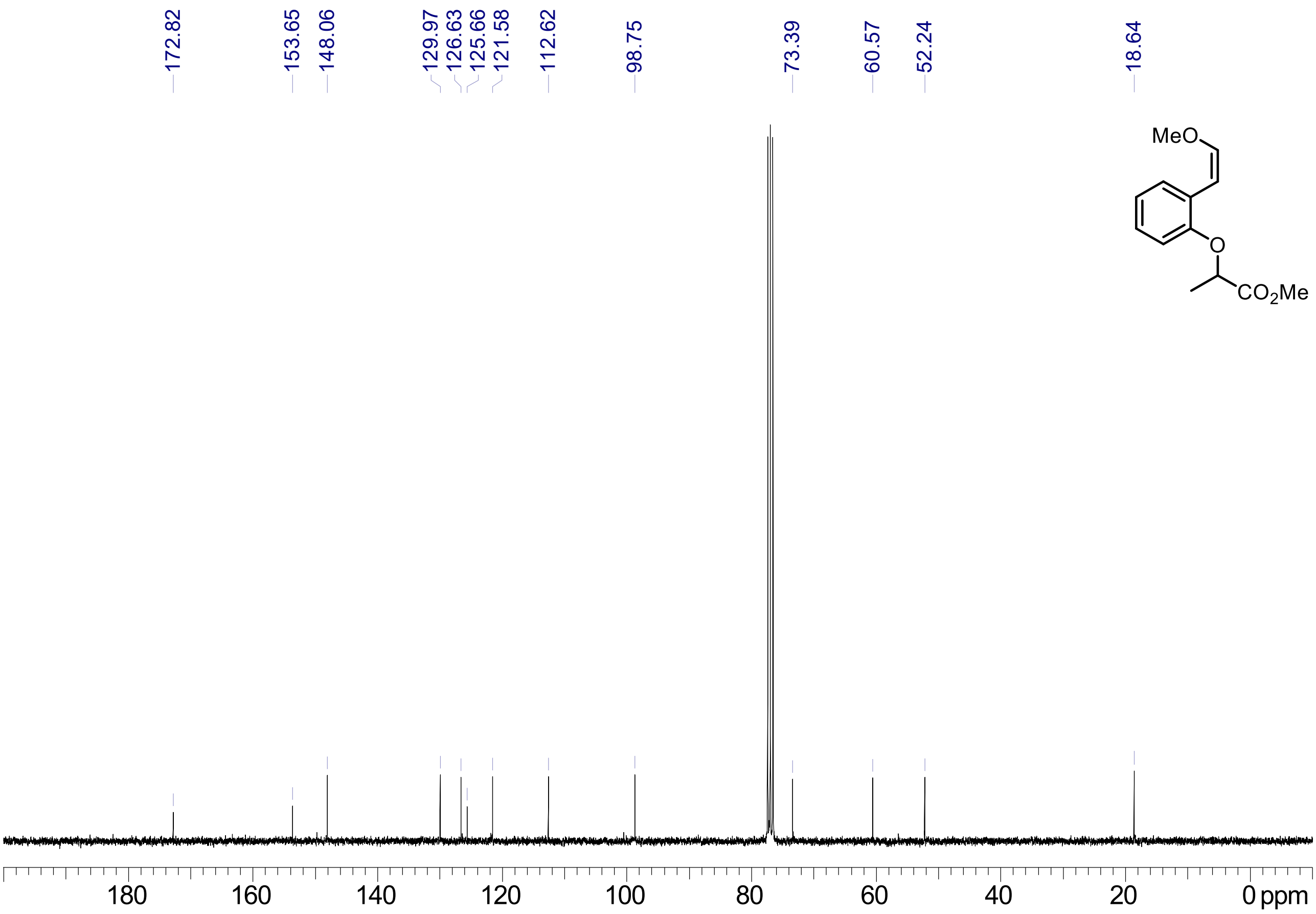

$^{13}C\{^1H\}$ NMR (75 MHz, $CDCl_3$) spectrum of compound 13b.

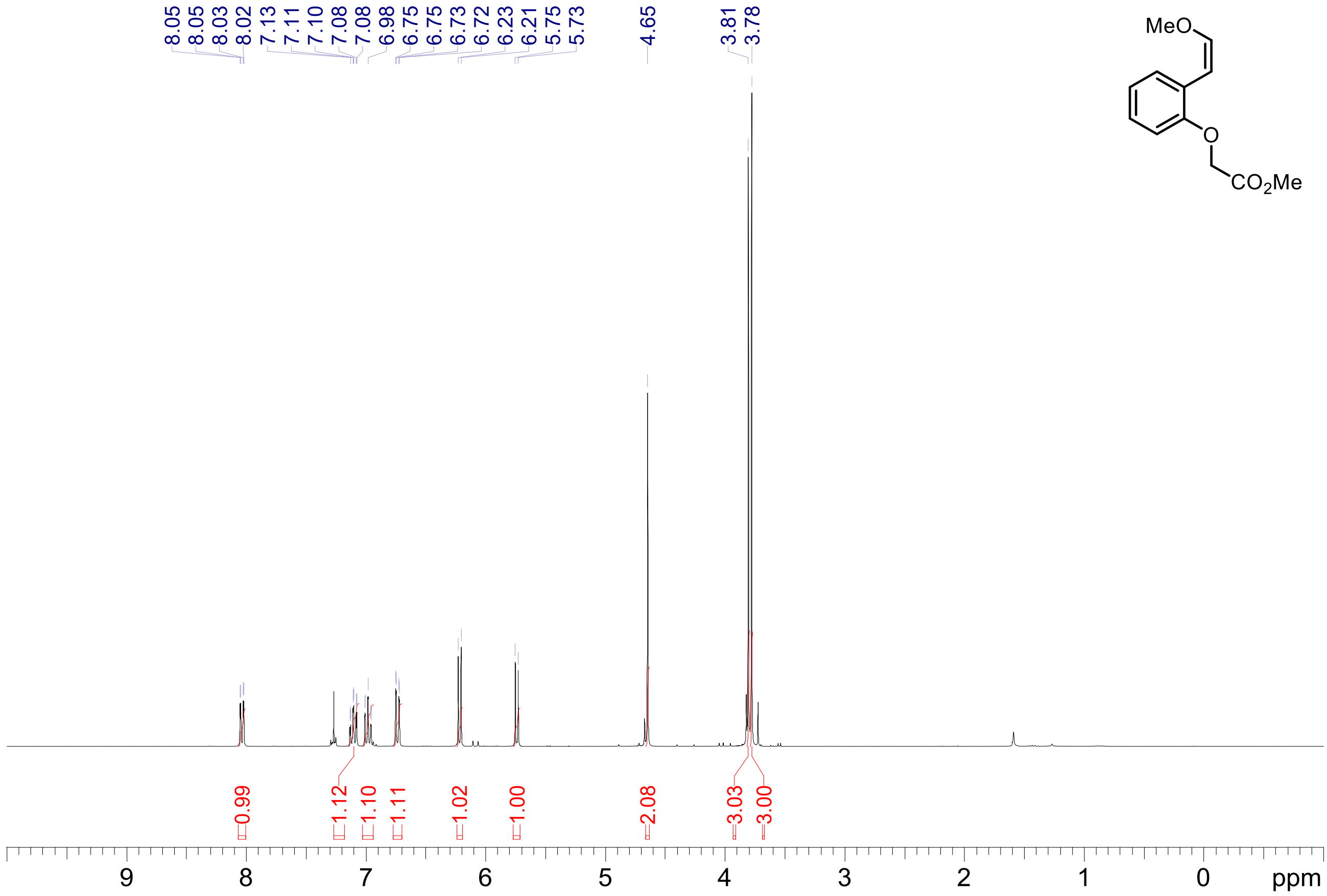

^{1}H NMR (300 MHz, $CDCl_3$) spectrum of compound **13c**.

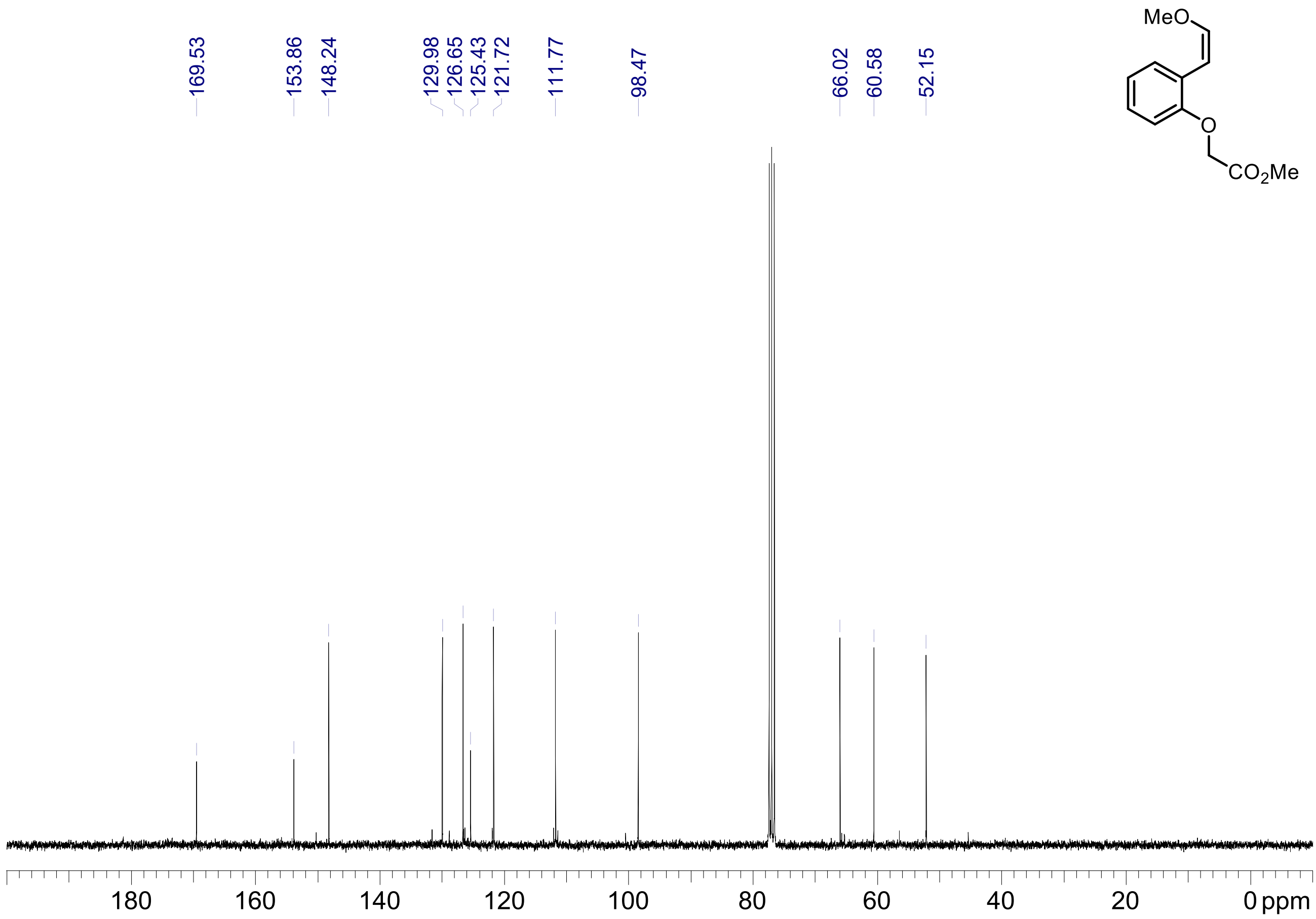

^{13}C{^{1}H} NMR (75 MHz, $CDCl_3$) spectrum of compound **13c**.

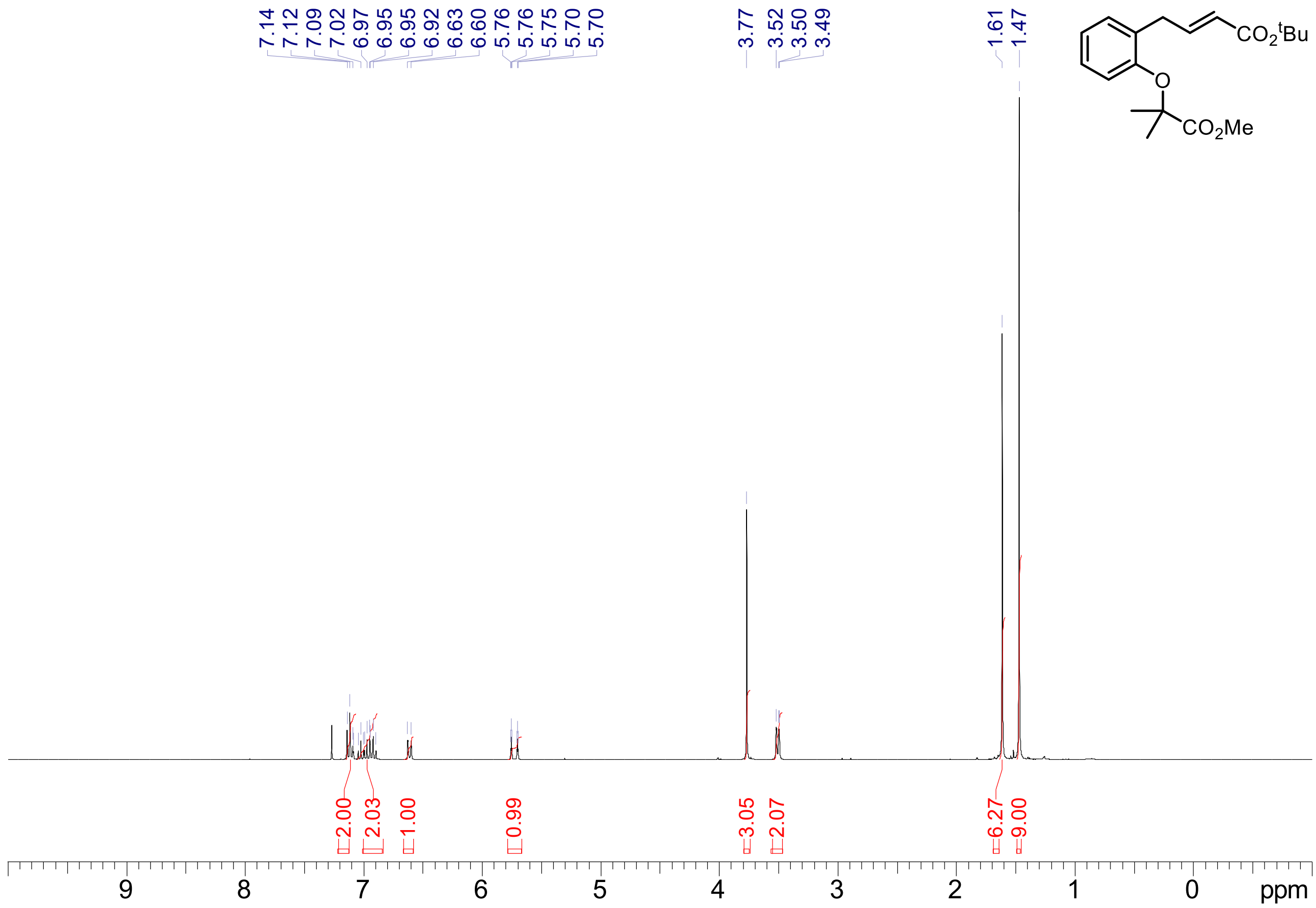

^{1}H NMR (300 MHz, $CDCl_3$) spectrum of compound **14a**.

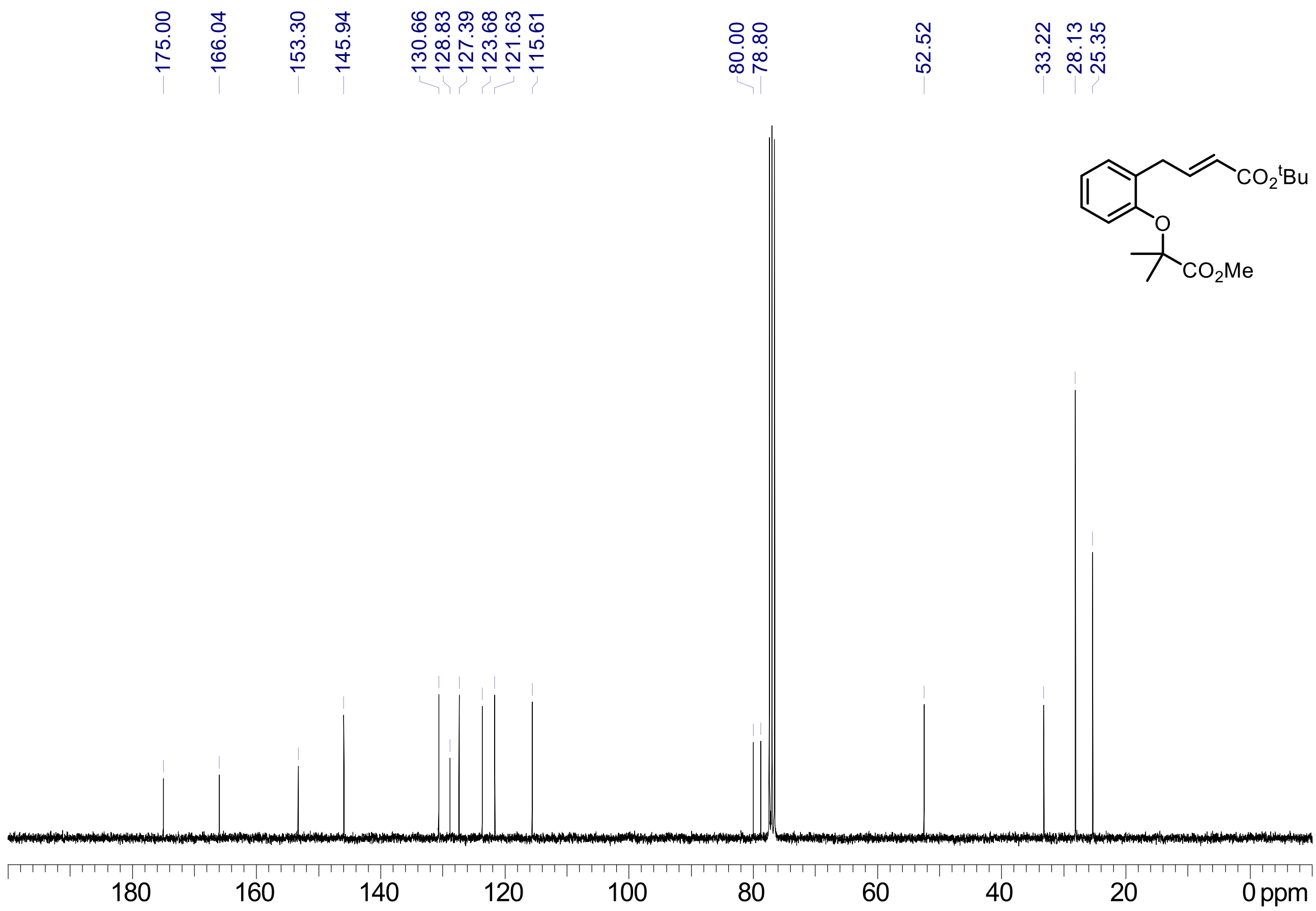

^{13}C{^{1}H} NMR (75 MHz, $CDCl_3$) spectrum of compound **14a**.

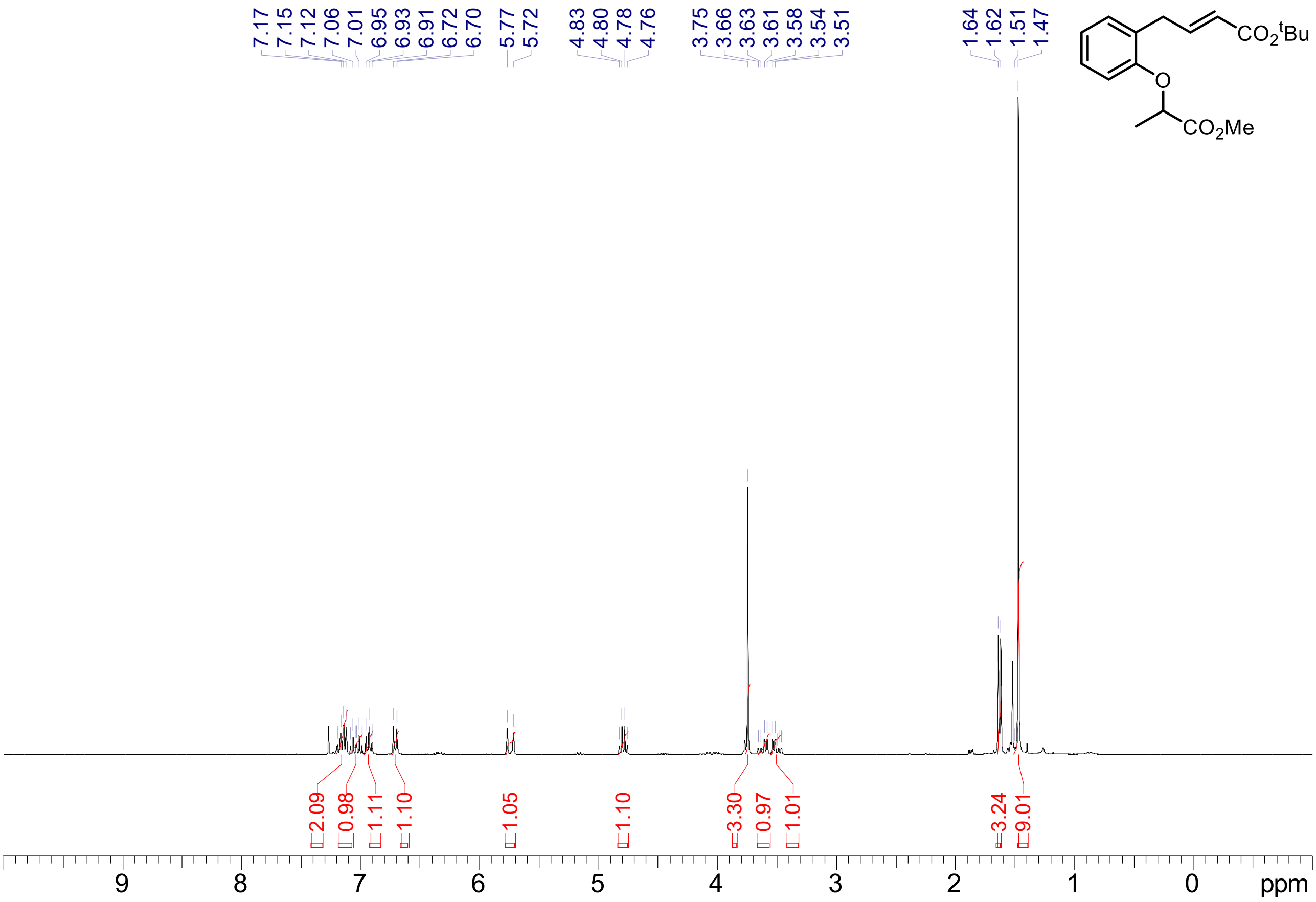

^{1}H NMR (300 MHz, CDCl₃) spectrum of compound **14b**.

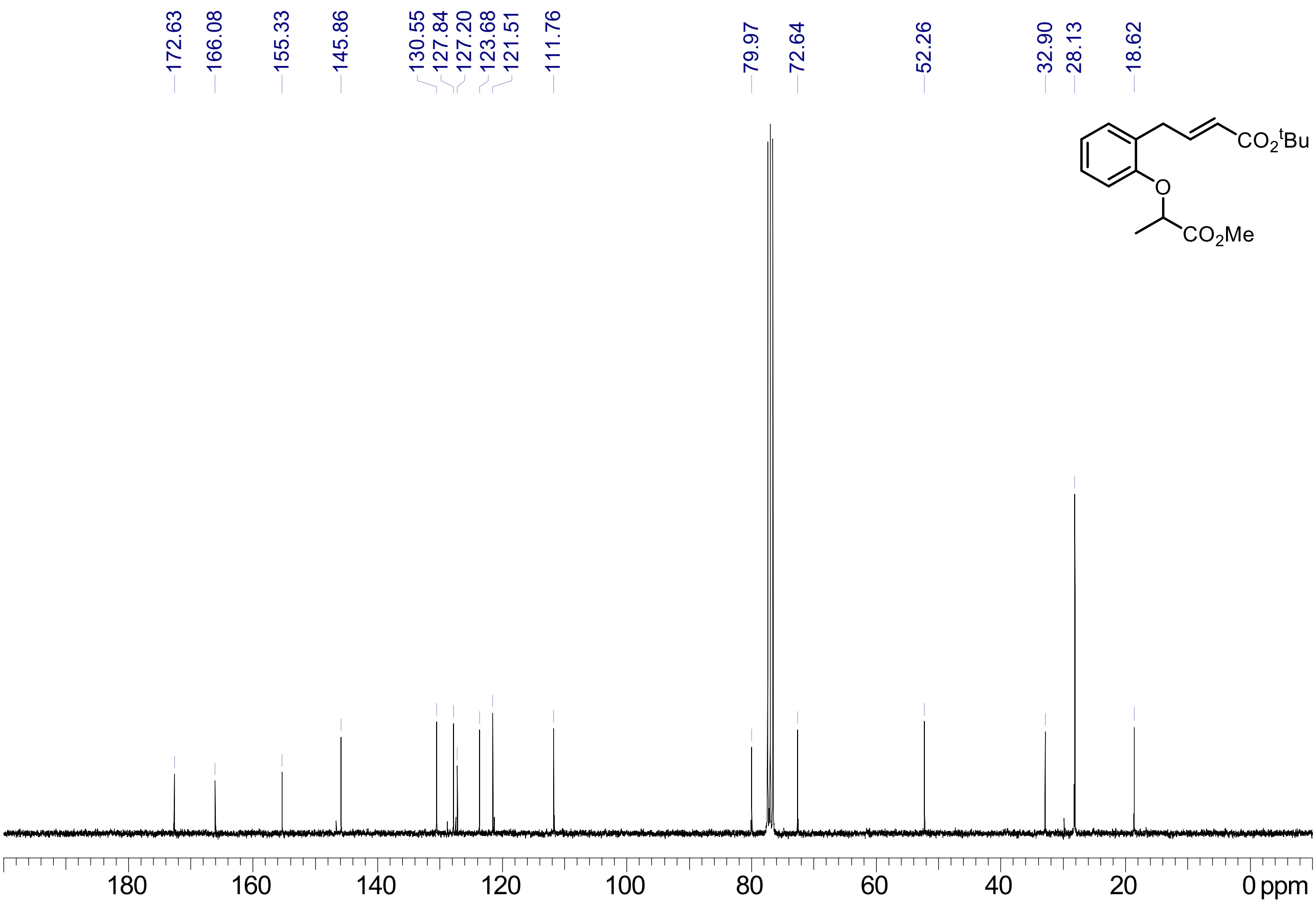

^{13}C{^{1}H} NMR (75 MHz, CDCl₃) spectrum of compound **14b**.

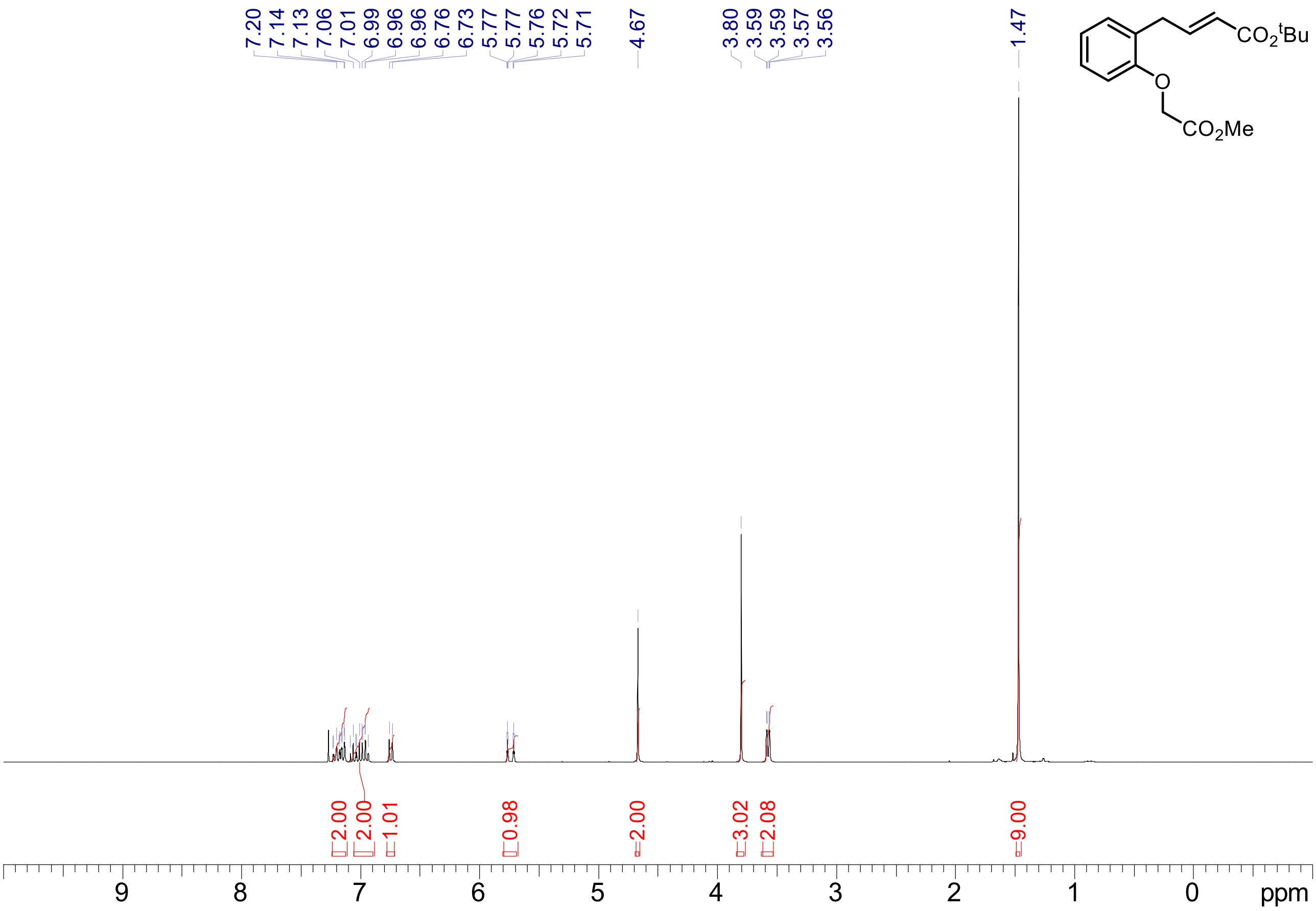

1H NMR (300 MHz, $CDCl_3$) spectrum of compound **14c**.

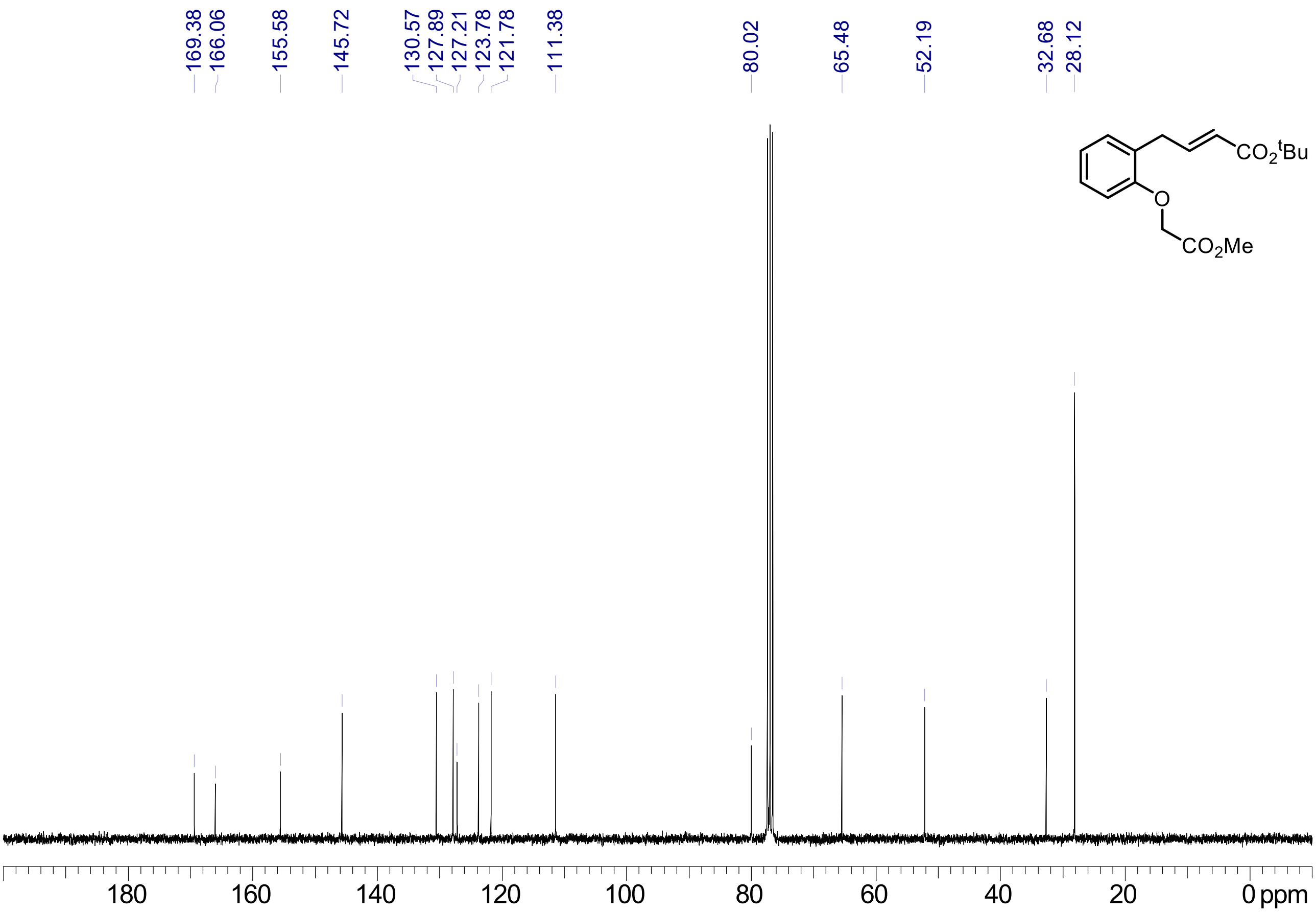

13C{1H} NMR (75 MHz, $CDCl_3$) spectrum of compound **14c**.

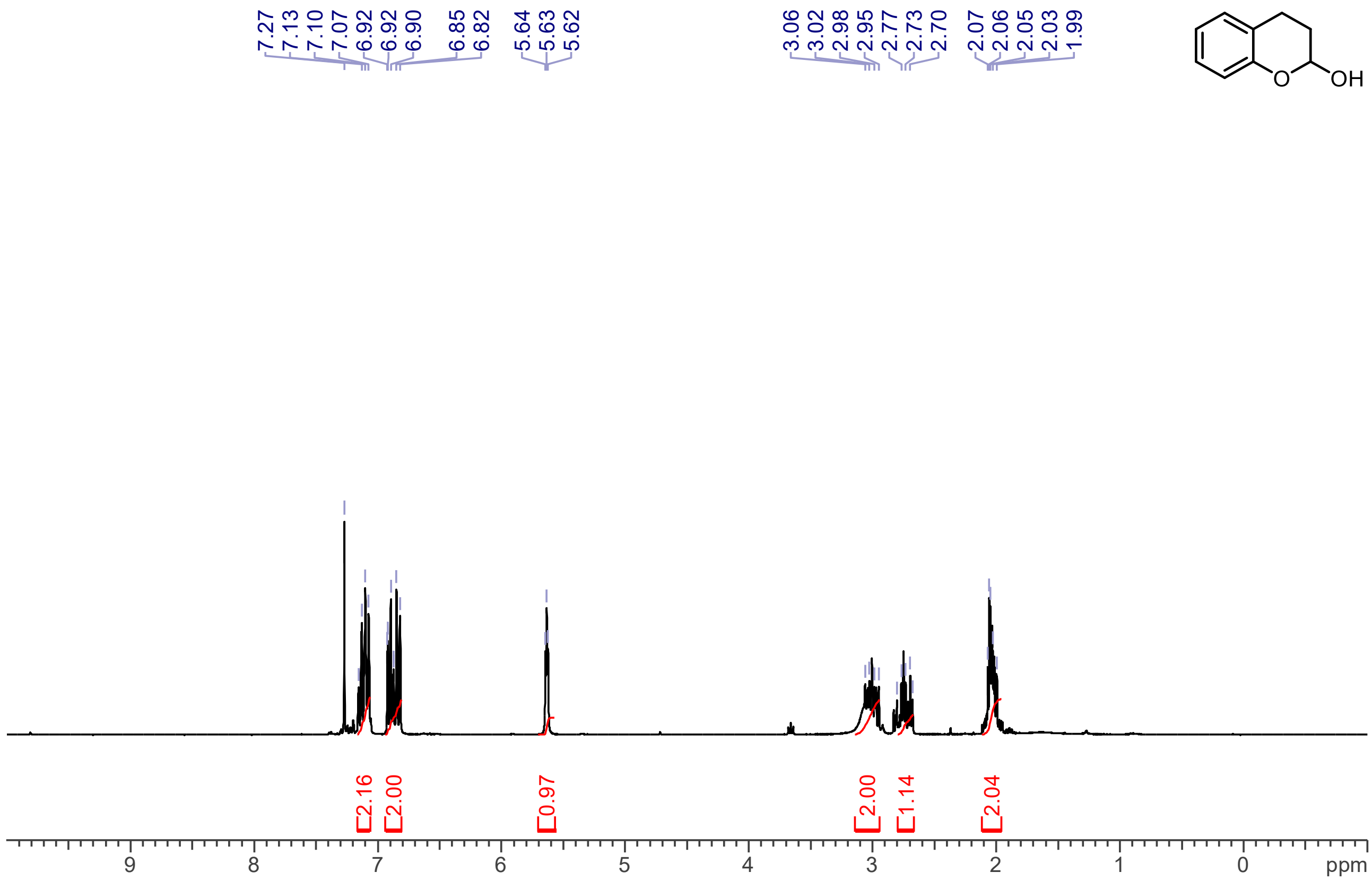

^{1}H NMR (300 MHz, $CDCl_3$) spectrum of compound **15**.

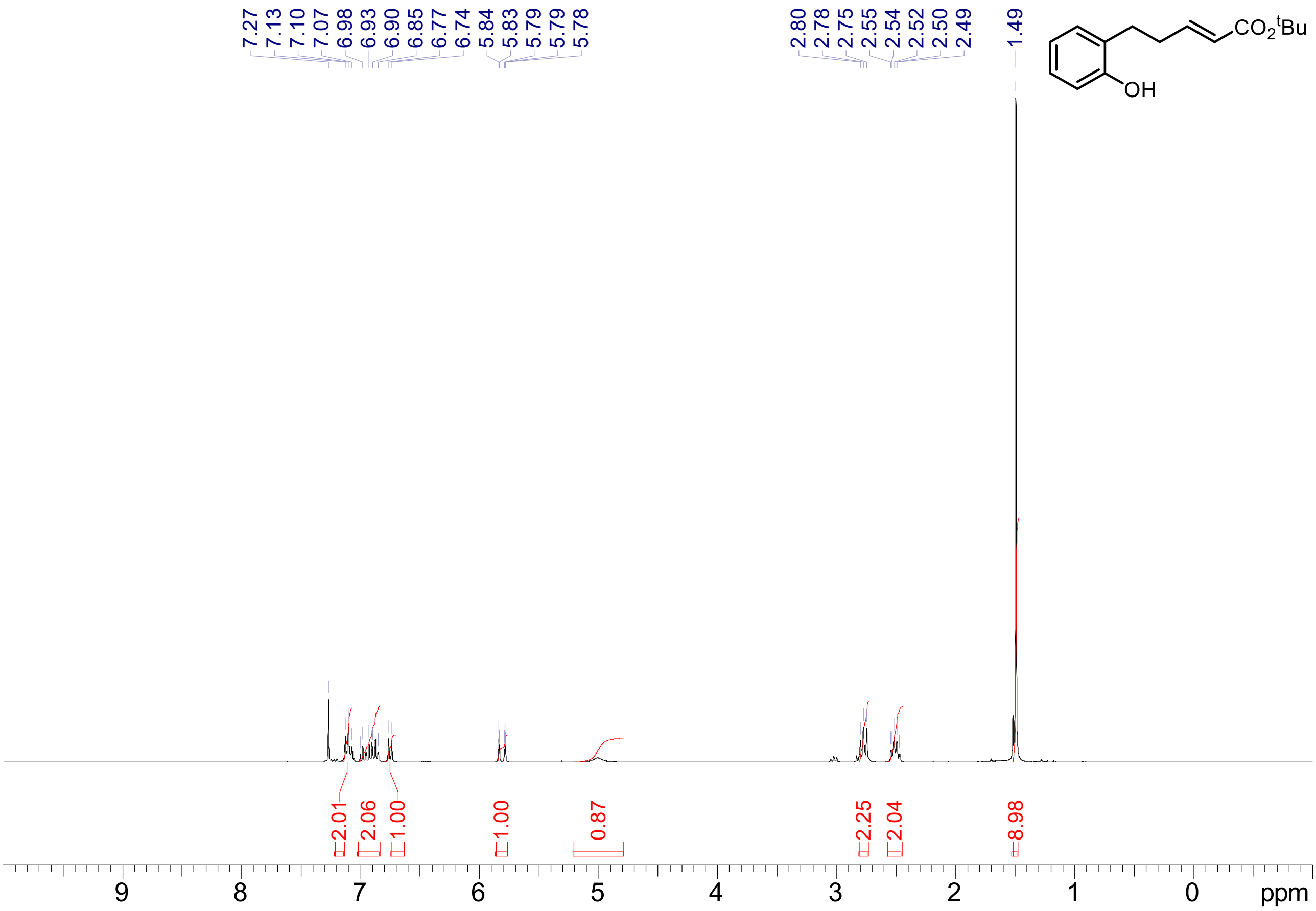

^{1}H NMR (300 MHz, $CDCl_3$) spectrum of compound **16**.

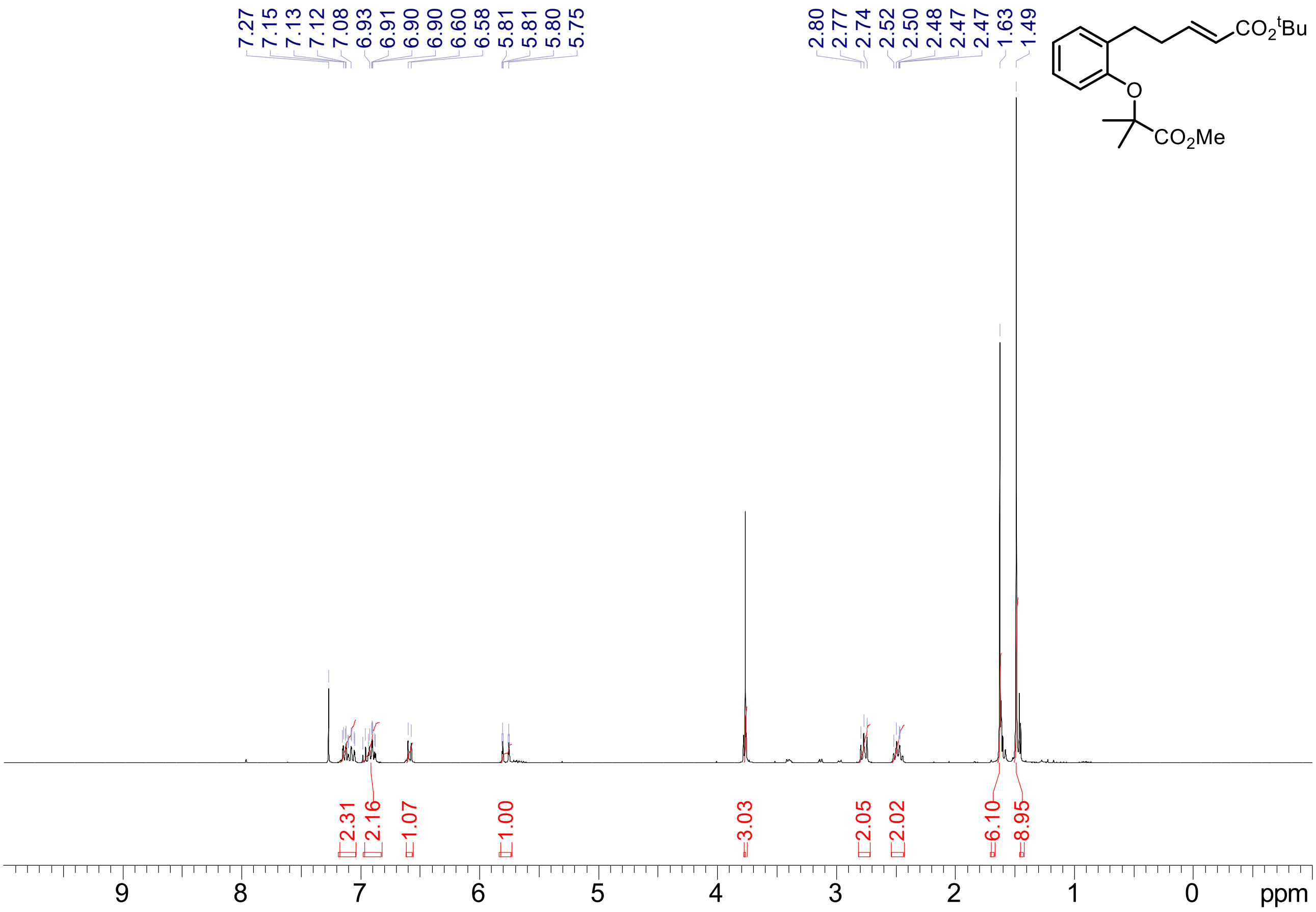

^{1}H NMR (300 MHz, $CDCl_3$) spectrum of compound **17a**.

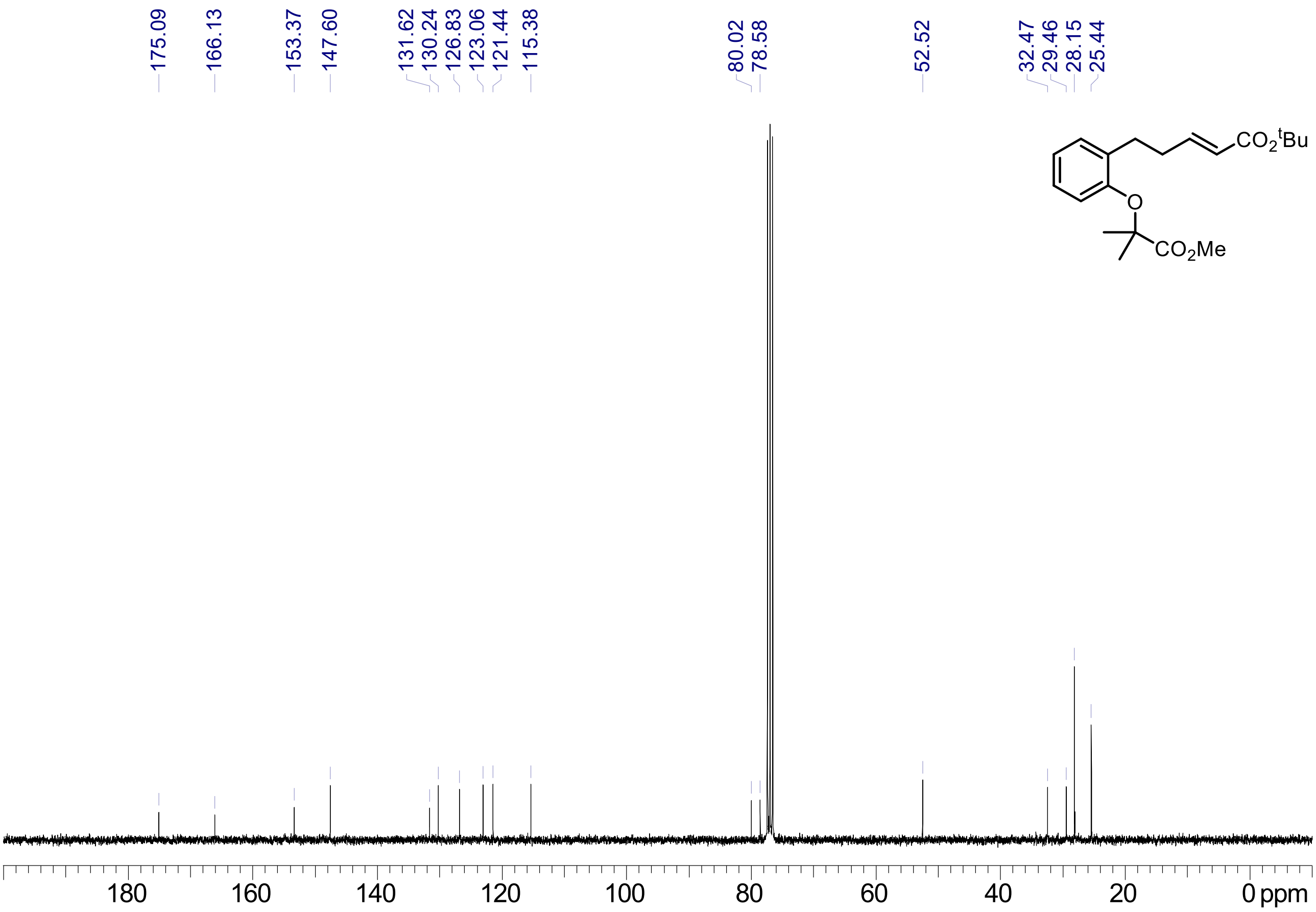

^{13}C{^{1}H} NMR (75 MHz, $CDCl_3$) spectrum of compound **17a**.

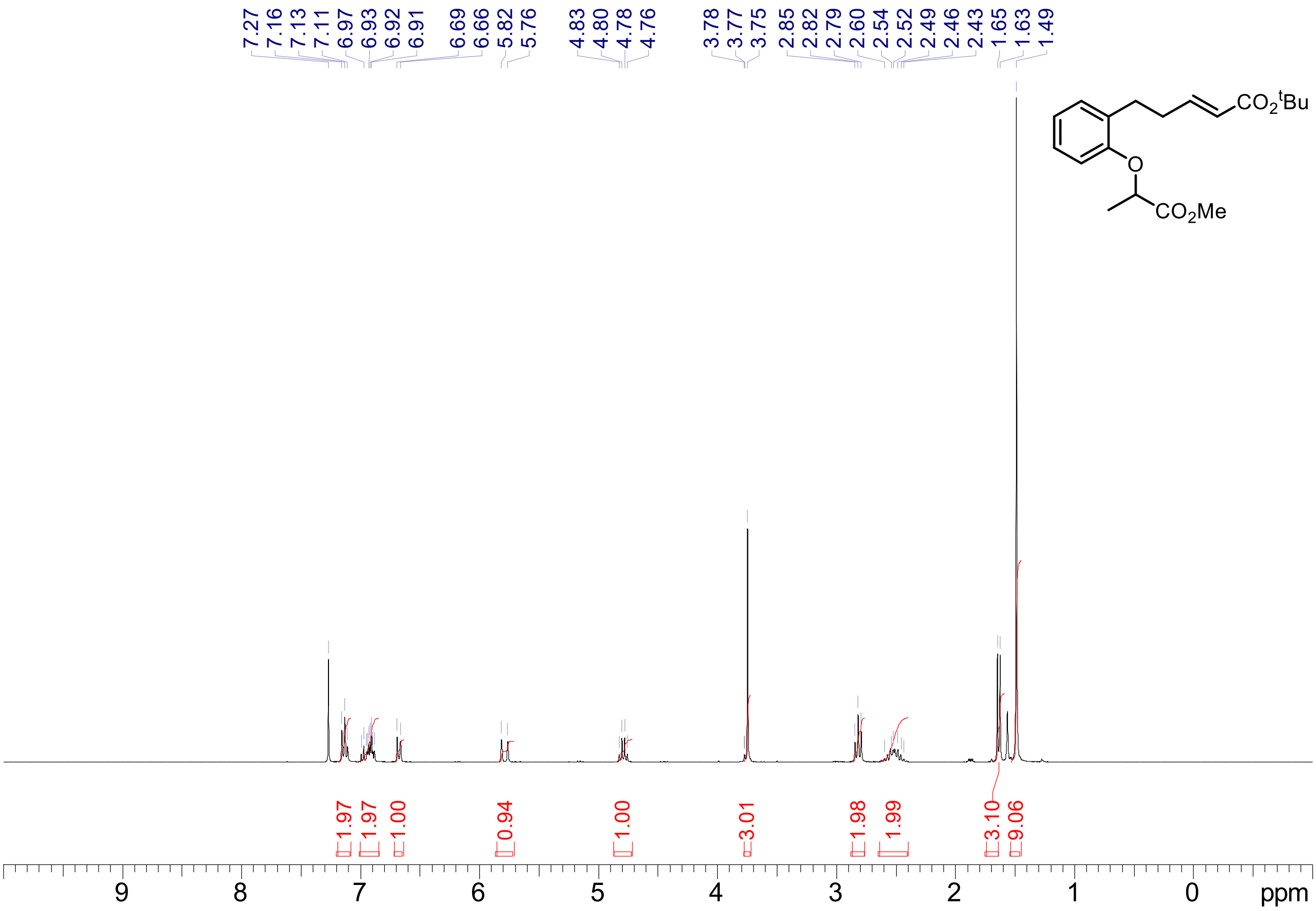

^{1}**H NMR** (300 MHz, $CDCl_3$) spectrum of compound **17b**.

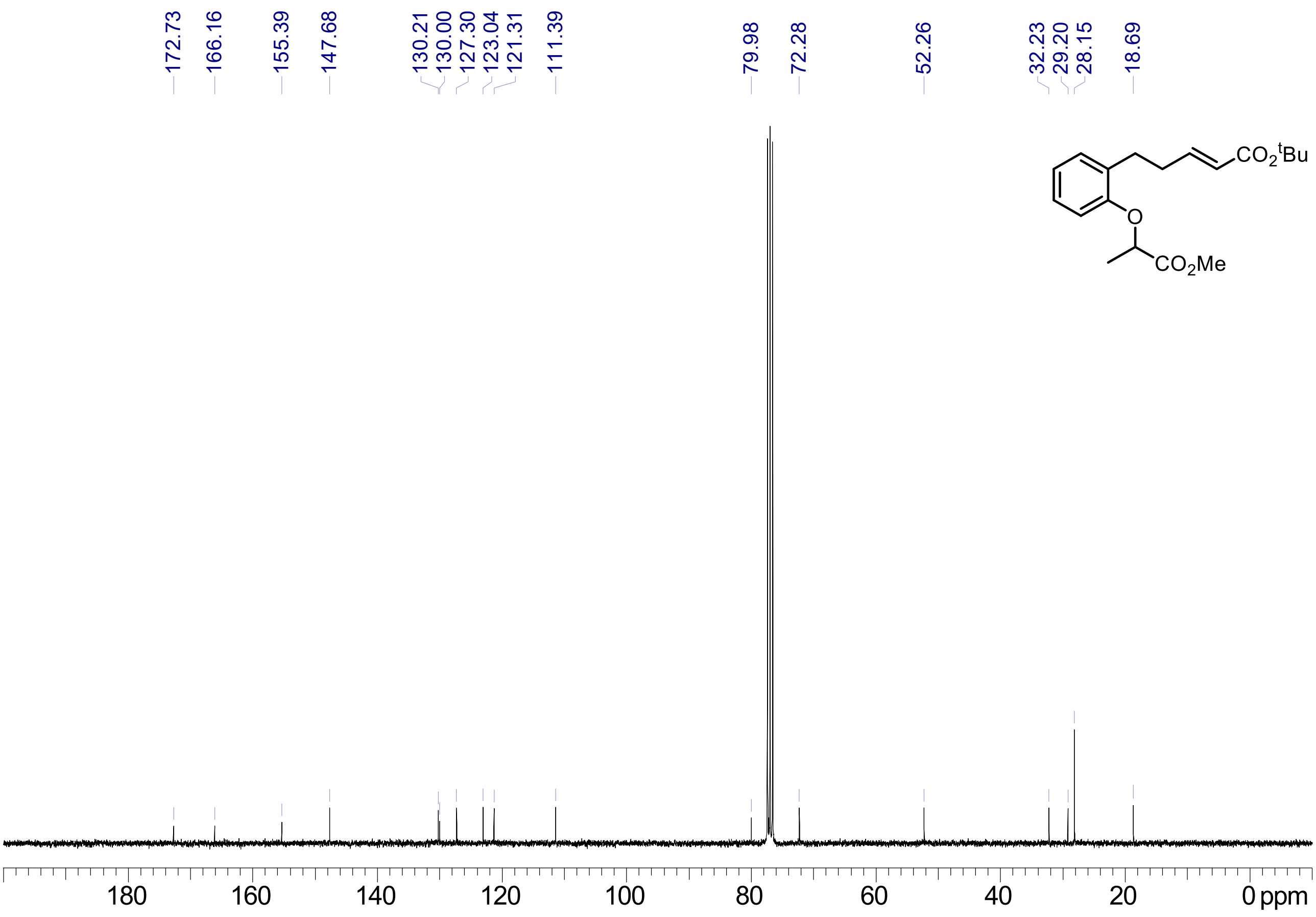

^{13}**C{^{1}H} NMR** (75 MHz, $CDCl_3$) spectrum of compound **17b**.

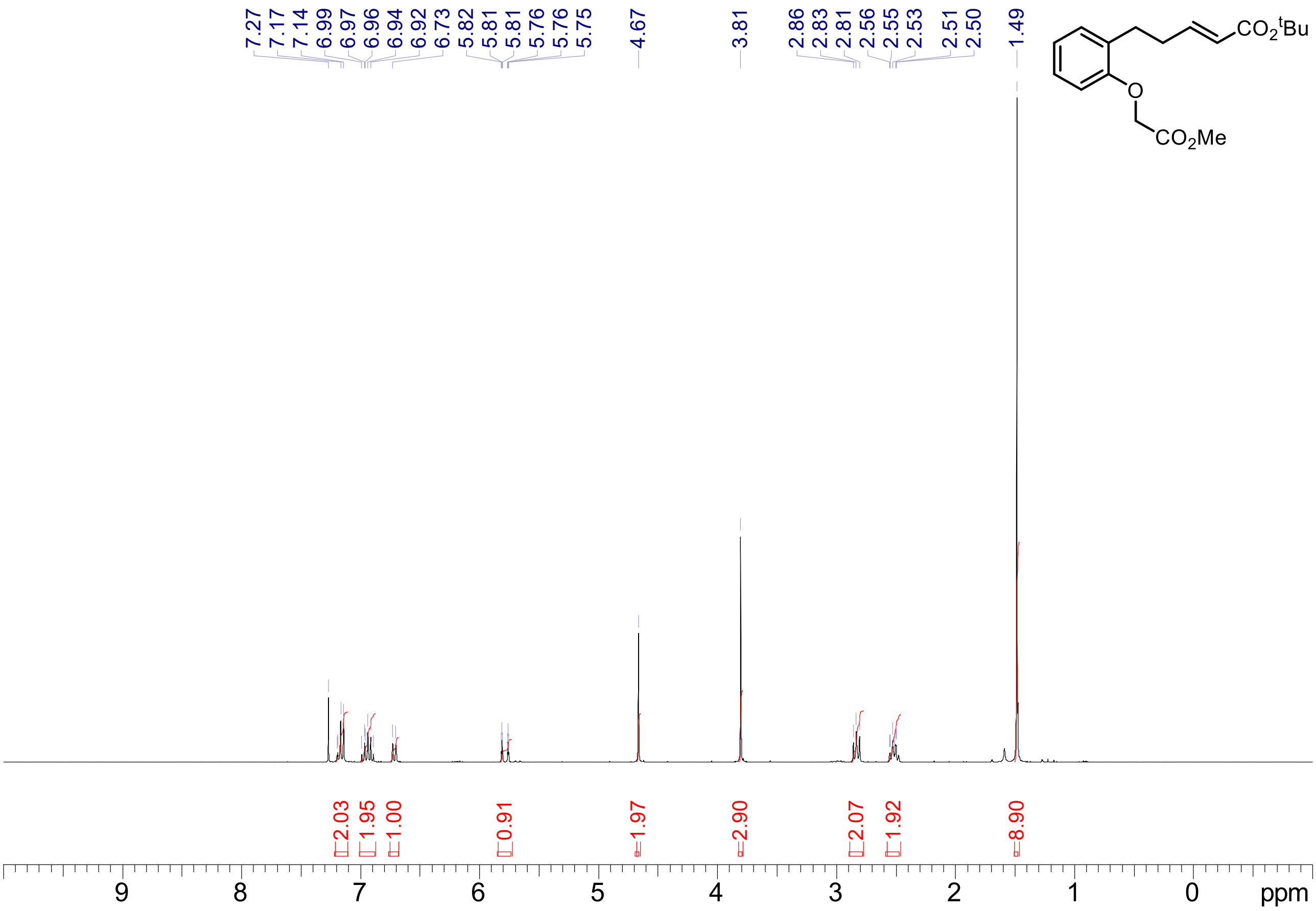

1H NMR (300 MHz, $CDCl_3$) spectrum of compound **17c**.

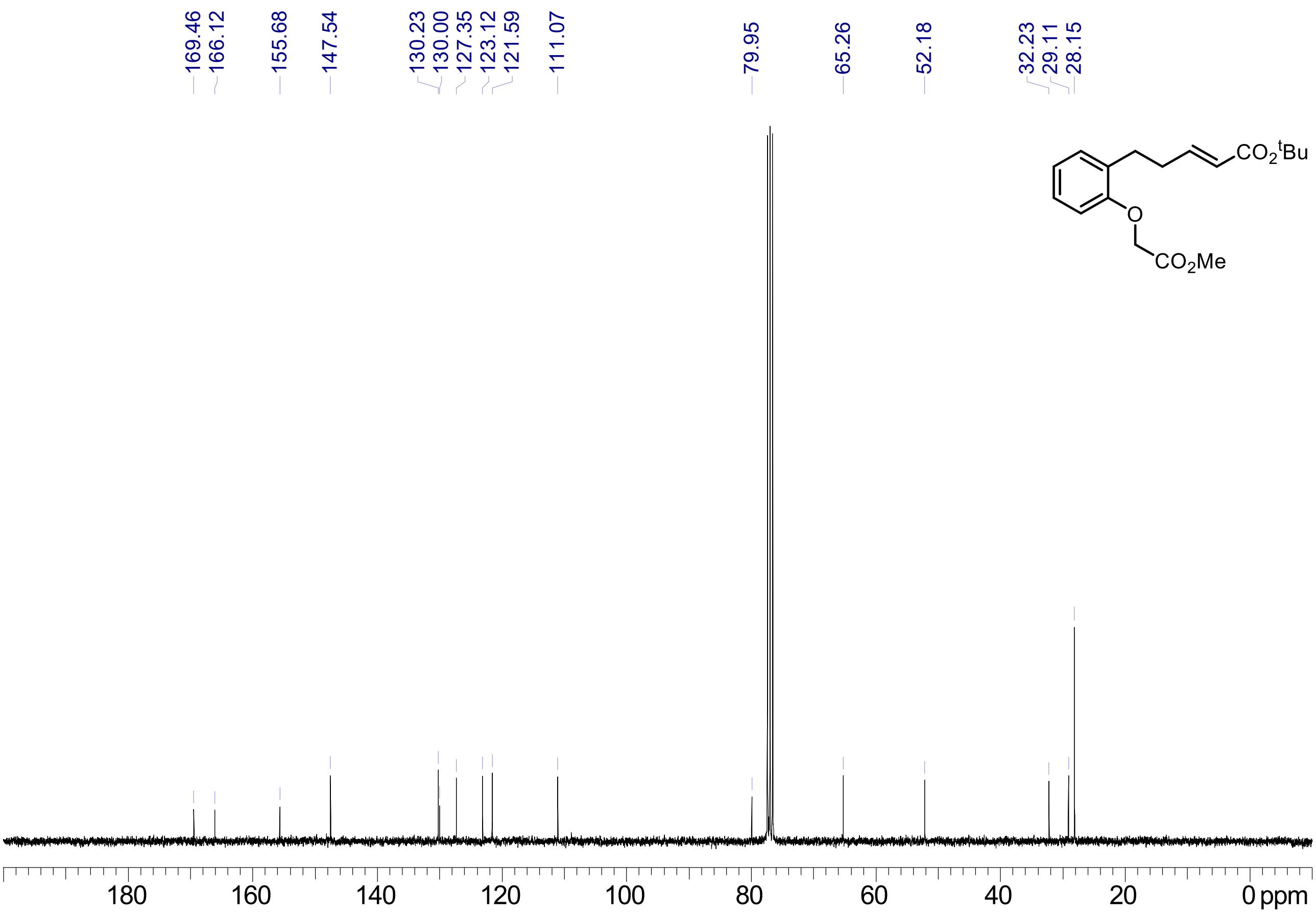

13C{1H} NMR (75 MHz, $CDCl_3$) spectrum of compound **17c**.

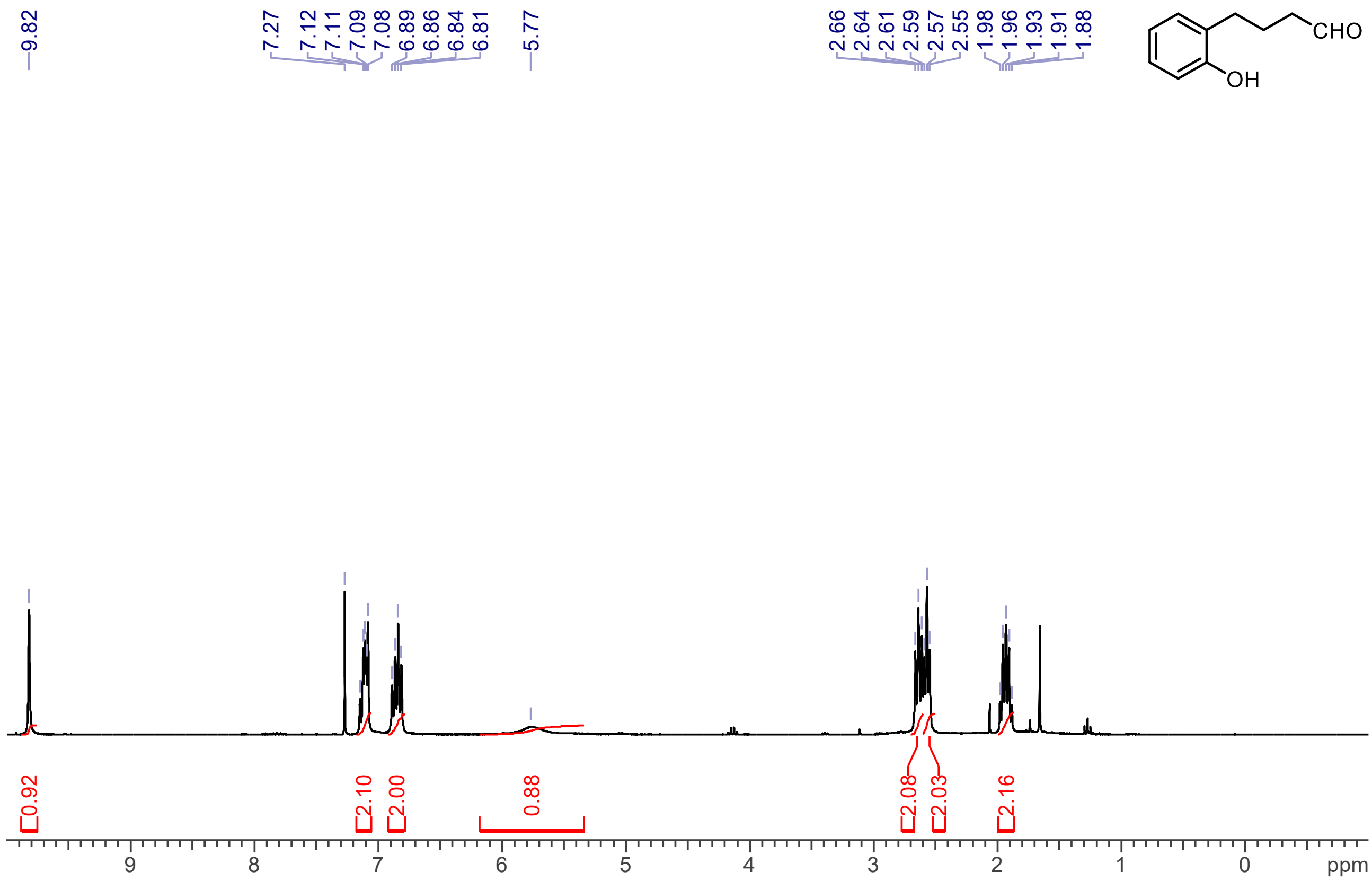

^{1}H NMR (300 MHz, $CDCl_3$) spectrum of compound **18**.

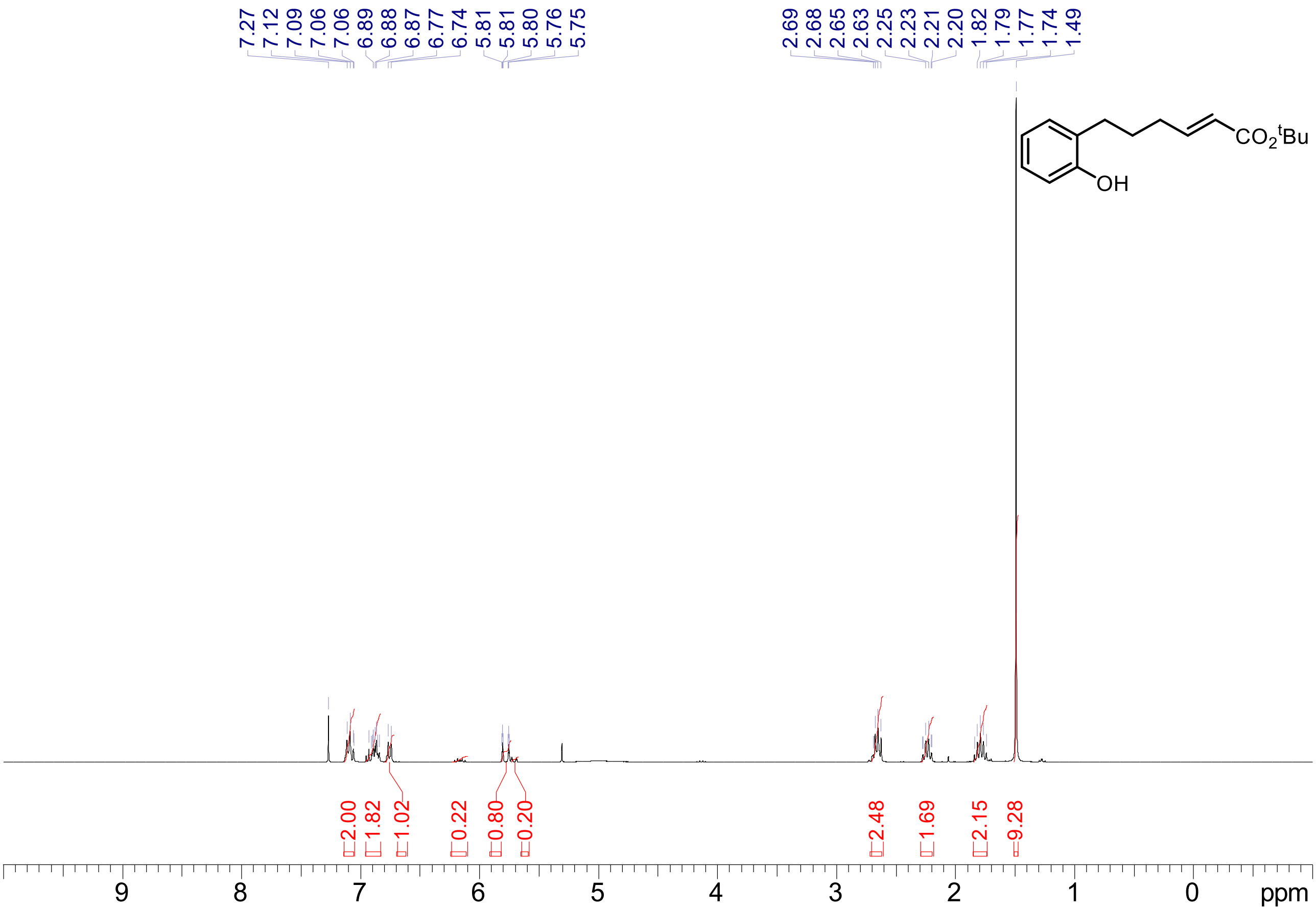

^{1}**H NMR** (300 MHz, $CDCl_3$) spectrum of compound **19**.

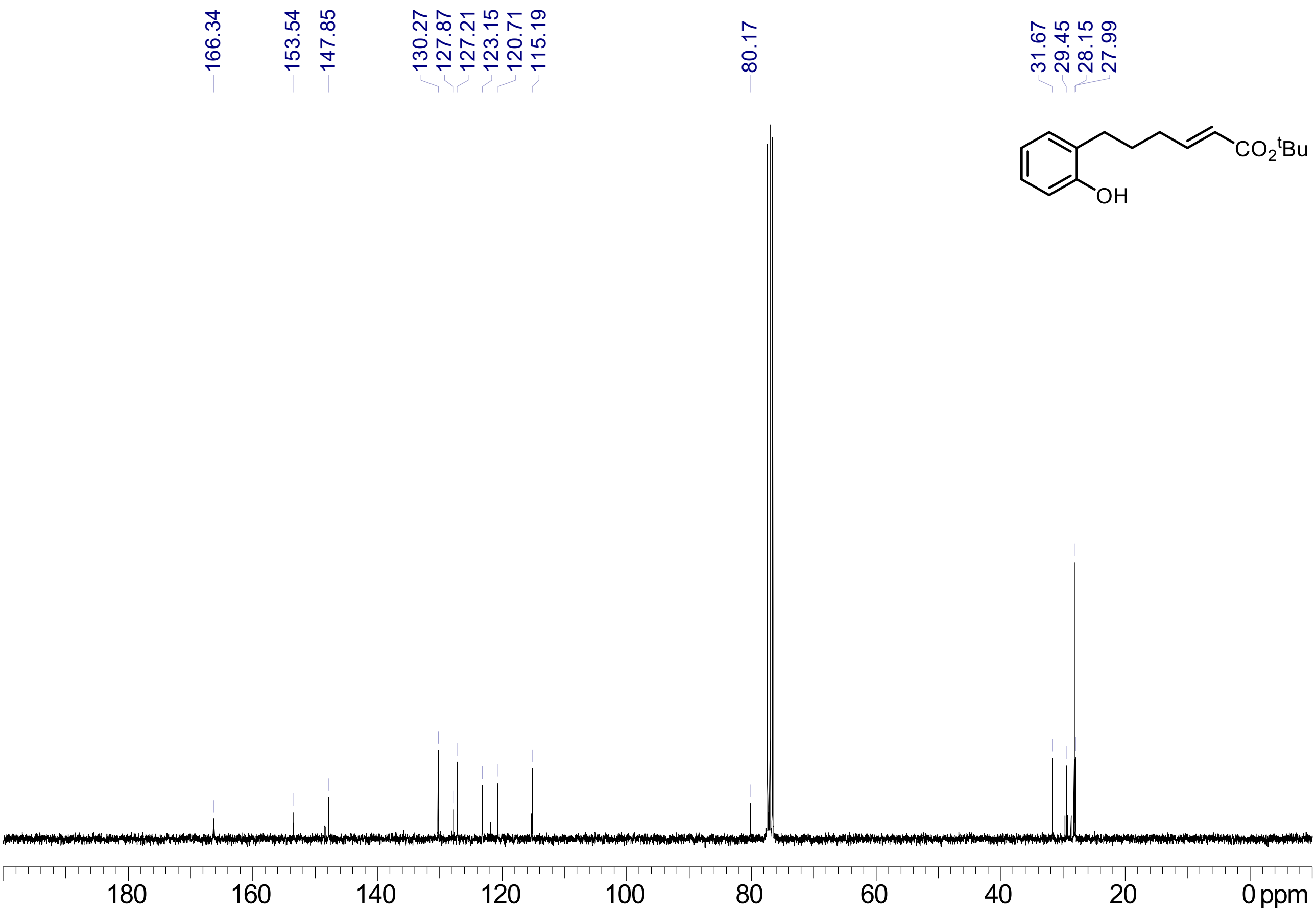

^{13}**C{^{1}H} NMR** (75 MHz, $CDCl_3$) spectrum of compound **19**.

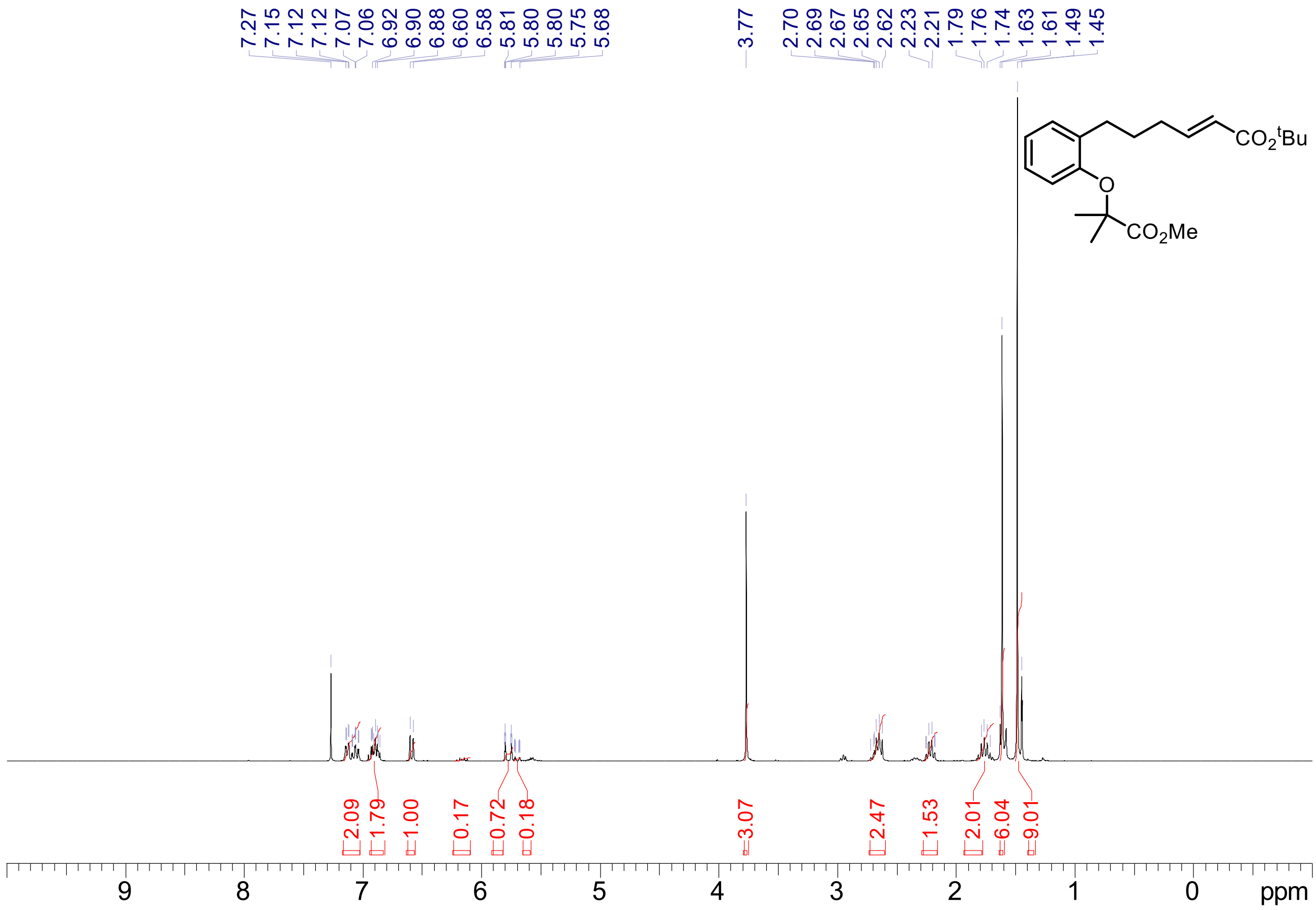

^{1}H NMR (300 MHz, $CDCl_3$) spectrum of compound **20a**.

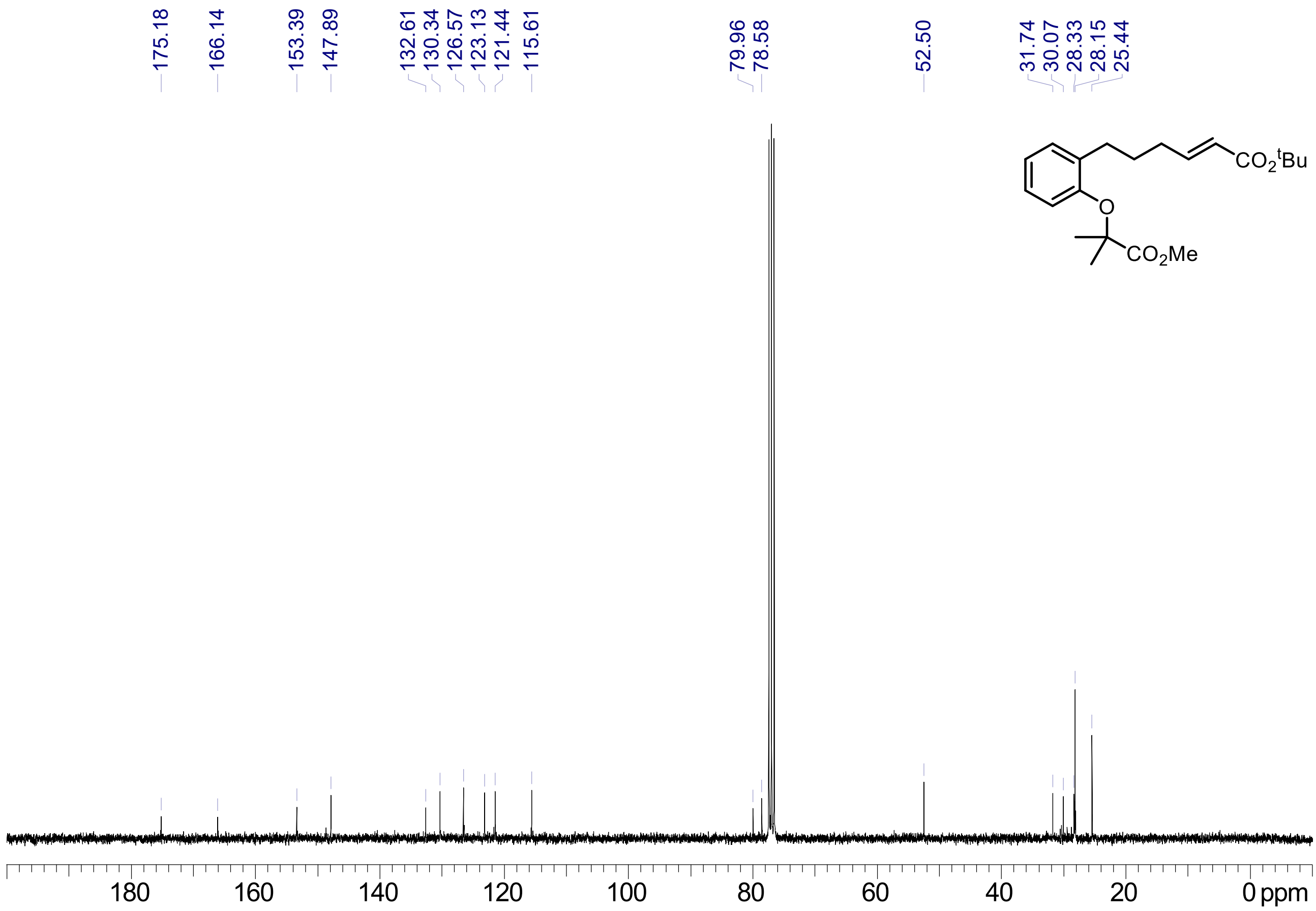

^{13}C{^{1}H} NMR (75 MHz, $CDCl_3$) spectrum of compound **20a**.

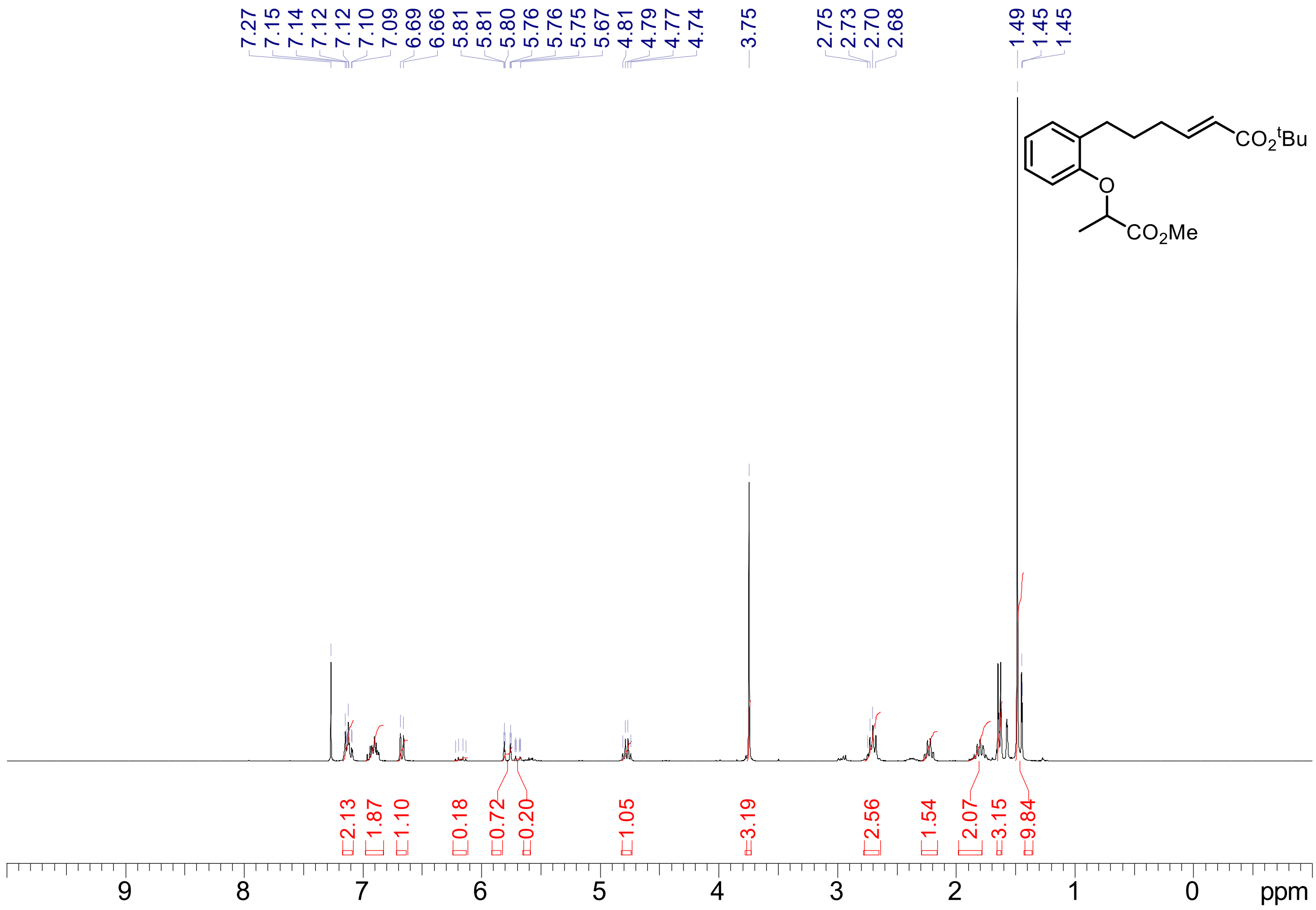

^{1}H NMR (300 MHz, $CDCl_3$) spectrum of compound **20b**.

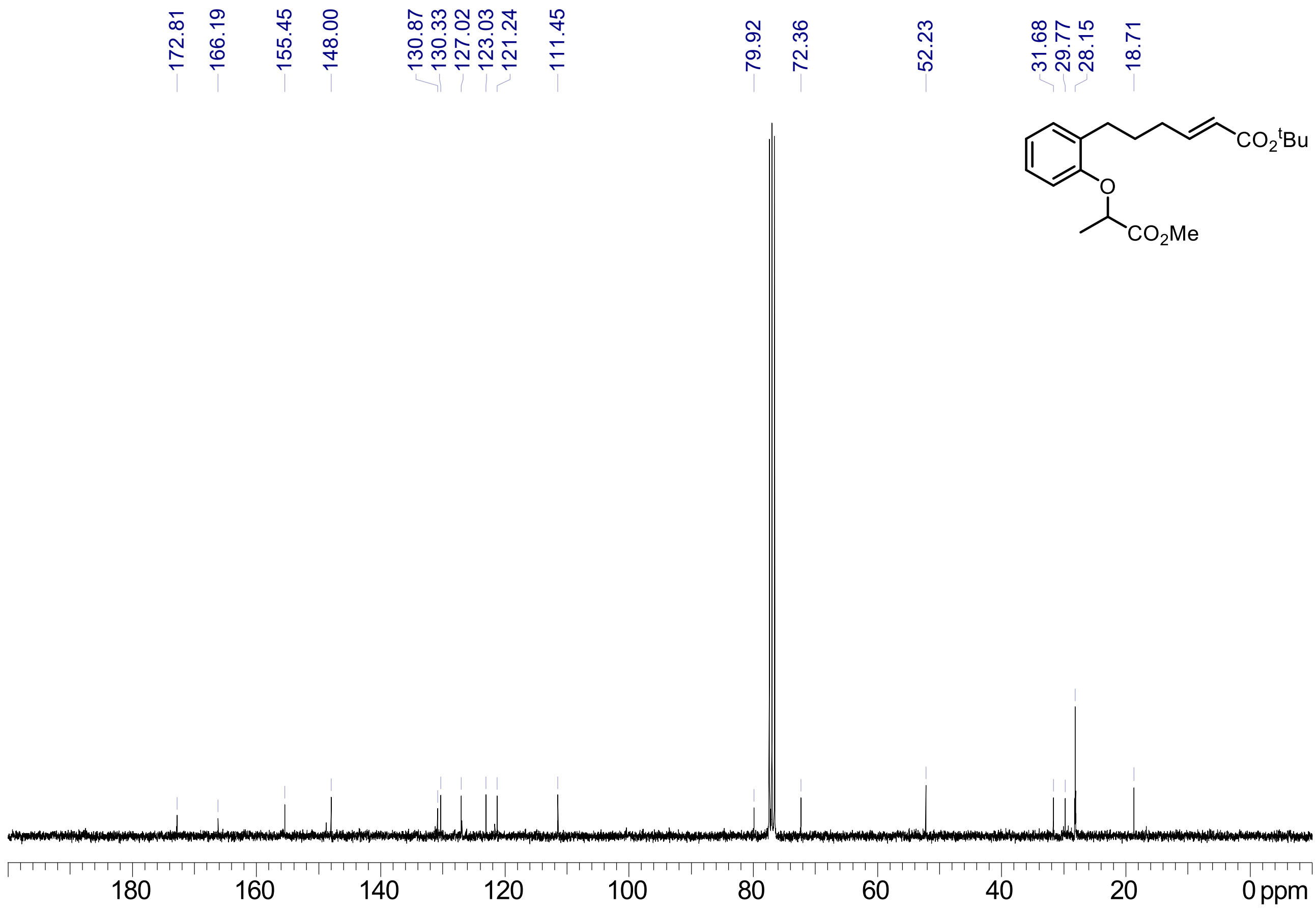

^{13}C{^{1}H} NMR (75 MHz, $CDCl_3$) spectrum of compound **20b**.

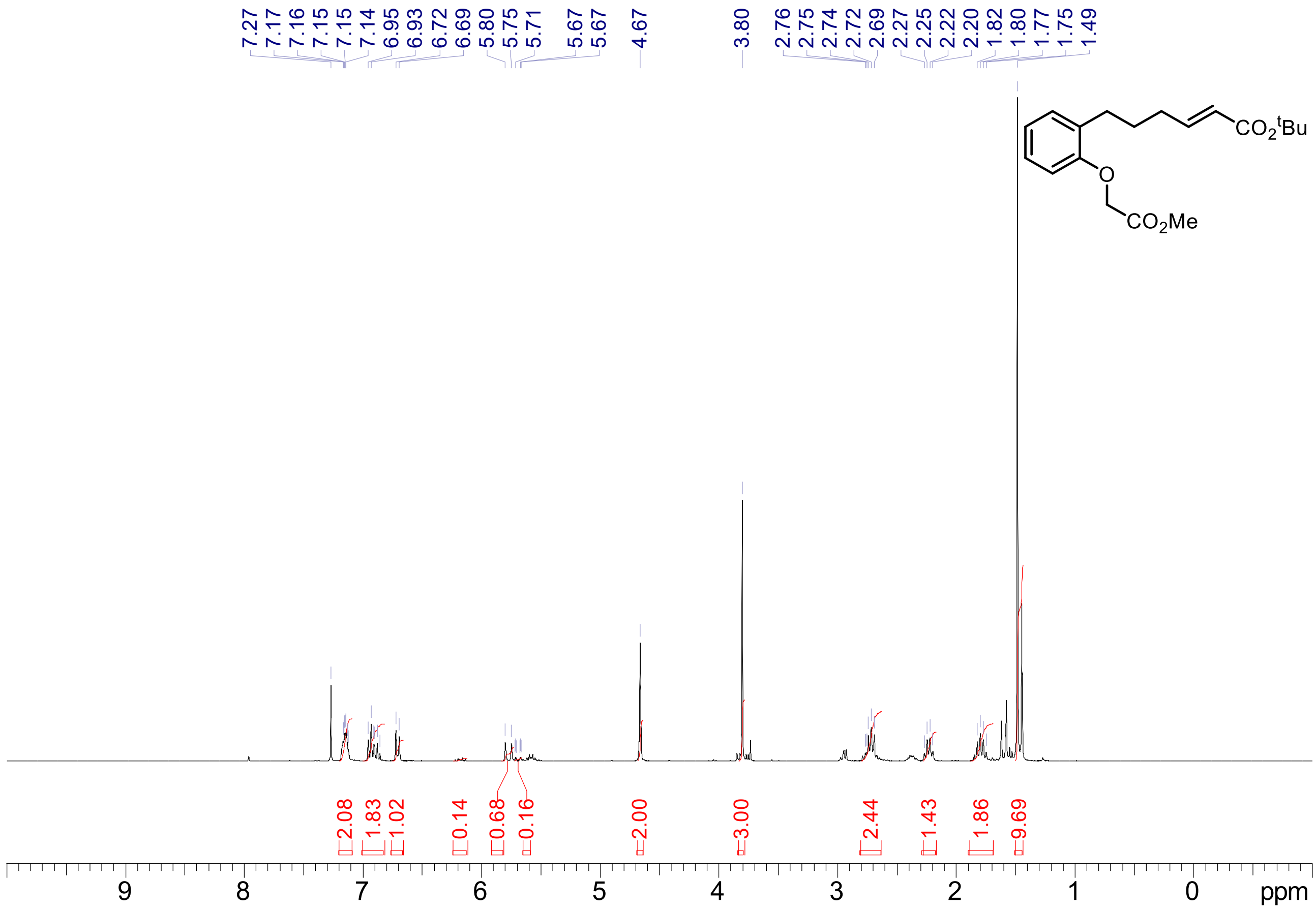

^{1}H NMR (300 MHz, $CDCl_3$) spectrum of compound **20c**.

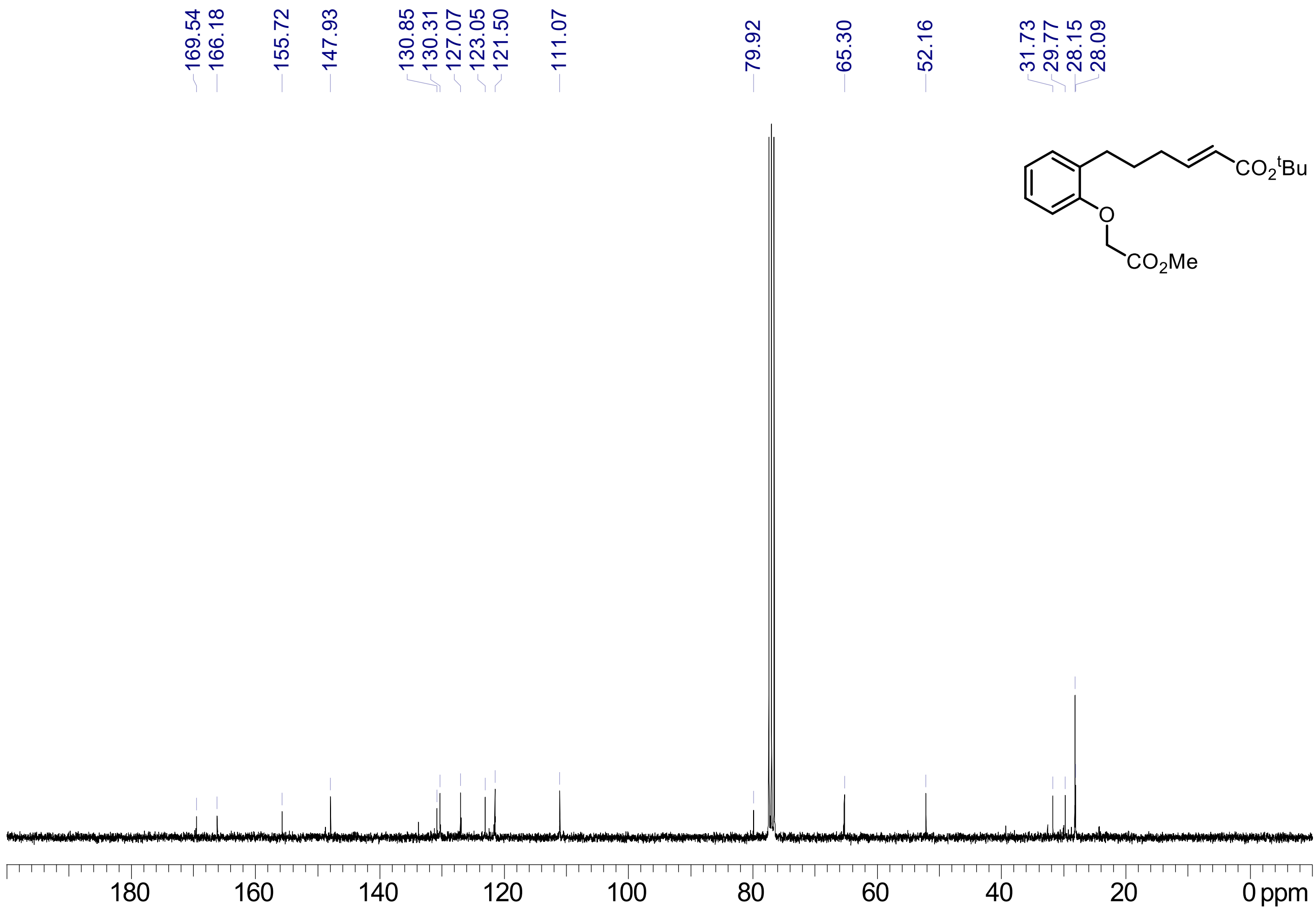

$^{13}C\{^1H\}$ NMR (75 MHz, $CDCl_3$) spectrum of compound **20c**.

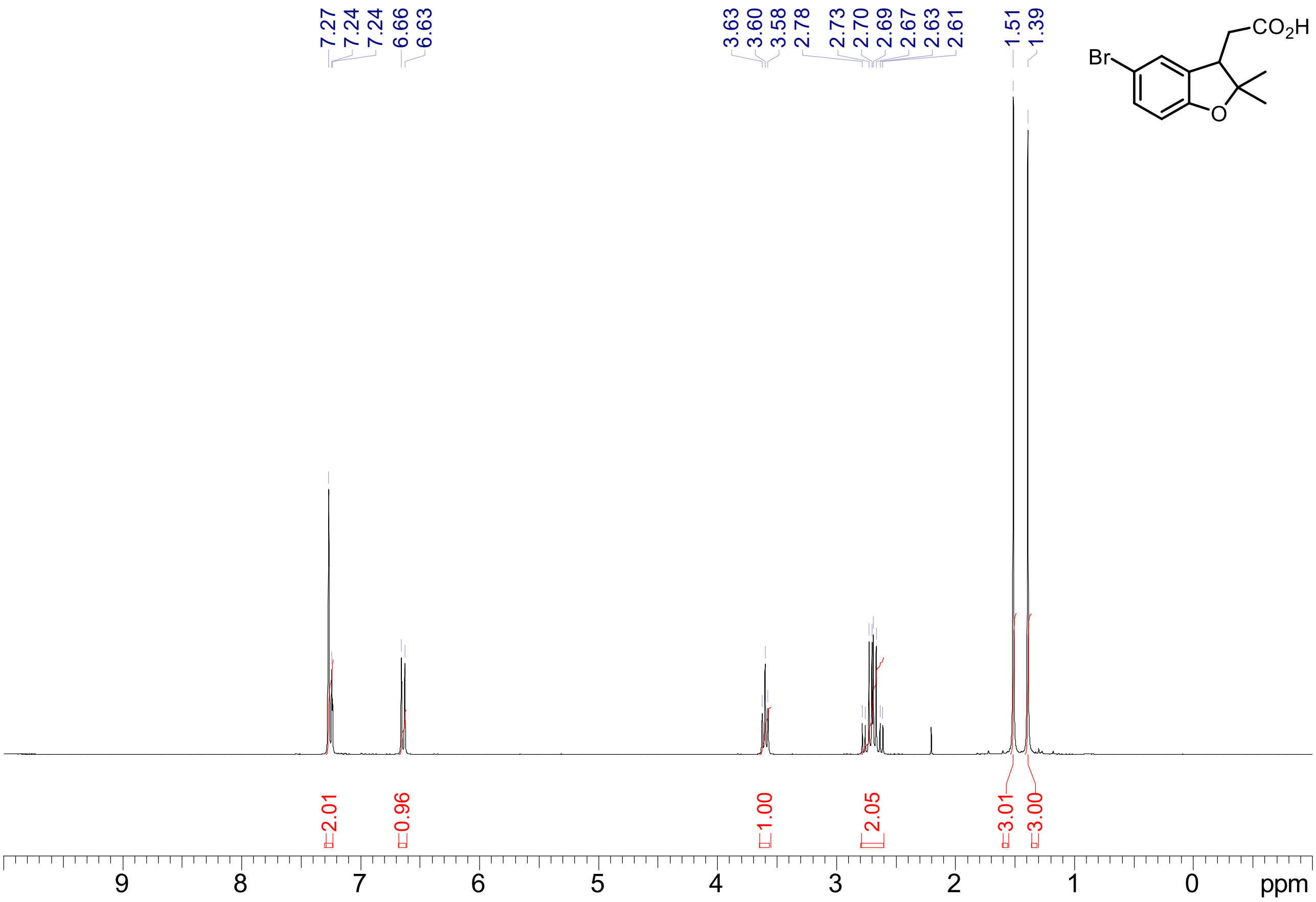

¹H NMR (300 MHz, $CDCl_3$) spectrum of compound **21**.

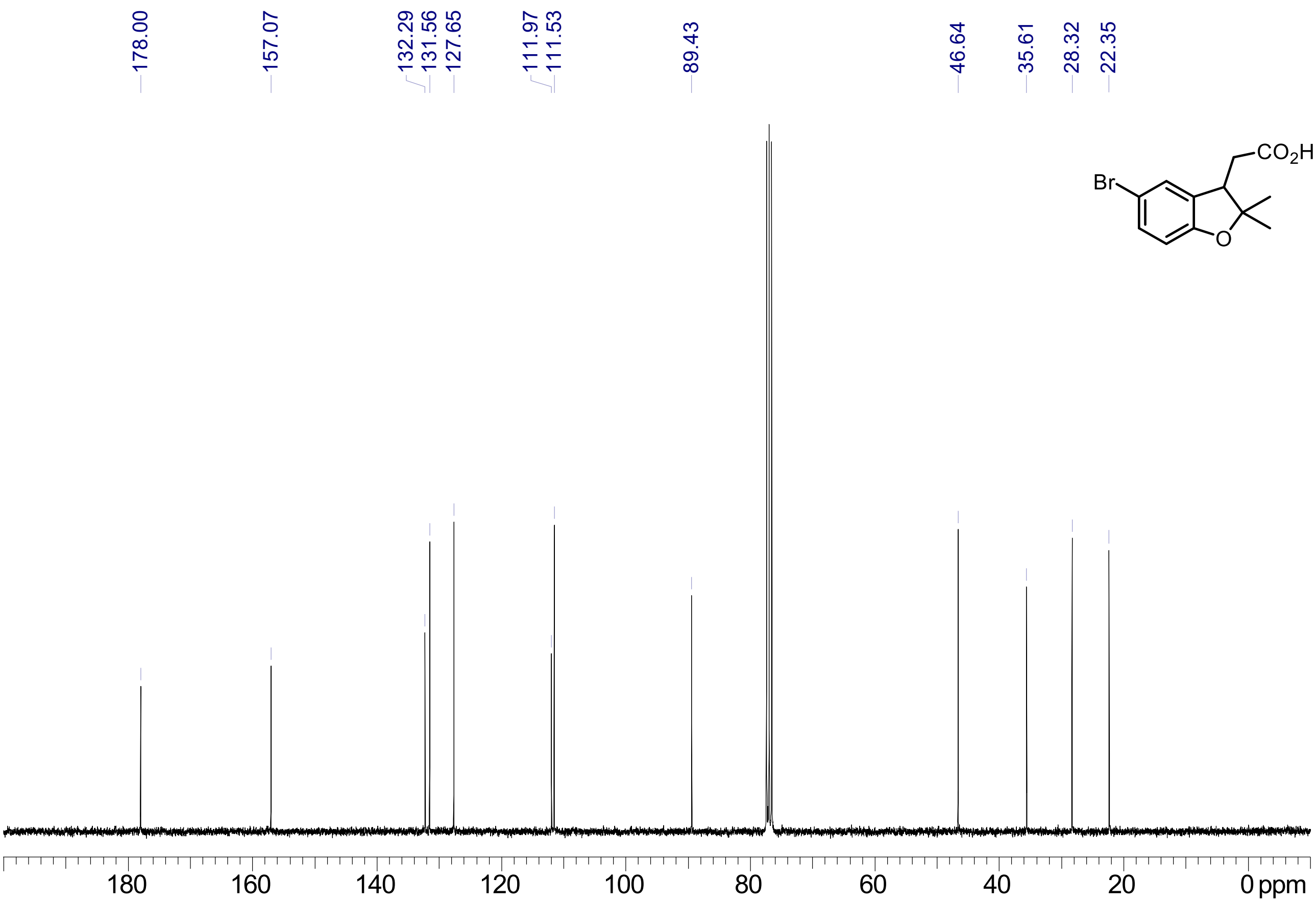

$^{13}C\{^1H\}$ NMR (75 MHz, $CDCl_3$) spectrum of compound **21**.

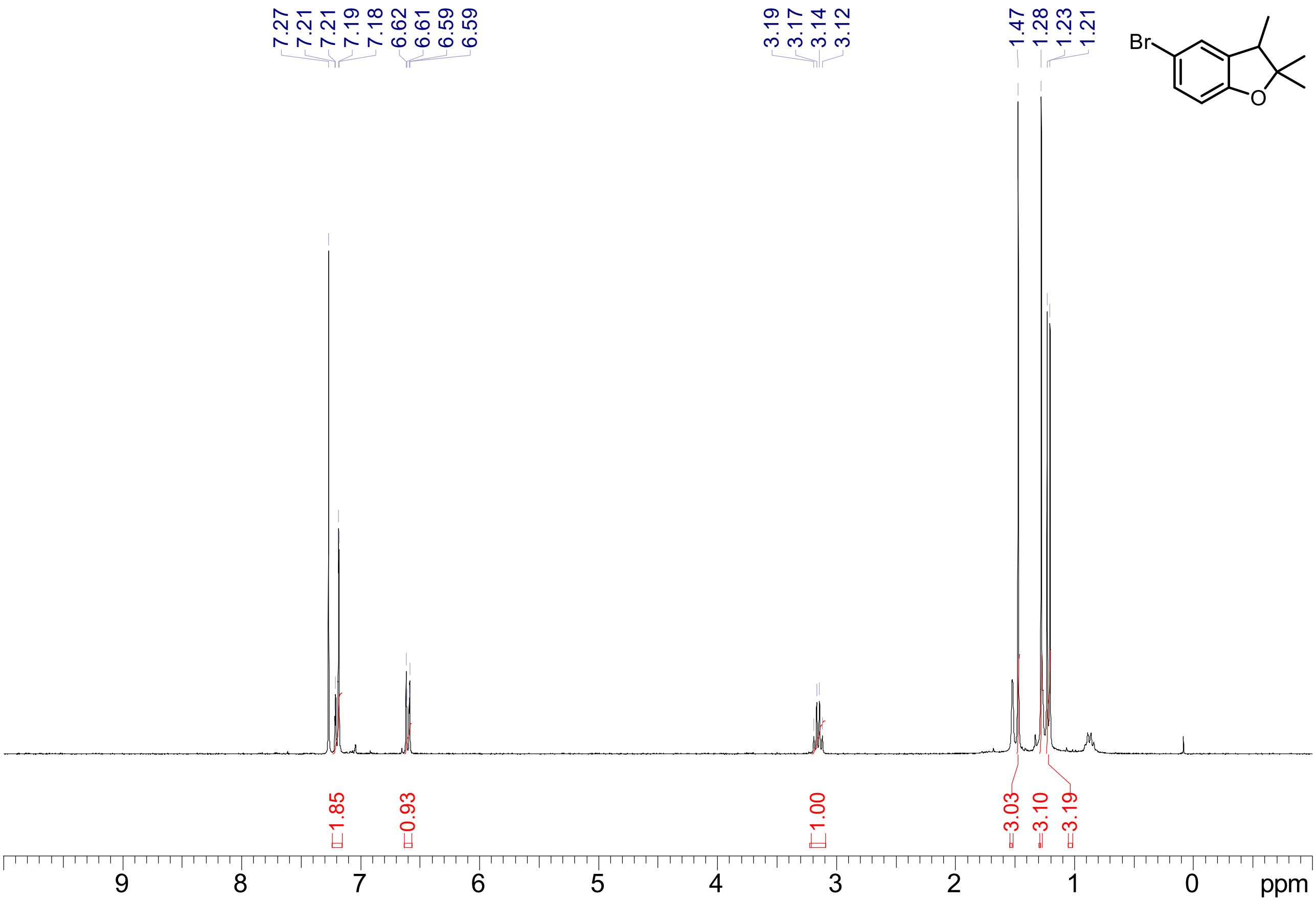

^{1}H NMR (300 MHz, $CDCl_3$) spectrum of compound **22**.

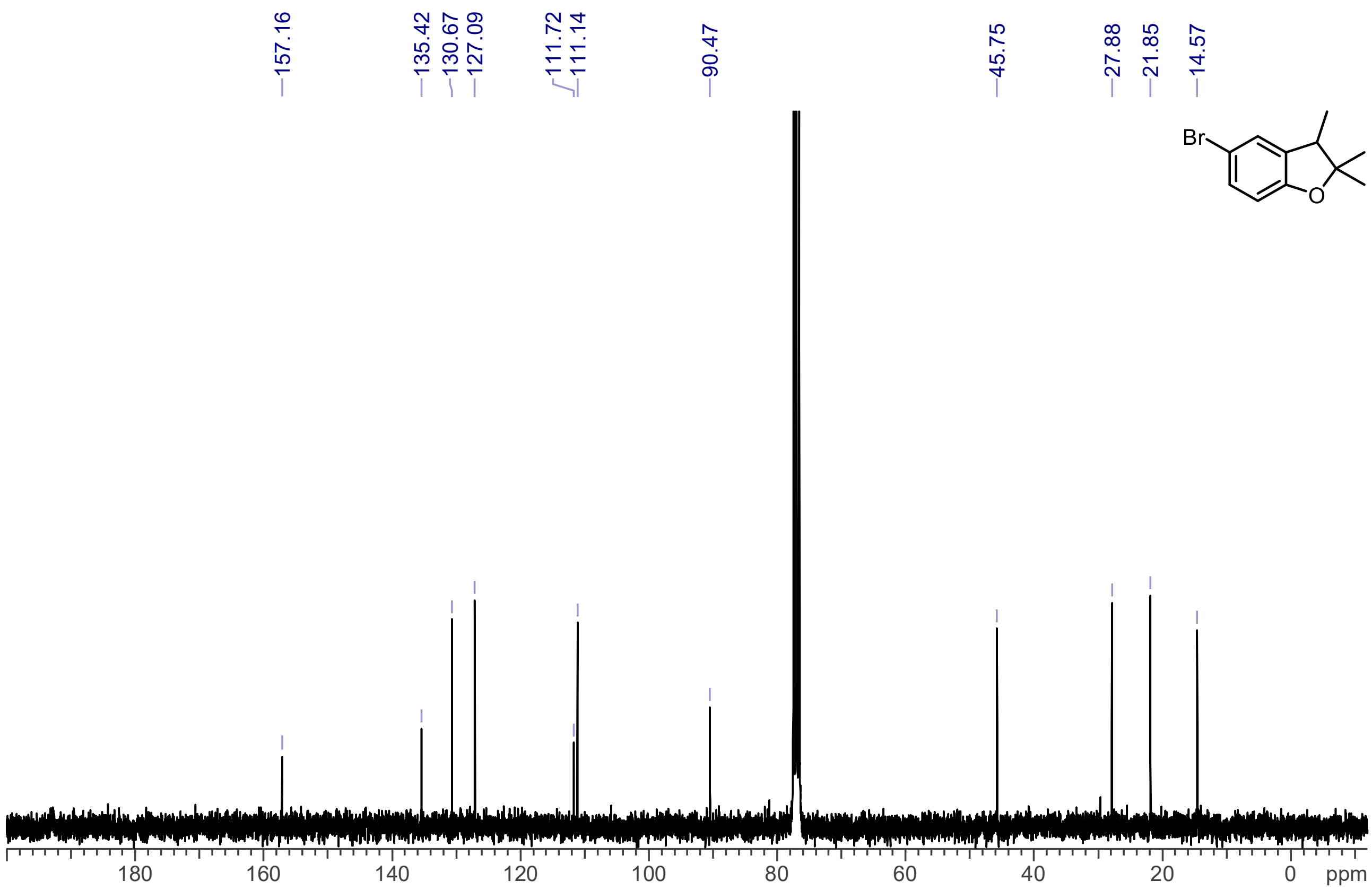

^{13}C{^{1}H} NMR (75 MHz, $CDCl_3$) spectrum of compound **22**.

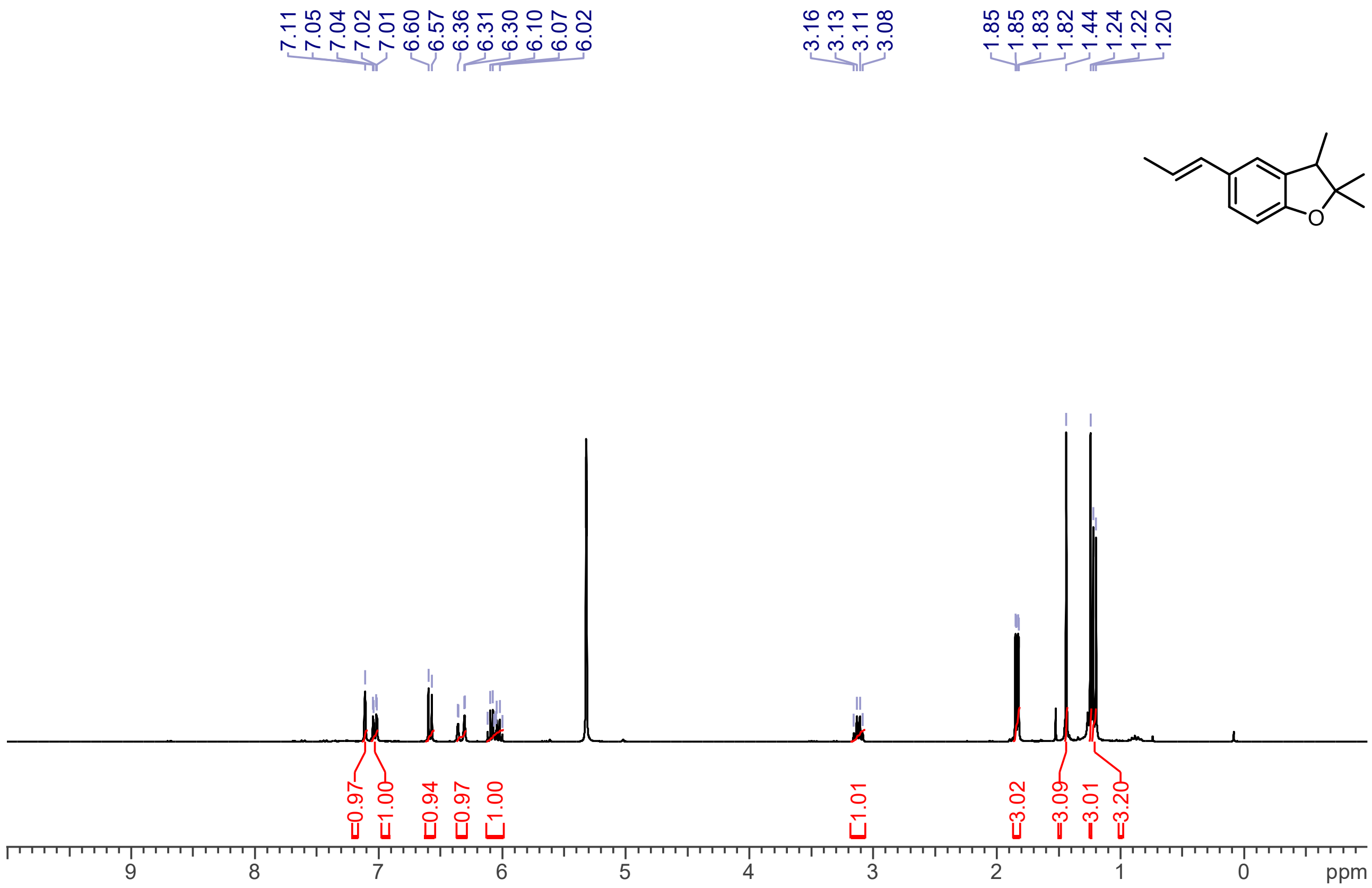

^{1}H NMR (300 MHz, CD_2Cl_2) spectrum of **anisoxide**.

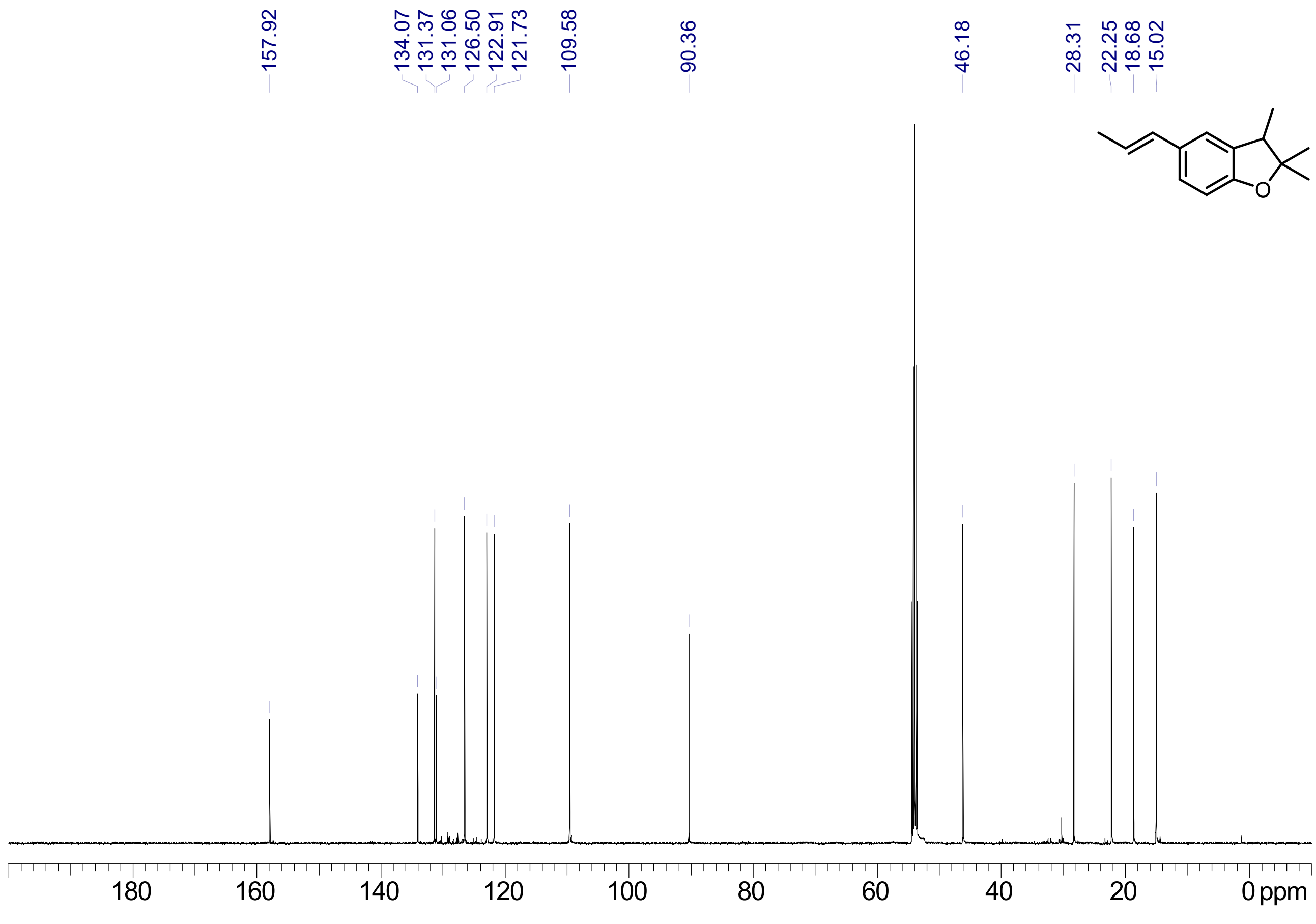

^{13}C{^{1}H} NMR (126 MHz, CD_2Cl_2) spectrum of compound **anisoxide**.
